# Abstracts From AIDS 2026, the 26th International AIDS Conference, 26 – 31 July 2026 Rio de Janeiro, Brazil & virtual

**DOI:** 10.1002/jia2.70125

**Published:** 2026-07-27

## Oral Abstracts

## OAA1002 Senescence‐Associated Degradation of Intestinal α1,2‐Fucose, a Host‐Intrinsic Prebiotic Glycan, Promotes Microbial Dysbiosis and Gut Dysfunction in People With HIV

L. Bertoni Giron ^1^, M. W. Shaikh^2^, T. M. Cantu Jungles^3^, P. A. Engen^2^, S. Shankaran^4^, M. Villanueva^2^, A. Landay^5^, F. J. Palella^1^, M. Corley^6^, B. Hamaker^3^, R. Lorenzo Redondo^1^, A. Keshavarzian^2,4^, M. Abdel‐Mohsen^1^

^1^Northwestern University, Medicine, Chicago, United States; ^2^Rush University Medical Center, Rush Center for Integrated Microbiome and Chronobiology Research, Chicago, United States; ^3^Purdue University, West Lafayette, United States; ^4^Rush University Medical Center, Department of Internal Medicine, Chicago, United States; ^5^University of Texas Medical Branch, Galveston, United States; ^6^University of California San Diego, San Diego, United States

**Background**: Despite antiretroviral therapy (ART), people with HIV (PWH) exhibit persistent microbial dysbiosis, including depletion of bacteria that produce short‐chain fatty acids (SCFAs), metabolites essential for intestinal barrier integrity and resilience to stress. Loss of epithelial α1,2‐fucose has recently emerged as a potentially targetable upstream mechanism driving the depletion of SCFA‐producing bacteria during disease. This host‐intrinsic prebiotic glycan fuels commensals to produce SCFAs. When α1,2‐fucose is degraded by glycan‐degrading enzymes (α‐L‐fucosidases) released by senescent cells, the microbiota is deprived of this substrate, causing dysbiosis with reduced SCFA output. We tested whether α1,2‐fucose depletion contributes to HIV‐associated SCFA depletion and intestinal dysfunction.

**Methods**: Ileal and colonic biopsies were obtained from PWH on ART (*n* = 25) and matched people without HIV (PWoH; *n* = 23). Epithelial α1,2‐fucosylation was quantified by lectin‐based glycomic profiling. α‐L‐fucosidase expression and senescence/stress signatures were evaluated by spatial transcriptomics. Mucosal microbiota was profiled by 16S rRNA gene‐amplicon sequencing. Plasma microbial translocation and inflammation markers were quantified using MesoScale multiplex assays. Biological ageing was assessed using epigenetic clocks. To test functional microbiome consequences of differential α1,2‐fucose levels, anaerobic stool fermentations from PWH and PWoH were performed with or without exogenous α1,2‐fucose provided as a clinically safe supplement (2′‐fucosyllactose; 2′FL). SCFAs were quantified by mass‐spectrometry in fermentation supernatants, which were applied to 3D intestinal organoids during stress challenge.

**Results**: Intestinal tissues from PWH exhibited significantly lower α1,2‐fucosylation than PWoH (*p* < 0.05; Figure 1). This reduction correlated with increased expression of α‐L‐fucosidase (*p* = 9.3e‐08) and increased senescence (*p* < 0.0001) and stress‐response (*p* < 0.05) programmes. Consistent with known roles of epithelial fucosylation, lower α1,2‐fucose levels correlated with fewer SCFA‐producing bacteria (FDR<0.05), higher microbial translocation and inflammation (*p* < 0.04), and premature ageing (*p* = 0.002). In anaerobic fermentation assays, stool from PWH produced less SCFAs at baseline than controls (*p* = 0.026). Supplementation with 2′FL restored SCFA production in PWH fermentations (*p* = 0.03; Figure 2A,B), and fermentation supernatants protected intestinal organoids from stress‐mediated disruption (Figure 2C,D).

**FIGURE 1:**
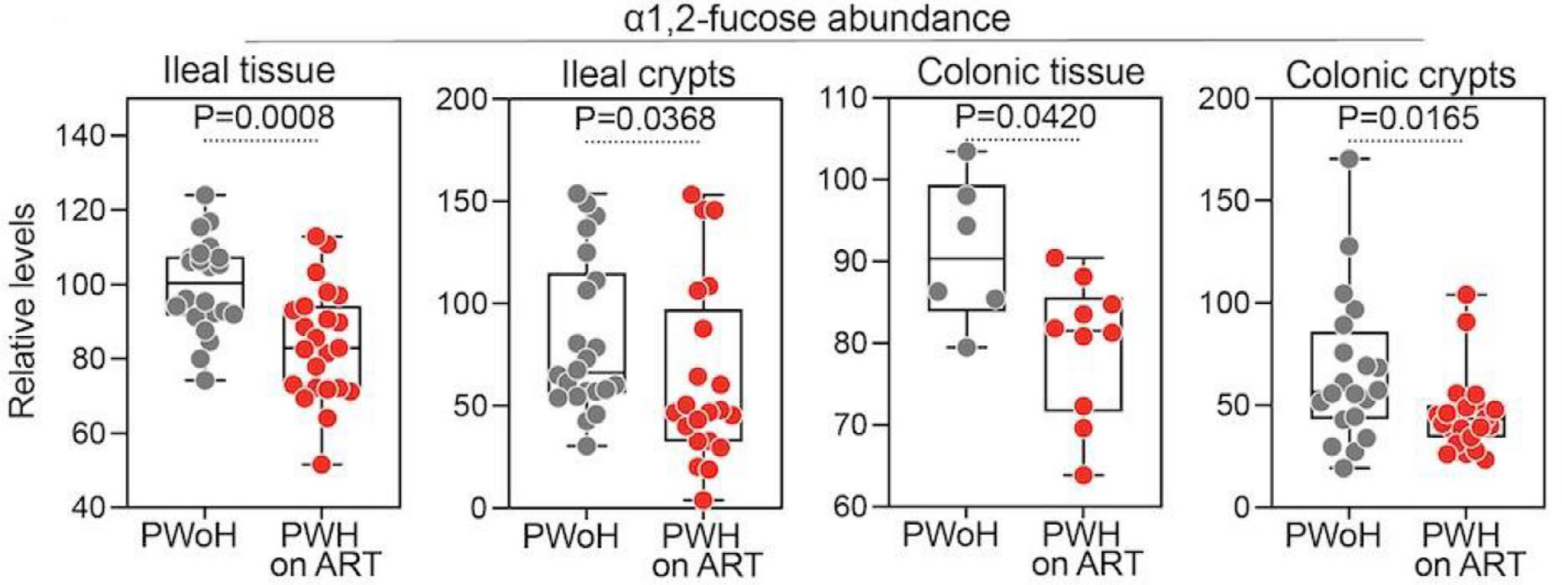
OAA1002

**FIGURE 2:**
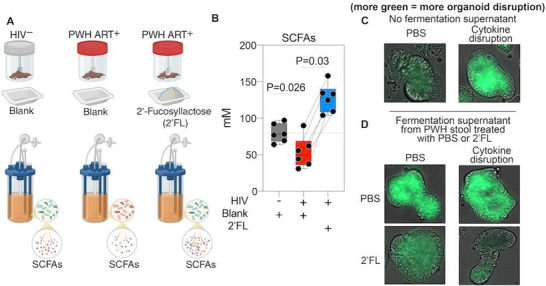
OAA1002

**Conclusions**: α1,2‐fucosylation represents a modifiable control point in the host‐microbiome axis that links reduced SCFAs, barrier fragility, inflammation and premature ageing in PWH. These data support a biomarker‐guided clinical evaluation of 2′FL as a precision prebiotic strategy to restore gut ecology in PWH.

## OAA1003 Inflammation Beyond Undetectable: A Proteomic Signature of Residual Cardiometabolic Risk in Virally Suppressed People Living With HIV in Taiwan

H. S. Toh ^1,2,3^, C.‐T. Liao^4,5^, Y.‐C. Su^4^, H.‐C. Ku^6^, B.‐Y. Lin^6^, W.‐T. Chang^5^, W.‐C. Ko^2^, Y.‐S. Tsai^2^, H.‐J. Tang^7^

^1^Chi Mei Medical Center, Department of Quality Management, Tainan City, Taiwan, Province of China; ^2^National Cheng Kung University, Institute of Clinical Medicine, Tainan City, Taiwan, Province of China; ^3^National Defense Medical University, College of Medicine, Taipei City, Taiwan, Province of China; ^4^Chi Mei Medical Center, Department of Medical Education, Tainan City, Taiwan, Province of China; ^5^Chi Mei Medical Center, Department of Cardiology, Tainan City, Taiwan, Province of China; ^6^Chi Mei Medical Center, Infection Control Center, Tainan City, Taiwan, Province of China; ^7^Chi Mei Medical Center, Department of Infectious Diseases, Tainan City, Taiwan, Province of China

**Background**: Despite effective antiretroviral therapy, people living with HIV (PLHIV) remain at elevated cardiometabolic risk even with sustained viral suppression. The biological mechanisms underlying this residual risk remain incompletely understood. We compared plasma proteomic profiles between virally suppressed PLHIV and individuals receiving HIV pre‐exposure prophylaxis (PrEP) to identify molecular signatures associated with non‐HIV cardiometabolic outcomes.

**Methods**: This study used the “Double V” prospective cohort at a tertiary medical centre in southern Taiwan. From April 2024, virally suppressed adults on ART (Exp) and HIV‐negative individuals on PrEP (Ctrl) were recruited during routine follow‐up visits; those with cardiovascular symptoms or heart failure were excluded by clinical assessment and echocardiography. Plasma was analysed using untargeted label‐free LC–MS/MS proteomics. In 52 male participants (Exp *n* = 41; Ctrl *n* = 11, mean age of 38), 409 proteins were identified and 375 passed QC for downstream analyses (identification FDR 1%). PCA and PLS‐DA assessed group separation, followed by differential protein analysis and GO/KEGG enrichment. Analyses were performed in R.

**Results**: Plasma proteomic profiling demonstrated distinct differential protein expression between virally suppressed PLHIV and PrEP users (Figure 1). Pathway enrichment analyses revealed coordinated biological changes rather than isolated protein shifts (Figure 2). Compared with PrEP controls, PLHIV showed enrichment of complement and coagulation cascades, platelet alpha and dense granule components, and cellular defence pathways, indicating persistent activation of innate immunity and haemostatic systems despite virologic control (Figure 2A,C). Concurrently, proteins involved in antioxidant activity, lipid metabolism, insulin‐like growth factor binding and extracellular matrix maintenance were relatively reduced (Figure 2B). Together, these findings indicate subclinical cardiometabolic vulnerability in PLHIV despite apparent virologic control.

**FIGURE 1:**
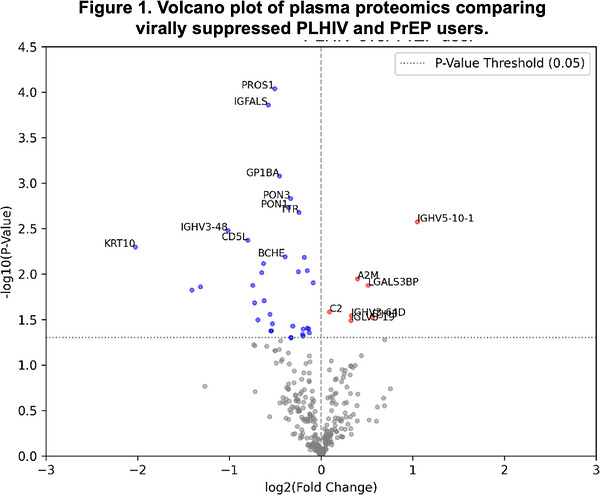
OAA1003 | Volcano plot of plasma proteomics comparing virally suppressed PLHIV and PrEP users.

**FIGURE 2:**
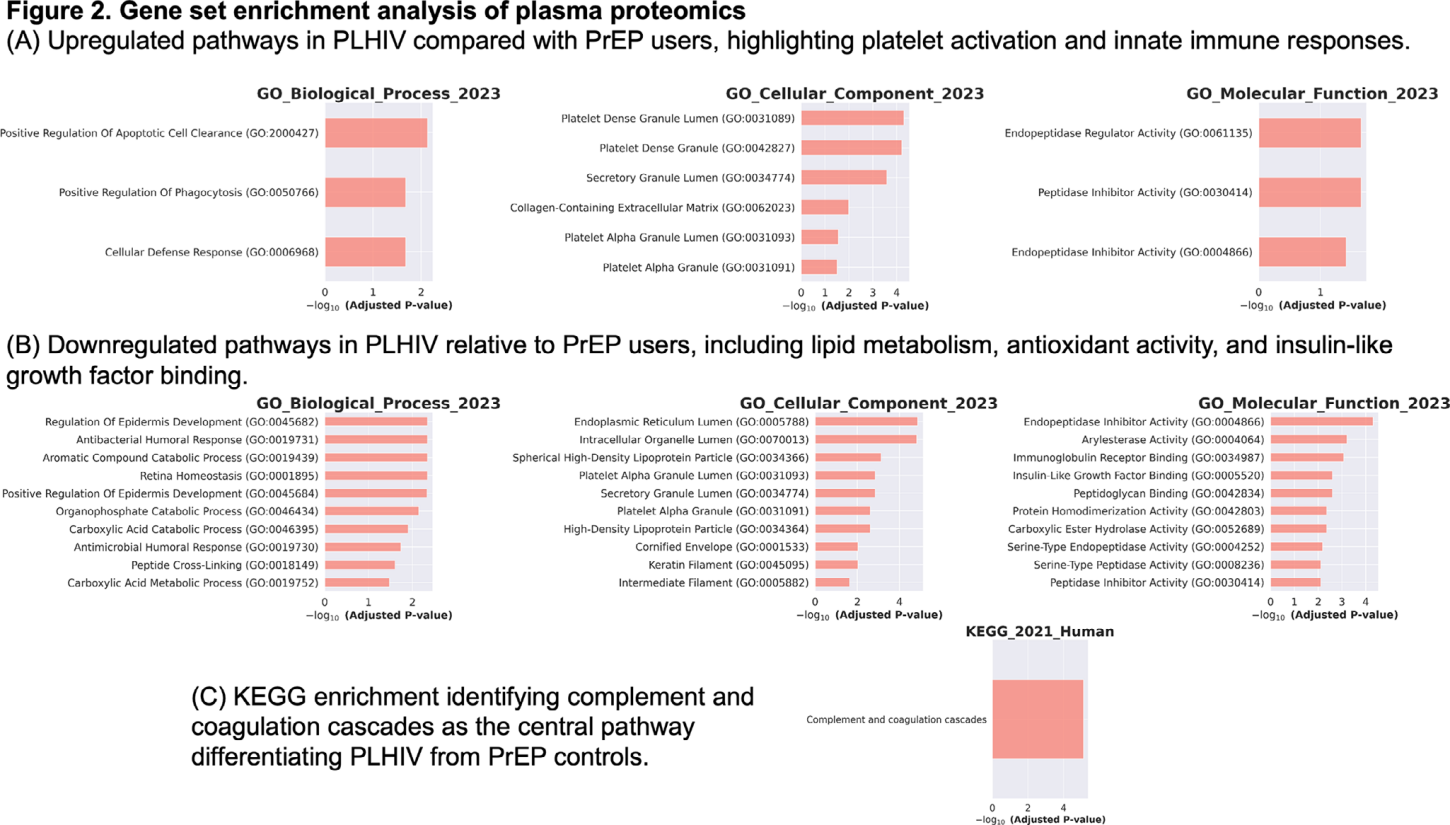
OAA1003 | Gene set enrichment analysis of plasma proteomics.

**Conclusions**: Virally suppressed PLHIV exhibited a distinct plasma proteomic profile compared with PrEP users, characterized by enrichment of complement–coagulation and platelet‐related pathways alongside relatively reduced metabolic protective processes, suggesting potential subclinical cardiometabolic vulnerability despite virologic control. As an ongoing prospective cohort, we will longitudinally track proteomic patterns together with cardiometabolic biomarkers and imaging measures to explore their stability over time and to generate hypotheses on how these molecular signatures may relate to future cardiometabolic risk and prevention targets.

## OAA1004 Overwhelming Evidence of HIV‐Associated Metabolic Dysregulation in Pregnancy That Reveals Pathway‐Level Mechanisms Underlying Adverse Pregnancy Outcomes

C. Mulenga ^1,2,3^, L. Kamulaza^1,3^, J. Sakala^1,4^, T. Pokaprakarn^5^, Y. Sebastião^5^, N. Sindano^1,5^, K. Rittenhouse^5^, M. Kasaro^1^, P. Musonda^1,3^, K. De Paris^5^, J. Stringer^5^

^1^University of North Carolina (UNC) – Global Projects, LLC Zambia, Lusaka, Zambia; ^2^Levy Mwanawasa Medical University (LMMU), Lusaka, Zambia; ^3^University of Zambia (UNZA), Lusaka, Zambia; ^4^University Teaching Hospital (UTH), Lusaka, Zambia; ^5^The University of North Carolina (UNC) at Chapel Hill, Chapel Hill, United States

**Background**: HIV affects over 1.2 million pregnancies annually and is consistently associated with increased risk of adverse pregnancy outcomes (APOs). However, the biological pathways linking HIV to spontaneous preterm birth (sPTB), small for gestation age (SGA) and stillbirth (SB) remain incompletely understood. This study used untargeted metabolomics to quantify the effect of HIV on the maternal plasma metabolome across gestation and to identify metabolic pathways associated with APOs.

**Methods**: Longitudinal maternal plasma samples with linked clinical data were collected from a combined observational cohort and contemporary randomized trial, including 2498 pregnant women in Lusaka, Zambia, with four aligned sampling time points during pregnancy (August 2015−January 2020) (Figure 1A,B). A total of 6102 samples underwent untargeted metabolomics, detecting 58,642 features and annotating 978 metabolites. Associations with APOs were evaluated using a case‐cohort design with logistic regression and false discovery rate control (FDR = 10%). Principal component analysis (PCA) and cross‐validated partial least squares discriminant analysis (PLS‐DA) were used to quantify effect of HIV on the metabolome. Disease‐based metabolite set enrichment analysis (MSEA) using the KEGG library was performed to identify enriched metabolic pathways.

**Results**: PCA demonstrated that HIV seropositivity exerted a dominant effect on the metabolome, accounting for study type independent of pregnancy outcome (Figure 2A1). PLS‐DA showed strong discrimination by HIV status, with sensitivity of 88.0% and specificity of 99.6% (Figure 2A2). A critical gestational age window from 24 to 28 weeks was identified, during which sPTB was associated with 23 metabolites (Figure 2B) and SGA with 43 metabolites (Figure 2C). SB was associated with three metabolites in both full cohort and HIV‐stratified analyses, occurring in a later window (≥32 weeks; Figure 2D). MSEA identified six enriched pathways associated with sPTB (Figure 2E) and 31 enriched pathways associated with SGA, with pregnancy‐related metabolic pathways showing the highest presentation (Figure 2F).

**FIGURE 1:**
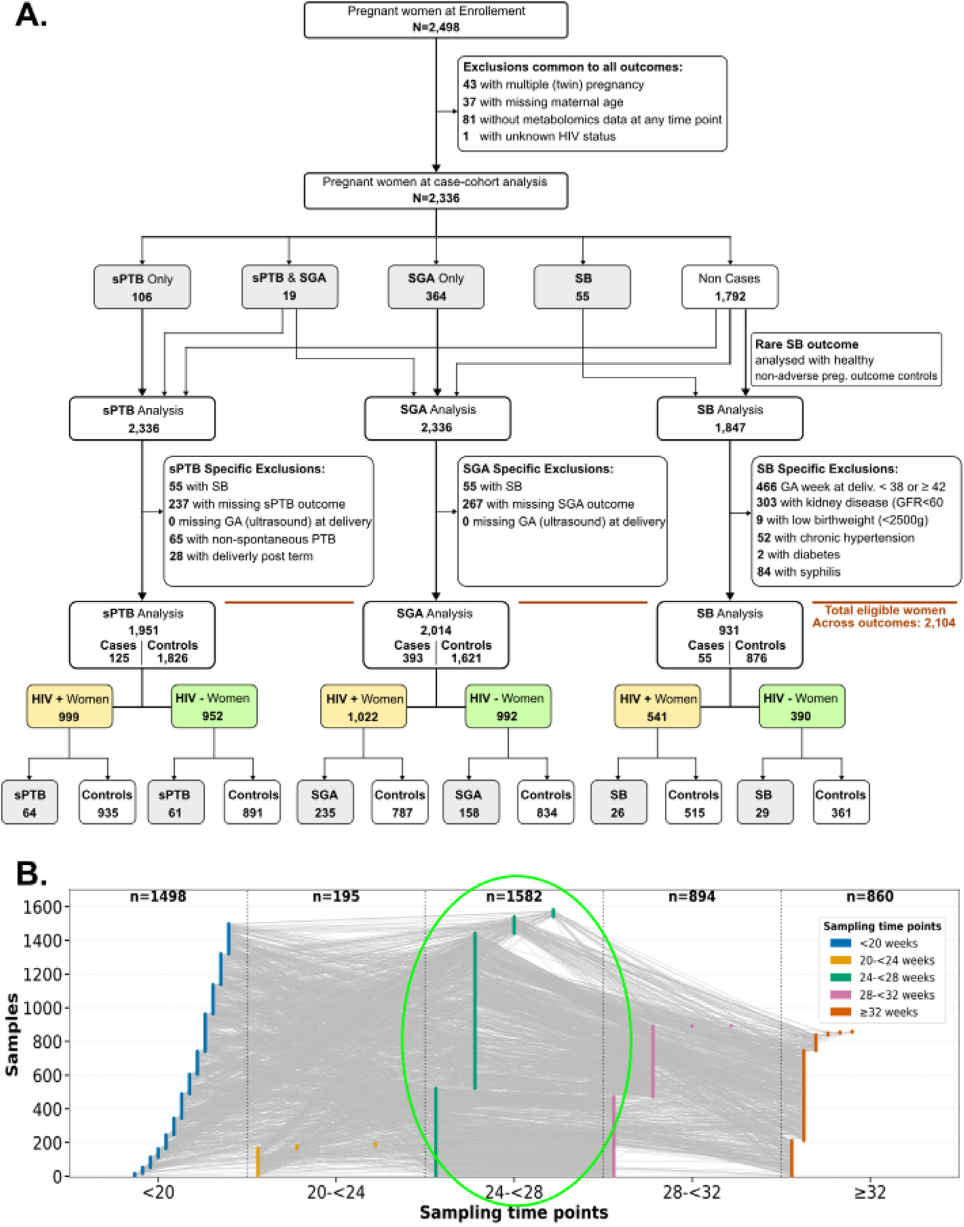
OAA1004 | Participants and samples used. (A) Participant eligibility flowchart of 2498 pregnant women from enrolment to the end of the follow‐up period and development of APOs assessed simultaneously, leading to 2104 eligible women across three outcomes (sPTB, SGA and SB). (B) Sample contributions at any sampling time point (most samples in interval: 24−28 weeks).

**FIGURE 2:**
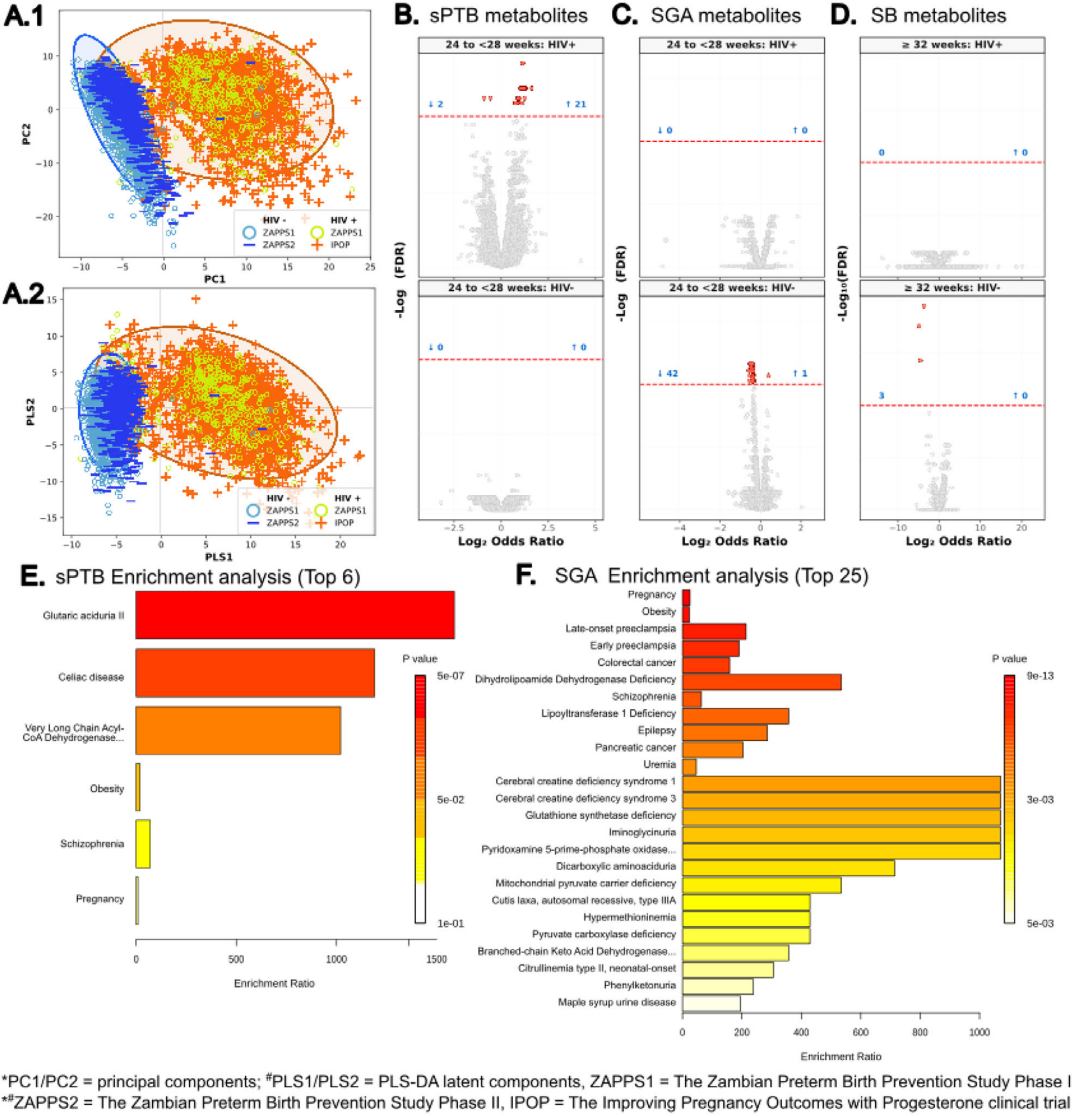
OAA1004 | Sample separation, modelling and MSEA results: (A.1) PCA*, (A.2) PLS‐DA#. HIV serostatus classification achieved sensitivity of 88.01% and specificity of 99.62%. (B) sPTB metabolites. (C) SGA metabolites. (D) SB metabolites. (E) sPTB metabolites enriched pathways. (F) SGA metabolites enriched pathways.

**Conclusions**: HIV profoundly reshapes the metabolome during pregnancy, affecting several metabolic processes associated with APOs. HIV‐associated metabolic dysregulation is liked to sPTB and appears to contribute to impaired foetal growth. These findings identify a critical mid‐gestation metabolic window (24−48 weeks) that may inform the timing of interventions to reduce HIV‐associated APO risk.

## OAA1005 Extracellular acyl‐coA‐Binding Protein Binds to Lipids, Decreasing T‐Cell Viability and Function in People With HIV

S. Isnard^1,2^, O. Aiyana^1^, N. Ghahari^3^, L. Royston^1,2,4^, T. Mabanga^1,2^, C. A. Berini^1,2^, N. F. Bernard^1^, J. Van Grevenynghe^3^, G. Kroemer^5^, J.‐P. Routy ^1,2,6^

^1^Research Institute of the McGill University Health Centre, Montreal, Canada; ^2^McGill University Health Centre, Chronic Viral Illness Service, Montreal, Canada; ^3^Centre Armand‐Frappier Santé‐Biotechnologie, Laval, Canada; ^4^Geneva University Hospitals, Geneva, Switzerland; ^5^Centre de recherche des Cordeliers, Inserm U1138, Paris, France; ^6^McGill University Health Centre, Division of Hematology, Montreal, Canada

**Background**: T‐cell function remains impaired in people with HIV (PWH), even following extended antiretroviral therapy (ART), partially due to metabolic dysfunction and inability to induce autophagy. We have found elevated levels of the autophagy inhibitor acyl coA binding protein (ACBP) in the plasma of ART‐treated PWH as compared to controls. Herein, we assessed the influence of extracellular ACBP on T‐cell function in PWH on ART.

**Methods**: Plasma ACBP were quantified by ELISA in 50 PWH on effective ART (mean duration of 14.7 years) and 30 controls with similar age. In vitro, recombinant ACBP (recACBP) was added at increasing concentrations up to 10 µg/mL on PBMCs from PWH on ART and controls. Lipid uptake and T‐cell responses (IFNg or TNFa production), CFSE dilution for proliferation assays were assessed by flow cytometry following anti‐CD3 antibodies, phorbol‐myristate acetate+ionomycin or HIV peptide stimulation. A synthetic acyl‐coA was added to cell culture at up to 10 µg/mL.

**Results**: Plasma ACBP levels were higher in PWH on ART compared to controls (median 127.5 vs. 78.1 ng/mL, *p* = 0.03), independently of age and sex.

To mimic the effect of extracellular ACBP, we added recACBP to PBMCs from ART‐treated PWH or controls before stimulating them. RecACBP prevented CD4 and CD8 T‐cell cytokine production and secretion as well as cell proliferation in response to PMA‐ionomycin, anti‐CD3 antibodies or HIV peptides.

RecACBP addition decreased expression of autophagy markers Beclin1, p62 and ULK1 in anti‐CD3 stimulated CD4 and CD8 T‐cells. Treatment with autophagy inducers spermidine or dimethyl‐oxoglutarate did not rescue T‐cell proliferation nor cytokine responses.

RecACBP treatment increased the lipid uptake as observed with BODIPY lipid probes. Addition of acyl‐CoA lipids only did not influence T‐cell responses. However, recACBP and acyl‐CoA addition totally blocked T‐cell responses and induced cell death.

**Conclusions**: Extracellular ACBP increase T‐cell lipid uptake, leading to metabolic irresponsiveness and T‐cell death, warranting the development of ACBP inhibitors to enhance anti‐HIV T‐cell responses in PWH, towards an HIV cure.

## OAA1502 IL‐15 Superagonist N‐803, With or Without bNAbs, Induces Temporary Reduction and Transcriptional Activation of the Intact HIV Reservoir

R. Scheck ^1^, I. Miller^1^, H. Mar^2^, R. Bosch^2^, A. R. Ward^1^, P. Sinha^1^, E. Naing^1^, S. Terry^1^, C. Ovies^1^, S. Shea^1^, C. S. Venuto^3^, R. DiFrancesco^4^, M. L. Freeman^5^, J. C. Cyktor^6^, J. W. Mellors^6^, G. D. Morse^7^, B. Reddy^8^, J. Berardi^1^, R. Tressler^9^, M. Caskey^10^, R. B. Jones^1^, T. Wilkin^11^

^1^Weill Cornell Medicine, Department of Medicine, New York, United States; ^2^Harvard TH Chan School of Public Health, Center for Biostatistics in AIDS Research, Boston, United States; ^3^University of Rochester, Center for Health and Technology, Rochester, United States; ^4^University at Buffalo, Translational Pharmacology Research Core Center for Integrated Global Biomedical Sciences, School of Pharmacy and Pharmaceutical Sciences, Buffalo, United States; ^5^Case Western Reserve University School of Medicine, Rustbelt Center for AIDS Research Division of Infectious Diseases and HIV Medicine Department of Medicine, Cleveland, United States; ^6^University of Pittsburgh, Division of Infectious Diseases, Pittsburgh, United States; ^7^University at Buffalo, Department of Community Health and Health Behavior, School of Public Health and Health Professions, Buffalo, United States; ^8^ImmunityBio, Inc., Culver City, United States; ^9^National Institutes of Health, Division of AIDS, National Institute of Allergy and Infectious Diseases, Bethesda, United States; ^10^The Rockefeller University, Laboratory of Molecular Immunology, New York, United States; ^11^University of California San Diego, San Diego, United States

**Background**: The HIV reservoir remains the principal barrier to cure. Immunomodulatory strategies that reverse latency and enhance immune‐mediated clearance, such as the IL‐15 superagonist N‐803, are, therefore, of high interest.

A5386 is an open‐label, phase 1 trial evaluating safety and tolerability of N‐803 administered ± a single infusion of broadly neutralizing antibodies (bNAbs, VRC07‐523LS, 10‐1074LS). Following intervention, participants underwent an analytical treatment interruption (ATI). Here, we characterize HIV reservoir dynamics prior to ATI using Q4ddPCR, a highly sensitive four‐target assay that precisely quantifies intact proviruses and closely correlates with viral outgrowth.

**Methods**: Intact reservoir size was measured by Q4ddPCR in 42 participants at study entry and 10 weeks after completion of N‐803 dosing. Additional samples after five of eight planned N‐803 doses were available for 39 participants. Transcriptionally active reservoir size was assessed in 30 participants by quantifying unspliced and total polyadenylated cell‐associated HIV RNA (caRNA). Reservoir analyses were pooled across both intervention arms.

**Results**: 48 participants enrolled (23 N‐803+bNAbs; 25 N‐803 alone): 90% male, with 19% Hispanic, 40% non‐white representation; median age 50 years. All participants completed bNAb infusion; 33 completed all doses of N‐803, 10 completed 5−7 doses. 19 N‐803+bNAbs participants entered the ATI: one (5%) maintained HIV RNA <200 c/mL at ATI week 8 (95% CI lower limit: 0.3%), three (16%) had HIV RNA <1000 c/mL at ATI week 8 and two remained off ART for 24 weeks.

After five N‐803 doses, intact HIV DNA declined transiently by a median 0.21 log_10_ (IQR 0−0.38; *p* = 0.003, Figure 1), corresponding to an estimated reservoir half‐life of ∼16 weeks. This coincided with a median 0.36 log_10_ reduction in total polyadenylated HIV RNA (IQR 0.04−0.83; *p* = 0.04, Figure 2). Intact HIV DNA rebounded to baseline levels 10 weeks post‐intervention. Defective proviruses remained unchanged. Unspliced caRNA increased median 0.3 log_10_ (IQR 0.07−0.8, *p* = 0.02) at 10 weeks post‐intervention.

**Conclusions**: N‐803 ± bNAbs induced a transient reduction of the intact reservoir alongside increased transcriptional activity, consistent with latency perturbation and enhanced proviral visibility. While this did not translate into sufficient ART‐free viral control to meet the primary endpoint, these data provide evidence that IL‐15‐based strategies can meaningfully modulate the intact reservoir.

**FIGURE 1:**
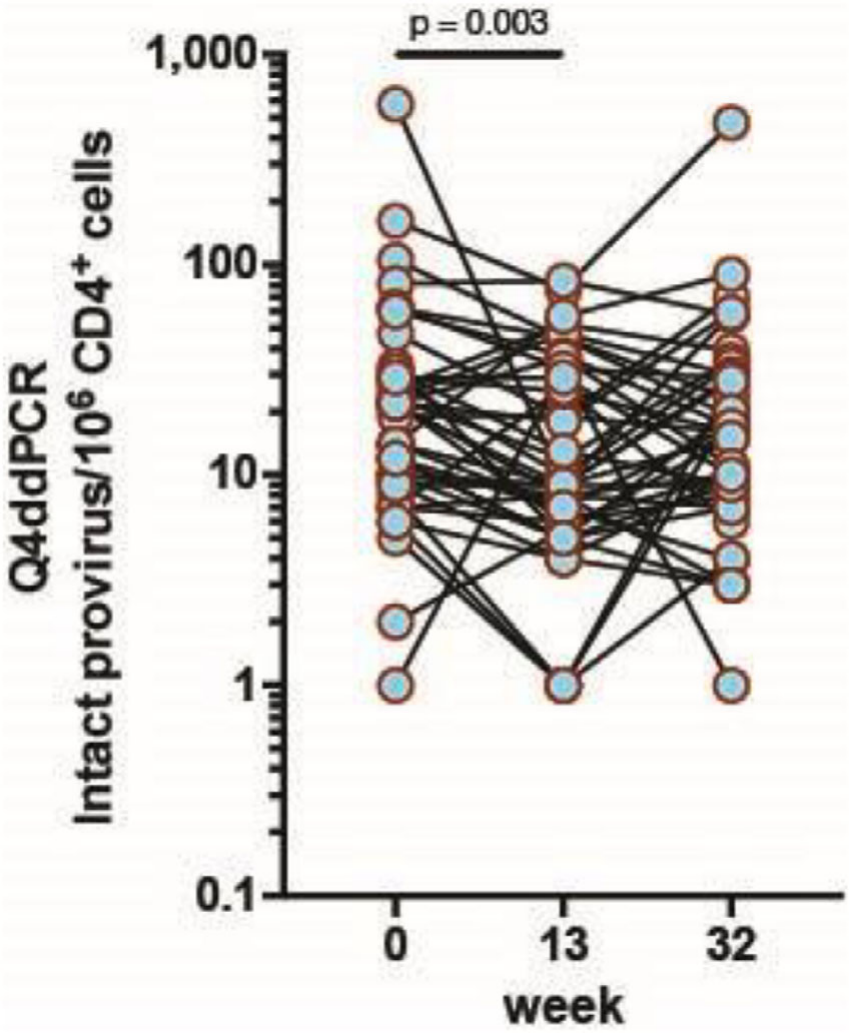
OAA1502

**FIGURE 2:**
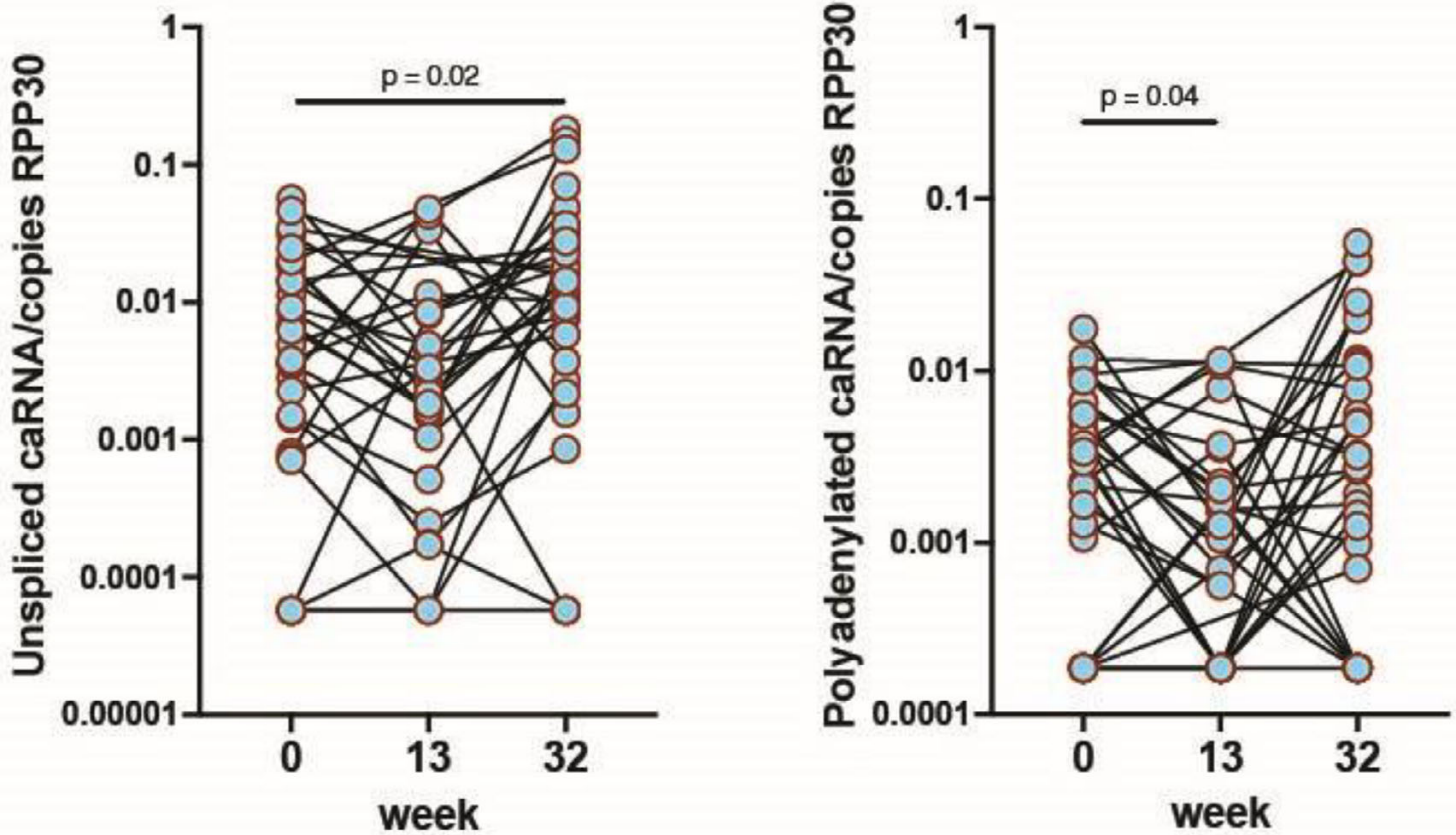
OAA1502

## OAA1503 RHIVIERA‐01: Analytical Treatment Interruption in Early Treated People With B35(53)‐Bw4TTC2 Genotype

C. Goujard^1^, L. Romero‐Martin^2^, G. Mchantaf^3,4,5^, R. Foare^6^, V. Meiffredy^6^, V. Monceaux^2^, M. Allombert^2^, M. Lechartier^2^, P. Delobel^7^, C. Allavena^8^, H. Mouquet^2^, C. Protière^9^, C. Durier^6^, V. Avettand‐Fenoel^3,4,5^, L. Meyer^6^, A. Saez‐Cirion ^2^, ANRS 175 Rhiviera‐01 Study Group

^1^Université Paris‐Saclay, AP‐HP, Hôpital Bicêtre, Le Kremlin Bicêtre, France; ^2^Institut Pasteur, Paris, France; ^3^CHU d'Orléans, Orléans, France; ^4^Université d'Orléans, Orléans, France; ^5^Université Paris Cité, INSERM, U1016; CNRS, UMR8104, Paris, France; ^6^Université Paris‐Saclay, Inserm CESP U1018, Paris‐Saclay, France; ^7^CHU de Toulouse, Toulouse, France; ^8^CHU de Nantes, Nantes, France; ^9^Reshape U1290, Lyon, France

**Background**: The combination of HLA‐B*35(53) alleles with the MHC‐related Bw4TTC2 genotype, associated with NK cell‐education, was identified in post‐treatment controllers from VISCONTI, supporting a role of NK cells in post‐treatment HIV‐1 control. The ANRS‐RHIVIERA‐01 trial evaluated the outcome of analytical ART interruption (ATI) in early treated people with this genotype.

**Methods**: The trial was prospective, multicentre, proof‐of‐concept assessing HIV control after ATI in participants from the ANRS PRIMO cohort, with the B35(53)‐Bw4TTC2 genotype and treated since acute infection (AHI). The primary endpoint was the proportion of participants maintaining viral load (VL)<400 cp/mL at 24 weeks post‐ATI. VL was monitored weekly for 8 weeks, then biweekly. Criteria for restarting ART included two consecutive VL>10,000 cp/mL or CD4<350/mm^3^.

**Results**: Sixteen participants (three women) were included; 14 restarted ART before W24 (seven without meeting immunovirological restart criteria). Median time to VL>50 cp/mL was 23 days [IQR: 17−43] and to VL>400 cp/mL was 30 days [22−47]. Eight participants experienced rapid viral rebound with VL (5.2 [4.8−5.9] log cp/mL) similar to AHI (5.9 [5.2−6.8], *p* = 0.12) and restarted ART within 10 weeks post‐ATI. The other eight remained off‐ART for median 15 weeks [12−22], showed lower rebound slopes than those with early ART resumption (*p* = 0.0019) and reached 2‐Log [1.1−2.7] lower VL during ATI than in AHI (*p* = 0.002). Groups with early or delayed ART re‐initiation were similar regarding VL during AHI (*p* = 0.7) or total‐HIV DNA pre‐ATI (*p* = 0.8), but those in the early ART group had higher integrated‐HIV DNA (*p* = 0.047). Six participants in the delayed‐ART group (including the three women) never reached VL > 10,000 cp/mL. Overall, four participants had VL < 1000 cp/mL at the end of ATI. One participant (6.25%) maintained VL<50 cp/mL at W24 post‐ATI (>30 months so far). Higher frequencies of NKG2A+CD57‐NK cells, stronger NK‐mediated HIV‐1 inhibition and HIV‐specific CD8+ T cells with combined expansion and effector capacities were positively correlated both at baseline and W4 post‐ATI with delayed and reduced rebound.

**Conclusions**: Constitutive characteristics of participants can, without immune intervention, strongly influence viral rebound in ATI‐studies. Improved viral control during ATI associated with enhanced NK cell antiviral activity and higher frequencies of immature NKG2A+NK cells is consistent with a favourable effect of the B35(53)‐Bw4TTC2 genotype.

## OAA1504 Long‐Term HIV Reservoir Clonal Dynamics Are Maintained by Antigen‐Driven CD4+ T‐Cell Proliferation During Viral Suppression in Cisgender Males With HIV

J. Stern ^1^, T. Traudt^1^, B. Haddock^1^, A. Person^1^, R. Hoh^2^, R. Fromentin^3,4^, A. Espinosa Ortiz^3,4^, S. G. Deeks^2,5^, N. Chomont^3,4^, D. B. Reeves^1^, L. B. Cohn^1^

^1^Fred Hutch Cancer Center, Vaccine and Infectious Disease Division, Seattle, United States; ^2^University of California San Francisco, Division of HIV, Infectious Diseases, and Global Medicine, San Francisco, United States; ^3^CHUM Research Centre University de Montréal, Montreal, Canada; ^4^Université de Montréal, Microbiology, Infectious Diseases and Immunology, Montreal, Canada; ^5^Zuckerberg San Francisco General Hospital, HIV, Infectious Diseases and Global Medicine, San Francisco, United States

**Background**: HIV‐1 persists due to a long‐lived reservoir of latently infected CD4+ T‐cells containing replication‐competent proviruses which is partly maintained through antigen‐driven proliferation. Progressive immune exhaustion may impair CD4+ T‐cell responses over time. We evaluated how antigen‐driven CD4+ T‐cell activation and proliferation shape the defective and intact HIV reservoirs longitudinally.

**Methods**: Peripheral blood mononuclear cells from longitudinal leukaphereses (median 5.1 years [range 3.6–9.8]) of five virally suppressed cisgender males with HIV were CD8‐depleted and stimulated ex vivo with overlapping antigen peptide pools. CMV pp65, HIV Gag and EBV EBNA1/BZLF1 epitopes were tested. Antigen‐responsive CD4+ T‐cells (ARCTs) were flow‐sorted by activation‐induced marker expression. Proliferation was assessed using a cell‐tracking dye. HIV DNA quantification, T‐cell receptor (TCR) sequencing and single‐genome near‐full length (NFL) proviral sequencing were performed. Negative‐binomial and power‐law modelling was used to estimate HIV DNA, and TCR and NFL clonality, respectively.

**Results**: CD4+ T‐cell responses to HIV (*R* = –0.85, *p* = 0.0001) and EBV antigens (EBNA1 *R* = –0.67, *p* = 0.035; BZLF1 *R* = –0.72, *p* = 0.029) declined over time on ART, while CMV responses remained stable (Figure 1A). ARCTs maintained strong proliferative capacity upon all antigen stimuli over 13−35 years of ART. HIV infection frequency remained stable over time within total CD4+ and ARCTs, with HIV DNA enriched in CMV‐responsive cells (2.25‐fold cf. Total CD4+, *p* = 0.025, Figure 1B,D). HIV DNA in HIV‐responsive cells increased after antigen‐driven proliferation in vitro (3.76‐fold, *p* = 0.0074, Figure 1C). NFL sequencing revealed substantial proviral clonality in total CD4+ and ARCTs, maintained over time (Figure 2C,D), with clonal expansion events of intact proviruses. TCR clonality of ARCTs was greater than total CD4+, and TCR/NFL sequence clonality increased after antigen‐driven proliferation (Figure 2B,C). Proviral clonality of total CD4+ and ARCTs mirrored antigen‐responsive TCR clonalities and were more clonal than total CD4 T‐cell TCRs.

**Conclusions**: Despite declining frequencies of antigen‐responsiveness over time on ART, ARCTs maintain high proliferative capacity upon antigen exposure. HIV infection remains stably clonal and is enriched in CMV‐responsive ARCTs which explains why during ART, the HIV reservoir is maintained by infected CD4+ T‐cells responding to antigen. In HIV‐responsive cells, HIV‐infected cells may outgrow uninfected cells.

**FIGURE 1:**
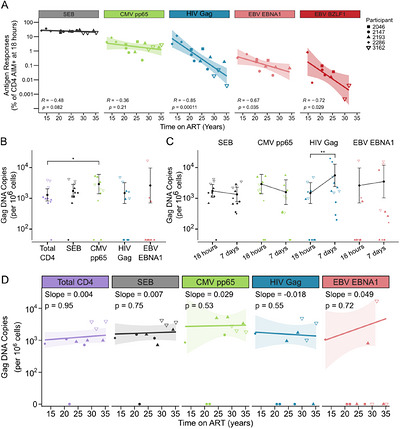
OAA1504

**FIGURE 2:**
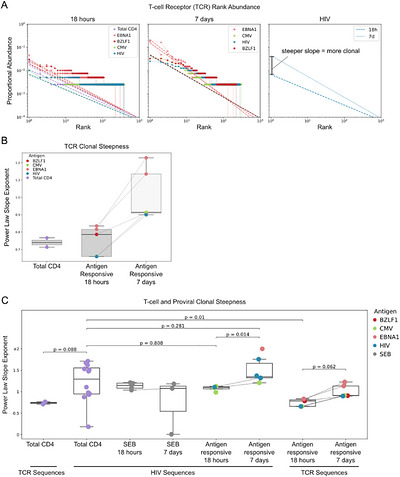
OAA1504

## OAA1505 Evidence of HIV‐1 Cure Following CCR5Δ32/Δ32 Allogeneic HSCT Under Complex Virological Conditions

C. Elsner ^1^, B.‐E. O. Jensen^2^, A. Wensing^3,4^, M. Nijhuis^3^, M. Salgado^5,6,7^, A. Walker^8^, E. Heger^9^, E. Knops^9^, L. Schöler^1^, M. Fiedler^1^, K. Sutter^1,10^, E. Littwitz‐Salomon^1,10^, H. S. Schwarzer‐Sperber^10^, R. Schwarzer^10^, N. Lübke^8^, J. Martinez‐Picado^5,6,7^, T. Harrer^11,12^, M. Däumer^13^, A. Thielen^13^, F. Maischack^14^, L. Cords^15^, J. Schulze zur Wiesch^15^, A. Mohring^16^, C. Rautenberg^16^, T. Schroeder^16^, M. Lindemann^17^, J. Timm^8^, R. Kaiser^18,19^, M. Trilling^1,10^, U. Dittmer^1^, S. Esser^10,14^

^1^Institute for Virology, University Hospital Essen, University Duisburg‐Essen, Essen, Germany; ^2^Department of Gastroenterology, Hepatology, and Infectious Diseases, Medical Faculty and University Hospital Düsseldorf, Heinrich Heine University Düsseldorf, Düsseldorf, Germany; ^3^Translational Virology, Department of Medical Microbiology, University Medical Center Utrecht, Utrecht, the Netherlands; ^4^Ezintsha, University of the Witwatersrand, Johannesburg, South Africa; ^5^IrsiCaixa, ICREA, UVic‐UCC, Catalonia, Spain; ^6^CIBERINFEC, Madrid, Spain; ^7^Germans Trias i Pujol Research Institute, Badalona, Spain; ^8^Institute of Virology, University Hospital Düsseldorf, Heinrich Heine University Düsseldorf, Düsseldorf, Germany; ^9^Institute of Virology, Faculty of Medicine and University Hospital Cologne, University of Cologne, Cologne, Germany; ^10^Institute for the Research on HIV and AIDS‐associated Diseases, University Hospital Essen, University Duisburg‐Essen, Essen, Germany; ^11^Department of Internal Medicine 3, Infectious Diseases and Immunodeficiency Section, Universitätsklinikum Erlangen, Friedrich‐Alexander‐Universität Erlangen‐Nürnberg, Erlangen, Germany; ^12^Deutsches Zentrum für Immuntherapie (DZI), Friedrich‐Alexander‐Universität Erlangen‐Nürnberg (FAU) and Universitätsklinikum Erlangen, Erlangen, Germany; ^13^Seq‐IT GmbH & Co KG, Kaiserslautern, Germany; ^14^Department of Dermatology, HPSTD Outpatient Clinic, University Hospital Essen, University Duisburg‐Essen, Essen, Germany; ^15^Department of Medicine, Infectious Diseases Unit, University Medical Center Hamburg Eppendorf, Hamburg, Germany; ^16^Department of Hematology and Stem Cell Transplantation, West German Cancer Center, University Hospital Essen, Essen, Germany; ^17^Institute for Transfusion Medicine, University Hospital Essen, University Duisburg‐Essen, Essen, Germany; ^18^Institute of Virology, University Hospital Duesseldorf, Heinrich Heine University Duesseldorf, Duesseldorf, Germany; ^19^German Center for Infection Research (DZIF), Partner Site Bonn‐Cologne, Cologne, Germany

**Background**: Rapid viral rebound after interruption of antiretroviral therapy (ART) proofs the persistence of replication‐competent HIV‐1 in long‐lived CD4^+^ T cell reservoirs as the main barrier to cure. Sustained HIV‐1 remission has been reported in rare cases following allogeneic hematopoietic stem cell transplantation (allo‐HSCT), most commonly using CCR5Δ32/Δ32 donor cells, but also in isolated cases of CCR5Δ32 heterozygosity or even two CCR5 wild‐type donor alleles, predominantly in the context of CCR5‐tropic virus. Evidence for a successful cure under complex virological conditions, including mixed HIV‐1 tropism and hepatitis B virus (HBV) coinfection, remains limited.

**Methods**: We conducted a longitudinally follow‐up of an HIV‐1 RNA–suppressed individual with resolved HBV coinfection who underwent allo‐HSCT from a 10/10 HLA‐matched CCR5Δ32/Δ32 donor. Assessments included HIV‐1 tropism analysis by proviral sequencing, quantification of HIV‐1 DNA by droplet digital PCR, quantitative viral outgrowth assays, tissue‐based intact proviral DNA analysis and immunological profiling. Following predefined remission criteria, analytical treatment interruption (ATI) was initiated following suppressive ART (TAF/FTC/BIC), with close monitoring of plasma HIV‐1 RNA, HIV‐1 persistence markers, HBV DNA and liver function tests.

**Results**: Proviral V3 sequencing revealed a mixed‐tropic HIV‐1 population (geno2pheno; FPR 9.6%). After allo‐HSCT and immune reconstitution, HIV‐1–specific antibody responses declined and HIV‐1–specific T cell responses were not detectable. HIV‐1 DNA were quantified at extremely low levels (7.4 copies per million CD4^+^ T cells), and no replication‐competent virus was detectable in viral outgrowth assays. In addition, intact proviral HIV‐1 DNA was absent in ileal or rectal biopsy specimens 42 months post‐transplantation. Accordingly, ATI was initiated 51 months after allo‐HSCT. While HIV‐1 RNA remained undetectable 12 months after ATI, HBV reactivation was observed 25 days post‐ATI. Despite slow progression, antiviral therapy with entecavir was initiated at 4.8 months post‐ATI when HBV DNA reached 14.79 × 10^6^ IU/mL. Longitudinal follow‐up and additional analyses will further characterize these findings.

**Conclusions**: This case highlights the feasibility of sustained HIV‐1 remission following CCR5Δ32/Δ32 allo‐HSCT even under complex virological conditions, including mixed viral tropism and HBV coinfection. However, analytical treatment interruption necessitates stringent safety monitoring, with HBV reactivation representing a key clinical risk independent of HIV‐1 rebound.

## OAA2702 Early Functional Adaptive NK Cell Responses in Acute HIV Acquisition Are Associated With Sustained HIV‐1 Viral Control

A. Alrubayyi ^1^, A. S. Hassan^2^, A. Hsieh^1^, J. Hare^3^, J. Gilmour^3^, J. Kokiçi^4^, M. A Price^5^, W. Kilembe^6^, E. Karita^7^, E. Hunter^8^, E. Ruzagira^9^, J. Esbjörnsson^10^, E. J. Sanders^11^, D. Peppa^4^, S. L. Rowland‐Jones^1^

^1^University of Oxford, Nuffield Department of Clinical Medicine, Oxford, United Kingdom; ^2^KEMRI/Wellcome Trust Research Programme, Kilifi, Kenya; ^3^IAVI, Human Immunology Laboratory, Imperial College, London, United Kingdom; ^4^University College London, Division of Infection and Immunity, London, United Kingdom; ^5^University of California at San Francisco, Department of Epidemiology and Biostatistics, San Francisco, United States; ^6^Center for Family Health Research in Zambia, Lusaka, Zambia; ^7^Center for Family Health Research, Kigali, Rwanda; ^8^Emory University, Atlanta, United States; ^9^Medical Research Council (MRC) Uganda Virus Research Institute (UVRI) and London School of Hygiene and Tropical Medicine (LSHTM) Uganda Research Unit; Entebbe & Masaka, Entebbe & Masaka, Uganda; ^10^Lund University, Department of Translational Medicine, Lund, Sweden; ^11^The Aurum Institute, Johannesburg, South Africa

**Background**: Early immune events during acute HIV acquisition (AHA) can set the trajectory for long‐term viral control, yet human data on early responses during this period remain poorly characterized, largely because samples from people with AHA are rare. Natural killer (NK) cells are important effector cells that can shape adaptive immune responses and contribute to HIV‐1 control. Using unique historical samples from a cohort recruited during and shortly after peak HIV‐1 viraemia and followed longitudinally without antiretroviral therapy (ART) for up to 6 years post estimated date of acquisition (EDA), we evaluated whether early NK cell responses during AHA influence subsequent disease progression.

**Methods**: We included a total of 52 participants with HIV‐1 subtypes A (*n* = 28), C (*n* = 17) and D (*n* = 7) recruited from Kenya, Rwanda, Zambia and Uganda between 2006 and 2011 under IAVI Protocol C. Samples were collected at approximately 2 weeks (median = 18 days), 1 month (median = 32 days) and 3 months (median = 91 days) post‐HIV‐1 acquisition. Multiparameter flow cytometry was used for phenotypic characterization. Elimination of HIV‐infected CD4+ T‐cells was assessed by p24‐flow‐based assay. Soluble markers were evaluated using multiplexed assays.

**Results**: AHA induced expansion of an NK cell subset with adaptive/memory features (FcεRIγ‐NKG2C+CD57+) NK as early as 2 weeks post‐acquisition, increasing in magnitude after resolution of peak viral load. The frequency of adaptive NK cells during the first month post‐HIV acquisition correlated negatively with setpoint viral load (*r* = –0.353, *p* = 0.012). Mechanistic studies demonstrated that these populations could expand through antibody‐dependent mechanisms in combination with IL‐12/IL‐15. NK cells mediated the clearance of HIV‐infected CD4+ T‐cells, with significantly higher degranulation mediated by adaptive versus canonical NK cells. The accumulation of adaptive NK cells during the first month of acquisition correlated with optimal CD8+ T‐cell activation and enhanced virus‐specific CD8+ T‐cell responses (*r* = 0.448, *p* = 0.006). Notably, higher levels of adaptive NK cells within the first month of HIV acquisition were associated with long‐term viral control (<10,000 copies/mL) for up to 6 years in seven participants not on ART (*p* = 0.0046).

**Conclusions**: These findings provide novel insights into the correlates of protective immunity, with implications for preventative or therapeutic vaccine strategies aimed at promoting adaptive NK cell responses.

## OAA2703 CD4 Downregulation Protects HIV‐1‐Infected Cells From Antibody‐Dependent Cellular Phagocytosis Mediated by Plasma From People With HIV‐1

É. Bélanger ^1,2^, A. Tauzin^1,2^, F. Tajebe^3^, M. Chandravanshi^4^, D. Yang^5^, H.‐C. Chen^5^, T.‐J. Chiu^5^, H. Medjahed^1^, C. Bourassa^1^, J. Richard^1,2^, W. D. Tolbert^4^, D. Huryn^5^, M. Durand^1,2,6^, S. Stäger^3^, M. Pazgier^4^, A. Finzi^1,2^

^1^CHUM Research Centre, Montréal, Canada; ^2^University of Montréal, Montréal, Canada; ^3^Armand‐Frappier Centre, Laval, Canada; ^4^Uniformed Services University of the Health Sciences, Bethesda, United States; ^5^University of Pennsylvania, Philadelphia, United States; ^6^CHUM, Montréal, Canada

**Background**: The HIV‐1 envelope glycoprotein (Env) represents the only viral antigen at the surface of infected cells, making it an ideal target for antibody‐based therapies. However, most antibodies elicited in people with HIV‐1 (PWH) do not recognize Env in its native “closed” conformation. This is mainly due to the action of the viral accessory proteins Nef and Vpu, which downregulate CD4 from the surface of infected cells, thereby preventing premature “opening” of Env that would otherwise expose vulnerable epitopes recognized by PWH plasma. We previously reported that Nef and Vpu prevent Env recognition and elimination of HIV‐1‐infected cells by antibody‐dependent cellular cytotoxicity (ADCC) mediated by PWH plasma. However, whether they protect from other Fc‐effector functions like antibody‐dependent cellular phagocytosis (ADCP) remains to be determined.

**Methods**: We developed a flow cytometry‐based assay to measure the susceptibility of HIV‐1‐infected cells to elimination by ADCP mediated by PWH plasma. Our assay measures primary monocyte‐mediated ADCP against autologous primary CD4 T cells infected with primary HIV‐1 isolates.

**Results**: Nef and Vpu protected HIV‐1‐infected primary CD4 T cells from ADCP mediated by PWH plasma. Indeed, their deletion from primary infectious molecular clones rendered infected cells vulnerable to ADCP mediated by PWH plasma. This was linked to the premature engagement of Env with CD4, since introduction of the Env D368R mutation, which abrogates its interaction with CD4, protected cells infected with Nef‐ and Vpu‐deleted viruses from ADCP. Similar to our approach sensitizing HIV‐1‐infected cells to ADCC by “opening” Env using small molecule CD4 mimetics (CD4mc), we found that CD4mc also sensitized both in vitro infected cells as well as ex vivo expanded CD4 T cells from ART‐treated PWH to ADCP mediated by autologous monocytes and PWH plasma.

**Conclusions**: CD4 downregulation protects HIV‐1‐infected cells from PWH plasma‐mediated ADCP by limiting Env−CD4 interactions. By “opening” Env and exposing vulnerable epitopes, CD4mc sensitized HIV‐1‐infected cells to ADCP. A better understanding of the natural resistance of HIV‐1‐infected cells to ADCP could guide the development of novel therapeutic approaches to eliminate the viral reservoir from PWH.

## OAA2704 Different Distributions of Mucosal Antigen Specific B Cells in the Gastrointestinal Tract During SHIV Infection

S. Vimonpatranon ^1^, Y. Phuang‐Ngern^1^, K. Chumpolkulwong^2^, C. Sajjaweerawan^1^, D. Silsorn^1^, M. Creegan^3,4^, P. Saetun^1^, S. Jongrakthaitae^1^, B. Keawboon^1^, N. Tragonlugsana^1^, L. Smith^3,4^, E. Garges^1^, R. Kelly III^2^, M. S. Parsons^1,3,4^, R. Imerbsin^2^, S. J. Krebs^3^, D. J. Leggat^3^, S. M. Townsley^3,4^, A. Schuetz^1,3,4^

^1^Walter Reed Army Institute of Research‐Armed Forces Research Institute of Medical Sciences, Retrovirology, Bangkok, Thailand; ^2^Walter Reed Army Institute of Research‐Armed Forces Research Institute of Medical Sciences, Veterinary Medicine, Bangkok, Thailand; ^3^U.S. Military HIV Research Program, CIDR, Walter Reed Army Institute of Research, Silver Spring, United States; ^4^Henry M. Jackson Foundation for the Advancement of Military Medicine, Bethesda, United States

**Background**: Mucosal B cells play a critical role in antibody‐mediated immunity at sites of HIV‐1 exposure, yet their tissue‐specific distribution and antigen specificity remain poorly understood. Here, we characterize HIV Env‐specific B cells in SHIV‐infected rhesus macaques across multiple tissue compartments to inform vaccine and remission strategies.

**Methods**: Peripheral blood (PB), lymph nodes (LN) and gut tissues from ileum, sigmoid colon and rectum were collected at necropsy from 12 intravenously SHIV_BG505_‐infected macaques with five uninfected macaques serving as controls. Using multiparameter flow‐cytometry, freshly isolated mononuclear cells were phenotypically characterized and HIV Env‐specific memory B cells were detected using biotinylated gp140‐specific probes with dual streptavidin‐labelled fluorochromes.

**Results**: In SHIV‐uninfected macaques, the highest frequency of mature B cells (CD20^+^/CD10^–^) was detected in LN (25.0%) and ileum (26.2%). Among gut compartments, the absolute number of mature B cells per gram of tissue was highest in ileum (1.6x10^6^), compared to colon (0.5x10^6^, *p* = 0.04) and rectum (0.9x10^6^, *p* = NS). HIV Env‐specific memory B cells were detectable across PB (0.43%) and LN (0.48%), and at lower frequencies, in the ileum (0.016%), colon (0.010%) and rectum (0.013%). Interestingly, within the gut compartments, the number of animals with detectable HIV Env‐specific B cells responses differed with 10/12 (83%) having detectable response in the ileum, 8/12 (67%) in rectum and 6/12 (50%) in colon. HIV Env‐specific B cells in PB and LN were mostly IgG^+^ (79.8% vs. 56.4%; *p* = NS), whereas HIV Env‐specific B cells in the gut were enriched for IgA^+^ (ileum: 26.2%, colon: 32.1%, rectum: 43.8%, *p* = NS, respectively). Notably, HIV Env‐specific B cells in the ileum predominantly exhibited an early memory phenotype (IgM^+^/IgD^–^) accounting for 50.6%, compared to 14.0% in colon (*p* = 0.03), and 26.2% in rectum (*p* = NS).

**Conclusions**: These data suggest compartment‐specific B cell responses during SHIV infection, consistent with the ileum functioning as a primary inductive site. Accordingly, a higher proportion of animals exhibited detectable HIV Env‐specific B cells in the ileum, which were enriched for an early memory phenotype, whereas the lower frequencies observed in the colon are consistent with its predominant role as an effector site. This spatial distribution provides insight into mucosal B cell dynamics relevant to HIV‐1 vaccine and remission studies.

## OAA2705 Maternal Human Milk Immune Profiles Associated With Postnatal HIV Transmission to Infants During Breastfeeding in the PROMISE Study

V. Korutaro ^1^, K. Baltrusaitis^2^, P. DeMarrais^2^, A. Rukyalekere Kekitiinwa^1^, L. Chinula^3^, S. Dadabhai^4^, M. Owor^5^, M. Glenn Fowler^6^, P. Flynn^7^, L. Kuhn^8^, K. Spann^9^, L. Bode^9^, R. Brody^10^, K. Luzuriaga^10^, S. Lockman^11,12,13^, J. Jao^14^, For the PROMISE Study

^1^Baylor College of Medicine Children's Foundation Uganda, Kampala, Uganda; ^2^Harvard TH Chan School of Public Health, Center for Biostatistics in AIDS Research, Boston, United States; ^3^University of North Carolina Project‐Malawi, Lilongwe, Malawi; ^4^Johns Hopkins Research Project‐Kamuzu University of Health Sciences, Blantyre, Malawi; ^5^Makerere University Johns Hopkins University Research Collaboration, Kampala, Uganda; ^6^Johns Hopkins University School of Medicine, Baltimore, United States; ^7^St. Jude Children's Research Hospital, Memphis, United States; ^8^Columbia University, Gertrude H. Sergievsky Center, New York, United States; ^9^University of California San Diego, Department of Pediatrics, Larsson‐Rosenquist Foundation Mother‐Milk‐Infant Center for Research Excellence (MOMI CORE), and the Human Milk Institute (HMI), La Jolla, United States; ^10^University of Massachusetts Chan Medical School, Worcester, United States; ^11^Harvard T.H. Chan School of Public Health, Department of Immunology and Infectious Diseases, Boston, United States; ^12^Brigham and Women's Hospital, Division of Infectious Disease, Boston, United States; ^13^Botswana Harvard Health Partnership, Gaborone, Botswana; ^14^Northwestern University, Chicago, United States

**Background**: Maternal antiretroviral therapy (ART) has markedly reduced postnatal HIV transmission during breastfeeding; however, residual transmission persists. Data on the role of specific human milk oligosaccharides (HMOs) and immunoglobulin (Ig) profiles in postnatal HIV transmission are scarce.

**Methods**: We conducted a nested case–control study within the multi‐country PROMISE (IMPAACT 1077BF/1077FF) trial. Cases were women with HIV (WWH) whose infants acquired HIV postnatally during breastfeeding (first positive nucleic acid test >2 weeks of age); controls were WWH whose breastfed infants remained without HIV. Each case was matched to two controls by site, type of follow‐up (postpartum randomization to maternal ART, postpartum randomization to infant prophylaxis or observational follow‐up) and specimen availability. Human milk samples collected at delivery and closest to but before infant HIV diagnosis were assayed for 19 HMOs using liquid chromatography and for Ig isotypes/subclasses, including HIV‐1 gp120–specific IgG, using ELISA. Conditional logistic regression models, adjusted for maternal age, plasma HIV RNA and CD4 count at delivery, and infant gestational age at birth, were used to estimate associations between standardized log10 transformed HMO and Ig levels with postnatal transmission.

**Results**: Among 19 case−dyads and 38 control−dyads, maternal and infant demographic characteristics were similar. While total HMOs were not associated with the odds of transmission, higher delivery levels of lacto‐N‐neotetraose (OR 0.46; 95% CI 0.20−1.05) and sialyllacto‐N‐tetraose b (OR 0.56; 95% CI 0.28−1.11) trended towards lower odds of transmission, although not statistically significant (Figure 1). In contrast, higher human milk IgG3 prior to infant HIV diagnosis (OR 3.42; 95% CI 1.02−11.53), and higher HIV‐1 gp120–specific IgG both at delivery (OR 8.91; 95% CI 1.57−50.69) and prior to infant HIV diagnosis (OR 4.38; 95% CI 1.24−15.51) were significantly associated with increased odds of transmission. Higher IgM prior to infant HIV diagnosis (OR 3.25; 95% CI 1.13−9.32) was also associated with increased odds of transmission (Figure 2).

**Conclusions**: Distinct human milk immune profiles may be associated with the risk of postnatal HIV transmission during breastfeeding. These findings suggest that human milk's immune composition may reflect and potentially interact with local viral dynamics, meriting further research.

**FIGURE 1:**
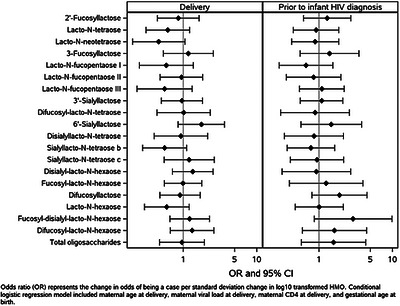
OAA2705 | Odds ratios‐HMOs.

**FIGURE 2:**
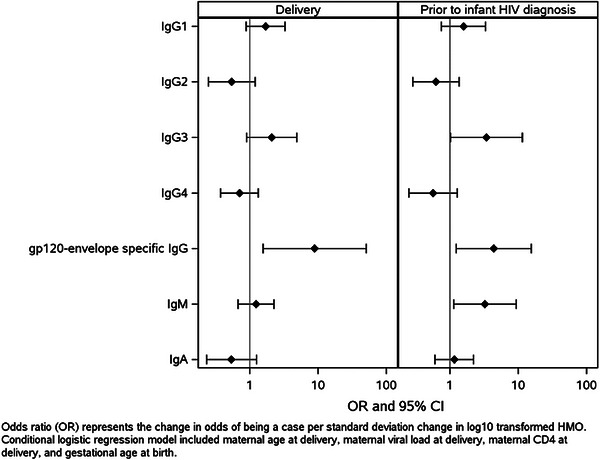
OAA2705 | Odds ratios‐Igs.

## OAA3002 Redirecting Antibody Specificity From the gp41 Base to the V3‐Glycan Epitope Through Env‐Based Immunization

M. Tarquis Medina ^1^, I. Relano Rodriguez^1^, C. Agostino^1^, A. Kriews^1^, M. Kerwin^1^, E. Urbano^1^, K. Dasteh Goli^1^, N. Laenger^1^, A. Aslanabadi^2^, Z. Zare^2^, M. G. Garay^1^, A. A. Walsh^3^, M. B. Melo^3^, D. B. Weiner^4^, D. W. Kulp^1^, M. M. Sajadi^2^, D. Fera^5^, A. Escolano^4^

^1^The Wistar Institute, Vaccine and Immune Therapy Center, Philadelphia, United States; ^2^Institute of Human Virology, University of Maryland School of Medicine, Baltimore, United States; ^3^Scripps Research Institute, Department of Immunology and Microbiology, La Jolla, United States; ^4^The Wistar Institute, Vaccine & Immune Therapy Center, Philadelphia, United States; ^5^Swarthmore College, Department of Chemistry and Biochemistry, Swarthmore, United States

**Background**: A major challenge in HIV‐1 vaccine development is that immunization with the viral Envelope (Env) protein predominantly elicits non‐neutralizing antibodies (Abs) targeting non‐conserved epitopes, which can outcompete broadly neutralizing antibody (bNAb) precursors in germinal centres. Among these responses, gp41 base–directed Abs are particularly frequent due to the high immunogenicity of this region in soluble Env immunogens. We observed that certain macaque gp41 base–binding Abs share notable amino acid sequence identity with human V3‐glycan bNAbs, suggesting that these off‐target lineages may retain latent potential for redirection. We, therefore, hypothesized that gp41 base–directed Abs could be redirected towards the V3‐glycan epitope and matured into bNAb‐like lineages through rational immunization strategies.

**Methods**: To test this hypothesis, we compared macaque gp41 base–binding Abs with known V3‐glycan bNAbs and selected the macaque base‐binding Ab1718 for its high sequence similarity to PGT121. We generated an immunoglobulin knock‐in (Ig KI) mouse model expressing Ab1718 and immunized these mice with engineered Env‐based immunogens designed to disfavour gp41 base recognition while promoting affinity maturation towards the V3‐glycan epitope. Antibody specificity and maturation were assessed using ELISA, liquid chromatography–mass spectrometry (LC‐MS) and flow cytometry. Single B‐cell antibody cloning and sequencing were used to assess acquisition of V3‐glycan bNAb‐like somatic mutations.

**Results**: ELISA and flow cytometry revealed a shift in serum antibody binding away from the gp41 base and towards the V3‐glycan region. LC‐MS and single B‐cell antibody sequencing showed that antibodies from the Ab1718 lineage acquired somatic mutations characteristic of V3‐glycan broadly neutralizing antibodies.

**Conclusions**: These data show that rational vaccine design can redirect off‐target antibody lineages towards conserved neutralizing epitopes of Env. We demonstrate that the specificity of a gp41 base–targeting antibody can be switched towards the V3‐glycan supersite through vaccination, driven by natural affinity maturation and germinal centre selection. Importantly, these findings indicate that some off‐target antibody responses are not immunological dead ends, but instead harbour latent features that can be leveraged to expand the pool of broadly neutralizing antibody precursor lineages. This strategy provides a promising framework for future HIV‐1 vaccine design and long‐term prevention efforts.

## OAA3003 Preclinical Data Supporting “First in Humans” Clinical Testing of V1‐Deleted HIV Envelope Immunogens in the Phase‐I CLEAR (Combined Long‐Term Efferocytosis and ADCC Responses) HIV Vaccine Trial

I. Silva de Castro^1^, M. Bissa^1^, M. A. Rahman^1^, L. Schifanella^1^, E. K. Woode^1^, T. Cardozo^2^, M. Robb^3^, T. Wellington^4^, J. Ake^5^, F. Maldarelli^6^, G. Franchini ^1^

^1^National Cancer Institute, Bethesda, United States; ^2^New York University Grossman School of Medicine, New York, United States; ^3^Henry M. Jackson Foundation for the Advancement of Military Medicine, Inc., Bethesda, United States; ^4^Walter Reed Army Institute of Research, Clinical Trials Center, Silver Spring, United States; ^5^Walter Reed Army Institute of Research, U.S. Military HIV Research Program, Silver Spring, United States; ^6^National Cancer Institute, Frederick, United States

**Background**: Studies in the SIV_mac251_ macaque model demonstrated removal of V1 from envelope immunogens (SIV‐ΔV1) elicits qualitatively different immunity and was more efficacious than WT envelope. Correlates of decreased risk included antibodies to prefusion helical conformation of V2 mediating ADCC, CD14^+^monocytes mediating efferocytosis and mucosal envelope‐specific IL‐17+NKp44^+^. Protective immunity was increased in the absence of V1. We investigated whether HIV ΔV1 immunogens exhibit similar properties, aiming to develop HIV ΔV1 envelope immunogens for testing in humans.

**Methods**: Study Design:

In vivo: Macaques were vaccinated with either WT HIV clade AE envelope immunogens (12 animals) or ΔV1‐deleted envelope (12 animals). Both groups were primed with WT or ΔV1A244 gp160 alongside *p55^gag^* plasmid DNA to form virus‐like particles. Both groups were boosted (weeks 8, 12) with ALVAC‐HIV vCP2438, expressing *gag‐pro* (HIV clade B) and gp120/TM (HIV clade C). At week 12, animals received WTA244 gp120 or ΔV1A244gp120 protein formulated in alum. Vaccinated animals and 11 naïve controls underwent up to 11 weekly intrarectal challenges with the clade C‐derived SHIV_1157(QNE)Y173H_ virus.

In vitro: Mucosal cell biopsies from naïve animals were exposed to WT HIV gp120/ΔV1 HIV gp120 or ΔV1 SIV gp120/SIV gp120 and analysed for functional and phenotypic responses.

**Results**: In vivo: ΔV1 vaccine reduced clade C SHIV_1157(QNE)Y173H_ mucosal acquisition risk by 81%, protecting 80% of animals. V2‐specific ADCC systemic mDC expressing CD73 correlated with protection. Mucosal correlates included CD73^+^DC‐10, CD14^+^ infiltrating cells and IL‐17^+^NKp44^+^ ILCs. In contrast, WT envelope vaccine was ineffective, elicited low anti‐envelope mucosal antibodies and high levels of IL‐15, CCR2+pDC and mucosal NKG2A‐NKp44‐ ILCs producing IFN‐γ+, correlating with increased risk.

In vitro: Stimulation of mucosal cells with SIV/HIV gp120 or without V1 elicited pro‐ and anti‐inflammatory responses, respectively.

**Conclusions**: Removal of V1 from envelope‐based SIV/HIV vaccine induces qualitatively distinct anti‐inflammatory immunity, potentially enhancing vaccine efficacy in humans. For details on the design and goals of the CLEAR Phase‐I HIV vaccine trial, see abstract from Luca Schifanella et al.

## OAA3004 Phase 1 Trial of Germline‐Targeting HIV‐1 DNA Vaccine Elicits CD4 Binding Site Specific Broadly Neutralizing Precursor Antibodies

S. Heath^1^, N. Naicker^2^, A. Sherman^3^, B. Furch^4^, W. Hahn^4^, M. Villaran^5^, L. Polakowski^6^, A. Takalani^7^, S. Hendricks^7^, C. Duplessis^6^, M. Miner^4^, R. Lederer^4^, N. McCloskey^4^, T. Glatt^8^, H. An^4^, C. Yu^4^, O. Hyrien^4^, J. Pallesen^9^, J. Huang^9^, S. Ghosh^9^, G. Ozorowski^10^, A. B. Ward^10^, K. R. Parks^4^, M. J. McElrath^4^, J. Heptinstall^11^, G. Tomaras^11^, D. Kulp^9^, J. Kublin^4^, L. Corey^4^, G. Ferrari^11^, D. Weiner^9^, T. Martin ^5^, HVTN 305 Study Team

^1^University of Alabama at Birmingham, Birmingham, United States; ^2^Center for the AIDS Programme of Research in South Africa, Durban, South Africa; ^3^Harvard University/Brigham and Women's Hospital, Boston, United States; ^4^Fred Hutchinson Cancer Center, Seattle, United States; ^5^Fred Hutchinson Cancer Institute, Seattle, United States; ^6^National Institutes of Health, Bethesda, United States; ^7^HIV Vaccine Trials Network (HVTN), Johannesburg, South Africa; ^8^South African National Blood Service, Johannesburg, South Africa; ^9^University of Pennsylvania/The Wistar Institute, Philadelphia, United States; ^10^The Scripps Research Institute, San Diego, United States; ^11^Duke University, Durham, United States

**Background**: HVTN 305 evaluated vaccination with a synthetic DNA self‐assembling eOD‐GT8 60‐mer nanoparticle co‐formulated with pIL‐12 (INO‐6172) to activate VRC01‐like CD4 binding site (CD4bs)‐specific germline B cell lineages. Heterologous boosting was performed with an adjuvanted soluble HIV‐1 envelope trimer. Here, we explore the robustness of this regimen to activate and boost humoral and cellular responses.

**Methods**: Forty‐six adults without HIV (19–55 years old) were enrolled in South Africa and the United States. Participants were randomized to one of three treatment groups: intradermal electroporation of (1) low dose (0.5 mg), (2) high dose (2.0 mg) or (3) high dose DNA given with the first three doses only and boosted intramuscularly with 3M‐052‐AF (5 mcg)/alum (500 mcg)/Trimer 4571 (100 mcg) in doses 3 and 4 only. Vaccines were given at 0, 1, 3 and 6 months (group 3 only). Humoral responses were assessed via differential binding antibody assay and electron microscopy‐based polyclonal epitope mapping (EMPEM). Vaccine‐specific B‐cell frequencies were determined by flow cytometry.

**Results**: Peak epitope‐specific Ab titres were observed in 83%−100% of participants after the first immunization, ranging between 16,000 and 24,000 AUC. With the next two doses, on‐target titres remained stable, whereas off‐target responses increased. Trimer 4571 boosting did not alter responses. While EMPEM of group 3 showed strong responses to the base of Trimer 4571 (100% response rate), there was no detectable CD4bs‐specific binding after the third or fourth vaccination. We observed peak response rates of epitope‐specific IgG+ memory B cells at month 3.5: 55.6% (group 1), 76.5% (group 2), 64.7% (group 3). Trimer 4571+ IgG+ memory B cells were only found in group 3 month 6.5 samples (94.4% response rate).

**Conclusions**: Synthetic DNA encoding eOD‐GT8 elicited antigen‐specific antibody and B‐cell responses supporting use of INO‐6172 as a CD4bs germline‐targeting immunogen. Trimer 4571 did not improve CD4bs specific responses. Ongoing work will further elucidate the presence of CD4bs antibody maturation through B cell receptor sequencing. Overall, our data indicate that DNA may be an effective platform to activate CD4bs germline targeting responses. Further work is underway to compare this vaccine with results from similar protein‐based and mRNA platforms.

## OAA3005 Multiepitope LNP‐HIV‐BMEP Priming and VLP‐Forming MVA Boosting to Initiate and Guide Broadly Neutralizing Antibody Responses Against HIV‐1

E. Álvarez ^1,2^, B. Perdiguero^1,2^, L. Marcos‐Villar^1,2^, L. Sin^1,2^, S. Guerra^3^, M. Esteban^1^, C. E. Gómez^1,2^

^1^National Centre for Biotechnology – Spanish National Research Council, Madrid, Spain; ^2^Biomedical Research Networking Center in Infectious Diseases (CIBERINFEC), Madrid, Spain; ^3^Autonomous University of Madrid, Madrid, Spain

**Background**: Despite four decades of research, induction of broadly neutralizing antibodies (bNAbs) by vaccination remains an unmet goal in the prevention of HIV‐1 acquisition. Most vaccine candidates fail to engage rare germline B‐cell receptors (BCRs) and preferentially expand non‐neutralizing B‐cell lineages. Passive administration of bNAbs protects against HIV‐1 acquisition in non‐human primates and humans, underscoring the need for immunization strategies that initiate and guide bNAb precursor maturation across conserved sites of viral vulnerability. We designed a combined approach using a multiepitope priming immunogen followed by a modified trimer candidate to evaluate their potential to elicit a broad and effective adaptive response.

**Methods**: We generated LNP‐HIV‐BMEP, a lipid nanoparticle‐formulated mRNA vector encoding the HIV‐BMEP multi‐epitope immunogen containing the recognition footprints of bNAbs targeting four conserved Env sites: the V1V2 glycan apex, CD4 binding site, fusion peptide and membrane‐proximal external region, to activate germline bNAb B‐cell precursors. As a boost, we generated MVA‐ConCv5‐KIKO‐GPN, a Gag‐induced VLP‐forming MVA vector co‐expressing a membrane‐tethered, prefusion‐stabilized, consensus clade C Env trimer and modified HIV‐1 Pol and Nef antigens. Protein expression and conformational integrity were assessed by western blot, flow cytometry, ELISA and transmission electron microscopy. BCR engagement was evaluated by calcium flux assays in Ramos B cells expressing lineage‐specific bNAb receptors. Immunogenicity was evaluated in C57BL/6 mice following a heterologous prime‐boost regimen of two LNP‐HIV‐BMEP primes and three MVA‐ConCv5‐KIKO‐GPN boosts, assessing the magnitude, breadth, and functionality of humoral and cellular immune responses.

**Results**: LNP‐HIV‐BMEP transfection induced stable secretion of HIV‐BMEP, while MVA‐ConCv5 GPN transduction resulted in surface expression of a closed Env trimer and production of Env‐decorated VLPs with preserved antigenic conformation. Both immunogens engaged bNAb BCRs in vitro in Ramos cells expressing lineage‐specific receptors. In mice, the prime‐boost regimen elicited strong binding antibody responses against multiple Env trimers and polyfunctional CD4^+^ and CD8^+^ T‐cell responses targeting Gag, Pol and Nef antigens.

**Conclusions**: This multiepitope LNP‐HIV‐BMEP prime and VLP‐forming MVA boost strategy supports coordinated humoral and cellular immunity and supports advancement to non‐human primate studies to evaluate bNAb maturation and protective efficacy within a translational framework relevant to global prevention of HIV‐1 acquisition and future clinical vaccine development (see Figure 1).

**FIGURE 1:**
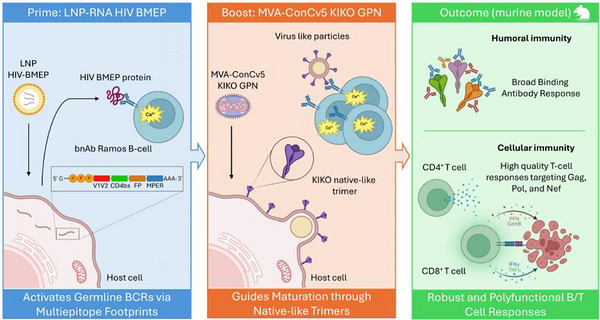
OAA3005

## OAA3802 Proviral and Inducible HIV‐1 Clade C Reservoir Activity in ART‐Suppressed Human Lymph Node Myeloid Cells: The Role of Bona‐Fide Germinal Centre Macrophages

M. Moodley ^1^, T. Hossain^2^, C. Chasara^1^, T. Khaba^3^, B. Mahlobo^1^, N. Reddy^1^, K. Reddy^1^, T. Ngubane^3^, J. Pansegrouw^4^, C. Mendoza‐Lopez^5^, P. Madlala^3^, T. Ndung'u^1,3,5^, T. Mahmoudi^2,6,7^, Z. Ndhlovu^1,3,5^

^1^Africa Health Research Institute, Durban, South Africa; ^2^Erasmus University Medical Center, Department of Biochemistry, Rotterdam, the Netherlands; ^3^University of KwaZulu‐Natal, HIV Pathogenesis Programme, Durban, South Africa; ^4^Prince Mshiyeni Memoria Hospital, Department of Surgery, Umlazi, South Africa; ^5^Ragon Institute of MGH, MIT, and Harvard, Cambridge, United States; ^6^Erasmus University Medical Center, Department of Urology, Rotterdam, the Netherlands; ^7^Erasmus University Medical Center, Department of Pathology, Rotterdam, the Netherlands

**Background**: Understanding whether lymph node (LN) myeloid cells, particularly germinal centre (GC) macrophages, function as HIV reservoirs relative to CD4+ T cells and peripheral blood (PB) represents an uncharacterized barrier to HIV cure. This study aimed to spatially characterize the phenotype of bona fide GC macrophages and assess whether they harbour HIV at the DNA, RNA and protein levels. Additionally, we assessed the functionality of the LN myeloid reservoir by quantifying proviral and inducible reservoir activity and comparing these findings to matched PB and CD4+ T cells in these compartments.

**Methods**: We analysed 42 excisional LNs from 39 people living with HIV (PLWH) clade C (89.7% female) from the FRESH and HPP LN Study cohorts in South Africa. Formalin‐fixed paraffin‐embedded LN tissues were used for super‐plex Lunaphore COMET imaging to spatially phenotype LN macrophages co‐expressing HIV Gagp24 and P17 proteins, while excluding B‐ and T‐cells. Dual DNAscope/RNAscope with immunofluorescence determined whether LN macrophages co‐express viral nucleic acids. Digital droplet PCR (ddPCR) quantified proviral DNA in LN myeloid cells and compared their contribution to that of CD4+ T cells. The Specific Quantification of Inducible HIV‐1 Reservoir by RT‐LAMP (SQuHIVLa) assay was used to quantify multiply spliced HIV RNA in sorted myeloid and CD4+ T cells in LNs and matched PB.

**Results**: Lymph node GC macrophages were predominantly IBA‐1+CD68+CD206−CD163−, and expressed HIV viral DNA, RNA and proteins while excluding B‐ and T‐cell markers. In ART‐suppressed individuals, proviral HIV DNA (3/6) and multiply spliced HIV‐1 RNA (msRNA) (3/3) confirmed inducible reservoir activity in LN myeloid cells. Compared to CD4+ T cells, myeloid cells harboured 1.6% (*p* = 0.0312) and 28.7% (*p* = 0.1250) of proviral DNA in ART‐suppressed and viraemic PLWH, respectively. LN myeloid cells had a reduced frequency of msRNA+ cells per million sorted cells compared to CD4+ T cells (*p* = 0.0176). Notably, LN frequencies of msRNA+ cells were higher than paired PB within both CD4+ T cells (4.98‐fold; *p* = 0.0085) and myeloid cells (35.04‐fold; *p* = 0.0585).

**Conclusions**: These findings identify and characterize functional HIV reservoir activity in LN myeloid cells, including bona‐fide IBA‐1+CD68+CD206−CD163− GC macrophages, enriched relative to PB, underscoring the importance of targeting LN tissue macrophages towards an HIV cure.

## OAA3803 Sustained Undetectable Intact Reservoir in Early Treated Infants in Africa

K. Reddy ^1,2^, N. Cotugno^3,4^, E. Morrocchi^3^, C. Molechan^1^, S. Dominguez‐Rodriguez^5^, S. Loubser^6^, C. T. Tiemessen^6^, S. Barnabas^7^, M. Grazia Lain^8^, K. Otwombe^9^, A. Maiga^10^, O. Behuhuma^1^, P. Rojo^5,11^, C. Giaquinto^12^, P. Rossi^4^, P. Palma^4^, T. Ndung'u^1,13,14^, A. Tagarro^5,15^

^1^Africa Health Research Institute (AHRI), Durban, South Africa; ^2^University of KwaZulu‐Natal, Durban, South Africa; ^3^Universitá di Roma Tor Vergata, Rome, Italy; ^4^Bambino Gesù Children Hospital, Clinical Immunology and Vaccinology Research Unit, Rome, Italy; ^5^Instituto de Investigación 12 de Octubre, Madrid, Spain; ^6^Centre for HIV and STIs, National Institute for Communicable Diseases, NHLS and the University of the Witwatersrand, Johannesburg, South Africa; ^7^Family Centre for Research with Ubuntu, Stellenbosch University, Department of Paediatrics and Child Health, Cape Town, South Africa; ^8^Fundação Ariel Glaser contra o SIDA Pediátrico, Maputo, Mozambique; ^9^Chris Hani Baragwanath Academic Hospital, University of the Witwatersrand, Perinatal HIV Research Unit, Soweto, South Africa; ^10^Centre Hospitalier Universitaire Gabriel Touré, Bamako, Mali; ^11^Universidad Complutense de Madrid, Madrid, Spain; ^12^Penta Foundation, Padova, Italy; ^13^Nelson R. Mandela School of Medicine, HIV Pathogenesis Programme (HPP), Durban, South Africa; ^14^University College of London, London, United Kingdom; ^15^Fundación Investigación Hospital Universitario Infanta Sofía, Madrid, Spain

**Background**: Our group and others have reported that 5%−10% of early ART‐treated infants with perinatally acquired HIV‐1 can achieve undetectable total HIV‐DNA measured by droplet digital PCR (ddPCR) in 5−10 million cells within the first years of life. However, reservoir measurement methods vary and require further validation to better understand reservoir heterogeneity, mechanisms of reservoir control and to identify candidates for cure‐related studies incorporating analytical treatment interruption (ATI).

**Methods**: We selected 11 infants from the EARTH/EPIICAL cohort (in Mozambique, South Africa and Mali) for further reservoir and host characterization, who 3 years after enrolment had undetectable total HIV DNA by ddPCR, sustained undetectable HIV viral load (VL) for >12 months and normal CD4>30% for >12 months. All infants started ART within 6 months of age, and were monitored at 2, 6, 12 and 24 weeks and biannually up to 4 years. In selected infants, we performed subtype C‐specific intact proviral DNA assay (IPDA), blood anti‐retroviral drug level testing and HLA typing. Lopinavir and ritonavir (LPV/r) levels were measured concomitantly with reservoir and VL.

**Results**: Among the 10 infants with HIV‐1 subtype C (1/11 had non‐C subtype), IPDA identified 5/10 (50%) with no detectable intact HIV DNA (0 copies/million PBMC) across ≥3 consecutive annual samples (up to five time points in three infants). The remaining 5/10 had low‐level intact HIV DNA (<20 copies/million PBMC), below the assay limit of detection (40 copies/million PBMC) in ≥2 consecutive annual samples after baseline. Protective HLA class I alleles were present in 8/10 infants, while all 10 carried alleles previously associated with HIV disease progression or acquisition. Caregiver reported ART adherence was >85% in 7/11 participants, 50%−85% in 3/11 and <50% in one (with five successive undetectable IPDA). All infants had LPV/r levels detectable, except one time point in one infant, a year post‐enrolment.

**Conclusions**: Early treated infants, particularly with protective HLA, can achieve durable undetectable intact proviral DNA, suggesting extremely small or absent intact reservoir. Those infants are strong candidates for ATI studies. Additionally, undetectable total HIV‐DNA by ddPCR reliably identified infants with absent or minimal intact provirus by IPDA, supporting ddPCR as an effective initial screening tool for cure‐related studies.

## OAA3804 Xevinapant, a SMAC Mimetic, Depletes the HIV‐1 Reservoir and Delays Viral Rebound In Vivo Without Reversing HIV‐1 Latency

L. Wang ^1,2^, M. Dayton^1^, L. Mackiewicz^1^, A. Tan^3^, J. Ong^3^, W. Clow^1,2^, R. Bhandari^1,2^, M. Roche^3^, S. R. Lewin^3,4,5^, K. Davidson^1,2^, M. Pellegrini^1,2,6^, M. Doerflinger^1,2^

^1^Walter and Eliza Hall Institute of Medical Research, Division of Infection and Global Health, Parkville, Australia; ^2^The University of Melbourne, Department of Medical Biology, Parkville, Australia; ^3^The University of Melbourne at the Peter Doherty Institute for Infection and Immunity, Department of Infectious Diseases, Parkville, Australia; ^4^The Royal Melbourne Hospital at the Peter Doherty Institute for Infection and Immunity, Victorian Infectious Diseases Service, Melbourne, Australia; ^5^Alfred Hospital and Monash University, Department of Infectious Diseases, Melbourne, Australia; ^6^The University of Sydney, Centenary Institute, Camperdown, Australia

**Background**: The HIV‐1 reservoir is responsible for viral rebound upon ART interruption, making HIV‐1 infection a chronic, lifelong disease. Recent ex vivo studies suggest that HIV‐1 reservoir may resist cell death, making IAP (inhibitor of apoptosis) proteins potential targets. In this project, we used Xevinapant (Debio‐1143), a SMAC Mimetic with favourable bioavailability and toxicity profiles in cancer clinical trials, to target the pro‐survival IAP proteins in HIV‐1‐infected cells. We hypothesize that SMAC Mimetic treatment will sensitize HIV‐1‐infected cells to undergo apoptosis, leading to a reduction in the HIV‐1 reservoir in vivo.

**Methods**: We used a humanized immune system (HIS) mouse model to study HIV‐1 infection in vivo. Newborn NOD/SCID/IL2rγ^null^ (NSG) mice were irradiated and engrafted with human CD34^+^ cord blood cells. After 16 weeks of reconstitution, mice were infected with HIV‐1_JRCSF_. 3 weeks post infection, mice began antiretroviral therapy (ART) treatment. After HIV‐1 suppression (PVL<200 cps/mL), mice were given either vehicle or 100 mg/kg Xevinapant by oral administration for 6 weeks along with ART. After treatment, we assessed the efficacy of Xevinapant using either a 4‐week analytical treatment interruption (ATI) or the intact proviral DNA assay (IPDA).

**Results**: Xevinapant did not show overt toxicity based on CD4^+^ T‐cell counts, total immune cell counts and body weight. 6 weeks of Xevinapant treatment delayed HIV‐1 rebound by a median of 7 days (*p* = 0.003) compared with the vehicle group. Xevinapant also significantly reduced (*p* = 0.003) HIV‐1 intact proviral load in spleens and lymph nodes in HIS mice by over 85%. Xevinapant did not increase cell‐associated HIV‐1 RNA (*long LTR, Pol, Poly A and Tat‐Rev*) and plasma viral load in HIS mice.

**Conclusions**: Our results show that Xevinapant can deplete the HIV‐1 reservoir in vivo. Unlike some other SMAC Mimetics, Xevinapant did not reverse HIV‐1 latency in HIS mice. Taken together, our results demonstrate the depletion of the HIV‐1 reservoir in vivo by a SMAC Mimetic independent of latency reversal, suggesting that exploiting the extrinsic apoptosis pathway offers a new direction towards an HIV‐1 cure (see Figures 1 and 2).

**FIGURE 1:**
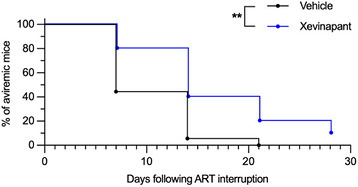
OAA3804 | Xevinapant treatment delayed HIV rebound in HIS mice (*n* = 38).

**FIGURE 2:**
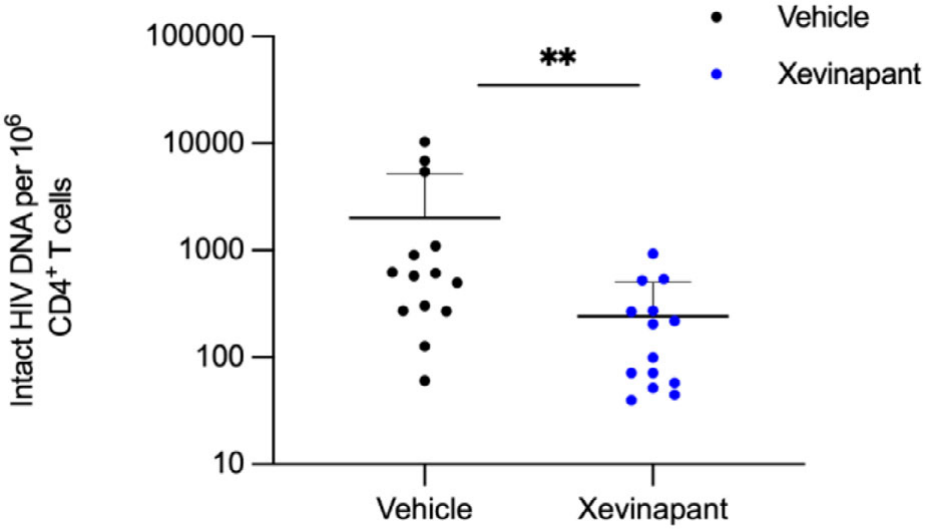
OAA3804 | Xevinapant treatment reduced intact proviral DNA load in HIS mice (*n* = 28).

## OAA3805 Integration Site Determines the Transcriptional Fate and Persistence of Integrated Proviruses

V. K. Pal^1^, A. Danesh^2^, M. Canis^1^, R. T. Dilling^2^, G. I. Miller^2^, T. T. Huynh^2^, D. Copertino^2^, D. Barrows^3^, T. Carroll^3^, T. Hatziioannou^1^, R. B. Jones^2^, G. Q. Lee^2^, F. Muecksch ^1,4^, P. D. Bieniasz^1,5^

^1^The Rockefeller University, Laboratory of Retrovirology, New York, United States; ^2^Weill Cornell Medical College, Division of Infectious Diseases, Department of Medicine, New York, United States; ^3^The Rockefeller University, Bioinformatics Resource Center, New York, United States; ^4^Heidelberg University, Department of Infectious Diseases, Virology, Heidelberg, Germany; ^5^Howard Hughes Medical Institute, The Rockefeller University, New York, United States

**Background**: The persistence and expansion of latent HIV‐1 reservoirs during antiretroviral therapy (ART) remain major barriers to HIV cure strategies. Proviral integration site selection has been proposed to influence both transcriptional activity and clonal expansion of infected cells, but causal mechanisms remain incompletely understood. We aimed to define how genomic and epigenomic features at proviral integration sites contribute to HIV‐1 latency and clonal persistence in vivo.

**Methods**: We established a novel model system in which populations of human memory CD4+ T cells, each bearing a single transcriptionally active HIV‐1 provirus, are engrafted into immunodeficient mice and followed for 2 months during which latency occurred in a subset of engrafted cells. Combining this model with cell sorting of latent cells and a method for efficient detection of integration sites and quantification of clonal expansion (PRISM‐seq), we determined how host‐specific genomic and epigenomic features associated with each distinct HIV‐1 integration site affect clonal survival and proviral expression in the absence of immunological or anti‐viral drug selection pressures.

**Results**: A total of 5239 and 3152 unique integration sites were mapped in pre‐engraftment and post‐engraftment cell populations, respectively, with extensive clonal expansion detected at >2 months after engraftment. Importantly, the integration site profile in cells harvested from mouse grafts mirrored that prior to engraftment and the observed clonal expansion was driven by T‐cell receptor identity rather than proviral insertional mutagenesis. Proviruses were detected in transcriptionally active, latent or transitioning cell populations. The integration sites of proviruses that became latent in vivo were enriched on chromosome 19, in intergenic and centromeric satellite regions, and genes whose expression is atypically low such as Znf genes. Pre‐existing repressive epigenetic features were associated with latency for subsets of proviruses.

**Conclusions**: These findings demonstrate that HIV‐1 latency and clonal persistence in vivo are shaped by a convergence of genomic location and epigenomic context rather than insertion‐driven growth advantages. Integration into transcriptionally repressive regions, or genes with low levels of expression including ZNF loci, promotes stable latency and mirrors patterns observed in people living with HIV on long‐term ART. Understanding these determinants has important implications for HIV reservoir targeting, reactivation strategies and curative interventions.

## OAB0102 Final Assessment of Real‐World Virological Effectiveness of Dolutegravir Versus Bictegravir Triple Regimens Among People Living With HIV and High Viral Load: The BicDol Study in the French Dat'AIDS Cohort

A. Chéret ^1,2^, B. Tressières^2^, I. Lamaury^3^, M. Hentzien^4^, C. Duvivier^5,6^, A. Makinson^7^, J. Reynes^7^, R. Palich^8^, A. Menard^9^, V. Martinez‐Pourcher^10,11^, F. Bani‐Sadr^4^, V. Avettand‐Fènoël^12,13^, L. Cuzin^14,15^, L. Hocqueloux^16^, C. Delpierre^15^, C. Allavena^17^

^1^Universitary Hospital of Guadeloupe, Plateforme de Diagnostic et de Thérapeutique Pluridisciplinaire, Pointe à Pitre, France; ^2^Universitary Hospital of Guadeloupe, INSERM‐CIC‐2504, Pointe a Pitre, France; ^3^Universitary Hospital of Guadeloupe, Infectious diseases Unit, Pointe a Pitre, France; ^4^University Hospital of Reims, Department of Internal Medicine, Infectious Diseases and Clinical Immunology, Reims, France; ^5^Necker Hospital, AP‐HP, Infectious Diseases Department, Paris, France; ^6^Institut Pasteur Paris, Necker‐Pasteur Infectiology Center, Paris, France; ^7^Universitary Hospital of Montpellier, Infectious Diseases Department, Unité Inserm U1175, Montpellier, France; ^8^Hotel‐Dieu Hospital AP‐HP, Paris Cité University, Immunology and Infectious Diseases Department, Paris, France; ^9^IHU – Méditérranée Infection, Infectious Diseases Department, Marseille, France; ^10^Hôpitaux Universitaires Pitié Salpêtrière‐Charles Foix, AP‐HP, Infectious Diseases Unit, Paris, France; ^11^Sorbonne Université, INSERM, Institut Pierre Louis d'Epidémiologie et de Santé Publique, Paris, France; ^12^Universitary Hospital of Orléans, Virology Department, Orléans, France; ^13^Orléans University, LI2RSO, Orléans, France; ^14^Universitary Hospital of Martinique, Infectious Diseases Unit, Fort De France, France; ^15^Toulouse University, CERPOP, Inserm‐UMR1295, UPS, Toulouse, France; ^16^Universitary Hospital of Orléans, Infectious Diseases Unit, Orléans, France; ^17^Universitary Hospital of Nantes, Infectious Diseases Department, INSERM EA1413, Nantes, France

**Background**: Data on bictegravir (BIC) plus two NRTIs as first‐line therapy in PWH (people living with HIV) with baseline HIV‐RNA ≥5 log₁₀ copies/mL (pVL5) remain limited. Moreover, there is a greater early viral load decline after 10 days of monotherapy with DTG (dolutegravir) (−2.46 log₁₀) versus BIC (−2.06). Preliminary BicDol data suggested faster virological suppression (VS) with DTG‐ versus BIC‐based regimens in pVL5 including acute HIV infection (pAHI) (EACS 2025, MeP05.1). Here, we report the final result.

**Methods**: Within the French Dat'AIDS cohort (2009−2023), we compared BIC‐ versus DTG‐based 3‐drug regimens in pVL5, pVL5.7 (baseline HIV‐RNA ≥5.7 log₁₀; 500,000 cp/mL) and pAHI. A propensity score was calculated on age, sex, AHI, CD4‐T count and baseline HIV‐RNA. Time to VS (<50 copies/mL) was analysed in intention‐to‐treat (ITT, overall follow‐up) and per‐protocol (PP, first‐line ARV follow‐up censoring at treatment discontinuation or switch), using weighted Kaplan–Meier curves. Hazard ratios (HR) were estimated with Cox models. To account for treatment changes, Cox models with time‐dependent covariates were fitted.

**Results**: DTG‐group (*n* = 703) and BIC‐group (*n* = 1826) were predominantly men (Table 1). Timing of HIV‐RNA measurements did not differ between groups (*p* = 0.23). In ITT and PP, VS was achieved more rapidly in DTG‐group (ITT: HR = 1.09 [1.00−1.20] *p* = 0.056; PP: HR = 1.15 [1.04−1.30] *p* = 0.007), with 5.7% more PWH reaching VS at week‐24 (7% PP). This effect was more pronounced in pAHI (ITT: HR = 1.24 [1.03−1.48] *p* = 0.024; PP: HR = 1.24 [1.00−1.50] *p* = 0.038) and pVL5.7 (ITT: HR = 1.14 95% CI [1.00−1.30] *p* = 0.054; PP: HR = 1.2 [1.04−1.40] *p* = 0.015), with week‐24 differences of 9.3% (10.2% PP) and 8.3% (10.3% PP), respectively. Results were reinforced by time‐dependent Cox models highlighting an effect up to week‐30 (Figure 1). CD4/CD8 ratio rose faster in DTG versus BIC during first 5 weeks (*p* = 0.030); overall CD4 gain kinetics were similar.

**Conclusions**: In real‐world data, first‐line DTG‐ and BIC‐based triple regimens were highly effective in PWH with high pretherapeutic HIV‐RNA. However, virological suppression was achieved significantly faster with DTG than with BIC. These results could be considered, particularly in the context of acute HIV infection and very high viraemia.

**TABLE 1:**
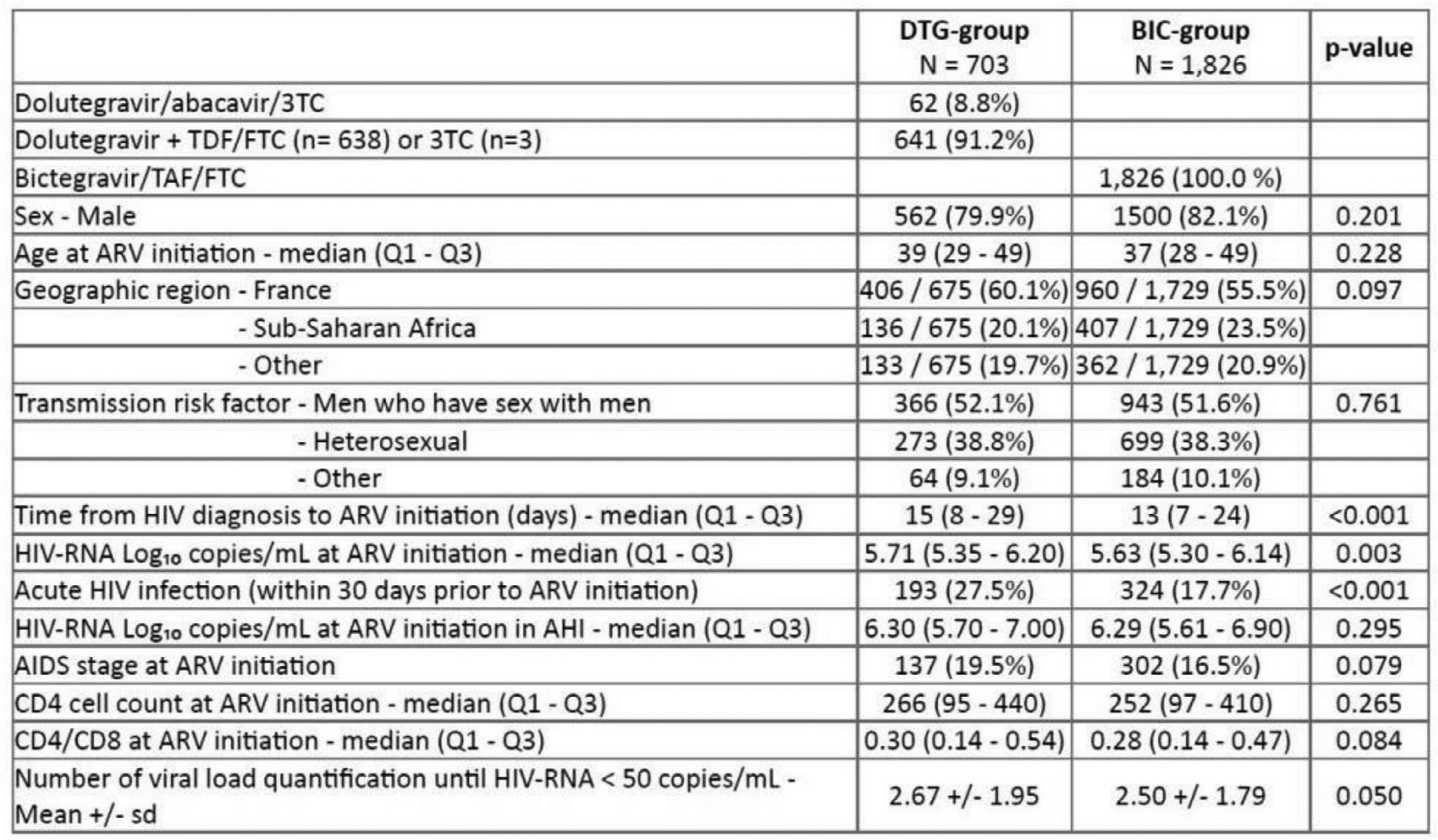
OAB0102 | Population characteristics.

**FIGURE 1:**
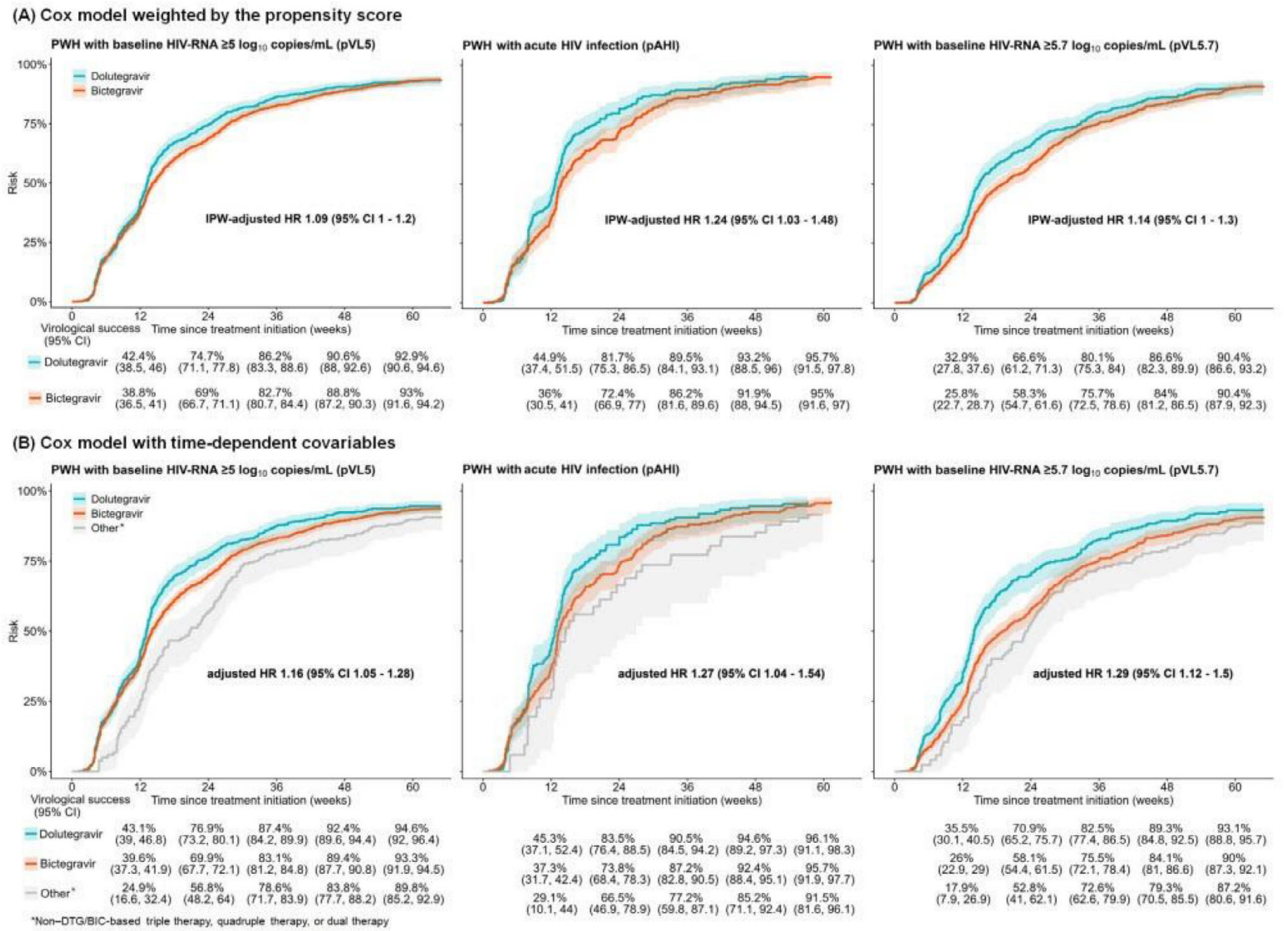
OAB0102 | Kaplan−Meier survival curves for time to HIV‐RNA < 50 copies/mL (intention‐to‐treat).

## OAB0103 Long‐Term Efficacy and Safety of Bictegravir/Emtricitabine/Tenofovir Alafenamide (B/F/TAF) in Children and Adolescents With HIV‐1 Aged 2 to <18 Years

E. Natukunda ^1^, K. Chokephaibulkit^2^, S. Fry^3^, E. Hellström^4^, P. Kosalaraksa^5^, U. Lalloo^6^, A. Liberty^7^, C. A. Rodriguez^8^, R. Strehlau^9^, K. Kersey^10^, A. Sudhakaran^10^, V. A. Vieira^10^, A. H. Gaur^11^

^1^Joint Clinical Research Centre, Kampala, Uganda; ^2^Faculty of Medicine Siriraj Hospital, Mahidol University, Bangkok, Thailand; ^3^FAMCRU, Stellenbosch University, Cape Town, South Africa; ^4^Be Part Yoluntu Centre, Paarl, South Africa; ^5^Faculty of Medicine, Khon Kaen University, Khon Kaen, Thailand; ^6^Enhancing Care Foundation, Durban University of Technology, Durban, South Africa; ^7^Perinatal HIV Research Unit, Chris Hani Baragwanath Hospital, Johannesburg, South Africa; ^8^Morsani College of Medicine, University of South Florida, Tampa, FL, United States; ^9^University of the Witwatersrand, Johannesburg, South Africa; ^10^Gilead Sciences, Inc., Foster City, CA, United States; ^11^St. Jude Children's Research Hospital, Memphis, TN, United States

**Background**: Children and adolescents with HIV‐1 have limited treatment options and often experience high pill burden. B/F/TAF is a well‐tolerated, once‐daily single‐tablet regimen with a high barrier to resistance, approved for children with HIV‐1 aged ≥2 years and weighing ≥14 kg.

**Methods**: Virologically suppressed (HIV‐1 RNA <50 copies/mL) children and adolescents with HIV‐1 enrolled in Cohorts 1−3 of a Phase 2/3 open‐label study evaluating B/F/TAF (NCT02881320). Participants were grouped by age and weight band at screening: 12−<18 years and ≥35 kg (Cohort 1); 6−<12 years and ≥25 kg (Cohort 2); ≥2 years and ≥14–<25 kg (Cohort 3). Cohorts 1 and 2 received B/F/TAF 50/200/25 mg; Cohort 3 received B/F/TAF 30/120/15 mg. Long‐term efficacy and safety outcomes of B/F/TAF were assessed.

**Results**: Overall, 122 participants (Cohort 1: *n* = 50, Cohort 2: *n* = 50, Cohort 3: *n* = 22) from South Africa, Thailand, Uganda and the USA were enrolled. At baseline, median (range) ages were 15 (12−17), 10 (6−11) and 6 (3−9) years in Cohorts 1, 2 and 3, respectively; 64%, 54% and 50% of participants were female; and 65%, 72% and 73% were Black. Median (Q1, Q3) exposure to B/F/TAF was 392 (156, 408) weeks, 371 (237, 390) weeks and 279 (264, 301) weeks. Of the 89 participants with available HIV‐RNA data at Week 240, a single participant had HIV‐RNA >50 copies/mL; CD4% remained stable across cohorts (Table 1). No new safety concerns emerged. Most treatment‐emergent adverse events (TEAEs) were mild or moderate in severity. One participant in Cohort 1 experienced serious TEAEs (Grade 3 upper abdominal pain, nausea and vomiting on Day 1957) that were considered related by the investigator; study drug was resumed 5 days later. No Grade 3−4 drug‐related TEAEs or serious drug‐related TEAEs were reported in other cohorts. Three participants discontinued study drug due to TEAEs, one of whom experienced two drug‐related TEAEs (insomnia and worsening anxiety). There were no clinically significant changes in growth, metabolic and renal parameters (Table 1).

**Conclusions**: After 5 years of treatment, B/F/TAF demonstrated favourable long‐term efficacy and safety in children and adolescents with HIV‐1 aged ≥2 years and weighing ≥14 kg.

**TABLE 1:**
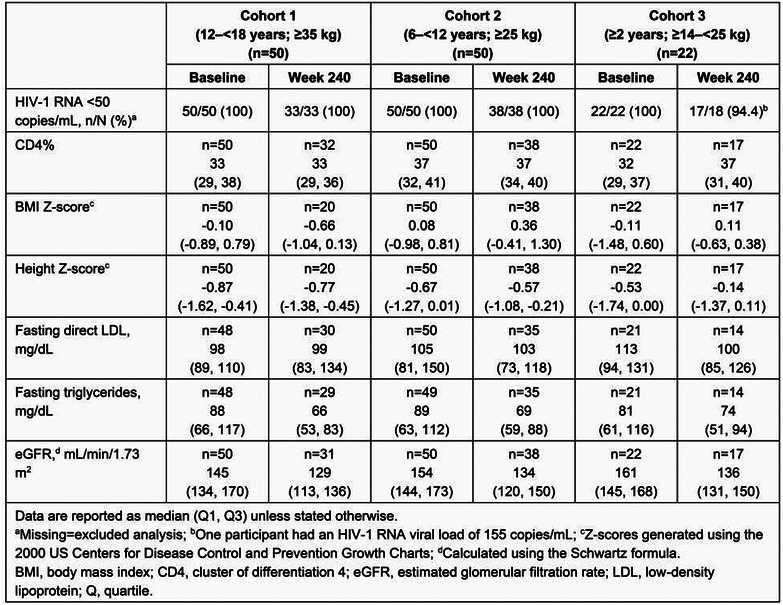
OAB0103 | Clinical outcomes at baseline and week 240.

## OAB0104 Point of Care Viral Load Monitoring Improves Viral Suppression at 6 Months Among Children, Adolescents and Young People With HIV in East Africa: A Cluster‐Randomized Trial

B. Kikaire ^1,2^, B. Watyaba^1^, B. Nyawanda^3^, A. Mtenga^4^, J. Berchmans Niyibizi^5^, R. Urio^6^, V. Mudhume^7^, M. Senkoro^8^, S. Mfinanga^8^, Y. Mayanja^9^, P. Kaleebu^1^

^1^Uganda Virus Research Institute, Kampala, Uganda; ^2^Makerere University, Paediatrics and Child Health, Kampala, Uganda; ^3^Kenya Medical Research Institute, Kisumu, Kenya; ^4^Kilimanjaro Clinical Research Institute, Moshi, the United Republic of Tanzania; ^5^University of Rwanda, Kigali, Rwanda; ^6^Management and Development for Health, Dar‐es Salaam, the United Republic of Tanzania; ^7^Kenya Medical Research Institute, Moshi, Kenya; ^8^National Institute of Medical Research, Dar‐es Salaam, the United Republic of Tanzania; ^9^MRC/UVRI & LSHTM Uganda Research Unit, Kampala, Uganda

**Background**: In sub‐Saharan Africa, 75% of women and 69% of men aged 15 years or more and living with HIV are virally suppressed. Challenges associated with centralized viral load (VL) monitoring, including delayed turn‐around time and sample loss, potentially explain the low VL suppression rates in the region. We assessed the effect of point‐of‐care viral load (PoC‐VL) monitoring on VL suppression among virally unsuppressed children, adolescents and young people (CAYP) living with HIV in East Africa.

**Methods**: We conducted a cluster‐randomized trial within the East Africa Consortium for Clinical Research across 28 health facilities (clusters) in Uganda (8), Kenya (4), Tanzania (12) and Rwanda (4). Facilities were randomized (1:1) to PoC‐VL monitoring using Abbott m‐PIMA or Cepheid GeneXpert platforms or centralized monitoring (standard of care). Eligible participants were CAYP aged 6 months to 24 years on antiretroviral therapy (ART) with detectable VL within 6 months prior to enrolment. Viral load testing was performed at 6 and 12 months. Viral suppression was defined as <50 copies/mL. Mixed‐effects logistic regression models accounting for clustering were used to compare suppression between study arms using an intention‐to‐treat approach.

**Results**: A total of 956 participants were enrolled; 527 (55.2%) were female and 399 (41.7%) were aged 13–19 years. Overall, 415 (43.4%) had been on ART for more than 10 years, and 766 (80.1%) were receiving first‐line ART. At 6 months, 422 (48.9%) participants achieved viral suppression, with higher suppression in the PoC‐VL arm compared to standard of care (54.6% vs. 42.8%; *p* = 0.001). PoC‐VL monitoring increased the odds of viral suppression at 6 months (adjusted OR 1.71, 95% CI 1.25–2.33; *p* < 0.001). At 12 months, 405 (50.4%) participants were virally suppressed, with no difference between study arms (50.7% vs. 50.1%; adjusted OR 1.11, 95% CI 0.80–1.52; *p* = 0.54). PoC‐VL improved time to clinical action and turnaround time for results.

**Conclusions**: PoC‐VL monitoring significantly improved viral suppression among CAYP at 6 months but did not sustain this effect at 12 months. Persistently low suppression at 1 year highlights the need for complementary interventions, including enhanced adherence and differentiated care models, to optimize long‐term viral suppression among CAYLHIV.

## OAB0902 The SAFE (Steatosis‐Associated Fibrosis Estimator) Score Predicts Incidence of Advanced Liver Fibrosis in People Living With HIV: A Prospective Analysis From the PROSPEC‐HIV Cohort

H. Perazzo, S. Cardoso, E. Nunes, J. Fittipaldi, C. De‐Almeida, P. De‐Brito, V. Veloso, B. Grinsztejn

Oswaldo Cruz Foundation (FIOCRUZ), Evandro Chagas National Institute of Infectious Diseases (INI), Rio de Janeiro, Brazil

**Background**: The recently developed artificial‐intelligence‐based Steatosis‐Associated Fibrosis Estimator (SAFE) score can be used to stratify people at risk of liver fibrosis. We aimed to evaluate the prognostic value of the SAFE score to predict incidence of advanced liver fibrosis in people living with HIV (PLWH).

**Methods**: ​We analysed data from the PROSPEC‐HIV cohort (NCT02542020), where 744 PLWH have been followed every 36 months since June‐2015 with questionnaires, blood sample and transient elastography (TE‐Fibroscan) on the same day. The presence of advanced fibrosis was defined by liver stiffness measurement (LSM) ≥9.5 kPa by TE. The SAFE score was calculated using age (years), BMI (kg/m^2^), diabetes (yes/no), AST/ALT ratio (U/L), globulin (g/dL) and platelet count (10^9^/mm^3^). People with unreliable TE‐exam (*n* = 13) and those with missing data for SAFE score calculation at baseline (*n* = 39); as well as those with advanced fibrosis at baseline (*n* = 62) and those with loss‐of‐follow‐up (*n* = 206) were excluded. The SAFE score < 0, between 0−100 and > 100 were defined as low, intermediate and high risk of fibrosis, respectively. The primary outcome was the incidence of advanced fibrosis (LSM ≥9.5 kPa) during follow‐up. Participants without advanced fibrosis during follow‐up were censored at the last visit up to 30 June 2025. Kaplan−Meier curves and Cox proportional hazards models were performed.

**Results**: 424 PLWH (53% female, median age = 44 [IQR,36−52] years, 19% with BMI>30 Kg/m^2^, 10% with viral hepatitis coinfection) were included. A total of 28 participants (6.6%) developed advanced fibrosis (incidence = 8.8 events per‐1000‐PY) during a median follow‐up of 8.0 years (IQR, 6.8−8.7). The incidence‐rate was higher in people with high‐SAFE compared to those without (19 vs. 7 events per‐1000‐PY; RR = 2.71 [95% CI, 1.23−5.99]). People with a high SAFE score (>100) had higher cumulative incidence of advanced fibrosis compared to those with intermediate (0−100) or low score (<0) (13.8% vs. 6.7% vs. 3.1%, log‐rank‐*p* = 0.008) (Figure 1).

The high‐SAFE score (vs. low‐SAFE) predicted incidence of advanced fibrosis in univariate Cox‐model (HR = 4.66 [95% CI, 1.66−13.09]) and adjusted for viral hepatitis coinfection and metabolic‐liver disease (HR = 3.35 [95% CI, 1.54−7.28]).

**Conclusions**: The SAFE score can be a simple non‐invasive tool to identify PLWH at increased risk of developing advanced liver fibrosis in a long‐term follow‐up.

**FIGURE 1:**
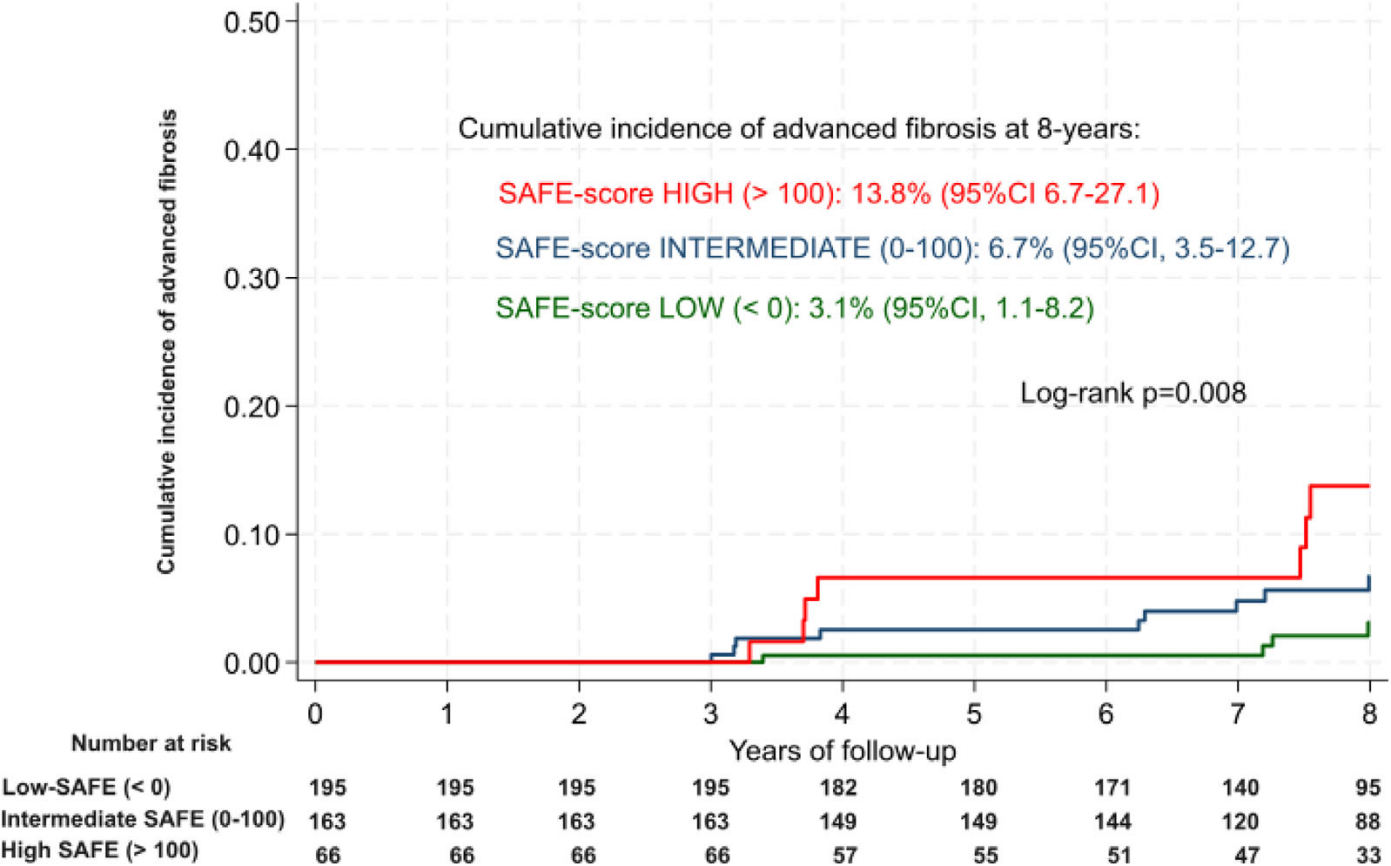
OAB0902

## OAB0903 Evaluation of Hepatitis B Virus Epidemiology and Impact of Select Demographic Factors on Seroprevalence Within an Urban Clinic in the United States

H. Jimenez ^1,2^, M. Abbasi^1^, F. Chuy^1^, S. Moawed^1^, J. S. Suh^2^

^1^Ernest Mario School of Pharmacy, Rutgers University, East Brunswick, United States; ^2^St. Joseph's University Medical Center, Comprehensive Care Center, Paterson, United States

**Background**: Hepatitis B virus (HBV) remains a concern among people with HIV (PWH). Knowing current serostatus is critical as newer antiretroviral options (long‐acting, dual therapy) may not contain tenofovir, placing individuals at risk of HBV acquisition or reactivation. This study identified the prevalence of HBV immunity and chronic infection, as well as evaluated patient‐specific variables impact on seroprevalence at an urban clinic in the United States.

**Methods**: A retrospective analysis of 1168 PWH patients receiving HIV care at a clinic in the United States between 8/2023 and 7/2024. Demographic characteristics and HBV serologic data were collected. HBV status was defined based on serologic data (surface antigen, surface antibody, core antibody) results. Associations between demographic variables and HBV categories were assessed utilizing Chi‐square tests.

**Results**: Patients were predominantly cisgender male (63.4%), Hispanic (56.3%) and ≥60 years of age (28.1%). The analysis found a widely varied distribution based on gender and age differences (*p* < 0.001) across racial groups. Black individuals with HIV comprised the highest proportion of females (47.0%) and had a higher mean age (51.8% of those ≥60 years). Meanwhile, Hispanic patients comprised the majority of younger age groups (66.1% of 18−29 years, 70.9% of 30−39 years).

Approximately half were not immune to HBV (49.0%), 41.7% demonstrated immunity (41.7%), 2.1% had chronic HBV infection and 7.2% had isolated anti‐HBc (unknown HBV status). Across all age groups, a significant proportion lacked HBV immunity (Tables 1 and 2). Furthermore, age‐ and race‐related differences in HBV immunity were observed (*p* = 0.001). Expectantly, immunity prevalence decreases with age ([18−29 years, 51.6%], [30−39, 49.8%], [40−49, 43.8%], [50−59, 36.3%], *p* < 0.001).

**Conclusions**: HBV serostatus remains an important component of HIV care. Despite a decreasing national prevalence of HIV/HBV coinfection, populations remain at risk of HBV acquisition. Efforts should be made to optimize immunity and monitoring ART for those with HIV/HBV coinfection. Our findings highlight demographic variability in epidemiology among PWH receiving care in a given community. Further research is warranted to evaluate age‐ and race‐related differences within seroconversion of patients and immunization records.

**TABLE 1:** OAB0903 | Baseline HBV seroprevalence by race/ethnicity.

Characteristic	Overall (*N* = 1168)	Asian (*n* = 8)	Black, non‐Hispanic (*n* = 432)	Hispanic (*n* = 658)	White, non‐Hispanic (*n* = 70)
Chronic HBV	25 (2.1%)	0 (0.0%)	13 (3.0%)	11 (1.7%)	1 (1.4%)
Immune	487 (41.7%)	5 (62.5%)	173 (40.0%)	276 (42.0%)	33 (47.1%)
Isolated	84 (7.2%)	0 (0.0%)	50 (11.6%)	28 (4.3%)	6 (8.6%)
Not immune	572 (49.0%)	3 (37.5%)	196 (45.4%)	343 (52.1%)	30 (42.9%)

*p*‐value <0.001.

**TABLE 2:** OAB0903 | Baseline HBV seroprevalence by age group.

Characteristic	Overall (*N* = 1168)	18−29 years (*n* = 124)	30−39 years (*n* = 237)	40−49 years (*n* = 217)	50−59 years (*n* = 262)	60+ years (*n* = 328)
Chronic HBV	25 (2.1%)	0 (0.0%)	5 (2.1%)	2 (0.9%)	10 (3.8%)	8 (2.4%)
Immune	487 (41.7%)	64 (51.6%)	118 (49.8%)	95 (43.8%)	95 (36.3%)	115 (35.1%)
Isolated anti‐HBc	84 (7.2%)	0 (0.0%)	5 (2.1%)	7 (3.2%)	15 (5.7%)	57 (17.4%)
Not immune	572 (49.0%)	60 (48.4%)	109 (46.0%)	113 (52.1%)	142 (54.2%)	148 (45.1%)

*p*‐value <0.001.

## OAB0904 Liver Transplantation in People With HIV: Long‐Term Survival, Rejection Trends, HCC Recurrence and Metabolic Outcomes

A. Cervo ^1^, M. Albertini^1^, F. Casari^1^, J. Milic^1^, B. Catellani^2^, E. Franceschini^1,3^, G. P. Guerrini^2^, C. Mussini^1,3^, F. Di Benedetto^2,3^, G. Guaraldi^1,3^

^1^Infectious Diseases Clinic, University Hospital of Modena, Modena, Italy; ^2^Hepato‐Pancreato‐Biliary Surgery and Liver Transplantation Unit, University Hospital of Modena, Modena, Italy; ^3^University of Modena and Reggio Emilia, Modena, Italy

**Background**: Data on long‐term outcomes and post‐transplant comorbidities in liver transplant (LT) recipients living with HIV remain limited, particularly regarding post‐LT hepatocellular carcinoma (HCC) recurrence and metabolic dysfunction–associated steatotic liver disease (MASLD).

**Methods**: This retrospective, propensity score–matched (1:3) case–control study included all consecutive 87 LT recipients with HIV and 261 without HIV transplanted at the University Hospital of Modena between 2003 and 2024. Matching variables were age, sex at birth, MELD, HCC, HBV/HCV and donor age. Long‐term survival and rejection were evaluated together with HCC recurrence and post‐LT MASLD prevalence, assessed by imaging.

**Results**: Of 348 recipients, 17% were females, with a median age of 53 (IQR 47−58). At LT, active HCV replication was present in 39% of HIV and 31% of non‐HIV recipients, and HCC prevalence was comparable (49.4% vs. 47.5%). Ten‐ and 15‐year survival was 59.6% and 48.9% in HIV versus 66.7% and 53.1% in non‐HIV (Figure 1a). The incidence rate (IR) of rejection was higher in HIV recipients (5.5 [95% CI 3.3−9.3] vs. 0.8 [95% CI 0.5−1.2] per 100 person‐year follow‐up [PYFU]), with HIV serostatus, CMV infection/reactivation and HCV replication as major predictors. After 2015, rejection IRs were comparable between HIV and non‐HIV recipients (1.1 vs. 0.9 per 100 PYFU), with CMV infection/reactivation as the sole risk factor (Table 1 and Figure 1b,c). HCC recurrence occurred at similar rates (1.8 [95% CI 1.0−3.5] vs. 1.4 [95% CI 1.0−2.1] per 100 PYFU in HIV and non‐HIV, respectively), significantly impairing survival in both groups. However, recurrence tended to occur later in HIV recipients (2.4 vs. 0.8 years, *p* = 0.06). New‐onset diabetes occurred in 15% of HIV and 25% of non‐HIV recipients (*p* = 0.052) and post‐LT overweight/obesity developed in 17% and 32%, respectively (*p* = 0.053). Post‐LT MASLD was diagnosed in 19.2% of recipients, with similar IRs between groups and no association with HCC recurrence.

**Conclusions**: LT in people with HIV achieves long‐term survival comparable to non‐HIV recipients, with a markedly reduced risk of rejection after 2015. HCC recurrence occurs later but not more frequently in recipients with HIV. MASLD develops in approximately one‐fifth of LT recipients, highlighting the need for integrated, multidisciplinary metabolic care after transplantation.

**FIGURE 1:**
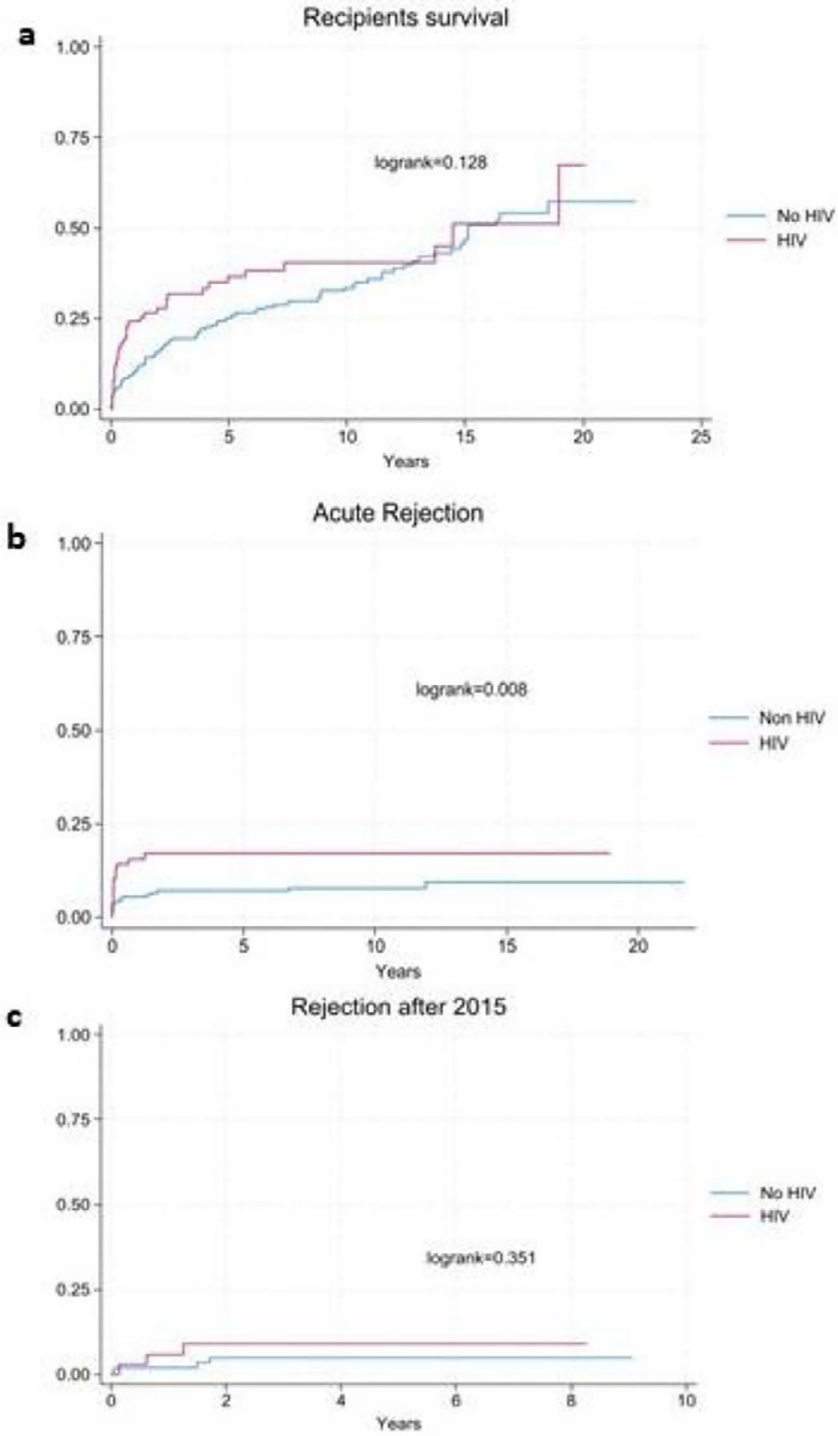
OAB0904 | Recipients’ survival (a), occurrence of graft rejection overall (b) and after 2015 (c) according to HIV serostatus.

**TABLE 1:** OAB0904 | Multivariable analysis on predictors of graft rejection in liver transplant recipients with and without HIV.

	OR (95% CI)	*p* value	aOR (95% CI)	*p* value
**Overall (from 2003 to 2024)**				
Male	0.28 (0.07−1.21)	0.088	0.33 (0.08−1.47)	0.148
HIV	2.31 (1.11−4.80)	0.025	2.43 (1.10−5.32)	0.027
CMV infection	4.08 (1.71−9.69)	0.001	3.25 (1.25−8.47)	0.016
Active HCV	2.57 (1.22−5.39)	0.012	2.40 (1.10−5.24)	0.028
**After 2015**				
HIV	2.02 (0.43−9.47)	0.372	2.15 (0.43−10.8)	0.350
CMV infection	5.47 (0.93−32.2)	0.061	5.51 (0.92−33.20)	0.062
Active HCV	1.31 (0.15−11.6)	0.808	1.37 (0.14−13.4)	0.787

## OAB0905 Lean Steatotic Liver Disease (SLD) in People Living With HIV Is Associated With Preserved Cardiometabolic Profiles but Comparable Liver Disease Severity

F. Bascombe ^1,2^, Y. Gharib^1,2^, M. Barlow^1,2^, R. Gilson^1,2^, S. L. Pett^1,2,3^, A. Arenas‐Pinto^1,2,3^

^1^University College London, Institute for Global Health, London, United Kingdom; ^2^Central and North West London NHS Foundation Trust, London, United Kingdom; ^3^University College London, MRC Clinical Trials Unit, London, United Kingdom

**Background**: Metabolic dysfunction–associated steatotic liver disease (MASLD) is increasingly recognized in people living with HIV, particularly in the context of overweight and obesity. However, steatosis can also occur in individuals with normal body mass index (BMI), and the clinical significance of “lean” MASLD remains poorly understood. We compared clinical characteristics of people with HIV with steatotic liver disease (SLD) by BMI category.

**Methods**: We conducted a cross‐sectional analysis of clinical data collected between 2019 and 2025 from a large HIV service in Central London. SLD was defined as controlled attenuation parameter (CAP) >268 dB/m. Participants were categorized as lean SLD (BMI <25 kg/m^2^) or non‐lean SLD (BMI ≥25 kg/m^2^). Demographic, cardiometabolic and HIV‐related variables were recorded alongside liver disease biomarkers and elastography‐derived risk scores and compared between groups. Logistic regression was used to identify factors associated with lean SLD.

**Results**: Among 391 people with HIV and SLD, 57 (14.6%) had lean SLD. Compared with those with BMI ≥25 kg/m^2^, lean SLD participants were older (median 58 vs. 54 years), had lower blood pressure, higher high‐density lipoprotein (HDL) cholesterol and lower CAP scores, consistent with a more favourable cardiometabolic profile. Despite this, there were no differences in markers of liver disease severity, including liver stiffness (median 5.2 vs. 5.4 kPa), FIB‐4 score or FAST score. There was no difference in alcohol consumption between groups. Lean SLD was independently associated with a history of lipodystrophy (adjusted OR (aOR) 6.27, 95% CI 1.55–25.39), higher HDL cholesterol (aOR 3.43, 95% CI 1.43–8.20) and use of tenofovir alafenamide–containing (TAF) antiretroviral therapy (ART) (aOR 3.10, 95% CI 1.45–6.61). Hepatitis B co‐infection was more common in lean SLD in univariate analyses.

**Conclusions**: In people with HIV, lean SLD appears to be associated with a more favourable cardiometabolic profile. However, liver disease burden is comparable in both lean and non‐lean SLD groups. These findings suggest that BMI‐ and metabolic risk–based screening strategies may miss a subgroup of people at risk of liver disease. The strong association with lipodystrophy raises the possibility of distinct mechanistic pathways, including altered fat distribution and mitochondrial dysfunction, warranting further investigation (see Table 1 and 2).

**TABLE 1:** OAB0905 | Clinical and liver disease characteristics of people with HIV and steatotic liver disease, by BMI category.

	BMI >25 kg/m^2^ (*n* = 334)	BMI <25 kg/m^2^ (*n* = 57)	Overall (*n* = 391)	*p* value
Age in years (median, interquartile range [IQR])	54 (47, 61)	58 (52, 63)	55 (48, 61)	0.014
History of lipodystrophy (*n*, %)	7 (2.1)	6 (10.5)	13 (3.3)	<0.001
Diastolic blood pressure (median, IQR)	80 (75, 86)	74 (69, 79)	79 (74, 85)	<0.001
Hepatitis B virus (HBV) co‐infection (*n*, %)	27 (8.1)	11 (19.3)	38 (9.7)	0.008
HDL cholesterol (median, IQR)	1.1 (0.9, 1.3)	1.2 (1.0, 1.6)	1.1 (0.9, 1.4)	0.004
CAP score in dB/m (median, IQR)	305 (286, 334)	289 (280, 312)	302 (284, 331)	0.002
Stiffness score in kPa (median, IQR)	5.4 (4.5, 6.6)	5.2 (4.3, 6.4)	5.3 (4.5, 6.6)	0.392
FAST score (median, IQR)	0.32 (0.23; 0.47)	0.25 (0.16; 0.39)	0.31 (0.22; 0.46)	0.02

**TABLE 2:** OAB0905 | Factors independently associated with lean steatotic liver disease among people with HIV.

	Multivariate		
	OR	95% CI	*p* value
History of lipodystrophy	6.27	1.55; 25.39	0.010
TAF‐containing ART	3.10	1.45; 6.61	0.003
Diastolic blood pressure	0.95	0.91; 0.98	0.005
HDL cholesterol	3.43	1.43; 8.20	0.006
CAP score in dB/m	0.99	0.97; 0.99	0.038
Alcohol consumption above recommended limits	1.19	0.69; 2.07	0.535

## OAB1402 HIV Drug Resistance Prevalence and Subtype Diversity Among African Adults Living With HIV With Adherence Challenges: Implications for the Use of Long‐Acting Cabotegravir and Rilpivirine

C. Norcross ^1,2^, G. Sanyu^2^, M. Nanyonjo^2^, B. Akatukunda^2^, P. Kafeero^3^, J. Kitonsa^1,2^, U. Bahemuka^1,2^, V. Tumusiime^2^, V. Ankunda^2^, J. Nabbuto^2^, J. F. Lunkuse^2^, E. Laker Odongpiny^4^, N. Owarwo^4^, I. Yawe^5^, L. Achieng Ombajo^6^, S. Mahomed^7^, S. Kassim^8^, N. Garrett^7,8,9^, A. Idahosa^10^, I. Eshun‐Wilsonova^10^, V. van Eygen^10^, N. Bbosa^2,3^, E. Ruzagira^2,3^, D. Grint^3^, F. V. Cresswell^1,2,3^, J. H. Vera^1^, D. Ssemwanga^2,3,11^

^1^Brighton & Sussex Medical School, Brighton, United Kingdom; ^2^MRC/UVRI & LSHTM Uganda Research Unit, Entebbe, Uganda; ^3^London School of Hygiene & Tropical Medicine, London, United Kingdom; ^4^Infectious Diseases Institute, Kampala, Uganda; ^5^Joint Clinical Research Center, Fort Portal, Uganda; ^6^University of Nairobi, Nairobi, Kenya; ^7^Centre for the AIDS Programme of Research in South Africa (CAPRISA), Durban, South Africa; ^8^Desmond Tutu Health Foundation, Cape Town, South Africa; ^9^University of Cape Town, Cape Town, South Africa; ^10^Johnson & Johnson Innovative Medicine, Beerse, Belgium; ^11^Uganda Virus Research Institute, Entebbe, Uganda

**Background**: Individuals with a recent history of suboptimal adherence to oral antiretroviral therapy may benefit from long‐acting injectable regimens. However, virological risk factors for subsequent long‐acting treatment failure such as rilpivirine resistance‐associated mutations (RAMs) and HIV subtype A6 are a concern. Understanding their prevalence and distribution in the African programmatic care setting is key to facilitating safe and effective rollout.

**Methods**: HIV pro‐viral DNA was extracted from baseline peripheral blood mononuclear cells of IMPALA (NCT05546242) participants, a trial investigating long‐acting cabotegravir/rilpivirine efficacy in Africa. Next‐generation sequencing was performed on the Illumina MiSeq platform following amplification of the full HIV‐1 *pol* gene (*protease*, *reverse transcriptase* and *integrase* regions) using the DeepChek Assay kit. Sequencing data were analysed using the HyDRA Web pipeline. HIV drug resistance and subtype classification were determined using the Stanford HIV Drug Resistance database, REGA v3.0 and COMET. Logistic regression was conducted to identify factors associated with resistance.

**Results**: High‐quality sequencing data in at least one region was obtained for 65.2% (341/523) of available samples. Median age was 40.0 years [IQR 34.0−49.0], all were receiving dolutegravir‐based therapy, 78.6% (268/341) had previously received non‐nucleoside reverse transcriptase (NNRTI)‐based therapy, median time since HIV diagnosis was 8.7 years [IQR 4.9−13.7] and 82.7% (282/341) had undetectable viral load. Subtype A1 predominated (50.4%; 172/341), followed by recombinant forms (19.4%; 66/341), C (18.2%; 62/341), D (11.7%; 40/341) and G (0.3%; 1/341). Subtype A6 was not observed. At the 20% detection threshold, prevalence of NNRTI resistance and rilpivirine RAMs was 14.7% (46/313) and 5.8% (18/313), respectively. For major integrase strand transfer inhibitor (INSTI) resistance and cabotegravir RAMs, this was 0.3% (1/339). At the 1% detection threshold, NNRTI resistance prevalence was 29.4% (92/313), rilpivirine RAMs 14.7% (46/313), and major INSTI and cabotegravir RAMs 18.0% (61/339). No demographic or clinical factors were associated with baseline resistance.

**Conclusions**: Baseline prevalence of established virological risk factors for long‐acting cabotegravir/rilpivirine failure was consistent with surveillance data, supporting the use of this regimen in this population. However, inclusion of minority variants substantially increased observed prevalence of both NNRTI and INSTI resistance. Clinical significance of low‐frequency resistance mutations for long‐acting injectable regimens remains unclear, underscoring the need for careful post‐implementation outcome monitoring.

## OAB1403 Efficacy and Safety of Twice‐Yearly Lenacapavir, Teropavimab and Zinlirvimab as an HIV‐1 Treatment for up to 104 Weeks

J. McMahon ^1^, J. Eron^2^, A. Gaur^3^, E. DeJesus^4^, D. A. Baker^5^, C. Brinson^6^, P. Cook^7^, G. Crofoot^8^, J. Gallant^9^, M. S. McKellar^10^, H. Liu^11^, N. Zhang^11^, K. Mponponsuo^11^, S. E. Collins^11^, O. Ogbuagu^12^

^1^The Alfred Hospital and Monash University, Melbourne, Australia; ^2^University of North Carolina, Chapel Hill, United States; ^3^St. Jude Children's Research Hospital, Memphis, United States; ^4^Orlando Immunology Center, Orlando, United States; ^5^East Sydney Doctors Darlinghurst, New South Wales, Australia; ^6^Central Texas Clinical Research, Austin, United States; ^7^East Carolina University, Division of Infectious Diseases, Greenville, United States; ^8^The Crofoot Research Center, Houston, United States; ^9^AXCES Research, Santa Fe, United States; ^10^Duke University School of Medicine, Division of Infectious Diseases, Department of Medicine, Durham, United States; ^11^Gilead Sciences, Inc., Foster City, United States; ^12^Yale School of Medicine, New Haven, United States

**Background**: The broadly neutralizing antibodies (bNAbs) teropavimab (TAB) and zinlirvimab (ZAB) are under investigation as a twice‐yearly HIV‐1 treatment with lenacapavir (LEN). In a Phase 2 randomized study (NCT05729568), LEN+TAB+ZAB maintained HIV‐1 RNA <50 copies/mL in 47/53 (89%) participants at Week (W)52 (FDA Snapshot Algorithm). We report W104 efficacy and safety.

**Methods**: Virologically suppressed (VS) people with HIV‐1 susceptible to both bNAbs were randomized 2:1 to twice‐yearly subcutaneous LEN 927 mg (+oral LEN 600 mg, Days 1+2) with intravenous TAB 2550 mg and ZAB 2550 mg, or continue their oral stable baseline regimen (SBR) through W52. At W52, VS participants could continue LEN+TAB+ZAB or switch from SBR–>LEN+TAB+ZAB in the extension phase (EP). Endpoints included HIV‐1 RNA <50 copies/mL (missing = excluded while on study treatment [M = E]), CD4 cell count change from baseline and adverse events (AEs) through W104 for participants receiving LEN+TAB+ZAB (104 weeks of LEN+TAB+ZAB for those randomized to LEN+TAB+ZAB and 52 weeks for SBR–>LEN+TAB+ZAB).

**Results**: Eighty participants enrolled: 47/53 participants randomized to LEN+TAB+ZAB continued in the EP; 24/27 SBR participants switched to LEN+TAB+ZAB at W52 and 23 were dosed in the EP. Prior to first LEN+TAB+ZAB dose in the LEN+TAB+ZAB and SBR–>LEN+TAB+ZAB groups, respectively, median (range) age: 46 (20–65) and 56 (30–65) years; mean (SD) BMI: 30.9 (7.4) and 29.4 (7.1) kg/m^2^; median (interquartile range) CD4 count: 710/µL (552–895) and 734/µL (595–941). At W104, 98% (40/41) of LEN+TAB+ZAB only participants had HIV‐1 RNA <50 copies/mL; 100% (19/19) of SBR–>LEN+TAB+ZAB participants had HIV‐1 RNA <50 copies/mL after 52 weeks of LEN+TAB+ZAB (M = E) (Table 1). During the EP, one participant in each group had confirmed virologic rebound and discontinued treatment. Median (interquartile range) changes in CD4 cell counts at W104 while on LEN+TAB+ZAB: LEN+TAB+ZAB, 34/µL (–71–111); SBR–>LEN+TAB+ZAB, –38/µL (–159–40). The most common treatment‐emergent AEs were subcutaneous LEN‐related injection site reactions, leading to treatment discontinuation in two participants. Infusion‐related reactions occurred in one participant (Grades 1 and 2). No participant receiving LEN+TAB+ZAB experienced a treatment‐related serious AE (Table 2).

**Conclusions**: Twice‐yearly LEN+TAB+ZAB maintained high rates of virologic suppression for up to 2 years and demonstrated a favourable safety profile.

**TABLE 1:** OAB1403 | Virologic outcomes at week 104.

	LEN+TAB+ZAB (received LEN+TAB+ZAB for 104 weeks)	SBR–>LEN+TAB+ZAB (received LEN+TAB+ZAB for 52 weeks)
Participants who received the complete study regimen in the extension phase, *n*	47^a^	23^b^
**Missing** = **excluded while on treatment analysis**		
HIV‐1 RNA <50 copies/mL, *n*/*N* (%) [95% CI]^c^	40/41^d^ (98) [87; 100]	19/19^e^ (100) [82; 100]
HIV‐1 RNA ≥50 copies/mL, *n*/*N* (%) [95% CI]^c^	1/41 (2) [0; 13]	0 [0; 18]

^a^Four of the 53 LEN+TAB+ZAB participants discontinued study treatment during the randomized phase, and two participants completed the randomized phase and did not enter into the extension phase.

^b^Two of the 27 SBR participants discontinued the study treatment during the randomized phase, and one participant completed the randomized phase and did not enter into the extension phase. Twenty‐four SBR–>LEN+TAB+ZAB participants entered the extension phase; one participant did not receive the complete study regimen (oral LEN and one of the two subcutaneous LEN doses) and was excluded from efficacy analyses.

^c^The 95% CI for percentage estimate was calculated based on the Clopper−Pearson exact method.

^d^Of the 47 participants in the LEN+TAB+ZAB group, seven discontinued study treatment during the extension phase: participant decision (*n* = 5), death (*n* = 1 [cardiac arrest unrelated to study treatment]) and lack of efficacy (*n* = 1). Two of the seven discontinued after Week 104 with HIV‐1 RNA data at Week 104: the participant who discontinued due to lack of efficacy at Week 104 is included in the Table “HIV‐1 RNA ≥50 copies/mL” category; one participant who discontinued due to participant decision at Week 104 is included in the Table “HIV‐1 RNA <50 copies/mL” category. One participant remained on treatment and was missing data at Week 104.

^e^Of the 23 participants in the SBR–>LEN+TAB+ZAB group, two discontinued study treatment during the extension phase: adverse event (*n* = 1) and investigator's discretion (*n* = 1) met criteria for confirmed virologic rebound. Two participants remained on treatment and were missing data at Week 104.

Abbreviations: CI, confidence interval; LEN, lenacapavir; SBR, stable baseline regimen; TAB, teropavimab; ZAB, zinlirvimab.

**TABLE 2:** OAB1403 | Safety^a^ at week 104.

	LEN+TAB+ZAB (received LEN+TAB+ZAB for 104 weeks)	SBR–>LEN+TAB+ZAB (received LEN+TAB+ZAB for 52 weeks)
Participants exposed to LEN, TAB or ZAB throughout the study, *n*	53	24^b^
Adverse events (excluding injection site reactions), *n* (%)	49 (93)	17 (71)
Grade ≥3	7 (13)	0
Adverse events (including injection site reactions), *n* (%)	52 (98)	24 (100)
Grade ≥3	7 (13)	1 (4)
Treatment‐related adverse events (excluding injection site reactions), *n* (%)	8 (15)	3 (13)
Grade ≥3	0	0
Treatment‐related adverse events (including injection site reactions), *n* (%)	39 (74)	20 (83)
Grade ≥3	0	1 (4)
Subcutaneous LEN‐related injection site reactions, *n* (%)	37 (70)	20 (83)
Grade ≥3	0	1 (4)^c^
Adverse events leading to premature discontinuation of study treatment, *n* (%)	0	2 (8)
Grade ≥3	0	1 (4)^c^
Serious adverse events, *n* (%)	5 (9)	0
Treatment‐related	0	0
Deaths, *n*	1 (2)^d^	0
Treatment‐related	0	0

^a^Safety is inclusive of all time with exposure to LEN+TAB+ZAB at the date of the data cut.

^b^Twenty‐four SBR–>LEN+TAB+ZAB participants entered the extension phase; one participant received oral LEN and one of the two subcutaneous LEN doses and is included in safety analyses.

^c^Grade 4 injection site reaction (necrosis) occurred, led to study treatment discontinuation.

^d^Cardiac arrest unrelated to study treatment (methamphetamine/amphetamine intoxication).

Abbreviations: LEN, lenacapavir; SBR, stable baseline regimen; TAB, teropavimab; ZAB, zinlirvimab.

## OAB1404 A Rapid and Inexpensive Phenotypic Assay for Measuring Resistance to Existing and Emerging HIV Drugs

D. K. Mims ^1^, M. M. Chang^1^, A. O. Olanrewaju^1^

^1^University of Washington, Bioengineering, Seattle, United States

**Background**: Rapid and inexpensive HIV drug resistance testing (DRT) would enable pre‐treatment screening, real‐time evaluation after virologic failure, and efficacy assessment of new and existing drugs during clinical trials. However, current DRT is complex and expensive, limiting routine use. Genotypic DRT is challenging to interpret because it cannot directly associate detected mutations with losses in antiretroviral susceptibility. Phenotypic DRT is simpler to interpret but typically requires time‐and labour‐intensive cell culture; phenotypic assays that instead measure the activity of isolated HIV enzymes are faster and simpler with potential for widespread use outside centralized labs. Here, we present an optimized product enhanced reverse transcriptase (PERT) assay that measures phenotypic susceptibility to HIV‐reverse transcriptase (HIV‐RT) inhibitors.

**Methods**: PERT combines (1) complementary DNA (cDNA) synthesis depending on HIV‐RT quantity and susceptibility to included inhibitors and (2) amplification and detection of cDNA products by quantitative PCR (qPCR). We quantified viral load by converting the qPCR threshold cycle (Ct) in the no‐drug condition to corresponding HIV RNA copies (assuming 80 copies HIV‐RT/virion, 2 copies RNA/virion). We quantified resistance using the ΔCt between drug and no‐drug conditions, normalized to wildtype sample at the maximum drug concentration, and used one‐way ANOVA and post‐hoc Dunnett's test to compare inhibition of mutant and wildtype HIV‐RT.

**Results**: The optimized assay shows linear quantification from 10 to 1E6 HIV‐RT copies, corresponding to a viral load ∼25−2.5E6 (Figure 1A). We measured inhibition of HIV‐RT activity (viral load ∼2500) by the nucleoside RT inhibitor lamivudine‐5’‐triphosphate (3TC‐TP), nucleoside RT translocation inhibitor MK‐8591‐TP, and non‐nucleoside RT inhibitors doravirine and rilpivirine. When combining wildtype and M184V mutant HIV‐RT in heterogenous mixtures, we detected 10% and 60% resistant fraction using 3TC‐TP and MK‐8591‐TP, characterized by 33.8% and 31.6% decrease in inhibition relative to wildtype (*p* = 0.0005 and *p* = 0.0289), respectively (Figure 1B).

**Conclusions**: This PERT assay meets WHO analytical sensitivity requirements for DRT. With its high sensitivity, short duration (∼2 h), compatibility with existing and emerging RT inhibitors, and ability to leverage existing instruments in high‐ and low‐resource settings, this enzymatic assay has the potential to significantly improve access to routine HIV DRT in point‐of‐care and clinical trial settings.

**FIGURE 1:**
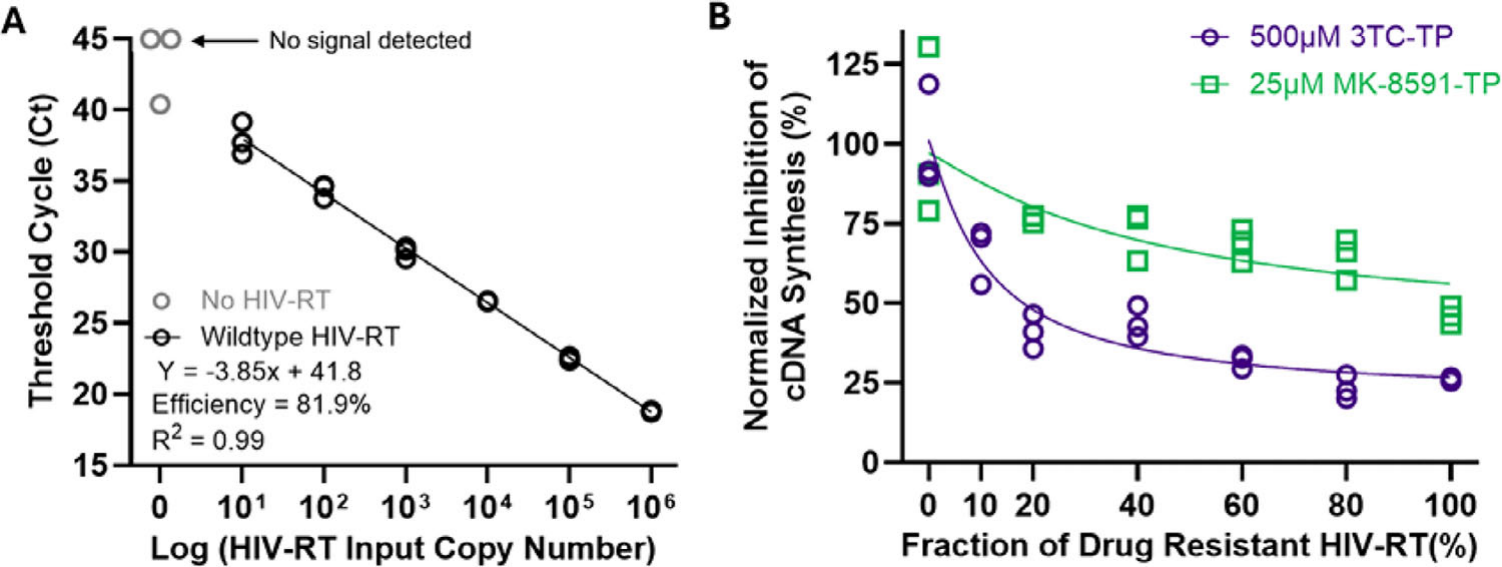
OAB1404

## OAB2002 Assessing Implementation and Expansion of Advanced HIV Disease in Malawi

J. Fly Phiri ^1^, T. Chimpandule^1,2^, A. Jahn^1,3^, R. Malunga^1^, G. Gomani^1^, F. Kalonga^1^, L. Chewere^1^, B. A. Tippett Barr^2^, S. Macheso^1^

^1^Ministry of Health and Sanitation, Department of HIV, STI's and Viral Hepatitis, Lilongwe, Malawi; ^2^Ministry of Health and Sanitation, Nyanja Health Research Institute, Lilongwe, Malawi; ^3^Clinton Health Access Initiative, Lilongwe, Malawi

**Background**: Despite remarkable progress with ART scale‐up, an estimated 60,000 (6%) of all PLHIV in Malawi were estimated to live with advanced HIV disease (AHD) in 2023. The Ministry of Health and Sanitation aimed to decentralize AHD screening and introduced dedicated AHD services in 2024 for all new and re‐initiating ART clients. We assessed implementation and evaluated gaps in diagnostics and treatment.

**Methods**: We used routine facility‐level programme data from July 2024 through September 2025 to evaluate AHD screening availability and results. We used the national HIV estimates (2025) to compare the proportion meeting AHD criteria to the national AHD data.

**Results**: Of the 76,415 total eligible clients (first‐time and reinitiation of ART), 81.8% (*n* = 62,539) were screened for AHD, of whom 58.7% (*n* = 36,690) were females, and 94.4% (*n* = 58,786) were age 15 or above. Ninety‐five percent (*n* = 59,512) of clients were examined clinically, 21.0% of whom (*n* = 12,517) were in WHO Stage in 3 or 4. About half (51.9%, *n* = 32,428) of clients screened for AHD had CD4 counts, 29.3% of whom (*n* = 9486) were <200 cells/mm^3^; 6.1% (*n* = 1974) CD4 results were missing or unknown. In total, 18,405 (29.4%) of all clients screened were diagnosed with AHD based on age, clinical staging and/or CD4 count; 39% of the modelled estimate of 47,334 people with AHD and on ART during this period in Malawi. Three quarters of clients diagnosed with AHD were tested for serum cryptococcaemia (75.2%, *n* = 13,841), and 6.2% (*n* = 860) of these were positive; 74.7% (*n* = 13,746) of AHD clients were screened for disseminated tuberculosis using LAM test and 14.1% (*n* = 1966) were positive. Eleven percent (11.5%, *n* = 2112) of AHD clients received GeneXpert testing for tuberculosis, of whom one quarter (25.6%, *n* = 541) were positive, and 12.7% (*n* = 2330) of the total AHD clients had chest x‐rays done for tuberculosis, of which 42.1% (*n* = 981) had abnormal findings.

**Conclusions**: Almost half of the estimated AHD cases among clients on ART were found among new and re‐initiating ART clients. Programme data reveal gaps that may be closed with further decentralization of CD4 count screening and consistent availability of diagnostics for opportunistic pathogens or microbes.

## OAB2003 Prevalence and Risk Factors Associated With Advanced HIV Disease Among People Living With HIV in the Philippines, 2019−2024

J. Bacha^1^, F. Smith^1^, A. Zamora^2^, E. Agyemang^1^, M. Tejan^1^, R. Lacson^2^, J. Yang^3^, R. Haynes ^3^, N. G. Saplagio^3^, N. Palaypayon^3^, N. Montevirgen^4^, E. Dano^4^, M. E. Evans^2^

^1^U.S. Centers for Disease Control and Prevention, Atlanta, United States; ^2^U.S. Centers for Disease Control and Prevention, Manila, the Philippines; ^3^Philippines Department of Health, Epidemiology Bureau, Manila, the Philippines; ^4^ICAP, Columbia University, Manila, the Philippines

**Background**: The Philippines has the fastest growing HIV epidemic in the Asia‐Pacific Region. Understanding the burden of advanced HIV disease (AHD) and its associated clinical factors is critically important for optimizing the HIV response and developing an AHD package of care in the Philippines.

**Methods**: A retrospective cohort analysis was conducted using routinely collected clinical data from the national HIV electronic medical record system of people living with HIV (PLHIV) receiving care in the Philippines between 2019 and 2024. AHD was defined as CD4<200 cells/mm^3^ among PLHIV ages ≥5 years old. Only PLHIV with CD4 results were included in the analysis. Tests of differences by AHD status were conducted using chi square tests for clinical categorical variables and Mann−Whitney−Wilcoxon tests for continuous variables. Multivariable logistic regression was used to determine the adjusted odds ratios (aOR) of AHD and for clinical outcomes of the AHD and non‐AHD.

**Results**: Among 52,904 PLHIV with CD4 data, 46.7% (24,801/52,904) had at least one CD4<200 result. The total number of PLHIV with CD4 testing increased by 145% from 2019 (8962 PLHIV) to 2024 (21,970 PLHIV), with AHD rates ranging from 48.5% to 56.4% (Figure 1). Among those with CD4<200, 89.1% were ART‐naïve and 10.9% treatment‐experienced. Factors associated with increased odds for AHD included older age (aOR = 1.93, >25 vs. 15−24‐year‐old), marriage (aOR = 1.25) and living in high HIV burden regions (aOR = 1.17). Decreased odds of AHD were associated with higher education (aOR = 0.85), >12 months on ART (aOR = 0.79), women (aOR = 0.78) and being on a dolutegravir‐based regimen (DBR, aOR = 0.59) (Figure 2). Compared to those with CD4≥200, those with CD4<200 had higher rates of tuberculosis (TB, 83.1% vs. 16.9%, *p* < 0.001) and other opportunistic infections (OIs, 74.9% vs. 25.1%, *p* < 0.001) and lower DBR use (42.1% vs. 57.9%, *p* < 0.001).

**Conclusions**: High rates of AHD were observed in the Philippines with the AHD cohort displaying clinical and treatment characteristics such as high rates of TB, OIs and non‐DBR use. Recognition of AHD through CD4 testing—especially for those at higher risk for AHD—and providing DBRs as part of the AHD package of care are critical steps to support those with AHD in the Philippines.

**FIGURE 1:**
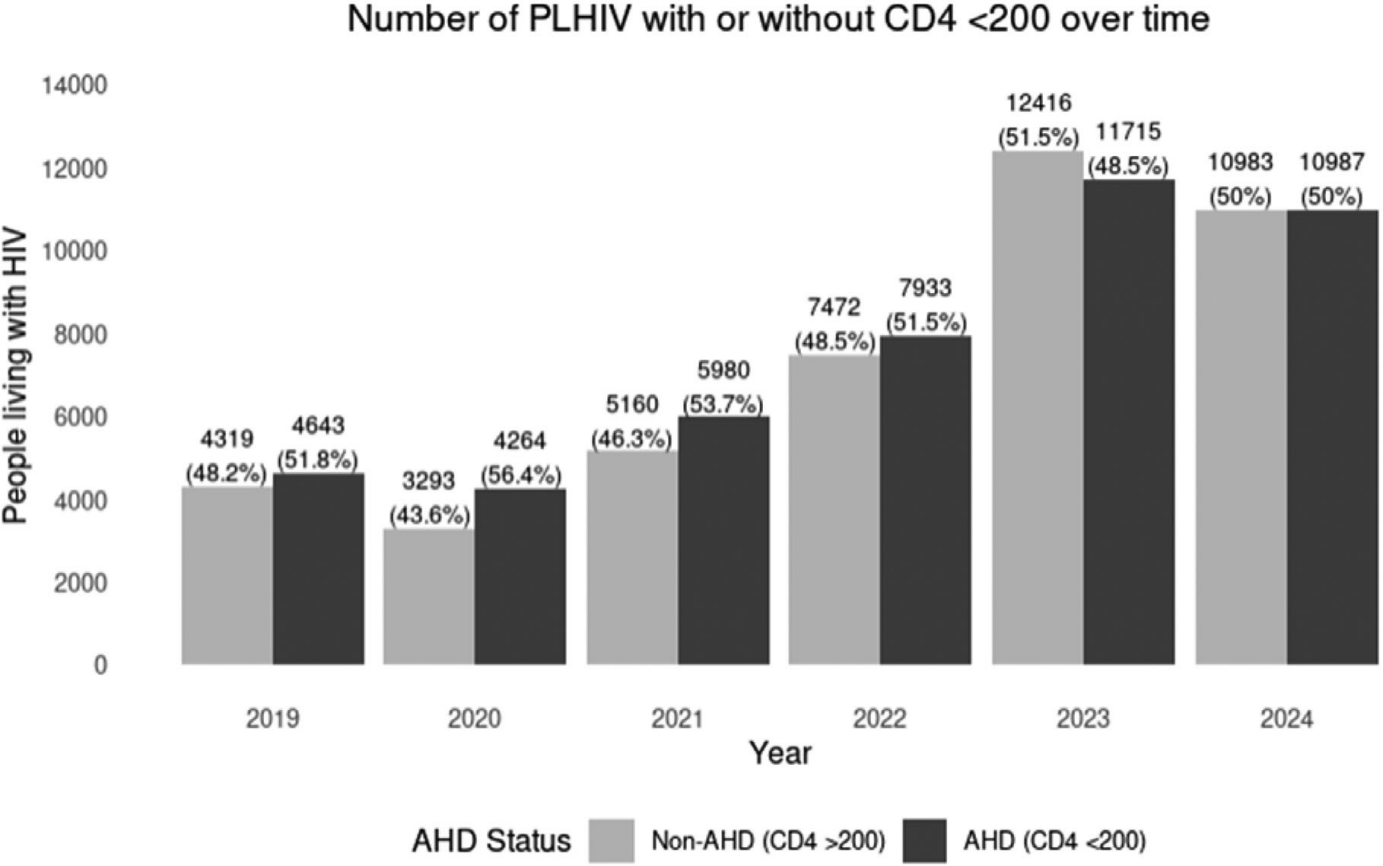
OAB2003

**FIGURE 2:**
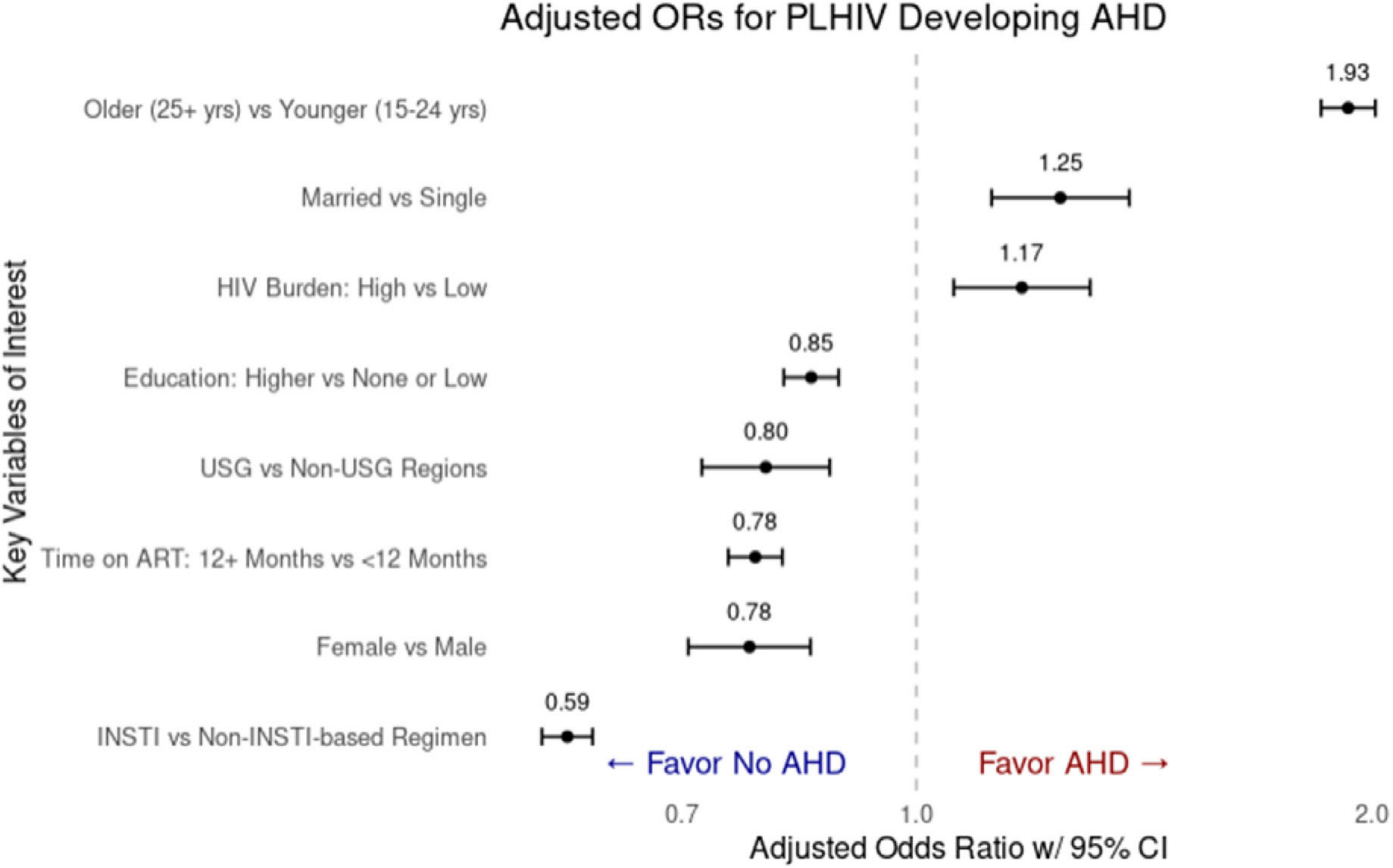
OAB2003

## OAB2004 Advanced HIV Disease and Subsequent Mortality and Hospitalization Among People Living With HIV in South Africa

S. S. Mbeje ^1^, X. Masombuka^2^, C. Pastellides^2^, C. Suttner^2^, J. A. Brown^3^, J. S. van der Molen^4^, S. Mahomed^4^, N. Garrett^5^, L. Lewis^1^, L. Wayburne^2^, J. Dorward^6,7^

^1^Centre for the AIDS Programme of Research in South Africa (CAPRISA), University of KwaZulu‐Natal, Statistics, Durban, South Africa; ^2^Discovery Health, Health Intelligence, Sandton, South Africa; ^3^Centre for the AIDS Programme of Research in South Africa (CAPRISA), University of KwaZulu‐Natal, Durban, South Africa; ^4^Centre for the AIDS Programme of Research in South Africa (CAPRISA), University of KwaZulu‐Natal, Data Management, Durban, South Africa; ^5^University of Cape Town, Desmond Tutu HIV Centre, Cape Town, South Africa; ^6^Centre for the AIDS Programme of Research in South Africa (CAPRISA), University of KwaZulu–Natal, Durban, South Africa; ^7^Nuffield Department of Primary Care Health Sciences, University of Oxford, Oxford, United Kingdom

**Background**: Advanced HIV disease (AHD; defined as CD4 <200 cells/µL or WHO Stage 3/4 disease) remains significant among people living with HIV (PLHIV) initiating antiretroviral therapy (ART) in low‐ and middle‐income countries. However, estimates of incidence and causes of subsequent mortality and hospitalization in these settings are scarce, largely due to a lack of linked primary and secondary care data. This study leverages comprehensive South African private healthcare sector data to characterize the burden of AHD and its impact on hospitalization and survival.

**Methods**: We conducted a retrospective cohort study using de‐identified claims and laboratory data from Discovery Health, a managed healthcare organization in South Africa. We determined AHD prevalence among PLHIV aged ≥15 years initiating ART from 2018 to 2024. We used multivariable Cox regression with time‐varying effect for baseline AHD status and log‐normal accelerated failure time models to estimate the effect of AHD on mortality and non‐pregnancy‐related hospitalization rates.

**Results**: Among 17,767 PLHIV, 58.2% were females, with a median age of 37 years (IQR: 32−44), 3% died and 33.1% were hospitalized. Annual ART initiation AHD prevalence ranged from 21.3% to 25.4%. Median follow‐up was 3.67 years (IQR: 1.62, 6.16), with overall incidence of death of 7.8 and hospitalization of 115.5/1000 person‐years. In the first 6 months, individuals with AHD were 15.92 times more likely to die compared to those without (aHR 15.92; 95% CI: 8.12−31.2). While the risk decreased over time, it remained elevated after 24 months (aHR 2.09; 95% CI: 1.35−3.25). The leading causes of death were tuberculosis (*n* = 55) and unspecified HIV‐related mortality among those with and without AHD, respectively. People with AHD had 81% shorter time to first hospital admission compared to those without AHD (TR 0.19; 95% CI: 0.16−0.22). Leading causes of hospitalization were pneumonia (*n* = 111), gastroenteritis (*n* = 37) and sepsis (*n* = 34) among those with AHD, and depression (*n* = 71), gastroenteritis (*n* = 21) and abnormal vaginal bleeding (*n* = 20) in those without AHD.

**Conclusions**: AHD prevalence at ART initiation remains high in South Africa and significantly increases the risk of subsequent mortality and hospitalization, underscoring the high clinical and economic burden of AHD (see Table 1 and Figure 1).

**TABLE 1:** OAB2004 | Cox regression model, with time‐varying effect of AHD status, of time to death.

Variable	Estimate (standard error)	Adjusted hazard ratio (95% confidence interval)	*p*‐value
**AHD status**			
AHD versus no AHD (0–6 months)	2.7677 (0.3433)	15.92 (8.12–31.2)	< 0.001
AHD versus no AHD (6−12 months)	1.5532 (0.3883)	4.73 (2.21–10.12)	< 0.001
AHD versus no AHD (18−24 months)	1.9821 (0.5335)	7.26 (2.55–20.65)	< 0.001
AHD versus no AHD (>24 months)	2.208 (0.6087)	9.10 (2.76–29.99)	< 0.001
Age (per year increase)	0.7394 (0.2238)	2.09 (1.35–3.25)	< 0.001
Sex (male vs. female)	0.0162 (0.0043)	1.02 (1.01–1.02)	< 0.001
	−0.0993 (0.0897)	0.91 (0.76–1.08)	0.268

**FIGURE 1:**
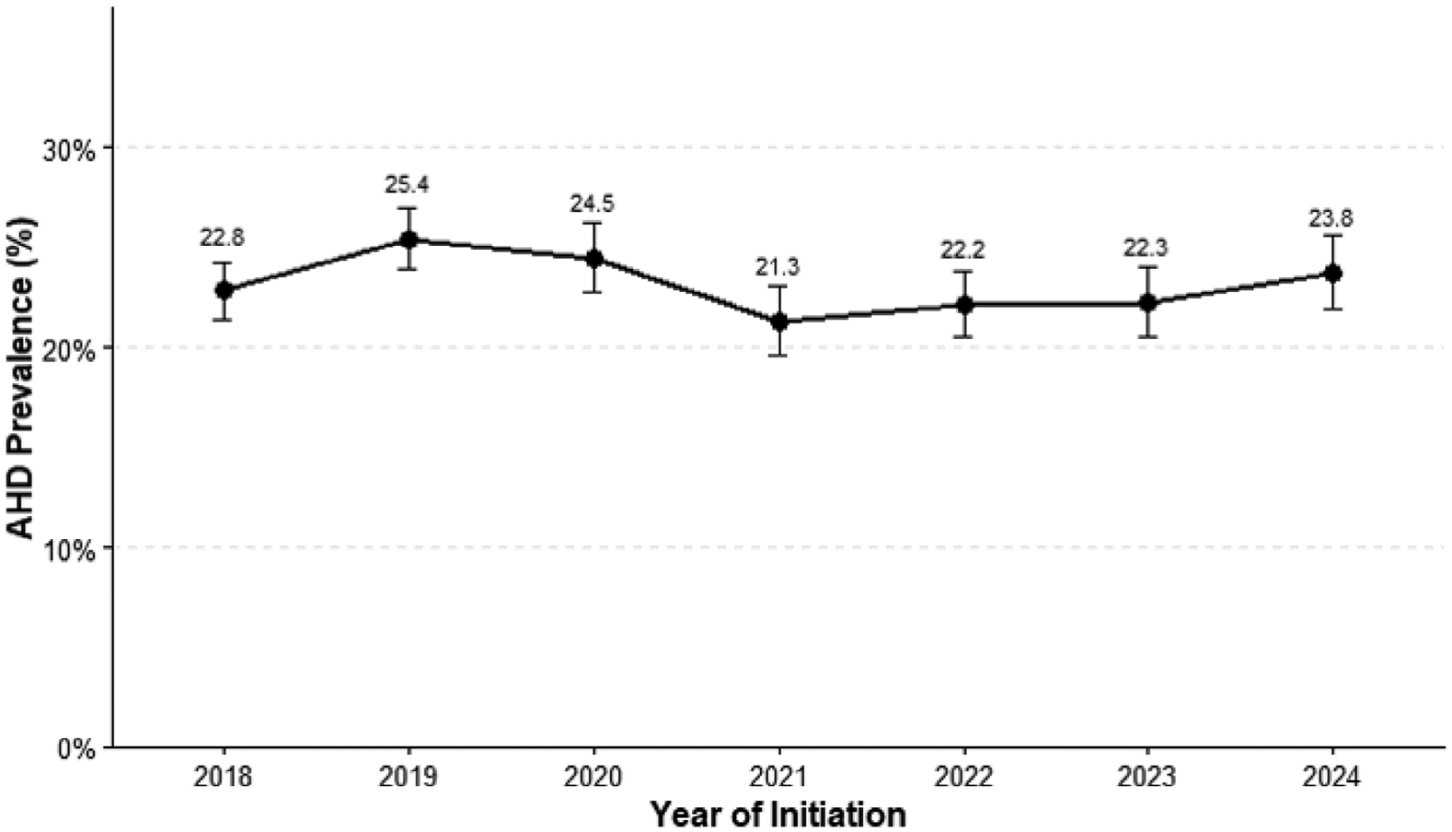
OAB2004 | Prevalence of advanced HIV disease (AHD) at ART initiation.

## OAB2005 Impact of Late Diagnosis on HIV Mortality in Cuba 2019−2025

M. Duque Vizcaino ^1^, L. Vazquez Bello^2^, M. Romero Placeres^3^, N. Ramos Iglesias^3^, N. Castillo Hernandez^4^, O. Sued^5^

^1^Pedro Kouri Tropical Medicine Institute, Hospital, Havana, Cuba; ^2^Pedro Kouri Tropical Medicine Institute, General Direction, Havana, Cuba; ^3^Cuban Ministery of Public Health, Epidemiology, Havana, Cuba; ^4^Cuban Ministery of Public Health, Epidemiology, Havana, Cuba; ^5^PAHO, Washington, United States

**Background**: Despite a decline in AIDS‐related deaths, late diagnosis of HIV, defined as a CD4 of <350 cells/mm^3^, affects individuals and society by increasing the risk of HIV‐associated morbidity and mortality. Latin America is one of the regions with an increase in the new HIV cases compared with 2010 figures. Cuba is one of the countries with the lowest HIV incidence and mortality. Still, late diagnosis continues to be significant. The aim is to describe the clinical characteristics of people with late diagnosis and its impact on HIV‐related death in Cuba from 2019 to 2025.

**Methods**: A nationwide, cross‐sectional study was conducted with a total of newly diagnosed HIV cases and HIV‐related deaths in Cuba between 2019 and 2025. The Medisys‐VIH national computerized registry was used for data collection. CD4 count was determined by flow cytometry, and late diagnosis was defined as a CD4 count below 350 cells/mL at the time of HIV diagnosis. Regarding mortality, the relationship between late HIV diagnosis and HIV‐related death within 1 year of diagnosis was studied. Relative and absolute frequency analyses were performed in the selected population groups. All personal data was protected according to national and international standards.

**Results**: During the study period, 13,516 and 2975 deaths were recorded, without significant annual changes. Epidemic is concentrated in males (78%), and young adults individuals (mean 35.7 years), 53% were men who have sex with men. In 2019, CD4 counts were performed on 54.3% of new HIV diagnoses, with a reduction in the 2021−2022 period (3.2%) and a gradual recovery by 2025 (18.8%). Overall, in Cuba, there has been a reduction in late HIV diagnosis from 52.9% in 2019 to 29.7% in 2025, and in HIV‐related mortality from 69.9% to 41% during the same period. However, HIV mortality within 1 year of diagnosis has remained at an average of 34% from 2019 to 2025, with 25% of these deaths occurring in late diagnosis.

**Conclusions**: Late HIV diagnosis contributes to HIV‐related death within a year of diagnosis in the Cuban population; therefore, the implementation of early diagnosis strategies can contribute to reducing mortality and achieving national programmatic goals.

## OAB2302 Biological and Psychosocial Factors in Cognitive Impairment Among Older Adults With HIV: A Cross‐Sectional Evaluation From ELEA‐Brasil Study

P. Corção ^1^, T. Silva Torres^1^, P. Ranadive^2^, B. E. Shepherd^2^, S. Wagner Cardoso^1^, R. C Moreira^1^, V. Veloso^1^, T. Cordeiro^3^, E. Luz^3^, C. Brites^3^, R. Schiavon Nogueira^4^, J. Valdez Ramalho Madruga^4^, C. de Albuquerque Moraes^4^, M. Bordignon Antonio^5^, L. Quintanilha^5^, L. Hypolito^5^, J. L. Castilho^6^, B. Grinsztejn^1^, on behalf of ELEA‐Brasil

^1^Fundação Oswaldo Cruz (FIOCRUZ), INI, Rio de Janeiro, Brazil; ^2^Vanderbilt University Medical Center, Department of Biostatistics, Nashville, United States; ^3^Hospital Universitário Professor Edgard Santos (HUPES), Salvador, Brazil; ^4^Centro de Referência e Treinamento DST/Aids (CRT), São Paulo, Brazil; ^5^Hospital das Clínicas da Faculdade de Medicina da Universidade de São Paulo (USP), São Paulo, Brazil; ^6^Vanderbilt University Medical Center, Division of Infectious Diseases, Nashville, United States

**Background**: Cognitive impairment is common in older persons living with HIV (PLWH) despite widespread viral suppression. Knowledge regarding biological factors and the related factors underlying cognitive impairment in middle‐income countries remains incomplete. We investigated biological, psychosocial and treatment‐related factors associated with cognition impairment in a Brazilian cohort of older PLWH.

**Methods**: Cross‐sectional study, using baseline data from the Longitudinal Study of HIV & Aging in Brazil (ELEA‐Brasil). PLWH aged ≥50 years on antiretroviral therapy and viral suppression were recruited from four Brazilian clinics (from 2022 to 2023). All participants underwent comprehensive geriatric assessment that included cognitive performance (Montreal Cognitive Assessment, MoCA, poor cognitive performance defined ≤ 23) and depressive symptoms (Patient Health Questionnaire‐9, PHQ‐9, higher depressive symptoms defined ≥15), collection of sociodemographic and medical history. Participants with dementia, chronic neurologic disease and visual impairment were excluded from this analysis. With MoCA score as primary outcome, we used unadjusted and multivariable cumulative probability regression models with restricted cubic splines for continuous covariates to examine social, psychological and clinical factors.

**Results**: Of 624 participants included, 50.5% had poor cognitive performance. In a multivariable model, older age, female at birth, Black/*Pardo* race, less education, higher PHQ‐9 scores, lower nadir CD4 and longer cumulative efavirenz (EFV) exposure were each independently associated with worse cognitive performance (Figure 1). Dolutegravir (DTG) exposure, number of comorbidities, history of opportunistic infections and years since HIV diagnosis were not independently associated with cognitive impairment.

**Conclusions**: Among older Brazilian PLWH, cognitive impairment was highly prevalent and was associated with social vulnerability (race, education), depression and lower CD4 nadir. Individuals with previous prolonged efavirenz exposure were less likely to have higher cognition, even after adjusting for confounders. Routine cognitive screening and integrated mental‐health and socially responsive care should be prioritized for vulnerable persons to promote healthy ageing with HIV.

**FIGURE 1:**
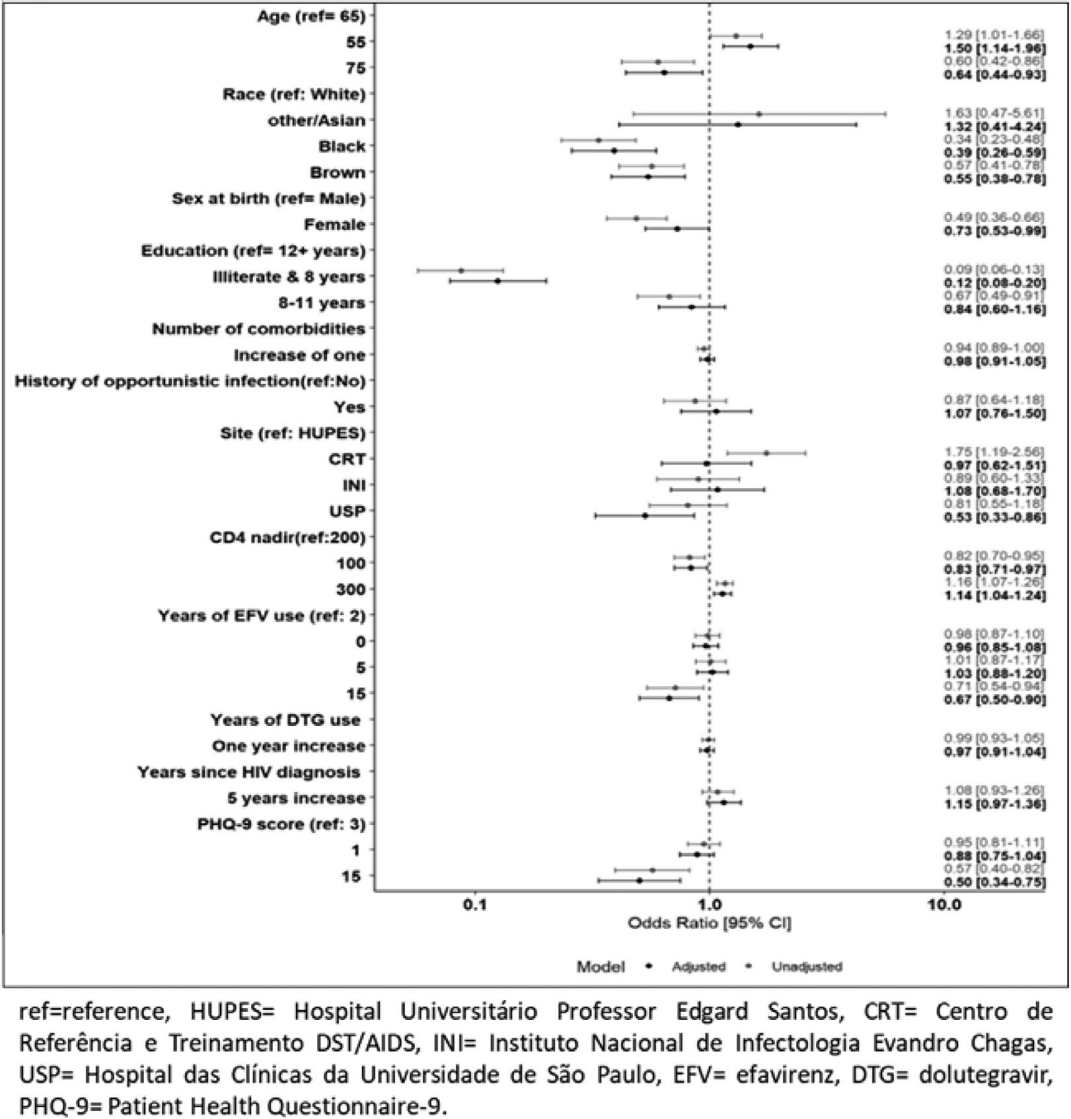
OAB2302 | Unadjusted and adjusted odds ratios (95% confidence intervals [CI]) for higher MoCA scores.

## OAB2303 Do Muscle Quantity and Muscle Quality Differ in Their Associations With Strength and Physical Performance in Ageing People With HIV? A Multimodal Imaging Study

A. dos Santos ^1,2,3^, G. Kulik^4^, M. Wilson^4^, C. Jankowski^5^, V. Khuu^4^, P. Cook^4^, D. Ghosh^6^, A. Webel^7^, V. Oliveira^7^, K. Erlandson^4^

^1^National Council for Scientific and Technological Development, Brasilia, Brazil; ^2^Sustained Training in Aging & HIV Research, University of California, San Diego, United States; ^3^University of Sao Paulo, Ribeirao Preto, Brazil; ^4^Department of Medicine, University of Colorado Anschutz, Aurora, United States; ^5^College of Nursing, University of Colorado Anschutz, Aurora, United States; ^6^Department of Biostatistics and Informatics, University of Colorado Anschutz, Aurora, United States; ^7^School of Nursing, University of Washington, Seattle, United States

**Background**: People with HIV (PWH) experience accelerated muscle loss, termed sarcopenia, which contributes to impaired physical function. Sarcopenia frameworks prioritize low muscle strength and reduced muscle mass for diagnosis; however, these approaches do not assess muscle quality, measured by fatty infiltration, relevant to functional decline. This study compared associations between imaging–derived muscle quantity and quality with muscle function in PWH.

**Methods**: This cross‐sectional analysis used baseline data from the HEALTH Study (NCT 04550676). PWH underwent dual‐energy X‐ray absorptiometry (DXA; *n* = 119), with subsets completing magnetic resonance imaging (MRI; *n* = 45) and ultrasound (US; *n* = 21). Muscle quantity included DXA‐measured appendicular lean mass/body mass index (ALM/BMI), relative skeletal muscle index (RSMI), MRI‐derived gastrocnemius cross‐sectional area and US‐derived rectus femoris thickness. Muscle quality included MRI‐derived gastrocnemius and thigh fat infiltration and US‐derived rectus femoris echogenicity. Physical performance was assessed by modified Short Physical Performance Battery (mSPPB), gait speed, 10x chair rise time, 400‐meter walk test (400MWT) and muscle strength assessed by grip strength, and one‐repetition maximum strength (1‐RM) for leg press, lateral pulldown, and bench press. Associations were assessed using Spearman correlations (*r* [95% confidence interval]), without correction for multiple comparisons.

**Results**: The 52 participants with DXA and additional imaging (MRI and/or US) had a median age of 58 (IQR 54, 62) years; 8% were women; and median HIV duration was 24 (IQR 18,30) years. Correlations between muscle quantity with grip strength and 1‐RM ranged from *r* = 0.41–0.70, with few associations with physical performance. Greater US rectus femoris thickness was associated with better chair rise time (*r* = 0.55 [0.13,0.81]). In contrast, muscle quality measures were not associated with grip strength and 1‐RM, but higher MRI‐derived thigh fat infiltration was moderately associated with poorer mSPPB performance (*r* = −0.47 [–0.71, –0.12]). US echogenicity and fat infiltrate at the calf were not associated with physical function performance. Figure 1 presents absolute correlations >0.2.

**Conclusions**: In older PWH, muscle quantity was associated with grip strength and 1‐RM measures, whereas muscle quality measured by thigh MRI was more closely associated with general physical performance. Together, these complementary measures may better identify functional vulnerability in ageing PWH.

**FIGURE 1:**
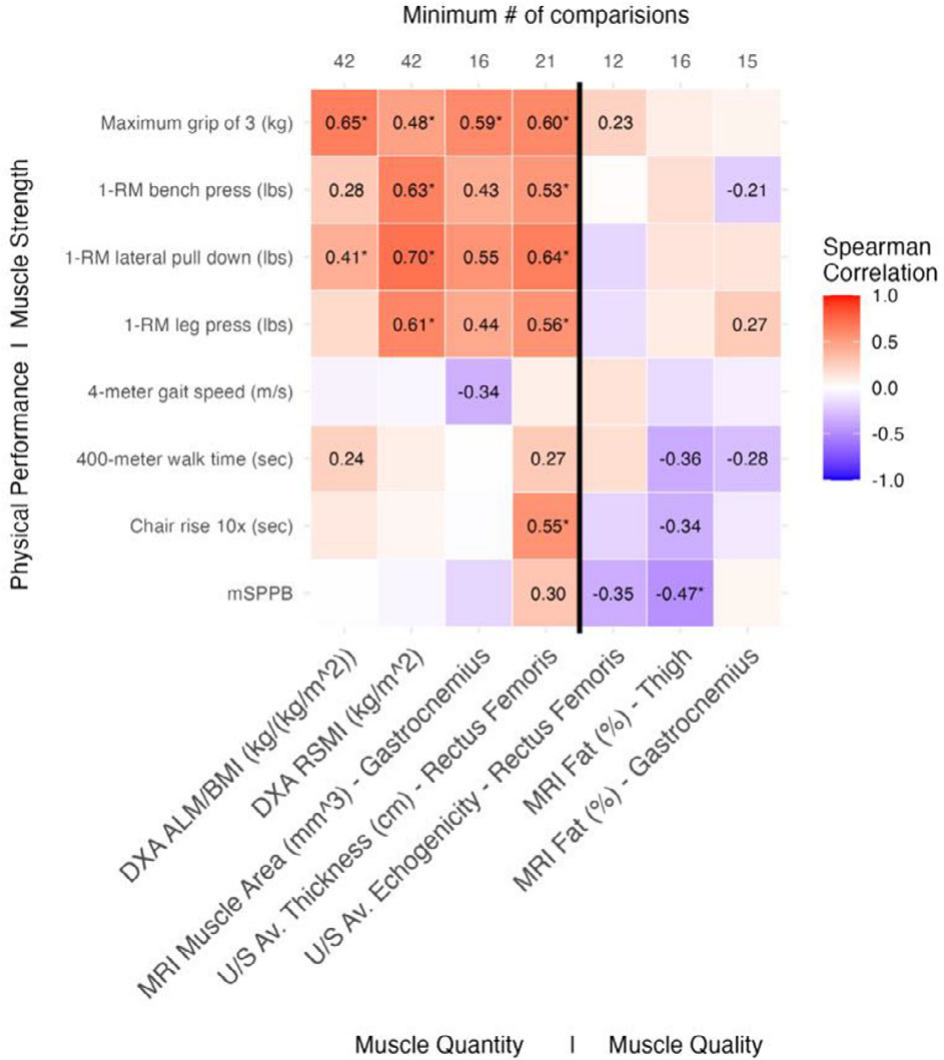
OAB2303 | Spearman correlations between muscle strength (grip strength and one‐repetition maximum strength 1‐RM) or physical performance (chair rise, 400‐meter walk, gait speed, mSPPB) and muscle quantity or quality measures (separated by black vertical line). Absolute correlations greater than 0.2 are displayed; * Indicates *p*‐values < 0.05.

## OAB2304 Predictors of Depressive Symptoms in People With HIV: A Harmonized Analysis of Cohorts in Thailand, Nigeria, Kenya, Uganda and Tanzania

T. Hamby ^1,2^, E. Schuler^1,2^, N. Burns^1,2^, H. Kibuuka^3^, J. Owuoth^4,5^, V. Sing'oei^4,5^, J. Maswai^6,7^, E. Bahemana^8,9^, Z. Parker^10,11^, N. Shah^1^, S. Sriplienchan^12^, C. Sacdalan^12,13^, R. Paul^14,15^, S. Vasan^1,10^, J. Ake^1^, T. Crowell^1,10^

^1^U.S. Military HIV Research Program, CIDR, Walter Reed Army Institute of Research, Silver Springs, United States; ^2^Henry M. Jackson Foundation for the Advancement of Military Medicine, Data Analysis and Coordinating Center, Bethesda, United States; ^3^Makerere University Walter Reed Project, Kampala, Uganda; ^4^U.S. Military HIV Research Program, Walter Reed Army Institute of Research—Africa, Kisumu, Kenya; ^5^HJF Medical Research International, Kisumu, Kenya; ^6^U.S. Military HIV Research Program, Walter Reed Army Institute of Research—Africa, Kericho, Kenya; ^7^HJF Medical Research International, Kericho, Kenya; ^8^U.S. Military HIV Research Program, Walter Reed Army Institute of Research—Africa, Mbeya, the United Republic of Tanzania; ^9^HJF Medical Research International, Mbeya, the United Republic of Tanzania; ^10^Henry M. Jackson Foundation for the Advancement of Military Medicine, Bethesda, United States; ^11^U.S. Military HIV Research Program, Walter Reed Army Institute of Research Africa, Lagos, Nigeria; ^12^SEARCH Research Foundation, Bangkok, Thailand; ^13^Chulalongkorn University, Faculty of Medicine, Bangkok, Thailand; ^14^University of Missouri, Missouri Institute of Mental Health, St. Louis, United States; ^15^University of Missouri, Department of Psychological Sciences, St. Louis, United States

**Background**: Depression is common among people with HIV (PWH) and is associated with poor HIV outcomes. We examined predictors of depression in a large, longitudinal dataset of PWH from five countries.

**Methods**: We analysed harmonized data from two ongoing cohorts: the RV254 cohort, which enrols people with acute HIV in Thailand, and the African Cohort Study (AFRICOS), which enrols PWH and people without HIV in four African countries. Questionnaires are administered every 24 or 48 weeks in RV254 and semiannually in AFRICOS, including the 9‐item Patient Health Questionnaire in RV254, and the 20‐item Center for Epidemiologic Studies Depression Scale in AFRICOS. Z‐scores were computed by study to facilitate comparisons of depressive‐symptom severity across studies. PWH with ≥1 depression measurement were included; data were collected from 2010 through 2025. A multivariable linear mixed model (LMM) was used to estimate regression coefficients (b) and 95% confidence intervals (CIs); random effects for intercepts and time were included. The b's estimate the changes, in standard deviations, in depressive‐symptom severity per unit change in predictor variables. Time since HIV diagnosis was included in the model using a spline for potentially non‐linear trends in depressive symptom‐severity over time. Other covariates of interest included demographic, substance use, sexual risk and HIV‐related variables.

**Results**: Among 3848 participants with median follow‐up of 9.5 years (interquartile range [IQR]: 6.5−13.8), 1928 (51.5%) were male, and median age was 34 (26−43) years. Nationalities included Thailand (17.7%), Uganda (14.9%), Kenya (43.0%), Tanzania (15.7%) and Nigeria (8.7%). Greater depressive‐symptom severity was observed in Kenya (*b* = 0.18, 95% CI = [0.09, 0.28], *p* < 0.001) and Tanzania (0.11, [0.01, 0.21], *p* = 0.037) versus Thailand; female sex (0.14, [0.09, 0.19], *p* < 0.001); being widowed (0.15, [0.06, 0.24], *p* = 0.001) versus married; drug use (0.16, [0.11, 0.22], *p* < 0.001); and having casual sexual partner(s) (0.13, [0.10, 0.17], *p* < 0.001). Uganda (–0.23, [–0.34, –0.12], *p* < 0.001; Figure 1) and being employed (–0.15, [–0.21, –0.09], *p* < 0.001) were associated with lower depressive‐symptom severity (Table 1).

**Conclusions**: The present analysis indicates that depressive‐symptom severity decreases with time since HIV diagnosis. Additionally, female sex, being widowed, unemployment, using drugs recreationally and having casual sex may be useful markers of depressive‐symptom severity in PWH for clinicians.

**FIGURE 1:**
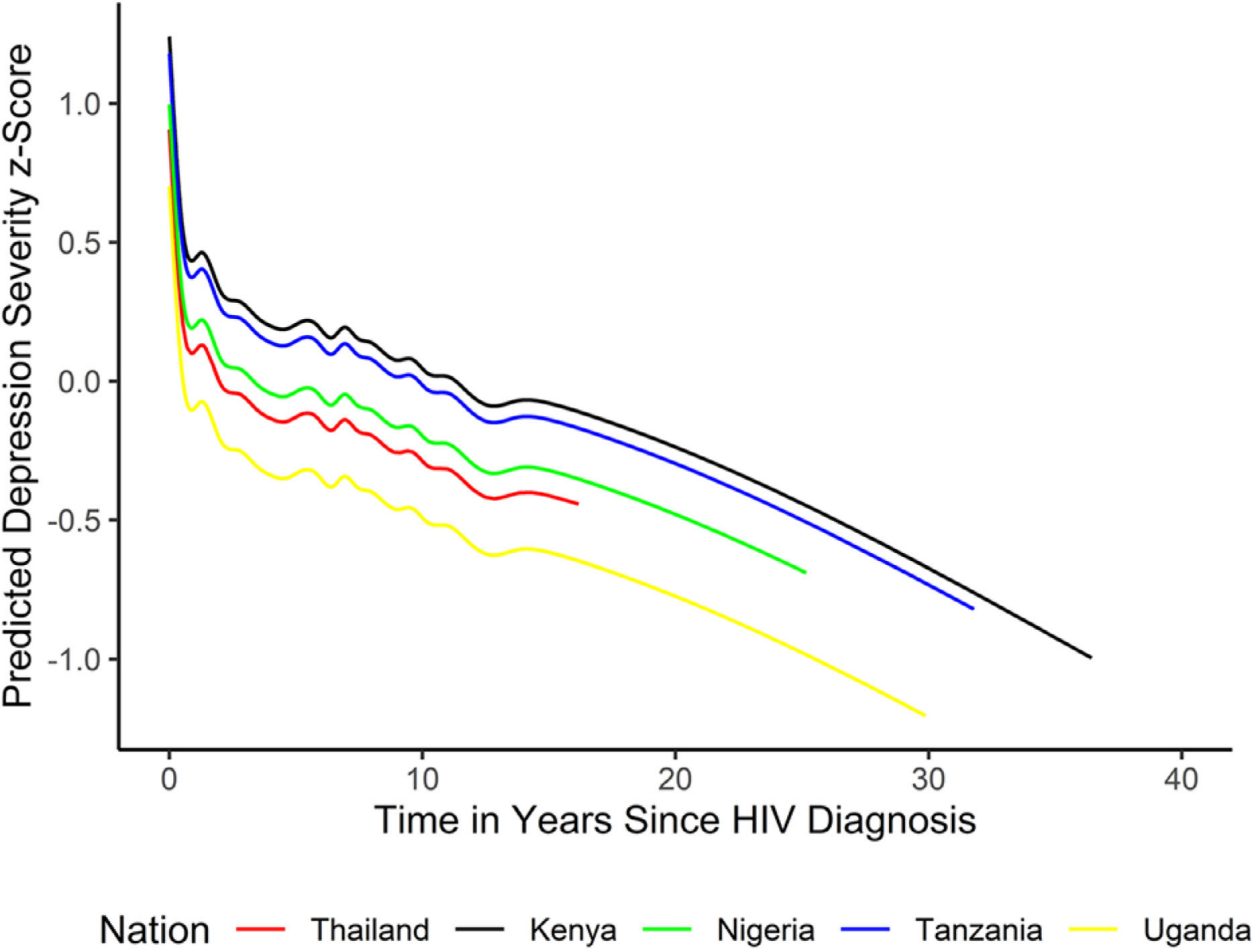
OAB2304

**TABLE 1:** OAB2304 | Descriptive statistics at enrolment and results of multivariable linear mixed model predicting depression severity z‐scores (*N* = 3848).

Variable/level	*n* (%)	*b* (95% CI)	*p*
Sex[a]: Female versus male	1866 (48.5%)	0.14 (0.09, 0.19)	<0.001
Marital status[a]: Widowed versus separated	412 (10.7%)	0.15 (0.06, 0.24)	0.001
Employed[a]: Yes versus no	1671 (43.4%)	−0.15 (–0.21, –0.09)	<0.001
Consume alcohol[b]: Yes versus no	1054 (27.4%)	0.06 (0.02, 0.09)	0.003
Use recreational drugs[b]: Yes versus no	300 (7.8%)	0.16 (0.11, 0.22)	<0.001
Has casual sex partner[b]: Yes versus no	746 (19.4%)	0.13 (0.10, 0.17)	<0.001
Taking antiretroviral therapy[b]: Yes versus no	1739 (45.2%)	−0.10 (–0.12, –0.07)	<0.001

Abbreviations: *N*, total number of participants; *n*, number of participants for variable level; *b*, fixed effect regression coefficient; CI, confidence interval; *p*, *p*‐value.

[a] Reported at enrolment for descriptive statistics and is not a time‐varying variable in the regression model.

[b] Reported at enrolment for descriptive statistics but is a time‐varying variable in the regression model.

## OAB2305 Pericoronary Fat Attenuation Index Reflects Current Rather Than Cumulative Cardiovascular Disease Risk Factors in the Canadian HIV and Aging Cohort Study

M. Durand ^1,2^, M. Messier‐Peet^2^, P.‐W. H. B. Traoré^2^, B. Mengesha^3^, V. Devine‐Ducharme^3^, V. Shirobokov^3^, B. Vulesevic^2^, M. El‐Far^2^, C. Fortin^1^, J.‐G. Baril^4^, B. Trottier^4^, M. Murray^5^, C. Tremblay^1,2^, C. Chartrand‐Lefebvre^2,6^, K. Boczar^3^, Canadian HIV and Aging Cohort Study

^1^Université de Montréal, Medicine, Montreal, Canada; ^2^Centre de Recherche du CHUM, Montreal, Canada; ^3^Univsersity of Ottawa Heart Institute, Ottawa, Canada; ^4^Clinique Médicale Urbaine du Quartier Latin, Montreal, Canada; ^5^University of British Columbia, Vancouver, Canada; ^6^Université de Montréal, Radiology, Radio‐oncology and Nuclear Medicine, Montreal, Canada

**Background**: People living with HIV (PWH) face increased risks of cardiovascular disease (CVD). In order to develop novel interventions targeting HIV‐specific pathways of CVD, we need a sensitive surrogate outcome reflecting current rather than cumulative drivers of CVD. The pericoronary fat attenuation index (PFAI), which measures inflammation in pericoronary adipose tissue, has emerged as a robust, non‐invasive biomarker predicting clinical CVD events. We investigated the association between PFAI and coronary artery disease, as well as current versus cumulative exposure to CVD risk factors in the Canadian HIV and Aging Cohort study.

**Methods**: Participants with and without HIV aged 40 years or older, with moderate CVD risk and no known prior CVD, underwent a computed tomography coronary angiography (CCTA). Subclinical CVD was defined as the presence of plaques on CCTA. PFAI was obtained by calculating the mean attenuation of all fat voxels (those with an attenuation between –190 and –30 Hounsfield units [HU]) surrounding the proximal right coronary artery. Higher values (closer to –30 HU) indicate more inflammation and increased CVD risk. Analyses were done using multivariable linear regression.

**Results**: Among 228 participants (30 women, 198 men), with a mean age of 56, 148 (65%) were PWH, with a mean duration of HIV of 18 years, and a mean CD4 count of 576 cells/µL. Subclinical CVD was associated with higher PFAI (–77 vs. –81 HU, *p* = 0.007), even after adjustment for traditional cardiovascular risk factors. HIV status was not associated with PFAI. Current (but not past or ever) smoking, current (but not past or ever) stimulant use, male sex and high blood pressure were independently associated with higher PFAI, while current statin treatment (but not LDL or HDL levels) was associated with lower PFAI. There were no association between CD4, CD4:CD8 ratio, duration or type of antiretroviral therapy and PFAI.

**Conclusions**: In a cohort of PWH, PFAI was associated with subclinical CVD, and varied by current smoking, stimulant use and statin treatment. Longitudinal analysis of PFAI in PWH is needed to determine its reactivity to changes in modifiable risk factors, and thus its potential as a surrogate outcome for interventional studies.

## OAB3402 A High Burden of Metabolic Syndrome and Metabolic Conditions in the Perinatally Acquired HIV Population in a Central London HIV Clinic

M. Barlow ^1,2^, M. Kohli^1,2^, Y. Gharib^1,2^, F. Bascombe^1,2^, A. Teague^2^, G. Lawrenson^2^, R. Gilson^1,2^, S. L. Pett^1,2,3^, A. Arenas‐Pinto^1,2,3^

^1^University College London, Institute for Global Health, London, United Kingdom; ^2^Central and North West London NHS Foundation Trust, Mortimer Market Centre, London, United Kingdom; ^3^University College London, MRC Clinical Trials Unit, London, United Kingdom

**Background**: Metabolic syndrome (MetS) is well‐described among those with horizontally acquired HIV (HaHIV), predicting an increased risk of cardiovascular disease. However, very little is known about the burden of metabolic conditions in those with perinatally acquired HIV (PaHIV), with prolonged exposure to HIV and anti‐retroviral therapy (ART) during major growth and development. We sought to estimate prevalence of MetS and metabolic conditions in the PaHIV sub‐population of our large United Kingdom (UK) HIV clinic, and compare with the overall clinic population.

**Methods**: Retrospective cross‐sectional analysis was performed on routine clinical data of PaHIV aged ≥16 who received specialist adolescent perinatal HIV care at a central London HIV clinic from 2015 to 2025. Metabolic conditions included a confirmed diagnosis, and/or treatment of the following: Diabetes (HbA1c >48 mmol/mol), hypertension (systolic BP >130 mmHg), dyslipidaemia (triglyceridaemia >1.7 mmol/L and/or low high‐density lipoprotein [HDL]) (men < 1.0 mmol/L; women < 1.3) and obesity (BMI ≥30 kg/m^2^). Chi‐square testing explored association between MetS with demographics, clinical markers and antiretroviral therapy (ART). Two‐sample Z‐testing was used for the difference between population proportions of metabolic conditions.

**Results**: 191 PaHIV were identified. Median age was 29 (range 16−41). The majority were of Black African ethnicity (81.2%), about half (48.7%) born in the UK and 45.5% were female. Prevalence of metabolic conditions were high: 16.2% met MetS criteria, significantly higher than the HAHIV cohort (Figure 1). 73.3% had ≥1 metabolic conditions. 55.3% of PaHIV were overweight, and 29.5% obese. 51.3% had hypertension, followed by dyslipidaemia (39.0%). Half (50.3%) had had an AIDS‐defining diagnosis, and 2.1% had died. Figure 1 displays the comparison of the adult and perinatal cohort. In people with PaHIV, depression was associated with MetS (*p* 0.037), whereas age, sex, ethnicity, viral load>50 and various ART were not (*p* >0.05) (Table 1).

**Conclusions**: MetS and its conditions are disproportionately prevalent in this younger PaHIV population. The MetS burden appears to be driven by obesity compared with the HaHIV cohort, with dyslipidaemia and diabetes comprising metabolic burden in the older HaHIV. This high prevalence of metabolic syndrome and conditions puts PaHIV at high risk of morbidity and mortality. There is an urgent need for guidelines for early screening and targeted interventions including weight‐loss.

**FIGURE 1:**
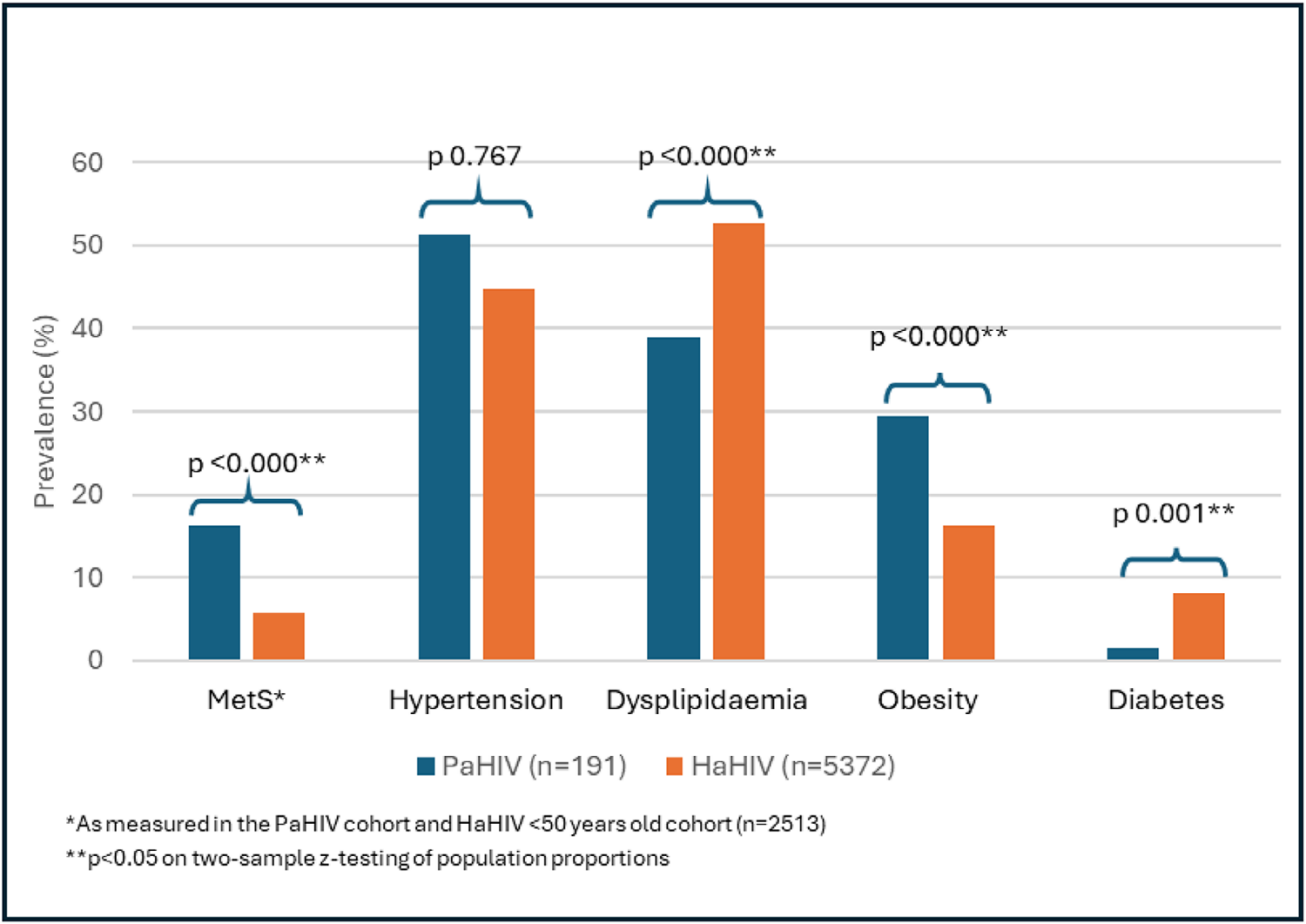
OAB3402 | Comparative prevalence of MetS and metabolic conditions in PaHIV and HaHIV.

**TABLE 1:** OAB3402 | Demographics, HIV parameters and ART association with metabolic syndrome (MetS) in a population of people living with perinatally acquired HIV (PaHIV) (*n* = 191).

	MetS *n* (%)	No MetS *n* (%)	Chi^2^ *p* value
**Age (years)**			0.384
<30	15 (48.4)	91 (56.9)	
≥30	16 (51.6)	69 (43.1)	
**Gender**			0.729
Male	15 (48.4)	72 (45.0)	
Female	16 (51.6)	88 (55.0)	
**Ethnicity**			0.205
Black African	28 (90.3)	127 (79.4)	
White	2 (6.5)	9 (5.6)	
Other	1 (3.2)	24 (15.0)	
**Viral load (*n* = 189)**			0.066
<50 copies/mL	16 (53.3)	112 (70.4)	
≥50 copies/mL	14 (46.7)	47 (29.6)	
**AIDS‐defining illness**			0.342
Yes	18 (58.1)	78 (48.8)	
No	13 (41.9)	82 (51.2)	
**Smoking status (*n* = 187)**			0.925
Smoker	9 (29.0)	42 (26.9)	
Ex‐smoker	2 (6.5)	13 (8.3)	
Non‐smoker	20 (64.5)	101 (64.8)	
**Depression**			0.037*
Yes	12 (38.7)	34 (21.3)	
No	19 (61.3)	126 (88.7)	
**Protease inhibitor use (*n* = 181)**			0.106
Yes	24 (82.8)	103 (67.8)	
No	5 (17.2)	49 (32.2)	

*Metabolic syndrome prevalence of the PaHIV (perinatally‐acquired HIV) cohort.

## OAB3403 Subclinical Coronary Atherosclerosis Is Prevalent Among People Living With HIV in Peru

M. A. Huaman ^1,2^, A. La Rosa^3^, J. Narrea^3^, J. R. Lama^3^, B. Zhang^4^, M. Leon^3^, J. Valencia^3^, S. E. Ramirez^5^, R. Egoavil‐Espejo^2^, M. Vilchez^3^, M. Sanchez^3^, L. Giarletta^6^, M. van Assen^6^, C. Chang^3^, S. Andorf^7^, C. A. Chougnet^8^, C. N. De Cecco^6^, C. T. Longenecker^9^

^1^Emory University, Medicine, Atlanta, United States; ^2^University of Cincinnati, Medicine, Cincinnati, United States; ^3^Asociacion Civil Impacta Salud y Educacion, Lima, Peru; ^4^Cincinnati Children's Hospital Medical Center, Biostatistics, Cincinnati, United States; ^5^Clinica Internacional, Unidad de Diagnostico por Imagen Cardiovascular, Lima, Peru; ^6^Emory University, Radiology and Imaging Sciences, Atlanta, United States; ^7^Cincinnati Children's Hospital Medical Center, Biomedical Informatics and of Allergy and Immunology, Cincinnati, United States; ^8^Cincinnati Children's Hospital Medical Center, Immunobiology, Cincinnati, United States; ^9^University of Washington, Medicine and Global Health, Seattle, United States

**Background**: People with HIV (PWH) have an elevated risk of cardiovascular disease, yet data on subclinical coronary atherosclerosis in Latin America are unknown. We estimated the prevalence and factors associated with coronary atherosclerosis in PWH in Peru.

**Methods**: We enrolled asymptomatic PWH aged 40–75 years on stable antiretroviral therapy in Lima, Peru between 2/2023 and 4/2025. Individuals with history of cardiovascular disease events or receiving lipid‐lowering medications were excluded. Participants underwent coronary computed tomography angiography (CCTA) and cardiometabolic laboratory testing. Coronary atherosclerotic disease (CAD) was defined as presence of any calcified or non‐calcified plaque by CCTA; obstructive CAD as presence of plaque causing >50% stenosis. Logistic regressions investigated the relationship between CAD and the Pooled Cohort Equations 10‐year atherosclerotic cardiovascular disease (ASCVD) risk score, traditional cardiovascular factors, HIV‐related parameters and poverty likelihood score.

**Results**: Of 500 participants, 464 (93%) completed CCTA. Of them, median age was 47 years (interquartile range [IQR], 43–53); 63 (14%) were female (see Table 1). Median HIV duration was 12.3 years (IQR, 8.7−16.3); 99% had HIV viral load ≤200 copies/mL. Most (86%) antiretroviral regimens contained an integrase inhibitor. Reported comorbidities included dyslipidaemia (9%), hypertension (8%), diabetes (3%) and smoking (19%). The median 10‐year ASCVD risk score was 2.7% (IQR, 1.3%–5.0%). CAD prevalence was 49% (95% CI, 45%–54%), with obstructive CAD identified in 9% (95% CI, 6%–12%). ASCVD risk score was associated with CAD (odds ratio [OR], 1.23; 95% CI, 1.15–1.32). Older age (adjusted OR per 10 years, 2.51, 95% CI, 1.82–3.48), higher body mass index (adjusted OR per 5 kg/m^2^, 1.38, 95% CI, 1.07–1.78) and smoking (adjusted OR, 2.02; 95% CI, 1.18–3.47) were independently associated with CAD. HIV‐related parameters and poverty score were not independently associated with CAD, after adjusting by ASCVD scores.

**Conclusions**: Nearly half of PWH on long‐term antiretroviral therapy had subclinical coronary atherosclerosis in Lima, Peru. These findings underscore the need for targeted lifestyle and pharmacologic interventions to reduce cardiovascular morbidity in PWH in the region.

**TABLE 1:** OAB3403 | Baseline characteristics of the study population by CAD.

Characteristics	Total (*n* = 464)	CAD (*n* = 229)	No CAD (*n* = 235)
Age, years (median, IQR)	47 (43, 53)	49 (44, 56)	45 (42, 50)
Female sex at birth, *n* (%)	63 (14%)	26 (11%)	37 (16%)
History of diabetes, *n* (%)	14 (3%)	9 (4%)	5 (2%)
History of dyslipidaemia, *n* (%)	43 (9%)	21 (9%)	22 (9%)
History of hypertension, *n* (%)	39 (8%)	27 (12%)	12 (5%)
Current smoking, *n* (%)	90 (19%)	55 (24%)	35 (15%)
BMI kg/m^2^ (median, IQR)	26.8 (24.4, 29.1)	27.1 (24.7, 29.8)	26.5 (24.2, 28.4)
LDL cholesterol mg/dL (median, IQR)	103 (83, 123)	105 (84, 122)	101 (81, 125)
Hb A1c, % (median, IQR)	5.5 (5.3, 5.8)	5.6 (5.3, 5.8)	5.5 (5.2, 5.7)

## OAB3404 Body Weight and BMI Changes Associated With DTG Treatment in Brazil: Results From CODE, A Country‐Wide Cohort Study

K. Page^1^, A. Aragon^1^, J. Anderson^1^, M. Carmody^1^, M. V. G. Lacerda^2^, A. Ramalho^3^, F. Bahia^4^, V. Madruga^5^, S. W. Cardoso^6^, C. Murray‐Krezan^7^, C. Brites ^8^, CODE Study Group

^1^University of New Mexico Health Sciences Center, Internal Medicine, Albuquerque, United States; ^2^Fundação de Medicina Tropical do Amazonas, Infectious Disease, Manaus, Brazil; ^3^Hospital Geral de Nova Iguaçu, Infectious Disease, Rio de Janeiro, Brazil; ^4^Centro Estadual Especializado em Diagnóstico Assistência e Pesquisa, Infectious Disease, Salvador, Brazil; ^5^Centro de Referência e Tratamento DST/AIDS, Sao Paulo, Brazil; ^6^Instituto Nacional de Infectologia Evandro Chagas, Rio de Janeiro, Brazil; ^7^University of Pittsburgh, Medicine, Pittsburgh, United States; ^8^Universidade Federal da Bahia/Fundacao Bahiana de Infectologia, Infectious Disease, Salvador, Brazil

**Background**: Dolutegravir (DTG)‐based antiretroviral therapy (ART) was adopted as first‐line therapy in Brazil in 2017. Adverse metabolic effects, particularly weight gain, are important to monitor as DTG regimens are scaled up globally, yet population‐level data from South America remain limited. The Clinical Outcomes of DTG Treatment in People Living with HIV in Brazil (CODE) study follows 4525 people living with HIV (PLHIV) across 15 sites is assessing real‐world outcomes of DTG rollout. We assessed significant weight gain (SWG, ≥10% increase) and BMI changes at 6 and 12 months post‐initiation or switch to DTG‐based ART.

**Methods**: We analysed four groups enrolled March 2022–January 2026: (1) ART‐naïve initiating DTG (*n* = 1972 at 6 months; *n* = 1870 at 12 months); (2) suppressed clients switching to DTG (*n* = 463; *n* = 454); (3) clients switching due to virologic failure (*n* = 192; *n* = 181); (4) non‐DTG comparator (*n* = 131; *n* = 123). Primary outcomes were SWG (≥10% weight increase) and median weight/BMI changes from baseline.

**Results**: Baseline median weights were similar across groups (67.8−70.5 kg) with BMI 23.5−25.2. At 6 months, median weight gains were: Group 1: +1.9 kg (IQR: –0.5, 5.1); Group 2: +1.0 kg (IQR: –0.7, 2.8); Group 3: +1.6 kg (IQR: –0.3, 5.0); Group 4: +0.1 kg (IQR: –0.7, 3.5). At 12 months: Group 1: +2.9 kg (IQR: –0.3, 7.0); Group 2: +1.3 kg (IQR: –0.6, 4.0); Group 3: +3.2 kg (IQR: 0, 6.1); Group 4: +1.1 kg (IQR: –1.0, 4.5). SWG at 12 months occurred in 25% (Group 1), 9% (Group 2), 26% (Group 3) and 15% (Group 4). Corresponding BMI increases at 12 months were +1.0, +0.4, +1.2 and +0.5 units, respectively. ART‐naïve and virologic failure groups showed the highest proportions with SWG, though absolute gains remained modest for most participants.

**Conclusions**: DTG‐based ART was associated with moderate weight gain in Brazilian PLHIV, with one‐quarter of treatment‐naïve and salvage therapy patients experiencing ≥10% increases by 12 months. Weight gain was lower in stable switchers, suggesting the “return‐to‐health” phenomenon in ART‐naïve individuals contributes substantially. Future analyses will examine predictors of weight gain including sex, age, baseline BMI and specific DTG‐based regimen components. (Table 1 and 2)

**TABLE 1:** OAB3404 | Key baseline characteristics of the CODE cohort by group.

Group	1 (*N* = 2273)	2 (*N* = 502)	3 (*N* = 207)	4 (*N* = 1528)
Age (years) Median (IQR)	30 (15)	50 (16)	42 (17)	35 (17)
Sex‐Male (*N*, %)	1848 (81.3%)	327 (65.1%)	97 (46.9%)	1096 (71.7%)
CD4 count, Median (IQR)	367 (355)	681 (371)	289 (400)	347 (357)
HIV viral load, Median (IQR)	57,175 (227,627)	25 (0)	22,789 (123,044)	26,055 (115,119)
Weight (kg), Median (IQR)	68 (19.7)	70.5 (17.8)	67.8 (20)	68.1 (19.3)
BMI, Median (IQR)	23.5 (6.2)	25.2 (6.1)	24.9 (7.6)	24.1 (6.6)

**TABLE 2:** OAB3404 | 6‐ and 9‐month changes in body weight and body mass index in the CODE cohort by group.

	Group 1	Group 2	Group 3	Group 4
Group	6‐mo (*N* = 1972)	6‐mo (*N* = 463)	6 mo (*N* = 192)	6 mo (*N* = 131)
6‐mo. Wt change Kg. (median [IQR])	1.9 (–0.5, 5.1)	1.0 (–0.7, 2.8)	1.6 (–0.3, 5)	0.1 (–0.7, 3.5)
SWG @ 6 mo. (*N* %)	342 (17%)	20 (4%)	33 (17%)	18 (14%)
6‐mo. BMI change (median [IQR])	0.6 (–0.2, 1.8)	0.4 (–0.2, 1)	0.6 (–0.1, 2.1)	0.4 (–0.1, 1.9)
	12‐mo (*N* = 1870)	12‐mo (*N* = 454)	12‐mo (*N* = 181)	12‐mo (*N* = 123)
12‐mo. Wt change Kg. (median [IQR])	2.9 (–0.3, 7)	1.3 (–0.6, 4)	3.2 (0, 6.1)	1.1 (–1, 4.5)
SWG @ 12 mo. (*N* %)	470 (25%)	43 (9%)	47 (26%)	18 (15%)
12‐mo. BMI change (median [IQR])	1 (–0.1, 2.4)	0.4 (–0.2, 1.4)	1.2 (0, 2.4)	0.5 (–0.3, 2.6)

## OAB3405 The Body Composition Signature of Switching to BIC/FTC/TAF or DTG/3TC Over 96 Weeks

S. Di Gregorio^1^, L. R. Brun^2^, S. Mourelo^3^, M. J. Crusells^4^, P. Domingo^5^, A. Curran^6^, R. Güerri^7^, M. Masia^8^, L. J. Garcia‐Fraile^9^, P. Ryan^10^, J. L. Blanco^11^, M. J. Vazquez^12^, M. De Miguel^13^, B. Alejos^14^, E. Martinez ^11^, on behalf of the PASO‐DOBLE (GeSIDA 11720) Study Group

^1^CP Endocrinologia i Nutrició S.L., Barcelona, Spain; ^2^Universidad Nacional de Rosario, Rosario, Argentina; ^3^CETIR ASCIRES, Barcelona, Spain; ^4^Hospital Clínico Universitario Lozano Blesa, Zaragoza, Spain; ^5^Hospital de la Santa Creu i Sant Pau, Barcelona, Spain; ^6^Hospital Universitario Vall d'Hebron, Barcelona, Spain; ^7^Hospital del Mar, Barcelona, Spain; ^8^Hospital General Universitario, Elche, Spain; ^9^Hospital Universitario de la Princesa, Madrid, Spain; ^10^Hospital Universitario Infanta Leonor, Madrid, Spain; ^11^Hospital Clínic & University of Barcelona, Barcelona, Spain; ^12^ViiV Healthcare, Tres Cantos, Spain; ^13^Fundación SEIMC‐GeSIDA, Madrid, Spain; ^14^Independent researcher, Madrid, Spain

**Background**: Weight gain after switching to integrase strand transfer inhibitors is well described, but weight does not differentiate fat from lean mass nor identify adipose depot selection, which may influence the clinical timing of metabolic comorbidities. Whether fat is initially buffered in subcutaneous depots or diverted to visceral adipose tissue (VAT) remains unclear. We evaluated 96‐week body composition changes after switching to BIC/FTC/TAF or DTG/3TC in the PASO‐DOBLE study (NCT04884139).

**Methods**: Virologically suppressed adults were randomized 1:1 to DTG/3TC or BIC/FTC/TAF. Dual‐energy X‐ray absorptiometry (DXA) and abdominal computed tomography (CT, L4) were obtained at baseline, week 48 and week 96. DXA quantified total, regional and visceral fat and lean mass; CT quantified abdominal subcutaneous (SAT) and VAT. Metabolic assessments included fasting lipids, glucose, haemoglobin A1c, HOMA‐IR and cardiometabolic medications. Longitudinal changes were analysed using mixed‐effects models.

**Results**: Among 446 participants (DTG/3TC *n* = 224; BIC/FTC/TAF *n* = 222), total fat increased and lean mass declined with both regimens, such that changes in body weight underestimated adipose accrual. DXA showed increases in total and regional fat with larger gains under BIC/FTC/TAF than DTG/3TC, while visceral fat accrual was similar between arms. CT confirmed increases in abdominal SAT and VAT in both arms, with no between‐arm differences in either depot; notably, SAT expansion attenuated between week 48 and week 96, whereas VAT accumulation persisted through week 96, consistent with an initial subcutaneous phase followed by delayed visceral participation (Table 1). Despite adipose remodelling, metabolic markers remained largely unchanged within each arm and cardiometabolic prescriptions were comparable between arms at week 96, suggesting a temporal lag between adipose redistribution and metabolic expression (Table 2).

**Conclusions**: Switching to BIC/FTC/TAF or DTG/3TC led to increased fat mass with loss of lean mass, while weight underestimated adipose accrual. Fat expansion was initially subcutaneous and was followed by later visceral involvement, without early metabolic deterioration at 96 weeks. The delayed visceral component suggests metabolic consequences may emerge beyond this period, indicating that weight‐based monitoring may miss clinically relevant adipose remodelling and supporting the incorporation of simple anthropometric assessments of visceral adiposity, such as waist circumference, into routine HIV care.

**TABLE 1:**
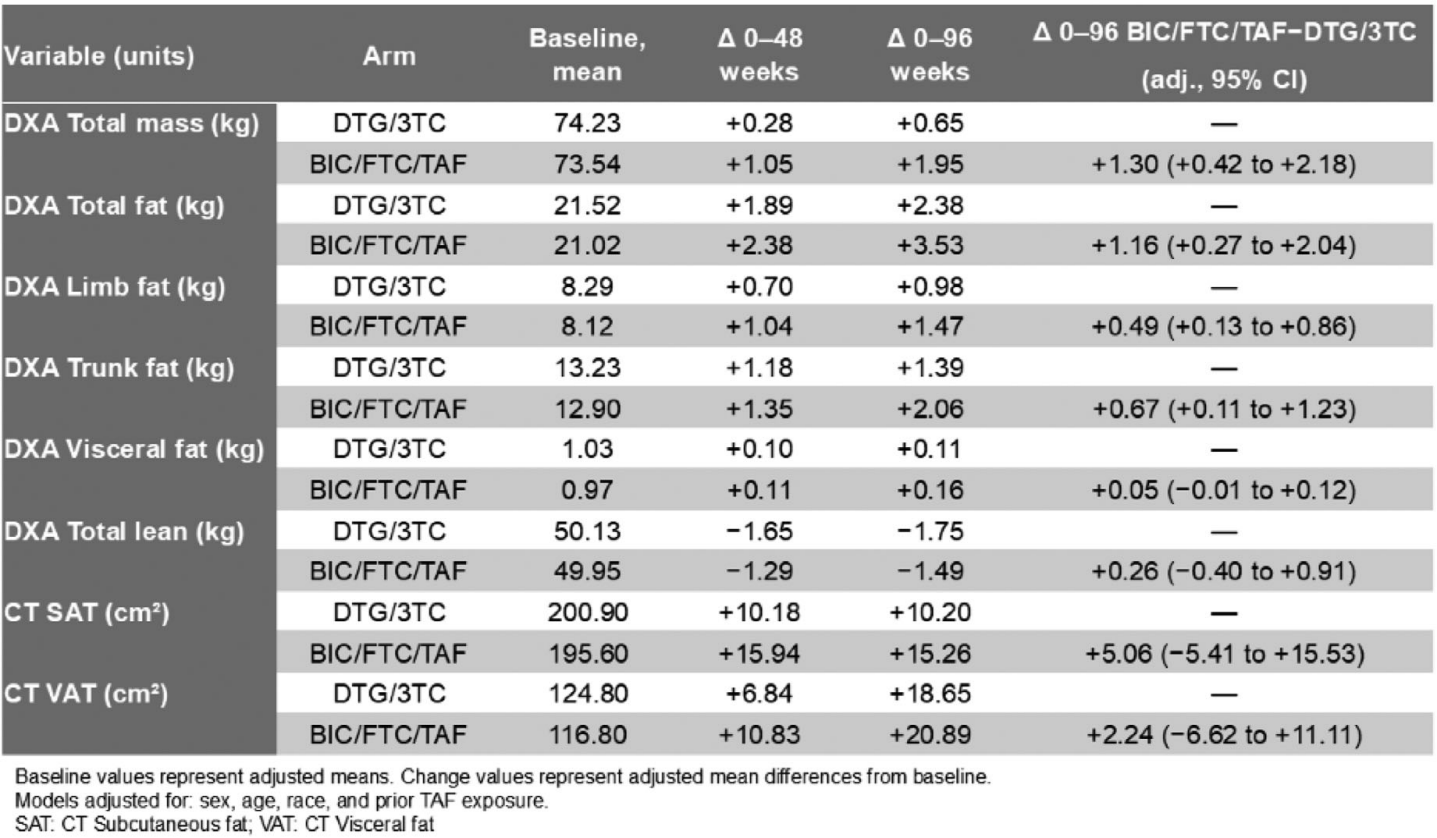
OAB3405 | Body composition changes from baseline to weeks 48 and 96.

**TABLE 2:**
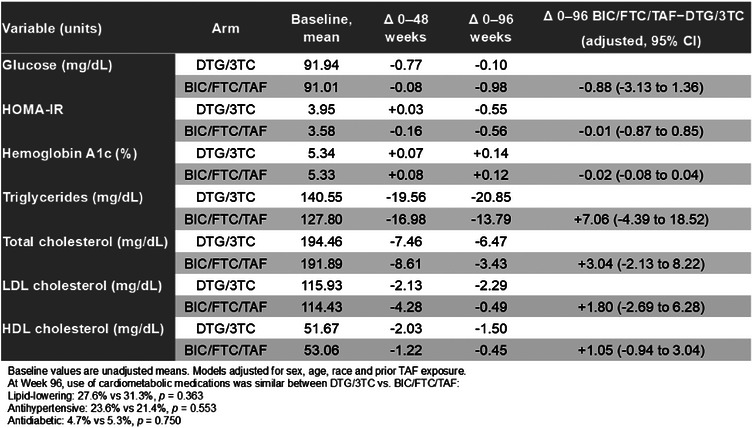
OAB3405 | Metabolic markers from baseline to weeks 48 and 96 and cardiometabolic medications to week 96.

## OAB4102 Tuberculosis Amidst the High Tide of Antiretroviral Therapy Interruptions: A Retrospective Cohort Study in South Africa

H. Moolla ^1^, C. Chinogurei^1^, M.‐A. Davies^1,2^, G. Meintjes^3,4^, J. Euvrard^1^, A. Boulle^1,2^, T. Jacobs^2^, J. Arendse^5,6^, H. Goeiman^6^, L. F. Johnson^1^

^1^University of Cape Town, Cape Town, South Africa; ^2^Western Cape Government, Health Intelligence Directorate, Cape Town, South Africa; ^3^Queen Mary University of London, Blizard Institute, London, United Kingdom; ^4^University of Cape Town, Department of Medicine and Institute of Infectious Disease and Molecular Medicine, Cape Town, South Africa; ^5^Stellenbosch University, Division of Health Systems and Public Health, Stellenbosch, South Africa; ^6^Western Cape Government, Department of Health and Wellness, Cape Town, South Africa

**Background**: Tuberculosis (TB) remains a leading cause of morbidity and mortality among people living with HIV. While antiretroviral therapy (ART) has reduced TB incidence, there remains a substantial TB burden in ART‐experienced individuals. We aimed to estimate TB incidence after ART initiation and estimate the effects of ART interruptions and other risk factors on incident TB.

**Methods**: This retrospective cohort study included adults starting ART for the first time between 2016 and 2024 in South Africa using demographic and clinical data from people receiving care in the public health system. We excluded people with prior TB and those who developed TB within 90 days of first starting ART, as the latter cases may reflect unmasking of TB by ART. Follow‐up ended at the earliest of first TB episode, death or last recorded ART prescription. Interruptions were defined by gaps of more than 28 days without medication in hand. We estimated adjusted hazard ratios (aHRs) for incident TB using a Cox proportional hazards model.

**Results**: Among 185,993 adults included, 68.0% were female. Median age was 32.0 years and median baseline CD4 count was 330 cells/µL. Median follow‐up time inclusive of interruptions was 2.82 years. The proportion of participants with a history of interruption increased from 20.2% in 2016 to 66.4% in 2024. Median interruption duration was 62 days. Overall TB incidence was 2.12 per 100 person‐years, with 56.4% of cases occurring during interruptions. Compared with first‐time ART use, TB risk was higher during interruptions (aHR 2.33, 95% confidence interval [CI]: 2.13−2.54), increasing by 1.030 (95% CI: 1.027−1.032) per month of interruption. TB risk remained elevated after resuming ART (aHR 1.52, 95% CI: 1.43−1.62). Risk was higher when not virally suppressed (aHR 3.83, 95% CI: 3.47−4.22), among males (aHR 1.51, 95% CI: 1.45−1.56) and increased with age (except among those over 65 years) and decreasing baseline CD4 count (Figure 1). Higher risk was also associated with chronic obstructive pulmonary disease, chronic kidney disease, epilepsy and mental health conditions.

**Conclusions**: ART interruptions and viral non‐suppression markedly increase TB risk. Improving treatment continuity and interventions supporting viral suppression will substantially reduce TB incidence.

**FIGURE 1:**
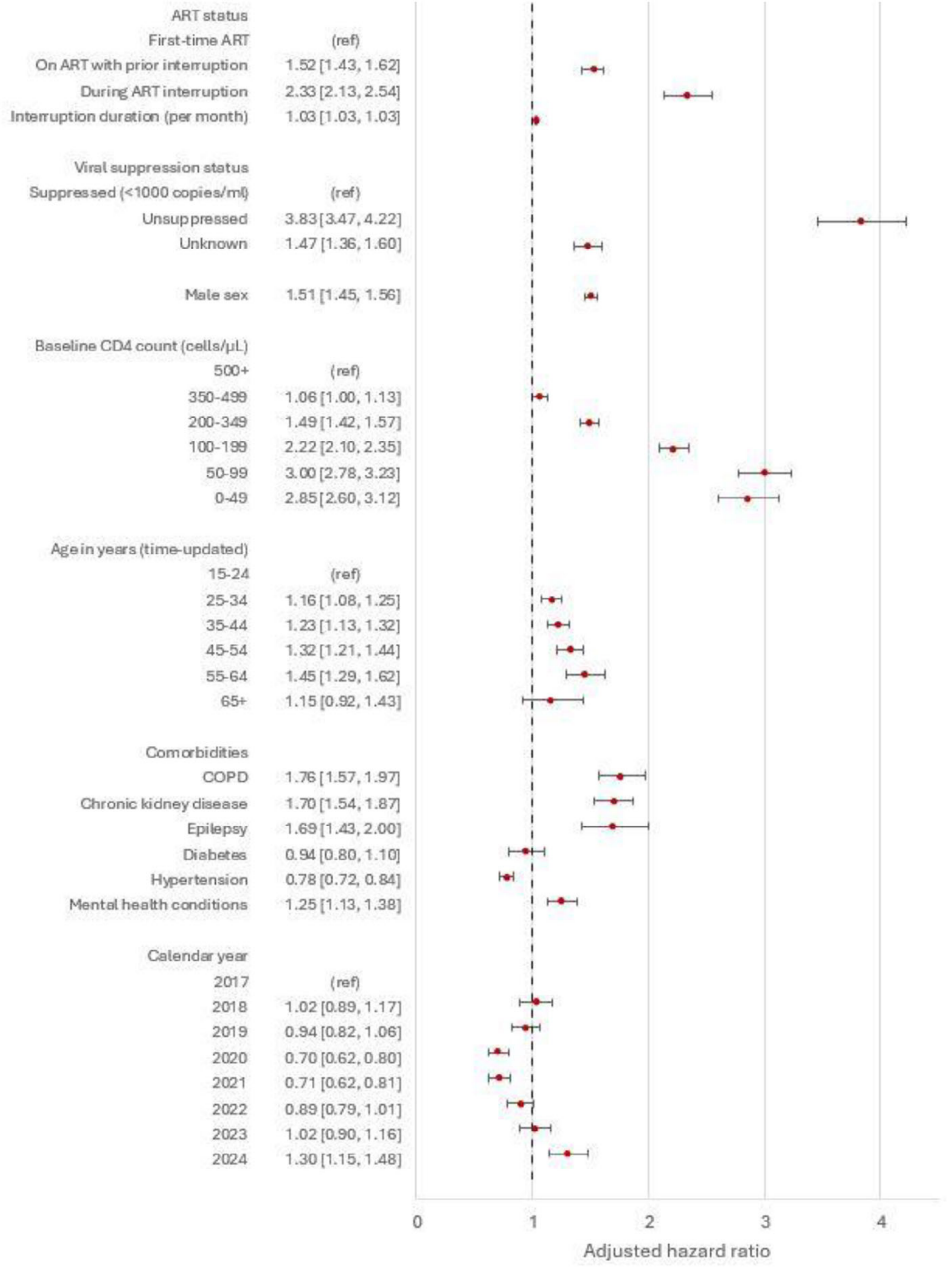
OAB4102

## OAB4103 High Prevalence of Opportunistic Infections Detected Across all CD4 Count Strata Through the Implementation of a Point‐of‐Care Testing Package for Opportunistic Infections in Latin America and the Caribbean

A. Camiro‐Zuñiga ^1,2^, O. Sued^2^, E. Candia^3^, M. Cardozo^4^, C. Benitez^5^, A. Garcia^5^, A. Pasqualotto^6^, C. Rios^7^, R. Luque^7^, A. Sebro^8^, M. Thormann^9^, W. Batista^9^, R. R. Tobar Robalino^10^, T. Jagnarine^11^, F. Pérez^2^

^1^Instituto Nacional de Cancerologia, Infectious Diseases, Mexico City, Mexico; ^2^Pan American Health Organization, CDE/HT, Washington, United States; ^3^Ministerio de Salud Pública y Bienestar Social, Programa Nacional de Control de VIH/sida e ITS, Asuncion, Paraguay; ^4^Instituto de Medicina Tropical de Asuncion, Asuncion, Paraguay; ^5^Ministerio de Salud, Estrategia Nacional de VIH, Lima, Peru; ^6^Universidade Federal De Ciências da Saúde de Porto Alegre, Porto Alegre, Brazil; ^7^Ministerio de Salud y Protección Social, Bogota, Colombia; ^8^Ministry of Health, Port of Spain, Trinidad and Tobago; ^9^Ministerio de Salud, Dirección Nacional de Enfermedades Transmisibles, Santo Domingo, the Dominican Republic; ^10^Ministerio de Salud, Programa Nacional de VIH, Quito, Ecuador; ^11^Ministry of Health, National Aids Programme Secretariat, Georgetown, Guyana

**Background**: Opportunistic infections (OIs) such as tuberculosis (TB), cryptococcosis and histoplasmosis remain leading causes of morbidity and mortality among people with advanced HIV disease (AHD) in Latin America and the Caribbean (LAC). However, routine implementation of antigen‐based tests remains limited in the Region.

**Methods**: We pooled data from eight prospective implementation studies conducted in LAC that evaluated a standardized opportunistic infection (OI) screening package among individuals with suspected AHD. The package included urinary TB‐LAM, serum Cryptococcus antigen (CrAg) and urinary Histoplasma antigen (HUAg). Eligible participants were naive individuals and <90 days of effective antiretroviral therapy (ART) or those who had interrupted ART for >90 days, who either had CD4 counts <200 cells/µL or presented with symptoms suggestive of an active OI regardless of CD4 count. Prevalence was defined as the proportion of positive tests among all tests performed. Among participants who received all three diagnostic tests and had quantitative CD4 measurements, prevalence was stratified by CD4 categories. Crude mortality was evaluated 30 days after enrolment.

**Results**: Among 2143 participants, TB‐LAM was positive in 407/1875 (21.7%), CrAg in 161/1917 (8.4%) and HUAg in 136/1753 (7.8%). Median age was 39 years (IQR 30.9–48.1), 63.7% were men and 48.8% were ART‐naïve at presentation. A total of 1088 participants received the full diagnostic package and had CD4 results available. Overall, 30‐day mortality was 11.2% and was highest among individuals with CD4 counts <50 cells/µL (16.4%). Mortality was higher among individuals with any OI (17.7%; OR 2.28) and for each individual positive test: TB‐LAM (15.7%; OR 1.55), CrAg (22.4%; OR 2.57) and HUAg (25.2%; OR 2.96).

**Conclusions**: Systematic antigen‐based screening reveals a substantial burden of OIs among people with suspected AHD in LAC, particularly among people with CD4 counts <50 cells/µL, who represented a third of enrolled participants. Notably, a significant proportion of TB and histoplasmosis diagnoses occurred among individuals with CD4 counts >200 CD4 cells/µL. which has important implications for routine screening strategies. Mortality remains high and is strongly associated with low CD4 and OIs, particularly histoplasmosis (see Table 1 and Figure 1).

**TABLE 1:** OAB4103 | Proportion of positive antigen tests and 30‐day mortality by CD4 stratum.

CD4 count	Total individuals	Positive TB LAM	Positive CrAg	Positive HuAg	>1 positive tests	All tests negative	30‐day mortality
<50	373	30.03%	13.88%	14.11%	12.33%	56.03%	16.40%
51−100	228	22.37%	9.92%	9.52%	4.39%	61.84%	9.56%
101−200	266	20.30%	5.83%	3.26%	2.63%	72.18%	6.81%
201−350	146	15.07%	4.85%	4.24%	2.74%	77.40%	5.46%
>350	75	18.67%	3.19%	1.06%	1.33%	77.33%	3.96%
Total	1088	23.26%	9.93%	8.55%	6.25%	65.53%	10.15%

**FIGURE 1:**
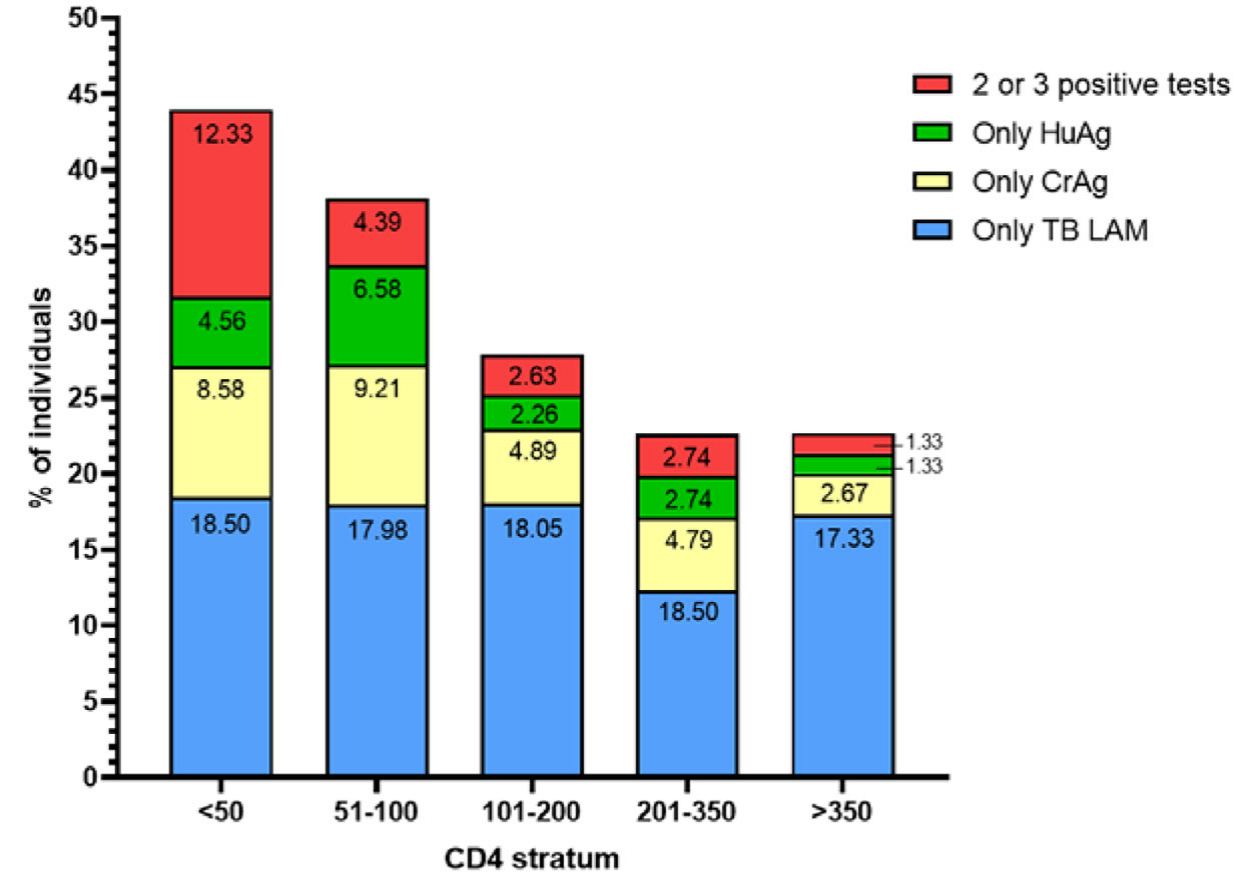
OAB4103 | Proportion of people with positive antigen tests by CD4 stratum, excluding overlapping tests.

## OAB4104 Evaluation of Global Treatment Decision Algorithms for Tuberculosis Screening in Children Living With HIV: An Analysis of the TB GAPS Study, July 2023−October 2025

D. Vambe ^1,2^, A. Seeger^1^, M. Dube^2^, B. E. O'Brien^1,3^, A. Munthali^3^, T. Steffy^1,4^, L. Komba^5^, P. Dlamini^2^, P. Elyanu^6^, C. Cazier^1^, D. Mulengwa^2^, S. Nandakumar^7^, J. Doyle^7^, A. Date^7^, A. Mandalakas^1^, A. Kay^1,2^, TB GAPS Study Group

^1^Baylor College of Medicine, Pediatrics, Global Immigrant Health, Houston, United States; ^2^Baylor Children's Foundation Eswatini, Global TB Program, Mbabane, Eswatini; ^3^Baylor Children's Foundation Malawi, Global TB Program, Lilongwe, Malawi; ^4^Baylor Children's Foundation Lesotho, Global TB Program, Maseru, Lesotho; ^5^Baylor Children's Foundation Tanzania, Global TB Program, Mbeya, the United Republic of Tanzania; ^6^Baylor Children's Foundation Uganda, Global TB Program, Kampala, Uganda; ^7^U.S. Centers for Disease Control and Prevention, Division of Global HIV and Tuberculosis, Atlanta, United States

**Background**: In 2022, the World Health Organization (WHO) published Treatment Decision Algorithms (TDAs) to improve paediatric tuberculosis (TB) diagnosis. TDAs were developed for children <10 years with symptoms suggesting pulmonary TB. However, TB is often asymptomatic and symptom‐agnostic TB screening tools exist for people with HIV, such as C‐reactive protein (CRP) and chest x‐ray (CXR). We evaluated the performance of TDAs with expanded, symptom‐agnostic entry criteria in children with HIV (CWH) aged 0−9 years.

**Methods**: TB GAPS enrolled CWH into a prospective TB screening and diagnostic cohort in five high‐TB‐burden countries: Eswatini, Malawi, Lesotho, Uganda and Tanzania. All children received CRP and CXR despite symptoms. We compared the diagnostic accuracy of TDAs (TDA‐A includes CXR and TDA‐B excludes CXR) using standard (WHO‐defined TB symptoms for TDA entry) and expanded entry criteria including TB symptoms of any duration, CRP ≥5 mg/L or an abnormal CXR. Sensitivity, specificity and diagnostic yield were calculated using the U.S. National Institutes of Health (NIH) consensus definition inclusive of lateral‐flow lipoarabinomannan assay.

**Results**: During July 2023 through October 2025, we evaluated 948 CWH (median age 6 years, range 0−9); 90 met NIH consensus criteria for TB. With the expanded criteria applied to TDA‐A, sensitivity was 88.9% (95% confidence interval [CI]: 80.5–94.5) and specificity was 72.0% (95% CI: 66.9–76.8) (Figures 1 and 2). The expanded criteria applied to TDA‐B demonstrated a lower sensitivity of 80.0% (95% CI: 70.2–87.7) but a higher specificity of 91.7% (95% CI: 88.2–94.4). When TDA entry criteria was limited to participants with standard TB symptoms, diagnostic yield was 46.7% (42/90) for TDA‐A and 40% (36/90) for TDA‐B, compared to 88.9% (80/90) for TDA‐A and 74.4% (67/90) for TDA‐B when using the expanded, symptom‐agnostic entry criteria.

**Conclusions**: Reliance on standard, symptom‐based entry criteria for TDAs failed to identify over half of CWH meeting the NIH consensus definition for confirmed or unconfirmed TB, compared to only 11% using the symptom‐agnostic criteria. These findings suggest that performance of TDAs in CWH may be improved by expanding entry criteria to include symptom‐agnostic screening tools, which may be a promising adjunct to close the TB diagnostic gap and end TB.

**FIGURE 1:**
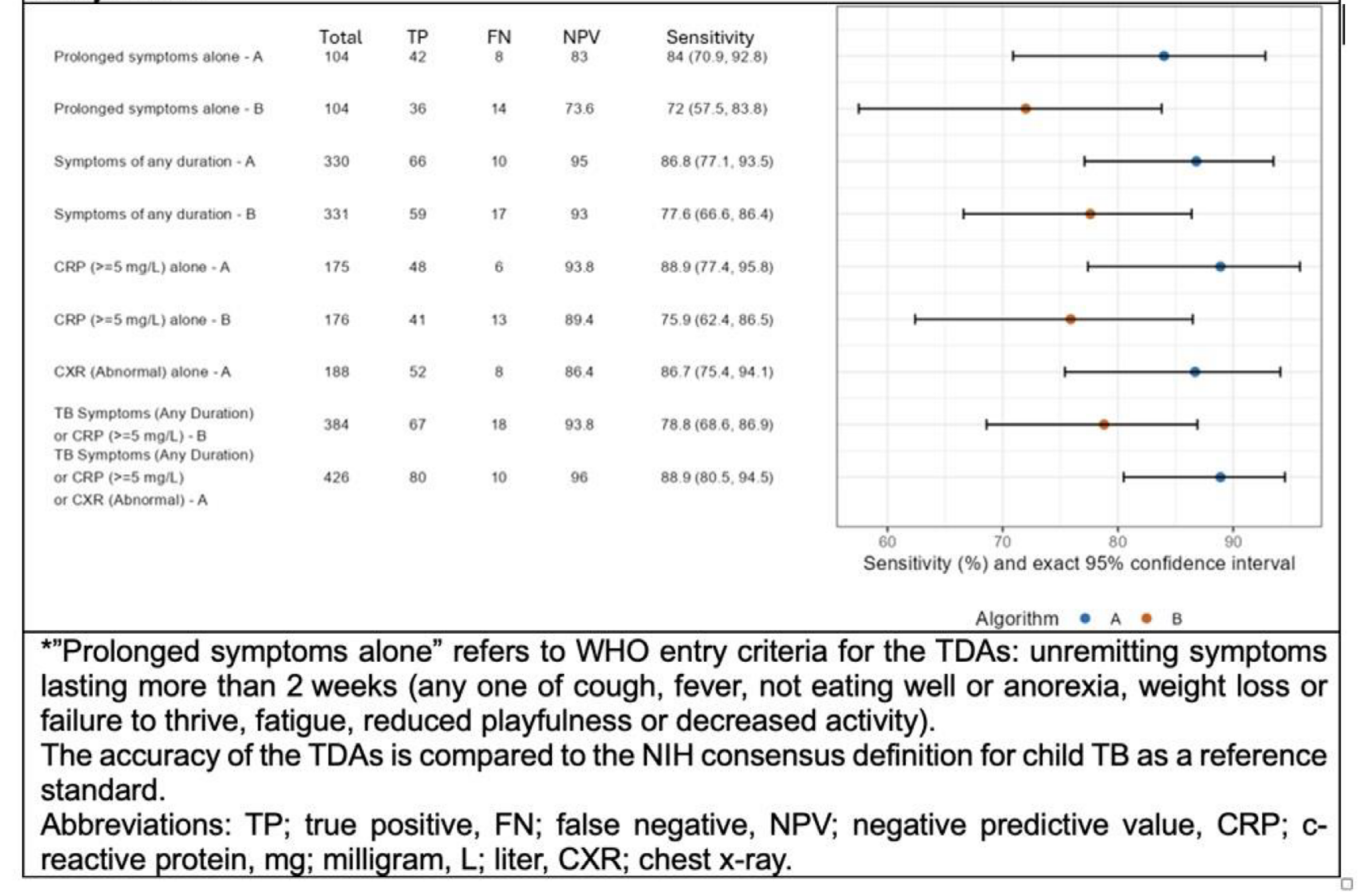
OAB4104 | Diagnostic accuracy and forest plots demonstrating the sensitivity of the TDAs among CWH enrolled in a screening and diagnostic cohort in Eswatini, Lesotho, Malawi, Tanzania and Uganda from July 2023 to October 2025 using symptom dependent and symptom agnostic TDA entry criteria.

**FIGURE 2:**
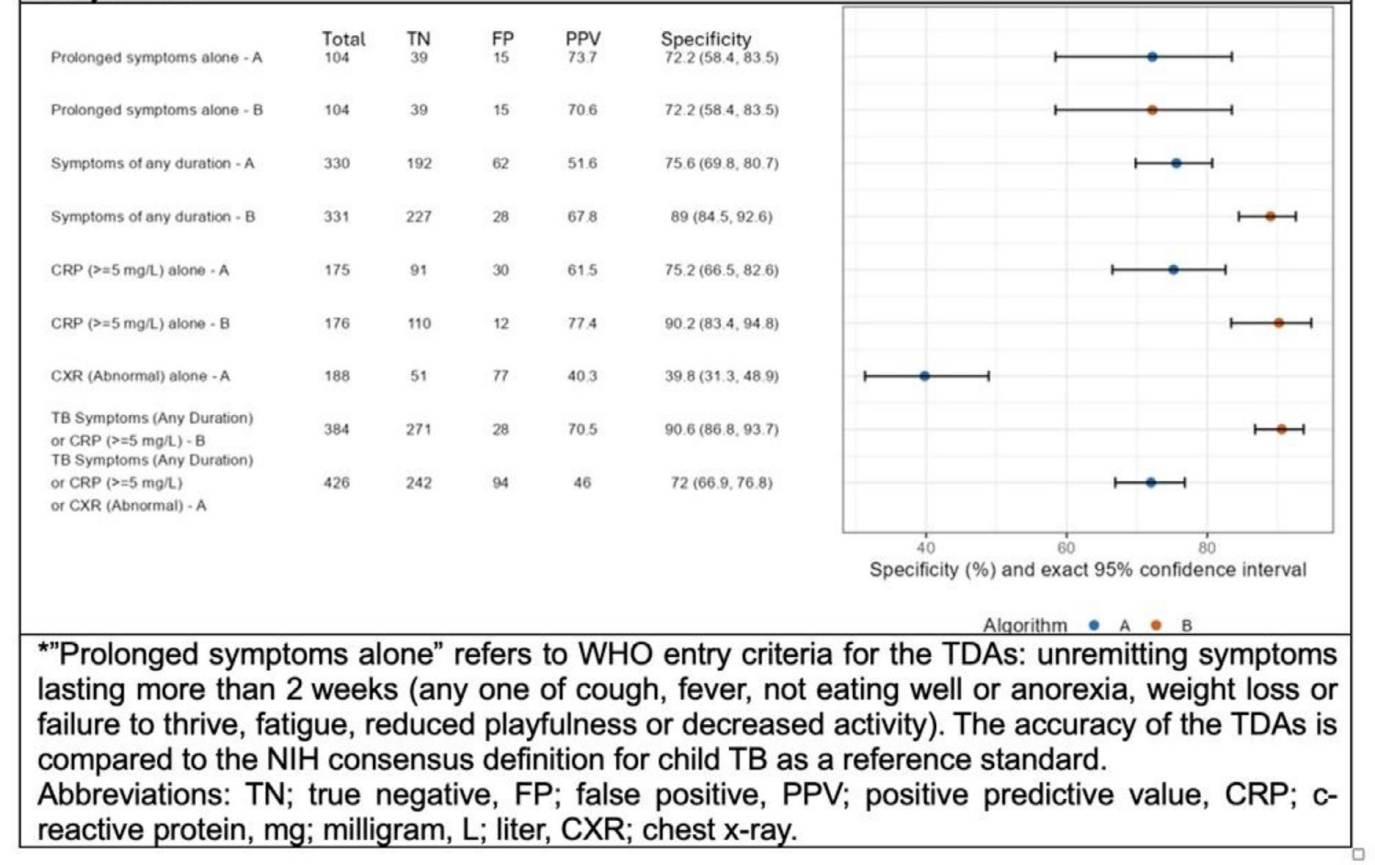
OAB4104 | Diagnostic accuracy and forest plots demonstrating the specificity of the TDAs among CWH enrolled in a screening and diagnostic cohort in Eswatini, Lesotho, Malawi, Tanzania and Uganda from July 2023 to October 2025 using symptom dependent and symptom agnostic TDA entry criteria.

## OAB4105 TB Relapse and Post‐Treatment Mortality Among People Living With HIV and TB Completing Anti‐Tuberculosis Treatment in Mumbai

A. Shinde^1^, S. Ughade ^2^, V. Karanjkar^3^, A. Afre^1^, A. Palkar^4^

^1^Mumbai Districts AIDS Control Society, Strategic Information, Mumbai, India; ^2^Mumbai Districts AIDS Control Society, Project Directorate, Mumbai, India; ^3^Mumbai Districts AIDS Control Society, Additional Project Director, Mumbai, India; ^4^UW International Training and Education Centre for Health Private Limited, Strategic Information, Mumbai, India

**Background**: Tuberculosis (TB) relapse is a critical yet under‐examined determinant of mortality among people living with HIV who complete anti‐tuberculosis treatment (ATT). While treatment completion is considered a programmatic success, recurrent TB episodes may signal persistent immunosuppression, suboptimal ART response or structural barriers to continuity of care. This study examined the association between TB relapse and post‐ATT mortality among people living with HIV and TB in Mumbai, with a focus on identifying clinical predictors of survival.

**Methods**: A retrospective cohort analysis was conducted using programmatic data from HIV–TB registers, ART Master Line Lists and the Nikshay TB surveillance system. People living with HIV and TB who completed ATT in 2018 and had documented outcomes were included. TB relapse status following ATT completion was the primary exposure variable. Survival outcomes were assessed using Kaplan–Meier survival analysis. Cox proportional hazards regression was applied to estimate unadjusted and adjusted hazard ratios (HRs) for mortality, controlling for age, sex, residence, CD4 count, viral load status and CD4 change. Statistical significance was set at *p* < 0.05.

**Results**: Among 815 people living with HIV and TB who completed ATT, 21.6% experienced TB relapse during follow‐up. Mortality among individuals with TB relapse was more than double that of those without relapse (32.4% vs. 15.2%; *p* < 0.0001). Kaplan–Meier analysis demonstrated significantly lower survival probability among relapsed individuals. In multivariable Cox regression, TB relapse was independently associated with increased mortality risk (adjusted HR: 1.39; 95% CI: 1.13–1.71; *p* = 0.002). Advanced immunosuppression (latest CD4 ≤200 cells/mm^3^) further amplified mortality risk, while a positive CD4 change following ATT completion was protective (adjusted HR: 0.69; *p* = 0.003).

**Conclusions**: TB relapse is a strong and independent predictor of post‐ATT mortality among people living with HIV and TB. These findings highlight the need to reframe TB treatment completion as a transition point rather than an endpoint of care. Intensified post‐ATT surveillance, early identification of relapse and strengthened ART optimization are essential to reduce preventable deaths among populations living with HIV and TB in urban settings.

## OAC0202 Intimate Partner Violence, Food Insecurity and HIV Incidence Among Women Aged 15–49 Years in Wakiso and Hoima, Uganda: A Population‐Based Longitudinal Cohort Study (2018−2023)

A. Daama^1,2,3^, S. G. Goldenberg^2^, S. Mugamba^1^, R. Bulamba^1^, D. Tuhebwe^2^, J. Nkale^1^, E. Kyasanku^1^, J. Nakakande^1^, F. Nalugoda^1^, G. Nakigozi^1^, S. Watya^1^, S. Kiene^2^, G. Kigozi^1^, A. P. Miller ^2^

^1^Africa Medical and Behavioral Sciences Organization, Kampala, Uganda; ^2^San Diego State University, School of Public Heath, San Diego, United States; ^3^University of California, San Diego, School of Public Health, San Diego, United States

**Background**: Sub‐Saharan Africa accounts for nearly two‐thirds of global HIV incidence, with women disproportionately affected. Despite Uganda's progress in HIV prevention, recent community‐based estimates of HIV incidence remain scarce. Prior research has largely emphasized individual‐level factors, often overlooking interpersonal, community and structural determinants of HIV incidence. We examined multilevel determinants of HIV incidence among women in two Ugandan districts.

**Methods**: We analysed longitudinal data from the Africa Medical and Behavioral Sciences Organization (AMBSO) Population Health Surveillance (APHS) cohort collected between 2018 and 2023. Participants were self‐identified women aged 15–49 years who were HIV seronegative at baseline. Person‐time was calculated from baseline to follow‐up HIV testing to estimate incidence per 100 person‐years (PY). Poisson regression models were used to compute incidence rate ratios (IRR) and 95% confidence intervals (CI) for primary exposures: intimate partner violence (IPV) in the past 12 months, district of residence, food insecurity and water insecurity in the past 1 month.

**Results**: Among participants (*N* = 3603), median age was 29 years [IQR 23−37]. Overall, 9.3% experienced IPV, food insecurity (72.2%), water insecurity (9.3%), Wakiso (44.4%) versus Hoima (55.6%). Over 54 seroconversions occurred over 3608 PY, yielding an incidence rate of 1.50/100 PY, with higher incidence observed in the Hoima district (1.61/100 PY) versus Wakiso (1.38/100 PY). In separate adjusted models, IPV was significantly and strongly associated with HIV incidence (adjusted rate ratio [ARR] = 3.25; 95% CI: 1.10−9.56), as was food insecurity (ARR = 3.43; 95% CI: 1.10−10.67). Both water insecurity and residing in Hoima (vs. Wakiso) were associated with a higher HIV incidence rate, though these associations were null (ARR = 1.56, 95% CI: 0.44−5.46) and (ARR = 1.01; 95% CI: 0.45−2.24, respectively).

**Conclusions**: Despite declining HIV incidence in Uganda, persistent interpersonal and structural vulnerabilities faced by women, including those related to both gendered violence and food insecurity, may act as critical drivers of new infections among women. IPV and food insecurity substantially elevate the vulnerability to HIV acquisition among women, underscoring the need for integrated prevention strategies addressing gender‐based violence and resource insecurity.

## OAC0203 Water Insecurity as a Structural Determinant of HIV Treatment Outcomes Among People With HIV in Kisumu County, Western Kenya

J. Odak ^1^, L. Ndunyu^1^, P. Omemo^1^, B. Rono^2^, E. A. Bukusi^2^, C. R. Cohen^3^, S. D. Weiser^4^

^1^Maseno University, School of Public Health & Community Development, Kisumu, Kenya; ^2^Kenya Medical Research Institute, Centre for Microbiology Research, Kisumu, Kenya; ^3^University of California San Francisco, Department of Obstetrics, Gynecology & Reproductive Sciences, San Francisco, United States; ^4^University of California San Francisco, Division of HIV, Infectious Diseases and Global Medicine, San Francisco, United States

**Background**: Reliable access to safe water is essential for sustaining daily HIV treatment routines, yet water insecurity remains an underexplored structural barrier to sustained HIV care engagement in high‐burden settings. In Kisumu County, little is known about the role of water insecurity in HIV treatment interruptions, or whether treatment outcomes differ by sex. We estimated the association between water insecurity and HIV treatment outcomes among people with HIV (PWH), with secondary analyses by sex.

**Methods**: We conducted a case‐control study (October 2024–March 2025) among 356 PWH (15−64 years). Cases had a composite HIV treatment outcome (viral load ≥200 copies/mL, CD4 <250 cells/mm^3^, opportunistic infections, missed clinic visits). Controls had none of these outcomes. We matched cases and controls on age, sex and time on ART, though matching was not perfect. We measured water insecurity using the 12‐item Individual Water Insecurity Experiences Scale. We used multivariable logistic regression to estimate the association between binary water insecurity and HIV treatment outcomes, adjusting for age, sex, time on ART, marital status, education, employment, housing and clinic site, and assessed effect modification by sex using an interaction term. We examined the severity of water insecurity using unadjusted predicted probabilities across four categories to support interpretation.

**Results**: Overall, the prevalence of water insecurity was 71%, higher among cases (82%) than controls (59%). After adjustment, water insecurity was strongly associated with poor HIV treatment outcomes (AOR: 5.0; 95% CI: 2.18–11.44; *p* < 0.001). When examined by severity, unadjusted predicted probabilities of poor HIV treatment outcomes increased sharply with worsening water insecurity, from 12% among participants with no to marginal water insecurity to nearly 90% among those experiencing high water insecurity. Differences across severity levels were statistically significant (*p* < 0.001). Predicted probabilities increased for both men and women, with no statistical evidence that sex modified the association.

**Conclusions**: Water insecurity is a structural determinant strongly associated with HIV treatment outcomes among PWH in Kisumu County, with no evidence of sex differences in the association. These findings highlight the need for policies and interventions that integrate reliable water access into HIV care to prevent viral rebound and improve outcomes in resource‐constrained, climate‐vulnerable settings.

## OAC0204 Early Impact of Funding Withdrawal on HIV Care and Treatment Programmes in East Africa

H.‐H. M. Truong ^1^, A. Semeere^2^, C. T. Ylannoutsos^3^, B. Musick^4^, K. Wools‐Kaloustian^4^, J. Lewis‐Kulzer^1^, F. Odhiambo^5^, L. Diero^6^, C. Kasozi^7^, D. Michael Mkwashapi^8^, C. R. Cohen^1^

^1^University of California San Francisco, San Francisco, United States; ^2^Infectious Diseases Institute, Kampala, Uganda; ^3^City University of New York, New York, United States; ^4^Indiana University, Indianapolis, United States; ^5^Kenya Medical Research Institute, Kisumu, Kenya; ^6^Moi University, Eldoret, Kenya; ^7^Masaka Regional Referral Hospital, Masaka, Uganda; ^8^National Institute for Medical Research, Mwanza, the United Republic of Tanzania

**Background**: The US government withdrew funding from HIV treatment and prevention programmes globally in January 2025. We assessed the early impact of funding loss on services and patient outcomes in the East Africa regional consortium of the International Epidemiology Database to Evaluate AIDS (EA‐IeDEA), a network of nine HIV care and treatment programmes supported by US government agencies.

**Methods**: We analysed programme data from 189 clinics in Kenya, Uganda and Tanzania, focusing on the number of new patients enrolled and viral load tests conducted. All patients with at least one completed visit or a visit scheduled during prior encounters in the period 25 January to 28 February 2024 and 2025 were included in this cohort. Viral load <400 copies/mL was considered suppressed. Frequencies, Kruskal−Wallis and chi‐square tests were used to describe changes year‐over‐year between 2024 and 2025.

**Results**: Our database included 227,328 encounters from 102,990 distinct patients. New enrolments declined by 59% (2811−1153) in 2025 compared to the same period in 2024. The decline was greater among men than women (64% vs. 56%; *p* = 0.003) and those aged <25 than ≥25 years (69% vs. 51%; *p* < 0.001). There was a 33% decline in the number of viral load tests conducted, decreasing from 16,181 to 10,872 tests year‐over‐year. The decline was similar among women and men (34% vs. 31%; *p* = 0.124) but was much higher in those aged <25 than ≥25 years (39% vs. 32%; *p* < 0.001). Viral load was suppressed in 91% versus 88% of tests performed in the relevant periods. No difference was observed in the median number of days between scheduled and actual visits in 2025 compared to 2024 (*p* = 0.744).

**Conclusions**: While HIV care and treatment programmes maintained services for existing patients in the period studied, the numbers of new patients enrolled and viral load tests conducted declined in the first month after funding withdrawal in 2025 compared to the same period in 2024, which more greatly impacted younger individuals. Further evaluations are needed to understand how changes in the region's funding model affect clinical services and long‐term outcomes.

## OAC0205 Rethinking HIV Prevention Prioritization During Funding Crisis: Evidence From Rapid Service Declines Among Key Populations in Nigeria

R. Aliyu Magaji ^1^, F. Agbo^1^, L. Kwaghga^1^, F. Idepefo^1^, J. E. Egwu^1^, K. Murtala Safana^1^

^1^National Agency for the Control of AIDS (NACA), Research, Monitoring and Evaluation, Abuja, Nigeria

**Background**: Recent advances in HIV prevention, including pre‐exposure prophylaxis (PrEP), have expanded options for reducing new infections. However, prevention service delivery remains vulnerable to funding and policy shifts. In early 2025, Nigeria experienced a major donor funding disruption alongside a policy re‐prioritization of PrEP delivery towards pregnant women, creating a natural experiment to assess implications for key population prevention services.

**Methods**: We conducted a quarter‐on‐quarter analysis of national routine HIV programme data, comparing Q1 and Q2 2025. Indicators included KP prevention reach, HIV testing services (HTS), PrEP initiation among KPs and condom distribution, disaggregated by KP typology.

**Results**: Between Q1 and Q2 2025, the number of key populations reached with prevention services declined by 50.5% (70,414−34,819), while HTS uptake fell by 70.2% (115,411−34,389). PrEP initiation among key populations decreased by 83% (37,442−6326), signalling a rapid re‐prioritization of prevention access away from populations at highest risk. Declines were most pronounced among sex workers, men who have sex with men and people who inject drugs. Male condom distribution decreased by 36% over the same period.

**Conclusions**: These findings demonstrate how quickly HIV prevention access can be reshaped by funding shocks and policy re‐prioritization. While expanding PrEP for pregnant women strengthens maternal and infant outcomes, the concurrent reduction in prevention access for key populations risks widening HIV acquisition gaps. Rethinking prevention in an era of constrained resources requires explicit equity safeguards to ensure that populations at highest risk are not systematically deprioritized.

## OAC0702 Beyond Barriers: Delivering Comprehensive Cervical Cancer Screening and Treatment for Women Living With HIV Via a Remote Static–Outreach Model in Mashonaland Central, Zimbabwe (October 2023–September 2024)

S. Murwira ^1^, M. R. Gadzayi^1^, B. Mushangwe^1^, G. Gonese^1^, B. Makunike‐Chikwinya^1^, B. Moyo^2^, J. Mandisarira^2^, C. Mapfumo^3^

^1^Zimbabwe Technical Assistance, Training and Education Centre for Health, Care and Treatment, Harare, Zimbabwe; ^2^Centers for Disease Control and Prevention (CDC), Division of Global HIV and TB (DGHT), Harare, Zimbabwe; ^3^Ministry of Health and Child Care, Harare, Zimbabwe

**Background**: Cervical cancer remains a leading cause of mortality among women living with HIV (WLHIV) in Zimbabwe, with low screening coverage in rural areas. A combined static and outreach model has been implemented by nurses nationwide since 2018 to improve access. This analysis describes outcomes from a high‐performing static site and nine outreach points, illustrating how comprehensive screening, timely treatment and follow‐up can be delivered efficiently in resource‐limited, hard‐to‐reach settings, providing lessons for scalable programmatic impact.

**Description**: Since 2018, Zim‐TTECH has supported cervical cancer screening and treatment for WLHIV across Zimbabwe, establishing services at 71 static clinics and 325 outreach sites by 2024. Screening is primarily conducted using visual inspection with acetic acid and cervicography (VIAC), with HPV testing gradually being introduced. Kamutsenzere Clinic, recognized for high performance in HIV and cervical cancer services, and its nine hard‐to‐reach outreach points were selected to evaluate programme delivery. From October 2023 through September 2024, WLHIV aged 25–49 years were offered VIAC screening, same‐day thermal ablation for eligible women and referral for loop electrosurgical excision procedure (LEEP). Cervical cancer services were integrated with ART refills and viral load monitoring. Screening result, treatment type and date of treatment were documented in registers.

**Lessons Learned**: All eligible WLHIV (*n* = 1164) were screened, 72% (837) during outreach. Overall, 103 (8.8%) were VIAC positive, with 65 (63.1%) eligible for ablation and 38 (36.9%) eligible for LEEP. In total, 100 (97.1%) received treatment: 67 thermal ablations and 35 LEEPs, three (2.9%) declined treatment to use traditional medicine. Same‐day thermal ablation was completed for 66 (98.5%) of WLHIV. Most thermal ablations (51; 76.1%) occurred during outreach, demonstrating the feasibility of decentralization. LEEP procedures were performed a median of 32 days after diagnosis and referral. Remote sites showed high performance in delivering cervical cancer services, with outreach expanding access and same‐day treatment improving coverage.

**Conclusions/Next Steps**: A remote, nurse‐led static and outreach model can be effective in delivering comprehensive cervical cancer care and can achieve high coverage and timely treatment among WLHIV. Efficient same‐day thermal ablation and coordinated LEEP referral demonstrate feasible, scalable strategies for strengthening cervical cancer prevention and care in hard‐to‐reach, resource‐limited settings.

## OAC0703 Feasibility and Outcomes of an Integrated High‐Risk Human Papillomavirus Self‐Sampling and Screen‐and‐Treat Model for Cervical Cancer Prevention Among Fisherfolk Women in Migori County, Kenya

R. Oyuga ^1^, E. Omari^1^, D. Odhiambo^1^, P. Apamo^1^, J. Ombagi^1^, A. Nyabiage^1^, E. Owino^2^, S. Wafula^1^, E. Koech^1^, N. Owuor^1^

^1^Center for International Health, Education and Biosecurity, Nairobi, Kenya; ^2^Ministry of Health, Migori County, Kenya

**Background**: The World Health Organization (WHO) targets elimination of cervical cancer by 2030 through 90% HPV vaccination, 70% screening and 90% treatment of precancerous lesions. Achieving this requires innovative approaches for underserved, high‐burden groups. Fisherfolk women in Migori County face high HIV prevalence, frequent mobility and poor access to cervical cancer screening. We evaluated high‐risk human papillomavirus (hrHPV) prevalence and tested the feasibility of a self‐sampling, screen‐and‐treat model among fisherfolk women in Migori county, Kenya, in the period September 2024−September 2025.

**Methods**: We conducted a stepped‐wedge, mixed‐methods study among fisherfolk women aged 30–49 years in Fisherfolk communities. Participants underwent hrHPV testing on the Cobas (R) 6800 Analyser using self‐ or clinician‐collected samples. hrHPV‐positive women underwent visual inspection with acetic acid (VIA) and offered same‐day thermocoagulation. Sociodemographic and clinical factors associated with hrHPV positivity were explored using bivariate analysis.

**Results**: Among 877 women of median age 35 (31,41) screened, 73% (640) preferred self‐sample collection, and hrHPV prevalence was 23.1% (201). Prevalence was higher in women aged 30–35 years (75/250, 30.0%) compared with 41–49 years (53/313, 16.9%). After adjusting for age, marital status, women with more births were at lower risk of HPV, with each increase in parity associated with a 12% decrease in positivity (OR: 0.88 [0.8, 0.98], *p* = 0.018). HIV‐positive women had greater hrHPV prevalence (70/258, 27.1%) and with a 67% chance to test positive (OR: 1.67, [1.1−2.53], *p* = 0.016) compared with HIV‐negative women (131/593, 22.1%). Of hrHPV‐positive women, 167/201 (83.1%) returned for VIA triage, and 91/93 (97.8%) VIA‐positive women were treated, all with thermocoagulation. Suspected cervical cancer was identified in 2/201 (1.0%) women and were referred for care.

**Conclusions**: Fisherfolk women in Migori carry a high hrHPV burden, especially younger and HIV‐positive women. Self‐collection was acceptable and improved detection, making it suitable for scale‐up. Integrating hrHPV testing with self‐collection, VIA and same‐day treatment achieved strong uptake, showing feasibility for expansion in resource‐limited settings like Kenya and fast‐tracking efforts towards WHO elimination goals.

## OAC0704 Rising Burden of Congenital Syphilis in South Africa: Trends From 2020 to 2023: Opportunities to Optimize Implementation of Dual HIV/Syphilis in Pregnant Women and Their Sexual Partners

A. Marsh ^1^, T. Maomela^2^, L. Lebese^3^, T. Chidarikire^4^

^1^Ministry of Health South Africa, HIV Prevention, Pretoria, South Africa; ^2^Ministry of Health South Africa, Monitoring and Evaluation, Pretoria, South Africa; ^3^Ministry of Health South Africa, Vertical Transmission Prevention, Pretoria, South Africa; ^4^World Health Organization, HIV Prevention, Pretoria, South Africa

**Background**: Congenital syphilis (CS) is a severe but preventable condition caused by vertical transmission of Treponema pallidum during pregnancy. Despite the availability of cost‐effective interventions such as antenatal screening and timely treatment, CS continues to pose a major public health challenge globally and in South Africa. The World Health Organization (WHO) has set an elimination target of fewer than 50 cases per 100,000 live births, emphasizing early antenatal care, universal screening and treatment completion. Understanding recent epidemiological trends is critical for guiding policy and strengthening maternal health programmes.

**Methods**: We conducted a retrospective analysis of national surveillance data from the National Institute for Communicable Diseases (NICD) for the period 2020–2023. Data included annual congenital syphilis incidence rates per 100,000 live births, total reported cases and live birth figures. Trends were assessed to identify changes over time and compare them against WHO elimination targets.

**Results**: CS incidence increased dramatically over 4 years, from 37 per 100,000 live births in 2020 to 198 per 100,000 in 2023. Reported cases rose from 382 in 2020 to 1739 in 2023, while live births declined from 1,035,255 to 876,972. These findings highlight a growing burden of CS despite ongoing elimination strategies and underscore the urgent need for strengthened antenatal screening, timely treatment and robust surveillance systems. The data suggests that without intensified interventions, South Africa will remain far from achieving the WHO elimination target of fewer than 50 cases per 100,000 live births as it represents a more than a fivefold increase in incidence despite ongoing elimination strategies.

**Conclusions**: Urgent action is required to strengthen maternal health services and achieve elimination targets. It would, therefore, be critical for South Africa to implement the dual HIV/syphilis modality routinely in ANC clinics in all nine provinces to reduce the number of CS in the country.

## OAC0705 Integrated Community‐Based HIV and Viral Hepatitis Testing Is Effective in Identifying Undiagnosed Acquisitions in the General Population in Pakistan

M. S. Jamil ^1^, A. Ali^2^, K. Mustafa^3^, A. A. Soomro^4^, S. A. Shah^4^, Z. Dharejo^3^, F. Hafeez^5^, M. S. Pasha^2^

^1^World Health Organization Regional Office for the Eastern Mediterranean, Department of Health Promotion and Disease Prevention and Control, Cairo, Egypt; ^2^World Health Organization Country Office Pakistan, Islamabad, Pakistan; ^3^CDC‐HIV/AIDS, Karachi, Pakistan; ^4^Bridge Consultants Foundation, Karachi, Pakistan; ^5^Common Management Unit for HIV, TB and Malaria, Islamabad, Pakistan

**Background**: Pakistan is facing a high burden of viral hepatitis and a rising burden of HIV, including sporadic nosocomial outbreaks. In the context of response to a cluster of HIV in the general population in Hyderabad district of Sindh Province, we integrated hepatitis testing in a community‐based HIV testing programme in affected areas. Here, we present the positivity and co‐infection rates.

**Methods**: Door‐to‐door testing was conducted in partnership with national and provincial authorities, focusing on selected areas that reported an increase in HIV diagnoses. Those aged up to 55 years were offered testing, while those who self‐reported HIV test in the past 6 months or were already on ART were excluded. Forty‐nine mobile teams of one trained male and female mobilizer each offered testing using rapid tests (HIV Antibody “Abbott Early Detect”; hepatitis B [HBV] antigen “Abbott Determine”; hepatitis C [HCV] antibody “Abbott Bioline”). Those with reactive results were referred to nearest treatment facilities for confirmatory testing, clinical assessment and treatment initiation. Information, education and communication materials related to risk factors were displayed in health facilities and in the community. We report positivity and co‐infection rates based on single rapid test results, disaggregated by sex and age group (children: <15 years).

**Results**: Between 23 October and 6 November, 2025, 27,645 persons (58% females; 39% children) were tested for HIV, HBV and HCV. Overall, 58 individuals (0.21%) were HIV‐positive, 118 (0.43%) were HBV‐positive and 1468 (5.34%) were HCV‐positive. Co‐infection rates were as follows: HIV‐HBV = 0.01%; HIV‐HCV = 0.05%; HBV‐HCV = 0.05%; HIV‐HBV‐HCV = 0.01%. Disaggregation by age and sex is presented in Table 1. In this setting, for each HIV‐positive person, two HBV‐positive and 25 HCV‐positive persons were identified. If every HCV‐positive person is offered HIV and HBV testing, one HIV‐positive and one HBV‐positive will be identified for every 105 HCV‐positive persons.

**Conclusions**: In this setting, integrating hepatitis testing in a community‐based HIV testing programme was effective in identifying people with HIV and hepatitis. These results provide strong justification for scaling‐up integrated HIV and hepatitis testing. If resources are limited, testing every HCV‐positive person for HIV and HBV can be a cost‐effective approach for case finding.

**TABLE 1:** OAC0705 | HIV, HBV and HCV positivity and co‐infection rates from single rapid test result among persons tested (*n* = 27,465).

	Sex**		Age group		
Results category Single infection positivity*	Male (*n* = 11,510) *n* (%)	Female (*n* = 15,954) *n* (%)	Adult (*n* = 16,630) *n* (%)	Child (*n* = 10,835) *n* (%)	Total *n* (%)
HIV+	27 (0.23)	31 (0.19)	40 (0.24)	18 (0.17)	58 (0.21)
HBV+	61 (0.53)	57 (0.36)	114 (0.69)	4 (0.04)	118 (0.43)
HCV+	557 (4.48)	911 (5.71)	1448 (8.71)	20 (0.18)	1468 (5.34)
Co‐infection positivity*					
HIV+ & HBV+	1 (0.01)	3 (0.02)	4 (0.02)	0 (0)	4 (0.01)
HIV+ & HCV+	6 (0.05)	8 (0.05)	14 (0.08)	0 (0)	14 (0.05)
HBV+ & HCV+	6 (0.05)	8 (0.05)	14 (0.08)	0 (0)	14 (0.05)
HIV+, HBV+ & HCV+	0 (0)	3 (0.02)	3 (0.02)	0 (0)	3 (0.01)

*Categories not mutually exclusive.

**1 TG.

## OAC1302 Equity Gaps in Postpartum Viral Suppression: A National Analysis of Pregnant Women Living With HIV or AIDS in Brazil

A. Krummenauer, A. Paula Betaressi da Silva, N. Mendonça Collaço Véras, I. Ornelas Pereira, T. Benoliel Rocha, R. do Socorro Marques de Oliveira, L. Henriette de Lannoy, A. Tiago Bernardo de Matos, P. Cristina Gaspar, A. Roberta Pati Pascom

Brazilian Ministry of Health, Department of HIV/AIDS, Tuberculosis, Viral Hepatitis and Sexually Transmitted Infections, Brasília, Brazil

**Background**: Brazil has eliminated perinatal transmission of HIV as a public health problem through testing, universal antiretroviral therapy access and clinical monitoring of pregnant women living with HIV or AIDS (PWLHA). Sustaining this milestone requires assessing whether viral suppression is equitably achieved and maintained postpartum. This study examines viral suppression trajectories through 24 months postpartum and identifies sociodemographic disparities in virologic outcomes.

**Methods**: National cohort study of PWLHA identified through probabilistic linkage of Brazilian health information systems (mortality, antiretroviral dispensing, laboratory and notification databases; 2015−2025). We analysed viral suppression (<50 copies/mL) among PWLHA documented as receiving antiretroviral therapy at delivery, 6, 12, 18 and 24 months postpartum, using the closest viral load (VL) measurement (±180‐day windows). Intersectional analyses included region, race or ethnicity, education and age.

**Results**: We identified 94,690 PWLHA. Viral suppression at delivery increased from 59.0% in 2015 to 76.9% in 2025. However, all sociodemographic groups experienced a decline postpartum, and inequalities observed at delivery persisted throughout follow‐up. Suppression was higher in the South (70.8%) and Southeast (68.7%) than in the North (58.8%) and Northeast (59.0%). White and Asian women achieved higher suppression (69.9%) than Black (65.2%), Mixed‐Race (63.7%) and Indigenous women (61.0%). Suppression ranged from 55.4% among adolescents (<20 years) to 73.5% among women aged ≥40 years, and from 61.7% among women with 0–7 years of schooling to 68.0% among those with ≥8 years. Intersectional analyses showed the lowest suppression among adolescents with low educational attainment (52.4%), while White women aged ≥40 years with intermediate or higher education exceeded 75%. Postpartum viral rebound was most pronounced among younger women with lower education, regardless of region or race.

**Conclusions**: Although viral suppression improved substantially in Brazil over the last decade, it was not achieved or sustained uniformly across populations. Adolescents, women with low education, Black women, and residents of the North and Northeast experienced the lowest rates of viral suppression, particularly where age and education overlapped. The expanding equity gap signals that sustaining postpartum suppression requires structural, equity‐focused interventions—a key challenge to maintain elimination of perinatal transmission (see Figure 1).

**FIGURE 1:**
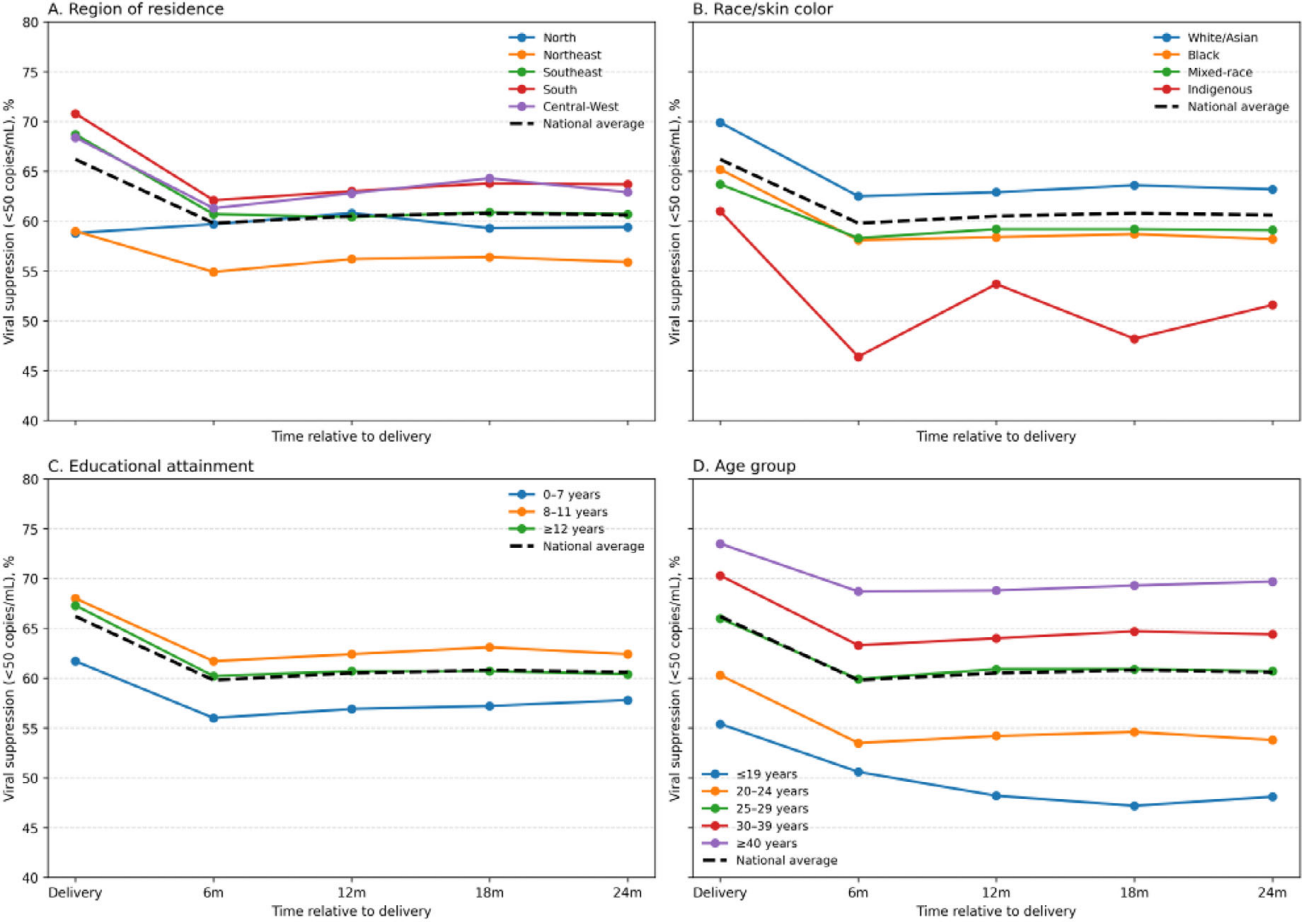
OAC1302

## OAC1303 Barriers to Eliminating Vertical HIV Transmission Identified Through Audits of Infants Acquiring HIV in Uganda, 2021−2025

L. K. Nabitaka ^1^, N. Buono^2^, D. Idipo^3^, N. Maina^4^, T. Nsubuga Nyombi^5^

^1^Ministry of Health, National Disease Control and Prevention, Mukono, Uganda; ^2^Independent Consultant, MD, Washington, United States; ^3^Ministry of Health, National Disease Control and Prevention, Kampala, Uganda; ^4^Independent Consultant, Nairobi, Kenya; ^5^Independent Consultant, Kampala, Uganda

**Background**: Eliminating vertical HIV transmission is a national priority for Uganda. Understanding missed prevention opportunities among infants acquiring HIV can inform targeted strategies. The Ministry of Health conducts routine clinical audits of infants acquiring HIV vertically as a quality improvement approach to identify programmatic gaps and strengthen efforts to achieve Uganda's vertical HIV elimination goals.

**Methods**: We conducted a retrospective audit of HIV exposed infants who acquired HIV between July 2021 and September 2025 across 47 health facilities in 12 regions of Uganda. Maternal and infant data were abstracted from health facility registers and DHIS2, including maternal timing of HIV diagnosis; viral load (VL) monitoring and suppression; partner testing, infant postnatal prophylaxis; and infant antiretroviral (ART) initiation. De‐identified data were compiled, cleaned, and de‐duplicated by outcome and analysed using R (tidyverse).

**Results**: Among 365 audited infants, 21.4% (78/365) of their mothers were first identified as living with HIV before pregnancy, 15.1% (55/365) during antenatal care, 1.6% (6/365) at labour & delivery, and 43.3% (158/365) during breastfeeding; timing was undocumented in 18.6% (68/365). Maternal VL results were available for 37% of mothers, of whom 29% had unsuppressed VL. Infant postnatal prophylaxis was documented for 51.2% of infants, undocumented for 45.8% and missing for 3.0%. A high proportion of mothers did not know their partner's HIV status (70.7%). Overall, 89.6% of infants initiated ART. Among infants not on ART (*n* = 29), recorded reasons included death (41.4%), transfer out (27.6%), loss to follow‐up (20.7%), refusal (3.4%) and unknown reasons (6.9%).

**Conclusions**: Late maternal diagnosis, limited access to maternal VL monitoring and suppression, unknown partner HIV status, and gaps in infant prophylaxis and documentation are key drivers of ongoing vertical HIV transmission in Uganda. Audits of infants acquiring HIV provide actionable insights to prioritize early and repeat maternal testing, partner testing, strengthen maternal VL access and suppression, improve documentation, and implement targeted support for mother−infant pairs and accelerate elimination of vertical HIV transmission (see Figure 1).

**FIGURE 1:**
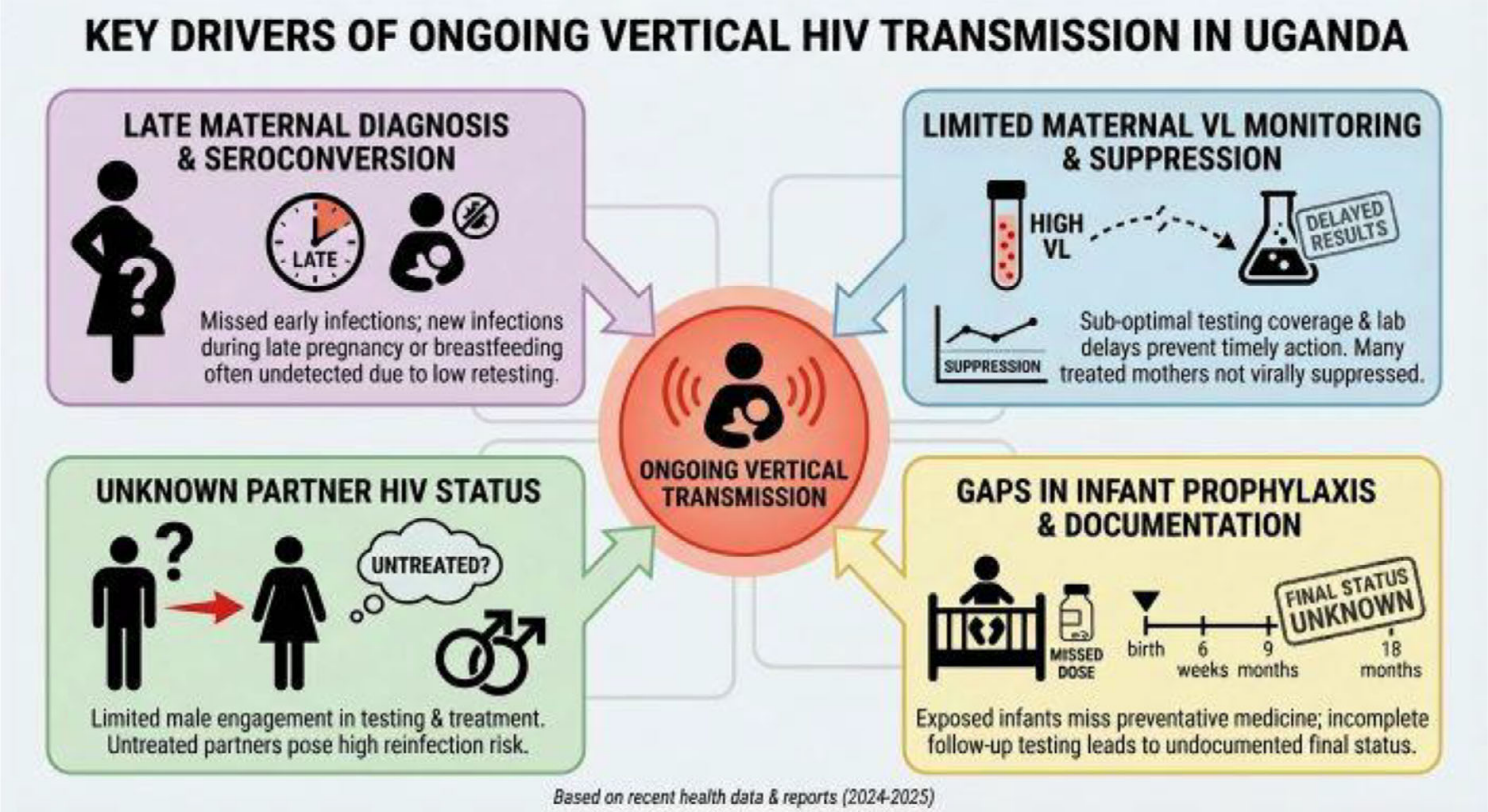
OAC1303

## OAC1304 Closing Vertical Transmission Prevention Gaps Through Community‐Based ANC Services: Outcomes From Traditional Birth Attendants and Faith‐Based Delivery in Settings in Northwest Nigeria

V. Ajuka‐Patrick ^1^, K. Olalekan Ayodeji^1^, E. Gideon^1^, J. Ogwu^1^, H. Bello Raji^1^, J. Awotoye^1^, E. Okpokoro^1^, O. Adebayo^1^, A. Adekunle^1^, O. Olupitan^1^, A. Agbaje^1^, C. Mensah^1^, P. Dakum^1^, A. Abimuku^1^, A. Bashorun^2^, M. Zamzu^3^

^1^Institute of Human Virology Nigeria (IHVN), Abuja, Nigeria; ^2^National AIDS and STI Control Program, Abuja, Nigeria; ^3^The Global Fund to Fight AIDS, Tuberculosis and Malaria, Global Health Campus, Geneva, Switzerland

**Background**: Pregnant women receiving antenatal and delivery care outside formal health facilities remain at high risk of missed HIV testing and delayed antiretroviral therapy (ART) initiation. Community delivery settings, including traditional birth attendants (TBAs), birth homes and faith‐based facilities, represent critical entry points for prevention of vertical transmission of HIV services. This analysis aimed to assess the effectiveness of a community‐based prevention of vertical transmission implementation model in expanding HIV testing coverage and improving linkage and timely ART initiation among pregnant women receiving care in non‐health facility settings in Northwest Nigeria.

**Methods**: A community‐based prevention of vertical transmission implementation model was deployed across TBAs, birth homes and faith‐based facilities in Kano and Katsina. Routine programme data between January 2024 and October 2025 was extracted, R software was used to analyse standard prevention of vertical transmission indicators, new antenatal care (ANC) enrolment, HIV testing and result delivery, HIV positivity yield, ART status at ANC entry and timing of ART initiation during pregnancy, labour, and the immediate postpartum period <72 h.

**Results**: A total of 166,341 pregnant women were newly enrolled for ANC in community settings during the reporting period. HIV testing with result delivery was achieved for 158,460(95.3%) women. Among those tested, 156 (0.1%) were identified as living with HIV. Of all pregnant women living with HIV, seven (4.5%) were already on ART before the current pregnancy. Among women newly diagnosed with HIV, ART initiation occurred at multiple points along the care sequence. 108 (69.2%) initiated ART during ANC before 36 weeks’ gestation, 40 (25.6%) during ANC after 36 weeks, 1 (0.6%) during labour and 1 (0.6%) within 72 h postpartum, and a total of 150 were linked to treatment. These findings demonstrate successful linkage and ART initiation even among late presenters traditionally missed by facility‐based prevention of vertical transmission programmes.

**Conclusions**: Integrating prevention of vertical transmission services into community delivery platforms significantly expands ANC enrolment, improves HIV testing coverage and enables timely ART initiation across pregnancy, labour and postpartum periods. Community‐based prevention of vertical transmission models are essential for reaching underserved populations and accelerating progress towards the elimination of vertical transmission of HIV.

## OAC1305 From 34% to 92%: Decentralizing Prevention of Vertical Transmission Through Community‐Led HIV Testing in India

K. Biswas, L. Rathore, R. Rana, M. Asif, S. Dasgupta

Plan International (India Chapter), Programme, Delhi, India

**Background**: While India has made significant progress in reversing the HIV epidemic, HIV testing coverage among pregnant women remained critically low in several states in 2015. Evidence indicated that only 34% of pregnant women in the 13 Plan India project states had access to HIV testing, despite an estimated 14 million pregnancies annually. Complementing the Government of India's National AIDS Control Programme (NACP) and EMTCT strategy, Plan India implemented the Global Fund–supported AHANA Project to expand HIV testing coverage and strengthen the PMTCT cascade.

**Description**: Three‐pronged strategy was adopted: (a) capacity building of over 40,000 community health workers across government health facilities to institutionalize routine HIV testing during ANC; (b) sustained national and sub‐national advocacy to ensure procurement and uninterrupted supply of HIV test kits and consumables; and (c) demand generation among pregnant women to promote early and repeated HIV testing during pregnancy.

**Lessons Learned**: HIV testing coverage among pregnant women increased from 34% in 2016–2017 to 92% in 2023–2024. The proportion of peripheral HIV testing rose from 25% to 80%, reflecting substantially improved access in rural and hard‐to‐reach areas. This expansion resulted in a 25% increase in identification of HIV‐positive pregnant women between 2016–2017 and 2023–2024. Over 30,000 HIV‐positive pregnant women were identified, linked to ART services, and followed up for care and support during 2016–2024. Early ART initiation during the ANC phase improved from 79% to 88%. Institutional delivery among HIV‐positive pregnant women increased from 90% to 93%. More than 16,000 infants were confirmed HIV‐negative after receiving definitive testing at 18 months contributing directly reducing the transmission by more than half from 25.28% in 2019 to 11.75% in 2023.

**Conclusions/Next Steps**: Capacity enhancement of peripheral public health units, combined with community‐driven HIV screening and strong supply‐side advocacy, resulted in a manifold increase in HIV testing among pregnant women. Testing services were decentralized from district hospitals to all Primary Health Centres (PHCs) and further to Village Health and Nutrition Days (VHNDs), effectively taking HIV testing to the doorstep of pregnant women and embedding PMTCT within routine maternal health services.

## OAC1902 The PrEP Boomerang: Who Stays, Who Leaves and Who Keeps Coming Back in Brazil?

I. Ornelas Pereira, A. Krummenauer, A. R. Pati Pascom

Ministry of Health of Brazil, Department of HIV/AIDS, Tuberculosis, Viral Hepatitis and Sexually Transmitted Infections, Brasília, Brazil

**Background**: PrEP effectiveness relies on engagement, yet metrics often classify discontinuation as failure, overlooking that cycling may reflect rational adaptation to dynamic HIV risk. Using routinely collected data from the Brazilian National Health System (SUS), profiles of engagement (sustained, cycling and discontinued) were characterized to identify population‐specific patterns beyond binary retention metrics.

**Methods**: In this retrospective cohort study, dispensing records of 261,150 PrEP users (2018–2025) were analysed. Engagement groups were defined by medication possession: (1) Sustained (continuous coverage; gaps <30 days); (2) Cycling (re‐engagement after a >30‐day gap); and (3) Discontinued (no dispensing for >180 days). Recent initiators (<6 months follow‐up) and on‐demand users were excluded to minimize selection and immortal time bias. Multinomial logistic regression estimated adjusted odds ratios (aOR) for cycling and discontinuation versus sustained use, controlling for sociodemographic factors.

**Results**: Engagement was highly heterogeneous. Travestis (a gender identity category in Brazil) had the highest cycling prevalence (47.2%; aOR 2.03, 95% CI: 1.61–2.56) versus cisgender men. Conversely, cisgender women showed the highest discontinuation (60.4%; aOR 4.47, 95% CI: 4.31–4.63). Younger users aged 18–24 years had increased odds of both cycling (aOR 1.64, 95% CI 1.57–1.72) and discontinuation (aOR 2.25, 95% CI 2.16–2.35). A strong social gradient was evident: low education nearly doubled discontinuation odds (aOR 1.93, 95% CI: 1.83–2.03), while Indigenous users showed elevated cycling (aOR 1.38, 95% CI: 1.16–1.65). Regionally, the Northeast exhibited lower odds of discontinuation than the Southeast (discontinuation aOR 0.76, 95% CI: 0.74–0.79). Non‐binary people showed cycling patterns similar to cisgender men (aOR 1.10, 95% CI: 0.96–1.27).

**Conclusions**: Distinct patterns of high cycling among Travestis challenge dropout as definitive disengagement, suggesting frequent re‐entry that may benefit from event‐driven modalities and flexible service models. High discontinuation among cisgender women and younger may reflect gender‐specific barriers or lower perceived risk. Public health policies should move beyond uniform retention targets and adopt differentiated strategies that both accommodate cyclical engagement where appropriate and address the underlying determinants of long‐term disengagement among socially vulnerable populations. (Table 1)

**TABLE 1:**
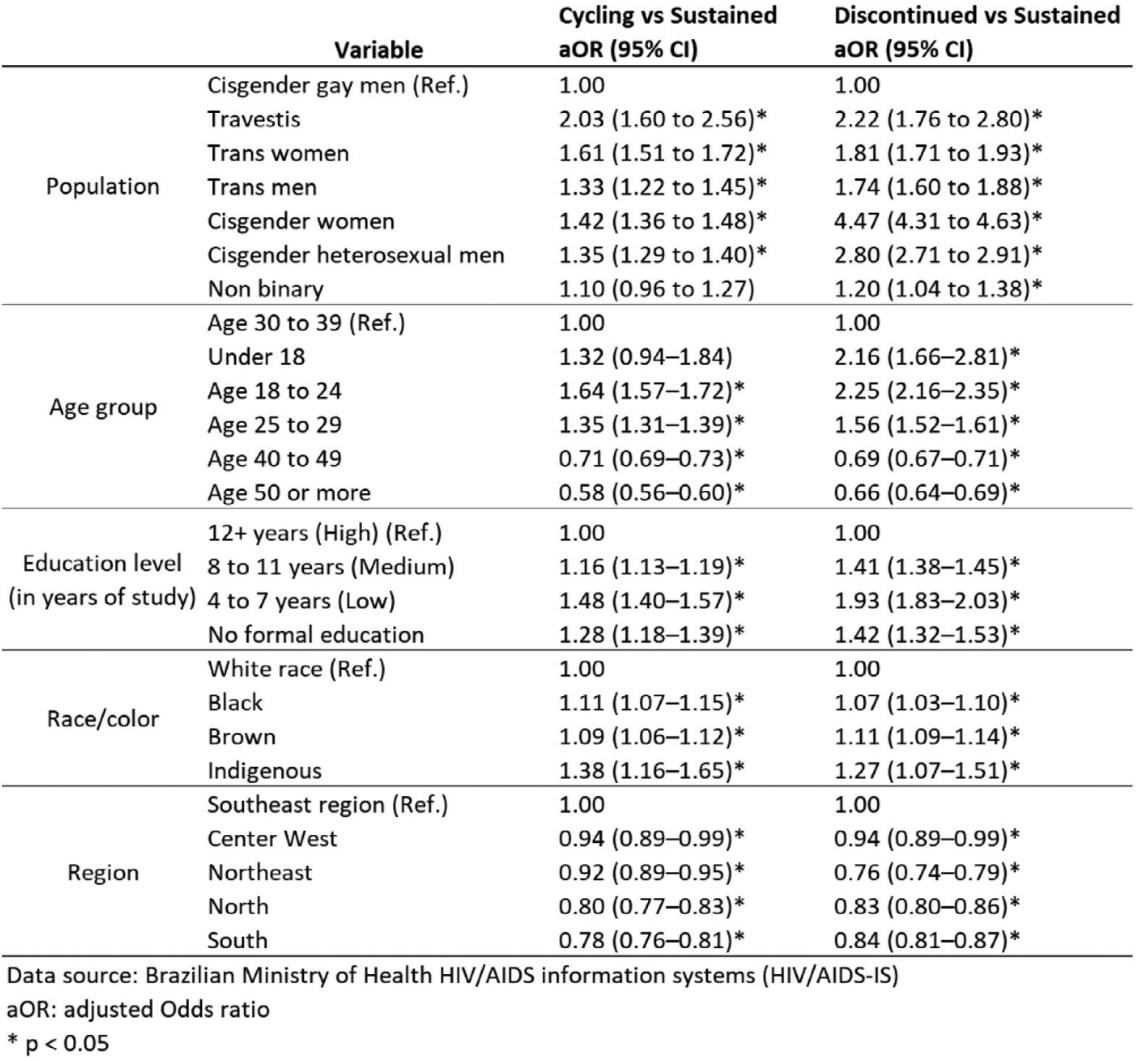
OAC1902 | Adjusted associations with PrEP engagement patterns.

## OAC1903 Effect of Different Pharmacy PEP Delivery Models on Initiation, Repeat Use and PEP‐to‐PrEP Transition: Findings From a 75‐Pharmacy Cluster‐Randomized Controlled Trial in Kenya

K. Ngure ^1^, A. Meisner^2^, V. Omollo^3^, T. Kareithi^4^, P. Ong'wen^5^, S. Roche^2^, P. Otieno^3^, L. Juma^3^, C. Kiptinness^4^, T. Schaafsma^6^, K. Thomas^6^, R. Malen^2^, K. Harkey^2^, M. Asewe^3^, E. Gichuru^4^, B. Rono^3^, M. Anyona^5^, P. Banerjee^2^, K. Yu^2^, M. Sharma^6^, J. Pintye^6^, M. Mugambi^6^, P. Shah^2^, D. Were^5^, E. Bukusi^3^, K. Ortblad^2^, On Behalf of the PharmPrEP Study Team

^1^School of Public Health, Jomo Kenyatta University of Agriculture and Technology, Nairobi, Kenya; ^2^Fred Hutchinson Cancer Research Center, Seattle, United States; ^3^Kenya Medical Research Institute, Nairobi, Kenya; ^4^Kenya Medical Research Institute, Partners in Health Research and Development, Nairobi, Kenya; ^5^JHPIEGO, Kenya, Nairobi, Kenya; ^6^University of Washington, Seattle, United States

**Background**: Private pharmacies—with long operating hours and convenient locations—are well‐positioned to deliver products for urgent needs, including HIV post‐exposure prophylaxis (PEP), effective within 72 h of a potential exposure. Barriers to implementation at scale, however, include uncertain financing, provider time constraints and lack of enabling policy.

**Methods**: The Pharm PrEP cluster‐randomized controlled trial (NCT05842122; 1:1:1:1 randomization) tested four public‐private partnership models for pharmacy‐based PEP and pre‐exposure prophylaxis (PrEP) delivery in Kenya. In three intervention arm, private pharmacies received free government commodities from linked public clinics: (A1) client‐sustained: clients (16+ years) paid pharmacies 250 KES (∼$2 USD) per PrEP/PEP visit; (A2) implementor‐sustained: clients paid nothing and implementors paid 250 KES/visit; and (A3) counsellor‐supported: clients paid nothing, implementors paid 100 KES (∼$1 USD)/visit, and counselling and HIV testing were task‐shifted to pharmacy‐stationed counsellors (paid for by implementors). Each model was compared to (A4) referral: implementors paid pharmacies 100 KES per PrEP/PEP referral to nearby clinics. In phone surveys 60‐ and 270‐days post‐enrolment, clients self‐reported PEP initiation, repeat PEP use and PEP‐to‐PrEP transition. Outcomes were pharmacy‐level counts. Rate ratios were estimated using Poisson generalized linear models accounting for pharmacy client volume and implementation duration.

**Results**: From June 2023 to April 2025, 75 pharmacies (A1: 20; A2: 17; A3: 18; A4: 20) enrolled 5808 clients (A1: 843; A2: 2254; A3: 2278; A4: 433); 64% (3718/5808) were PEP candidates. Most PEP candidates were male (58%), unmarried (62%) and age 25+ (66%); survey response was 70% (2610/3718) at 60 days and 58% (1815/3103 eligible) at 270 days. At both time points, PEP initiation rates were significantly higher with client‐sustained, implementor‐sustained and counsellor‐supported delivery compared to referral (Table 1). Compared to referral, recurrent PEP use rates were significantly higher with counsellor‐supported delivery at 60 and 270 days; PEP‐to‐PrEP transition rates were significantly higher with implementor‐sustained and counsellor‐supported delivery at 60 and 270 days, and client‐sustained delivery at 270 days.

**Conclusions**: Enabling pharmacies to directly deliver versus refer clients to clinic‐based PEP increased PEP initiation in Kenya, even when clients paid for services, highlighting this venue's potential to avert new HIV infections. Availing PrEP for free at pharmacies could facilitate PEP‐to‐PrEP transition.

**TABLE 1:**
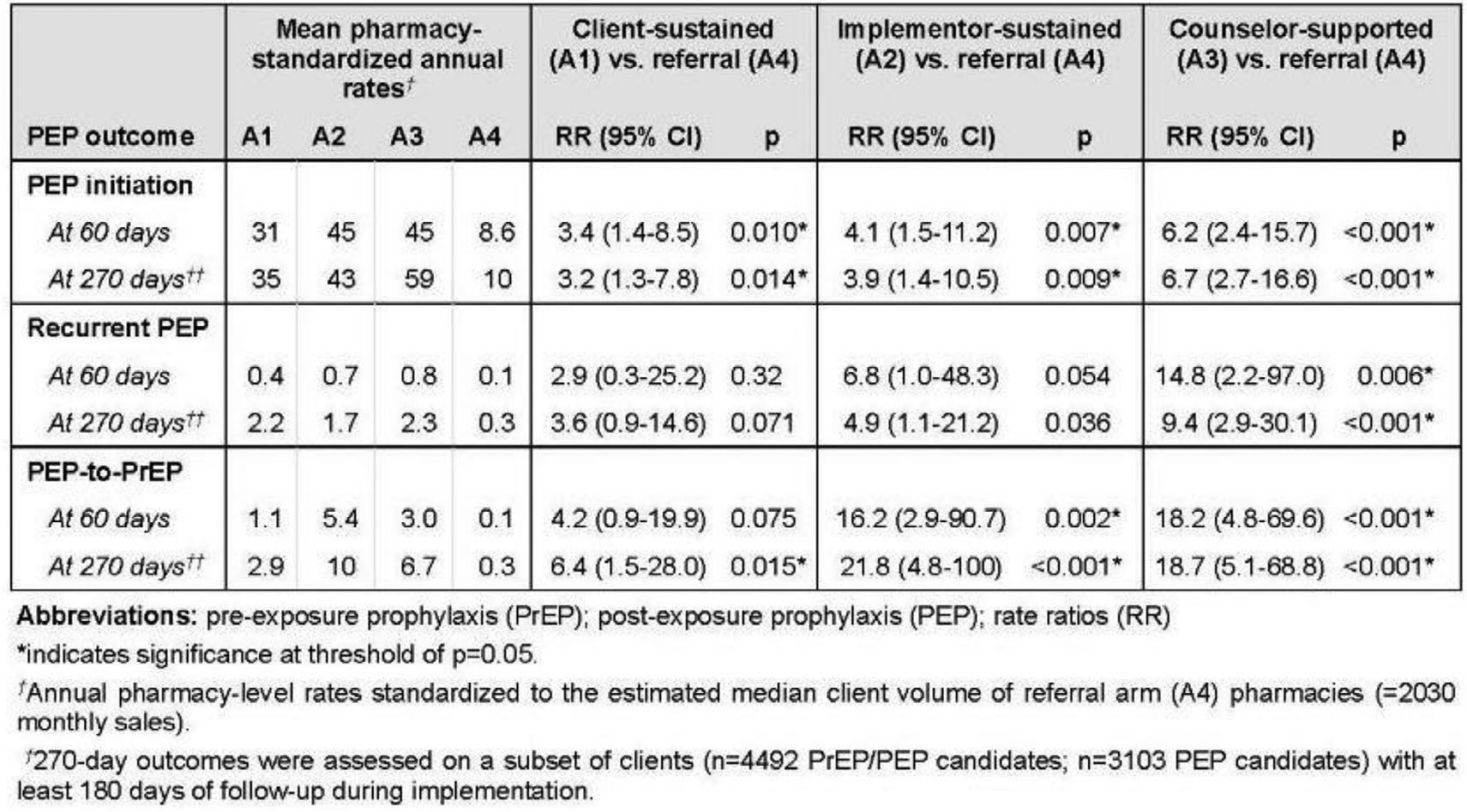
OAC1903 | Pharmacy PEP outcome at 60‐ and 270‐days post enrolment.

## OAC1904 Time to Discontinuation of Long‐Acting Injectable Cabotegravir for HIV Pre‐Exposure Prophylaxis Among Female Sex Workers and Adolescent Girls and Young Women in Zambia: A Retrospective Cohort Study (2024–2025)

P. B. Bubala ^1,2^, W. Malambo^2^, G. Moonga^2^, N. Sinyange^3^

^1^Zambia Field Epidemiology Training Program, Lusaka, Zambia; ^2^University of Zambia, School of Public Health, Department of Epidemiology and Biostatistics, Lusaka, Zambia; ^3^Zambia National Public Health Institute, Work Force Development, Lusaka, Zambia

**Background**: Zambia has one of the highest HIV burdens in SSA, with approximately 30,000 adults aquiring HIV in 2025. Women and girls of all ages carry a disproportionate burden of HIV, with adolescent girls and young women (AGYW) accounting for 38% of new acquisitions and FSWs having a prevalence as high as 45%. While oral PrEP is effective, it bears the burden of daily pill intake and stigma. The recent introduction of long‐acting (cabotegravir) injectable PrEP offers a new, potentially more discreet and convenient alternative that promises improved retention through reduced dosing. Evidence demonstrating PrEP discontinuation patterns outside of clinical trials remains limited. This study aimed to assess the survival of AGYW and FSWs on CAB‐LA for HIV prevention in Zambia, and to identify factors associated with discontinuation.

**Methods**: A retrospective cohort study was conducted using routinely collected, individual‐level longitudinal data from the DHIS2 Tracker system for AGYW and FSWs initiating CAB‐LA across seven districts in Zambia between February 2024 and June 2025. Kaplan–Meier estimates and Cox proportional hazards regression were employed to determine survival time and factors associated with discontinuation over a 20‐month period.

**Results**: The survival analysis involved 696 individuals, contributing 415,579, person‐days of follow‐up, with 427 recorded discontinuations. The median survival time was 224 days with a discontinuation incidence rate of 1.03 x 1000 person‐day and corresponding to a period between injection four and five. Survival probabilities decreased over time; 98% at 1 month, 62.3% at 3 months, 55.7% at 5 months, and 37.6% at 7 months and 3.9% at 13 months. PrEP naïve individuals had a 38% higher hazard of discontinuation [HR = 1.38, *p* = 0.037]. Urban residence was protective [HR = 0.32, *p* = <0.001] with a 68% lower hazard of discontinuation.

**Conclusions**: Survival probabilities for CAB‐LA demonstrate early and progressive discontinuation over time. Findings underscore the importance of interventions tailored at improving retention in the first few months (see Figures 1 and 2).

**FIGURE 1:**
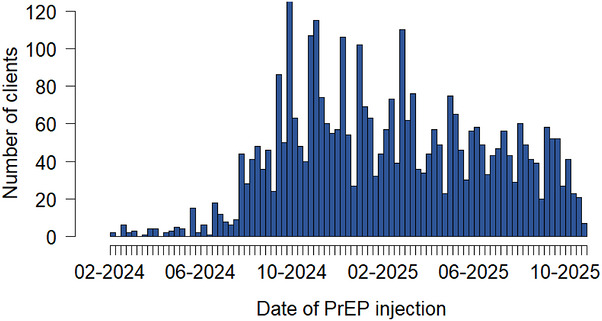
OAC1904 | Histogram of CAB‐LA update, 2024–2025.

**FIGURE 2:**
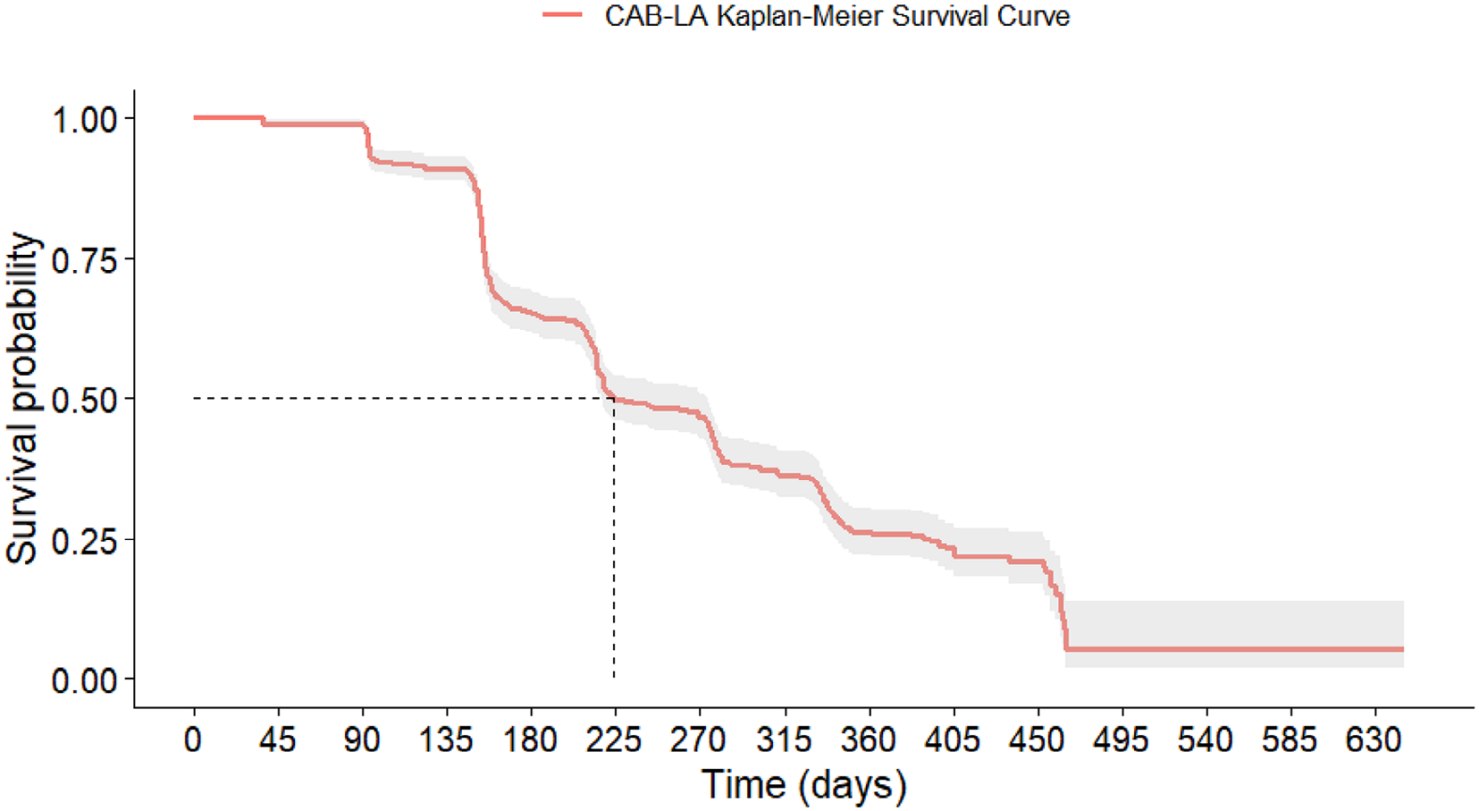
OAC1904 | Kaplan‐Meier survival curve for CAB‐LA discontinuation.

## OAC1905 Back to *PrEP à Porter*: Addressing Lost to Follow‐Up Participants in a Community‐Based Multidisciplinary PrEP Implementation Strategy for Trans Women in Paris

F. Pipitone ^1,2^, G. Girard^1^, A. Faye^1^, V. Isernia^3^, F. Peralta^4^, L. Cruz Villanueva^4^, F. Ouvrard^5^, B. Spire^1^, M. Mora^1^, L. Zimmermann^4^, G. Rincо́n^4^, C. Pignedoli^1^, M. Cervantes^3^, M. Auriche^3^, A. Deprez^3^, S. Le Gac^5^, A. Benalycherif^6^, R. Landman^6^, L. Blanquart^4^, L. Sagaon Teyssier^1^, J. Ghosn^3,5^

^1^Aix Marseille Univ, Inserm, IRD, SESSTIM, Economic and Social Sciences of Health & Medical Information Processing, Marseille, France; ^2^Coalition PLUS Scientific Network, Pantin, France; ^3^SMIT, Department of Infectious and Tropical Diseases, Bichat–Claude Bernard Hospital, Paris, France; ^4^ACCEPTESS‐T, Concrete Actions Combining Education, Prevention, Work, Equity, Health, and Sports for Transgender People, Paris, France; ^5^CoReSS, Regional Coordination for Sexual Health, North‐West Île‐de‐France, Paris, France; ^6^IMEA, Institute of Applied Medicine and Epidemiology, Paris, France

**Background**: Loss to follow‐up (LTFU) among transgender women (TW) in PrEP programmes represents a major public health challenge. This study aims at exploring factors associated with PrEP disengagement within a TW‐tailored programme in Paris.

**Methods**: *PrEP à Porter* (2022–2026) is a single‐arm, monocentric study, aiming to improve PrEP retention among TW. It relies on a community‐based programme at Acceptess‐T association, tailored to TW. Mixed methods were used: Quantitative data (sociodemographics, health status, access to care, sexual behaviours and programme‐related satisfaction, self‐reported PrEP adherence) and qualitative data (in‐person/phone interviews with LTFU) were collected. Data from inclusion until week 96 (W96) were analysed. Quantitative outcome: LTFU when missing the two last visits (W84−W96). Multivariate logistic regression estimated LTFU‐associated factors (OR & 95% CI). Qualitative thematic analysis was conducted over the interviews.

**Results**: Of the 101 TW (839 visits), 94% were born in Latin America, median [IQR] age was 34 [29−40] years, 50.5% without social security and 91.1% sex workers. At baseline, 40.6% were PrEP‐naive, and among those on PrEP, 76.2% were on daily regimen. Acceptess‐T and Bichat‐hospital followed up 31.7% and 43.6% of participants, whereas 24.8% were in both structures. Multivariate regression: LTFU was more likely for PrEP‐naive (OR [95% CI]: 4.54 [2.93, 7.03]), low/unknown adherence (3.76 [2.28, 6.20]) and non‐French speakers (2.37 [1.60, 3.51]). LTFU odds were lower for gender‐affirming‐related surgery (0.34 [0.16, 0.71]), silicone injection (0.46 [0.29, 0.73]), financial difficulties (0.39 [0.27, 0.59]) and association‐hospital follow‐up (0.17 [0.09, 0.34]). Willingness to use mental health (0.26 [0.17, 0.39]) and social (0.30 [0.18, 0.49]) services had lower odds of LTFU. Actual/perceived side‐effects and low PrEP‐related experience explained low/unknown adherence. Not expressing social or mental health needs was related to unstable living conditions and discrimination leading to disengagement. PrEP‐interruption–driven LTFU was explained by changes in sexual behaviours, low awareness of PrEP‐delivery options, fear of visiting healthcare structures; whereas some LTFU are now followed up by general practitioners. Participants emphasized the importance of community‐mediator outreach; when this support was missing due to system failures, it translated with disengagement from PrEP follow‐up.

**Conclusions**: Improving TW‐sensitive social skills among medical staff, diversifying PrEP services delivery, increasing awareness of PrEP options and strengthening mediation services may be crucial to foster retention but also as part of a “re‐engagement” strategy.

## OAC2202 Community Insights on Acceptability of Location‐Enabled Devices to Improve Retention Among People Who Inject Drugs in Oakland and San Francisco, California

L. Sheira ^1^, B. Supraset^2^, R. Ruiz^2^, P. Lella^2^, I. O'Neal^2^, G. Velasquez^2^, E. Wilson^2^, W. McFarland^2^

^1^University of California, San Francisco, School of Nursing, San Francisco, United States; ^2^San Francisco Department of Public Health, San Francisco, United States

**Background**: San Francisco has made significant gains in addressing the HIV epidemic, but progress has been uneven. While there was a 65% reduction in incident HIV cases among men who have sex with men from 2012 to 2020, the decline was much less (38%) among people who inject drugs (PWID). PWID face intersecting marginalizations like housing insecurity and racial inequities. Marginalizations are impediments to their engagement and retention in HIV prevention, care and research. We collected data to assess the acceptability and willingness of PWID to use location‐enabled devices to maintain contact for retention.

**Methods**: The Brief Longitudinal Incident Sentinel Surveillance (BLISS) cohort study recruited 600 PWID in San Francisco and Alameda counties and is currently following them over 3 years to measure HIV and HCV intervention uptake and link participants to care. Despite rapidly recruiting the complete cohort, retention remains a challenge. We conducted a mixed‐methods formative research study to understand how to better retain PWID in HIV and HCV prevention studies. We collected qualitative and quantitative data around acceptability of using a GPS device for retention in the BLISS cohort.

**Results**: We recruited 245 BLISS participants to the survey and 11 participants to two Participant Advisory Board Meetings. GPS for retention was acceptable among most survey participants. Most (61%) were completely willing to carry a GPS device and an additional 13% were open to carrying a GPS device after learning more about it (13%); fewer were not at all willing (14%) or unsure (7%). Themes in the Participant Advisory Board meeting included worry about losing the GPS device as many of their belongings are regularly lost or stolen, preference for not being located while acquiring substances and fear of being surveilled. Notably, when specifically prompted, data security concerns were not salient. Willingness to try a GPS device overall outweighed concerns.

**Conclusions**: Given advances in HIV treatment and prevention, as well as in affordable GPS technologies, the use of GPS devices as a method to improve BLISS participant retention may have far‐reaching implications for other research and healthcare programmes with PWID and other harder‐to‐reach populations, warranting testing their use in trials.

## OAC2203 Vínculo Seguro: Implementing HIV Self‐Testing Through Dating Apps to Close the HIV Diagnosis Gap in Mexico

A. Piñeirua‐Menéndez ^1^, F. Macías‐González^2^, J. Hecht^3^, T. Mosugu^3^, M. Hernández‐Leyva^1^, F. Rivera‐Buendía^1^, M. Sundararaj^4^, E. Huang^4^

^1^CISIDAT, Cuernavaca, Mexico; ^2^Instituto Nacional de Salud Pública, Cuernavaca, Mexico; ^3^Building Healthy Online Communities (BHOC), Richmond, United States; ^4^Grindr for equality, Los Angeles, United States

**Background**: HIV diagnosis remains the largest gap in achieving the 95–95–95 targets in Mexico. This study aimed to assess the acceptability and feasibility of an HIV self‐testing (HIVST) strategy primarily promoted through men who have sex with men (MSM) dating applications in two Mexican states. Additionally, we explored awareness, knowledge and use of PrEP among this population.

**Methods**: In collaboration with BHOC and Grindr, we implemented a digital campaign offering free HIVST kits to Grindr users and individuals reached through other social media platforms in two Mexican states (@vinculoseguro). Participants could choose to collect their HIVST kits at community‐based organizations (CBOs) or receive home delivery via courier services within 48–72 h. Multiple linkage‐to‐care and counselling options were provided, including chatbots, telecounselling, WhatsApp‐based counselling and informational videos. Individuals requesting a test were invited to complete a pre‐order questionnaire assessing PrEP knowledge and prior use, as well as a post‐order questionnaire evaluating their HIV self‐testing experience and test results.

**Results**: Over a 9‐week period, 541 self‐test orders were placed. The mean age of participants was 30.5 years (SD: 8.1), with 25.4% aged 18–24 years. Most participants identified as cisgender men (91.9%), followed by cisgender women (4.1%) and transgender women (1.3%). Overall, 86.5% of participants were aware of PrEP, and 85.8% expressed interest in using it; however, only 10.5% had ever used PrEP and 3.3% were current users. 33.1% of participants reported condomless sex in the previous 72 h, and 29.8% were first‐time HIV testers. Among delivered kits, 38.4% completed the post‐order questionnaire: 99% of the individuals reported that the test was easy to use and would recommend the service; 25% reported a history of chemsex, and 4.2% of them reported a reactive HIV self‐test result.

**Conclusions**: HIV self‐testing is a feasible and acceptable approach for reaching at‐risk individuals, particularly those who have never tested. Despite high awareness and willingness to use PrEP, actual use remains low. The goal of this study is to assess feasibility for possible national scale‐up of this strategy. By the time the conference is held, we expect to reach at least 3000 orders.

## OAC2204 Reaching Vulnerable Populations With Online HIV Self‐Testing: Results From the Stand By You Platform, Thailand, 2022–2025

U. Kritsanavarin ^1^, P. Pongsakul^2^, K. Sripanidkulchai^3^, Y. Durier^4^, P. Pattarayanon^1^, A. Maleestharn^4^, P. Urujchtchairut^4^, B. Khumcha^4^, T. Buason^4^, S. Tophong^5^, T. Puangmalee^1^, P. Prommali^6^, M. Vasantiuppapokakorn^6^, Y. Somphoh^7^, S. Rungmaitree^4^, S. Jiamsiri^5^, K. Chokephaibulkit^4^, S. Northbrook^1^

^1^U.S. Centers for Disease Control and Prevention, Division of Global HIV and TB, Nonthaburi, Thailand; ^2^Ministry of Public Health, Division of AIDS and STIs, Nonthaburi, Thailand; ^3^Mahidol University, Faculty of Medicine Siriraj Hospital, Department of Preventive and Social Medicine, Bangkok, Thailand; ^4^Mahidol University, Division of Infectious Disease, Department of Pediatrics, Faculty of Medicine Siriraj Hospital, Bangkok, Thailand; ^5^Ministry of Public Health, Department of Disease Control, Nonthaburi, Thailand; ^6^Ministry of Public Health, Division of AIDS and STIs, Department of Disease Control, Nonthaburi, Thailand; ^7^Thai National AIDS Foundation (TNAF), Bangkok, Thailand

**Background**: Since August 2022, the Ministry of Public Health and Siriraj Hospital have implemented the Stand By You (SBY) platform, an online service designed to reach high‐risk individuals with HIV self‐testing (HIVST) and link them to health services through social media. We report on the uptake of HIVST and HIV‐positivity among high‐risk individuals from August 2022 to August 2025.

**Methods**: SBY was promoted across four major social media websites. All eligible participants provided informed consent, completed a risk assessment and received counselling. Those who reported high‐risk behaviours were mailed HIVST kits, performed the HIVST unassisted and reported results online. High‐risk individuals were defined as those who reported inconsistent or no condom use, multiple sex partners, a history of sexually transmitted infections (STIs), sex in exchange for money or injecting drug use. Data on risk behaviours and HIVST screening were collected. Participants with reactive results were instructed to obtain confirmatory testing and initiate treatment at designated hospitals.

**Results**: Of 33,636 eligible individuals, 84% (28,293) reported being at risk for HIV and received HIVST kits. Of those, 72% (20,453) reported their results online, with 3% (606) testing reactive; 63% (383) of those linked to confirmatory testing. The most common risk behaviour was inconsistent condom use reported by 13,748 participants, with 7% (462) testing reactive, followed by multiple sexual partners (10,450 participants, 3% [336] reactive), history of STIs (6220 participants, 4% [226] reactive), sex in exchange for money (3359 participants, 5% [165] reactive), no condom use (3286 participants, 3% [87] reactive) and injection drug use (264 participants, 8% [24] reactive). Approximately 82% (494) of participants with reactive results reported at least two high‐risk behaviours. Among 13,924 participants aged 15−24 years, 72% (9996) reported results with 3% (272) testing reactive. Of these, 58% (159) were first‐time HIV testers.

**Conclusions**: The SBY platform reached high‐risk populations with HIVST, with nearly half of participants aged 15−24 being first‐time testers. Our findings highlight the role social media can play in connecting individuals to essential health services and the ongoing need to address high‐risk behaviours to reduce HIV transmission in Thailand.

## OAC2205 Effect of an Interactive Text‐Messaging Intervention on Retention in HIV Care Among Men Who Have Sex With Men in Peru: A Randomized Controlled Trial (WelTel Peru)

L. A. Menacho Alvirio ^1^, G. Díaz^1^, C. Pantoja^2^, S. Villanueva^2^, A. Duerr^3^

^1^Universidad Peruana Cayetano Heredia, Instituo de Medicina Tropical Alexander Von Humboldt, Lima, Peru; ^2^Hospital Cayetano Heredia, PROCETSS, Lima, Peru; ^3^University of Washington, Global Health and Epidemiology, Seattle, United States

**Background**: The WelTel platform has been used to promote adherence to antiretroviral therapy (ART) among people living with HIV. We adapted this web‐based platform and evaluated its efficacy in increasing retention in care among HIV‐positive men who have sex with men (MSM) in Peru.

**Methods**: From January 2022 to May 2023, we enrolled 208 participants at one of the largest government hospitals in Lima, Peru. We conducted a two‐arm randomized controlled trial. Participants in the intervention arm were followed by trained nurses using the WelTel platform for 6 months. Retention was defined as attendance at scheduled HIV clinic visits, with retention requiring four visits within 1 year of enrolment.

**Results**: The mean age was 30 years; 20% self‐identified as bisexual, and more than 25% were from Venezuela. Over 96% reported smartphone use, and 45% had completed graduate education. Nearly one‐third (33%) reported having one sexual partner in the past 3 months, and 17% reported unprotected anal sex at their most recent encounter. Retention in HIV care was defined as attendance at nursing visits for medication refills, medical appointments, and laboratory monitoring of CD4 count and viral load. As a binary outcome, retention was 94% (98/104) in the intervention group and 85% (89/104) in the control group (Pearson χ^2^(1) = 4.29, *p* = 0.038). The risk of being retained in care was 10% higher in the intervention group (risk ratio = 1.10; 95% CI: 1.004–1.207). As a continuous outcome, the mean number of attended appointments was 6.67 in the intervention group and 5.69 in the control group (Wilcoxon rank‐sum test: *z* = −2.72, *p* = 0.0065). The physical component summary score of health‐related quality of life was also higher in the intervention group (68.8 vs. 61.5; *t* = −2.04, *p* = 0.043).

**Conclusions**: The WelTel platform improved retention in HIV care and quality of life among HIV‐positive clients at a public hospital in Peru after 1 year of follow‐up. These findings indicate that mobile‐based interventions delivered by trained nurses can strengthen HIV care engagement. Future interventions could incorporate mental health components to further enhance retention and quality of life.

## OAC2802 Zero HIV Acquisitions With Twice‐Yearly Subcutaneous Lenacapavir for PrEP During 52 Weeks of Open‐Label Extension in PURPOSE 1

N. Kiwanuka ^1^, M. Malahleha^2^, G. Nair^3^, I. Harkoo^4^, F. M. Kiweewa^5^, S. Puryear^6^, C. C. Carter^6^, Y. Chen^6^, A. Akhtar^6^, C. Louw^7^, L. E. Mansoor^4^

^1^Department of Epidemiology and Biostatistics, Makerere University School of Public Health, Kampala, Uganda; ^2^Synergy Biomed Research Institute, East London, South Africa; ^3^Desmond Tutu HIV Foundation, University of Cape Town, Cape Town, South Africa; ^4^Centre for the AIDS Programme of Research in South Africa, University of KwaZulu‐Natal, Durban, South Africa; ^5^Makerere University‐Johns Hopkins University Research Collaboration, Kampala, Uganda; ^6^Gilead Sciences, Inc., Foster City, CA, United States; ^7^Madibeng Centre for Research, Brits, South Africa

**Background**: Twice‐yearly subcutaneous lenacapavir for HIV pre‐exposure prophylaxis (PrEP) showed superior efficacy to daily oral emtricitabine/tenofovir disoproxil fumarate (F/TDF) in cisgender women in PURPOSE 1 (NCT04994509). Following the randomized blinded phase (RBP), participants could choose to receive lenacapavir in an open‐label extension (OLE). We report HIV incidence, safety, adherence to injections during the first 52 weeks of OLE and cumulative HIV incidence from study start through OLE Week 52 for participants receiving lenacapavir.

**Methods**: Following the primary analysis of PURPOSE 1, participants completed final RBP visits on a rolling basis (July 8−October 21, 2024). At the final RBP visit, participants on randomized study drug were offered open‐label lenacapavir. In the optional OLE, participants received subcutaneous lenacapavir every 26 weeks, including oral lenacapavir loading if they had not received lenacapavir in the prior 28 weeks. HIV incidence was assessed in participants who received lenacapavir in any study phase through OLE Week 52. Adverse events were assessed at all study visits. Adherence was defined as lenacapavir administration within 28 weeks after the last injection.

**Results**: Of 4417 participants eligible to join the OLE, 4206 (95.2%) elected to start or continue lenacapavir. There were zero incident HIV acquisitions among all participants on open‐label lenacapavir during the first 52 weeks of OLE with over 4493 person‐years (PY) of follow‐up. From trial start through OLE Week 52, over 7178 PY of follow‐up, there was one incident HIV acquisition in a participant on blinded lenacapavir (incidence 0.014/100 PY; 95% CI 0.000, 0.078). There was one additional acquisition in a participant following switch from blinded lenacapavir to open‐label F/TDF. HIV incidence among participants on F/TDF or emtricitabine/tenofovir alafenamide (F/TAF) in the RBP was 1.94/100 PY and 1.98/100 PY, respectively; in those who switched to lenacapavir in OLE, HIV incidence was 0/100 PY through the OLE Week 52 visit. No new safety concerns were identified. Adherence to injections at OLE Week 52 was 96%.

**Conclusions**: Twice‐yearly lenacapavir demonstrated high efficacy with zero HIV acquisitions through Week 52 of the OLE; this, in combination with high adherence and a consistent safety profile, reinforces lenacapavir as a transformative PrEP option for cisgender women.

## OAC2803 Challenges in Early HIV Detection During CAB‐LA PrEP: Characterization of Inconclusive Serological Results in ImPrEP CAB Brasil

J. Jacobs ^1^, B. Hoagland^2^, C. Coutinho^2^, M. Benedetti^2^, D. Hoeth^1^, A. Naqvi^1^, A. Heaps^1^, K. Penrose^1^, J. Mellors^1^, S. Nazer^2^, R. Trefiglio^2^, R. Landovitz^3^, V. Veloso^2^, B. Grinsztejn^2^, U. Parikh^1^, ImPrEP Study Team

^1^University of Pittsburgh, Medicine, Division of Infectious Disease, Pittsburgh, United States; ^2^Instituto Nacional de Infectologia Evandro Chagas, Fundação Oswaldo Cruz, Rio de Janeiro, Brazil; ^3^University of California Los Angeles, Division of Infectious Diseases, Los Angeles, United States

**Background**: Although long‐acting cabotegravir (CAB‐LA) PrEP is highly effective, rare HIV‐1 acquisition events occur. CAB‐LA's impact on viral replication dynamics makes early detection challenging. We present the first analysis of ultrasensitive HIV‐1 RNA in individuals on CAB‐LA PrEP in routine public health settings in Brazil with inconclusive HIV diagnoses.

**Methods**: ImPrEP CAB Brasil is an implementation study of same‐day CAB‐LA initiation at oral PrEP clinics across six Brazilian cities. Individuals aged 18–30 years are enrolled after a negative rapid antibody (Ab)‐only diagnostic test (RDT) and undetectable HIV‐1 RNA by GeneXpert. At each subsequent visit, CAB‐LA is administered following a negative Ab RDT; HIV‐1 RNA (Roche COBAS), Ab/Ag RDT and 4th‐gen Abbott Architect is performed retrospectively. Positive RDT are confirmed by Geenius. Discrepant results were further evaluated with BioPlex 2200 HIV Ag/Ab and ultrasensitive HIV‐1 RNA (SCA; LOD ≥3 c/mL) on samples at time points on or after positive serology results.

**Results**: Among 1220 participants (pt) enrolled in the ImPrEP incidence cohort between 11/2023 and 9/2024, 14 pt (1%) on CAB‐LA (all MSM) had at least one positive serological test. The median time from CAB‐LA initiation to first positive serological test was 142 days (range 0−552), with a median of 3 (range 0−10) CAB‐LA injections prior to first positive serological test. Of these 14, four (Group A) had a single positive serological test; six (Group B) had a positive serological test at >1 time point; and four (Group C) had multiple positive serological tests at a single time point. Retrospective higher‐sensitivity research‐use testing was non‐reactive (Bioplex) or not detected (SCA) across all groups. Study disposition was: From Group A, 2/4 remained on CAB‐LA; 2/4 withdrew from the study. From Group B, 4/6 remained on CAB‐LA; 1/6 was lost to follow‐up; 1/6 switched to oral PrEP. From Group C, all 4/4 remained on CAB‐LA.

**Conclusions**: The absence of detectable HIV‐1 RNA by ultrasensitive SCA among participants with inconclusive HIV diagnoses suggests either false‐positive Ab and/or Ab/Ag serological test results or suppression of viraemia with continued CAB‐LA injections. Further studies are needed to better define diagnostic strategies for resolving discrepant results in CAB PrEP programmes. (Table 1)

**TABLE 1:**
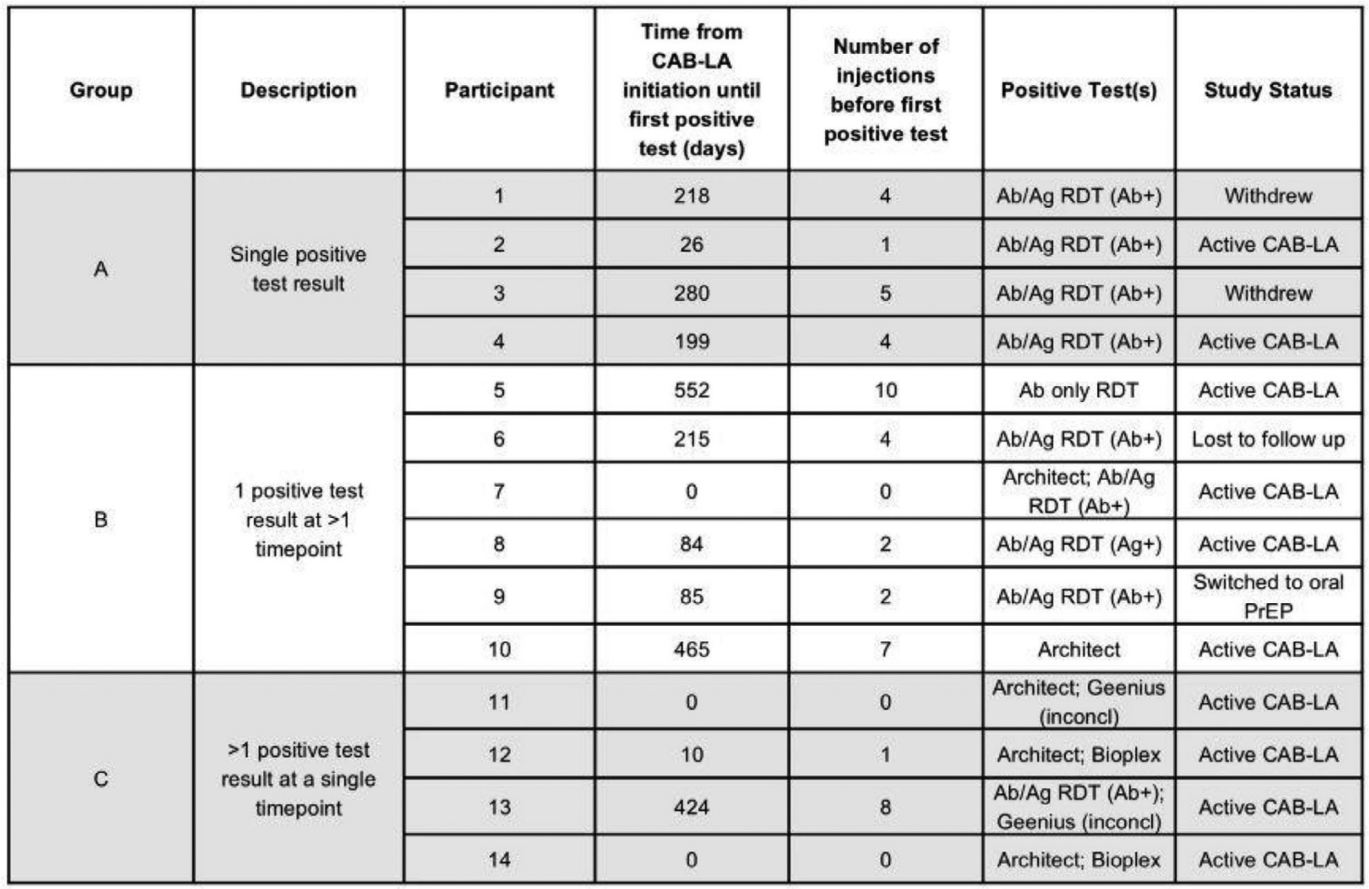
OAC2803

## OAC2804 Results of a Randomized, Double‐Blind, Phase 1b Study to Evaluate the Pharmacokinetics, Safety and Bleeding Associated With Two 90‐Day Multipurpose Dapivirine‐Levonorgestrel Vaginal Rings for HIV and Pregnancy Prevention

K. Kleinbeck ^1^, J. Nuttall^1^, E. Dorman^1^, J. Visser^2^, M. Plagianos^1^, M. Kamupira^2^, J. Ivorra^3^, C. Dart^3^, A. Blackmon^3^, A. Edelman^4^, B. A. Chen^5^, D. Blithe^6^, B. Devlin^1^

^1^Population Council, Center for Biomedical Research, New York City, United States; ^2^Population Council, International Partnership for Microbicides – South Africa, Cape Town, South Africa; ^3^Premier Research, Morrisville, United States; ^4^Oregon Health and Science University, Department of Obstetrics and Gynecology, Portland, United States; ^5^University of Pittsburgh, Complex Family Planning, Obstetrics and Gynecology, Pittsburgh, United States; ^6^NIH National Institute of Child Health and Human Development, Bethesda, United States

**Background**: Women face overlapping risks of unintended pregnancy and HIV acquisition, yet condoms remain the only multipurpose prevention technology widely available. Two candidate 90‐day, women‐controlled vaginal rings (VRs) have been developed for pregnancy and HIV prevention.

**Methods**: This double‐blind, Phase 1b trial (CCN019B/IPM‐056) randomized 40 healthy, HIV‐negative women with BMI <40 kg/m^2^ at two United States sites. Participants received one of two VRs (Ring‐105 or Ring‐106) for approximately 90 days of continuous use. Both ethylene‐vinyl acetate (EVA) core‐sheath VRs contained dapivirine (DPV, 87 or 45 mg) (with the same first‐order release profile as the DapiRing) and levonorgestrel (LNG, 147 or 167 mg) (releasing in two different zero‐order profiles). The primary endpoint was local and systemic pharmacokinetics (PK) of DPV and LNG concentrations in plasma and cervicovaginal fluid; secondary endpoints included safety and bleeding patterns. Ovarian function (serum progesterone and oestradiol concentrations), adherence and acceptability were exploratory endpoints.

**Results**: PK (Figure 1): For both rings, mean LNG plasma concentrations for the 38 participants completing the study exceeded the target threshold of ≥350 pg/mL, with Ring‐106's concentrations being consistently higher. Mean DPV plasma concentrations for both rings demonstrated first‐order release in‐line with levels comparable to the commercially available DPV‐only vaginal ring.

Ovarian Function: Adherent use of either ring consistently suppressed progesterone (<0.1 ng/mL) from 22 days of use onwards; Ring‐106 suppressed oestradiol more consistently (Day 70 mean, standard deviation [SD]: 37.9, 22.62) than Ring‐105 (Day 70 mean, SD: 121.9, 129.19).

Safety: Adverse events (AEs) were generally mild or moderate (Table 1). Fewer AEs related to product were observed in participants using Ring 106. A single Grade 4 was deemed not to be treatment related.

Bleeding: Ring‐106 resulted in fewer bleeding episodes, defined as intervals of bleeding bound by 2 days of no bleeding or spotting, (mean 1.7, SD 1.22) than Ring‐105 (mean 2.8, SD 1.32) and fewer days of heavy bleeding (mean 0.3, SD 0.92) than Ring‐105 (mean 1.2, SD 1.57).

**Conclusions**: Both rings were found to be safe and achieved the PK targets; Ring‐106 was found to have superior suppression of progesterone and oestrogen, fewer treatment‐related AEs and a reduced bleeding pattern.

**FIGURE 1:**
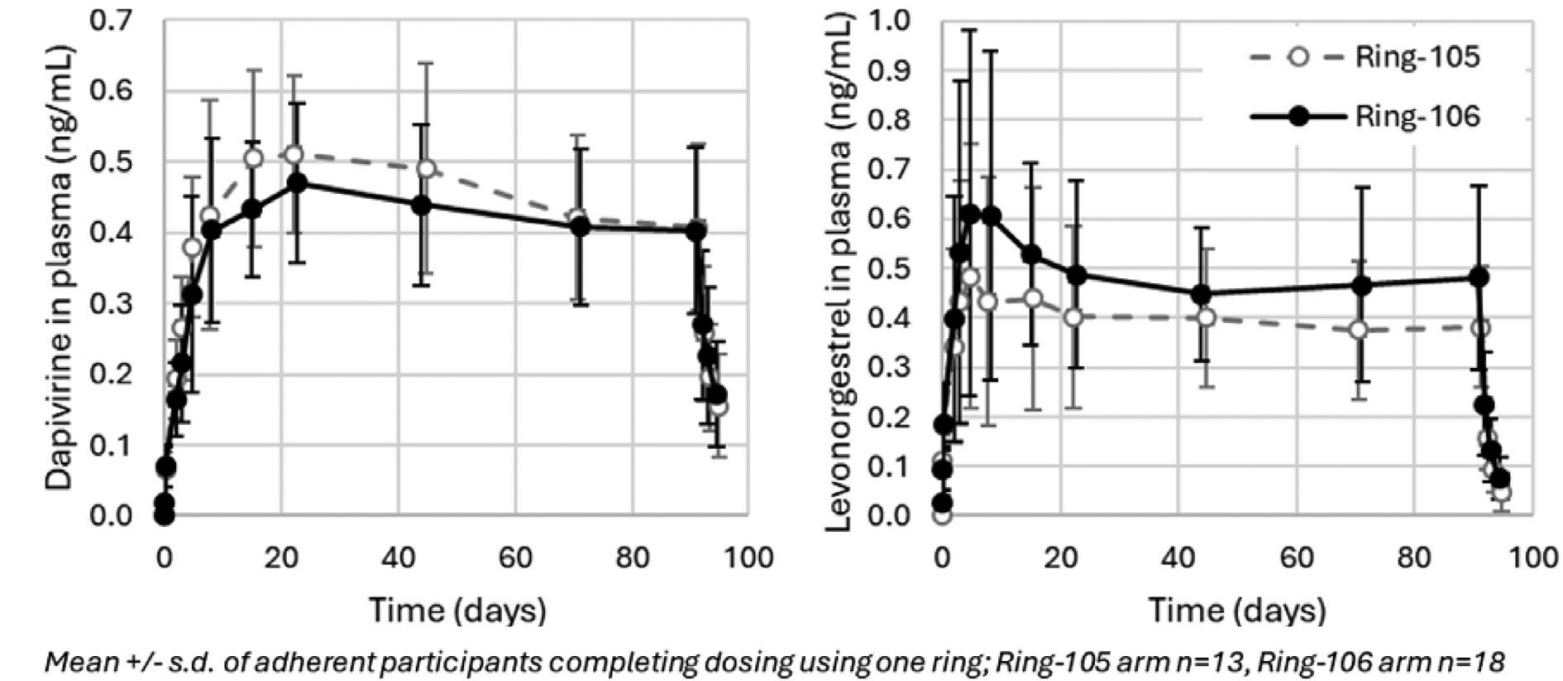
OAC2804 | Mean plasma pharmacokinetic profile, dapivirine and levonorgestrel.

**TABLE 1:** OAC2804 | Adverse events occurring in one or more participants.

Adverse events	Ring‐105	Ring‐106	Total
Grade 5 (death)	−	−	−
Grade 4 (potentially life‐threatening)	−	1 (5%)	1 (2.5%)
Grade 3 (severe)	−	−	−
Grade 2 (moderate)	5 (25%)	7 (35%)	12 (30%)
Grade 1 (mild)	13 (65%)	11 (55%)	24 (60%)
Related to product	13 (65%)	9 (45%)	22 (55%)
Unrelated to product	5 (25%)	10 (50%)	15 (37.5%)
AEs leading to study discontinuation	3 (15%)	1 (5%)	4 (10%)

## OAC2805 No HIV Acquisitions Among Women on Cabotegravir Long‐Acting Injectable PrEP Despite Mostly Short Injection Delays in the US OPERA Cohort

L. Armas‐Kolostroubis ^1^, L. Brunet^2^, M.‐G. Toeque^3^, J. Altamirano^4^, Q. Cochran^5^, C. Markarian^3^, C. Sherman^6^, A. Guignard^7^, K. Brown^8^, A. de Ruiter^9^, V. Vannappagari^8^, B. Levis^2^, J. S. Fusco^2^

^1^AIDS Healthcare Foundation, Dallas, United States; ^2^Epividian, Inc., Raleigh, United States; ^3^AIDS Healthcare Foundation, Los Angeles, United States; ^4^CAN Community Health, Miami, United States; ^5^AIDS Healthcare Foundation, Fort Lauderdale, United States; ^6^CAN Community Health, Arlington, United States; ^7^ViiV Healthcare, Wavre, Belgium; ^8^ViiV Healthcare, Durham, United States; ^9^ViiV Healthcare, London, United Kingdom

**Background**: While the efficacy of cabotegravir long‐acting pre‐exposure prophylaxis (CAB LA PrEP) has been demonstrated among transgender and cisgender women, information on real‐world effectiveness is limited. We described patterns of CAB LA PrEP use and HIV acquisition among women in the US‐based, real‐world OPERA Cohort.

**Methods**: Cisgender or transgender adult women without HIV who received ≥1 CAB LA PrEP injection (21DEC2021−31DEC2024) were followed until censoring (30APR2025, loss to follow‐up, death). Adherence to maintenance injections and HIV acquisition were assessed until first of discontinuation or censoring event. Reinitiation (≥1 additional injection after incomplete initiation or discontinuation) was assessed until first censoring event.

**Results**: Of 315 women starting CAB LA PrEP, 66% had previously received an oral PrEP prescription. At the time of the first injection, median age was 28 years (IQR: 23, 37), 43% were Black, 32% were transgender, 43% had a BMI ≥30 kg/m^2^ and 32% had an STI diagnosis in the past 12 months. Within 2 weeks of the first injection, 84% had received an HIV test. Most women (80%) completed CAB LA PrEP initiation (87% on‐time; Table 1). Of the 63 with incomplete initiation, 23 (36%) received additional injection(s) a median of 105 days later (IQR: 75, 157). Over a median of 13 months of follow‐up (IQR: 8, 19), 44% of complete initiators had ≥1 late maintenance injection (Table 2), 64% of whom had only one late injection. Most delays were short; the next injection was received a median of 8 days after the target (IQR: 3, 17). Of the 80 women who discontinued CAB LA PrEP, 25% received additional injection(s) a median of 216 days later (IQR: 156, 262). There were no HIV acquisitions among women on CAB LA PrEP.

**Conclusions**: CAB LA PrEP was highly effective against HIV acquisition among women in routine clinical care in the United States. No HIV acquisitions were observed despite mostly short delays between 44% of injections. Incomplete oral bridging records may contribute to the overestimation of delays and discontinuation. Among women who did not complete initiation or discontinued CAB LA PrEP, up to a third reinitiated, indicating continued interest in this PrEP option.

**TABLE 1:** OAC2805 | Timing of CAB LA PrEP initiation injections among women with ≥1 injection (*N* = 315).

	Timing	*n* (%)
Complete initiation	≤67 days between injections	252 (80)
2nd initiation injection received on‐time	23−37 days	220 (87)
2nd initiation injection received late	38−67 days	32 (13)
Incomplete initiation	>67 days without injection	63 (20)

**TABLE 2:** OAC2805 | Timing of CAB LA PrEP maintenance injections among women with ≥1 maintenance injection (*N* = 210).

	Timing	*n* (%)
All maintenance injections received on‐time	53−67 days between injections	118 (56)
Any maintenance injections received late	68−127 days between injections	92 (44)
Any short delay not requiring reinitiation	68−97 days	82 (89)
Any long delay requiring reinitiation	98−127 days	14 (15)
Discontinuation	>127 days without injection	80 (38)

## OAC4202 Impact of Doxy‐PEP on Sexually Transmitted Infections (STIs) Diagnoses and Antibiotic Use Among Men Who Have Sex With Men and Trans Women on PrEP in Brazil: A Modelling Study

M. Secco Torres da Silva ^1^, R. Ismério Moreira^1^, I. Costa Leite^2^, M. Cunha^2^, M. Benedetti^1^, A. Farias^3^, T. Silva Torres^1^, C. Coutinho^1^, B. Hoagland^1^, V. Gonçalves Veloso^1^, B. Grinsztejn^1^

^1^Fundação Oswaldo Cruz, Instituto Nacional de Infectologia Evandro Chagas, Rio de Janeiro, Brazil; ^2^Fundação Oswaldo Cruz, Escola Nacional de Saúde Pública, Rio de Janeiro, Brazil; ^3^Secretaria do Estado de Saúde da Bahia, Salvador, Brazil

**Background**: In Brazil, syphilis rates are challenging and molecular STI testing for chlamydia (Ct) and gonorrhoea (Ng) has been recently incorporated into the public health system. Doxy‐PEP is a proven strategy for STI prevention and is likely to be implemented shortly. Using data from the ImPrEP study, we modelled the number of STI diagnoses that could be averted under different doxy‐PEP prescribing strategies and estimated the corresponding impact on antibiotic consumption among people on PrEP in Brazil.

**Methods**: ImPrEP study (2018−2021) demonstrated same‐day oral PrEP effectiveness in Brazil, Mexico and Peru, enrolling MSM and TGW aged ≥18 years, HIV‐negative and reporting condomless anal sex, sexual partners with HIV, STI diagnosis or transactional sex. In this sub‐analysis, we included participants from Brazil with ≥2 STI results. Using syphilis, Ct and Ng incidence data, we estimated doxy‐PEP use and averted STI diagnoses under three counterfactual strategies: (S1) initiation at baseline; (S2) initiation after any positive STI test; and (S3) initiation if baseline STI was positive. Averted diagnoses were estimated and number needed to avert one HIV acquisition was calculated. Antibiotic consumption in people on PrEP in Brazil was estimated using country‐specific ImPrEP data, accounting for reduced consumption from averted STI treatments, and for doxy‐PEP doses, assuming 75% doxy‐PEP uptake and median use of four doses/person/month, expressed in monthly Defined‐Daily‐Doses (DDD) per WHO standards.

**Results**: Among the 3447 participants, 100% of them would receive doxy‐PEP under S1 compared to 42.7% under S2 and 23.6% under S3, with an estimated percentage of avoidable STI of 79.4%, 43.9% and 28.6%, respectively. NNT was significantly higher for Ng compared to Ct and syphilis throughout all strategies, and for S1 compared to S2 or S3 across all pathogens. Compared to S1, estimated antibiotic consumption decreased by 58% under S2 and 77% under S3.

**Conclusions**: Our findings point to doxy‐PEP as a promising strategy for STI prevention in Brazil, suggesting that target prescription using incident STI diagnosis as a proxy for higher vulnerability may optimize effectiveness, reduce the NNT and minimize net monthly doxy‐PEP‐related antibiotic consumption compared to universal prescription strategy. These results might inform implementation strategies in Brazil and other Latin American countries (see Table 1).

**TABLE 1:** OAC4202

	Strategy 1			Strategy 2			Strategy 3		
	Chlamydia	Gonorrhoea	Syphilis	Chlamydia	Gonorrhoea	Syphilis	Chlamydia	Gonorrhoea	Syphilis
Averted diagnoses	642	277	698	394	165	335	243	99	241
Person‐year	5536.1	5526.8	6122.3	1941.5	1941.5	2086.5	1295.4	1292.7	1411.8
Number needed to treat (NNT)	9	20	9	5	12	7	6	14	6
Monthly net doses variation (DDD)	400,459			168,761			93,471		

## OAC4203 Substantial Population‐Level Reductions in Syphilis and Chlamydia Among Gay and Bisexual Men After Introduction of doxyPEP Guidance in Australia

M. Traeger ^1,2^, J. Asselin^1,2,3^, H. L. Aung^3^, A. Carter^3,4,5^, A. Wilkinson^1^, V. Cornelisse^3,6,7^, D. Heath‐Paynter^8^, E. Chow^7,9^, J. Ong^7,9^, R. Varma^3,10^, D. Vujcich^11^, C. Tng^12^, J. McCloskey^13^, T. Rees^14^, B. Quinn^1^, B. Haire^3^, M. Hellard^1^, R. Guy^3^, M. Stoové^1,2^

^1^Burnet Institute, Melbourne, Australia; ^2^Monash University, School of Public Health and Preventive Medicine, Melbourne, Australia; ^3^The Kirby Institute, UNSW, Sydney, Australia; ^4^Australian Human Rights Institute, UNSW Sydney, Sydney, Australia; ^5^Simon Fraser University, Faculty of Health Sciences, Burnaby, Canada; ^6^NSW Health, Mid North Coast & Northern NSW Local Health Districts, Australia; ^7^Monash University, School of Translational Medicine, Faculty of Medicine, Nursing and Health Sciences, Melbourne, Australia; ^8^Health Equity Matters, Sydney, Australia; ^9^Melbourne Sexual Health Centre, Melbourne, Australia; ^10^Sydney Sexual Health Centre, Sydney, Australia; ^11^West Australian AIDS Council, Perth, Australia; ^12^Gold Coast Sexual Health Service, Gold Coast, Australia; ^13^Perth Sexual Health Service, Perth, Australia; ^14^SA Health, Adelaide, Australia

**Background**: Doxycycline post‐exposure prophylaxis (doxyPEP) has shown high efficacy in preventing bacterial STIs among gay and bisexual men (GBM) in clinical trials, yet evidence of its population‐level impact is limited. In September 2023, Australia released national clinical guidance recommending doxyPEP for GBM at increased STI risk. We evaluated population‐level changes in bacterial STIs among GBM before and after dissemination of doxyPEP guidance.

**Methods**: We analysed national sentinel surveillance data from 25 sexual health and general practice clinics in the ACCESS network. GBM aged ≥16 years attending services between January 2022 and June 2025 were included. Monthly diagnoses of infectious syphilis, chlamydia and gonorrhoea were analysed using interrupted time‐series regression across three periods: pre‐guidance, evidence dissemination and post‐guidance. Periods were defined by two interruption points: April 2023 (publication of doxyPEP trial evidence and a national doxyPEP interim position statement) and October 2023 (release of the national Consensus Statement providing guidance on doxyPEP use and prescribing). Observed post‐guidance STI trends were compared with counterfactual projections assuming continuation of pre‐guidance trends, and cumulative diagnoses averted were estimated.

**Results**: Among 83,441 GBM tested, 30,637 were diagnosed with at least one STI. During the study period, there were 5712 syphilis cases, 30,070 chlamydia cases and 29,326 gonorrhoea cases. In the pre‐guidance period, syphilis diagnoses increased by 1.1% per month, while chlamydia and gonorrhoea rates remained stable. Following dissemination of national guidance, syphilis diagnoses declined by 1.8% per month and chlamydia diagnoses by 2.8% per month, representing significant departures from pre‐doxyPEP trends. Compared with counterfactual projections, observed cumulative syphilis diagnoses were 46.5% lower (rate ratio [RR] = 0.54; CI = 0.41–0.65) and chlamydia diagnoses were 27.2% lower (RR = 0.73; CI = 0.51–0.94). No significant reduction was observed for gonorrhoea (RR = 1.11; CI = 0.62–1.61). Declines in diagnoses were mirrored by reductions in test positivity, supporting a true reduction in incidence.

**Conclusions**: National dissemination of doxyPEP clinical guidance in Australia was associated with substantial population‐level reductions in syphilis and chlamydia among GBM, but not gonorrhoea. These findings provide the first country‐level evidence that doxyPEP can translate from individual‐level efficacy to measurable public health impact and support its integration as a complementary biomedical STI prevention strategy in high‐income settings (see Figure 1).

**FIGURE 1:**
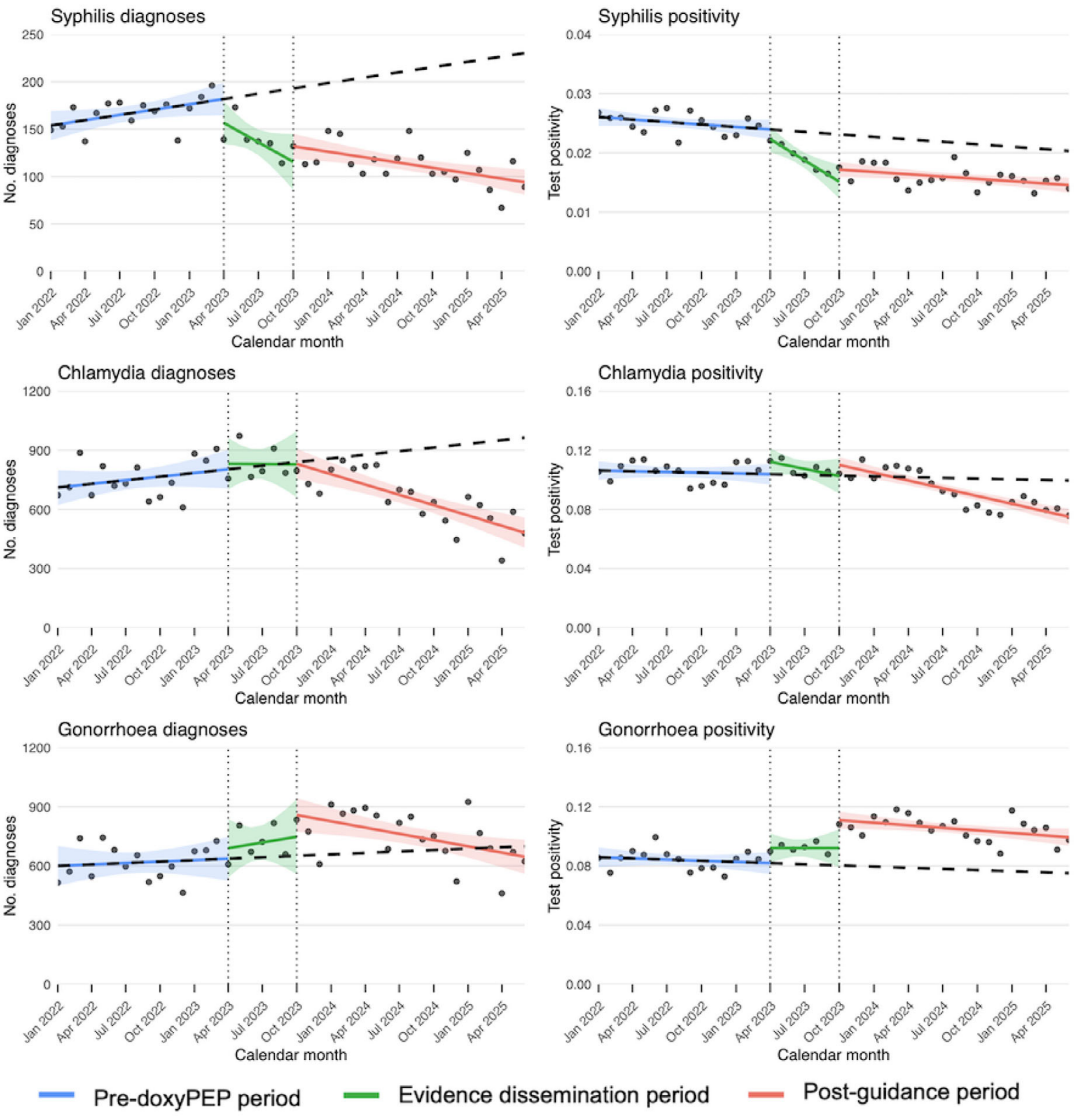
OAC4203 | Monthly number of STI diagnoses and monthly test positivity among gay and bisexual men attending ACCESS clinics from January 2022 to June 2025, with interruptions at April 2023 (doxyPEP trial evidence released) and October 2023 (national doxyPEP guidance released).

## OAC4204 Uptake of Post‐Exposure Prophylaxis With Doxycycline (doxy‐PEP) and Preferred Access to STI Testing and doxy‐PEP Among Gay and Bisexual Men Who Have Sex With Men in Taiwan: 2025 HEART Survey

S. W.‐W. Ku ^1,2^, C.‐W. Li^3^, T.‐T. Chuang^4^, P. Huang^5^, H.‐J. Wu^6^, A. Bourne^7^, C. Strong^4^

^1^Taipei City Hospital Renai Branch, Taipei City, Taiwan, Province of China; ^2^London School of Economics and Political Science, Department of Health Policy, London, United Kingdom; ^3^National Cheng Kung University Hospital, Tainan City, Taiwan, Province of China; ^4^National Cheng Kung University, Tainan City, Taiwan, Province of China; ^5^National Taiwan University, Taipei City, Taiwan, Province of China; ^6^Kirby Institute, University of New South Wales, Sydney, Australia; ^7^Australian Research Centre in Sex, Health and Society, La Trobe University, Melbourne, Australia

**Background**: The WHO has been developing recommendations relating to doxy‐PEP for STI prevention. While its uptake has increased rapidly, many still access this antibiotic via unconventional routes, with or without prior STI testing. The need for differentiated service delivery for doxy‐PEP in this key population remains unknown.

**Methods**: Between December 6th 2025 and January 13th 2026, a survey comprising 83 questions was administered online to adult GBMSM using social networking applications in Taiwan. Beyond demographics, HIV serostatus, risk behaviours and PrEP use, respondents were asked about their awareness, willingness and uptake of biomedical preventive strategies, and preferred accesses to STI testing and doxy‐PEP.

**Results**: In total, 1473 survey responses were included in this analysis (mean age = 34.8 years, SD 8.6). There were 212 respondents living with HIV, 89% of whom reported being undetectable. Among those reported negative or unknown HIV status, 42.7% were currently on PrEP. Awareness of doxy‐PEP was observed among half of the respondents (*n* = 739), with 46.2% expressing willingness to use doxy‐PEP in the coming 6 months. Around one‐fifth (*n* = 324) had ever used doxy‐PEP. Use was more common among those with a significantly higher income, those living in major cities, those who were also currently using PrEP, those with a higher number of male sexual partners and those engaging in condomless anal intercourse. Doxy‐PEP use was also more common among men reporting any STI in the past 12 months as well as those engaging chemsex compared to those never using doxy‐PEP. While hospitals and clinics remained top options that people would like to access STI testing and doxy‐PEP, other routes such as pharmacies, drop‐in centres and online platforms were also popular. A quarter or more wished to have STI testing (24.0%) and doxy‐PEP (34.1%) outside of hospitals and clinics (Table 1). Multivariable logistic regression found older age and current PrEP use were associated with the preference of these unconventional accesses to both STI testing and doxy‐PEP (Table 2).

**Conclusions**: While accessing doxy‐PEP through routes other than hospitals and clinics might raise concerns about safety and increasing antimicrobial resistance, these unconventional channels may help to facilitate STI testing and increase doxy‐PEP uptake.

**TABLE 1:**
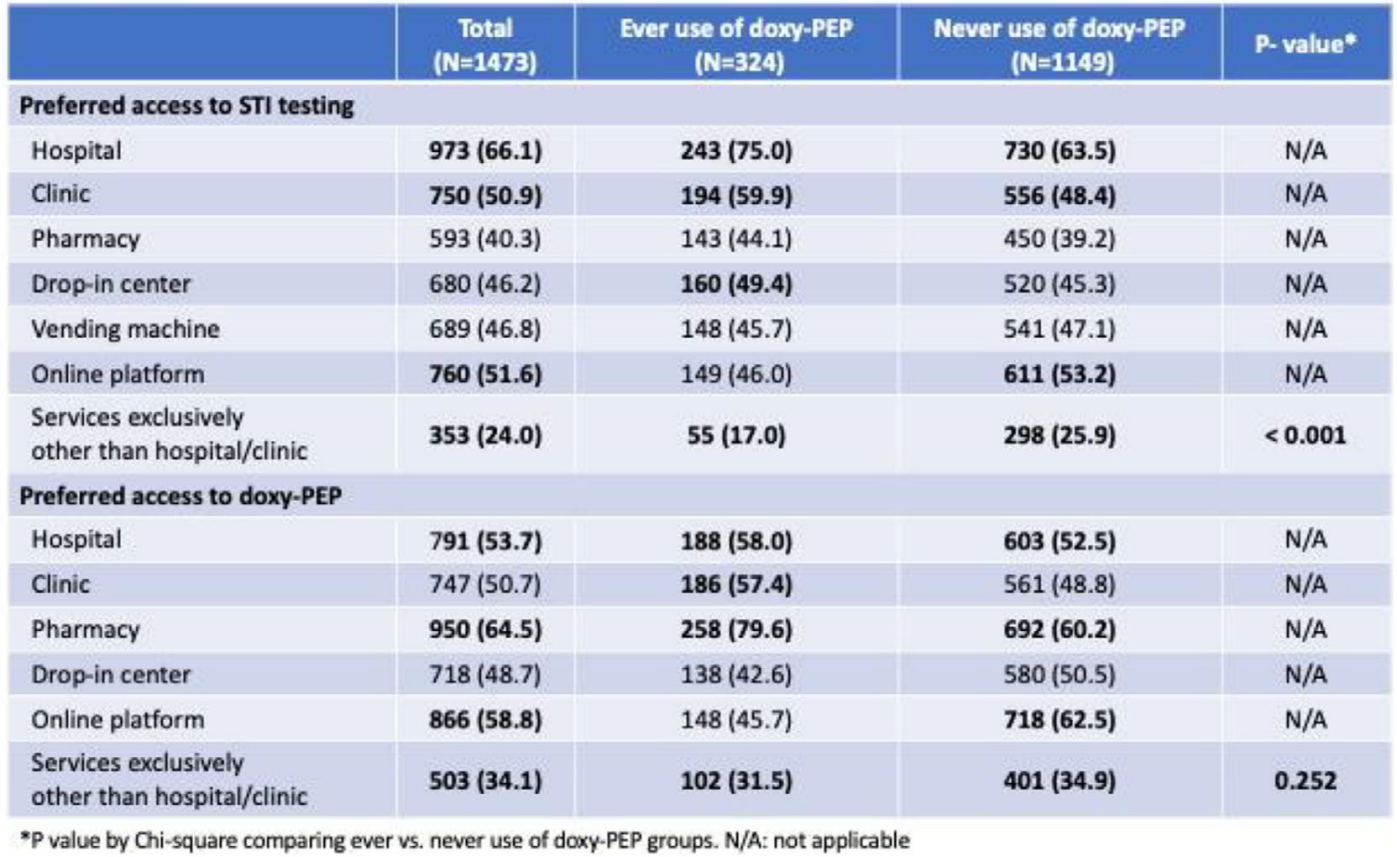
OAC4204 | Preferred access to STI testing and doxy‐PEP.

**TABLE 2:**
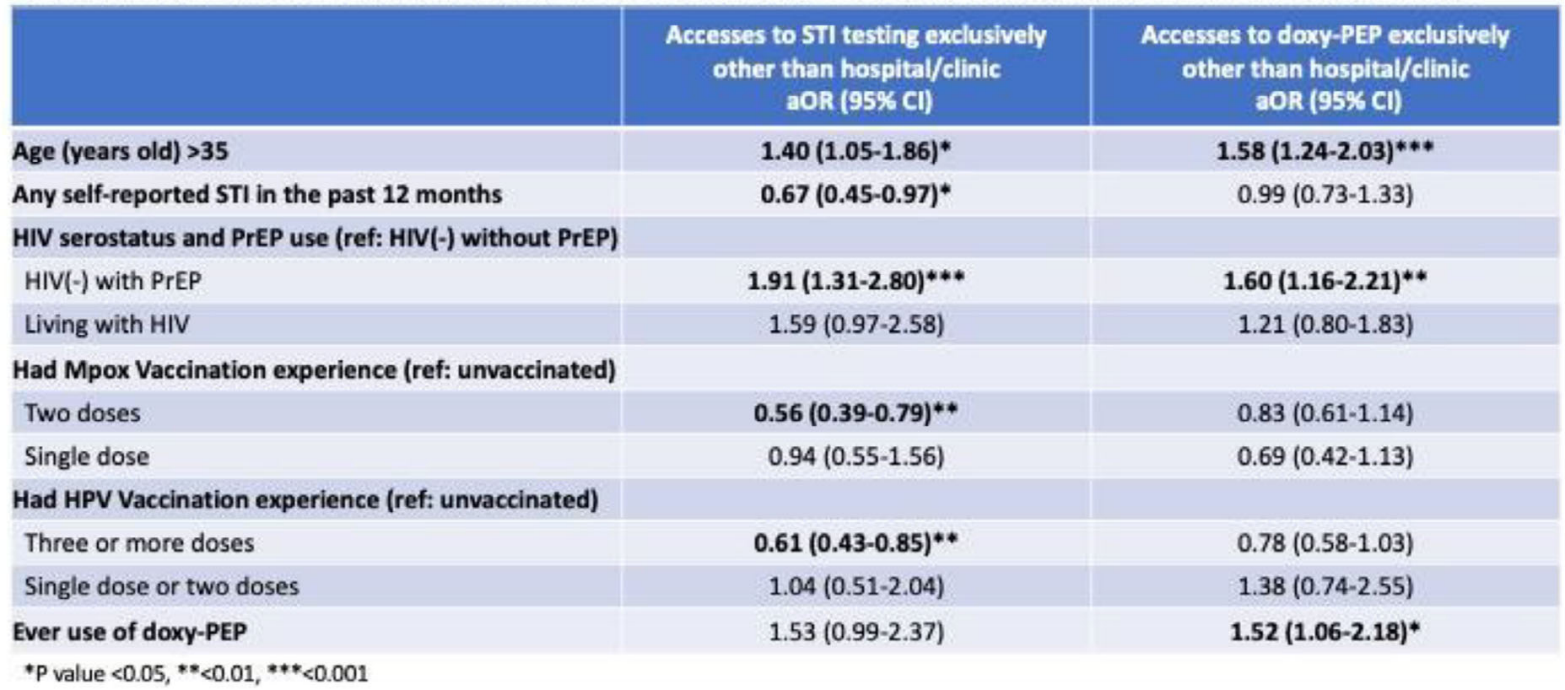
OAC4204 | Factors associated with preferred access exclusively other than hospital/clinic to STI testing and doxy‐PEP.

## OAC4205 Real‐World Effectiveness of Doxycycline Post‐Exposure Prophylaxis for STI Prevention in a Southern U.S. HIV/PrEP Clinic

E. D. Niehaus ^1^, M. S. McKellar^1^, A. Kenny^2^, N. L. Okeke^1^

^1^Duke University, Department of Medicine, Durham, United States; ^2^Duke University, Department of Biostatistics and Bioinformatics, Durham, United States

**Background**: Doxycycline post‐exposure prophylaxis (DoxyPEP) has been shown to reduce cumulative STI incidence among high‐risk men who have sex with men (MSM) and transgender women (TGW) by approximately 60%. STI incidence in North Carolina exceeds the U.S. average by more than 25%, yet real‐world data on DoxyPEP effectiveness in the Southern U.S. are limited. Regional outcomes may differ due to sexual network structure, healthcare access and testing frequency, and background tetracycline resistance patterns. We evaluated real‐world DoxyPEP effectiveness using a pre–post design among individuals prescribed DoxyPEP in a North Carolina HIV/PrEP clinic.

**Methods**: We identified individuals within the Duke University Health System who received HIV or PrEP care and were prescribed DoxyPEP prior to 12/31/2024. STI testing results were abstracted from 1/1/2022 or first clinic encounter through 10/1/2025. STI case positivity was summarized with exact binomial confidence intervals (CI), and incidence rates per 100 person‐years with exact Poisson CIs. Analyses were descriptive pre‐post comparisons, with potential for selection bias. All analyses were conducted in R Studio version 4.4.3.

**Results**: A total of 219 persons received DoxyPEP during the study period. Median age was 37 years; 97% were MSM and 52% were living with HIV. Median follow‐up was 23 months pre‐DoxyPEP and 15 months post‐initiation. Pre‐DoxyPEP cumulative STI case positivity was 8.2% (95% CI 7.2–9.4%), with an incidence rate of 59 infections per 100 person‐years (95% CI 52–68). Following DoxyPEP initiation, case positivity declined to 3.9% (95% CI 3.1–4.9%) with an incidence of 23 infections per 100 person‐years (95% CI 18–30). Incidence reductions were strongest for chlamydia (IRR 0.20, 95% CI 0.11–0.36) and syphilis (IRR 0.33, 95% CI 0.15–0.68), with smaller reductions for gonorrhoea (IRR 0.59, 95% CI 0.40–0.85). STI testing rates decreased modestly, from 7.2 to 5.9 tests per person‐year.

**Conclusions**: In this real‐world cohort from a high‐incidence Southern US HIV/PrEP clinic, DoxyPEP use was associated with substantial reductions in STI incidence, consistent with randomized trial data. Given the high STI burden and unique structural factors in the Southern United States, these findings provide important evidence to inform regional DoxyPEP implementation strategies (see Figure 1).

**FIGURE 1:**
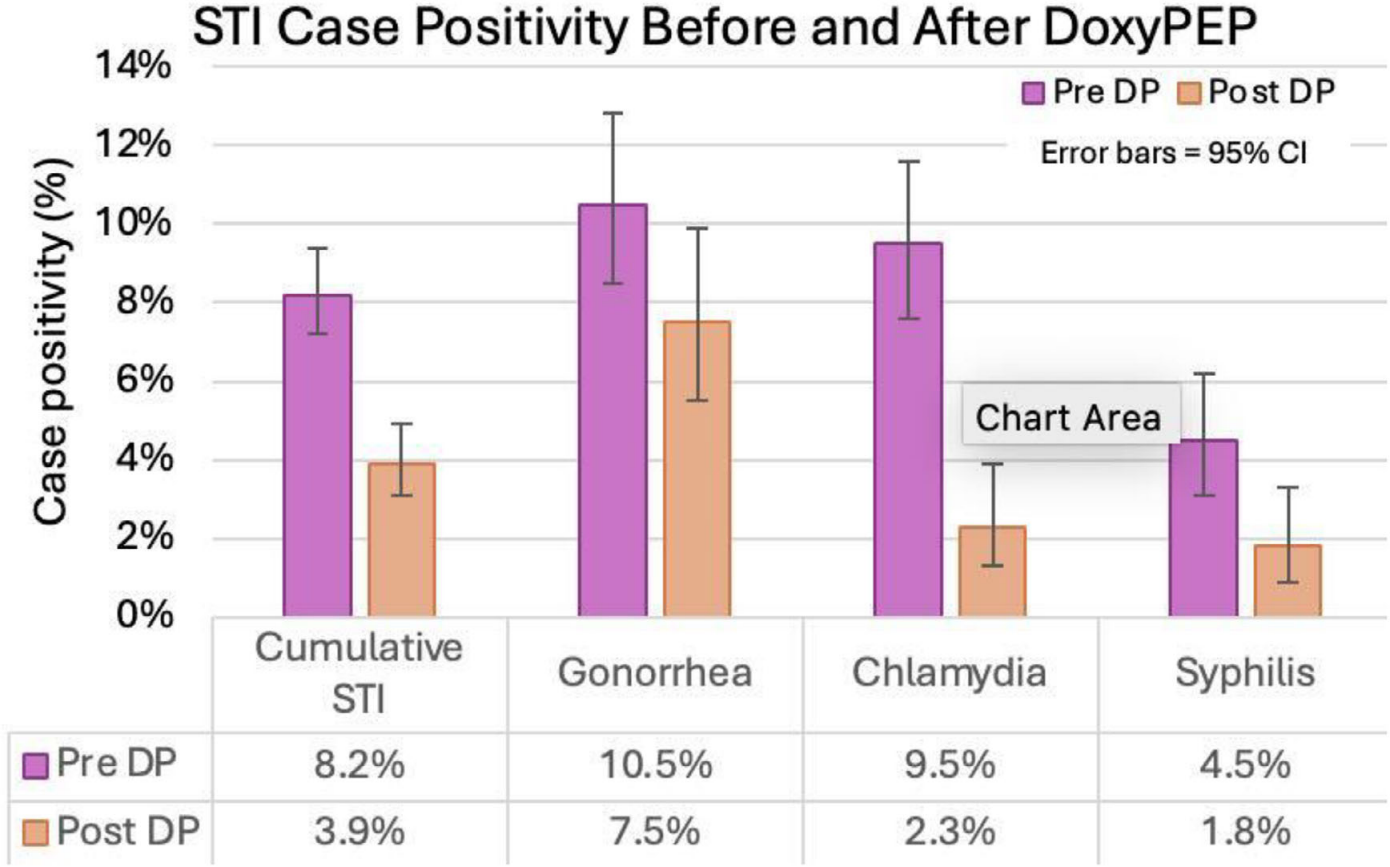
OAC4205 | STI case positivity before and after DoxyPEP.

## OAD0402 When Sexual Health Prevention and Care Ends at the Border: Cross‐Border Sexual Mobility, HIV and STI Burdens, and PrEP Cascades Among Young Men Who Have Sex With Men in Southeast Asia

H. Wang ^1^, Y. Kongjaroen^2^, W. Waratworawan^2^, S. H. Lim^3^, C. Wei^4^, K. Jonas^1^, T. E. Guadamuz^2^

^1^Maastricht University, Work and Social Psychology, Maastricht, the Netherlands; ^2^Mahidol University, Mahidol Center for Health, Behavior and Society, Faculty of Tropical Medicine, Bangkok, Thailand; ^3^University of Malaya, Department of Social and Preventive Medicine, Faculty of Medicine, Kuala Lumpur, Malaysia; ^4^Rutgers School of Public Health, Department of Health Behavior, Society, and Policy, New Brunswick, United States

**Background**: Cross‐border mobility is an important, yet overlooked, structural determinant of HIV and STI transmission in Southeast Asia. Sexual health prevention and care systems, however, remain fragmented by national borders. Young MSM increasingly travel across borders, often in sexualized contexts, yet empirical evidence linking cross‐border mobility/travel, sexual behaviours and prevention gaps in the region remains limited.

**Methods**: We analysed data from a multi‐country, web‐based cross‐sectional survey among 3007 young MSM aged <25 years in Cambodia, Lao PDR, Malaysia and Thailand. Recent cross‐border travel, sexual behaviours during travel (CAI, sex tourism, sex work and chemsex) were investigated. HIV status, recent STIs and PrEP awareness, uptake, and discontinuation were compared by travel status and sexual behaviours. Determinants for these sexual behaviours during cross‐border travel were explored using multivariable logistic regression.

**Results**: Overall, 16.3% reported recent cross‐border travel. Flows were highly structured: Thailand served as the primary regional hub for travellers from Cambodia, Lao PDR and Malaysia across all sexual behaviours, while Thai travellers reported dispersed destinations (Figure 1). Among travellers, 31.4% reported at least one selected sexual behaviour during travel, including CAI (24.7%), sex tourism (7.2%), sex work (6.1%) and chemsex (5.4%). Compared with non‐travellers (Figure 2), those reporting these sexual behaviours during travel had higher HIV prevalence (2.8% vs. <1%), recent syphilis (5.6% vs. 1.4%) and viral hepatitis (HBV and HCV each 2.8% vs. <1%). Although HIV status awareness was higher among these travellers, their current PrEP uptake remained negligible (6.1%), yet discontinuation was common (20.0%). Reporting these sexual behaviours during travel was associated with full‐time employment (aOR = 2.75, 95% CI = 1.39−5.56), having more sex partners (aOR = 2.00, 95% CI = 1.16−3.47), experiences of violence or bullying (aOR = 2.30, 95% CI = 1.29−4.12) and considering religion less important (aOR = 0.26, 95% CI = 0.10−0.65).

**Conclusions**: Cross‐border mobility among young MSM in Southeast Asia is sizable, patterned and associated with increased HIV, STI and viral hepatitis burdens, alongside substantial gaps in PrEP uptake and persistent use. The disconnect between mobility and static, nationally bounded prevention systems creates a “prevention void” for the most vulnerable, particularly those driven by economic necessity or social stigma. Addressing these determinants requires transnational structural interventions, including cross‐border PrEP access, HIV/STI services and harm reduction tailored to mobile populations.

**FIGURE 1:**
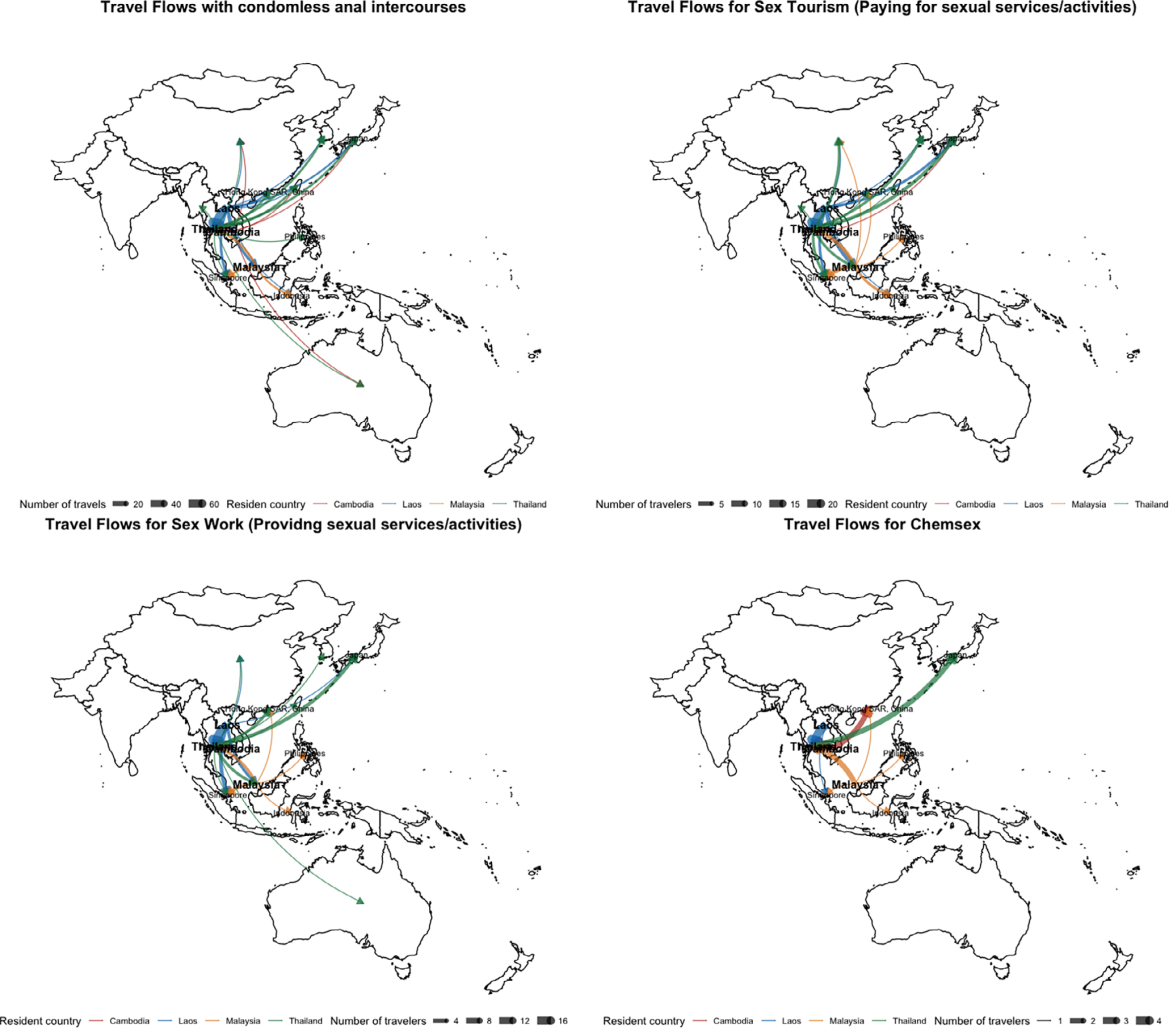
OAD0402 | Cross‐border travel flows with condomless anal intercourse, travel flows for sex tourism, travel flows for sex work and travel flows for chemsex among young MSM living in Cambodia, Laos, Malaysia and Thailand in the WHO Asia‐Pacific Region.

**FIGURE 2:**
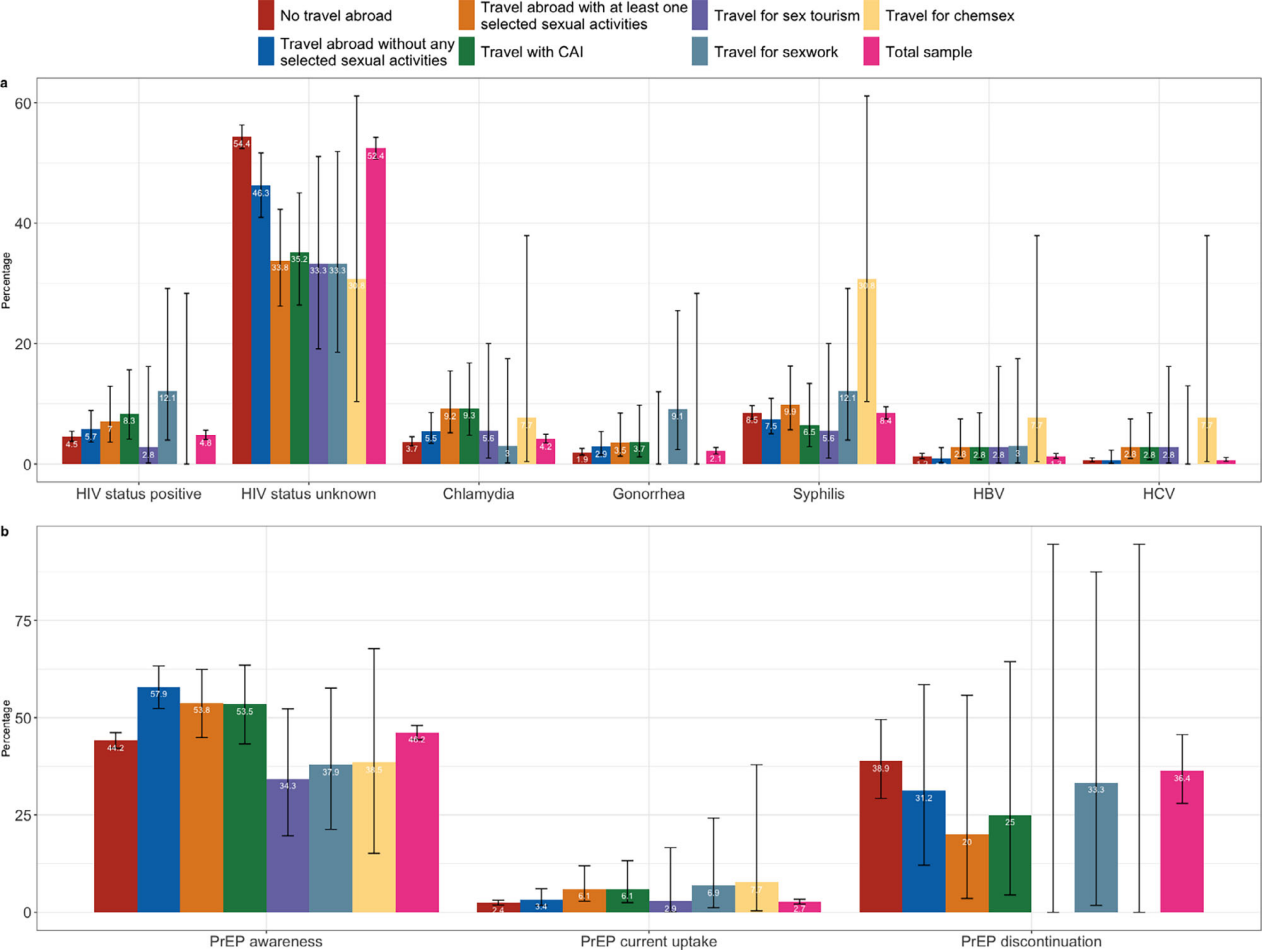
OAD0402 | Self‐reported (a) HIV status and recent STIs, and (b) PrEP cascades among young MSM by their cross‐border travel activities and sexual behaviours during travels.

## OAD0403 Life‐Course Trajectories of Migration, Transgender Visibility and Transactional Sex Involvement in Relation to HIV Diagnosis Among Trans Women Living With HIV in France (ANRS‐Trans&HIV)

M. Annequin ^1^, M. Mora^1^, R. Van Huizen^1^, A. Faye^1^, M. Fiorentino^1^, C. Protière^1^, M. Bourrelly^1^, G. Maradan^2^, C. Berenger^2^, F. Michard^3^, Y. Yazdanpanah^3^, A. Freire Maresca^4,5^, E. Rouveix^4^, M. Costa^6,7^, D. Michels^6,7^, L. Blanquart^8^, G. Rincon^8^, B. Spire^1^, the ANRS‐Trans&HIV study group

^1^Aix Marseille Univ, Inserm, IRD, SESSTIM, Sciences Economiques & Sociales de la Santé & Traitement de l'Information Médicale, ISSPAM, Marseille, France; ^2^ORS PACA, Southeastern Health Regional Observatory, Marseille, France; ^3^Service de Maladies Infectieuses, Hôpital Bichat – Claude‐Bernard, APHP, Paris, France; ^4^Service de Médecine Interne, UFR Paris Île‐de‐France Ouest, Hôpital Ambroise‐Paré, Boulogne‐Billancourt, France; ^5^Service de Maladies Infectieuses et Tropicales, Hôpital Avicenne, APHP, Bobigny, France; ^6^Laboratoire de Recherche Communautaire, Coalition PLUS, Pantin, France; ^7^AIDES, Paris, France; ^8^ACCEPTESS‐T, Paris, France

**Background**: Information is limited regarding the context of HIV diagnosis among transgender women living with HIV, who are disproportionately marked by migration and transactional sex in France. This study aimed to analyse the timing and sequencing of migration, involvement in transactional sex and visibility of transgender identity in relation to HIV diagnosis, and examine how these life‐course trajectories are associated with social risk factors for HIV acquisition.

**Methods**: ANRS‐Trans&HIV (2020−2022) was a cross‐sectional, nationwide, retrospective survey conducted in France. Community‐based interviewers administered face‐to‐face questionnaires to 536 transgender women living with HIV, recruited from 36 HIV care units. Multidomain sequence analysis used life‐event history calendars to identify retrospective life‐course trajectories for the 11‐year period surrounding HIV diagnosis, using three dimensions: country of residence, involvement in transactional sex and visibility of transgender identity. Zero‐inflated Poisson regressions were used to assess associations between trajectory typologies and the cumulative number of social risk factors for HIV acquisition (housing precarity, relationship dissolution and experiences of violence).

**Results**: Among 423 participants with complete data, the median age was 45 years (IQR: 37–51), 85% had foreign citizenship and the median age at HIV diagnosis was 28 years (IQR: 24−33). Sequence analysis identified six trajectories surrounding HIV diagnosis (Figure 1). In the year preceding or the year of HIV diagnosis, 52% experienced housing precarity, 23% violence and 18% relationship dissolution, resulting in 46% of participants experienced one social risk factor, 20% experienced two and 2.6% experienced three. Regression models showed significant associations between trajectories involving migration, transactional sex and transgender visibility (Cl3, Cl4, Cl5) and the cumulative number of social risk factors for HIV acquisition (Table 1).

**Conclusions**: With regard to timing and sequencing, HIV diagnosis most often occurred after the visibility of transgender identity, but not systematically in the context of transactional sex, and occurred at various stages of migration. However, transgender women with a history of migration, visible as transgender and engaged in transactional sex around the time of HIV diagnosis faced a higher accumulation of social risk factors. These findings highlight the importance of accounting for intersecting life‐course vulnerabilities when designing HIV‐prevention strategies for transgender women.

**FIGURE 1:**
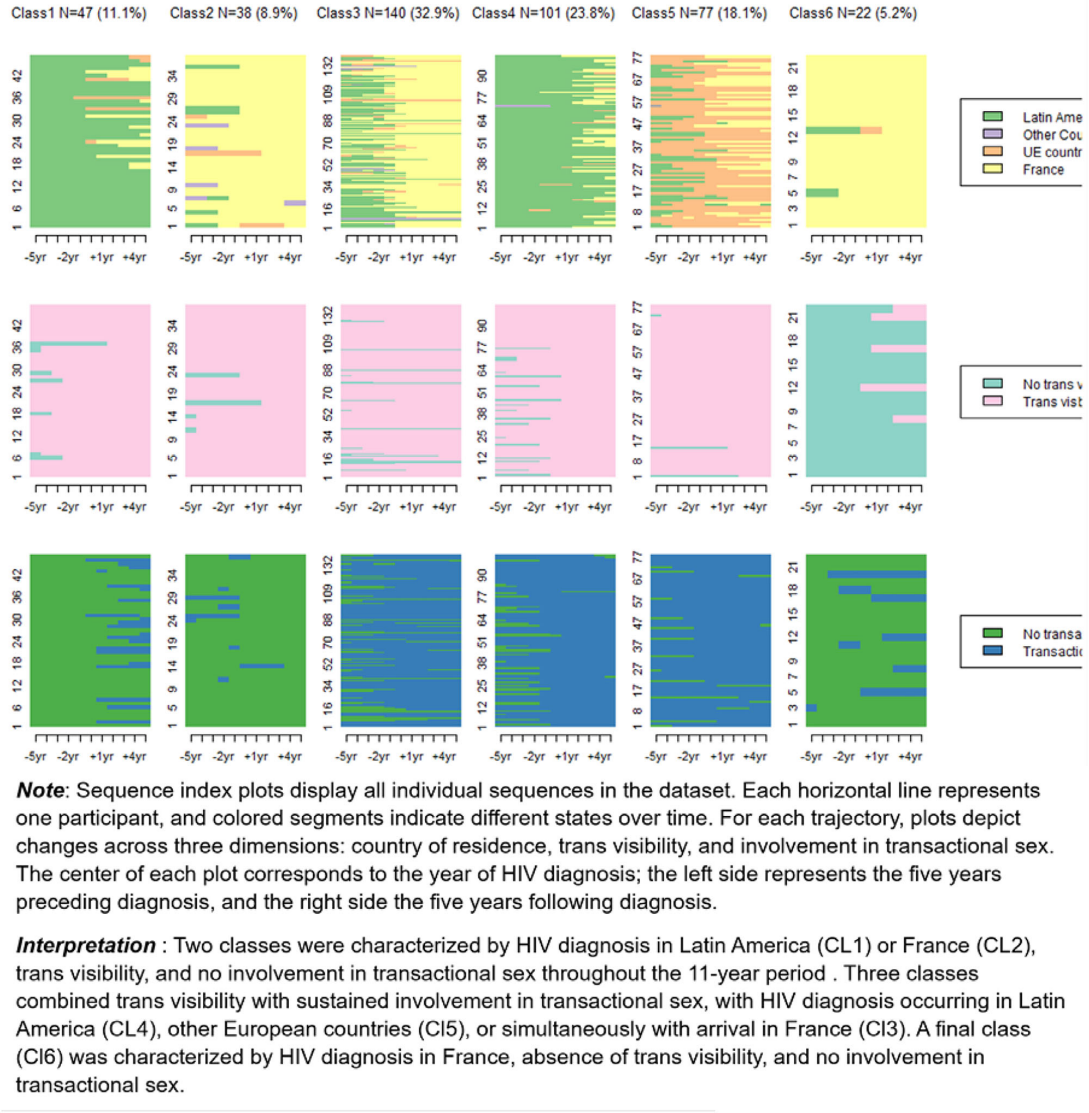
OAD0403 | Sequence index plots of trajectories of (1) country of residence, (2) trans visibility and (3) involvement in transactional sex over an 11‐year period centred on HIV diagnosis, by trajectory typology.

**TABLE 1:** OAD0403 | Zero‐inflated Poisson model of cumulative social risk factors for HIV acquisition (housing precarity, relationship dissolution and experiences of violence) in the year preceding or the year of HIV diagnosis (*N* = 423)—ANRS‐Trans&HIV.

	IRR	95% CI	*p*‐value
**Trajectory classes**			
Cl.6—DiagFr‐NoTransVi‐NoTransacSex	—	—	0.009
Cl.1—DiagLA‐TransVisible‐NoTransacSex	1.71	0.81, 3.63	
Cl.2—DiagFR‐TransVisible—NoTransacSex	1.65	0.78, 3.52	
Cl.3—Diag‐FRArrival‐TransVisible—TransacSex	2.49	1.26, 4.92	
Cl.4—DiagLA‐TransVisible—TransacSex	2.14	1.06, 4.31	
Cl.5—DiagEU‐TransVisible—TransacSex	2.31	1.15, 4.64	
**Age**			
[23−35]	—	—	0.051
[36−45]	1.20	0.87, 1.64	
[46−55]	1.29	0.90, 1.83	
[56−74]	0.98	0.61, 1.58	
**Administrative status**			
Legal residency status	—	—	0.94
Undocumented	0.92	0.72, 1.17	
**Date of HIV diagnosis**			
[1984, 1995]	—	—	0.60
[1995, 2006]	1.17	0.79, 1.73	
[2006, 2021]	1.24	0.81, 1.89	

Abbreviation: CI = confidence interval, IRR: incidence rate ratios from the count component of a zero‐inflated Poisson model, DiagLA: HIV diagnosis in Latin America, TransVisible: Visible as a transgender woman; TransacSex: Involvement in transactional sex.

## OAD0404 Fragmented Antiretroviral Therapy Access for Gay and Bisexual Colombian and Venezuelan Migrant Men Living With HIV in Colombia's Two‐Tier Public Healthcare System: An Exploratory Qualitative Study

J. Brisson ^1,2^, M. Castro‐Arteaga^2^, Y. Vélez Agudelo^3^, A. Zea^3^, A. Perez‐Brumer^2^

^1^University of Montreal, Faculty of Pharmacy, Montreal, Canada; ^2^University of Toronto, Dalla Lana School of Public Health, Toronto, Canada; ^3^Mas Que Tres Letras, Medellin, Colombia

**Background**: Venezuela's humanitarian crisis has generated Latin America's largest mass migration, with Colombia hosting the largest number of displaced Venezuelans. Colombia's two‐tier public healthcare system—comprised of a contributory regime for formal workers and a subsidized regime for low‐income or uninsured populations—creates structurally differentiated pathways to HIV care. However, little is known about how this stratification shapes comparative HIV care experiences for migrants and citizens. This study examined differences in the HIV care continuum between gay and bisexual Colombian men and Venezuelan migrant men living with HIV, with specific attention to how system structure facilitates or impedes antiretroviral therapy (ART) access and continuity.

**Methods**: From October 2024 to April 2025, we conducted 66 semi‐structured interviews with gay and bisexual Colombian men (*n* = 40) and Venezuelan migrant men (*n* = 26) living with HIV across Colombia. Recruitment was facilitated by an HIV community‐based organization. Interviews explored participants’ navigation of the healthcare system, initiated ART and experienced continuity or disruptions in ART care. Data were analysed using inductive thematic analysis to identify patterns related to ART access within the two‐tier public health system.

**Results**: Participants had a mean age of 31.5 years (range: 19−60) and an average year of HIV diagnosis of 2020. Participants covered under the contributory regime through formal employment—regardless of nationality—reported expedited ART initiation and consistent access across the HIV care continuum. In contrast, participants enrolled in the subsidized regime, or those who moved between regimes due to employment instability or socioeconomic precarity (mostly Venezuelan migrants) described more fragmented care trajectories. These included prolonged administrative procedures for activating or transferring coverage, periodic delays in ART dispensation and recurrent interruptions in continuity of care.

**Conclusions**: Although Colombia has established important mechanisms to extend healthcare coverage to Venezuelan migrants, the persistent divide between the contributory and subsidized regimes continues to generate inequitable HIV care experiences for both migrants and Colombian citizens. System stratification undermines timely and consistent ART access for anyone reliant on the subsidized regime, highlighting the need for policy reforms that harmonize administrative processes, strengthen continuity of care and reduce regime‐based disparities in HIV treatment across all populations.

## OAD0405 Borderless Digital Outreach to Reach Migrant Sex Workers and Clients Through Language‐Specific Social Media During Cross‐Border Tension

K. H. Thy ^1^, S. Srichau^2^, S. Janyam^3^, C. Phaengnongyang^1^, S. Sumalu^1^

^1^Service Worker In Group Foundation, Bangkok, Thailand; ^2^Chulalongkorn University, Bangkok, Thailand; ^3^Srinakharinwirot University, Bangkok, Thailand

**Background**: Migrant sex workers and their clients in Thailand face persistent barriers to HIV prevention and care, including language gaps, mobility, legal precarity, stigma and fear of visibility. These challenges intensify during periods of cross‐border political tension, when misinformation and restricted movement further limit access to trusted health information. Innovative, culturally relevant outreach models are needed to sustain HIV responses for mobile and marginalized populations.

**Description**: This programme implemented a borderless outreach model targeting migrant sex workers and migrant clients, primarily from Cambodia, working in Thailand. Conducted in urban and border‐adjacent settings, the intervention combined community‐informed outreach with digital communication using TikTok. Short‐form videos in the Cambodian language delivered HIV, STI, harm reduction and service‐access messages using peer‐informed narratives. Content prioritized safety, neutrality and public health during periods of heightened Thailand−Cambodia tension, avoiding political messaging while maintaining continuity of care and referral pathways.

**Lessons Learned**: Language‐specific, migrant‐informed digital outreach significantly improved engagement with populations otherwise unreachable through conventional services. TikTok proved effective in delivering discreet, culturally resonant messages to both sex workers and clients. During periods of political tension, neutral and care‐focused communication helped maintain trust and reduce fear. Community‐informed content creation strengthened credibility, while digital platforms enabled continuity despite physical and structural barriers. Borderless outreach requires flexibility, risk awareness, and constant adaptation to social and political contexts.

**Conclusions/Next Steps**: Borderless, migrant‐led digital outreach offers a resilient and scalable approach to sustaining HIV prevention and information access for mobile key populations, even during crises and cross‐border instability. Integrating language‐specific social media strategies into HIV programmes can reduce exclusion and strengthen continuity of care. Future steps include strengthening referral linkages, documenting outcomes, and expanding the model to other migrant communities and regional contexts.

## OAD0802 Experiences With Twice‐Yearly Lenacapavir for PrEP in the PURPOSE 1 Open‐Label Extension Phase

T. Nkosi ^1^, A. Nyamaizi^2^, I. Hawley^2^, N. Mosery^3^, J. Smit^3^, K. Gill^1^, C. Milford^4^, A. Kubeka^5^, A. Tlagadi^5^, Z. Njengele‐Tetyana^6^, P. Sibiya^6^, T. Palanee‐Phillips^6,7^, A. Kintu^8^, C. C. Carter^8^, E. T. Montgomery^2,9^

^1^The Desmond Tutu HIV Centre, University of Cape Town, Cape Town, South Africa; ^2^RTI International, Research Triangle Park, NC, United States; ^3^Wits MatCH Research Unit, School of Obstetrics and Gynecology, Faculty of Health Sciences, University of the Witwatersrand, Durban, South Africa; ^4^Centre for the AIDS Programme of Research in South Africa (CAPRISA), Vulindlela, South Africa; ^5^The Aurum Institute, Rustenburg Clinical Research Centre, Rustenburg, South Africa; ^6^Wits Reproductive Health and HIV Institute, Faculty of Health Sciences, University of the Witwatersrand, Johannesburg, South Africa; ^7^Department of Epidemiology, School of Public Health, University of Washington, Seattle, WA, United States; ^8^Gilead Sciences, Inc., Foster City, CA, United States; ^9^Department of Epidemiology and Biostatistics, School of Medicine, Univ of CA, San Francisco (UCSF), San Francisco, CA, United States

**Background**: Following demonstration of 100% efficacy of twice‐yearly subcutaneous lenacapavir in preventing HIV‐1 among adolescent girls and young women (AGYW) in the PURPOSE 1 study (NCT04994509), participants were offered the choice to continue receiving lenacapavir in an open‐label extension (OLE) phase. More than 95% of participants chose this option, while the remainder received open‐label daily oral pre‐exposure prophylaxis (PrEP) or discontinued study participation. In this ancillary study, we qualitatively assessed the decision‐making for this PrEP method choice, and attitudes and experiences with lenacapavir during the OLE phase.

**Methods**: Single in‐depth interviews were conducted with 85 randomly or purposively selected AGYW from five South African PURPOSE 1 study sites. Interviews occurred after participant unblinding at OLE onset or within ∼1−3 months of OLE initiation. Interviews were translated, transcribed, coded and analysed thematically.

**Results**: In the OLE phase, the demonstrated high efficacy of lenacapavir strengthened participants’ confidence in its use, increasing the perceived value of its convenient twice‐yearly administration. Participants described lenacapavir as dependable, discreet and trustworthy. OLE phase participants highlighted lenacapavir's long‐acting protection and practicality, noting that it integrated smoothly into their routines, unlike oral PrEP, which was “boring” (tedious) to use daily. Lenacapavir offered discreet continuous protection and aligned with the active lifestyles of AGYW. Injection‐site reactions were common but generally short‐lived and viewed as an acceptable trade‐off for long‐acting protection. The introduction of pain‐management measures, including using ice compress pre‐ and post‐injection, substantially improved the injection experience. Decision‐making regarding the preferred PrEP modality following study unblinding was described as autonomous: most participants continued lenacapavir independently of partner, peer or family input. AGYW's anticipated future interest in lenacapavir use varied by life stage, with consistent interest anticipated while dating and mixed views regarding expected use during marital and perinatal periods. Participants expressed interest in the use of more familiar anatomic injection sites (upper arm and buttocks). Of those who discontinued, many still expressed willingness to recommend lenacapavir to others.

**Conclusions**: In the post‐trial context of known efficacy, lenacapavir was widely appreciated and chosen as the preferred PrEP option for its demonstrated high efficacy, convenience and alignment with the active lifestyles of AGYW.

## OAD0803 Endorsement and Anticipated Challenges to Adopting Twice‐Yearly Lenacapavir for HIV Pre‐Exposure Prophylaxis (PrEP) Among Cisgender Men Who Have Sex With Men: Qualitative Insights From the PURPOSE 2 Trial

S. Meanley ^1,2^, L. Listerud^1,2^, A. Richards^1,2^, B. Kosciow^1,2^, D. Watson^2,3^, I. Frank^2,3^, V. D. Cantos^4^, S. Doblecki‐Lewis^5^, L. B. Brown^6^, J. C. Hojilla^6^, C. Carter^6^, J. A. Bauermeister^1,2^

^1^University of Pennsylvania School of Nursing, Family and Community Health, Philadelphia, United States; ^2^University of Pennsylvania School of Nursing, Eidos LGBTQ+ Health Initiative, Philadelphia, United States; ^3^University of Pennsylvania Perelman School of Medicine, Infectious Diseases, Philadelphia, United States; ^4^Emory University School of Medicine, Infectious Diseases, Atlanta, United States; ^5^University of Miami Miller School of Medicine, Infectious Diseases, Miami, United States; ^6^Gilead Sciences, Inc., Foster City, United States

**Background**: Next‐generation PrEP formulations are critical to support prevention‐priority communities overcome common engagement barriers observed when taking other PrEP modalities. The PURPOSE 2 trial (NCT04925752) demonstrated efficacy of twice‐yearly subcutaneous lenacapavir for PrEP in cisgender men and gender‐diverse individuals. We qualitatively explored acceptability of lenacapavir among a racially/ethnically diverse sample of adult cisgender MSM from PURPOSE 2.

**Methods**: PrEP‐indicated MSM in US‐based, high‐incidence jurisdictions completed three sequential in‐depth virtual interviews (IDIs) during the randomized blinded phase (IDI1: before first injection [04/2023−12/2023]; IDI2 and IDI3: after second and third injection [01/2024−01/2025] of lenacapavir or placebo). IDIs explored participants’ attitudes, experiences and anticipated challenges of taking lenacapavir post‐trial, including comparisons with other available modalities (daily oral or bimonthly long‐acting injectable PrEP). Transcripts were analysed cumulatively and thematically across IDIs.

**Results**: Participants were predominantly Black or Hispanic (∼90%). Most endorsed a strong, durable preference for lenacapavir versus daily oral and bimonthly formulations, highlighting an eliminated pill burden, high confidence in the 6‐month protection window and reduced clinic visits. One stated, “I would recommend [lenacapavir]…not that taking pills is time‐consuming, but remembering to do so on a daily basis can be a challenge.” Across IDIs, injection side effects (nodules, pain) were described as manageable. Few participants cited injection‐site pain as the reason for preferring daily oral PrEP, but most described the pain as transient, improved at subsequent visits and acceptable given the convenience of the twice‐yearly regimen. There was high enthusiasm for choosing lenacapavir outside of the study and recommending it to peers. Many emphasized using lenacapavir post‐trial would depend on insurance or costs. One stated, “I would love to get on [lenacapavir], but I don't know financially. If the difference is too much, [I'd] rather take the pills.”

**Conclusions**: PURPOSE 2 MSM largely preferred twice‐yearly PrEP. Many emphasized lenacapavir's benefits (protection window and reduced clinic visits) outweighing time‐limited side effects, highlighting its potential to support MSM whose prevention needs are unmet by alternative formulations. Insurance and access were anticipated challenges for lenacapavir's real‐world implementation pre‐approval. Addressing access concerns will be important to maximize the long‐term impact of lenacapavir on HIV incidence among MSM from disproportionately affected communities. (Table 1)

**TABLE 1:**
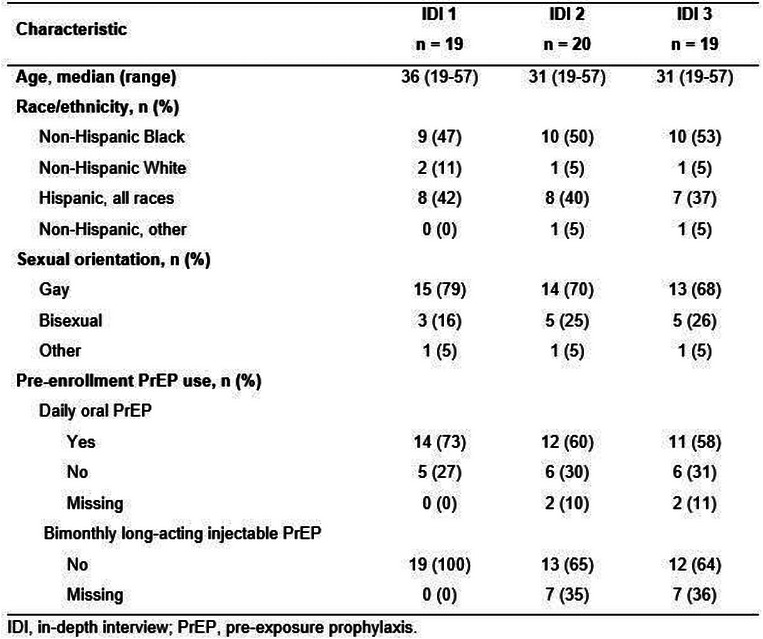
OAD0803 | Participant characteristics by IDI time point in PURPOSE 2.

## OAD0804 “We Want It, but Will It Reach Us?”: Community‐Driven Insights on Acceptability, Feasibility and Barriers to Twice‐Yearly Lenacapavir for HIV Prevention in Nakivale Refugee Settlement, Uganda—A Qualitative Exploration

U. Muhumuza^1^, E. Surafel Abay^2^, Z. Nahumuza ^3^, B. Nanyange^4^

^1^Africa CDC, Kampala, Uganda; ^2^Africa CDC, Addis Ababa, Ethiopia; ^3^International University of Africa, Khartoum, the Sudan; ^4^Mbarara University of Science and Technology, Mbarara, Uganda

**Background**: Twice‐yearly subcutaneous lenacapavir (LEN) represents a breakthrough in HIV pre‐exposure prophylaxis (PrEP), demonstrating near‐100% efficacy in PURPOSE 1 (0 acquisitions among cisgender women) and 96% overall reduction versus daily oral PrEP in pivotal trials, leading to FDA approval in 2025 and Uganda's regulatory nod in early 2026. In humanitarian contexts like Nakivale—one of Africa's largest refugee settlements hosting over 170,000 displaced people amid conflict, mobility limits and stigma—no evidence yet exists on its real‐world acceptability or implementation challenges, despite its potential to overcome daily pill burdens in high‐risk groups.

**Methods**: Between April and August 2025, we conducted 12 focus group discussions (*n* = 106) and 31 in‐depth interviews in Nakivale Refugee Settlement, Uganda. Purposive maximum‐variation sampling captured diverse voices: adolescents, pregnant women, sex workers, men who have sex with men, community leaders, health workers and policymakers. Data were thematically analysed using the Theoretical Framework of Acceptability, with co‐design elements embedded throughout.

**Results**: Participants hailed LEN as “life‐changing,” praising 6‐monthly dosing for eliminating daily adherence struggles, visible pill bottles and associated stigma—bolstered by trust in injectables from prior contraceptive experiences. Key facilitators included peer‐led education and mobile outreach. Dominant barriers encompassed fears of side effects without prompt care access, conflict‐driven mobility restrictions, nationality‐based discrimination and historical mistrust of novel injections. Co‐created solutions emerged: village‐based administration by community health workers, emergency transport funds and nationality‐blind registration to ensure equity.

**Conclusions**: Twice‐yearly lenacapavir holds transformative promise for crisis‐affected populations, where structural and cultural barriers hinder conventional PrEP. This first humanitarian acceptability study directly informed Uganda's 2026 national LEN introduction strategy, emphasizing community co‐creation for equitable rollout. Findings urge global humanitarian HIV programmes to prioritize refugee‐led adaptations—potentially accelerating progress towards ending AIDS by bridging prevention gaps in overlooked settings.

## OAD0805 Multilevel Social Influences on PrEP Modality Choice and Adherence Among a Subset of Persons Enrolled in HPTN083, an International Injectable PrEP Trial

Y. Chen^1^, R. J. Landovitz^2^, E. M. Waldron^3^, W. Rice^4^, C. F. Kelley^4^, T. Oyedele^5^, L. Coelho^6^, N. Phanuphak^7^, Y. Singh^8^, K. Middelkoop^9^, M. McCauley^10^, Z.‐M. R. Aoun^3^, N. Lawrence^9^, T. Jupimai^11^, J. Rooney^12^, A. Rinehart^13^, J. Clark^2^, V. Go^14^, J. Sugarman^15^, S. Fields^16^, L. Soto‐Torres^17^, B. Grinsztejn^6^, S. A. Safren^1^, C. Psaros ^3^

^1^University of Miami, Psychology, Coral Gables, United States; ^2^University of California, Los Angeles, Los Angeles, United States; ^3^Mass General Hospital/Harvard Medical School, Boston, United States; ^4^Emory University, Atlanta, United States; ^5^AstraZeneca, N/A, United States; ^6^The Oswaldo Cruz Foundation, Rio de Janeiro, Brazil; ^7^Institute of HIV Research and Innovation (IHRI), Bangkok, Thailand; ^8^University of Cape Town, City of Cape Town, South Africa; ^9^Desmond Tutu HIV Center, City of Cape Town, South Africa; ^10^HIV Prevention Trials Network (HPTN) Leadership and Operations Center, FHI 360, Durham, United States; ^11^Chulalongkorn University, Bangkok, Thailand; ^12^Gilead Sciences, San Mateo, United States; ^13^ViiV Healthcare, Chapel Hill, United States; ^14^University of North Carolina at Chapel Hill, Chapel Hill, United States; ^15^Johns Hopkins University, Baltimore, United States; ^16^The Pennsylvania State University, University Park, United States; ^17^The National Institutes of Health, Rockville, United States

**Background**: Pre‐exposure prophylaxis (PrEP) has the potential to reduce new HIV acquisitions, yet effectiveness depends on uptake and adherence when HIV acquisition is possible. While social factors, particularly social support and stigma, have been shown to influence PrEP initiation and adherence, less is known about how these factors may inform PrEP modality choice and modality‐specific adherence. Guided by the Socio‐Ecological Model of Health (SEMH), we explored these socially driven pathways across intrapersonal, interpersonal and institutional levels (i.e. clinics).

**Methods**: HPTN083 demonstrated superiority of long‐acting cabotegravir (CAB‐LA) over oral PrEP. Post‐unblinding interview data from a subset of participants from two U.S. and three international sites were analysed (*N* = 150). Participants were grouped by post‐unblinding modality choice (oral or CAB‐LA; Table 1); the constant comparative method identified socially driven themes influencing PrEP modality choice and adherence across SEMH levels.

**Results**: Both intrapersonal and interpersonal factors shaped PrEP modality choice. Internalized stigma and disclosure concerns drove preferences for CAB‐LA as a more discreet prevention strategy. Although oral PrEP users also reported HIV‐ and sexuality‐related stigma, often leading to pill concealment, many selected this modality due to familiarity. Interpersonal and institutional supports shaped adherence to the chosen PrEP modality (Figure 1). Oral PrEP users emphasized that encouragement, personal reminders and accountability partnerships from friends and clinic staff helped mitigate stigma and facilitate ongoing pill‐taking. CAB‐LA users described intrinsic motivators such as altruism (i.e. contributing data to advance HIV prevention science) that facilitated study participation, which in turn cultivated social connectedness and belongingness with clinic staff and other LGBTQ+ individuals, reinforcing continued engagement with injection visits. Across modalities, interpersonal influences, such as coordinating clinic appointments and transportation, and creating safe spaces to discuss PrEP and sexual health, supported adherence. Institutional‐level supports, including reminders and flexible scheduling, improved accessibility and feasibility of injection visits and promoted adherence.

**Conclusions**: There are multilevel pathways through which social factors shape PrEP modality choice and adherence. Results highlight actionable, modality‐specific implementation strategies, including addressing disclosure concerns and other stigma‐related barriers among oral PrEP users and leveraging clinic‐based supports and intrinsic motivators to facilitate CAB‐LA scale‐up and sustained engagement among all PrEP users.

**TABLE 1:**
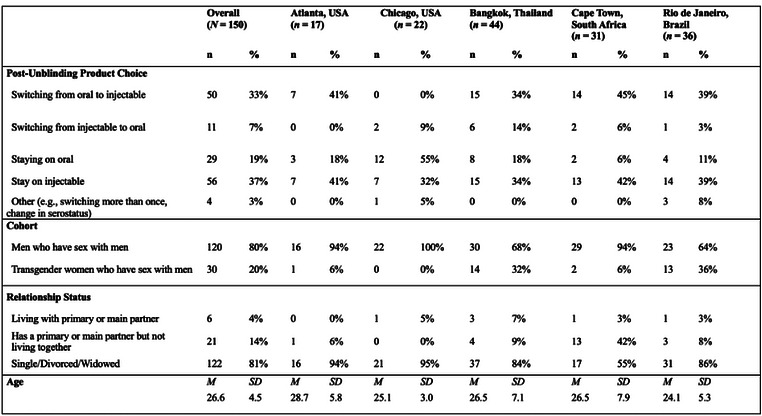
OAD0805 | Participant demographics characteristics and modality choice.

**FIGURE 1:**
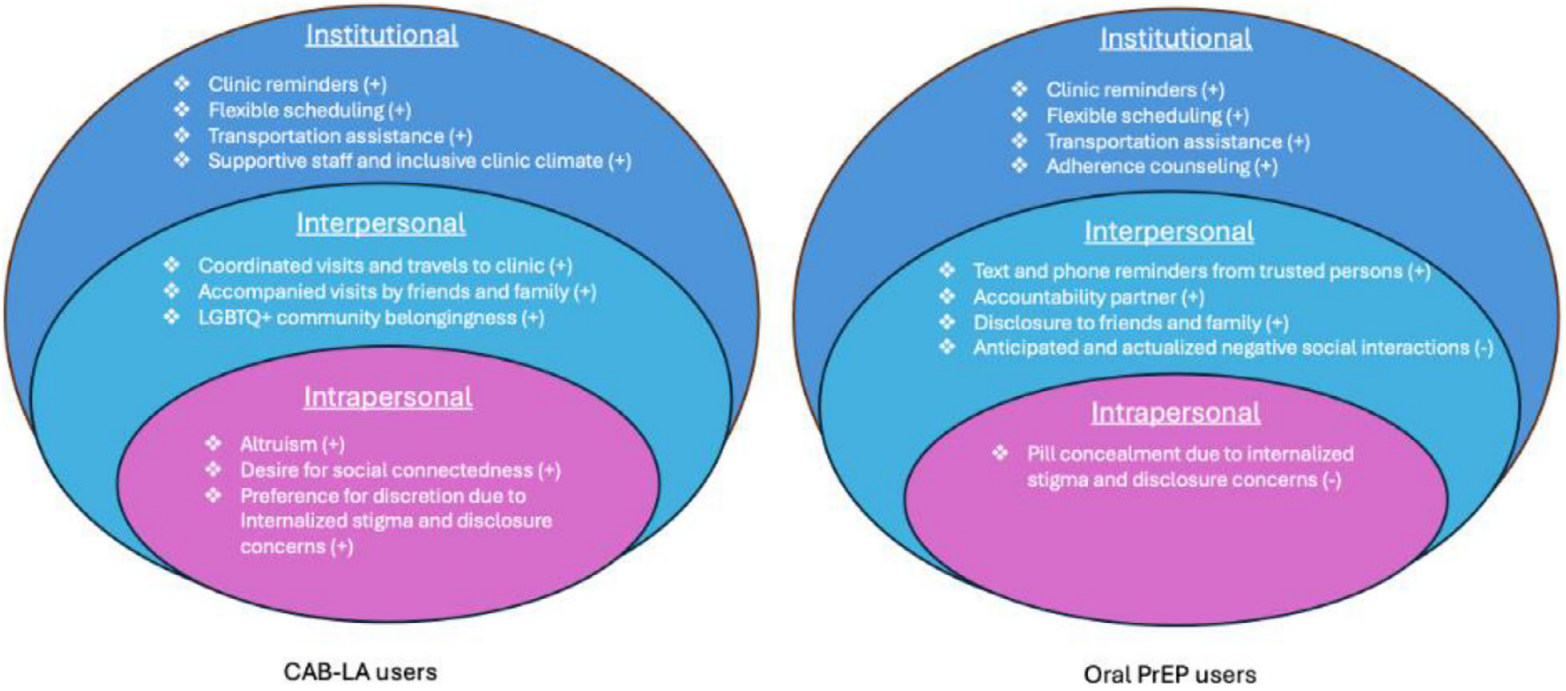
OAD0805 | Socially driven barriers (–) and facilitators (+) to PrEP adherence by modality choice within a socioecological framework.

## OAD1802 Addressing Inter‐Institutional Partnerships on Combination HIV Prevention for Chemsex in São Paulo, Brazil

B. Kauss ^1^, F. Almeida^2^

^1^Federal University of Rio Grande so Sul, Porto Alegre, Brazil; ^2^University of Brasília, Communication Department, Brasília, Brazil

**Background**: Chemsex, which involves using drugs to enhance sexual experiences, has emerged as a significant driver of increased risk for HIV and other sexually transmissible infections (STIs) (UNODC; UNAIDS, 2024). In 2022, the relative risk of acquisition of HIV was 14 times higher among people who inject drugs compared to general adult population (UNAIDS, 2024). In Brazil, studies report high prevalence of chemsex among men who have sex with men (MSM), ranging from 27% to 69.9% (Souza et al., 2023). In response, UNAIDS promotes community‐led responses, combined HIV prevention and harm‐reduction approaches. Within this framework, the non‐governmental organization Centro de Convivência É de Lei (CCEDL), in partnership with municipal and state governments, implemented a pilot harm‐reduction project targeting chemsex in party settings in São Paulo, Brazil.

**Description**: The initiative prioritized gay men, MSM, and cisgender and transgender women, applying an intersectional approach that considered gender, identity, age and race/skin colour. The 6‐month pilot included seven outreach actions conducted at chemsex parties in São Paulo, reaching an estimated 3000 participants. Prevention commodities distributed included 2000 condoms, 6000 lubricant units, 4500 informational materials, 200 HIV self‐test kits and 300 combined prevention and safer drug‐use kits. In addition, a capacity‐building workshop on combined HIV prevention in chemsex contexts was conducted for health professionals and public managers. A technical guide, Chemsex—Recommendations on Practices and Harm Reduction, produced by CCEDL consolidated evidence to support health management.

**Lessons Learned**: Findings underscore that meaningful engagement of people who use drugs, strong inter‐institutional partnerships and coordinated governance are critical to the effectiveness of harm‐reduction interventions in chemsex settings. Collaboration facilitated territorial access, supported ethical implementation and enabled tailoring actions to local contexts. The pilot demonstrated that sustainable care in vulnerable settings depends on collective, territorially grounded and human rights–based approaches.

**Conclusions/Next Steps**: This experience demonstrates that community‐led, harm reduction–based interventions in chemsex settings are both feasible and effective when implemented through strong partnerships between civil society and public institutions. Embedding these initiatives within a multisectoral response, as advocated by UNAIDS, is essential to address persistent gaps in HIV prevention for populations most vulnerable in chemsex contexts.

## OAD1803 Meeting Transgender‐Specific Needs: Service Acceptance and Identified Needs Among Thai Trans Women Engaging in Substance Use or Chemsex (iT‐REX Study)

A. Hiransuthikul ^1,2^, T. Payuha^2^, J. Boonruang^2^, K. Boontho^2^, T. Amatsombat^2^, A. Chancham^2^, P. Pumphosuwan^2^, R. Janamnuaysook^2,3^, N. Phanuphak^2,3^, iT‐REX Study Team

^1^Faculty of Medicine, Chulalongkorn University, Preventive and Social Medicine, Bangkok, Thailand; ^2^Institute of HIV Research and Innovation (IHRI), Bangkok, Thailand; ^3^Faculty of Medicine, Chulalongkorn University, Center of Excellence in Transgender Health (CETH), Bangkok, Thailand

**Background**: Substance use and chemsex among transgender women are increasingly recognized, yet harm reduction services remain scarce in Thailand. Building on a pre‐implementation study, which identified barriers and facilitators, we implemented a multi‐component model integrating substance literacy, sexual health, mental health and socio‐legal support. This analysis reports baseline findings on service acceptability and needs identified across components.

**Methods**: The iT‐REX study is a 12‐month prospective cohort enrolling self‐identified transgender and non‐binary persons aged ≥18 years who use substances or engage in chemsex and are not living with HIV. Participants undergo assessments across four domains—substance‐related, sexual health, mental health and socio‐legal/rights—with tailored interventions for those screening positive.

**Results**: A total of 63 participants, all transgender women, were enrolled (median age 31.1 years); 34 (54.0%) were sex workers, 42 (66.7%) had gender‐affirming surgery and 42 (66.7%) currently used gender‐affirming hormones. Substance use patterns included non‐chemsex (16; 25.4%), chemsex only (15; 23.8%) and both (32; 50.8%). Overall, 26 (41.3%) used multiple substances, with 6/26 (23.1%) using them simultaneously; 6 (9.5%) reported injection use.

All but two (96.8%) completed in‐person visit. All participants agreed to substance use, sexual health, mental health and socio‐legal/rights assessments. Substance‐related harms were identified in 24 (38.1%); among them, 12/24 (50.0%) requested substance literacy counselling, compared with 19/39 (48.7%) without identified harms. Sexual health needs included 6/55 (9.8%) CT/NG infections and 4/60 (6.7%) new syphilis diagnoses, of whom 6/8 (75.0%) accepted treatment. Among 28 anti‐HBs negative participants,15/28 (53.6%) accepted vaccination. The online participant underwent HIV self‐testing but could not complete other STI testing. Most agreed to PrEP (44; 69.8%), including four who had never used it. Among 42 current hormone users, 23 (54.8%) requested hormone‐level testing. Mental health issues were detected in 35 (55.6%), with only two declining further care. Stigma and discrimination screening was positive in 44 (69.8%), but only 1/44 (2.3%) requested support.

**Conclusions**: Transgender women using substances, including chemsex, showed high acceptance of gender‐affirming, multi‐component harm reduction assessments. Needs emerged across four domains. Care uptake was highest for mental health, STI treatment and PrEP, while uptake of HBV vaccination, substance literacy and rights literacy requires further improvement.

## OAD1804 Improving HIV Retention Among Men Who Have Sex With Men Who Engage in Chemsex in South African Townships: Evidence From the EJAF Chemsex Programme Community Model

D. Nel, M. Slabbert, H. Mudzanani

OUT LGBT Wellbeing, Pretoria, South Africa

**Background**: Chemsex, the intentional use of psychoactive substances in sexual contexts, is a growing public health concern among men who have sex with men (MSM). MSM who engage in chemsex (MSMC) experience disproportionately low retention in HIV care in South African townships due to intersecting stigma, substance use and systemic barriers. In 2024, OUT LGBT Well‐being piloted a community‐led chemsex programme, with Elton John AIDS Foundation support, to address structural and psychosocial determinants of ART retention among MSMC in Soweto, Orange Farm and Johannesburg Inner City.

**Description**: The 15‐month pilot (January 2024–March 2025) and no‐cost extension (April–September 2025) implemented a multi‐level intervention informed by the Social Ecological Model. Services addressed individual, social network and community needs, with structured intake, mental health screening, harm reduction, peer navigation and community advisory groups. Biomedical services were integrated through mobile outreach during the pilot and client transition to Department of Health facilities following the termination of USAID support in March 2025. Psychosocial services included tailored treatment plans, group cognitive behavioural therapy, gatekeeper engagement at eight chemsex houses and informal peer support.

**Lessons Learned**: 127 HIV‐positive MSMC enrolled, 69% newly diagnosed (50% positivity rate). ART retention increased from 42% at baseline to 83% by March 2025; viral suppression reached 88%. At programme close, retention declined to 67%, reflecting reliance on community‐based delivery. Among 81 clients with complete baseline and follow‐up data, statistically significant improvements were observed in anxiety, depression, psychosis, internalized homophobia and suicidal ideation (*p* < 0.01). Harmful drug use decreased from 79% to 57% (*p* < 0.001), with skills development training linked to reduced use. Most clients were socioeconomically marginalized (only 9% formally employed; 70% lived with family) and 94% accessed peer‐led services. Peer‐led engagement, gatekeeper partnerships, and Community Advisory Groups fostered social cohesion and reduced isolation.

**Conclusions/Next Steps**: This programme demonstrates that a tailored, peer‐led, context‐responsive intervention can significantly improve ART retention and psychosocial wellbeing among MSMC in resource‐limited settings. Future scale‐up should integrate services into public health systems, with sustained investment in community‐based delivery, gatekeeper partnerships and mental healthcare. Lessons informed national chemsex harm reduction guidelines.

## OAD1805 Breaking the Silence: Introducing Chemsex Harm Reduction in Criminalized Asian Settings

P. Loh^1^, P. Narayanan^2,3^, M. Basnayake ^4^, Y. Yusoff^2,3^, B. Goh^2,3^, J. Kuru‐Utumpala^4^, R. Siriwardana^4^, M. Kusen^1^, F. Young^5^

^1^Health Equity Matters, Bangkok, Thailand; ^2^Malaysian AIDS Council, Kuala Lumpur, Malaysia; ^3^Malaysian AIDS Foundation, Kuala Lumpur, Malaysia; ^4^Family Planning Association of Sri Lanka, Colombo, Sri Lanka; ^5^Health Equity Matters, Sydney, Australia

**Background**: Chemsex, or sexualized drug use, has emerged as a critical but often overlooked issue in Asia, intersecting with HIV, STIs, mental health, stigma and criminalization. In many settings, chemsex remains invisible within health systems due to legal risk, moral framing and limited programmatic guidance. Prior to 2024, neither Sri Lanka nor Malaysia had structured, health‐led approaches to support people engaging in chemsex.

**Description**: Under SKPA‐2, partners in Sri Lanka and Malaysia piloted structured chemsex harm reduction interventions focused on capacity building and systems engagement. Two Training of Trainers (ToTs) were delivered. In Sri Lanka, a 5‐day ToT (January 2025) trained 20 peer outreach workers and community leaders working with key populations. The programme combined technical content on chemsex and harm reduction with facilitation and adult education skills, using lectures, role‐plays, case discussions and participant‐led practice sessions. Pre‐ and post‐training assessments were administered to measure knowledge change. In Malaysia, a 5‐day ToT trained 10 core team members, focusing on harm reduction approaches and facilitation skills to support cascade implementation. Across both countries, participants developed session plans, concept notes and action plans to deliver follow‐on trainings.

**Lessons Learned**: Four key lessons emerged. First, early and sustained engagement with government counterparts was critical to legitimize chemsex work, reduce institutional resistance and create safer operating space for community‐led interventions. Second, peer‐led facilitation fostered trust and enabled open, non‐judgemental discussion of highly stigmatized issues. Third, effective chemsex responses must extend beyond HIV prevention to include mental health, sexual health and rights‐based referral pathways. Fourth, the Sri Lanka ToT demonstrated measurable gains in implementation readiness: post‐training assessments showed substantial improvements in knowledge of harm reduction practices (+54%), risks associated with chemsex (+40%), adult education principles (+47%) and safer sex strategies including PrEP (+29%). Despite these gains, chemsex harm reduction requires sustained, long‐term investment, particularly in criminalized and highly sensitive settings where short‐term funding limits continuity and safe system integration.

**Conclusions/Next Steps**: These pilot efforts demonstrate the feasibility of introducing chemsex harm reduction in criminalized contexts through meaningful government–community collaboration. Scaling and sustaining impact will require long‐term financing, integration of mental health services and regional guidance to support replication across Asia‐Pacific.

## OAD2102 Psychosocial Mechanisms Linking Intersecting Stigmas and Condomless Anal Sex Among Trans Women in India: Mediation or Interactions or Both?

V. Chakrapani ^1,2^, J. Kaur^3^, A. C. Tsai^4,5,6^, A. I. Scheim^7,8,9^, M. Shunmugam^1^, M. Sivasubramanian^2^

^1^Centre for Sexuality and Health Research and Policy (C‐SHaRP), Chennai, India; ^2^The Humsafar Trust, Mumbai, India; ^3^Postgraduate Institute of Medical Education and Research (PGIMER), Chandigarh, India; ^4^Center for Global Health and Mongan Institute, Massachusetts General Hospital, Boston, United States; ^5^Harvard Medical School, Boston, United States; ^6^Department of Epidemiology, Harvard T.H. Chan School of Public Health, Boston, United States; ^7^The Williams Institute, School of Law, University of California, Los Angeles, United States; ^8^Department of Epidemiology and Biostatistics, Schulich School of Medicine and Dentistry, Western University, London, Ontario, Canada; ^9^Unity Health Toronto, Toronto, Canada

**Background**: Transgender women in India experience disproportionate HIV burden. The psychosocial pathways linking intersecting stigmas—transgender identity stigma (TGS), HIV‐related stigma (HPS) and sex work stigma (SWS)—to condomless receptive anal sex (CAS) remain unexplored. Using minority stress theory, psychological mediation and intersectionality frameworks, we examined how these stigmas directly and indirectly influence CAS and whether their combined effects are synergistic rather than additive.

**Methods**: Between November 2020 and March 2022, we conducted a prospective three‐wave cohort study of 500 transgender women (mean age = 27.6; 60.2% in sex work) in Mumbai and Chennai. We assessed three stigma types at Wave‐1 (TGS, HPS, SWS); five psychosocial variables at Wave‐2 (depression [PHQ‐9], anxiety [GAD‐2], problematic alcohol use [AUDIT‐C], internalized transprejudice, social support); and CAS at Waves 2 and 3. We used causal mediation analysis (estimating Natural Indirect Effects [NIE]) and additive interaction analysis (estimating Attributable Proportion [AP] and Relative Excess Risk due to Interaction [RERI]) to test synergistic effects.

**Results**: Baseline mean stigma levels were high (TGS = 15.27, HPS = 6.06, SWS = 14.48). At Wave‐2, anxiety (38%), internalized transprejudice (46%) and low social support (46%) were prevalent; 61% reported CAS, rising to 81% by Wave‐3. Social support mediated all three stigmas’ effects on CAS (TGS: NIE OR = 1.02, *p* = 0.004; HPS: OR = 1.33, *p* = 0.004; SWS: OR = 1.03, *p* = 0.001). Anxiety mediated all three (ORs 1.04–1.07, *p*≤0.03); depression mediated only TGS and HPS. For two‐way interactions on Wave‐2 CAS, HPSxTGS showed strong synergy (AP = 0.31, 95% CI 0.11–0.52), while HPSxSWS showed weak synergy (AP = 0.14, 95% CI 0.03–0.26). For psychosocial mediators, depression and anxiety showed strong synergistic effects across multiple stigma combinations (depression: HPSxTGS AP = 0.50; SWSxTGS AP = 0.56; anxiety: HPSxSWS AP = 0.50, SWSxTGS AP = 0.45). Alcohol use showed strong synergy for HPSxSWS (AP = 0.65), while social support showed antagonistic effects for HPSxTGS (RERI = −0.09).

**Conclusions**: We found support for both mediation and interaction effects. Social support and anxiety were found to be the most robust mediators of stigma's effects on CAS. The synergistic interactions between stigma pairs demonstrate that trans women experiencing multiple concurrent stigmas face compounded, not additive, HIV risk. Interventions must simultaneously address multiple stigmas and their psychosocial consequences‐integrating mental health screening and treatment with HIV interventions (see Figure 1).

**FIGURE 1:**
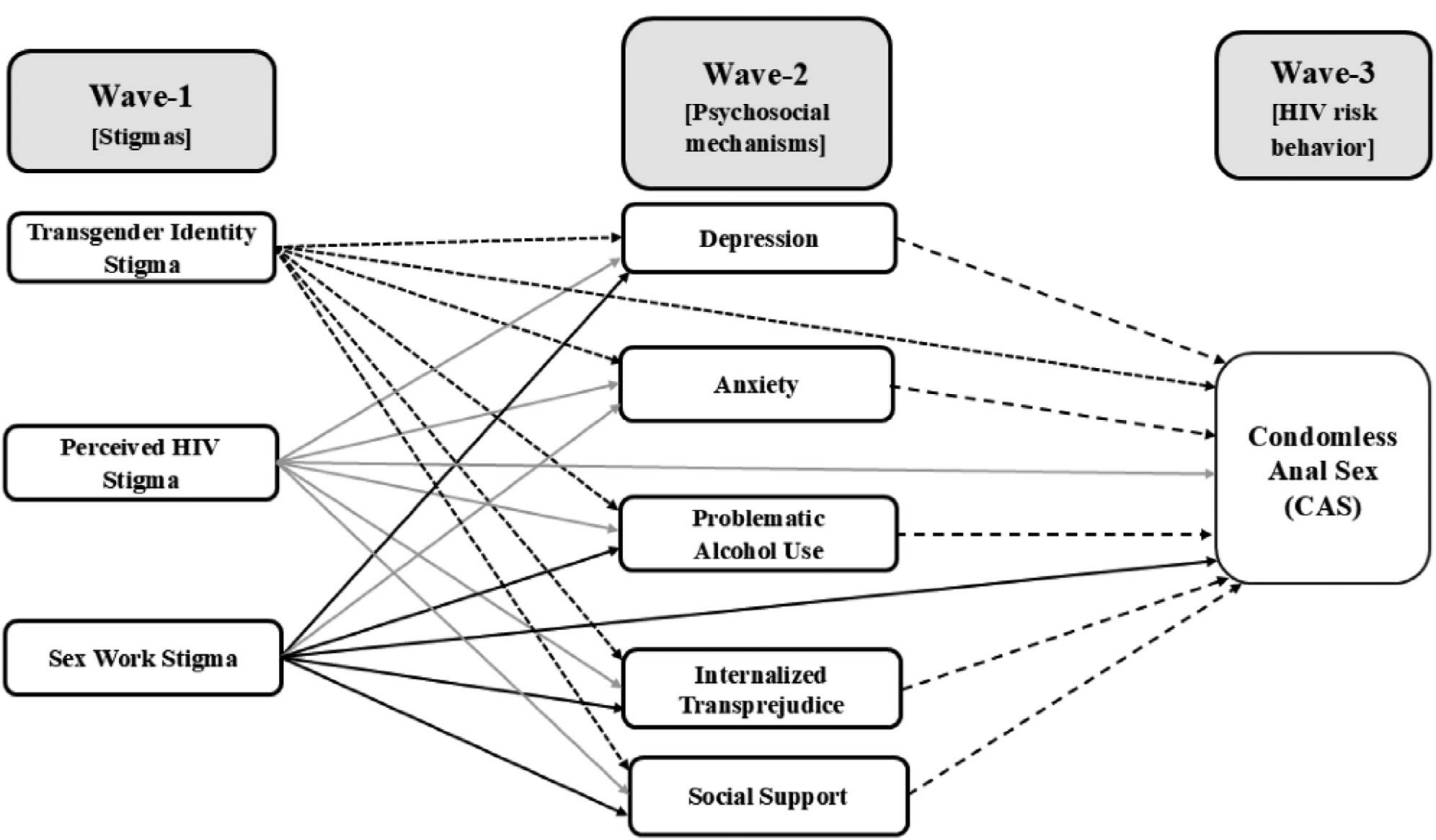
OAD2102 | Conceptual model to test the mediation hypothesis.

## OAD2103 Institutionalized Intersectional Stigma Among Medical Students in the Southern United States: The Impact of HIV Knowledge, Heterosexism and Racism on HIV Stigma

K. D. Samuel ^1,2,3^, R. E. Davis^1,3^, M. E. Buttram^1,2^, P. D. Dobbs^1,2^, S. Hirshfield^4^

^1^University of Arkansas, Health, Human Performance and Recreation, Fayetteville, United States; ^2^University of Arkansas, Center for Public Health and Technology, Fayetteville, United States; ^3^University of Arkansas, Substance Use and Mental Health Lab, Fayetteville, United States; ^4^SUNY Downstate Health Sciences University, School of Public Health, New York City, United States

**Background**: Intersectional stigma—HIV stigma, heterosexism and racism—remains a major barrier to HIV prevention, treatment and quality of life for people living with HIV (PLHIV), particularly Black men who have sex with men (MSM) in the Southern United States. Few studies examine medical schools as institutional‐level structures that can either perpetuate or reduce stigma. This study addresses this gap using the Health Stigma and Discrimination Framework to explore intersectional stigma among medical students (future healthcare providers). A key hypothesis was that HIV knowledge would reduce the effects of racism and heterosexism on HIV stigma.

**Methods**: A cross‐sectional online survey was conducted with Southern US medical students (January–March 2024). Validated measures of heterosexism, symbolic racism and HIV stigma were used; PrEP awareness was measured with a single item. HIV knowledge was assessed using a new 21‐item expert‐reviewed scale. Analyses included bivariate relationships, multiple regression modelling and moderation analyses.

**Results**: A total of 128 students participated (mean age = 25, ±2.5); mostly women (62%) and non‐Hispanic White (66%), with only 4% Black students. Students attended medical school in Arkansas (51%), Florida (41%) and South Carolina (8%). Most (65%) reported frequent clinical exposure and exposure to PLHIV in clinical settings (69%), but only 20% encountered PLHIV in everyday life. PrEP awareness was high (91%). HIV knowledge was moderate (mean score = 13.44/21, ±4), with 64% of items answered correctly. Advances like Undetectable = Untransmittable (U = U) were less known, while PrEP knowledge was higher. Heterosexism had the strongest correlation with HIV stigma (*r* = 0.72, *p* < 0.001), followed by racism (*r* = 0.55, *p* < 0.001); HIV knowledge had a moderate negative correlation with HIV stigma (*r* = –0.27, *p* < 0.05). Multiple regression revealed that heterosexism and racism strongly predicted HIV stigma, while HIV knowledge was the weakest predictor and lost significance when controlling for other variables. The moderation hypothesis was not supported.

**Conclusions**: Heterosexism and racism strongly drive HIV stigma among medical students. HIV knowledge alone is insufficient. Medical schools must implement multicomponent curricular and extra‐curricular interventions targeting systemic heterosexism and racism to reduce HIV stigma and improve future medical practice.

## OAD2104 Stigma and Resilience Trajectories in the *Manas Por Manas* Peer‐Navigation HIV Prevention Trial Among Trans Women and Travestis in São Paulo, Brazil

S. R. Cluesman ^1,2^, T. B. Neilands^3^, J. L. Gomez^4^, A. R. Mocello^3^, G. Saggese^4^, J. Sevelius^3,5^, M. A. S. M. Veras^4^, S. A. Lippman^6^

^1^Columbia University Irving Medical Center and the New York State Psychiatric Institute, HIV Center for Clinical and Behavioral Studies, Division of Gender, Sexuality, and Health, New York, United States; ^2^New York University, Center for Drug Use and HIV Research, School of Global Public Health, New York, United States; ^3^University of California San Francisco, Department of Medicine, San Francisco, United States; ^4^Faculdade de Ciências Médicas da Santa Casa de São Paulo, Department of Public Health, São Paulo, Brazil; ^5^Columbia University Irving Medical Center, Department of Psychiatry, New York, United States; ^6^University of California San Francisco, Center for AIDS Prevention Studies, San Francisco, United States

**Background**: In Brazil, transgender women and *travestis* (TWT) have a high burden of HIV alongside persistently low PrEP uptake. These inequities are exacerbated by intersectional stigma related to HIV, gender identity and sex work, which constrain access to HIV prevention. While peer navigation interventions have demonstrated feasibility and acceptability among TWT in Brazil, little is known about how stigma and resilience factors change over time or whether effects vary by baseline psychosocial vulnerability. This study examined longitudinal patterns of stigma and resilience among TWT participating in a gender‐affirming peer‐navigation randomized controlled trial, *Manas por Manas*, in São Paulo, Brazil.

**Methods**: Data were drawn from Year 1 of *Manas por Manas* (*N* = 392). Participants were randomized to an immediate peer‐navigation and group‐based intervention or waitlist control, and completed interviewer‐administered surveys at baseline, 6 months and 12 months. Measures captured indicators of intersectional stigma across HIV, sex work, PrEP and anticipated stigma domains, alongside resilience indicators including transgender identity, transgender pride and psychological gender affirmation. Longitudinal change was examined using latent growth curve modelling (LGCM).

**Results**: At baseline, participants were 33 years old on average (SD = 10), 45% identified as mixed‐race, 38% reported current sex work and 27% reported unstable housing. LGCM's showed significant heterogeneity in stigma and resilience trajectories over 12 months. In unconditional models, PrEP and sex work stigma declined significantly across the full sample (Figure 1). No significant mean change was observed for the remaining outcomes. In conditional two‐group models, lower baseline transgender pride was significantly associated with greater increases over time in the intervention arm (*β* = −0.28, SE = 0.07, *p* < 0.001; Figure 2), with no such association in the control arm. All models demonstrated satisfactory global fit (CFI ≥0.99; RMSEA ≤ 0.06; SRMR ≤ 0.03).

**Conclusions**: While reductions in PrEP and sex work stigma were observed across the full sample, changes in transgender pride were specific to the intervention arm and concentrated among participants with lower baseline levels. Findings suggest that peer‐navigation may be particularly effective in strengthening positive identity among those with greater initial vulnerability, while broader stigma reductions may reflect contextual or non‐specific influences. The results support the value of peer‐based interventions.

**FIGURE 1:**
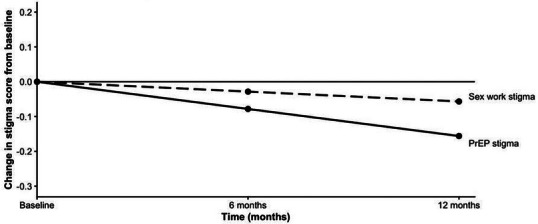
OAD2104 | Longitudinal change in stigma outcomes.

**FIGURE 2:**
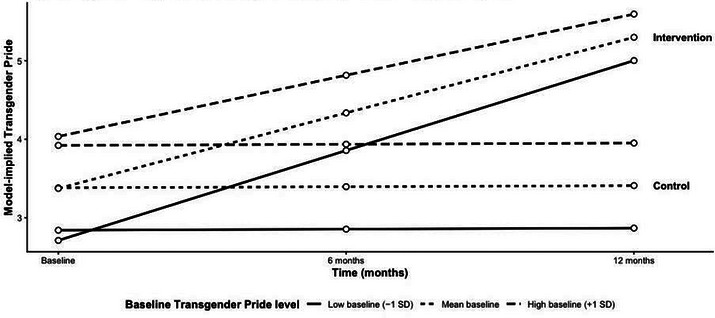
OAD2104 | Longitudinal trajectories of transgender pride by baseline level and study arm.

## OAD2105 Evaluating an Intervention to Reduce Stigma Towards Key Populations Among Healthcare Workers in Mombasa, Kenya

A. Adam ^1^, P. Owira^2^, R. Hashim^1^, K. David^3^, C. Kithinji^1^, G. Mwendar^3^, V. Winchester^3^, H. Masud^3^, C. Mugun^3^, R. Antonov^3^, B. Imani^4^, M. Kimani^5^, M. Pastrana^3^, A. Guadarrama^3^, I. Ciglenecki^3^, D. Callander^3,6^

^1^Mombasa County, Department of Health, Mombasa, Kenya; ^2^International Centre for Reproductive Health, Health, Mombasa, Kenya; ^3^Médecins Sans Frontières, Health, Mombasa, Kenya; ^4^Hope Transgender Initiative, Health, Mombasa, Kenya; ^5^Kenya Medical Research Institute, Health, Mombasa, Kenya; ^6^Kirby Institute, University of New South Wales, Sexual Health, Sydney, Australia

**Background**: Stigma towards HIV “key populations” remains a barrier to healthcare. From 2024 to 2025, Médecins Sans Frontières implemented a multifaceted anti‐stigma intervention for healthcare workers at three primary care clinics in Mombasa. Activities combined education, intergroup contact and reflective discussions related to key populations generally (*n* = 3) and specific sub‐populations (*n* = 3 sexual and gender minorities, *n* = 3 people who use drugs). The intervention also included activities targeting other stigmatized topics (*n* = 10), including mental health, abortion and sexual violence. This study evaluates the intervention's effects.

**Methods**: A prospective cohort of healthcare workers was conducted (June 2023−December 2025) at three clinics receiving the anti‐stigma intervention (“intervention sites”) and three non‐intervention matched clinics (“control sites”). Stigma levels were quantified using validated scales standardized from 1 (low stigma) to 5 (high stigma). Multivariable ordinal regression was used to assess changes in stigma before (Waves 1−3) and after (Waves 4−6) the introduction of the intervention. Adjusted odds ratios (aOR) and confidence intervals (CI) quantify effects before/after stratification by intervention exposure, accounting for gender, education and work experience.

**Results**: Over six waves, 532 healthcare workers contributed 1298 data points (*n* = 688 intervention, *n* = 610 control). At intervention sites, sexual and gender minority stigma decreased from 2.37 to 2.19 (aOR = 0.64, 95% CI:0.48−0.86) but was unchanged at control sites (2.62 vs. 2.61, aOR = 0.99, 95% CI:0.75−1.29; Figure 1). Sex worker stigma was stable at intervention sites (1.90 vs. 1.90; aOR = 1.11, 95% CI:0.91−1.36) while increasing at control sites (2.08 vs. 2.20, aOR = 1.35, 95% CI:1.02−1.79). Stigma towards people who use drugs was stable at both intervention sites (2.55 vs. 2.44; aOR = 0.79, 95% CI:0.59−1.06) and control sites (2.73 vs. 2.74; aOR = 1.03, 95% CI:0.79−1.35). Stigma towards other health‐related topics decreased at intervention sites (2.19 vs. 2.09; aOR = 0.70, 95% CI:0.50−0.99) while remaining stable at control sites (2.29 vs. 2.20; aOR = 0.81, 95% CI: 0.59−1.12).

**Conclusions**: The intervention was associated with decreased stigma towards sexual and gender minorities but no other key populations. It also reduced stigma towards other sensitive topics. Results suggest need to balance “general” and population‐specific efforts to reduce stigma. This study provides evidence that education, reflection and contact can effectively challenge stigma among healthcare workers.

**FIGURE 1:**
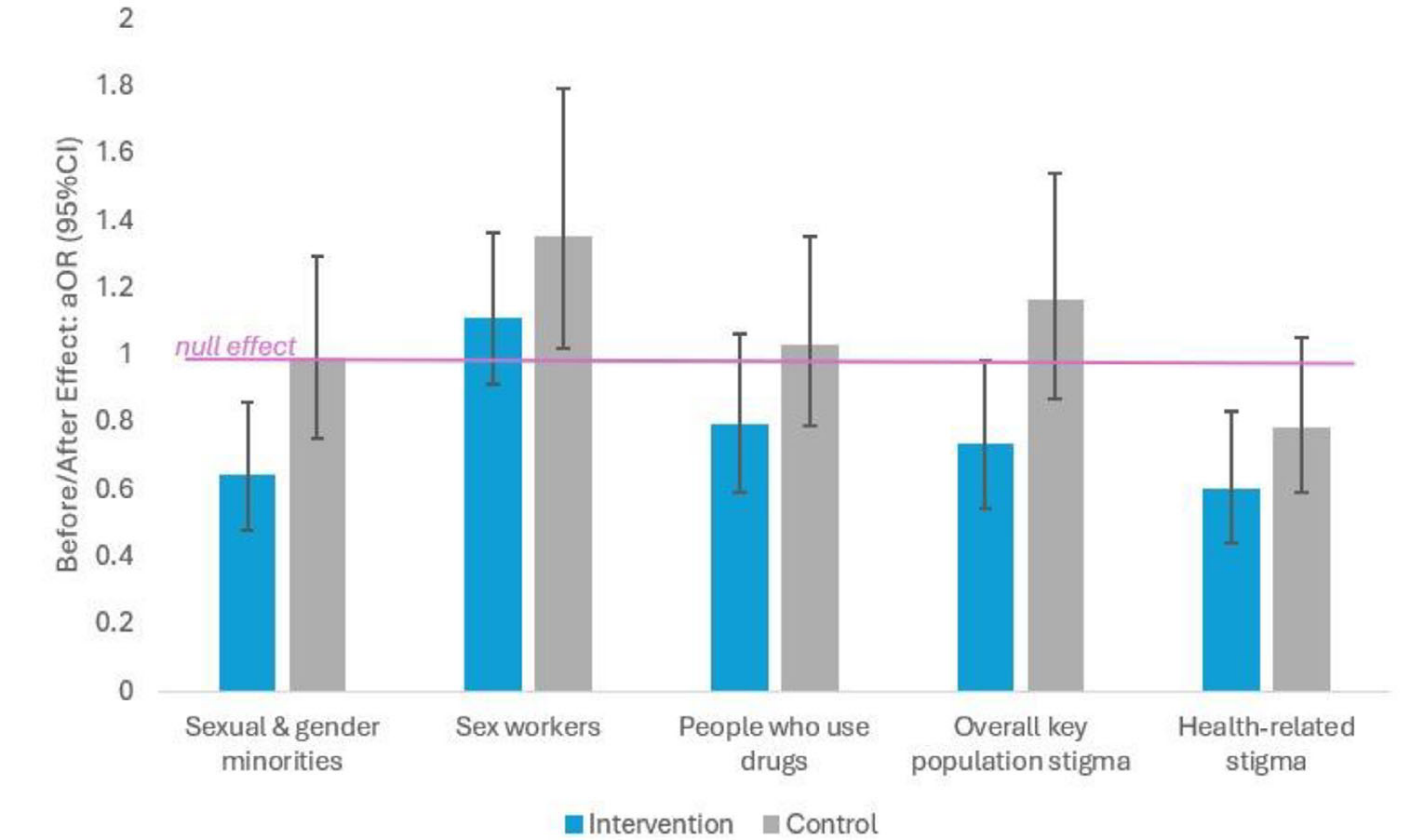
OAD2105 | Effects before/after an anti‐stigma intervention for healthcare workers at intervention and control sites (*n* = 1298).

## OAD2402 Typologies of Stigma and Implications for HIV Prevention Among Women Who Exchange Sex and Use Drugs in Kazakhstan

T. McCrimmon ^1^, M. Darisheva^2^, B. West^3^, M. Chang^3^, A. Terlikbayeva^2^, N. Zholnerova^4^, E. Grigorchuk^5^, S. Primbetova^2^, L. Gilbert^3^, N. El‐Bassel^3^, V. Frye^3^

^1^Yale University School of Medicine, Department of Psychiatry, New Haven, United States; ^2^Global Health Research Center of Central Asia, Almaty, Kazakhstan; ^3^Columbia University School of Social Work, New York, United States; ^4^NGO Amelia, Taldykorgan, Kazakhstan; ^5^Kazakhstan Union of People Living with HIV, Almaty, Kazakhstan

**Background**: HIV‐related stigma is a well‐documented structural barrier to healthcare access, including pre‐exposure prophylaxis (PrEP) for HIV prevention. Women who exchange sex and use substances (WESUS) in Kazakhstan embody multiple stigmatized identities, but few empirical studies examine how sex work and substance use stigma intersect and influence healthcare access. This study examines the patterns of stigma experienced by WESUS, and how they are differentially associated with engagement in HIV prevention.

**Methods**: From November 2022 to August 2023, we recruited 90 HIV‐negative, PrEP‐eligible women with recent substance use and sex exchange through community partners and sex work venues in Almaty, Kazakhstan. Participants completed computerized surveys in a field office location, self‐reporting three types of stigma (anticipated, enacted, internalized) each for both sex work and drug use. We used these six measures as indicators in a latent profile analysis identifying distinct stigma profiles among participants. We then used multivariable regression models to examine how these profiles were associated with measures of medical mistrust, access to care and attitudes towards PrEP, controlling for sociodemographics.

**Results**: We identified three distinct stigma profiles: “low” levels of all six sex work and substance use stigma measures (*n* = 24, 26.7%), “high” levels of all measures (21, 23.3%) and high levels of anticipated stigma measures for sex work and substance use, but moderate levels of all enacted and internalized stigma measures, called “high anticipated” (45, 50%). Compared to low‐stigma, high‐stigma participants reported significantly more positive PrEP attitudes (*b* = 4.9, 95% CI [2.3, 7.4]) and less access to care (–3.09 [–6.0, –0.1]). Compared to low‐stigma, both high‐ and high anticipated‐stigma participants reported significantly higher medical mistrust (4.4 [1.9, 6.8] and 2.8 [0.9, 4.6], respectively).

**Conclusions**: Latent profiles in this sample clustered by type of stigma (anticipated, enacted, internalized) rather than by focus (sex work vs. substance use) suggesting that WESUS experience sex work and substance use stigmas not as distinct phenomena, but as intersecting experiences of marginalization. The high‐stigma profile's lower access to care, yet more positive attitudes towards PrEP, suggests that they may hesitate to access PrEP via traditional institutions; trusted NGOs and community‐based partners are best positioned to reach these women, alongside online prescribing and long‐acting PrEP options.

## OAD2403 Implementing Intercultural Mobile Health Brigades to Expand HIV Screening Among Awajún and Wampis Indigenous Communities in the Peruvian Amazon

Á. García, C. Benites, A. Vera, N. Romero, R. Terán, J. Olivera

Ministry of Health of Peru, Directorate for the Prevention and Control of HIV, STIs and Hepatitis, Lima, Peru

**Background**: In 2019, an epidemiological study conducted at the request of the Ministry of Health identified a high HIV prevalence (1.8%) among Awajún indigenous communities in Condorcanqui and Datem del Marañón, four times the national prevalence. Indigenous communities in the Peruvian Amazon face major geographic, cultural and socioeconomic barriers to accessing health services. In response, and with support from the Global Fund, an intercultural intervention using mobile health brigades was implemented between 2024 and 2025 to expand HIV prevention, screening and linkage to care among Awajún and Wampis populations in the Amazonas region.

**Description**: Four Amazonian mobile health brigades were deployed in 324 indigenous communities in the provinces of Bagua and Condorcanqui. Brigades were composed of physicians, midwives, nurses and indigenous health technicians, and worked in coordination with community authorities to ensure cultural appropriateness and community engagement. Services included culturally adapted health education, counselling, rapid screening for HIV, syphilis and hepatitis, and follow‐up of people living with HIV. In total, 28,956 individuals were screened (79% Awajún, 18% Wampis and 4% mestizo), representing approximately 30% of the estimated population in the intervention area; 53% were aged 12–29 years.

**Lessons Learned**: Mobile brigades with an intercultural approach enabled large‐scale HIV screening in geographically isolated indigenous territories. Delivering information and counselling in indigenous languages was critical to community acceptance and uptake of HIV testing. The programme successfully reached 69% of Awajún and Wampis communities in Bagua and Condorcanqui. Among those screened, 334 individuals had reactive HIV results; 177 were newly diagnosed and referred to 18 antiretroviral treatment centres to initiate care. Key best practices included engagement of indigenous health workers, coordination with local leadership and integration of HIV screening with other priority health services.

**Conclusions/Next Steps**: Intercultural mobile health brigades are a feasible and effective strategy to expand HIV screening and linkage to care among indigenous populations in the Peruvian Amazon. This model addresses geographic and cultural barriers while prioritizing adolescents and young adults. Scaling up and sustaining mobile, community‐based, intercultural interventions could strengthen HIV prevention, diagnosis and care for indigenous communities in remote settings.

## OAD2404 Bridging Last‐Mile HIV Care Gaps Using Drone‐Enabled Antiretroviral, Medical Consumables and Nutritional Supplement Delivery for Children and Adolescents in Unreached Communities of Bayelsa State, Nigeria: A Pilot Study

P. Ashinze ^1^, M. Olley^1^, O. Adeyemi^1^, C. Aruku^1^, F. Lannap^1^, K. Usman^1^, A. Ajonye^1^, S. Amadiegwu^1^, A. Olutola^1^, J. Pius^2^

^1^Center for Clinical Care and Clinical Research, Abuja, Nigeria; ^2^US Department of State, Abuja, Nigeria

**Background**: Sustained access to antiretroviral therapy (ART) is essential for viral suppression and long‐term survival among children and adolescents living with HIV (CALHIV). In Nigeria's Niger Delta, hard‐to‐reach riverine communities face intense logistical barriers to routine ART refills due to poor road infrastructure, seasonal waterways, high transport costs and safety risks. These constraints increase the risk of treatment interruption, poor adherence and virologic failure. Innovative, context‐adapted delivery strategies are urgently needed to bridge this last‐mile access gap.

**Objective**: To assess the effectiveness of drone‐enabled delivery of ART refills, test kits and medical consumables to CALHIV residing in hard‐to‐reach riverine communities of Bayelsa State, Nigeria.

**Methods**: A prospective, single‐arm pilot study was conducted across selected hard‐to‐reach communities in Sagbama, Yenagoa and Ekeremor Local Government Areas. Thirty CALHIV on stable ART and due for routine refills were purposively enrolled. A licensed commercial drone logistics provider (Zipline Nigeria) delivered pre‐packaged ART refills and nutritional supplements from a central dispatch point to pre‐identified secure community drop‐zones using predefined flight paths. Initial community apprehension was mitigated through targeted sensitization. Primary outcomes included delivery success rate, timeliness and medication integrity. Secondary outcomes assessed beneficiary and caregiver acceptability through brief structured interviews. Operational challenges were documented through field logs and debriefs.

**Results**: All scheduled deliveries were completed successfully (100% delivery and pickup rate), with no medication damage, temperature compromise or security breaches. Mean drone flight time was 40–60 min, compared with an estimated 3–4 h of conventional boat travel. Beneficiaries and caregivers reported high satisfaction, citing substantial reductions in travel time, transportation costs, physical stress and disruption to schooling or livelihood activities. Key operational challenges included weather dependency, communication delays with local focal persons and regulatory coordination requirements. No adverse events were recorded.

**Conclusions**: Drone‐enabled delivery of ART refills and nutritional supplements to CALHIV in remote communities of Bayelsa State is feasible and operationally effective. This pilot demonstrates the potential of unmanned aerial systems to strengthen last‐mile HIV service delivery and support adherence among geographically isolated paediatric populations. Larger‐scale implementation studies incorporating cost‐effectiveness analyses and integration into routine HIV supply chains are necessary.

## OAD2405 “Invisible But Not Silent”: Mental Health and Psychosocial Support Needs Among Marginalized and Criminalized Communities in Uganda

P. Muwanguzi ^1^, R. Nabunya^2^, S. J. Nalubega^2^

^1^The University of Manchester, Humanitarian and Conflict Response Institute, Manchester, United Kingdom; ^2^African Center for Health Equity Research and Innovation (ACHERI), Kampala, Uganda

**Background**: In Uganda, criminalization of sexual and gender minorities acts as a structural determinant of mental health, intensifying stigma and discrimination and undermining HIV programming. This study examined the psychosocial and mental health needs of sexual and gender minority (SGM) individuals and the barriers they face in accessing care.

**Methods**: This exploratory qualitative study was conducted between July and December 2024 in urban and peri‐urban Uganda, within SGM‐led community‐based organizations and sexual and reproductive health clinics providing HIV prevention and care services. The study focused on gay, bisexual and other men who have sex with men (GBMSM) and transgender individuals living in a criminalized context. Using purposive sampling, we recruited 38 SGM participants aged ≥18 years. Data were collected through three focus group discussions (*n*≈27) and 11 in‐depth interviews, conducted in safe community spaces, clinics or via secure virtual platforms. Semi‐structured guides explored mental health experiences, psychosocial stressors, service access and priorities for support. Transcripts were analysed using deductive reflexive thematic analysis, with iterative coding by two researchers.

**Results**: The findings identified five interconnected mental health and psychosocial support (MHPSS) needs among SGM populations. First, there is a critical need for accessible and affirming MHPSS, including early identification, prevention, non‐facility‐based care, online support and self‐management strategies. Second, participants highlighted the need for comprehensive substance use reduction, recovery and reintegration programmes, including individualized care, community‐based rehabilitation, peer support and reintegration pathways. Third, respondents emphasized integrated MHPSS within SGM‐focused health services, particularly HIV prevention and care, supported by peer‐led models. Fourth, there was a strong need for inclusive, safe and trusted mental health services, including SGM‐affirming providers, crisis support and safe spaces. Finally, participants underscored the urgent need to address structural and legal barriers, including stigma reduction, legal reform, and engagement with law enforcement and other stakeholders (Table 1).

**Conclusions**: The findings show that unmet mental health and psychosocial needs directly undermine HIV prevention, treatment engagement and retention in care among sexual and gender minority populations in criminalized settings. Integrating rights‐affirming MHPSS into HIV programmes is essential, and future research should evaluate scalable, community‐led models and their impact on HIV outcomes.

**TABLE 1:**
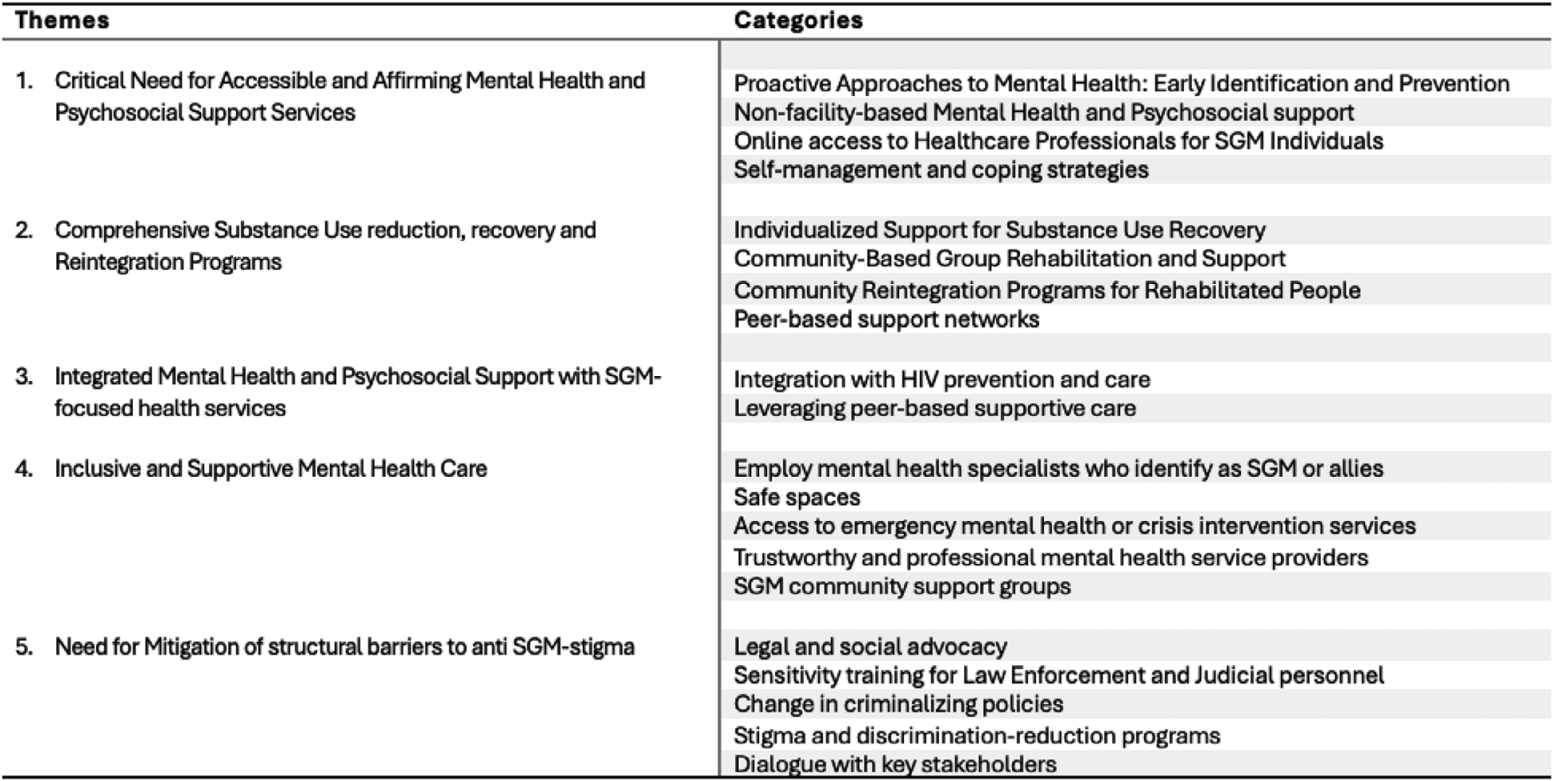
OAD2405

## OAD3302 Mobile Health for HIV Prevention Agency Among Nigerian Adolescent Girls and Young Women: Behavioural Pathways From System Quality to Health Capability

A. Phillips ^1^, C. Imarhiagbe^1^, T. Odebunmi^2^, M. Ukor^1^, D. Udanwojo^1^, A. Adekogbe^1^, O. Azurunwa^1^, F. Muhammed^2^, H. Akinniranye^2^, I. Suleiman^1^, O. Oladokun^2^, E. Ogbonna^3^, G. Aondoakula^1^, D. Kalu^1^, A. Sule^2^, F. Tamuno^2^, H. Bako^2^, M. Ashie^2^, R. Omodele^2^, A. Akinlalu^2^, J. Alozie^2^, B. Afolabi^2^, R.‐D. Umechinedu^1^, S. Hayab^2^, I. Ayo^2^, F. Ogirima^2^, B. Olatosi^4^, S. Weissman^4^, X. Li^4^, B. Oyeledun^5^

^1^Centre for Integrated Health Programs, Strategic Information, Abuja, Nigeria; ^2^Centre for Integrated Health Programs, Clinical Service Unit, Abuja, Nigeria; ^3^Centre for Integrated Health Programs, Special Project, Abuja, Nigeria; ^4^University of South Carolina, Big Data Health Science Center, Columbia, United States; ^5^Centre for Integrated Health Programs, Leadership & Management, Abuja, Nigeria

**Background**: Adolescent girls and young women (AGYW) in sub‐Saharan Africa face a disproportionate HIV burden. Digital health interventions may widen inequities if benefits depend on baseline education or age. To achieve digital equity, interventions should foster health agency (the internal capability to pursue valued health goals). Grounded in Ruger's Health Capability Paradigm, we examined whether the CoolGirls mobile app (an mHealth educational HIV prevention intervention) supports equitable health agency through hypothesized pathways linking system quality, psychological safety (social comfort), and technology acceptance to HIV knowledge and agency independent of age or education.

**Methods**: In December 2025, we conducted a cross‐sectional survey among 256 AGYW (15–24 years) using CoolGirls primarily in Lagos, Kaduna and Kogi states, Nigeria. HIV health agency was a composite capturing resilience to HIV‐related stigma, cognitive access to testing/healthcare and prevention readiness. Key predictors were System Usability Scale (SUS) and Technology Acceptance Model (TAM) scores (Cronbach's *α* = 0.98 each). Using structural equation modelling, we tested a hypothesized pathway from system quality to perceived social comfort and technology acceptance, and subsequently to HIV knowledge and agency, adjusting for age and education using robust standard errors.

**Results**: Mean age was 20 years (SD = 3.0); 64% had tertiary and 36% secondary education. The SEM demonstrated excellent fit (SRMR = 0.039; overall *R* ^2^ = 0.792). System quality predicted social comfort (*β* = 0.87, *p* < 0.001, *R* ^2^ = 0.764) and technology acceptance (*β* = 0.53, *p* < 0.001, *R* ^2^ = 0.410). Technology acceptance predicted HIV knowledge (*β* = 0.25, *p* < 0.001, *R* ^2^ = 0.082). Paths involving age and education were non‐significant. HIV knowledge (*β* = 0.22, *p* < 0.001) and perceived HIV risk (*β* = 0.13, *p* = 0.028) predicted health agency. Indirect effects of system quality (*β* = 0.022, *p* = 0.018) and technology acceptance (*β* = 0.034, *p* = 0.012) on agency were significant.

**Conclusions**: Findings are consistent with a capability‐expanding pathway in which high‐quality design supports a digitally safe environment and strengthens technology acceptance, enabling HIV knowledge and health agency. Within this sample, associations did not differ by age or education, suggesting potential for equitable impact. Future studies should test these pathways longitudinally and in more socioeconomically diverse settings (see Figure 1).

**FIGURE 1:**
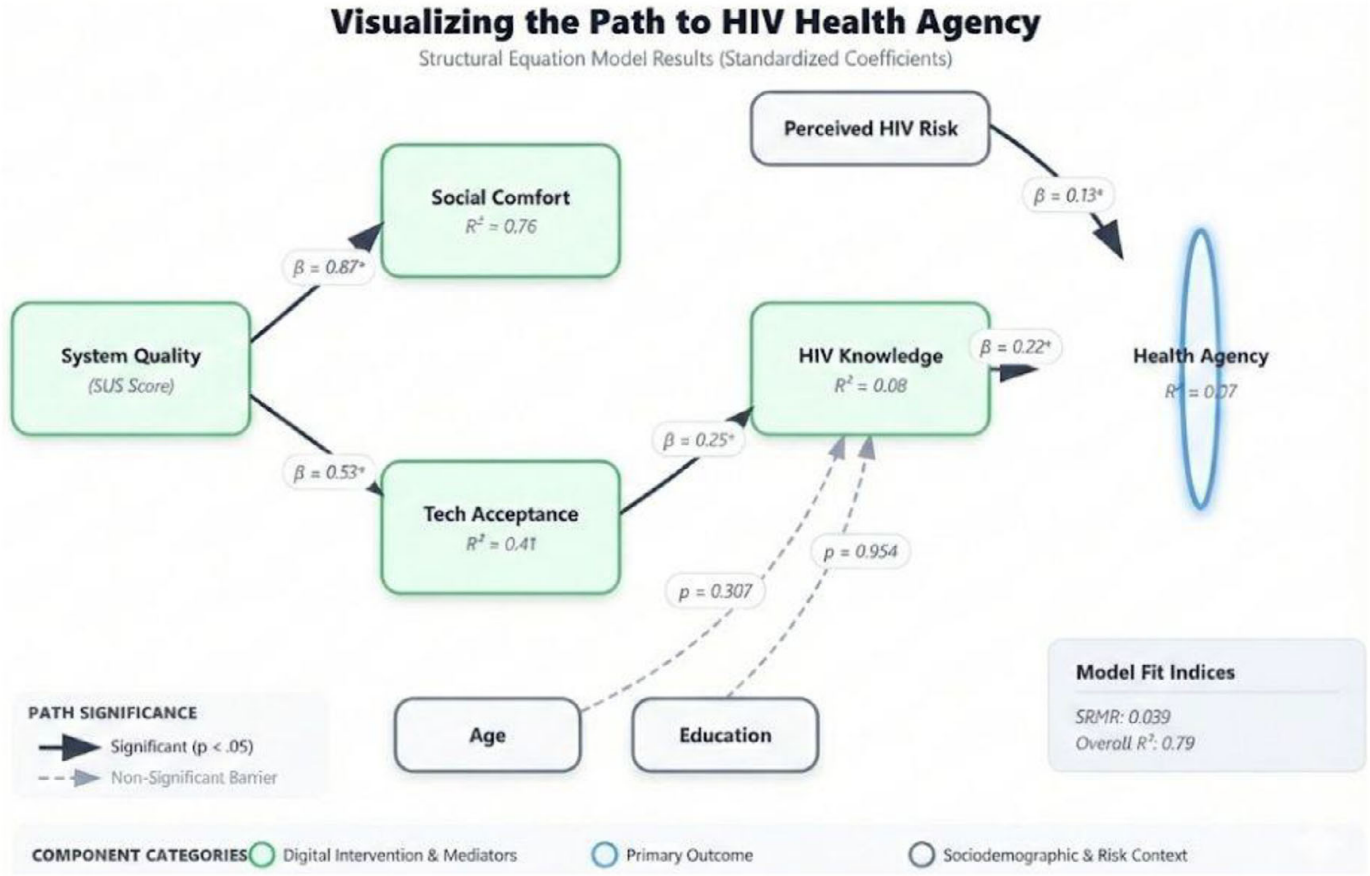
OAD3302

## OAD3303 Strategies to Improve HIV Prevention Service Uptake Among Young Women Who Sell Sex in Sub‐Saharan Africa: A Scoping Review

N. M. Prudence Tatiana ^1,2,3^, C. Noestlinger^1^, J. Vanhamel^4^, K. Michielsen^3^, P. Achiangia Njukeng^2,5^, B. H. Hensen^1^

^1^Institute of Tropical Medicine, Antwerp, Department of Sexual Health Including HIV, Antwerp, Belgium; ^2^Global Health Systems Solution, Public Health, Douala, Cameroon; ^3^KU Leuven, Institute for Family and Sexuality Studies, Leuven, Belgium; ^4^University of the Witwatersrand, School of Public Health, Faculty of Health Sciences, Johannesburg, South Africa; ^5^University of Buea, Faculty of Science, Buea Came, Cameroon

**Background**: Young women engaged in selling sex in sub‐Saharan Africa are disproportionately vulnerable to HIV due to factors like young age, stigma, violence, economic hardship, limited negotiating power and restrictive norms. These factors limit access to HIV prevention service such as pre‐exposure prophylaxis (PrEP) and worsen HIV‐related outcomes. Despite this, young women who sell sex are not adequately prioritized in regional strategies. This review synthesizes evidence on factors influencing HIV service engagement among young women who sell sex, aged 15−24 in sub‐Saharan Africa and strategies to improve uptake.

**Methods**: We systematically searched PubMed, Embase and Web of Science for peer‐reviewed studies that either described factors influencing HIV prevention service uptake among young women who sell sex or HIV prevention strategies targeting this group. We identified 30 studies for analysis of which six were interventional and 24 were observational. We employed the Capability, Opportunity, and Motivation Model of Behaviour to map factors influencing service uptake and used the Behaviour change wheel to determine the policy options and intervention types for tailored HIV prevention strategies.

**Results**: Findings showed that physical and psychological capability to use services were enhanced by community mobilization, peer education and mobile health initiatives. Motivation to engage with HIV prevention services was enhanced through community empowerment, social protection and peer support. Social opportunities were strengthened through peer‐led outreach, participatory approaches, healthcare provider training and social support addressing support for mental health, violence and substance abuse. Physical opportunities were expanded by implementing integrated youth‐friendly services and mobile clinics. Strategies to enhance individual capability and motivation, largely affected by internal and anticipated stigma, are underexplored. There remains a need to increase PrEP awareness and access in the subregion. Peer‐led models cut across as strategies that improve service engagement at both individual and environmental levels. Mobile health and participatory approaches involving young women in design show promise but evidence remains limited.

**Conclusions**: HIV prevention efforts specifically targeting young women who sell sex require comprehensive, community‐driven and tailored approaches that address structural, social and individual barriers. However, more research particularly interventional in Central and West Africa are needed (see Figures 1 and 2).

**FIGURE 1:**
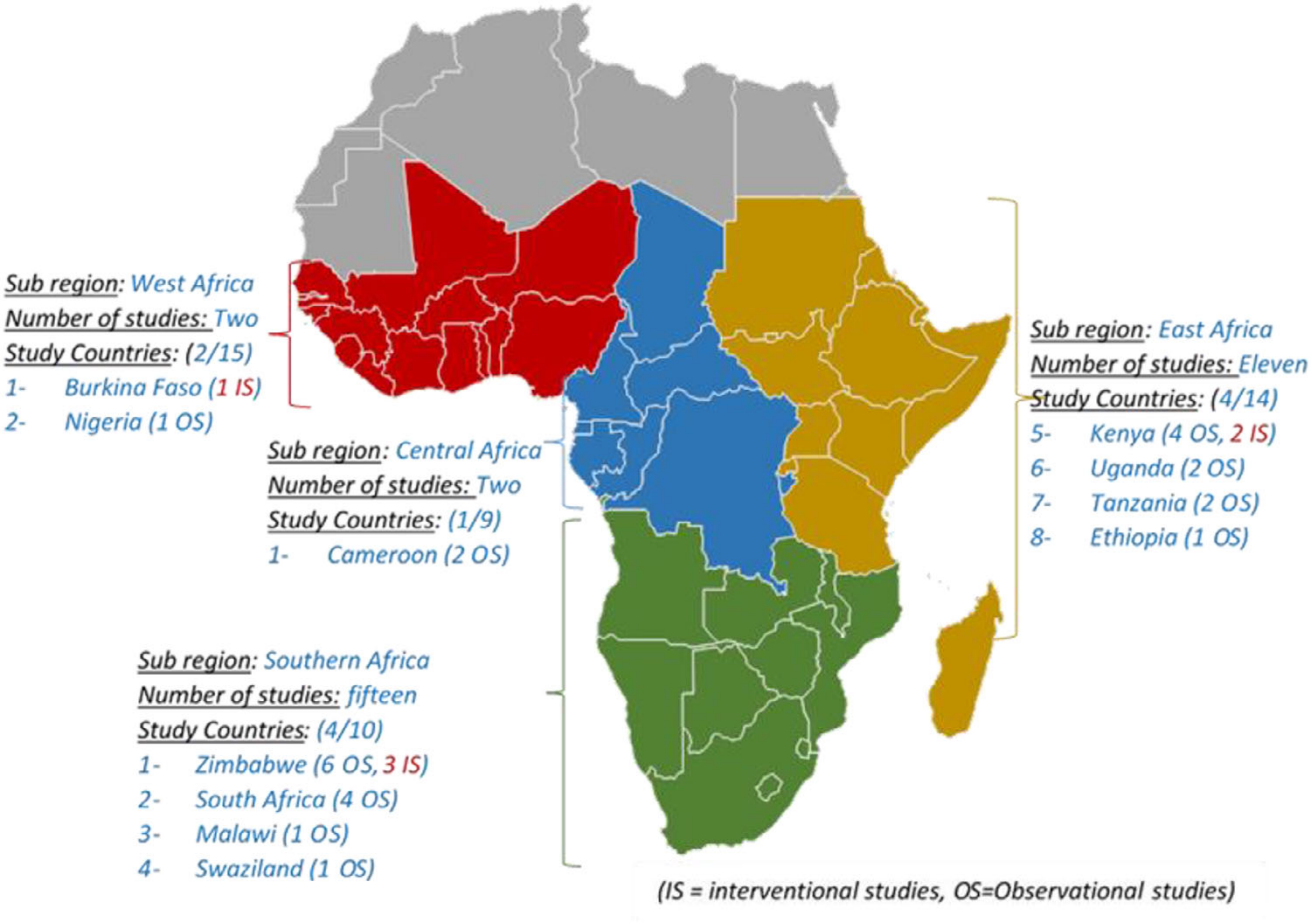
OAD3303

**FIGURE 2:**
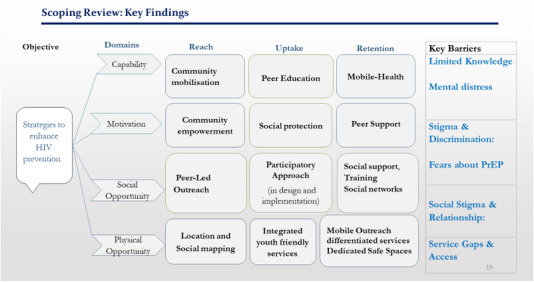
OAD3303

## OAD3304 From Play to Prevention: Reaching Rural Adolescents Through a Youth‐Led and Play‐Based HIV Prevention Package in Northern and Muchinga Provinces, Zambia

E. Soko, C. Manyele, K. Simukonda, J. Chibuye, W. Mwansa, M. Musonda, B. Mkandawire

Grassroot Soccer Zambia, Lusaka, Zambia

**Background**: Despite significant investments in HIV programming in Zambia, rural adolescents and young people (AYP) aged 10–24 years remain vulnerable to acquiring HIV, accompanied by limited AYP‐focused HIV prevention interventions that integrate play to build life‐saving behaviours. In 2025, Grassroot Soccer (GRS) Zambia partnered with the Ministry of Health (MoH) to implement a youth‐led, play‐based HIV prevention project, targeting rural AYP in Northern and Muchinga Provinces of Zambia.

**Description**: GRS trains youths aged 18−30 as “Coaches” to deliver HIV prevention and SRHR programmes with adolescents using a play‐based approach. As part of demand creation activities, Coaches, in collaboration with government health facility staff, conduct peer‐led community outreaches to provide HIV testing/SRHR services, distribution of HIV self‐test kits and demand creation for PrEP among AYP. Coaches additionally facilitate escorted referrals to health facilities for AYP to access HIV prevention services. Lastly, GRS staff and Coaches engage in stakeholder meetings with community and traditional leaders to get buy‐in for programme activities. Routine monitoring data is captured through MoH and GRS attendance/referral registers.

**Lessons Learned**:

- GRS trained 45 Coaches (18 male and 27 female) to facilitate demand creation activities in three sites, reaching 27,142 AYP (13,388 male and 13,754 female) between November 2025 and January 2026.
- The use of trained Coaches provided conducive environments for AYP to access HIV testing (*n* = 3036), HIV self‐test kits (*n* = 5188) and other HIV prevention services.
- Attaching Coaches to health facilities is cardinal in bridging gaps, such as lack of youth‐friendly services, that limit AYP from accessing HIV prevention services.
- There is a continued need to leverage on traditional structures through dialogues with traditional leaders to build support for rural AYP, especially girls, who face stigma and discrimination due to existing negative gender norms.
- GRS strengthened its partnerships with MoH by leveraging on existing MoH outreaches in remote areas to overcome distance barriers that limit reach of AYP.

**Conclusions/Next Steps**: Youth‐led, play‐based sport interventions remain promising approaches for reaching rural AYP with HIV prevention services, and GRS intends to expand this model to additional communities in rural Zambia.

## OAD3305 Effects of an Arts‐Based Intervention to Increase HIV Knowledge and Reduce HIV‐Related Stigma Among Young People Attending School in Northern Uganda: Results of a Stepped‐Wedge Cluster‐Randomized Trial

J. B. Mendelsohn ^1^, B. Fournier^2^, S. Caron‐Roy^3^, M. Sanches^4^, M. Xi^5^, C. Logie^5^, S. Ojok^6^, S. Sommerfeldt^7^, O. Bilash^7^

^1^Pace University, New York, United States; ^2^Thompson Rivers University, Kamloops, Canada; ^3^University of Saskatchewan, Saskatoon, Canada; ^4^Centre for Addiction and Mental Health, Toronto, Canada; ^5^University of Toronto, Toronto, Canada; ^6^Tochi Youth Resource Centre, Gulu, Uganda; ^7^University of Alberta, Edmonton, Canada

**Background**: HIV‐related stigma among young people in Uganda is a persistent challenge that hinders HIV prevention and care. In sub‐Saharan Africa, culturally grounded, community‐engaged, participatory arts‐based interventions have reduced stigma among young people; however, these approaches have not been evaluated in post‐conflict settings. We aimed to assess HIV knowledge, stigma and resilience in response to an arts‐based stigma intervention integrated into school curricula in post‐conflict, rural Northern Uganda.

**Methods**: We conducted an open‐cohort, stepped‐wedge cluster‐randomized trial (January−November 2023) among young people aged ≥10 years attending primary and secondary schools, randomly selected from a complete sampling frame in Omoro District, Uganda. Over 1 school year (three terms; T1/T2/T3), 12 schools (six primary, six secondary) were evenly grouped into blocks to crossover at random from control to intervention condition. Baseline and follow‐up survey questionnaires scored HIV‐related stigma (perceived, courtesy, anticipated), HIV knowledge and resilience. Analyses estimated intervention effects as post‐term change in scores (Δscore). Following intention‐to‐treat, we estimated intervention effects using linear mixed‐effect and generalized linear mixed‐effect models with random effects for youth nested within schools, adjusting for age, sex and term duration.

**Results**: We recruited 1912 youth, aged 14.9 years (mean; SD = 2.16; 51.8% female) attending primary and secondary school. Compared to no intervention, the odds of answering any HIV knowledge question correctly improved after T1 (aOR = 1.39, 95% CI: 1.16,1.66; *p* < 0.001) and continued to improve after T3 (aOR = 1.45, 95% CI: 0.99,2.14; *p* = 0.058). Perceived stigma scores increased slightly after T1 (Δscore = 0.74, 95% CI: 0.06,1.41; *p* = 0.032) but this association did not hold after T2 and T3. Courtesy stigma scores also increased slightly after each period with the largest effect observed after T3 (Δscore = 1.83, 95% CI: 0.70, 2.97; *p* = 0.002). No changes were detected in anticipated stigma or resilience after any term.

**Conclusions**: An arts‐based intervention successfully integrated into Ugandan school curricula measurably improved HIV knowledge. However, the intervention led to increases in courtesy stigma possibly related to greater awareness after having receive the intervention. Given that HIV knowledge gains tend to precede stigma reduction, longer follow‐up periods and complementary interventions are needed to translate improved knowledge and increased awareness to measurable and sustained stigma reduction among young people attending primary and secondary schools in post‐conflict settings.

## OAD3602 Project IMPACT: Behavioural Activation With Integrated Risk Reduction Counselling Among Men Who Have Sex With Men Significantly Reduces Stimulant Use and HIV Sexual Risk Behaviour–Results of an Efficacious Randomized Controlled Trial

M. J. Mimiaga ^1^, D. W. Pantalone^2^, K. B. Biello^3^, J. Tian^4^, S. Blankespoor^4^, J. M. W. Hughto^3^, S. L. Reisner^5^, E. Yonko^4^, J. Segil^4^, C. O'Cleirigh^6^, K. H. Mayer^7^, S. A. Safren^8^

^1^University of California Los Angeles, Epidemiology and Psychiatry, Los Angeles, United States; ^2^University of Massachusetts Boston, Boston, United States; ^3^Brown University, Providence, United States; ^4^University of California Los Angeles, Epidemiology, Los Angeles, United States; ^5^University of Michigan Ann Arbor, Ann Arbor, United States; ^6^Harvard Medical School/Massachusetts General Hospital, Boston, United States; ^7^Harvard Medical School/Beth Israel Deaconess Medical Center, Boston, United States; ^8^University of Miami, Miami, United States

**Background**: Stimulant use disorder poses a significant/challenging issue among men who have sex with men (MSM) in the United States and potentiates sexual acquisition of HIV. Behavioural treatments that address stimulant use in MSM at risk for HIV are lacking, and pharmacological treatments have been underwhelming. This may be due to a lack of attention to behavioural health, and specifically to replacement activities. For stimulants, continued use can lead to both disengagement from other potentially positive life activities and increases in depressed mood. Behavioural activation (BA) is an evidence‐based mental health treatment that involves re‐learning to identify and engage in pleasurable, goal‐directed activities. Hypothesis: That a BA approach would facilitate the ability of MSM with problematic stimulant use to benefit from sexual risk reduction (SRR) counselling.

**Methods**: From 2017 to 2024, we conducted a multicity RCT to test the efficacy of a psychosocial intervention (Project IMPACT) that integrates SRR counselling with BA for HIV‐negative MSM to reduce problematic stimulant use and HIV acquisition risk. After two sessions of SRR, participants (*N* = 121) were randomized to either: (1) IMPACT (*n* = 71): eight BA‐oriented counselling sessions; or (2) standard of care (SOC) control (*n* = 51). Outcomes included: (1) total condomless anal sex acts not protected by antiretroviral pre‐exposure prophylaxis (CAS‐acts‐no‐PrEP); (2) total CAS‐acts‐no‐PrEP while using stimulants; and (3) total stimulant‐free days—all past 4 months. Participants were followed for 12 months and completed biospecimen collection and a quantitative assessment battery at baseline, 4, 8 and 12 months. Conceptual mediators included BA skills and depressive symptoms.

**Results**: At 12 months, CAS‐acts‐no‐PrEP in the IMPACT arm was 71% lower than in the SOC control (incidence rate ratio [IRR] = 0.29;95% CI = 0.12–0.70, *p* = 0.006). Similarly, at 12 months, the IMPACT arm had 74% fewer CAS‐acts‐no‐PrEP while using stimulants (IRR = 0.26; 95% CI = 0.10–0.67, *p* = 0.006). Participants in the IMPACT arm significantly increased their number of stimulant‐abstinent days by 87% compared to the SOC control at 12 months (IRR = 1.87;95% CI = 1.13–3.11, *p* = 0.015). Improvements in BA skills and depressive symptoms significantly mediated all outcomes.

**Conclusions**: A BA‐SRR intervention for MSM at risk of HIV acquisition significantly reduced CAS‐acts‐no‐PrEP and stimulant use over 12 months, with evidence that the intervention worked through the key target mechanisms‐of‐action. Integrating behavioural health treatment with HIV prevention may augment the potential effects of prevention interventions. Implementation science approaches for Project IMPACT are indicated.

## OAD3603 Effectiveness and Implementation Outcomes of Participatory Co‐Designed Integrated Mental Health Services for Adolescents and Young People Living With HIV in Nigeria: A Mixed‐Methods Evaluation

A. Isah ^1,2^, C. Anosike^1^, E. J. Ugochukwu^1,2^, A. Ngige^2^, C. Nwachuya^2^, G. Ezenri^2^, J. C. Onyehalu^2^, C. Idabor^2^, M. M. Abubakar^2^, B. O. Ukoha‐Kalu^2^

^1^University of Nigeria, Department of Clinical Pharmacy and Pharmacy Management, Nsukka, Nigeria; ^2^University of Nigeria, Person‐Centred HIV Research Team, Nsukka, Nigeria

**Background**: Mental health disorders adversely affect adherence, retention and viral suppression among adolescents and young people living with HIV. In resource‐limited settings, mental health services remain poorly integrated into the services rendered in HIV clinics due to workforce limitations and low acceptability. Therefore, the objective of this study was to evaluate the effectiveness, acceptability and feasibility of a participatory co‐designed mental health integration model embedded within youth HIV clinics.

**Methods**: This survey adopted a mixed‐methods longitudinal design, incorporating 96 youth peer researchers, 28 clinicians, 14 community stakeholders, as well as 30 adolescents and young people living with HIV. The team co‐designed a stepped‐care model, incorporating peer screening (using validated tools), task‐shifted counselling, digital self‐help tools and referral pathways across four youth HIV clinics in Nigeria. A pre–post evaluation was conducted among 412 adolescents and young people living with HIV over 9 months, beginning January 2025. Outcomes included distress, depressive symptom prevalence, self‐reported adherence, viral suppression and retention. Descriptive findings were presented as frequencies and percentages, while multivariable paired regression models were used to assess the impact of the intervention. Qualitative interviews assessed acceptability and implementation fidelity using thematic analysis.

**Results**: Four clinic‐integrated components of service were co‐developed by the team: peer screening, task‐shifted counselling, digital self‐help tools and referral pathways. Among the 412 participants, the median age was 19 years; 62.24% were females. Screening uptake reached 93.7%, with 41.5% screening positive for moderate–severe distress. Task‐shifted counselling completion was 82.4%, and digital tool engagement was 68.9%. Prevalence of moderate–severe depressive symptoms declined from 38.2% to 21.6% (adjusted RR = 0.57; 95% CI:0.44–0.73), while viral suppression increased from 71.5% to 81.9% (*p* = 0.004) and 9‐month retention improved by 11.3 percentage points. Qualitative data indicated high acceptability, improved youth–provider trust and strong perceived confidentiality.

**Conclusions**: Participatory co‐design enabled effective and acceptable integration of mental health services within youth HIV clinics, leading to significant improvement in the depressive symptoms, adherence, viral suppression and retention among the youth. This suggests that task‐shifted models can improve psychosocial and biomedical outcomes without overburdening specialist resources. Policymakers should incorporate participatory mental health integration into adolescent HIV service standards and financing plans.

## OAD3604 Trauma Recovery as an HIV Treatment Accelerator: Early Outcomes of Accelerated Resolution Therapy for LGBTQI Clients in Kenya

P. Odongo^1^, M. Mudany ^2^

^1^Help Reach Africa, MERL Led, Nairobi, Kenya; ^2^Help Reach Africa, Executive Directorate, Nairobi, Kenya

**Background**: LGBTQI people in Kenya experience high rates of trauma, violence and discrimination—factors that negatively affect HIV treatment adherence, retention and viral suppression. The Huduma Mtaani Project funded by Elton John Foundation in collaboration with Yale University introduced Accelerated Resolution Therapy (ART), a trauma‐focused psychotherapy, to strengthen mental health and HIV outcomes among LGBTQI clients. This evaluation reports early findings from the first Kenyan cohort.

**Methods**: Twenty‐four mental health providers were trained in ART delivery. A mixed‐methods evaluation enrolled 60 LGBTQI clients, including 42 people living with HIV (PLHIV). Clients received 3–5 ART sessions. Mental health outcomes were assessed using the PDSQ and PCL‐5 at baseline, post‐ART and 3 months. HIV treatment indicators—medication adherence, clinic attendance and viral suppression—were abstracted from routine records for the 3 months before and after ART. Fidelity was measured through standardized checklists; acceptability through LGBTQI‐affirming satisfaction surveys.

**Results**: Mental health improved significantly:

- PTSD symptoms decreased 55% (mean PCL‐5: 48→22, *p* < 0.001).
- Depression reduced 47%, anxiety 42%.
- 83% maintained gains at 3 months.

Among PLHIV (*n* = 42), improvements in mental health were accompanied by measurable HIV treatment gains:

- ART adherence increased from 68% to 89% (+21 points).
- Missed appointments dropped 41%.
- Viral suppression improved from 62% to 81%.
  Improvements were greatest among clients with high baseline trauma burden.

Implementation findings showed high feasibility and acceptability:

- 92% of sessions met high ART‐fidelity standards.
- 96% of clients reported feeling safe, respected and affirmed.
  Providers described ART as culturally adaptable and effective for trauma linked to stigma, violence and discrimination.

**Conclusions**: Accelerated Resolution Therapy is a feasible and acceptable approach for addressing mental health co‐morbidities among LGBTQI populations in Kenya. Integration of trauma‐focused care within routine HIV services was associated with improved ART adherence, clinic attendance and viral suppression among PLHIV. High fidelity and client acceptability support the potential for scaling integrated mental health interventions to strengthen HIV treatment outcomes in key populations.

## OAD3605 Reducing HIV and Hazardous Drinking Risk and Improving Mental Health and Addiction Management During Incarceration: Learnings From Help OAT Prison Engagement Intervention

J. Rozanova ^1^, S. Shenoi^1^, M. Sabirova^2^, D. Madybaeva^2^, O. Zeziulin^3^, I. Zaviryukha^3^, I. Kharandiuk^3^, L. Grau^1^, R. Sevrukaite^1^, V. Fedorchenko^4^, A. Deac^1,5^, F. Altice^1^, N. Shumskaya^2^

^1^Yale University, School of Medicine, New Haven, United States; ^2^Public Foundation Den Soluuk Nuru, Bishkek, Kyrgyzstan; ^3^Ukrainian Institute on Public Health Policy, Kyiv, Ukraine; ^4^Independent researcher, Kyiv, Ukraine; ^5^University of East London, London, United Kingdom

**Background**: In Kyrgyzstan 10% of carceral population have HIV and 1/3 have OUD, but only 15% of eligible prisoners receive MOUD. Unmet mental health needs fuel in‐prison HIV risk behaviours and must be addressed by HIV prevention efforts alongside increasing MOUD uptake.

**Methods**: With lived‐experience partners, we co‐developed HOPE (Help OAT Prison Engagement) intervention to improve coping skills during incarceration. 12 weekly 90‐min group sessions addressed self‐acceptance; autonomy; stress; emotions; relationships; life goals; personal growth; and managing context. Each session had a mini‐lecture by a psychologist and group activities facilitated by a peer expert‐by‐experience, applying learnings to prison life. 98 men enrolled across four prisons (HOPE = 53; control = 45) were ≥18 years, self‐reported prior opioid injection, not on MOUD in the past month and ≥12 months remaining. Follow‐up was conducted at 3, 6 and 12 months. Analyses were performed using R version 4.0.0. Baseline characteristics were summarized with descriptive statistics. Between‐group comparisons of continuous variables used Welch's *t*‐tests or Wilcoxon rank‐sum tests; categorical data used Fisher's exact tests.

**Results**: At 6 months, MOUD uptake was higher in the HOPE group than in controls (11.5% vs. 2.3%; 95% CI 4.8–24.1; *p* = 0.12), with a similar pattern at 12 months (13.2% vs. 4.4%; 95% CI 0.1–13.5; *p* = 0.18). HOPE participants had significantly greater mental health improvement by 12 months: PHQ‐9 12.5 versus 18.6 (*p* < 0.001), life satisfaction 26.1 versus 20.0 (*p* < 0.001) and resilience 3.5 versus 2.9 (*p* = 0.002). HOPE participants also showed sign of reduced hazardous drinking with a greater proportion in the lower‐risk AUDIT category at month 3 (41.9% vs. 7.7%; *p* = 0.007) and 6 (32.4% vs. 11.1%; *p* = 0.044). Higher hazardous drinking risk was significantly associated with younger age (95% CI 0.89−0.99; *p* = 0.018) at baseline and higher resilience score (95% CI 1.01, 1.31; *p* = 0.036) at month 6 in univariate analysis, and with being in Control arm (95% CI 0.12, 0.97; *p* = 0.044) at month 3 in multivariable analysis.

**Conclusions**: HOPE can simultaneously improve MOUD initiation, mental health and hazardous drinking risk in‐prison. Delivery by a psychologist and an expert‐by‐experience pair creates a credible learning environment leading to behaviour change. Adapting HOPE beyond Kyrgyzstan and examining HOPE effects sustaining post‐release is needed.

## OAD3902 One Shock After Another! From the Anti‐Gay Law to Unexpected HIV Funding Cut: A Comparative Assessment of Mental Health and Suicidality by Men Who Have Sex With Men During These Two Extremes in Uganda

D. Kazibwe ^1^, R. Kikonyogo^2^, A. Nanyonjo^3^, I. Mugagga^1^

^1^Save the Youth Uganda, Kampala, Uganda; ^2^Reach All Clinic Uganda, Kyegegwa, Uganda; ^3^Plan AIDS Support Organization, Wakiso, Uganda

**Background**: Men who have sex with men (MSM) in Uganda face elevated mental health risks driven by stigma, criminalization and limited access to HIV/AIDS health services. The signing of the Anti‐Homosexuality Act in 2023 marked a significant escalation in legal and social risk for MSM including death sentence, while the subsequent freezing of HIV donor funding in 2025 escalated their plight. Save the Youth Uganda (SYU) conducted a comparative assessment to examine the mental health and suicidality of these overlapping shocks and to inform adaptive programming for MSM.

**Description**: SYU conducted a comparative study to examine changes in mental health outcomes between the post‐law period and the post‐funding freeze period. The first survey had been completed in 2023 following the enactment of the Anti‐Homosexuality Act. A total of 142 MSM had been recruited through peer networks and assessed measuring depressive symptoms, anxiety, suicidal ideation, substance use, stigma, exposure to violence, and access to HIV and psychosocial services. In 2025, following freezing of HIV donor funding and subsequent service disruptions, a second survey though not planned, was initiated among the same cohort of MSM. Similar methods were used.

**Lessons Learned**: Following enactment of the Anti‐Homosexuality Act, 31% of MSM screened positive for moderate to severe depressive symptoms and 15% reported suicidal ideation. After the donor funding freeze, these figures increased significantly, with 44% screening positive for moderate to severe depression and 24% reporting suicidal ideation. 6% fled the country. Reports of anxiety, substance use and avoidance of health facilities also increased between the two study periods. Loss of access to trusted community‐based services was strongly associated with worsening mental health outcomes.

The findings demonstrate that while legal criminalization alone has a substantial negative impact on MSM mental health, the withdrawal of donor‐funded services further exacerbated psychological distress and suicidality. Community‐based HIV and psychosocial services serve as a critical protective factor for MSM, and their disruption magnifies the harm caused by hostile legal environments.

**Conclusions/Next Steps**: SYU will prioritize and advocate for continuity of mental health and HIV services for MSM through diversified funding, discreet and peer‐led service delivery models, and strengthened referral and crisis support systems.

## OAD3903 Community Resilience Amid an Anti‐LGBT Backlash in Côte d'Ivoire: A Community‐Led, Response Implemented by ESPACE CONFIANCE to Sustain HIV Services for Populations More Vulnerable in Emergency Situations

V. Keipo, C. Anoma, L. Zougouri, J. N'Guessan

ONG Espace Confiance, Abidjan, Côte d'Ivoire

**Background**: Human rights violations and structural stigma remain major barriers to HIV service access for populations at higher risk for HIV(KPs). Between August and September 2024, Côte d'Ivoire experienced an unprecedented escalation of anti‐LGBT violence fuelled by disinformation and hostile narratives amplified through social media. This backlash led to physical assaults, forced displacement, extortion and threats, severely restricting the mobility and visibility of men who have sex with men (MSM) and transgender persons (TG). Consequently, access to HIV prevention and treatment services was critically disrupted. In this rights‐restrictive context, ESPACE CONFIANCE implemented an emergency, community‐led response to protect individuals at highest risk and sustain essential HIV services.

**Description**: A rights‐based, community‐centred intervention integrated protection, service continuity and participatory engagement. Emergency shelter mechanisms were activated to provide immediate protection to individuals under direct threat, in coordination with LGBT civil society partners. To uphold continuity of care despite mobility constraints, a secure home‐based delivery system for antiretroviral therapy (ART), PrEP and HIV self‐testing kits was implemented using sensitized community couriers. In parallel, a participatory consultation—combining an online survey and 10 focus group discussions across multiple districts of Abidjan, documented human rights violations, emerging risks and priority community needs. Psychosocial support and reinforced security measures for community spaces complemented the response.

**Lessons Learned**: The intervention enabled the immediate protection of 15 individuals facing imminent threats. Home‐based delivery ensured uninterrupted HIV services for 127 beneficiaries, including 80 people living with HIV and 47 PrEP users, preventing treatment interruptions. Among 107 consultation participants, 38 described homophobia as “extremely severe” and 52 as “alarming.” Documented impacts included physical violence, job loss, family rejection, cyberharassment and avoidance behaviours with significant mental health implications. Despite these challenges, retention in HIV services remained high and community trust was strengthened.

**Conclusions/Next Steps**: This experience demonstrates that community‐led, rights‐based responses are critical to sustaining HIV services for KPs during periods of acute hostility. Integrating protection, adaptive service delivery and participatory monitoring strengthens resilience, safeguards rights and preserves continuity of care in crisis settings.

## OAD3904 Turning Visibility Into Protection: Implementation Lessons in Coordinated Media Advocacy Programme Advancing HIV and SRHR Rights for LGBTIQ+ Key Populations in Nigeria

T. Opatola ^1^, R. Oyeniyi^2^, O. Olajubu^3^

^1^The Momentum Support Initiatives, Monitoring and Evaluation, Lagos, Nigeria; ^2^The Momentum Support Initiatives, Director, Lagos, Nigeria; ^3^The Rainbow Alive Hub, Monitoring and Evaluation, Lagos, Nigeria

**Background**: In Nigeria, LGBTIQ+ persons and other key populations face intersecting behavioural, social and structural barriers to HIV prevention, treatment and care, driven by criminalization, stigma and hostile public discourse. Media coverage of HIV and SRHR involving key populations frequently relies on medicalized, moralistic or exclusionary framing, shaping public attitudes and reinforcing discrimination within healthcare settings. While visibility is necessary for advocacy, uncoordinated media engagement can increase exposure to violence, arrest and service denial without shifting dominant narratives or policies. In the lead‐up to World AIDS Day (WAD) 2024, fragmented advocacy efforts risked reproducing harmful stereotypes rather than advancing rights‐affirming HIV responses. TMSI developed a coordinated, community‐led media advocacy programme to address these structural determinants affecting key populations.

**Description**: In December 2024, TMSI implemented a three‐phase media advocacy programme in Lagos and Ogun States with support from Y+ Global. The programme engaged 20 young LGBTIQ+ and other key population activists living with HIV, alongside representatives of grassroots organizations working on HIV. Phase 1 focused on capacity strengthening through a 2‐day training on rights‐based narrative framing, ethical use of lived experience, visual storytelling, misinformation management and digital safety. Phase 2 involved collective action through a synchronized 4‐week campaign across more than 20 organizations, integrating social media storytelling, community radio interviews and press engagement. Phase 3 translated online engagement into offline influence through media roundtables and policy dialogues with journalists, state‐level health officials and human rights actors, focusing on discriminatory healthcare practices affecting key populations.

**Lessons Learned**: The programme generated 300,000+ digital engagements and a 40% increase in participants’ media competence. Significantly, 45% of organizations replicated the model, and media monitoring documented a shift towards rights‐affirming language in major outlets. Crucially, policy dialogues secured written commitments from Lagos and Ogun state health officials to review discriminatory service delivery practices.

This includes:

1. Synchronized messaging reduces individual risk while amplifying collective impact.
2. Peer‐led storytelling effectively counters misinformation.
3. Linking media advocacy to policy engagement strengthens accountability within HIV programmes.

**Conclusions/Next Steps**: This programme demonstrates that coordinated, community‐led media advocacy can address behavioural and structural drivers of HIV vulnerability among key populations. Integrating capacity building, collective action can support inclusive HIV responses.

## OAD3905 Navigating HIV Funding Declines: Ally Heart's Peer‐Led Digital Cross‐Subsidization Model for Integrated Gender‐Affirmative Care in India

Shayna, L. Arora, A. Khan, R. J, U. Swetha

Ally Heart, Delhi, India

**Background**: Global HIV funding crises, including 2025 PEPFAR reductions risking 1.5 million excess Acquisitions, exacerbate disparities for transgender populations in low‐resource settings. Community‐based LGBTQIA+ services remain donor‐dependent, limiting scalability and integration with mental health and gender‐affirming care. Ally Heart addresses this through a digital, community‐led platform delivering affirmative healthcare to 5000+ LGBTQIA+ clients in India since 2024.

**Description**: The model integrates HIV services (testing, referrals, PrEP/ART linkage) with gender‐affirmative care (hormone therapies, gender dysphoria certification, mental health counselling) via an AI Chatbot‐based telehealth system. Peer‐led cross‐subsidization drives sustainability: a hybrid revenue approach (user‐paid services, subscriptions, corporate partnerships and limited grants) covers 63% of operational costs and 95% of marketing, while cross‐subsidization supports free/low‐cost access for marginalized users. Implementation spans urban‐rural India, with peer navigators ensuring cultural safety and digital tool training.

**Lessons Learned**: From 2024 to 2025, Ally Heart reached over 5000 transgender and gender diverse individuals across multiple Indian cities through its peer‐led digital platform. The solidarity‐based financing model “Those Who Can Pay, Support Those Who Cannot” operated via peer‐driven cross‐subsidization: revenue from user‐paid premium services (e.g. subscriptions for gender dysphoria certification, hormone therapy navigation and corporate wellness partnerships) covered over 60% of operational costs and marketing expenses, subsidizing free or low‐cost access to integrated HIV testing/referrals, mental health counselling and gender‐affirmative care for economically marginalized users. This reduced unrestricted donor funding dependence by over 90%, enabling uninterrupted service delivery despite global funding constraints. Digital integration via the AI Chatbot achieved 98% completion of referred HIV services also underscoring high unmet needs met through community solidarity. Peer‐led governance, including navigators and feedback loops, built trust and cultural safety, with over 70% of users accessing multiple service domains (e.g. combined HIV linkage, gender‐affirmative care and psychosocial support).

**Conclusions/Next Steps**: This community‐owned digital model demonstrates peer‐driven cross‐subsidization can enhance sustainability, integration and equity in gender‐affirmative HIV care amid funding constraints. It offers a replicable pathway beyond donor dependence, centring community leadership. Policy recommendations: Integrate such platforms into national UHC frameworks for key populations. Next steps: Multi‐site scaling, cost‐effectiveness modelling, and trials to quantify long‐term viral suppression and health equity gains.

## OAE0302 Machine Learning for Risk Factor Identification and Outcome Improvement in Prevention of Vertical HIV Transmission in Nigeria

R. Abdullahi ^1^, S. Adetoye^1^, O. Ogorry^2^, O. Airoje^2^, S. Mawandia^2^, C. Showalter^3^, L. Claussen^4^, N. Nigussie^5^, K. Ofim^6^

^1^The Palladium Group – Data for Implementation (Data.FI) Nigeria, Health Informatics, Abuja, Nigeria; ^2^The Palladium Group – Data for Implementation (Data.FI) Nigeria, Management, Abuja, Nigeria; ^3^The Palladium Group – Data for Implementation (Data.FI) Nigeria, Monitoring and Evaluation, Abuja, Nigeria; ^4^The Palladium Group – Data for Implementation (Data.FI) Nigeria, Knowledge Management, Abuja, Nigeria; ^5^The Palladium Group – Data for Implementation (Data.FI) Nigeria, Digital Programs, Abuja, Nigeria; ^6^The Palladium Group – Data for Implementation (Data.FI) Nigeria, Communications, Abuja, Nigeria

**Background**: Despite targeted prevention of vertical transmission programmes, vertical HIV transmission during pregnancy, delivery or breastfeeding remains a critical challenge. Nigeria has the largest paediatric HIV burden, accounting for approximately one in four new childhood HIV acquisitions globally despite having less than 3% of the world's population. Existing tools cannot reliably predict vertical transmission risk among pregnant women living with HIV or explain underlying risk drivers. To address this gap, we used machine learning to identify risk factors and improve prevention of vertical transmission outcomes.

**Methods**: We analysed de‐identified records of 1000 mother−infant pairs from Nigerian prevention programmes (318 infants acquired HIV; 682 did not). We examined seven clinical variables: maternal age, antiretroviral therapy (ART) initiation timing, viral load, CD4+ count, delivery mode, feeding method and medication adherence. We trained four models on 800 cases using stratified five‐fold cross‐validation with resampling to address class imbalance, then tested on 200 separate cases. We applied SHAP (SHapley Additive exPlanations) analysis to identify factors driving individual risk predictions.

**Results**: The Random Forest model achieved the best performance with 80% accuracy, comparable to existing clinical prediction tools, while identifying modifiable risk factors that could prevent two‐thirds of high‐risk transmissions. The model achieved 69.8% precision, 68.8% sensitivity and ROC‐AUC of 0.773 (ability to distinguish transmission cases), outperforming Logistic Regression, XGBoost and Neural Network models. SHAP analysis identified ART initiation timing, CD4+ count and medication adherence as dominant modifiable risk factors. Risk stratification showed women with 95% or higher adherence and undetectable viral load faced 1.0% transmission risk versus 8.7% for those with 60% adherence and high viral load. Simulations showed intensive support at mid‐pregnancy reduced transmission risk by 64%.

**Conclusions**: We developed an explainable machine learning approach to achieve 80% accuracy in predicting vertical HIV transmission. For clinicians, this enables early identification of high‐risk pregnancies and reveals which modifiable factors to prioritize. For programmes, our finding that two‐thirds of high‐risk transmissions are potentially preventable justifies investment in early intervention. For Nigeria, this could help prevent thousands of the estimated 15,000 annual vertical transmissions by directing resources towards pregnant women who need support most (see Figures 1 and 2).

**FIGURE 1:**
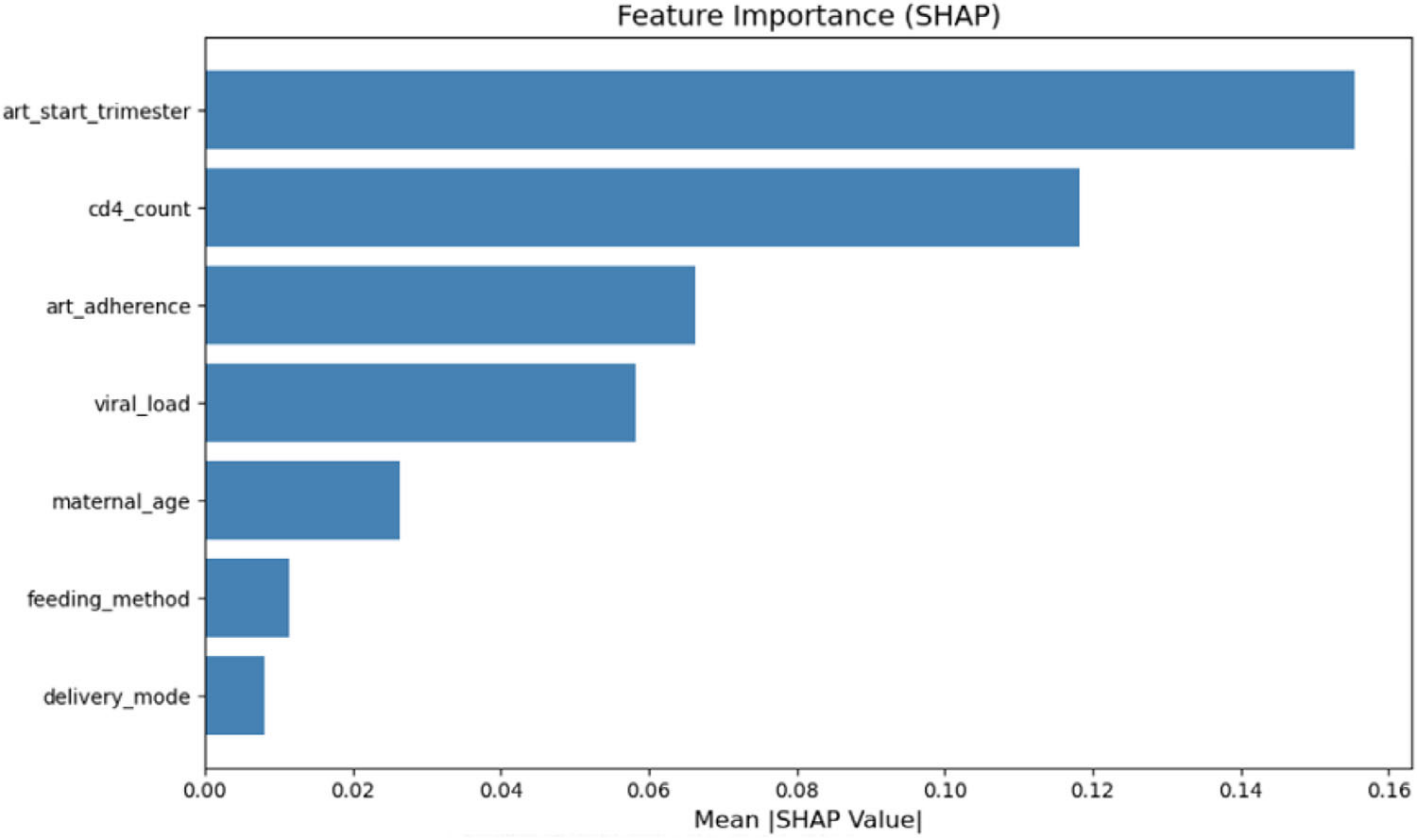
OAE0302

**FIGURE 2:**
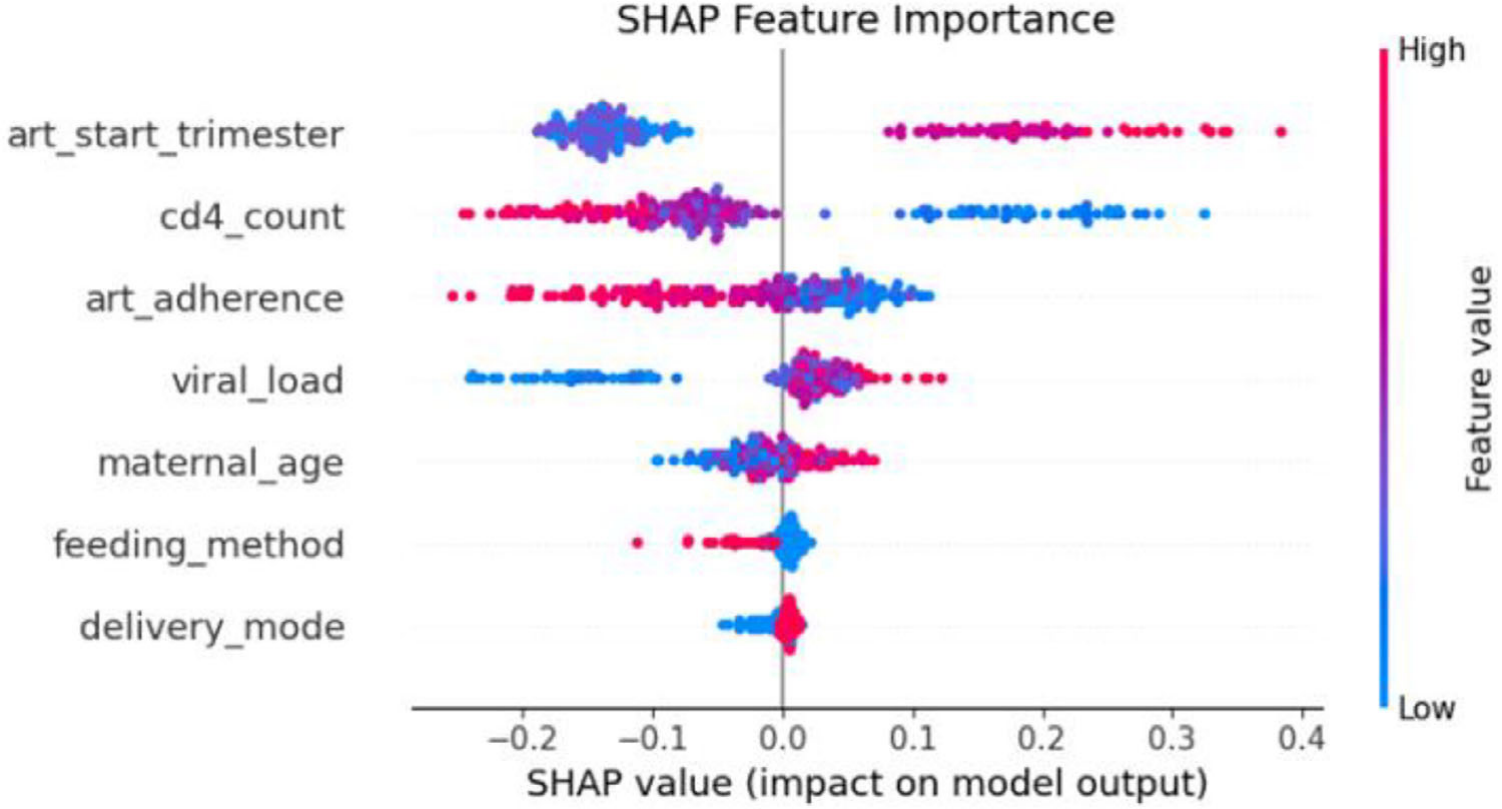
OAE0302

## OAE0303 Machine Learning Framework for Predicting Antiretroviral Therapy Adherence: Early Warning System for Latin American Healthcare Providers

S. K. Nuñez Pumacallahui

Universidad Tecnológica del Perú, Lima, Peru

**Background**: Antiretroviral therapy (ART) adherence in Latin America remains suboptimal, with many people on ART experiencing challenges in maintaining consistent treatment during the first year. Current monitoring systems often detect adherence concerns only after biological or clinical consequences appear, limiting opportunities for early support. This project addresses this gap by developing a machine learning framework that anticipates adherence challenges 30–60 days in advance using publicly available, de‐identified datasets. The objective is to explore how open data and explainable artificial intelligence can strengthen early support strategies in resource‐constrained Latin American settings.

**Description**: A mixed‐methods approach was applied, combining model development with implementation science principles. Multiple open datasets containing longitudinal indicators related to ART refills, appointment attendance, medication pick‐up behaviour and demographic information from Latin America and global contexts were harmonized into a unified structure. Temporal features were engineered to capture behavioural trends, such as increasing delays in refills or decreasing engagement in routine visits. Models evaluated included logistic regression, random forest, gradient boosting and LSTM networks, using training, validation and temporal testing to ensure stability over time. SHAP values were applied to generate interpretable outputs simulating a decision‐support interface for healthcare providers. Insights from digital health practitioners and people with lived experience of HIV care informed design considerations to ensure usability, acceptability and ethical implementation.

**Lessons Learned**: Findings show that the gradient boosting model achieved an average AUC of 0.82 across three independent datasets and remained robust under temporal validation. Behavioural trajectories consistently predicted adherence challenges more effectively than static demographic variables, reinforcing the importance of dynamic, person‐centred indicators. Best practices include the critical role of dataset harmonization, the contribution of temporal features which improved performance by up to 10 percentage points and the necessity of transparent output explanations for future clinical use.

**Conclusions/Next Steps**: These results demonstrate that publicly accessible datasets can be leveraged to build predictive tools that support proactive adherence management without accessing sensitive clinical records. The next phase will focus on developing a prototype early warning system co‐designed with patients, clinicians and community advocates, and exploring collaborations with Latin American digital health initiatives to evaluate adaptation in local contexts.

## OAE0304 “Used Right, It Can Save Lives”: Community Voices on AI‐Driven HIV Risk Prediction in Atlanta, GA

J. D. Patino‐Mateus^1^, N. Quiroga‐Gutierrez^1^, J. S. Ramon^1,2^, T. Moscovich^2^, C. E. Ordóñez^3,4^, L. Mena^1^, C. S. Saldana ^1,5^

^1^Emory University, Infectious Diseases, Atlanta, United States; ^2^Emory University, Rollins School of Public Health, Atlanta, United States; ^3^University of the Witwatersrand, School of Public Health, Johannesburg, South Africa; ^4^Emory University, Rollins School of Public Health, Hubert Department of Global Health, Atlanta, United States; ^5^Emory University, Emory Center for AIDS Research (P30 AI050409), Atlanta, United States

**Background**: Machine learning (ML) models are emerging tools for HIV prevention, using clinical and public health sexually transmitted infection (STI) surveillance data to flag individuals at elevated acquisition risk and tailor testing, prevention, and education. However, the perspectives of interest holders, including members from surveilled communities, regarding this use of ML, remain understudied. We examined perspectives among members of HIV priority communities in Atlanta, GA to guide the future implementation of our predictive ML model.

**Methods**: We conducted 60‐min in‐depth interviews (IDIs) in English and Spanish with LGBTQ+ individuals. Prior to the IDIs, participants completed a sociodemographic survey and watched an introductory video that covered basics and applications of ML for HIV prediction. The IDI guide explored artificial intelligence (AI)/ML understandings, views on surveillance data use, conditions for community trust and ethical implementation recommendations. Survey results were summarized descriptively, and qualitative data were analysed using a rapid qualitative analysis approach, incorporating both deductive and inductive thematic analysis.

**Results**: Table 1. Participant characteristics (*N* = 20). All measures were self‐reported. Levels of concern reflect responses following educational video.

Among 20 participants, most were aged 25–34 years and identified as Latino/a/e and Black/African American. Most participants reported familiarity with AI/ML and expressed moderate to high concern about the use of STI data in predictive models (Table 1). Five cross‐cutting themes (Figure 1) emerged:

1. ML was seen as valuable for guiding resource allocation, tailoring outreach and supporting education;
2. community trust depends on strong privacy protections, de‐identification and transparency;
3. participants expressed concerns about data misuse and stigma, particularly if models target vulnerable groups and reinforce existing inequities;
4. concerns about model design and performance included quality of training data, representation biases and fairness; and
5. linking models to existing, actionable prevention and care services was seen as essential for achieving tangible outcomes.

These themes were mapped to ML use and implementation recommendations by the study team.

**Conclusions**: Community members believed ML could strengthen HIV prevention but emphasized the need for transparency, privacy safeguards and meaningful community engagement. Predictive tools should be clearly connected to tangible services to ensure acceptability and utility.

**TABLE 1:** OAE0304

Characteristics	Participants *n* (%)
**Age**	
18−24	2 (10)
25−34	16 (80)
35−44	2 (10)
**Race/ethnicity**	
Latino/a/e/x	10 (50)
Black/African American	9 (45)
White	1 (5)
**Primary language spoken at home**	
Spanish	8 (40)
English	11 (55)
Both (bilingual)	1 (5)
**Gender identity**	
Cisgender woman	2 (10)
Cisgender man	12 (60)
Transgender and/or gender diverse	6 (30)
**Sexual orientation**	
Straight/heterosexual	1 (5)
Gay	10 (50)
Lesbian	1 (5)
Bisexual	7 (35)
Other	1 (5)
**HIV status**	
Positive	8 (40)
Negative	8 (40)
Unknown	4 (20)
**Prior STI diagnosis**	
Yes	13 (65)
No	7 (35)
**AI/ML familiarity**	
High (very familiar)	12 (60)
Moderate (somewhat familiar)	5 (25)
Low/non‐familiar	3 (15)
**Concern about using STI surveillance data for AI/ML**	
High (very concern)	6 (30)
Moderate (somewhat concern)	5 (25)
Low/no concern	9 (45)

**FIGURE 1:**
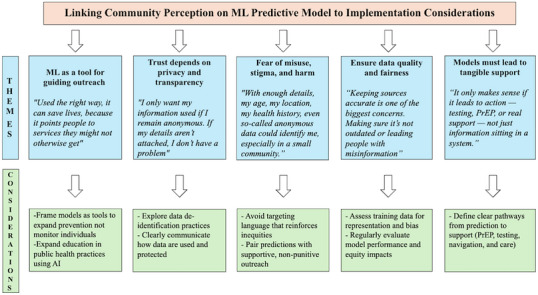
OAE0304

## OAE0305 AI‐Assisted Point‐of‐Care Radiologic Presumptive Tuberculosis Across HIV Strata Using Radiological and Laboratory Ground Truths in a Retrospective Multicentre Brazilian Dataset

A. C. Garcia Ferreira ^1^, M. C. Bueno da Silva^1^, G. A. Sousa Ribeiro^1^, J. Driemeyer Correia Horvath^1^, G. de Souza Mendes^1^, C. Pereira Kuss^1^, P. V. Alves Silva^1^, D. Silva Rodrigues^2^, R. G. Feijó Andrade^3^, N. Rolim Gonçalves de Alencar^4^, S. Sakabe^5^, E. R. Vieira Gratão Cordeiro^6^, R. Spener‐Gomes^7,8,9^, F. Teixeira^10^, V. de França Diniz Rocha^11^, P. Bresciani Martins de Andrade^1^, M. G. de Almeida Rodrigues^1^, R. C. Mendes^1^, J. R. Mendes Andrade^3^, E. de Barros Campelo Júnior^4^, J. Peres Queiroz de Paiva^1^

^1^Albert Einstein Instituto de Ensino e Pesquisa, PROADI‐SUS, São Paulo, Brazil; ^2^Instituto Clemente Ferreira da Secretaria de Estado da Saúde, São Paulo, Brazil; ^3^Faculdade Medicina da UFRGS e Hospital de Clínicas de Porto Alegre, Porto Alegre, Brazil; ^4^Hospital das Clínicas da Universidade Federal de Pernambuco, Recife, Brazil; ^5^Centro de Referência e Treinamento DST Aids São Paulo, São Paulo, Brazil; ^6^Hospital Municipal de Aparecida de Goiânia (HMAP), sob gestão do Einstein Hospital Israelita, Goiânia, Brazil; ^7^Universidade Federal do Amazonas (UFAM), Manaus, Brazil; ^8^Fundação de Medicina Tropical Doutor Heitor Vieira Dourado, Manaus, Brazil; ^9^Universidade do Estado do Amazonas (UEA), Manaus, Brazil; ^10^Complexo Hospital das Clínicas da Universidade Federal do Paraná (UFPR), Curitiba, Brazil; ^11^Instituto Couto Maia, Salvador, Brazil

**Background**: Early identification of pulmonary tuberculosis (TB) remains challenging in primary and emergency health services, particularly among people living with HIV (PLHIV). Artificial intelligence (AI) tools supporting radiologic presumptive TB may strengthen implementation of integrated TB/HIV pathways, especially where expertise and workflow constraints limit diagnostic performance. We evaluated an AI model in development by multicentre partners to Brazilian Public Health System (SUS). The model is designed to assist radiologic presumptive TB, embedded in a point‐of‐care workflow, comparing its performance across HIV serostatus using both radiological and laboratory reference standards (ground truths).

**Methods**: We retrospectively analysed chest radiographs (CXR) from a multicentre Brazilian dataset (*n* = 500). Most of the retrospective image selection was guided by TB laboratory confirmation, resulting in a high laboratory confirmed TB prevalence (90%). Two ground truths were applied:

1. radiological (thoracic radiologist presumptive TB report) and
2. laboratory (microbiologic/molecular confirmation).

Sensitivity, specificity, PPV, NPV, F1‐score, AUC and net benefit were evaluated. Analyses were stratified by HIV status (positive, negative, unknown), for the full set and excluding HIV‐unknown.

**Results**: Among PLHIV in the digital dataset (*n* = 252), radiological presumptive TB by radiologist was 41.2%, laboratory confirmation 82.1% and concordance between both 36.9%.

Against the radiological ground truth, performance using AI evaluation of digital images in PLHIV (*n* = 252) was: sensitivity 0.76, specificity 0.64, AUC 0.77; HIV‐negative (*n* = 191), sensitivity 0.84, specificity 0.39, AUC 0.66.

Against the laboratory ground truth, AI evaluation of digital images in PLHIV (*n* = 252) showed: AUC 0.65 (PPV 0.88, NPV 0.24); among HIV‐negative, AUC 0.89 (only four TB‐negative cases).

**Conclusions**: The AI model demonstrated consistently high sensitivity compared with thoracic radiologist presumptive TB in PLHIV, supporting its potential role as a triage tool in emergency and primary healthcare TB/HIV pathways. However, when compared with the microbiological gold standard, AI performance was moderate, with reduced discriminatory ability particularly among PLHIV, reflecting the well‐known complexity and heterogeneity of TB radiographic presentations in this population. At the same time, performance against the laboratory standard was strongly influenced by the high TB prevalence, leading to high PPV but low NPV. Prospective validation and workflow integration studies are warranted to optimize implementation in this population.

## OAE0502 Long‐Acting Cabotegravir Plus Rilpivirine for People Living With HIV and Suboptimal Viral Suppression: Trial‐Based Lifetime Cost‐Effectiveness

H.‐J. Wu ^1^, S. W.‐W. Ku^2^, C.‐W. Li^3^, C. Strong^4^, C.‐Y. Cheng^5,6^, N.‐Y. Chen^7,8^

^1^The Kirby Institute, UNSW, Sydney, Australia; ^2^Division of Infectious Diseases, Department of Medicine, Taipei City Hospital Renai Branch, Taipei, Taiwan, Province of China; ^3^Division of Infectious Diseases, Department of Internal Medicine, National Cheng Kung University Hospital, College of Medicine, National Cheng Kung University, Tainan, Taiwan, Province of China; ^4^Department of Public Health, College of Medicine, National Cheng Kung University, Tainan, Taiwan, Province of China; ^5^Department of Infectious Diseases, Taoyuan General Hospital, Ministry of Health and Welfare, Taoyuan, Taiwan, Province of China; ^6^Institute of Public Health, School of Medicine, National Yang‐Ming Chiao Tung University, Taipei, Taiwan, Province of China; ^7^Division of Infectious Diseases, Department of Internal Medicine, Linkou Chang Gung Memorial Hospital, Taoyuan, Taiwan, Province of China; ^8^School of Medicine, Chang Gung University, Taoyuan, Taiwan, Province of China

**Background**: People living with HIV (PLWH) and suboptimal adherence to antiretroviral therapy (ART) face high risks of virologic failure and onward HIV transmission, yet remain underserved by current treatment options. Long‐acting injectable cabotegravir plus rilpivirine (LA CAB+RPV) offers a promising alternative to daily oral ART, but its indication is currently restricted to virologically suppressed individuals. Emerging evidence from the Phase IIIb SUPLA trial (NCT06507059) supports the clinical benefit of LA CAB+RPV in this population, but cost‐effectiveness data are needed to inform policy. We use SUPLA data to estimate the lifetime cost‐effectiveness of LA CAB+RPV initiation among PLWH with suboptimal ART adherence and to explore the impact of incorporating costs averted through reduced HIV transmissions from a healthcare payer perspective in Taiwan.

**Methods**: We adopted a hybrid decision‐tree/Markov model to compare LA CAB+RPV versus daily oral ART among PLWH with suboptimal ART adherence in Taiwan over a lifetime horizon (Figure 1).

Clinical inputs were derived from the SUPLA trial. Costs for ART, administration, HIV‐1 genotyping and AIDS‐defining events were obtained from Taiwan's National Health Insurance Research Database and reported in US dollars (1 USD ≈ 30 New Taiwan dollars) in 2025 and utilities from published literature. We estimated incremental cost‐effectiveness ratios from a healthcare payer perspective with 3% annual discounting and modelled averted HIV transmissions.

**Results**: Over a lifetime horizon, immediate initiation of LA CAB+RPV yielded a gain of 2.25 additional QALYs and increased discounted lifetime costs by US$61,667 per patient compared with daily oral ART, resulting in an ICER of US$27,432/QALY, below the 1 × GDP per capita cost‐effectiveness threshold in Taiwan (US$37,827). When lifetime costs of secondary HIV acquisitions were incorporated, LA CAB+RPV averted 10.3 HIV acquisitions per treated patient. At an estimated US$163,333 lifetime cost per acquisition, this translated into net savings of US$1.6 million per patient with the same health gains, making LA CAB+RPV a dominant (more effective and less costly) strategy.

**Conclusions**: Among PLWH with suboptimal adherence to oral ART, LA CAB+RPV substantially improves health outcomes and is cost‐effective compared with standard daily oral ART. When prevention benefits are included, LA CAB+RPV becomes cost‐saving from the Taiwan National Health System perspective.

**FIGURE 1:**
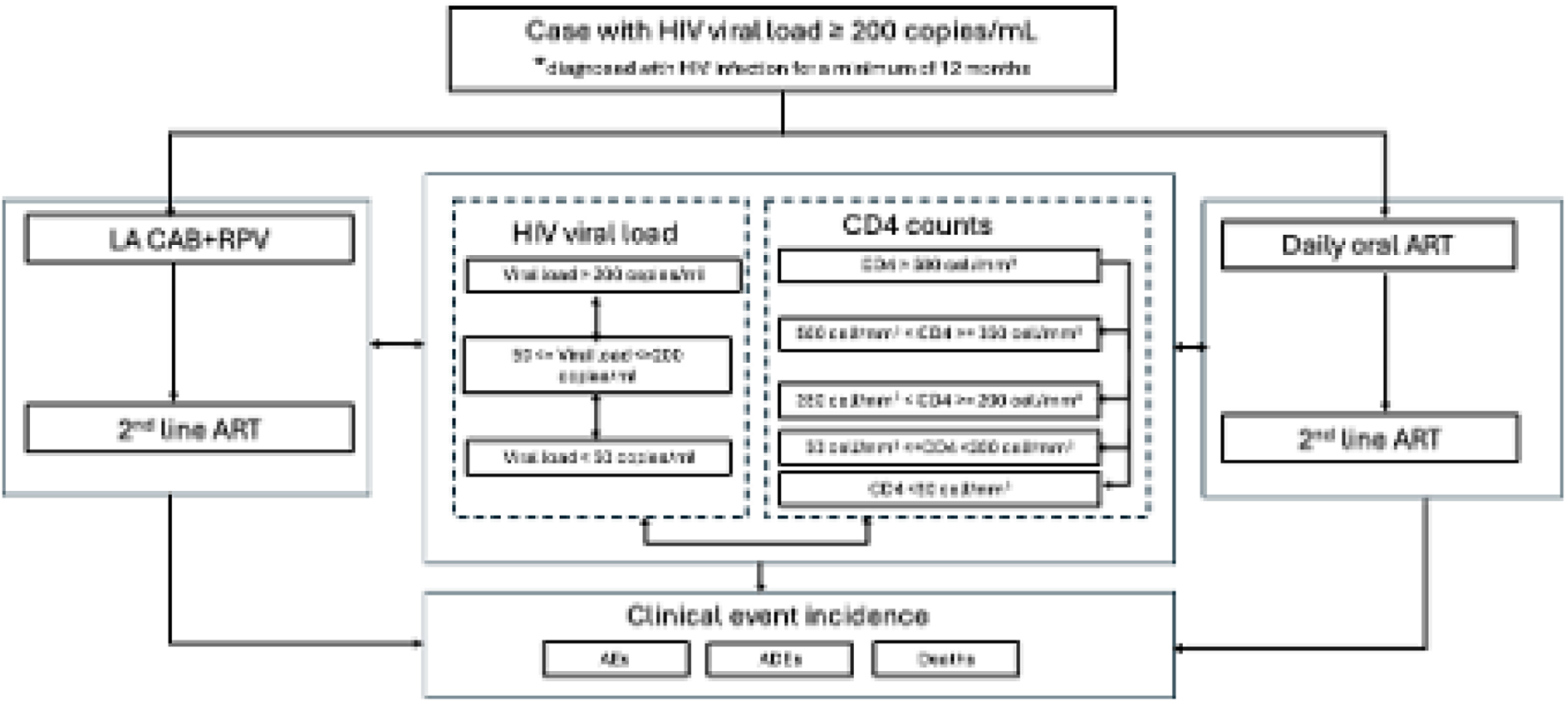
OAE0502

## OAE0503 Costing the Implementation of 6MMD Utilizing 6‐ and 12‐Month Scripting in South Africa

N. Muwanula, A. Huber

University of Witwatersrand – Wits Health Consortium, Health Economics and Epidemiology Research Office, Johannesburg, South Africa

**Background**: The South African National Department of Health (NDoH) is scaling up 6‐month multi‐month dispensing (6MMD) of antiretroviral therapy (ART) to improve treatment access and retention and reduce crowding in healthcare facilities. While client health outcomes of 6MMD have been shown to be equivalent to or better than shorter dispensing intervals in other countries, the overall cost impact on national treatment programmes is uncertain. We estimated the costs and staffing impact of different 6MMD scale‐up scenarios over 2 years in South Africa.

**Methods**: We used the Thembisa 4.7 model to estimate the number of ART clients eligible for 6MMD in South Africa (on first‐line TLD treatment for >12 months, two consecutive suppressed viral loads and no comorbid conditions). We assessed three implementation scenarios:

1. 3‐month dispensing (on CCMDD) with 6‐month scripting and two annual facility visits (previous SOC);
2. facility‐based 6MMD with 6‐month scripting and two annual facility visits and medication pickups (facility 6MMD); and
3. community‐based 6MMD with 12‐month scripting, one annual facility visit and medication pickup, and one annual Central Chronic Medicines Dispensing and Distribution (CCMDD) programme medication pickup (CCMDD 6MMD).

Costs estimated were for ART procurement and service provision, including CCMDD fees and staff.

**Results**: The costs calculated are for approximately 1 million clients would be eligible for 6MMD initially in year 1. Scenario costs are shown in Table 1. Facility 6MMD requires higher upfront medication costs in the first year ($7.1 million additional compared to SOC) but would cost $13 million (72%) less than SOC over 2 years due to a reduction in service provider costs. CCMDD 6MMD would increase service provider fees compared to facility 6MMD $11 million, but staff time would be reduced annually from approximately 2.5 to 1.7 h (30%) for the one million clients.

**Conclusions**: Scaling up facility‐based 6MMD with 6‐month scripting could reduce overall ART programme costs in South Africa but would require earlier cash outlays for medication procurement. Policies enabling CCMDD‐based 6MMD with annual clinic visits would increase budgetary costs while substantially reducing staff workload and enhancing client‐centred service delivery, with important implications for workforce planning.

**TABLE 1:**
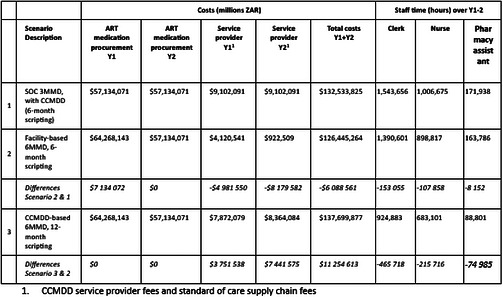
OAE0503 | Estimated costs and staff hours associated with 6MMD for different scenarios.

## OAE0504 MK‐8527: Mass Production for US $15/Year as PrEP

S. Cross ^1^, J. Levi^2^, F. Venter^3^, A. Hill^4^

^1^NHS Greater Glasgow and Clyde, Glasgow, United Kingdom; ^2^NHS Royal Free Hospital Trust, London, United Kingdom; ^3^University of Witwatersrand, Johannesburg, United Kingdom; ^4^University of Liverpool, Liverpool, United Kingdom

**Background**: Merck MK‐8527 is a nucleoside reverse transcriptase translocation inhibitor (NRTTI), similar in structure to islatravir. Its long half‐life supports monthly oral dosing as pre‐exposure prophylaxis (PrEP). The EXPRESSIVE 10 and 11 trials are evaluating MK‐8527 11 mg monthly, versus daily TDF/FTC as PrEP (results expected in 2027). Other antiretrovirals are mass produced at sustainable prices close to costs of manufacture in low‐ and middle‐income countries (LMICs), either due to expiration of patents or voluntary licenses (VLs). Current cost‐plus models have proven accuracy; lenacapavir was predicted and is subsequently set to be produced for $40 per person‐year (ppy). Primary and secondary “evergreening” patent applications for MK‐8527 are pending or already granted in LMICs, facilitating dictation of API cost by Merck.

**Methods**: The Trade Vision database was searched from 2020 to 2025 for records of MK‐8527 Active Pharmaceutical Ingredient (API) imported to India. The annual cost of MK‐8527 was calculated using cost+ modelling. The average costs/kg of API is multiplied by annual dosing and subsequently added to costs of formulation and 5% API loss, packaging, 15% transportation and labour, 30% profit margins and standard Indian tax rates (27%). For reference, cost of production was also calculated for islatravir.

**Results**: Two low‐volume API shipments imported into India between 2020 and 2025 yielded a weighted mean API price of US$69,239/kg. At the phase 3 dosing regimen of 11 mg/month (132 mg/person‐year), annual API cost is estimated at $9.14. After full cost+ analysis as described above, the estimated generic launch price for MK‐8527 PrEP is $15.43 ppy. Comparable estimates for islatravir range from $19.82 to 21.77 ppy, depending on dosing.

**Conclusions**: The NRTTI MK‐8527 could be mass produced for only $15 ppy. Low unit prices could allow highly cost‐effective mass distribution of MK‐8527 as PrEP in LMICs for significantly less than either oral generic TDF/FTC or long‐acting lenacapavir (both currently $40/year). Monthly MK‐8527 could also offer important delivery advantages over daily oral or injectable formulations, particularly in resource‐constrained settings. Access in LMICs will depend primarily on intellectual property and licensing decisions. Early negotiation of VLs will be essential to ensure timely and equitable access should phase 3 trials confirm efficacy and safety (see Figure 1).

**FIGURE 1:**
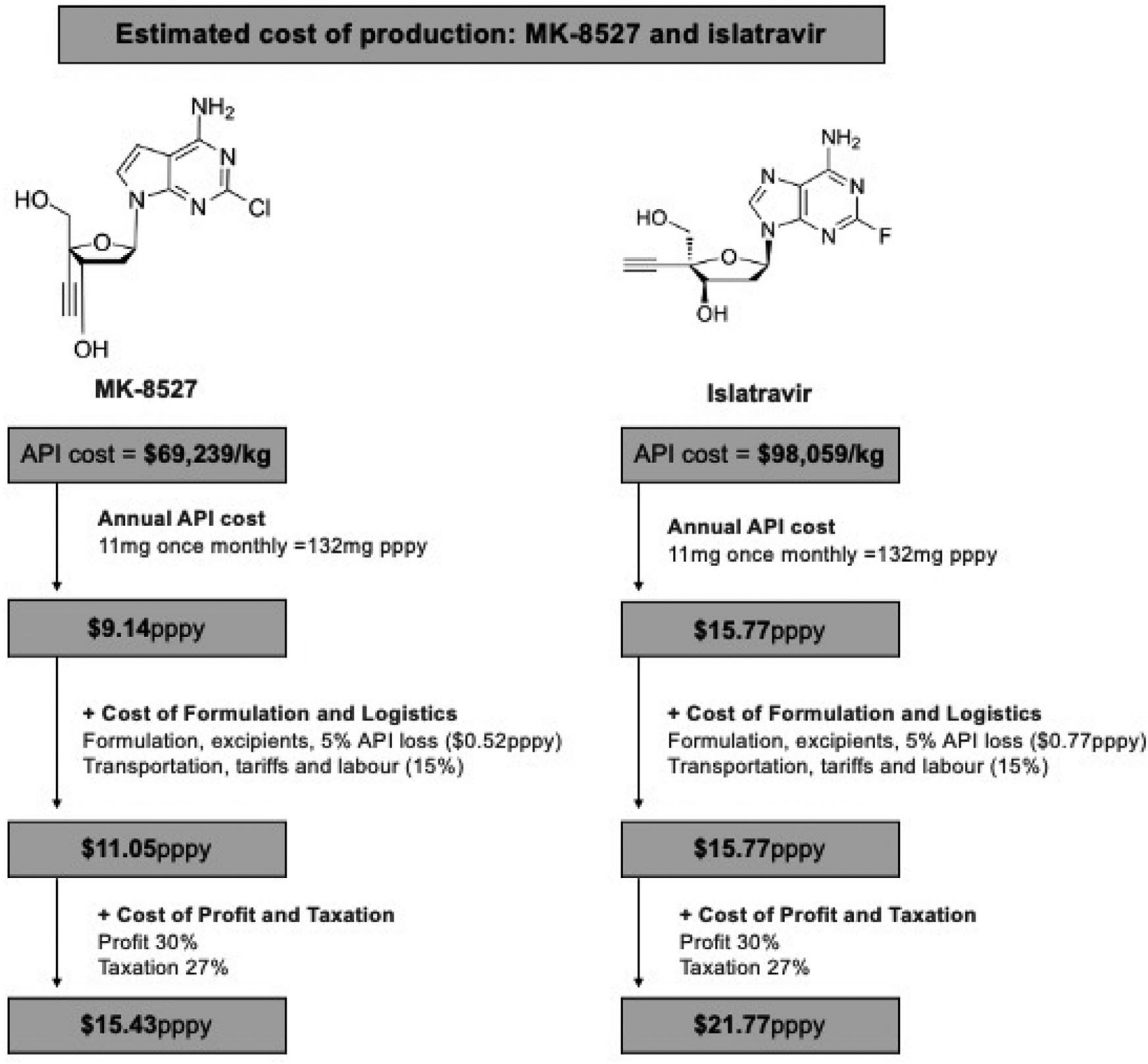
OAE0504

## OAE1602 Sustaining HIV Care and Support in Ukraine: A Model of Integrated, State‐Coordinated Community‐Based Services

L. Legkostup ^1^, L. Hetman^1^, N. Zapolska^1^, A. Bilets^1^, O. Gvozdetska^1^, P. Prytuliak^1^

^1^Public Health Center of the Ministry of Health of Ukraine, Kyiv, Ukraine

**Background**: To strengthen the sustainability and resilience of HIV programmes, Ukraine began transitioning non‐medical HIV services—including adherence counselling and treatment support—from donor‐funded pilots to national public financing in 2019. This transition aimed to institutionalize care and support (C&S) services for people living with HIV (PLHIV) as an integral part of the public health system. Although the full‐scale invasion in 2022 created broad humanitarian disruptions, the earlier shift to domestic financing enabled the country to maintain availability, integration and continuity of HIV services during the crisis.

**Description**: Ukraine's national HIV C&S programme is implemented by community‐based NGOs in partnership with health facilities across all 24 oblasts and Kyiv. Services are provided to PLHIV receiving or preparing for ART, including members of key populations and their partners. Standardized state‐funded interventions include motivational counselling, treatment literacy and partner navigation, with telehealth modalities applied in humanitarian settings. Service delivery follows national protocols grounded in voluntariness, confidentiality, peer involvement and person‐centred care. NGOs enter all service data into the national public health information system, which synchronizes with the national medical database, enabling seamless cascade tracking and integration.

**Lessons Learned**: Institutionalization of HIV C&S services in Ukraine has led to sustained ART retention. In 2024, 17,958 PLHIV newly enrolled in the national programme. By January 1, 2026, 17,093 of them remained on ART—representing 97.5% after accounting for 423 recorded deaths in 2025. Integration of NGO‐entered data with national systems enabled real‐time risk detection and proactive case management. Centralized coordination, community‐based partnerships and digital infrastructure were key enablers.

**Conclusions/Next Steps**: Ukraine's experience shows that institutionalized, state‐coordinated HIV care and support—embedded in national systems and co‐delivered with communities—can remain functional even during conflict. Rather than relying solely on donor funding, the model emphasized long‐term sustainability through national ownership, digital integration and service continuity. Future priorities include strengthening peer‐delivered psychosocial care, improving quality assurance and scaling up integrated support for underserved groups, particularly people who inject drugs. Ukraine's approach offers a transferable model for building resilient HIV systems in unstable global funding environments.

## OAE1603 Improving the HIV Care Cascade Among Venezuelan Migrants in Colombia Through a Rights‐Based Model, Community‐Based Testing and Assisted Partner Notification

J. R. Guillen Cañizares ^1,2,3^, M. A. Barriga Talero^4^, J. F. Ramirez Correa^5^, N. Reyes Galindo^6,7^, D. A. Collazos^8^, N. Santafe^9^, M. A. Gonzalez Cortes^1^, S. Castro Ramírez^1^, J. A. Ortega^10^

^1^Corporacion Red Somos, Bogotá, Colombia; ^2^Profamilia, Bogotá, Colombia; ^3^Universidad Monserrate, Bogotá, Colombia; ^4^Corporacion Red Somos, Executive Direction, Bogotá, Colombia; ^5^Corporacion Red Somos, Medical Unit Management, Bogotá, Colombia; ^6^Corporacion Red Somos, Projects, Bogotà, Colombia; ^7^Universidad El Bosque, Humanities, Bogotá, Colombia; ^8^Corporacion Red Somos, Planning and Monitoring, Bogotá, Colombia; ^9^Corporacion Red Somos, Projects, Bogotá, Colombia; ^10^Corporacion Red Somos, Coordination, Bogotá, Colombia

**Background**: The political, social and humanitarian crisis in Venezuela has led to the migration of millions of people, disrupting access to health services—including HIV prevention, diagnosis and treatment. Venezuelan migrants with HIV, living in Colombia, face barriers related to irregular migration status, stigma, poverty and exclusion from health insurance that impede timely diagnosis, initiation of antiretroviral therapy (ART), adherence and continuity of care in Colombian healthcare.

**Description**: Corporación Red Somos, a community‐based organization, between January‐2024 and December‐2025, established a rights‐based and peer‐led programme in Bogotá, Soacha/Cundinamarca and Barranquilla/Atlántico. The intervention comprised community‐based HIV testing‐assisted partner notification/index‐testing, peer navigation, psychosocial care, legal counselling and social work. Peer educators and community health workers provided education, counselling, service accompaniment, adherence support and facilitated psychosocial support groups. With assistance from public health providers and migration authorities, the programme targeted Venezuelan migrants newly diagnosed with HIV or re‐engaging in care, ensuring rapid linkage to ART with continuous clinical follow‐up, case management, migration regularization and enrolment in the Colombian health system.

**Lessons Learned**: In total, 7631 community‐based HIV tests were conducted with 503 people living with HIV positively diagnosed, with an overall positivity rate of 6.6%. Assisted partner notification was highly effective: 508 contacts were tested, producing 130 new HIV diagnoses and a positivity rate of 25.6%. With continuity of comprehensive care and ART through Red Somos, 96% of participants in the regularization and referral process received care, preventing treatment interruption. Peer‐led counselling and psychosocial support groups tackled stigma, emotional distress and migratory grief, enhancing adherence to treatment. An estimated 80% of participants gained regular migration status following integrated and peer‐led psychological, legal and social work support, and were enrolled in the national health system within 6 months. Of individuals who had viral load monitoring, 93.8% demonstrated documented viral suppression prior to transfer to the health‐system.

**Conclusions/Next Steps**: This community‐led and rights‐based model demonstrates that the integration of community HIV testing, assisted partner notification, ongoing clinical care, psychosocial support and migration regularization can result in early detection, high viral suppression and continuity of care for Venezuelan migrants with HIV, reducing structural barriers and respecting the right to health, especially in the context of forced migration and humanitarian crisis.

## OAE1604 Adapting HIV Service Delivery in Myanmar: Lessons Learned From Community‐Led Interventions During Public Health Emergencies

A. T. Oo, Myanmar HIV Implementation Research Network

Rotary Peace Center of Makerere University, Research and Education Planning, Kampala, Uganda

**Background**: This programme was initiated to address the severe disruption of HIV services for key populations in Myanmar—including female sex workers, men who have sex with men and people who inject drugs—during COVID‐19 and periods of political instability. Traditional facility‐based models became inaccessible or unsafe, threatening progress in HIV prevention, treatment and viral suppression. The main objectives were to safeguard treatment continuity, reduce HIV incidence and document new approaches to service delivery adaptable to emergency and unstable settings.

**Description**: From April 2022 to June 2025, the intervention operated across urban and peri‐urban zones of Yangon and Mandalay. The programme pivoted to differentiated ART distribution strategies, such as multi‐month dispensing, peer‐facilitated home delivery and mobile clinics for high‐risk zones. Digital platforms (SMS reminders, Telehealth, WhatsApp chatbots) were leveraged for appointment tracking, virtual adherence counselling and rapid symptom screening. Peer educators and community mobilizers received advanced training to coordinate outreach, provide psychosocial first aid, and facilitate linkage to both HIV and ancillary health services (TB, hepatitis, SRHR). Collaboration with grassroots organizations allowed ongoing feedback, localized risk communication and context‐sensitive adaptations, including support for mental health and domestic violence.

**Lessons Learned**: Despite ongoing crises, ART pick‐up rates improved by 35% (from 58% to 78%), viral suppression increased by 18% and fewer clients missed appointments (down 25%) compared to pre‐intervention periods. Direct involvement of peer networks broke down barriers to access, built trust, and improved disclosure and retention in care. Community feedback revealed high satisfaction with confidentiality and convenience. Implementation was hampered occasionally by supply chain shocks, digital divide issues and increased provider burnout; mitigation involved buffer stock planning, low‐tech outreach solutions and staff psychological support. The participatory, flexible management style allowed the team to anticipate and respond quickly to localized surges in need.

**Conclusions/Next Steps**: Community‐driven and technology‐enhanced service models ensured life‐saving HIV care during Myanmar's instability. The programme demonstrates that flexible delivery, real‐time feedback and local leadership enable rapid adaptation amid crisis. Future priorities include scaling these approaches regionally, integrating them with broader health and humanitarian responses, and advocating for their inclusion in national HIV policy to future‐proof HIV care and prevention efforts.

## OAE1605 Retention Breaks HIV Prevention: Sustaining Survivor Peer Educators for Trans Women Living With HIV in Nigeria's Safe Houses and IDP Camps

K. Kalu ^1,2^, J. Inyali^2^

^1^MIVA Open University, Public Health, Abuja, Nigeria; ^2^Supporting Trans Intersex and Gender Nonconforming People Initiative (STAG), Lagos, Nigeria

**Background**: Peer‐led HIV prevention often fails at the workforce point: survivor peer educators are recruited, trained, then lost. When peers exit, trust collapses, coverage becomes unstable and programmes restart the same cycle. This failure is sharper in safe houses and IDP camps, where trans women living with HIV face displacement, criminalization, violence and health‐system hostility. We mapped what drives recruitment, retention and sustainment of survivor peer educators delivering HIV prevention in these shelter settings in Nigeria.

**Methods**: We conducted an implementation science–focused qualitative study (2023–2025) across 10 sites (seven safe houses, three IDP locations) in six Nigerian states. We completed 65 key informant interviews and focus group discussions with 127 survivor peer educators (current and former), supervisors and programme stakeholders, and reviewed programme documents (selection criteria, role descriptions, supervision tools, training curricula and retention records). We used CFIR‐informed, framework‐guided thematic analysis, mapping determinants to implementation outcomes (acceptability, feasibility, appropriateness and early sustainment). Procedures were trauma‐informed, with confidentiality protections and referral pathways for distress.

**Results**: Recruitment was strongest where entry pathways were community‐endorsed (peer nomination, trusted gatekeepers), onboarding protected confidentiality and role expectations were explicit. Retention broke down when compensation was unpredictable, tasks expanded without role protection (“role creep”), safety risks were unmanaged and peers were treated as labour rather than decision‐makers. Emotional load and secondary trauma were persistent; the absence of structured debriefing accelerated burnout and exit. IDP contexts added specific attrition pressures: mobility restrictions, camp governance and gatekeeping, heightened exposure to harassment and fewer private spaces for outreach. 51% exited within or less than 2 months, and only 32% stayed up to or more than 6 months. Retention improved where programmes combined predictable compensation, supportive supervision, routine debriefing and psychosocial check‐ins, flexible scheduling aligned with safety realities, and peer leadership pathways (senior peer roles, paid coordination, skills‐building linked to livelihood options).

**Conclusions**: Sustaining survivor peer educators in shelter settings requires a protection‐and‐power package: fair pay, safeguarding, psychosocial support and real decision space. There should be set minimum peer‐support standards in contracts and track retention as a primary implementation outcome for durable HIV prevention in displaced and criminalized settings.

## OAE2602 Implementing Community‐Based Hepatitis C Screening for Key Populations in the Absence of Established Service Infrastructure: A Demonstration Study From Singapore

R. K. J. Tan^1,2^, F. Maurer‐Stroh ^2^, E. Libau^2^, E. J. Ong^1,2^, A. Tan^2^, R. Q. Yeo^2^, G. Pereira^2^, K. C. Yew^3^

^1^National University of Singapore, Saw Swee Hock School of Public Health, Singapore, Singapore; ^2^The Greenhouse Community Services Limited, Singapore, Singapore; ^3^Tan Tock Seng Hospital, Singapore, Singapore

**Background**: Hepatitis C virus (HCV) remains a significant cause of liver‐related morbidity among populations affected by HIV. Although highly effective curative therapies are available, gaps in testing persist, particularly among individuals exposed through sexualized substance use and sexual networks. Community‐based data among populations accessing HIV and sexual health services remain limited.

**Description**: In Singapore, HCV screening is not approved outside of healthcare institutions. This landmark demonstration study attempted to spur collaborations to introduce a community‐based care model. We implemented a community‐based sentinel HCV screening project using the RE‐AIM framework to assess feasibility, acceptability and public health value. The project was conducted through collaboration between a national foundation, community‐based organization and academic institution, with ethical approval under the Human Biomedical Research Act. Adults aged ≥21 years underwent oral‐fluid HCV antibody testing and completed an anonymous behavioural survey. A pilot phase (*n* = 25) informed protocol refinement, followed by community screening sessions in December 2025 (*n* = 41).

**Lessons Learned**: Reach: Participants (*n* = 41) reported multiple overlapping HCV risk exposures. Prior sexually transmitted infections were reported by 55%, including syphilis (67%), gonorrhoea (48%) and chlamydia (43%). Twenty‐four percent reported living with HIV. Sexual practices associated with mucosal trauma were reported by 24%, and 42% reported sex toy use; among these, 25% reported inconsistent or no sanitization. Sexualized substance use was common, including GHB/GBL (54%), methamphetamine (44%) and MDMA (38%). Lifetime injection drug use was reported by 11%.

Adoption: 64% of participants reported prior use of anonymous NGO‐based HIV testing services, indicating strong preference for community‐led testing models. Implementation: All tests completed successfully with no procedural failures following targeted staff training and quality assurance. Effectiveness: No reactive HCV antibody screens were identified. Maintenance: Community promotion across community partners reached over 50,000 individuals, and 13 community swabbers were accredited for future deployment, laying the groundwork for future scale‐up.

**Conclusions/Next Steps**: Using RE‐AIM as an organizing framework, this study demonstrates that community‐based HCV screening can reach populations engaged in HIV prevention and care who experience substantial sexual and substance‐related HCV risk. Integration of HCV screening into existing HIV and sexual health services, alongside targeted education on non‐injection transmission, may strengthen progress towards hepatitis C elimination goals.

## OAE2603 A Festival‐Based, CHW‐Led Testing and Navigation Model in Rural Appalachia: Reach, Screening Gaps and Linkage Outcomes at Healing Appalachia 2025

K. Keith ^1^, A. T. Young^1^, D. Lavender^2^, A. Reese^3^, L. Todd^2^, H. Arnold^1^, H. Viens^1^

^1^Community Education Group, Lost River, United States; ^2^Hope in the Hills, Huntington, United States; ^3^Harm Reduction Ohio, Columbus, United States

**Background**: Rural Appalachia experiences overlapping epidemics of substance use, viral hepatitis and HIV, compounded by transportation barriers, stigma and limited local service capacity. Recovery festivals may provide a practical platform for low‐threshold, community‐led testing with immediate navigation. We implemented and assessed a multi‐component model at Healing Appalachia 2025, combining community health workers (CHWs) crowd engagement, an on‐site prevention and testing booth, and structured linkage follow‐up.

**Description**: Community Education Group (CEG), in collaboration with Harm Reduction Ohio, deployed CHWs to conduct crowd‐based education and brief intercept surveys. We analysed three de‐identified data sources:

1. a testing and navigation log for all people screened for HIV and HCV (*n* = 141);
2. a CHW crowd survey capturing testing uptake that day and care‐navigation knowledge (cleaned analytic *n* = 256; testing item *n* = 225; care item *n* = 210); and
3. a booth visitor survey representing an engaged subset of attendees who self‐selected into deeper interaction (analytic *n* = 46).

**Lessons Learned**: Screening identified substantial HCV burden and high linkage: HIV positive 2/141 (1.4%) and HCV positive 16/141 (11.3%); confirmatory documentation was recorded for 2/2 HIV and 14/16 HCV positives; linkage to care was documented for 16/18 (88.9%) positives. In the crowd survey, 103/225 (45.8%) reported testing that day; among testers, most reported HIV and HCV co‐testing (74/103, 71.8%); a small subset reported HIV, HCV and syphilis testing (5/103, 4.9%). Testing uptake was highest among ages 18–24 (56.8%) and declined with age. Care‐navigation knowledge was high (193/210, 91.9%) and did not differ by age. In the engaged subset (booth survey), 27/46 (58.7%) reported never testing for HIV, HCV or syphilis despite high reported access indicators (regular provider 40/46, 87.0%; knowing where to seek treatment if positive 36/46, 78.3%). Prior testing differed by age and was strongly associated with injection history.

**Conclusions/Next Steps**: A CHW‐led festival model can generate high‐reach engagement, meaningful same‐day testing uptake and strong linkage outcomes, while also revealing a critical “connected‐to‐care but not screened” gap among engaged attendees. Next steps include strengthening confirmatory pathways, standardizing follow‐up protocols for open cases, and tailoring youth‐focused and first‐time tester messaging and navigation supports for future events.

## OAE2604 Integrating STI Self‐Testing as a Scalable Entry Point to HIV Prevention Among Adolescent Girls and Young Women in Dar es Salaam: Evidence From a Community‐Led Advocacy Initiative

E. J. Zahabu ^1^, L. B. Mwakyosi^2^, B. N. Jjuuko^3^

^1^WeCare Initiative, Programs, Dar es salaam, the United Republic of Tanzania; ^2^DARE for Progress, Dar es salaam, the United Republic of Tanzania; ^3^AVAC, New York, United States

**Background**: Adolescent girls and young women (AGYW) in Tanzania continue to bear a disproportionate burden of STIs and HIV, with untreated STIs increasing biological chances to HIV acquisition by up to three times. Despite this intersection, STI screening and HIV‐prevention uptake remain low due to stigma, fragmented services and limited youth‐friendly options. This community‐led project, implemented under the AVAC Advocacy Navigator programme, explored the potential of STI self‐testing as an innovative gateway into HIV testing and PrEP demand creation among AGYW.

**Description**: A multi‐component intervention (October–November 2025) combined facility mapping, community sensitization and pilot distribution of self‐testing kits. Facility assessments examined STI–HIV service integration, PrEP availability and adolescent readiness. Peer educators conducted targeted sessions with AGYW, caregivers and providers to increase STI literacy and emphasize STI–HIV risk linkages. A total of 250 syphilis self‐testing kits were distributed, complemented by onsite testing and structured referral pathways to youth‐friendly HIV services. Quantitative service uptake data and qualitative feedback from dialogues and facility visits informed the analysis.

**Lessons Learned**: STI self‐testing significantly increased AGYW engagement with HIV‐prevention services. Of the 250 distributed kits, 72% of AGYW who sought follow‐up care also completed HIV testing, and 41% received PrEP counselling through referral pathways. Facility mapping revealed systemic gaps, including limited integration of STI–HIV services, inconsistent condom availability and absence of PrEP in several mapped facilities. Community dialogues improved knowledge of STI–HIV interactions, reduced testing stigma, and strengthened trust between AGYW and providers. The intervention demonstrated that STI self‐testing not only improved early syphilis detection but also created an accessible, non‐judgemental entry point into HIV testing and prevention for AGYW.

**Conclusions/Next Steps**: Integrating STI self‐testing into community health strategies offers a promising, scalable pathway to increase HIV‐prevention uptake among AGYW. Strengthening service integration, reducing affordability barriers and expanding access to self‐testing could substantially reduce HIV vulnerability in urban Tanzanian settings. These findings position community‐led advocacy as a critical lever for transforming HIV‐prevention ecosystems for young women in Africa.

## OAE2605 Integrating HIV Testing, ART Initiation and Cervical Cancer Screening in Primary Care: Programmatic Results From Mozambique

S. V.d. C. Uamba Tualufo ^1^, N. Acubo^1^, M. Raivoso^1^, A. Couto^2^, O. Tiberi^3^, C. Mate^1^, C. Amado^1^

^1^Ministry of Health, NCD Department, Maputo, Mozambique; ^2^Ministry of Health, Public Health Directorate, Maputo, Mozambique; ^3^Ministry of Health, HIV Program, Maputo, Mozambique

**Background**: Integrating sexual and reproductive health services with HIV care is a key strategy to expand HIV diagnosis and timely linkage to antiretroviral therapy (ART) in high‐burden settings such as Mozambique. Cervical cancer screening services offer a unique, woman‐centred platform to identify previously undiagnosed HIV and facilitate ART initiation within routine primary care. We assessed HIV testing uptake, positivity and ART initiation among women attending cervical cancer screening services in Mozambique in 2025.

**Description**: We conducted a descriptive analysis of routine programmatic data from primary healthcare cervical cancer screening services in 2025. Indicators were analysed at national and provincial levels and included: total women attending screening consultations, HIV status at entry, HIV testing during the consultation, newly diagnosed HIV‐positive clients and ART initiation among women diagnosed with HIV.

**Lessons Learned**: In 2025, 1,441,901 women attended cervical cancer screening consultations. Of these, 503,394 (34.9%) had unknown HIV status at entry. Among women with unknown status, 471,780 (93.8%) were tested for HIV during the consultation, identifying 8568 (1.8%) newly diagnosed HIV‐positive cases. HIV testing uptake was highest in Gaza (99.6%), Sofala (96.9%) and Inhambane (96.0%). Overall, 17,923 women initiated ART during the screening consultation. Cervical cancer screening services contributed 2.8% of all national HIV‐positive diagnoses (8568/297,965) and 7.3% of all new ART initiations nationally (17,923/244,125). The higher number of ART initiations compared with newly diagnosed cases likely reflects limitations in routine programmatic data, including misclassification of HIV status and inconsistencies in ART reporting. Marked discrepancies were observed in Maputo City (1426.9%), Manica (466.0%) and Zambezia (324.9%).

**Conclusions/Next Steps**: Integrating HIV testing and ART initiation into cervical cancer screening services is feasible and contributes meaningfully to early HIV diagnosis and linkage to care in Mozambique. This integrated, one‐stop service delivery model strengthens the reach of HIV services while advancing women‐centred care. However, discrepancies observed in routine data highlight the need to strengthen health information systems to ensure accurate reporting of HIV status and treatment initiation. Improved data quality is essential to monitor programme performance and support progress towards both HIV epidemic control and cervical cancer prevention targets.

## OAE2902 First Evidence of Adherence, Safety and Effectiveness of Long‐Acting Injectable Cabotegravir for PrEP Among Sexually and Gender‐Diverse Adolescents in Brazil

I. Dourado ^1^, B. Oliveira Leite^1^, F. Soares^1^, P. Caires^1^, L. Dezanet^2^, M. Westin^2^, U. Tupinambás^2^, D. Greco^2^, P. Massa^3^, A. Grangeiro^3^, L. Magno^1,4,5^, On Behalf of the PrEP15‐19 Choices Study Group

^1^Universidade Federal da Bahia, Instituto de Saúde Coletiva, Salvador, Brazil; ^2^Universidade Federal de Minas Gerais, Faculdade de Medicina, Belo Horizonte, Brazil; ^3^Universidade de São Paulo, Faculdade de Medicina, São Paulo, Brazil; ^4^Universidade do Estado da Bahia, Departamento de Ciências da Vida, Salvador, Brazil; ^5^Fundação Oswaldo Cruz, Instituto Gonçalo Moniz, Salvador, Brazil

**Background**: Long‐acting injectable HIV PrEP (LAI‐PrEP) may be more suitable for sexually and gender‐diverse adolescents (SGDA) than oral regimens. This study evaluates the adherence, safety and effectiveness of LAI‐PrEP among SGDA in Brazil.

**Methods**: The PrEP15‐19 Choice study is a real‐world implementation cohort in three Brazilian cities. Participants included cisgender men who have sex with men (MSM), transgender/non‐binary people ages 15–19, enrolled between April 2024 and December 2025. Participants chose between LAI‐PrEP(cabotegravir) or oral PrEP(daily/event‐driven). Follow‐up for LAI‐PrEP was conducted at month 1 and every 2 months thereafter, while oral PrEP was conducted at month 1 and every 4 months. HIV testing utilized NAAT and fourth‐generation rapid tests. Adherence to LAI‐PrEP was defined by the proportion of days covered (PDC) (up to 37 days for the initial injection and 67 days for subsequent doses). Participants exceeding a 30‐day interval from their scheduled return required regimen reinstatement. Participants were also screened for STIs and monitored for adverse events. Analysis included descriptive and time‐dependent Cox proportional hazards models.

**Results**: Of 2303 adolescents reached, 643 (27.9%) initiated PrEP: 58.2% oral and 41.8% LAI‐PrEP. Most were aged 18–19 (74%) and identified as MSM (79%). In the LAI‐PrEP arm, 89.7% received >1 injection. The median PDC was 100% (IQR 96.7–100). Over 80% of participants received injections within scheduled windows, with no differences by age (Table 1). Regarding safety, the most common adverse event was Grade 1/2 injection site pain (35.4%), decreasing over time. No significant renal or hepatic deviations were noted. However, 25 suicidality events (20 in the LAI‐PrEP arm/5 in the oral arm) were recorded among 16 participants with previous mental health distress (75%). There were seven cases of HIV incidence. The incidence rate (IR) was 1.81 times higher in the oral PrEP arm (IR = 1.86/100 PY, *n* = 4) compared to the LAI‐PrEP arm (IR = 1.03/100 PY, *n* = 3) (Figure 1).

**Conclusions**: Real‐world data from PrEP15‐19 Choices demonstrate that high adherence to LAI‐PrEP is achievable among SGDA in Brazil. Although LAI‐PrEP appears more effective than oral PrEP in this cohort, the high prevalence of mental distress and suicidality events highlights the need for integrated mental health support within youth‐centred prevention models.

**TABLE 1:**
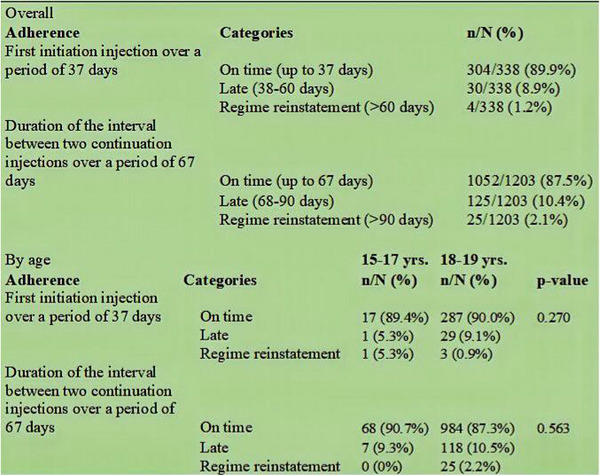
OAE2902 | LAI‐PrEP adherence overall and by age. PrEP15‐19 Choices Brazil Study.

**FIGURE 1:**
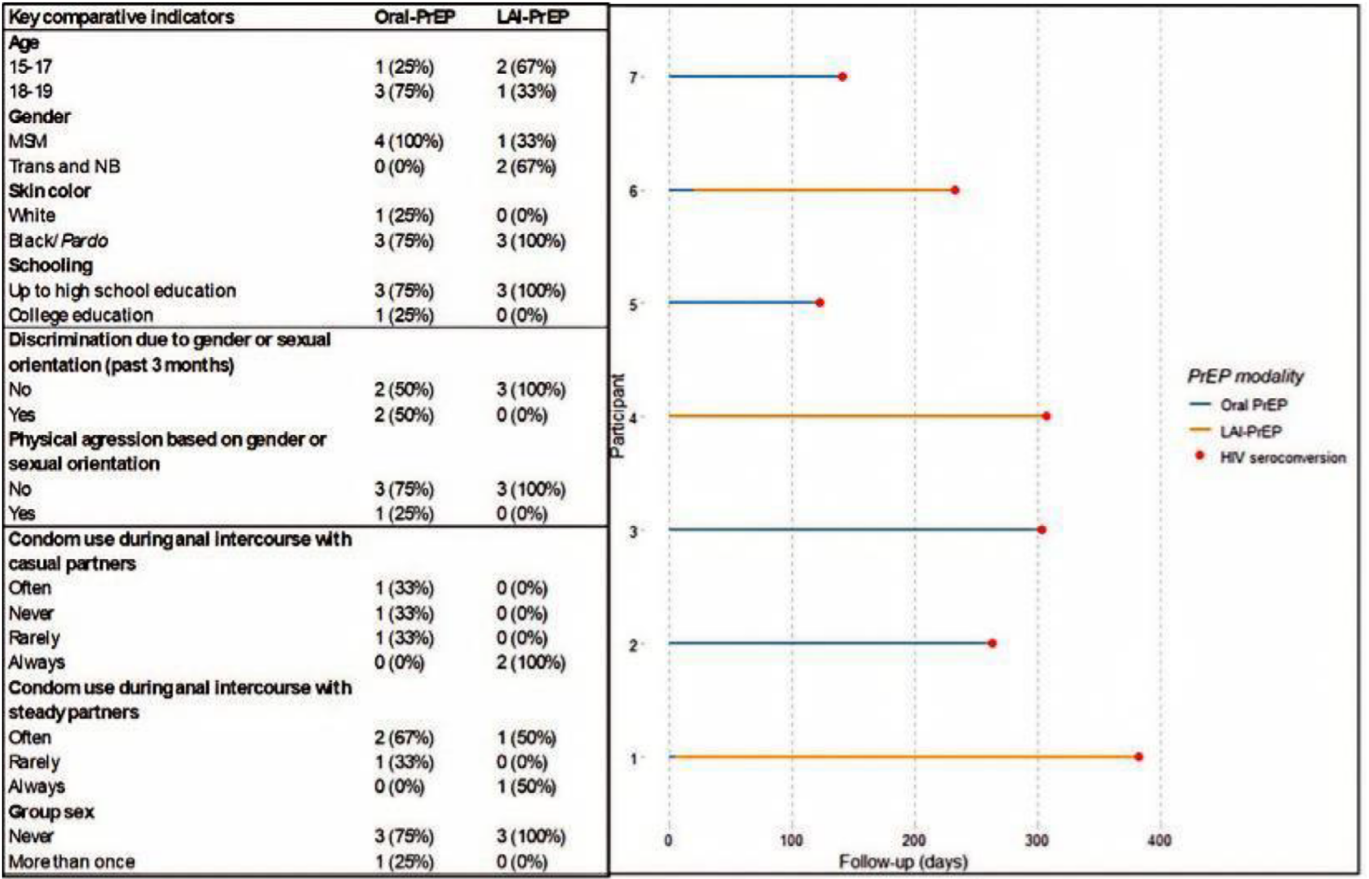
OAE2902 | HIV seroconversion within the PrEP15‐19 Choices Brazil Study.

## OAE2903 Leveraging Youth Champions to Enhance PrEP Uptake Among Clients Substantially Vulnerable to HIV at South Lunzu Health Center, Blantyre, Malawi: A Quality Improvement Initiative

G. Chisamba Baila ^1^, D. Kasale^2^, S. M. Allinder^3^, D. Hoege^4^, Y. Kamgwira^2^, C. B. Holmes^3^, G. Kawalazira^1^

^1^Blantyre District Health Office, Blantyre, Malawi; ^2^Malawi National AIDS Commission, Blantyre, Malawi; ^3^Center for Innovation in Global Health, Georgetown University, Washington, DC, United States; ^4^Center for Innovation in Global Health, Georgetown University, New York, United States

**Background**: Despite the availability of both oral and injectable pre‐exposure prophylaxis (PrEP), uptake at South Lunzu Health Center, a peri‐urban, public health facility in Blantyre, Malawi, that sees approximately 220 clients per week across four entry points, has remained low. A 2024 “community lab,” used in the district to gather insights from clients and communities, identified long waiting times as a key barrier to PrEP initiations and continuation. To address this, the facility's Quality Improvement Support Team (QIST) reoriented existing youth champions to accelerate PrEP service delivery.

**Description**: In June 2025, four youth champions, already supporting adolescents and youth living with HIV (AYLHIV) through the “Red Carpet Program,” were trained on PrEP services and health education and counselling, and re‐deployed to support potential PrEP clients. By escorting them between service points, linking them to the sexually transmitted infections (STIs) room, HIV testing services (HTS) and the PrEP room, the aim was to ensure faster facility navigation, reduced waiting times and enhanced client experience. The champions’ role also included providing tailored health education and counselling to improve understanding of PrEP and strengthen linkage to care.

**Lessons Learned**: Following implementation, PrEP uptake increased markedly. In Q1 2025, 48 clients initiated PrEP (oral: 41; injectable: 7). In Q4 2025, this number rose to 193 (oral: 81, injectable: 112), representing a substantial improvement compared to the pre‐intervention period. Youth champions also expanded their support to other HIV prevention services, including voluntary male medical circumcision (VMMC) and condom distribution. Continuous health education helped clients differentiate PrEP from ART, reducing stigma and strengthening confidence in PrEP use.

**Conclusions/Next Steps**: The integration of youth champions into PrEP service delivery can substantially increase uptake among adolescents and young people at risk of HIV acquisition. Equipping youth champions with PrEP knowledge enables them to provide accurate information, support informed decision‐making and strengthen overall HIV prevention efforts (see Figure 1).

**FIGURE 1:**
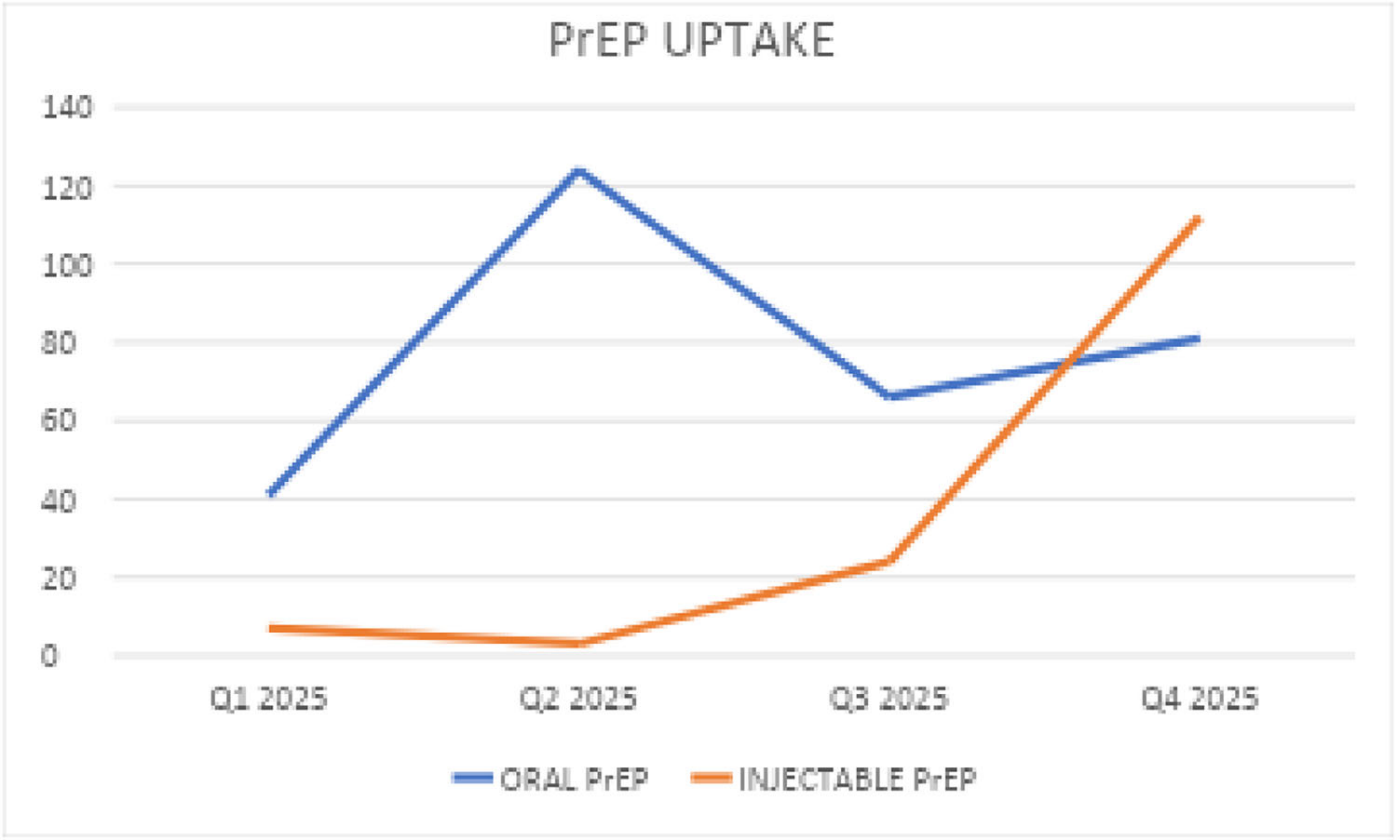
OAE2903

## OAE2904 Empowering Gen Z: Peer‐Led Digital Campaign for HIV Prevention and Mental Health Among Thai Youth

N. Manojai ^1^, A. Pechsamrit^1^, N. Wajasuwan^1^, N. Taddee^2^, T. Srimuang^3^, C. Hempongchanachai^4^, R. Apiputthipan^5^, P. Patpeerapong^5^

^1^MPLUS Foundation, Chiangmai, Thailand; ^2^MPLUS Foundation, Phitsanulok, Thailand; ^3^MPLUS Foundation, Nakhon Ratchasima, Thailand; ^4^MPLUS Foundation, Lampang, Thailand; ^5^MPLUS Foundation, Chiangrai, Thailand

**Background**: Thai youth (ages 15−24) have experienced an increase in HIV rates as well as increasing issues with mental health; however, traditional healthcare systems often remain inaccessible to many youth due to the stigma associated with these issues as well as the lack of integration between physical/sexual health and mental health. To meet this need, the MPLUS Foundation has developed the “YES! Young Program,” a youth‐designed and youth‐led project that includes three main components: sexual health, mental health and gender based‐violence (GBV) prevention.

**Description**: In Chiang Mai (2024−2025), the YES! Young Program used a co‐design approach where young people (GenZ) who would lead the project. Youth were able to use social media platforms such as TikTok, Instagram, Facebook and YouTube to access the programme without fear of stigma. The programme partnered with seven universities and 12 schools, providing both online outreach and community‐based programmes which included; HIV testing, mental health screening and direct referral to clinical providers that provided youth‐friendly care.

**Lessons Learned**: The YES! Young Program reached 26,386 youth through online HIV and mental health screenings during the period of October 2024−September 2025. Out of the 26,386 youth that were screened for HIV, 181 were identified with HIV and 100% youth received antiretroviral therapy (ART) treatment. These results support the idea that using digital first engagement methods can lower barriers to testing and increase levels of trust among youth. Additionally, including mental health assessments into the HIV testing process increases acceptance of services and empower youth to focus on their overall wellness.

**Conclusions/Next Steps**: The YES! Young program provides evidence that peer‐led, digitally enhanced models can be very successful in engaging at risk youth populations. This programme also supports Thailand's goals to eliminate AIDS by 2030, demonstrating that youth‐driven innovations are a viable method for creating sustainable and scalable frameworks.

## OAE2905 Integrating U = U Into HIV Care for Adolescents and Young Adults in Cape Town, South Africa: A Randomized Trial

T. Sineke ^1^, J. Bor^2^, M. Dukashe^1^, R. King^3^, R. A. Ruiter^4^, D. Onoya^1^

^1^University of the Witwatersrand, Department of Internal Medicine, School of Medicine, Johannesburg, South Africa; ^2^Boston University, Department of Global Health, School of Public Health, Boston, United States; ^3^University of California, Institute for Global Health Sciences, California, United States; ^4^Maastricht University, Department of Work and Social Psychology, Maastricht, the Netherlands

**Background**: South Africa's adolescents and young adults (AYA, 15−24 years) face disproportionately high HIV incidence and suboptimal retention in care and viral suppression, undermining progress towards the UNAIDS 95–95–95 targets. The Undetectable Equals Untransmissible (U = U) message, grounded in evidence that viral suppression eliminates HIV transmission, offers a promising approach to reduce stigma, motivate adherence and strengthen engagement in care among AYA. We evaluated the impact of the Undetectable and You mobile application, which we previously developed, on knowledge and attitudes related to the prevention benefits of ART.

**Description**: The intervention was implemented between May and August 2025 at a public‐sector youth HIV clinic in Cape Town as a randomized controlled trial embedded in routine care. It enrolled 150 adolescents and young adults aged 18–24 years living with HIV and evaluated the Undetectable & You intervention integrating U = U messaging. The intervention aimed to improve U = U knowledge, reduce stigma, support disclosure and promote positive attitudes towards ART and sexual wellbeing, while assessing feasibility, acceptability and potential to strengthen engagement in care among young people living with HIV.

**Lessons Learned**: The intervention showed that a brief, U = U intervention can improve HIV‐related knowledge, attitudes and psychosocial outcomes among adolescents and young adults living with HIV. Baseline awareness of U = U was low (24.7%), with widespread misconceptions about transmission on ART. Following exposure to the Undetectable & You App, U = U knowledge improved markedly, with 78% of intervention participants correctly identifying zero transmission risk when virally suppressed compared to 34.2% of controls, and larger gains among those with no prior U = U exposure. The intervention was also associated with more positive attitudes, including greater comfort with intimacy and improved sexual wellbeing, suggesting reduced fear and internalized stigma. Acceptability was high, with over 95% of participants reporting the App as easy to use and useful, demonstrating feasibility of integrating brief digital U = U tools into routine HIV care.

**Conclusions/Next Steps**: The Undetectable and You App significantly increased U = U knowledge and improved perceived sexual wellbeing among AYA. By reducing stigma‐related concerns and correcting misconceptions, this digital U = U intervention could improve ART adherence and viral suppression among AYA in high‐burden settings.

## OAE3102 Real‐World Implementation of Long‐Acting Lenacapavir for HIV PrEP in Zambia: Early Programmatic Outcomes From a Phased Rollout

L. B. Mulenga ^1,2,3^, C. Phiri^1,2^, D. Kampamba^1,2^, M. Siame^1^, K. Zyambo^1,2^, S. Sivile^1,2,3^, I. Bwalya^1,2^, D. Engamba^2^, M. Mwitumwa^2^, A. Bhebhe‐Moyo^2^, M. Mutinta‐Mbewe^2^, G. Ngoma^2^, N. Namusukuma^2^, M. Kalima^2^, A. Ndhlovu^2^, L. Kampilimba‐Mwango^4^, M. Phiri^5^, P. Banda^2^, M. Musosha^5^, R. Nzoolo^6^, G. Muyembe^6^, C. Baumhart^7,8^, O. Chilyabanyama^4^, D. Daka^1^, M. Mulenga^9^, H. Phiri^1^, D. Chanda^2,3^, C. Kalombo^1^, M. Muvwimi^1^, M. Kasonde^1^, G. Sinyangwe^1^, L. Chitembo^10^, K. Lishimpi^1^, C. W. Claassen^7,8^

^1^Ministry of Health, Lusaka, Zambia; ^2^University Teaching Hospital, Lusaka, Zambia; ^3^University of Zambia, School of Medicine, Division of Infectious Diseases, Lusaka, Zambia; ^4^Ciheb Zambia, Lusaka, Zambia; ^5^Lusaka Provincial Health Office, Lusaka, Zambia; ^6^Chawama General Hospital, Lusaka, Zambia; ^7^Center for International Health, Education, and Biosecurity, University of Maryland, School of Medicine, Baltimore, United States; ^8^Institute of Human Virology, University of Maryland, School of Medicine, Baltimore, United States; ^9^Clinton Health Access Initiative, Lusaka, Zambia; ^10^World Health Ogranisation, Lusaka, Zambia

**Background**: Long‐acting HIV prevention products offer an opportunity to overcome persistent barriers to daily oral PrEP, particularly for women, adolescents and young people (AYP), and other high risk sub‐populations. However, real‐world evidence on uptake, population reach and delivery models for lenacapavir (LEN) for HIV pre‐exposure prophylaxis remains limited. Zambia initiated a phased rollout of LEN for HIV prevention. We describe early LEN implementation outcomes in Zambia to inform national scale‐up using 2 months of real‐world experience.

**Methods**: We conducted a descriptive programmatic analysis of routine service delivery data from a phased rollout of lenacapavir for PrEP at three public‐sector facilities in Zambia. Data were collected during the first 8 weeks of implementation (December 2025–January 2026). Variables included client age, sex, pregnancy or breastfeeding status, PrEP history and population type (general population vs. key and priority populations). Uptake trends and client characteristics were summarized using descriptive statistics. Integration within routine and maternal health service platforms was assessed through documentation of service entry points.

**Results**: Between 1 December 2025 and 22 January 2026, a total of 445 clients were initiated on long‐acting injectable LEN for PrEP across three implementation sites. Overall uptake averaged 8.4 initiations per day, with a strong launch week followed by sustained weekly initiations in December and a decline in January due to limited LEN stock. Majority of clients were female (60%), with mean age of 33.3 years (range: 16–59). AYP aged 15–24 years accounted for 14.8% (*n* = 66) of initiations, including 56 adolescent girls and young women. Notably, 71% of clients were PrEP‐naïve. Pregnant and breastfeeding women comprised 30.9% of female initiators (*n* = 82). Key populations represented 22% of all initiations. Additionally, 29% of clients switched to LEN from other PrEP modalities, with gender differences observed by prior method.

**Conclusions**: Zambia successfully launched LEN for PrEP and early data shows outreach to both general population and key and priority populations. High uptake among PrEP naïve groups indicates reach of populations not previously reached by existing PrEP modalities. Early integration into maternal health platforms demonstrates feasibility for differentiated service delivery models. Six‐month data will inform PrEP persistence and future directions.

## OAE3103 Early Drivers of Lenacapavir (LEN) PrEP Uptake and Switching Patterns Across Five Health Facilities in Eswatini, 1 December 2025–23 January 2026: Evidence From the First 8 Weeks of Rollout

M. Mkhontfo ^1^, A. Dlamini^1^, W. Simelane^2^, L. Mpango^1^, S. Matse^3^

^1^Jhpiego, Clinical, Mbabane, Eswatini; ^2^Jhpiego, Strategic Information, Mbabane, Eswatini; ^3^Ministry of Health, Eswatini National AIDS Programme, Mbabane, Eswatini

**Background**: Long‐acting lenacapavir (LEN) offers twice‐yearly dosing and may address adherence barriers seen with daily oral pre‐exposure prophylaxis (PrEP). As Eswatini integrates LEN into routine prevention, early insights on who initiates versus who switch from other PrEP modalities can guide scale‐up.

**Methods**: We conducted a retrospective analysis of LEN implementation programme data from five facilities covering 1 December 2025–23 January 2026. Aggregated records were reshaped to distinguish between new LEN initiations and switching from other PrEP methods. Descriptive statistics summarized distributions by sex, age, region, location and client volume. Pearson χ^2^ tests with Cramér's V statistical analysis were used to estimate associations between uptake type and covariates. Pregnancy/breastfeeding (PBFW) counts were summarized separately.

**Results**: Among 415 LEN clients, 228 (54.9%) were new initiations and 187 (45.1%) switches. New initiations were predominantly female (155; 68%) versus male (73; 32%). LEN initiations peaked at 25–34 years (*n* = 86), followed by ≥35 years (*n* = 75) and 16–24 years (*n* = 67). Urban and rural initiations were balanced (Urban 121; Rural 107) but switching concentrated in urban settings (143 vs. 44) (χ^2^ = 23.30, *p* < 0.001; *V* = 0.24). High‐volume facilities accounted for 155 new and 166 switch clients, versus 73 new and 21 switch in low‐volume facilities (χ^2^ = 24.17, *p* < 0.001; *V* = 0.24). New initiations were linear, while switching clustered in Manzini region (117) (χ^2^ = 37.48, *p* < 0.001; *V* = 0.30). High‐volume sites recorded the highest numbers (83 new; 117 switches; 58.5% switch share), whereas rural, low‐volume, showed strong initiation (43 new) with limited switching (7). Among PBFW, 22 new and 20 switch clients were recorded.

**Conclusions**: These findings reflect Eswatini's prevention landscape, where high‐volume urban facilities host mature PrEP cohorts likely to transition to LEN for convenience and reduced visit burden. In contrast, new initiations were broadly distributed, with strong uptake among women, consistent with Eswatini's HIV epidemiology. Rural clinics demonstrated substantial initiation demand, suggesting LEN may overcome historical adherence barriers. Age and sex‐specific patterns highlight distinct prevention needs, while early PBFW uptake signals growing acceptability in maternal populations. Tailoring service delivery, and providing age‐ and gender‐responsive counselling, will be critical as Eswatini scales LEN nationally.

## OAE3104 Boosting Youth Uptake of HIV Self‐Testing and Pre‐Exposure Prophylaxis Via Peer‐Led Alternative Access Points in Greater Metro Manila, Philippines

M. R. Cabuso ^1^, E. Bagasol Jr.^1^, P. Lopez^2^, D. A. Ching^2^, A. J. Villapando^3^, M. Sumagaysay^3^

^1^Family Health International 360, Program, Makati City, the Philippines; ^2^HIV & AIDS Support House (HASH), Quezon City, the Philippines; ^3^Free To Be YOUTH, Quezon City, the Philippines

**Background**: Despite wide availability, HIV self‐testing (HIVST) and oral PrEP uptake among youth remain low in Greater Metro Manila, Philippines. Data in 2025 from the 51 U.S. Government‐funded Meeting Targets and Maintaining Epidemic Control (EpiC)‐supported sites showed that 38% of allocated HIVST kits were used. Only 7% of people who received negative results through EpiC‐supported testing enrolled on PrEP. Youth‐focused community partners attributed these challenges to time and cost to travel to service sites, courier fees for home‐delivered services, and hesitancy to use HIVST and PrEP. We introduced youth‐led alternative access points (“ALTERS”) to reduce access barriers and increase uptake.

**Description**: Youth‐led network Free To Be YOUTH and community‐led HIV services group HIV & AIDS Support House co‐developed the strategy. From November to December 2025, 10 highly networked youth from low‐uptake areas were trained to (1) recruit peers through dating/social media apps, (2) provide professional virtual client support and (3) coordinate confidential delivery. Using a standard protocol, ALTERS offered access options (delivery/pick‐up/meet‐up) and HIVST support options (independent testing/in‐person guidance/virtual guidance). We used ChatGPT to produce tailored social media posts; and monitored service requests, fulfilment and service uptake.

**Lessons Learned**: ALTERS received 189 HIVST unique requests with 46% preferring home delivery, 37% meet‐ups and 17% pick‐up points. Among 155 clients reached, 17% accessed PrEP, exceeding the 7% PrEP enrolment observed in facility‐based services. Most clients reached preferred independent self‐testing (51%); 38% reported testing for the first time. On average, delivery took 4 days. Conversion—the percentage of people who viewed the content or received a peer message and submitted an HIVST request—was highest through ALTERS peer networks (40%), followed by AI‐generated Facebook content (39%). X and dating app posts produced lower conversion (average 21%).

**Conclusions/Next Steps**: Youth‐led, multi‐channel delivery can provide discreet HIVST and PrEP access for young people and may help supplement testing in low‐uptake areas, including increasing linkage to PrEP among youth. Delivery time and data encoding support can be improved by increasing peers and ALTERS. Future initiatives should evaluate social media application integration and AI‐assisted targeting to improve reach and linkage to services.

## OAE3105 Patterns of PrEP and PEP Use Through Automated Dispensing Machines in São Paulo, Brazil: Modality, Motivations and Gender Differences

M. C. Abbate^1^, J. Araújo de Oliveira Silva^2^, L. T. Queiroga de Souza^2^, R. Fernandes de Camargo^3^, M. A. Barbosa^4^, A. Queiroz da Silva ^5^, C. M. Matos^6^

^1^São Paulo City Health Department, Municipal HIV/STI Coordination, São Paulo, Brazil; ^2^São Paulo City Health Department, Municipal HIV/STI (Research and Development Coordination), São Paulo, Brazil; ^3^São Paulo City Health Department, Municipal HIV/STI Clinical Area, São Paulo, Brazil; ^4^São Paulo City Health Department, Municipal HIV/STI Information, São Paulo, Brazil; ^5^São Paulo City Health Department, Municipal HIV/STI Prevention Area, São Paulo, Brazil; ^6^São Paulo City Health Department, Municipal HIV/STI Diagnosis, São Paulo, Brazil

**Background**: Innovative service delivery strategies are essential to expand timely access to HIV prevention, particularly for populations facing stigma and structural barriers. In São Paulo, Brazil, automated dispensing machines have been implemented to provide PrEP, PEP and HIV self‐tests within the public health system. This study examines patterns of use, modalities and motivations for accessing these machines, with attention to gender differences.

**Methods**: This cross‐sectional study analysed data from an online, self‐administered questionnaire completed by individuals aged 18 years or older who accessed automated dispensing machines located in metro stations in São Paulo. The machines provide PrEP, PEP and HIV self‐tests. Sociodemographic characteristics, modality of PrEP use (daily or on‐demand), and reasons for accessing and using PrEP or PEP were analysed descriptively using valid responses only. Categorical variables were summarized as frequencies and percentages. The study was approved by the Research Ethics Committee (CAAE 85263124.2.0000.0086).

**Results**: A total of 1200 individuals participated, with varying completeness across variables. Among respondents who provided gender information (*n* = 487), most identified as cisgender (91.8%), with a smaller proportion identifying as non‐binary, transgender, gender‐fluid or agender. Regarding modality of use (*n* = 420), daily PrEP was the most common strategy (63.6%), followed by on‐demand PrEP (29.3%). Access to the machines was primarily motivated by PrEP use (77.3%), while 21.6% reported accessing PEP. Among respondents reporting reasons for PrEP or PEP use (*n* = 483), continuous prevention was the most frequently cited motivation (72.5%), followed by recent exposure to HIV (23.2%). Patterns of modality and motivation were broadly consistent across gender groups, with daily PrEP and continuous prevention predominating among all genders.

**Conclusions**: Automated dispensing machines primarily support sustained HIV prevention through daily PrEP use, while also facilitating timely access to PEP following recent exposure. The predominance of continuous prevention across gender identities highlights the role of these technologies in supporting ongoing engagement with HIV prevention strategies. These findings underscore the potential of automated delivery models to strengthen combination prevention within public health systems and to promote equitable access to PrEP and PEP (see Figures 1 and 2).

**FIGURE 1:**
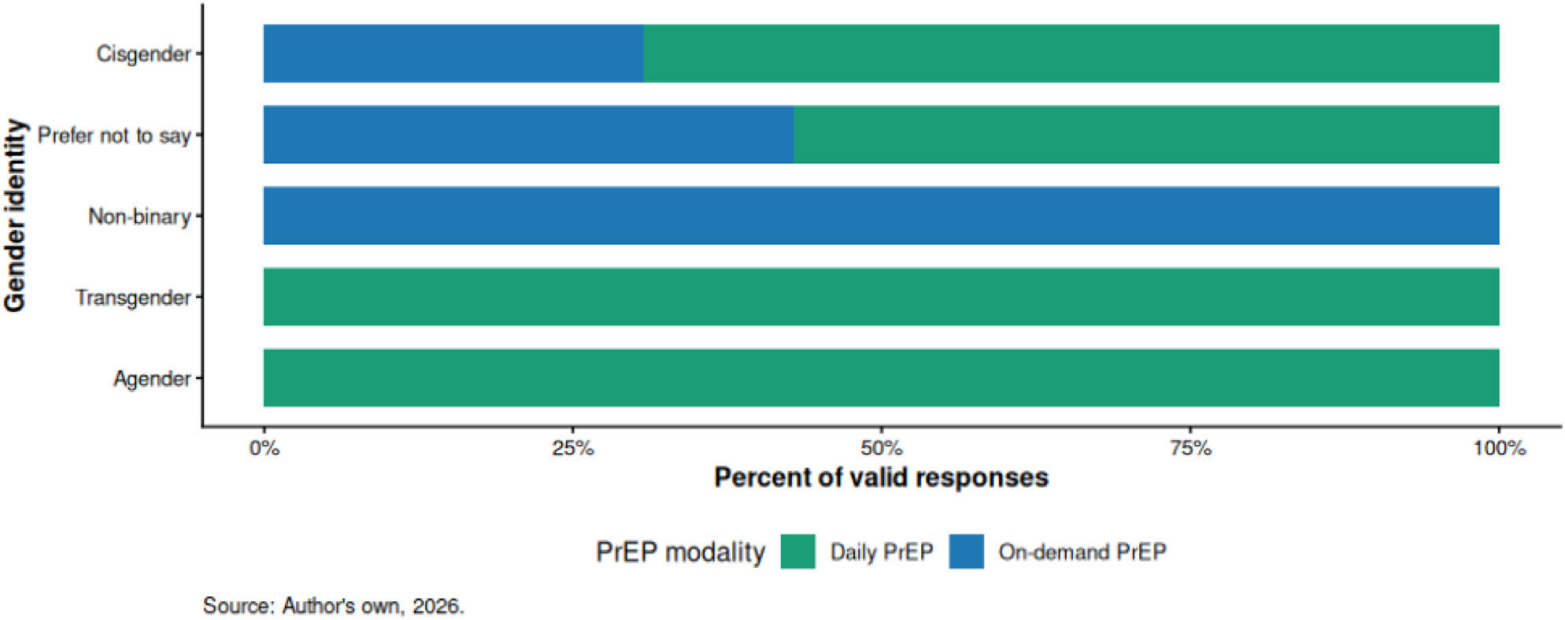
OAE3105 | PrEP modality by gender identity.

**FIGURE 2:**
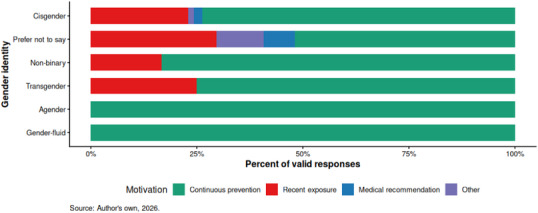
OAE3105 | Motivations for PrEP and PEP use by gender identity.

## OAE3702 Integrated Community‐Based Care Versus Facility‐Based Integrated Care for People Living With HIV, Diabetes or Hypertension in Sub‐Saharan Africa (INTE‐COMM): A Multi‐Country Cluster‐Randomized Trial

F. X. Kasujja ^1,2^, F. Aikaeli^3,4^, A. Garrib^5,6^, I. Namakoola^1^, E. van Widenfelt^5^, J. Birungi^1,7,8^, S. Kivuyo^3,9^, S. Mfinanga^3^, K. Ramaiya^10,11^, M. Nyirenda^1^, S. Jaffar^5^

^1^MRC/UVRI & LSHTM Uganda Research Unit, NCD Theme, Entebbe, Uganda; ^2^Uppsala University, Global Health and Migration Unit, Department of Women's and Children's Health, International Maternal and Child Health, Uppsala, Sweden; ^3^National Institutes for Medical Research, Dar es Salaam, the United Republic of Tanzania; ^4^KCMC University, Department of Public Health, Kilimanjaro, the United Republic of Tanzania; ^5^University College London, Institute for Global Health, London, United Kingdom; ^6^Liverpool School of Tropical Medicine, Department of Clinical Sciences, Liverpool, United Kingdom; ^7^The AIDS Support Organisation, Mulago Hospital Complex, Kampala, Uganda; ^8^La Trobe University, School of Psychology and Public Health, Melbourne, Australia; ^9^University of Barcelona, Barcelona Institute for Global Health Hospital Clinic, Barcelona, Spain; ^10^Tanzania NCDs Alliance, Dar es Salaam, the United Republic of Tanzania; ^11^Shree Hindu Mandal Hospital, Research and Training Unit, Dar es Salaam, the United Republic of Tanzania

**Background**: In sub‐Saharan Africa, the burden of diabetes and hypertension is now high, alongside a continuing high prevalence of HIV. We compared community‐based with facility‐based integrated management of HIV, diabetes and hypertension.

**Methods**: We conducted a cluster‐randomized trial in Tanzania and Uganda. Adults living with HIV, diabetes or hypertension were grouped into clusters of 8−14 participants based on residence. Clusters were randomized to provide either integrated facility‐based or integrated community‐based care by the same healthcare team at a single visit irrespective of the participant's condition. In the facility arm, participants shared registration, waiting areas, healthcare workers, pharmacy and laboratory services. In the community arm, a nurse and a trained lay worker met the group at focal points in the community to administer their treatment. Participant follow‐up was 12 months with two co‐primary endpoints: disease control (blood pressure <140/90 mmHg for hypertension, fasting glucose <7.0 mmol/L for diabetes or both) and plasma HIV viral load suppression (<1000 copies/mL). Statistical analyses were adjusted for clustering using generalized linear models. The trial is registered (ISRCTN15319595).

**Results**: Between 30th January and 6th October 2023, 1864 participants were recruited into 124 clusters. 62 clusters were randomized to each of the community‐based and facility‐based integrated care models. Baseline characteristics were well balanced: 75.6% females versus 24.4% males in the community and 78.7% females versus 22.7% males in the facility arm. Among those with hypertension and/or diabetes, the proportion whose condition was controlled did not differ significantly between the two arms: 317/574 (55.2%) in the community arm and 304/571 (53.2%) in the facility arm (adjusted risk difference = 1.80% [95% CI: –4.52%, 8.12%] *p* = 0.58). Among people living with HIV alone, 227/229 (99.1%) in the community arm and 229/232 (98.7%) in the facility arm had HIV viral suppression (adjusted risk difference = 0.44% [95% CI –1.12%, 1.99%], *p*‐value for non‐inferiority <0.0001). Participants with HIV and hypertension and/or diabetes in the community arm had a lower mean diastolic BP (adjusted mean difference = –8.37 [95% CI –14.71, –2.02], *p* = 0.0098).

**Conclusions**: In sub‐Saharan Africa, integrated community‐based chronic care services could achieve a high standard of diabetes and hypertension care without undermining HIV outcomes.

## OAE3703 Community‐Led One‐Stop‐Shop for Integrated Communicable Disease and NCD Diagnostics and Monitoring: Digital Case Management for Key Populations in Moldova

R. Poverga, C. Cearanovski, A. Cojocari

Positive Initiative, CSO, Chisinau, the Republic of Moldova

**Background**: Key affected populations and people living with HIV (PLHIV), despite formal primary care, often face limited access to diagnostics and basic services due to stigma and discrimination, fear of punishment/criminal prosecution, and geographically fragmented HIV, TB, STI and addiction services for >60,000 people. This creates major barriers to comprehensive diagnosis and follow‐up. Overlapping needs add complexity: >15% belong to multiple key populations and require specific care (TB, opioid agonist therapy, STIs), while >20% show abnormal primary health indicators linked to non‐communicable conditions (mental health, cardiovascular risk, blood glucose), disability and vision/mobility limitations. Vulnerability increased further during COVID‐19 and is compounded by economic insecurity and gender‐related factors.

**Description**: A community‐led, licensed medico‐social centre is piloting a trusted one‐stop‐shop model (online and offline) for integrated diagnostics and monitoring, followed by referral, accompaniment and follow‐up. The package includes capillary‐blood rapid tests for HIV, syphilis and viral hepatitis; TB symptom screening with referral for confirmatory testing; mental health screening; stigma/discrimination screening; a social vulnerability questionnaire; and basic clinical measurements (blood pressure, temperature, oxygen saturation, ECG, blood glucose, lung volume and weight). Data are recorded in an electronic system linked to a unique client code/card, enabling rapid capture of clinical and social signals and continuity across providers. The ecosystem connects 16 service providers and includes >50,000 client profiles and >1.5 million registered services/goods. Follow‐up uses reminders, teleconsultations and care navigation to reduce loss after initial testing. A mobile app supports secure communication, request logging/routing and task monitoring between visits.

**Lessons Learned**: Integrated communicable + NCD screening with social determinants assessment is operationally valuable because intersections drive pathways and disengagement risk. Digital registration and electronic case management strengthen control through standardized data, role‐based tasking and completion monitoring. Mobile‐app follow‐up reduces barriers and accelerates linkage to needed medical and social support.

**Conclusions/Next Steps**: This one‐stop‐shop model with digital case management can shift support from fragmented, reactive care to integrated, data‐informed pathways. Next steps include evaluating implementation outcomes (reach, diagnostic completion, referral timeliness, task completion and outcomes of confirmatory testing/treatment) and preparing a scale‐up package (indicator sets and data‐governance/confidentiality templates) (see Figure 1).

**FIGURE 1:**
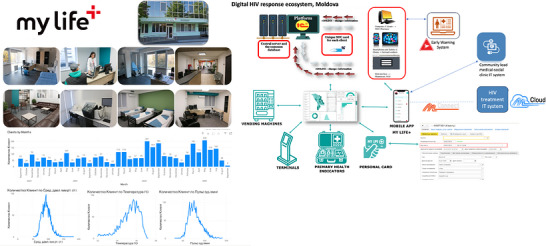
OAE3703

## OAE3704 Hearing the Voice of the People: Impact of Integration of Hypertension Care Into HIV Clinics in Botswana on Improving Experiential Quality

M. Ramotsababa^1^, T. Gaolathe^2^, K. Kgotlaetsile^2^, S. Reddy^3^, T. Moshomo^4^, L. Bogart^5,6^, E. Mogaetsho^2^, M. Moagedi^2^, K. Kebotsamang^2^, M. Mosepele ^2^, L. Hirschhorn^7^

^1^Government of Botswana, Ministry of Health, Gabarone, Botswana; ^2^University of Botswana, Internal Medicine, Gabarone, Botswana; ^3^Northwestern University Feinberg School of Medicine, Infectious Disease, Chicago, United States; ^4^University of Botswana, Gabarone, Botswana; ^5^Rand Institute, Santa Monica, United States; ^6^Charles R. Drew University of Medicine and Science, Los Angeles, United States; ^7^Northwestern University Feinberg School of Medicine, Medical Social Sciences, Chicago, United States

**Background**: As effective treatment has prolonged survival among people who acquired HIV (PWH), designing health systems to manage comorbidities including hypertension (HTN) while ensuring people‐centred care (PCC) is needed to reduce morbidity and mortality and improve retention in care. PCC is measured as health system responsiveness (HSR) through patient‐reported experiential quality measures (PREMs) and health system quality outcomes. InterCARE, funded through U.S. National Institutes of Health, conducted a randomized controlled type‐2 implementation hybrid trial to determine the impact of integrating HTN care into HIV care on blood pressure control. We conducted a substudy to measure if integration improved PREMS and health system quality outcomes.

**Methods**: We measured PREMS in five intervention/control pairs using validated tools. Areas measured included communication, respect, shared decision‐making (5‐point Likert scale) and health system quality outcomes (confidence in providers, likelihood to see the same provider, trust and meeting the patient's healthcare needs). Total HSR score was calculated by summing responses of individual area scores and normalizing 0−100. A total of 250 individuals from five paired intervention and control sites were convenience sampled. Data were analysed using descriptive statistics and Generalized Linear Mixed Model to determine factors associated with HSR scoring.

**Results**: 500 participants (259 control, 241 intervention) were surveyed (median age 52 [IQR 44−59], 62.6% female, 13% no formal education). Ratings were significantly higher (*p* < 0.001) in intervention than control sites in all HSR areas except wait time and availability of equipment and supplies. HSR score was higher in intervention sites (mean 73.6 [SD 17.34]) than control sites (mean 66.5 [SD 15.74], *p* < 0.0001) (Table 1). Higher ratings were also seen for intervention sites in overall healthcare met needs, trust, confidence in providers, likelihood of seeing the same provider and recommending the clinic (all *p* < 0.001). In a multivariable analysis, only receiving HIV/HTN care in intervention site and reporting any income in last year remained significant for higher HSR.

**Conclusions**: Integration of hypertension into HIV clinics resulted in higher HSR and confidence and trust in providers and healthcare needs met. Results support integration to improve care experience and outcomes for PLH and HTN.

**TABLE 1:** OAE3704 | Health system quality outcomes by study arms.

	Intervention % (*N*)	Control % (*N*)	*p*‐value
How well did the care received meet your health needs			
Excellent/VG	88.4 (213)	70.6 (183)	<0.0001
Trust skills and abilities of provider			
Very/Somewhat	90.5 (218)	70.3 (182)	<0.0001
Likelihood to be seen by same provider			
Very Likely/Likely	71.0 (171)	65.3 (159)	<0.0001
Recommend the clinic to someone with HIV			
Very Likely/Likely	(89.3) 215	66.0 (171)	<0.0001

## OAE3705 Challenges in Integrating Cervical Cancer and HIV Services: Experiences of Women Accessing Care in Nairobi, Kenya

K. Kigen ^1^, G. Wango^2^

^1^University of Wales, Trinity St David, Institute of Education and Humanities, Lampeter, United Kingdom; ^2^St Paul's University, Philosophy and Theology, Limuru, Kenya

**Background**: Integration of cervical cancer screening into HIV services is critical for comprehensive care among women living with HIV. However, barriers to integration limit access, uptake and continuity of essential services. This study aimed to explore the challenges women face when accessing integrated cervical cancer and HIV services in Nairobi, Kenya.

**Methods**: We conducted a mixed‐methods study from January to June 2025 at three public and private health facilities providing HIV care. The study included 250 women aged 18–49 years living with HIV, recruited using purposive sampling. Quantitative data on service utilization and wait times were collected through structured surveys, while qualitative data on experiences and perceived barriers were gathered through in‐depth interviews and focus group discussions. Data were analysed using descriptive statistics and thematic analysis, with disaggregation by age and duration of HIV care.

**Results**: Among participants, 68% reported challenges in accessing cervical cancer screening within HIV care due to fragmented services, long wait times and limited provider availability. Women aged 35–49 years were more likely to face barriers related to clinic scheduling, while younger women reported stigma‐related concerns. Qualitative findings highlighted issues such as limited health education on integrated services, unclear referral pathways and inconsistent availability of screening equipment. Women emphasized the need for coordinated service delivery and community engagement to enhance uptake.

**Conclusions**: Our findings indicate that integration of cervical cancer and HIV services in Nairobi is suboptimal, limiting comprehensive care for women living with HIV. Addressing system‐level barriers, improving health provider training and engaging communities are essential to enhance service uptake, continuity and health outcomes. These insights are critical for policy‐makers and programme implementers seeking to strengthen integrated service delivery in similar low‐resource settings.

## OAE4002 Adaptive, Safety‐Centred HIV Service Delivery for Men Who Have Sex With Men in Ghana Amid Escalating Anti‐LGBTQ+ Pressures: A Pre‐Post Evaluation of Case‐Finding and Linkage Outcomes

S. E. Owusu ^1^, C. Yalley^1^, S. K. Wosornu^2^, K. Nketsiah^1^

^1^Maritime Life Precious Foundation, Programs, Takoradi, Ghana; ^2^Maritime Life Precious Foundation, Directorate, Takoradi, Ghana

**Background**: Men who have sex with men (MSM) in Ghana face escalating barriers to HIV services amid intensifying legal and political hostility. Ongoing anti‐LGBTQ+ legislative debates have heightened fear, displaced MSM from physical outreach venues and disrupted continuity of care. Consequently, traditional group‐based outreach has become unsafe and inefficient. We implemented a resilience‐focused, adaptive service delivery model to sustain HIV service access while prioritizing safety under restrictive national conditions.

**Description**: From January to December 2025, peer educators delivered discrete, one‐on‐one outreach complemented by encrypted digital platforms, rotating mobile “pop‐up” clinics and low‐profile commodity distribution points. The intervention integrated psychosocial support, multi‐month ART dispensing, doorstep delivery and anonymous WhatsApp consultations. Outcomes including MSM reached, HIV testing uptake, positivity yield and ART linkage were compared across pre‐intervention (January–June 2025) and post‐intervention (July–December 2025) phases. Qualitative data assessed participant acceptability, trust and perceived safety of the modified delivery channels. This longitudinal evaluation utilized a quasi‐experimental design to measure the model's effectiveness in bypassing structural barriers while maintaining high standards of clinical oversight and community‐led ethical governance in a restrictive landscape.

**Lessons Learned**: The number of MSM reached increased by 21%, from 690 pre‐intervention to 834 post‐intervention. HIV‐positive cases identified rose substantially from 49 to 141 (a 188% increase), while linkage to ART improved from 85% to 94%. Missed appointments decreased by 35%, reflecting enhanced treatment engagement despite the hostile environment. Digital platform integration successfully navigated 213 MSM to testing services. Qualitative feedback confirmed that participants felt significantly safer and more confident accessing services through peer‐led, digitally mediated and low‐profile physical channels compared to traditional models. Furthermore, the 188% diagnostic surge indicates that decentralized, one‐on‐one engagement fostered higher disclosure rates and reduced testing hesitancy compared to conventional venue‐based mobilization strategies.

**Conclusions/Next Steps**: Conclusions: Adaptive, safety‐centred service delivery effectively sustains HIV testing and treatment among MSM in restrictive environments. Community‐led, digitally integrated strategies overcome severe structural barriers and enhance linkage to care. These findings provide a resilient, scalable model for programming where legal constraints threaten public health gains. Sustaining these outcomes requires institutionalizing flexible, safety‐first methodologies within national HIV strategies to ensure the protection of both providers and healthcare seekers.

## OAE4003 A Beautiful Choice: Transgender‐Led Differentiated HIV Testing and Prevention Through Beauty Salon Owners and Beauticians in the Philippines

J. Laguing ^1,2^, J. D. Rosadiño^2^, R. Pagtakhan^2^

^1^DIOSSA, Taguig City, the Philippines; ^2^LoveYourself Inc., Mandaluyong City, the Philippines

**Background**: The Philippines remains off‐track towards the 95‐95‐95 targets (57‐66‐47), with transgender women among the least reached: Only an estimated one‐third have engaged with HIV prevention programmes. Stigma, discrimination and heavily medicalized pathways impede access in formal health facilities. DIOSSA, a transgender‐led organization, implemented a community‐led initiative engaging beauty salons and beauty professionals, social hubs trusted by transgender women, to deliver de‐medicalized HIV testing and prevention services in Taguig and Parañaque.

**Description**: The initiative, branded #Parlor2ParlorAttack, began in 2014 by training 20 beauticians and salon operators to distribute condoms and lubricants. A later expansion engaged 30 additional partners and introduced modules on HIV, STIs, SOGIESC and human rights. A second expansion added 20 partners to perform community‐based HIV screening and establish formal referral networks.

In its latest phase, 30 more partners were empowered to distribute HIV self‐testing kits, initiate PrEP in community settings and provide access to gender‐affirming consultations. A de‐medicalized PrEP model was developed to allow initiation and follow‐up outside clinical facilities. Across phases, 100 beauty sector partners, including salons, beauticians, salon owners, makeup artists and transgender leaders, became active HIV service delivery points.

These partners delivered multiple testing modalities (community‐based testing and self‐testing), distributed commodities and facilitated referrals for reactive clients. When a client screened reactive and required treatment initiation, a structured referral mechanism linked them to Lily by LoveYourself, a community centre for transgender populations. Lily served both as the “mother centre” supplying commodities for prevention and testing, and as the receiving facility for confirmatory testing, PrEP continuation and HIV treatment initiation.

**Lessons Learned**: In 2025, partners distributed 1971 self‐testing kits, producing a 4.2% reactivity yield and achieving 90% linkage‐to‐treatment among reactive clients. The model demonstrated that transgender‐led beauty sector networks function as non‐stigmatizing and socially embedded HIV service hubs.

**Conclusions/Next Steps**: Community leadership and participatory engagement were central to the model's success. Scaling transgender‐led differentiated delivery platforms through non‐traditional community spaces expands access, increases choice and addresses persistent inequities in reaching transgender women.

## OAE4004 Engaging Communities Through Participatory Mapping to Strengthen Responsive HIV Programming in Malawi: Lessons From the RESPOND Project

I. Robson ^1,2^, M. Mphande^2^, K. Balakasi^2^, L. Kamtsendero^2^, K. Phiri^2^, S. Macheso^3^, B. Matanje^4^, M. Cornell^5^, S. Phiri^2,6^, K. Dovel^1,2^

^1^University of California, Los Angeles, Division of Infectious Diseases, David Geffen School of Medicine, Los Angeles, United States; ^2^Partners in Hope, Lilongwe, Malawi; ^3^Malawi Ministry of Health, Directorate of HIV, STIs and Viral Hepatitis, Lilongwe, Malawi; ^4^Malawi National AIDS Commission, Lilongwe, Malawi; ^5^University of Cape Town, CIDER (Centre for Integrated Data and Epidemiological Research), School of Public Health, Cape Town, South Africa; ^6^Kamuzu University of Health Sciences, School of Global and Public Health, Lilongwe, Malawi

**Background**: Despite progress in HIV programmes, disparities persist between communities. Community engagement can improve programme acceptability and sustainability by aligning interventions with local needs and resources, yet communities are rarely involved in health systems planning. We developed a participatory mapping approach to identify and co‐create locally responsive HIV programmes for communities experiencing increased need.

**Methods**: We conducted 1‐day participatory mapping workshops in 15 facility catchment areas across Malawi, facilitated by two programme coordinators. Each workshop included 15−20 multi‐sectoral stakeholders, evenly divided between healthcare workers (HCWs) and community representatives (local leaders, people living with HIV, youth, key populations). Workshops aimed to:

1. map geospatial areas with unmet HIV service needs, contextual factors and risk behaviours impacting service engagement;
2. identify geographic “priority areas” where needs and behaviours were most concentrated; and
3. co‐create interventions informed by needs and existing strengths within priority areas.

We captured data in visual maps and structured memos. Ethnographic notes documented workshop dynamics. We iteratively refined the approach and developed an implementation toolkit for replication.

**Results**: Fifteen workshops engaged 245 stakeholders (136 men). Participants identified 3−5 priority areas per catchment area, with HIV testing and treatment interruption the most common service gaps (Figure 1). Key contextual factors were seasonal economic activity, poverty and extreme weather. Risk behaviours included transactional sex, mobility and cultural practices (Figure 2). Participants identified major gaps such as limited outreach and person‐centred care, and felt general populations, not key populations, needed additional services. This information directly informed solutions optimizing existing services (e.g. adapting community‐based outreach for other conditions and improving flexibility at facilities). Stakeholders liked the workshops, valuing the collaborative goal setting process. Community members felt heard and HCWs reported improved clarity on partnering with communities.

**Conclusions**: Participatory mapping generated insights into contextual factors, unmet needs and service adaptations needed to tailor services to priority areas. Geographic prioritization incorporating multi‐sectoral perspectives was feasible and effective, producing actionable plans with buy‐in across stakeholders. This method operationalizes “nothing for us without us,” offering a replicable approach for national HIV planning to ensure interventions reflect community needs, priorities and strengths.

**FIGURE 1:**
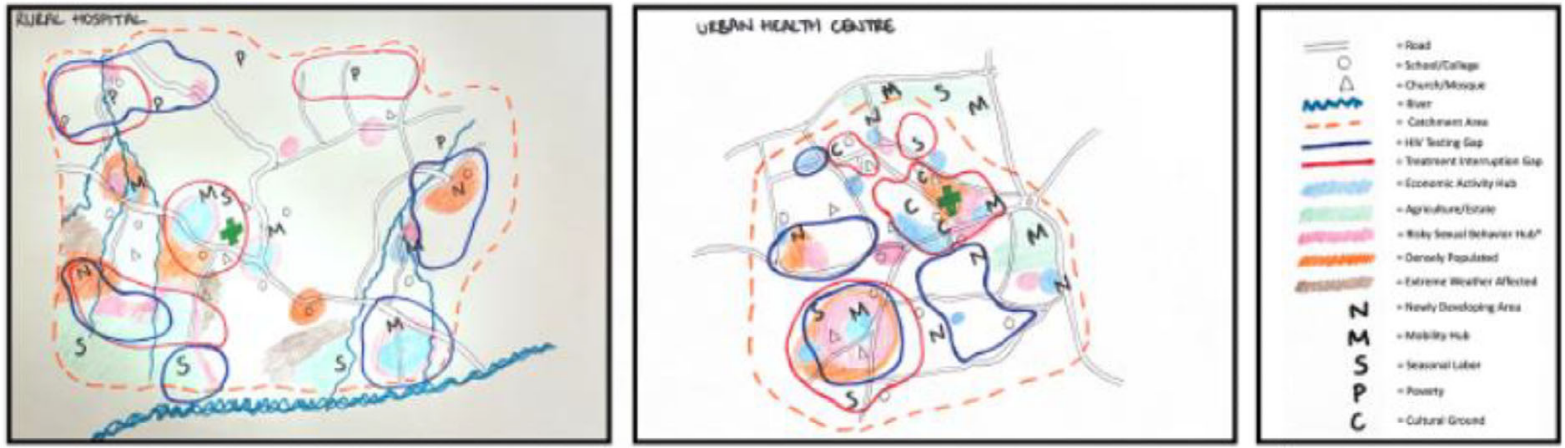
OAE4004 | Illustrative anonymized maps from two facilities mapping HIV service needs, contextual factors and risky behaviours to identify priority areas.

**FIGURE 2:**
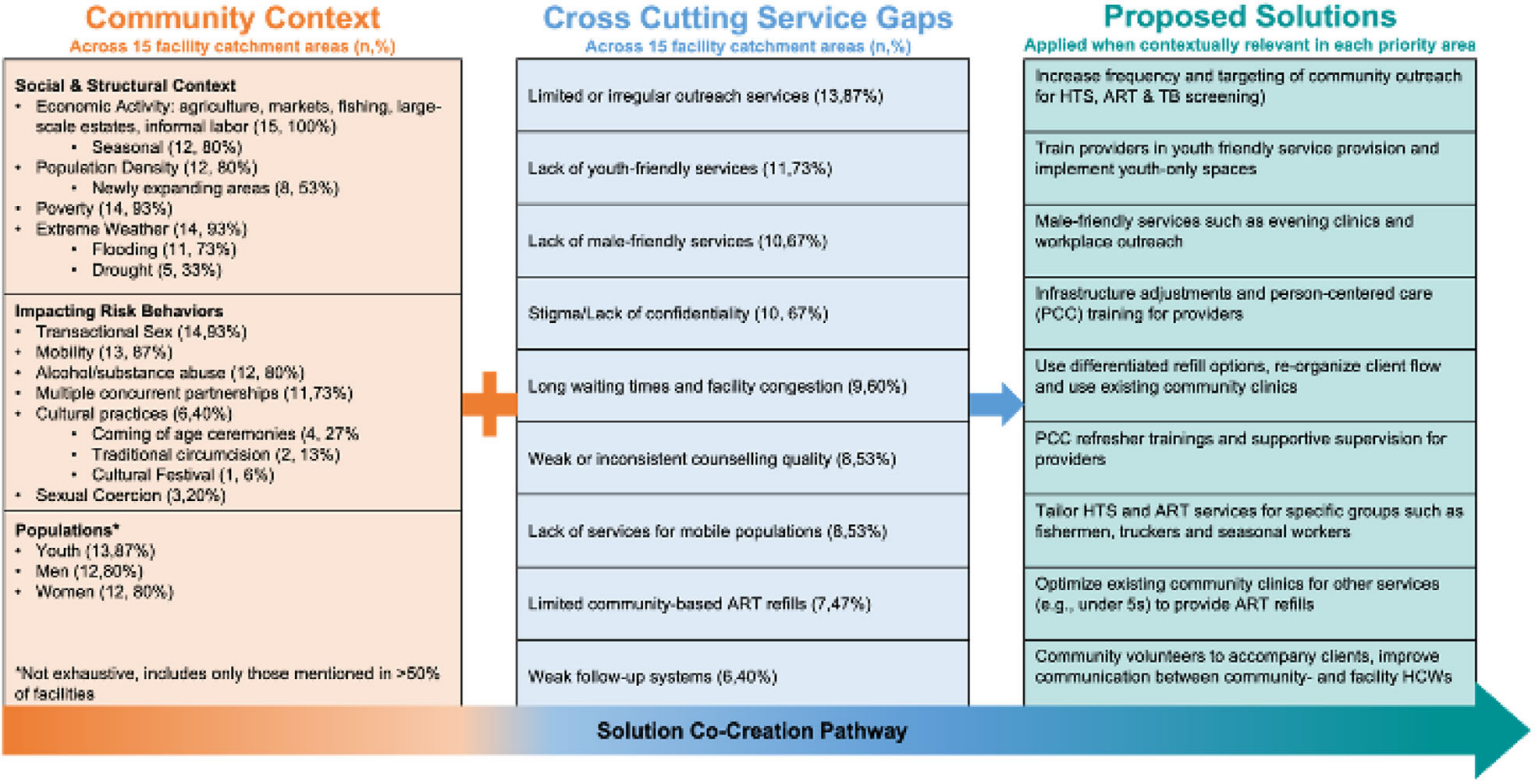
OAE4004 | Workshop solution co‐creation pathway: considering contextual factors and service gaps to co‐create solutions for addressing priority areas.

## OAE4005 Impact of Community‐Based Interventions on Viral Load Among Adolescents and Young Adults Living With HIV

T. Lewis, E. Carras‐Terzian, S. Charles, M. Masson

Caris Foundation International, Port‐au‐Prince, Haiti

**Background**: Adolescents and young adults (AYA) living with HIV experience persistent challenges in achieving and sustaining viral suppression, due to developmental, social and structural barriers to care. Community‐based interventions are increasingly used to address these gaps, yet empirical evidence linking integrated community platforms to virologic outcomes at scale remains limited. This study assessed the impact of community programmes and community‐based adherence support (CAD) interventions on viral suppression and community viral load among AYA enrolled in Caris' Foundation International (CFI) HIV programme in Haiti.

**Description**: We conducted a retrospective cohort analysis using routine programme data collected between January 2015 and January 2026 among AYA aged 13–25 years living with HIV. The final sample included 2966 clients (45.7% female; median age 17 years [IQR: 15–20]). Viral suppression was defined as viral load (VL) <1000 copies/mL. Associations between suppression and demographic, educational and programme participation factors were examined using chi‐square and Mann–Whitney U tests. Propensity score matching (PSM) reduced selection bias, and dose–response analyses assessed cumulative and synergistic effects of structured community programmes and CAD interventions.

**Lessons Learned**: A total of 20,851 VL measurements were analysed. 89% percent of participants achieved viral suppression at least once during follow‐up. Participation in psychosocial support groups was associated with higher suppression rates than non‐participation (83.7% vs. 70.6%, *p* < 0.0001). Age group, schooling status and programme participation were significantly associated with viral suppression (*p* < 0.0001). PSM analyses confirmed a treatment effect of community interventions. Dose–response analyses identified two pathways: cumulative benefits from structured programmes and stronger synergistic effects when combined with CAD interventions (psychosocial clubs, home visits and follow‐up calls).

**Conclusions/Next Steps**: Integrated community‐based interventions significantly improve viral suppression and reduce community viral load among AYA living with HIV. These platforms operate as Treatment as Prevention and should be systematically integrated and sustainably financed within national HIV strategies. A multi‐level risk‐based approach, offering a minimum package of structured community programmes for all AYA in care with targeted CAD intensification for high‐risk youth—offers an efficient pathway towards epidemic control by 2030.

## OAF0602 Trips Safeguard in Practice: Patent Opposition as a Rights‐Based Tool for PrEP Access in Brazil

C. T. Scopel, S. van der Ploeg, V. Terto Junior

Brazilian Interdisciplinary AIDS Association (ABIA), Rio de Janeiro, Brazil

**Background**: Patent monopolies can delay generic entry and sustain high prices for HIV prevention technologies, threatening the affordability and scale‐up of pre‐exposure prophylaxis (PrEP) within universal health systems. Brazilian industrial property law allows third parties to challenge patent applications, providing arguments before or after the patent is granted (pre‐ or post‐grant opposition). This study aims to describe civil society experience combining patent opposition and community mobilization to protect PrEP access in Brazil.

**Description**: The Working Group on Intellectual Property (GTPI) has been using patent opposition since 2006 as part of broader access‐to‐medicines advocacy. In 2010 and in 2016, GTPI filed oppositions on a patent application (PI0406760‐6, WO2004064845) for the fixed‐dose combination tenofovir disoproxil fumarate/emtricitabine (TDF/FTC), arguing lack of inventive step for an obvious combination of known medicines. Additionally, since 2016, the “Truvada Livre!” campaign lobbied for the rejection of the application and massive distribution of the medicine as PrEP through the public health system. In 2018, the application was definitely rejected by Brazil's Patent Office, TDF/FTC has remained free of patent barriers, enabling domestic generic production at a reduced price. Since 2024, GTPI has been promoting debates, engaging in dialogue with the government, public producers and other organizations in order to anticipate barriers to access to lenacapavir, the injectable PrEP. In 2025, after conducting a patent landscape, GTPI filed a patent opposition on a secondary application (BR112024010162‐2, WO2023102239) related to lenacapavir prodrugs. This application is still pending a final decision.

**Lessons Learned**: The Truvada experience shows that civil society initiatives can prevent undue exclusivity surrounding high‐impact prevention technologies and support conditions for competition. For newer long‐acting options, monitoring patent landscape and challenging patent barriers can contribute to address access. Being embedded in PLWHA networks strengthens legitimacy and helps translate technical intellectual property disputes into rights‐based, public‐interest accountability demands.

**Conclusions/Next Steps**: Civil society initiatives to challenge patent barriers can contribute to generic local production, price reduction environment and increasing access. Next steps include continuous monitoring of patent landscape, development of productive capacities, price monitoring and advocating for increased access in coordination with PLWHA networks.

## OAF0603 Reduction of HIV Stigma and Discrimination Among Key Populations in Nigeria Through Community‐Led Interventions

O. J. Ifeanyi, G. Kalu

The Initiative for Equal Rights (TIERs), Research and Knowledge Management Unit, TIERs, Mende, Maryland, Nigeria

**Background**: Despite Nigeria's HIV/AIDS Anti‐Discrimination Act of 2014, people living with HIV (PLHIV) continue experiencing systematic discrimination. The 2021 NEPHWAN Stigma Index Survey of 1240 PLHIV across 17 Nigerian states revealed that 22% experienced stigma and discrimination, with 7% reporting health workers disclosing their HIV status without consent. Among those whose rights were violated, 40.8% did not seek redress due to unawareness of the legal mechanisms, while 12% feared disclosure of their status. These findings expose critical gaps in awareness of legal protections among PLHIV, healthcare workers and employers; gaps this project addresses through a multi‐layered intervention approach.

**Description**: The “Tackling Multi‐Layered Stigma in Healthcare Settings and Dismantling Discriminatory Laws in Nigeria” project, funded by ViiV Healthcare, launched in January 2025 with four key interventions. First, partnerships across six government facilities in Nigeria raise awareness of the Anti‐Discrimination Act and Patient Bill of Rights through multilingual IEC materials. Second, 10 trained PLHIV champions, including sex workers, MSM and LGBTQIA+ individuals, will conduct monthly U = U campaigns. Third, workplace anti‐discrimination campaigns engage 12 employers on DEI policies. Fourth, an emergency reporting channel integrated into TIERs’ existing SOGIESC‐based human rights networks provides access to litigation support for cases involving HIV discrimination.

**Lessons Learned**: Within 1 year, 59 healthcare workers across six government facilities in Lagos, Ogun and Ekiti States in Nigeria co‐developed multilingual IEC materials such as brochures, PSAs and educational manuals, in English, Igbo, Yoruba, Hausa and Pidgin, now awaiting NACA approval for national rollout. Ten trained PLHIV peer advocates amplified this work through 18 social media campaigns reaching 10,968 people, while also referring peers to TIERs for access to justice. Their referrals enabled legal support for four PLHIV facing employment discrimination, resulting in ₦2.5 million compensation and a formal apology in two cases.

**Conclusions/Next Steps**: Even in Nigeria, where the Same‐Sex Marriage Prohibition Act criminalizes same‐sex relations, community‐led approaches drive real change. We co‐created IEC materials with PLHIV champions, many LGBTQIA+ individuals and healthcare workers, building trust through authentic and relatable messaging. Peer advocates ran campaigns from lived experiences. Next, we will engage 12 employers on anti‐discrimination policies, push state‐level Act adoption and launch #IStandAgainstDiscrimination campaigns nationwide.

## OAF0604 Community‐Led Approaches to Reducing Human Rights–Related Barriers in HIV Services in Indonesia

A. A. Cindy

Indonesia AIDS Coalition, Non Government Organization, South Jakarta, Indonesia

**Background**: Human rights–related barriers remain a critical structural constraint to equitable access to HIV and TB services for key populations in Indonesia. Persistent stigma, discrimination, gender‐based violence (GBV), and broader human rights violations within healthcare settings and social environments undermine service uptake, delay treatment initiation and discourage survivors from reporting abuses. Addressing these barriers is essential to achieving rights‐based and community‐centred HIV responses.

**Description**: In 2024, Indonesia AIDS Coalition (IAC), with support from the Global Fund, implemented a Human Rights Program under the Community System Strengthening – Human Rights (CSS HR) framework. The programme applied a community‐ and systems‐based approach comprising:

1. documentation and response to human rights violations by paralegal officers and virtual paralegals;
2. rights awareness through sensitization and socialization activities;
3. service quality monitoring using a digital Community Led Monitoring (CLM) platform; and
4. capacity building for healthcare providers, frontliners and first responders through Medical Ethics, GBV and Human Rights trainings.

Programme data were complemented by qualitative interviews with community members, healthcare providers and local government representatives.

**Lessons Learned**: A total of 987 human rights–related cases were documented in 2024, occurring both within and outside healthcare settings. Most cases were addressed through survivor‐centred non‐litigation mechanisms, reflecting survivors’ preferences and contextual barriers to formal legal processes. The digital CLM platform collected 3836 community feedback reports, with consistently positive ratings across service availability, accessibility, acceptability and quality indicators. Qualitative findings indicate measurable shifts in attitudes and increased empathy among healthcare workers and frontliners, alongside strengthened trust between communities and service providers. Cross‐sectoral trainings also improved coordination among local government agencies in responding to HIV‐related vulnerabilities.

**Conclusions/Next Steps**: This programme demonstrates that integrating community‐led documentation, survivor‐centred case responses and real‐time service monitoring can effectively reduce human rights–related barriers to HIV and TB services. Direct engagement between communities, service providers and policymakers—supported by community‐generated data—emerged as a key enabler of inclusive and accountable health systems. The approach offers a scalable and transferable good practice model for rights‐based HIV responses in similar settings.

## OAF0605 Rethink, Rebuild, Rise: Strengthening HIV Service Quality and Accountability Through Community‐Led Monitoring in Lesotho

N. Makhooa

Bacha Re Bacha Youth Forum, Programs and Monitoring and Evaluation, Maseru, Lesotho

**Background**: Despite progress towards HIV epidemic control, persistent service quality gaps, stigma and accountability challenges continue to affect HIV outcomes in Lesotho. Conventional monitoring systems often fail to capture lived experiences of people accessing care. In alignment with the IAS 2026 theme Rethink, Rebuild, Rise,

The implementation of Community‐Led Monitoring (CLM) aimed to reconsider the creation of evidence, restore community‐health system trust and assist communities in becoming active participants in the HIV response.

**Methods**: Starting from 2023 to current, CLM was implemented in selected six of the 10 districts in Lesotho being Mokhotlong, Thaba‐Tseka, Quthing, Leribe, Qacha and Butha‐Buthe by trained community data monitors (adolescent girls and young women [AGYW]) who systematically collect data on service accessibility to AGYW, waiting times, stock‐outs, confidentiality, stigma and provider attitudes across 96 public health facilities. Data were analysed monthly and quarterly using excel and shared through structured feedback dialogues with facility managers, district Health Management Teams and Ministry of Health to inform corrective actions—Have clear commitments and resolutions to identified problems and timeframes of when to solve such issues.

**Results**: CLM documented a reduction in reported service‐related stigma, decrease in waiting times and reduction in commodity stock‐outs for Ministry of Health and Red Cross facilities while there is still a need for strengthened advocacy on stock‐outs of commodities at Christian Health Association of Lesotho (CHAL) facilities. Facilities implementing CLM‐informed actions demonstrated improved linkage to care. Community data monitors reported increased confidence and leadership in health advocacy, while facility staff reported improved communication and responsiveness to community needs.

**Conclusions**: Community‐Led Monitoring generates actionable, people‐centred evidence that strengthens accountability, service quality and trust in HIV programmes. Rethinking monitoring systems, rebuilding collaborative relationships and enabling communities to rise as leaders are critical for resilient, rights‐based HIV responses and sustained epidemic control.

## OAF1202 Impact of Coordinated Market Shaping Investment for Lenacapavir Access in Low‐ and Middle‐Income Countries

L. Pillay^1^, K. Mthombeni^1^, V. Butler ^1^, N. Naidoo^1^, E. Briedenhann^1^, S. Mullick^1^, C. Amole^2^, S. Jenkins^2^, D. Resar^2^

^1^Wits RHI, University of the Witwatersrand, Implementation Science, Johannesburg, South Africa; ^2^Clinton Health Access Initiative, HIV Prevention, Boston, United States

**Background**: The regulatory approval of twice‐yearly injectable lenacapavir marks a pivotal moment, fuelling optimism that we finally tools to change the trajectory of the HIV epidemic. Realizing this potential, however, depends on the rapid and coordinated alignment and implementation of supply‐ and demand‐side market‐shaping interventions. Historically, global health markets have underperformed when supply‐side incentives are prioritized without parallel investment in demand‐side readiness, resulting in fragmented implementation, delayed uptake and limited impact. This case study examines a coordinated market‐shaping approach implemented by strategic partners, Wits RHI and CHAI through Unitaid's investment to accelerate access to affordable generic LEN while strengthening country readiness for introduction.

**Description**: Unitaid rapidly funded targeted technical assistance to catalyse demand and supply‐side interventions. This coordinated approach enabled early visibility of product development timelines, anticipated volumes and pricing assumptions. In parallel, demand‐side interventions, structured around a product value‐chain framework, focused on addressing policy, regulatory, supply chain, health system, community engagement and advocacy requirements for achieving scale. Further Unitaid also invested in implementation science studies to understand the potential demand and develop implementation materials for providers and communities.

**Lessons Learned**: Integrating supply‐ and demand‐side interventions through strategic partnerships enabled rapid feedback loops that informed affordability negotiations and national scale‐up planning. On the supply side, this coordination contributed to securing an access price of US$40 (near price parity with oral prevention therapy) per patient per year for generic injectable LEN. Concurrently, early clarity on pricing and timelines catalysed demand‐side actions including PrEP guideline updates; clinical training packages with job aids and IEC materials; quantification and forecasting; regulatory approvals; facility readiness and implementation planning; demand‐generation strategies; and community engagement models. These activities enabled Zambia to commence with LEN introduction in the same year as high‐income countries, with product introduction imminent for South Africa, Kenya and Nigeria.

**Conclusions/Next Steps**: Early, coordinated investment across supply and demand levers enabled agreement on affordable generic LEN while achieving country readiness for rapid scale up following regulatory approval of generic LEN. This case demonstrates how integrated market‐shaping strategies can mitigate market failure and accelerate equitable access to transformative HIV prevention innovations.

## OAF1203 Cracking the Code to Remove Patent Barriers

S. Kondratyuk, M. F. Pignataro, G. Costa Chaves, T. Swan, D. Peeler, M. Ahmar, O. Mellouk

ITPC Global, Johannesburg, South Africa

**Background**: Unmerited pharmaceutical patents lead to high prices and limited access to essential medicines. Legal measures, called patent oppositions (POs), can be used to prevent or reject unmerited patents, and enable access to affordable generic medicines. POs can be one of the most accessible legal options for civil society (CS) and community‐based organizations (CBOs) to improve access to medicines.

The Make Medicines Affordable (MMA) consortium, a group of CS and CBOs from 24 low‐and middle‐income countries (LMICs), is leading patent opposition work in Africa, Eastern Europe and Central Asia, Latin America and South East Asia. Since 2015, MMA partners have filed 149 challenges to filed and granted patents on pharmaceutical products, 59 of which were for 15 HIV antiretrovirals (ARVs).

**Description**: POs are technical, and require specialized scientific expertise, which is not always available in LMICs. Examples of successful POs are not always available from countries where local expertise exists. To address this gap, the MMA Science Team of experienced pharmaceutical chemists has prepared and disseminated 32 global‐level patent opposition templates, for adaptation by national CS organizations. These templates have facilitated POs, especially for CS and CBOs who are filing for the first time and they are time savers for partners with more experience filing POs. Overall, 25 of these PO templates were used to file 59 POs, including six oppositions using the nirmatrelvir/ritonavir template; and five oppositions each, on lenacapavir, doravirine and molnupiravir templates.

**Lessons Learned**: Use of PO templates facilitated the preparation of POs at national and regional levels and enabled joint multi‐country PO campaigns. MMA's experience underscores the importance of capacity‐building and technical support for successful PO success.

**Conclusions/Next Steps**: The MMA experience demonstrates that, despite the complex technical aspect of POs, local CS and CBOs are able to file successful cases; 38% of POs filed by MMA members were successful, while 43% are in process. PO templates enabled the filing of additional patent oppositions on prioritized medicines across multiple countries.

## OAF1204 Implementation of the First Compulsory Licence in Colombia for a Medicine: The Case of Dolutegravir

H. H. Silva Carvajal ^1^, J.f. Pínzon Zakzuk^1^, A. C. Reyes Rojas^1^, J. Lopez Mendez^1^, O. Andia Salazar^2^

^1^Fundación IFarma, Bogotá D.C., Colombia; ^2^Fundación Observamed Star, Bogotá D.C., Colombia

**Background**: Colombia's first progressive government was elected in 2022, enabling the declaration of public interest and implementation of a compulsory license (CL) to import and acquire generic versions of dolutegravir (DTG) a World Health Organization‐recommended antiretroviral (ARV), to benefit people living with HIV. In preparation to implement the DTG CL, Colombia's government enabled international purchasing mechanisms, developed the technical infrastructure necessary to support evidence‐based decision‐making and launched access to lower‐priced generic versions of DTG, through the PAHO Strategic Fund.

**Description**: The IFARMA Foundation has been monitoring the implementation of the DTG CL in Colombia, as well as defending the DTG CL as an intervener in various legal proceedings to block the CL. To support the CL, IFARMA has examined the relationship between the CL and price reduction, expansion of therapeutic coverage and financial sustainability of the government's healthcare system.

**Lessons Learned**: Compulsory licensing led to a significant reduction in the price of dolutegravir (from COP 410,000 to COP 14,000), enabling more efficient use of public resources. This cost reduction facilitated the expansion of treatment coverage and contributed to strengthening sustainability of the national health system. TRIPS public health safeguards, such as CLs, are cost‐effective tools for addressing access barriers arising from pharmaceutical monopolies.

**Conclusions/Next Steps**: Compulsory licenses are a key strategy for improving access to HIV treatment and advancing towards epidemic control goals, and they are cost‐effective. In this case, the importation of DTG through the PAHO Centralized Fund lowered DTG's price from 410,000 COP to 14,000 COP per person, per year, translating into a 96.59% reduction. Stricter guidelines on patentability may be even more effective in reducing unjustified pharmaceutical monopolies and making better use of health system resources.

## OAF1205 Overcoming “TRIPS‐plus” Barriers: Lessons Learned From Armenia's Legal Reforms and Patent Challenges for HIV Medicines

A. Harutyunyan ^1^

^1^Positive People Armenian Network Social NGO, Yerevan, Armenia

**Background**: In Armenia, the right to health often clashes with rigid intellectual property (IP) rules. It is more than a legal concept; it is a daily reality for those seeking life‐saving HIV treatment. Currently, this right is under threat from rigid “TRIPS‐plus” patent rules that favour industry monopolies over people. Our study moves beyond technical policy analysis to capture the lived experience of “Positive People Armenian Network” Social NGO, advocates to dismantle the legal “deadlocks” that keep medicine prices high and generic alternatives out of reach.

**Description**: This analysis details how the “Positive People Armenian Network” Social NGO navigated Armenia's 2021 Patent Law reforms. We moved beyond observation to launch a landmark court challenge against the Tenofovir/Emtricitabine monopoly. We evaluate tools like the “Bolar exception,” currently too limited for immediate generic entry, advocating for its expansion to support a “first‐day launch.” Additionally, the study examines our push for a faster administrative compulsory licensing system. This reform empowers the Armenian government to prioritize public health during crises, bypassing judicial delays to ensure sustainable, life‐saving treatment access for the entire community.

**Lessons Learned**: Findings reveal “procedural traps” such as the “Positive People Armenian Network” dismissal for lacking “legal standing.” Although the “8+1+1” rule freezes competition for a decade, “pre‐grant” oppositions offer a “silver lining,” enabling advocates to block pharmaceutical “evergreening” and unfair monopoly extensions.

**Conclusions/Next Steps**: Law must ultimately serve humanity, not just industry. For Armenia to achieve sustainable HIV care, the civil society organizations need a guaranteed “seat at the table” in the legal system. Key recommendations include:

- Establishing administrative compulsory licensing to allow for swift government action.
- Expanding the “Bolar exception”; a legal provision allowing generic manufacturers to conduct necessary tests and registration procedures while a patent is still active, to ensure medications are ready for a “first‐day launch” the moment a monopoly expires.
- Fostering a comprehensive interagency approach to monitor the impact of IP barriers on treatment access.

Empowering people living with HIV to be the primary watchdogs of the laws that govern their lives is the only path to a truly just system.

## OAF1702 Criminalization and HIV Treatment Continuity Among People Who Use Drugs in 10 Latin American Countries

M. S. Quintanilla Cantizano ^1^, R. E. Valencia Gil^2^

^1^Latin American and Caribbean Network of People Who Use Drugs, San Salvador, El Salvador; ^2^Fundación Ancla, Educación e Investigación Comunitaria, Medellin, Colombia

**Background**: Across Latin America, HIV responses operate within legal and policy environments that criminalize drug use, creating structural barriers to public health and human rights. While most countries formally recognize the rights to health and non‐discrimination for people living with HIV, a critical gap persists between legal guarantees and institutional practice. This study presents the first multi‐country analysis in Latin America linking drug use criminalization with antiretroviral therapy (ART) continuity and effective access to accountability mechanisms among people living with HIV who use drugs (PLHIV‐PWUD), with the aim of informing rights‐based public policy reform.

**Methods**: A mixed‐methods regional study was conducted between 2023 and 2024 in Bolivia, Costa Rica, Ecuador, El Salvador, Guatemala, Honduras, Nicaragua, Panama, Paraguay and Peru. Quantitative data were collected through a self‐administered survey of 399 people living with HIV who use drugs, recruited via community‐based organizations and peer networks. Data were disaggregated by age and gender, including men, women and non‐binary/trans participants. The qualitative component included a comparative review of legal frameworks, drug policies and HIV regulations, alongside an assessment of institutional practices related to health access, non‐discrimination and accountability. De jure and de facto findings were integrated using the Traffic Light (Penta‐Semáforo) methodology.

**Results**: Participants were 51% men, 37.6% women and 11.4% non‐binary/trans, predominantly aged 25–34 years. Findings reveal three systemic failures: (i) ineffective complaint mechanisms and low institutional trust, (ii) persistent stigma within healthcare services and (iii) punitive policing practices. Overall, 74% of participants reported distrust in formal reporting mechanisms. In addition, 52% reported experiencing arbitrary detention events that directly disrupted ART adherence. Non‐binary and trans participants consistently reported higher levels of harassment and rights violations.

**Conclusions**: Drug use criminalization functions as a structural determinant undermining ART continuity and weakening HIV responses across Latin America. By providing the first region‐wide evidence on how policing and detention disrupt treatment, this study offers guidance for reorienting HIV and drug policies towards public health and human rights–based approaches. Sustainable outcomes require decriminalization measures, explicit legal protection for harm reduction services, safeguards to ensure ART continuity during detention and accessible accountability mechanisms, with a differentiated focus on non‐binary and trans communities.

## OAF1703 Legal Political Determinants of Access to HIV Services: Lessons Learned From 12 Years of Global Fund Programme Implementation and Policy Engagement in Cameroon, With Comparative Insights From Benin and DRC

G. M. Mendo Ze

CAMNAFAW, Programmes et Recherches, Yaoundé, Cameroon

**Background**: This abstract is adapted from lessons learned through long‐term programmatic implementation and policy engagement on HIV and sexual and reproductive health and rights (SRHR). The purpose is to examine how legal and political frameworks shape access to HIV services for vulnerable populations. The scope focuses on Cameroon, with comparative insights from Benin and the Democratic Republic of the Congo (DRC). The objectives are to document policy‐related barriers and facilitators, assess alignment with Article 14 of the Maputo Protocol and inform global policy discussions ahead of the 2026 United Nations High‐Level Meeting on HIV/AIDS.

**Description**: The analysis draws on over 12 years of HIV programme implementation led by the Cameroon National Association for Family Welfare (CAMNAFAW) as Principal Recipient of Global Fund grants. The programme was implemented nationally and targeted people living with HIV, women, young people and key populations. Activities included service delivery support, policy monitoring, documentation of legal barriers affecting access to HIV services and structured engagement with policymakers. A Parliamentary Task Force was established to support the domestication of the Maputo Protocol, providing an institutional mechanism for legislative review, multisectoral dialogue and accountability. Comparative policy experiences from Benin and the DRC were reviewed to contextualize reform pathways.

**Lessons Learned**: Key lessons indicate that criminalization, stigma and restrictive reproductive health laws undermine HIV prevention, treatment continuity and service uptake. Programmatic data show that fear of prosecution and discrimination contributes to delayed testing and care avoidance. Conversely, sustained parliamentary engagement and rights‐based policy dialogue—such as through the Task Force mechanism—emerge as effective practices for advancing legal reform and aligning national frameworks with regional commitments.

**Conclusions/Next Steps**: These findings highlight the central role of legal and political reform in strengthening HIV responses. Ahead of the 2026 UN High‐Level Meeting on HIV/AIDS, countries should align national laws with Article 14 of the Maputo Protocol, institutionalize parliamentary accountability mechanisms and integrate rights‐based approaches into HIV policies to improve prevention, treatment, care and support outcomes.

## OAF1704 Against the Grain: From Policing to Partnership—Community‐Led Safety, Rights and Service Access for Sex Workers in Peri‐Urban Mining Communities Near Bulawayo, Zimbabwe

B. Runya

Zimbabwe Open University, Development Studies, Bulawayo, Zimbabwe

**Background**: Sex workers in Zimbabwe experience intersecting harms driven by criminalization, stigma and routine violence, including harassment and exploitation by clients, third parties and authorities. In peri‐urban mining communities around Bulawayo, these risks are intensified by mobility, informal economies and limited, stigmatizing services, undermining HIV prevention and treatment uptake and delaying reporting and care after gender‐based violence (GBV). Adult Glam Divas (AGD), a registered sex worker–led network, implemented a rights‐based community model to improve safety, psychosocial wellbeing and access to HIV/sexual and reproductive health (SRH) and GBV/legal services in Mazwi, St Peters, Robert Sinyoka and Esigodini.

**Description**: Implemented April–September 2025 in four peri‐urban mining communities near Bulawayo, the programme served adult sex workers working in these areas. AGD trained peer facilitators to deliver:

1. moderated WhatsApp daily dialogues and safety alerts
2. rapid‐response escalation pathways
3. peer psychosocial first‐line support with counselling referral options
4. rights literacy and leadership sessions (consent, safer‐sex negotiation, incident documentation and service navigation)
5. structured “warm referrals” plus optional peer accompaniment to HIV/SRH, GBV and legal services.

**Lessons Learned**: About 160 sex workers participated via eight WhatsApp groups. Facilitators logged ∼95 safety alerts, including ∼58 GBV‐related incidents; ∼70% received support within 24 h (safety planning, peer check‐ins, accompaniment and referrals). Around 120 participants received referrals to HIV/SRH services and ∼85 completed a facility visit within 1 month. Among survivors needing GBV care, ∼34 accessed clinical services (including PEP where indicated) and ∼22 received legal advice and/or assistance with survivor‐centred documentation. Participants described increased confidence to refuse unsafe sex, improved knowledge of rights and service options, and reduced isolation through peer solidarity. Best practices were strict confidentiality/consent procedures, consistent facilitator presence and warm referrals with follow‐up to reduce stigma‐related drop‐off.

**Conclusions/Next Steps**: A sex worker‐led, rights‐based approach combining digital peer support, rapid response and warm referrals can improve safety and increase HIV/SRH and GBV service uptake in high‐stigma peri‐urban mining areas. It supports prevention (safer sex, timely PEP), treatment linkage and ongoing care. Next steps include formalizing clinic and GBV/legal referral protocols, expanding to more sites, strengthening peer supervision and trauma support, and improving confidential monitoring of referral‐completion and time‐to‐service.

## OAF1705 Strengthening Trans‐Led Organizational Resilience to Anti‐Gender Movements to Safeguard HIV‐Related Rights and Access: Lessons From a Global Capacity‐Building and Advocacy Programme

B. Chitsanupong ^1^, E. Castellanos^2^, C. Euzebio de Lima^3^, D. Ocheret^4^, L. Berianidze^5^

^1^Global Action for Trans Equality, Chiang Mai, Thailand; ^2^Global Action for Trans Equality, Mijdrecht, the Netherlands; ^3^UNAIDS, Bangkok, Thailand; ^4^UNAIDS, Geneva, Switzerland; ^5^GATE – Global Action for Trans Equality, Barcelona, Spain

**Background**: Anti‐gender movements target trans and gender diverse communities through criminalization, surveillance, violence and funding restrictions. This hostile context exacerbates stigma and discrimination and undermines access to HIV prevention, treatment and care. This programme aimed to strengthen the capacity of trans‐led organizations to strategize, prevent and respond to anti‐rights movements, while reinforcing community protection and enabling effective engagement in rights‐based HIV responses.

**Description**: Global Action for Trans Equality (GATE), in partnership with UNAIDS, implemented a multi‐country programme combining capacity strengthening, regranting, knowledge production and advocacy. Fifteen trans‐led organizations across regions were supported through funding and technical assistance. It included production of an evidence‐based regional analysis of anti‐trans oppression in South‐West Asia and North Africa, two advocacy toolkits and online learning courses, capacity‐building grants to implement context‐specific anti‐gender response activities and support for submissions to the Universal Periodic Review and UN treaty bodies.

**Lessons Learned**: Programme outputs included one regional anti‐gender report, two online training programmes reaching more than 60 countries, nine capacity‐building grants delivered across eight countries and six trans‐led submissions to UN mechanisms across five countries. More than 20 activities were implemented globally, with over 100 participants and allies reporting increased knowledge and skills, and digital campaigns exceeding 100,000 in online reach. Grantees reported that community‐owned documentation and storytelling strengthened confidence, strategic advocacy and alliances, including with broader civil society and institutional stakeholders. These results indicate that investment in trans‐led capacity and protection infrastructure can strengthen movement resilience under human rights attacks and sustain engagement in rights‐based HIV advocacy.

**Conclusions/Next Steps**: Anti‐gender movements are a direct structural threat to equitable HIV responses for trans and gender diverse communities and require flexible, safety‐centred programming that integrates documentation, legal literacy, psychosocial support and digital security as core elements, not separate “add‐ons.” This programme shows that resourcing trans‐led organizations to monitor and counter anti‐rights tactics, protect community wellbeing and engage with human rights mechanisms can generate significant gains. Next steps include expanding peer learning and strengthening safeguarding and digital security systems. These actions are critical to protecting HIV‐service continuity and advancing rights‐based, community‐led HIV responses in increasingly restrictive contexts (see Figure 1).

**FIGURE 1:**
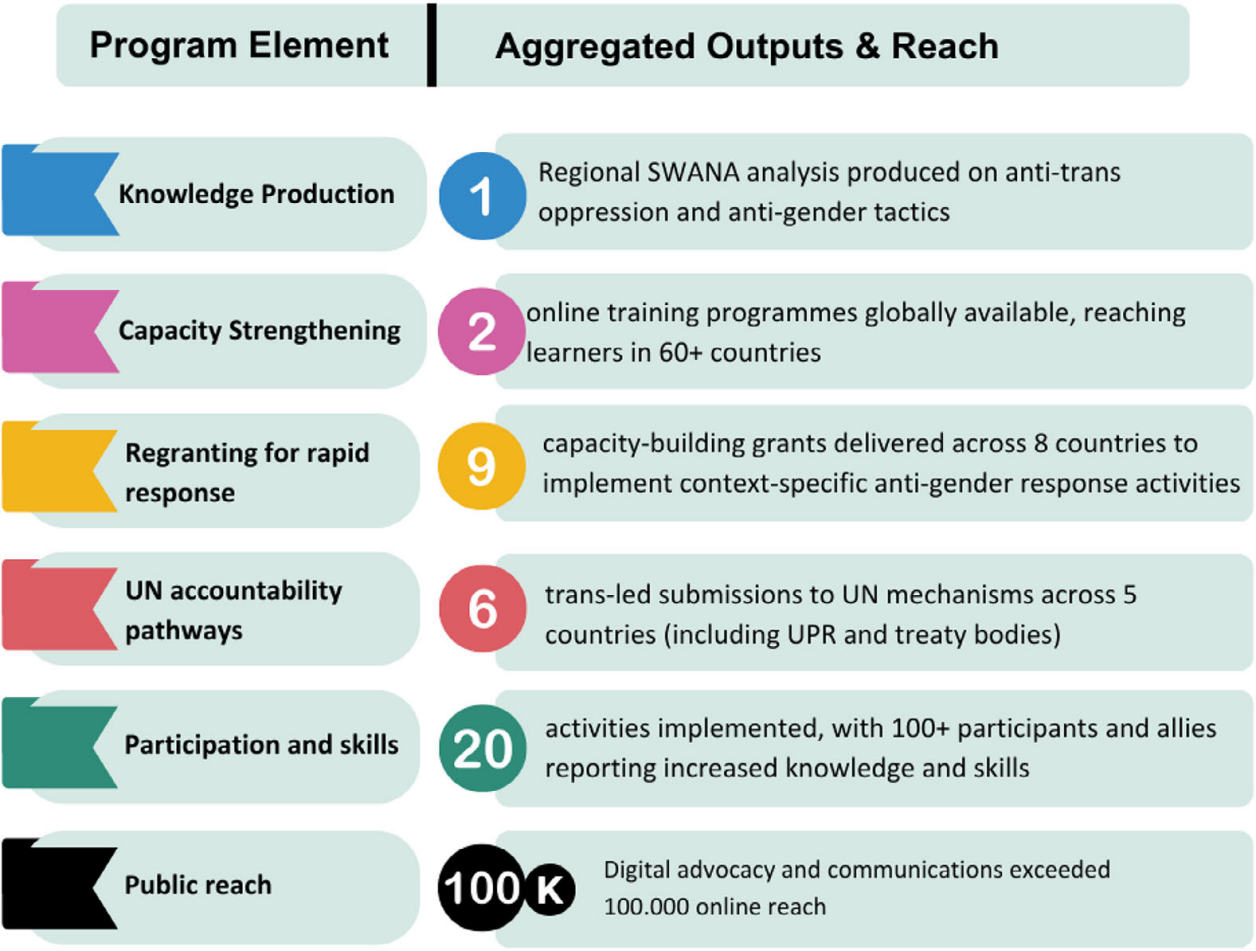
OAF1705

## OAF2502 Impact of Armed Conflict on HIV Testing Services, Positivity Rates and Treatment Services in Sudan 2022–2024

A. Adam ^1^, A. Elrayah^1^, A. Mirzazadeh^1^, M. Elmubarak^2^, K. Awad^2^, E. Ali^3^

^1^UCSF, Institute for Global Health Sciences, San Francisco, United States; ^2^Sudan Ministry of Health, Sudan National HIV Control Program, Port Sudan, the Sudan; ^3^UNAIDS, Port Sudan, the Sudan

**Background**: In Sudan, the outbreak of civil war in April 2023 disrupted the health systems and essential service delivery, including the HIV programme, threatening the progress towards epidemic control. The conflict affected both active battle zones and displacement‐hosting states, potentially reshaping access to HIV prevention, testing and treatment services.

**Methods**: We conducted a quantitative analysis using national HIV programme data from January 2022 to December 2024, covering HIV prevention, testing (VCT, PMTCT, TB/HIV services) and antiretroviral therapy (ART) services. Conflict exposure was derived from the UCDP Georeferenced Event Dataset and IDMC displacement data. Trend analysis and interrupted time series were applied to evaluate changes in service delivery and HIV positivity before and during the conflict period.

**Results**: The conflict led to major disruptions across HIV service delivery platforms. VCT testing volumes declined sharply, with tests among males dropping from 57,724 to 18,977 (78% reduction; *p* < 0.001) and among females from 35,059 to 17,069 (71% reduction; *p* < 0.001). Concurrently, HIV positivity increased significantly, rising from 3.2% to 8.7% among males (+177%; *p* < 0.001) and from 3.5% to 5.1% among females (+81%; *p* < 0.001). PMTCT testing volumes decreased from 65,984 to 49,126 (*p* = 0.001), while positivity doubled from 0.013% to 0.026% (*p* = 0.001). TB/HIV testing volumes also slightly declined: among males from 3489 to 1962, and among females from 1903 to 905. Positivity increased among both sexes, reaching statistical significance among females (0.014%−0.035%; *p* = 0.020). Treatment services experienced an initial sharp decline, with total ART coverage falling by 59% (from 11,073 to 4594), followed by a substantial recovery to 10,393 by the end of 2024 (94% of pre‐conflict levels). New ART initiations dropped by 66% (from 2512 to 46) and recovered to 1948, though they remained below pre‐conflict levels (*p* < 0.001).

**Conclusions**: Armed conflict severely disrupted HIV services in Sudan, with HIV testing most affected. Rising positivity despite reduced testing indicates delayed diagnosis and increased transmission risk. While ART services showed relative resilience and recovery due to its prioritization and redistribution to stable states, testing and prevention remain critically weakened, underscoring the urgent need for decentralized, community‐based testing, expanded prevention outreach and more resilient service delivery models (see Table 1 and 2).

**TABLE 1:** OAF2502 | HIV testing and positivity trends at VCT services before and during conflict in Sudan 2022–2024.

Services	Indicator	2022	2023	2024	Change	*p*‐value
VCT	Male tested	53,893	11,834	10,974	−78%	<0.001
	Male positive (%)	3.23%	8.95%	8.72%	+177%	<0.001
	Female tested	31,958	9206	10,964	−71%	<0.001
	Female positive (%)	3.55%	6.43%	5.11%	+81%	<0.029
PMTCT	Women tested	53,825	24,161	37,124	−55%	<0.001
	Women positive (%)	0.13%	0.16%	0.26%	+0.1%	<0.001
TB/HV	Male tested	3489	2485	1962	−29%	<0.001
	Male positive (%)	2.35%	2.94%	3.92%	+25%	0.474
	Female tested	1903	1577	905	−17%	0.550
	Female positive (%)	1.00%	2.47%	3.43%	+147%	<0.02

**TABLE 2:** OAF2502 | Interrupted time series analysis of conflict impact on adult ART in Sudan (2022–2024).

Indicator	Before conflict (15 months)	April 2023 (conflict onset)	During the conflict (21 months)	Immediate drop recovery status	Recovery status	*p*‐value
New ART Enrolments	2512	46	1948	−66%	No recovery	<0.001
Total on ART	11,073	4594	10,393	−59%	94%	<0.001

## OAF2503 Internal Migration of People Living With HIV During Armed Aggression Against Ukraine: Challenges to HIV Care Retention and Lessons From a Country Experience

S. Riabokon, N. Ryzhenko, A. Bilets, Y. Sobolieva

State Institution “Public Health Center of the Ministry of Health of Ukraine”, Department of HIV Management and Counteraction, Kyiv, Ukraine

**Background**: Russia's full‐scale armed aggression against Ukraine triggered large‐scale internal displacement, posing significant risks to continuity of HIV care. Ukraine has one of the largest HIV epidemics in Eastern Europe, making uninterrupted access to ART during population mobility a critical public health priority. This abstract describes national programme and policy measures implemented to maintain retention in HIV care among internally migrating PLHIV.

**Description**: Since 2022, Ukraine has implemented nationwide adaptive HIV service delivery policies covering all regions and healthcare facilities providing HIV care. The programme targeted adult PLHIV receiving ART and was supported by routine national HIV programme data.

Key interventions:

- rapid scale‐up of MMD;
- accelerated transition to TLD as the preferred first‐line regimen;
- prioritization of fixed‐dose combinations (TLD, TAF/FTC/DTG, ABC/3TC/DTG, TLE);
- regulatory authorization to dispense up to 3 months of ARVs at the moment of HCF or regional change, regardless of prior registration;
- establishment of a central buffer stock of ARVs to mitigate sudden regional shortages;
- continuous operation of a nationwide unified electronic medical information system (IS MSSD), enabling all HIV physicians to access “ART history” and clinical data in real time.

A national visualization of inter‐regional PLHIV movements was developed using IS MSSD data to assess migration patterns among PLHIV on ART.

**Lessons Learned**: The combination of regulatory flexibility, simplified regimens and system‐level preparedness enabled ART continuity despite massive internal migration. MMD and TLD reduced logistical complexity and supported rapid treatment continuation. Emergency 3‐month dispensing minimized immediate treatment interruptions. Central buffer stocks were crucial during periods of critically low ART reserves.

Analysis of inter‐regional migration flows across Ukraine's 24 regions/oblasts (Figure 1) demonstrated that PLHIV movement was predominantly chaotic rather than following stable or predictable geographic patterns with a clear tendency for out‐migration from frontline regions, underscoring the necessity of nationwide, rather than region‐specific, continuity mechanisms.

**Conclusions/Next Steps**: Ukraine's experience shows that resilient HIV care during armed conflict is achievable through flexible regulation, differentiated ART delivery, supply chain buffering and interoperable information systems. These lessons are applicable to other humanitarian settings with high population mobility. Future efforts should focus on strengthen long‐term retention among internally displaced PLHIV.

**FIGURE 1:**
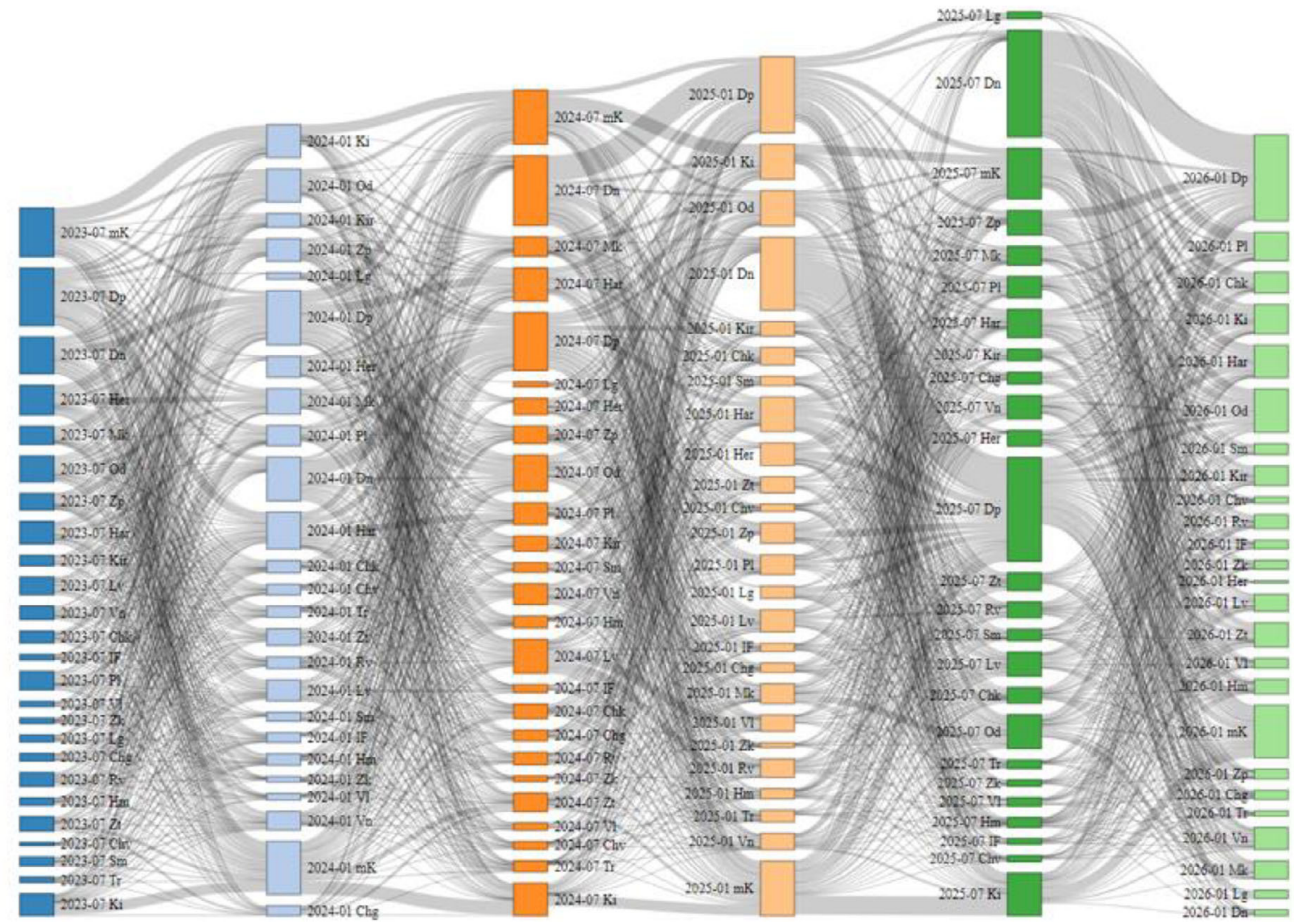
OAF2503 | Inter‐regional migration of PLHIV receiving ART in Ukraine during armed conflict.

## OAF2504 Community Feedback Mechanisms (CFM) to Protect People Who Inject Drugs in Conflict‐Affected Myanmar: Rights‐Based, Peer‐Led Model

P. A. Hein, M. S. Tun, T. Win, M. Thu

Myanmar Positive Group (MPG), Community System Strengthening, Yangon, Myanmar

**Background**: As a global Fast‐Track priority country with high rates of drug use, Myanmar continues to struggle to implement harm reduction programmes for PWID amid political instability. HIV prevalence among PWID is 22.3%. Of 117,000 PWID, only 28% were tested and 14% were on ART in 2024.Global donor reprioritization strategy in 2025 has further disrupted services, creating gaps in prevention and treatment. Led by Myanmar Positive Group (MPG) and Community Network Consortium, CFM operates in 40 townships for PLHIV and KP, with dedicated focus on 14 high‐burden PWID locations including insecure areas. Through partnership with National Drug Users Network, the project strengthened local peer case facilitators to document PWID rights violations and support enabling environment.

**Description**: From January 2024 to June 2025, data in high‐burden areas including Mandalay, Sagaing, Kachin and Shan were collected via MyRights application for documentation across social, legal, healthcare and workplace. Cases are validated and reported to CFM township committees. Case facilitators provide tailored assistance, including emergency aid and psychosocial support, following clear timelines: 2 weeks for social/health cases, 3 months for workplace issues and 6 months for legal cases.

**Lessons Learned**: Among 256 validated cases, (75%) were resolved, (25%) remained unresolved. Most unresolved cases involved physical abuse and extortion targeting PWID arbitrary arrest by police and armed groups. PWID experienced violence/discrimination from family members, barriers in accessing methadone treatment, dismissed/discriminated at work or were forced to leave their homes. The major challenge is advocating with police and armed groups. Legal cases made up 81% of unresolved cases, reveal structural barriers and need for continue advocacy in highest burden areas. Even though social and healthcare‐related issues were manageable through direct support, the legal environment remains the primary barrier to safety and protection.

**Conclusions/Next Steps**: Community leadership and trained case facilitators enabled timely action 75% resolution rate, mostly for social and healthcare cases in fragile setting. However, global donor reprioritization has disrupted harm reduction, legal aid, and referrals services while protracted crisis and criminalization of drug use continues. While peer‐led model is efficient and effective, donor support is needed to sustain community‐led efforts and ensure continued humanitarian support for PWID in fragile setting (see Figures 1 and 2).

**FIGURE 1:**
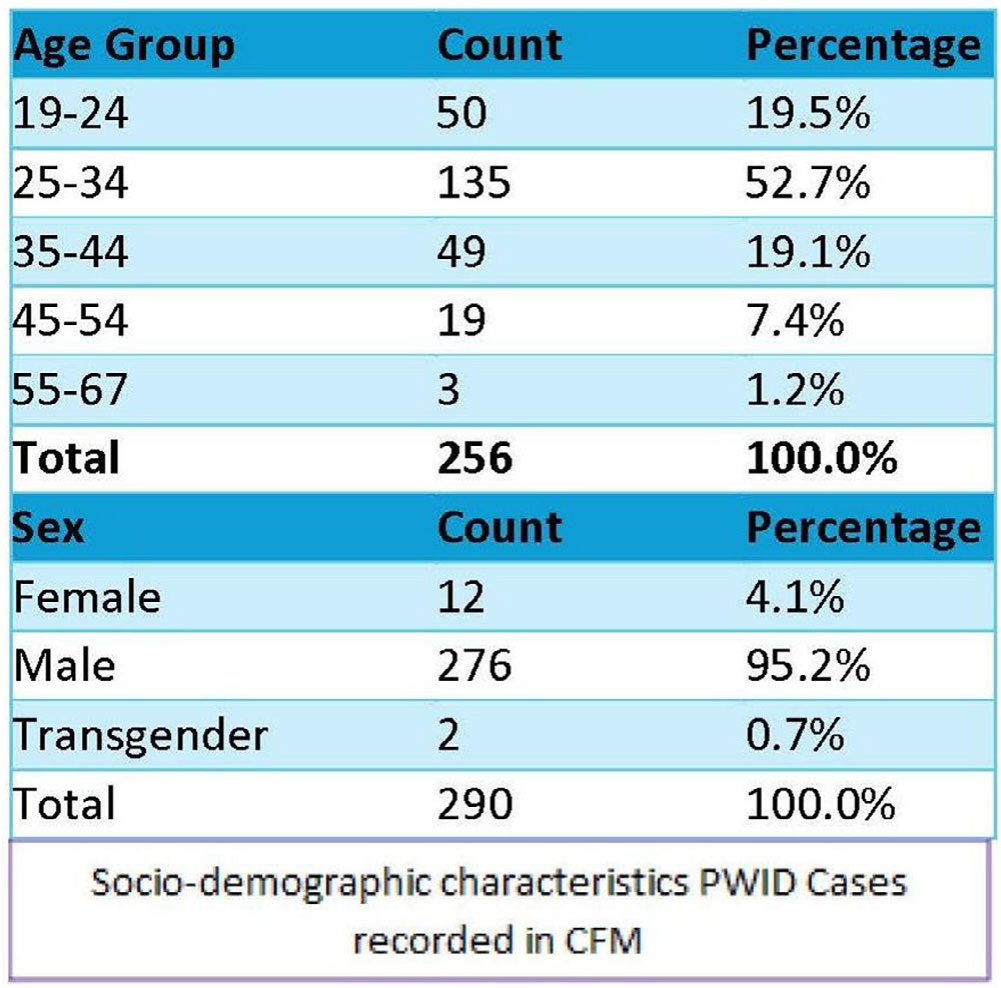
OAF2504

**FIGURE 2:**
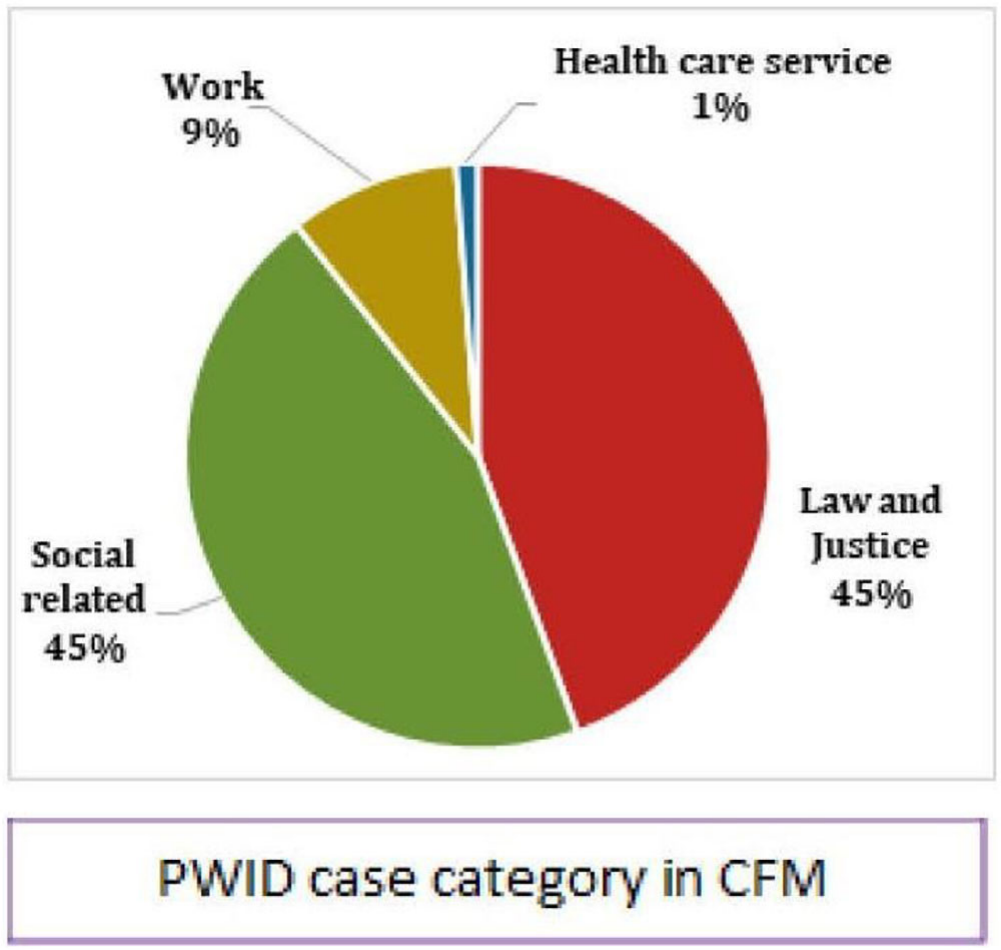
OAF2504

## OAF2505 Lifesaving Law for Undocumented Migrants With HIV and TB in Peru

A. Boccardi ^1^, M. Castillo^2^, P. Giusti^3^, A. Vera Vargas^4^, C. Benites^4^

^1^UNAIDS Country Office Brazil, Brasilia, Brazil; ^2^SIDAVIDA, Lima, Peru; ^3^Instituto de Analisis y Gestion, USAID LHSS Project, Lima, Peru; ^4^Ministry of Health Peru, DPVIH, Lima, Peru

**Background**: Peru hosts more than 1.5 million Venezuelan refugees and migrants, many of whom lack regularized status and are excluded from the national public health insurance system (SIS). This exclusion creates significant barriers to healthcare, exposes migrants to discrimination, and contributes to high burdens of HIV and tuberculosis (TB). Although HIV treatment is officially free regardless of migratory status, pretreatment laboratory tests and the management of complications require out‐of‐pocket payment when individuals are not covered by SIS. These costs delay treatment initiation, increase complications and AIDS‐related mortality. This good practice was first presented at HSR2024 in Nagasaki, Japan; 1 year after the approval of the new law, updated results are presented.

**Description**: In 2023, rising AIDS‐related deaths among Venezuelan migrants spurred the creation of a multisectoral coalition, the Grupo Impulsor. Civil society, international agencies, the Ombudsman's Office, academia, the Ministry of Health and other stakeholders collaborated to draft legislation enabling migrants with HIV and/or TB to be included in SIS. The advocacy strategy engaged legislators and officials from the Ministries of Health and Finance and was supported by a cost–benefit analysis demonstrating the fiscal advantages of early treatment. Law Bill 5253 was introduced in June 2023; Congress approved it in October 2024, and it was published on 5 November 2024. By 23 January 2026, 197 Venezuelan migrants had been enrolled in SIS—54 with TB and 143 living with HIV—while antiretroviral treatment coverage increased by 633 people. Civil society monitoring mechanisms led by GIVAR addressed 50 complaints in 2025 related to medication shortages and access barriers.

**Lessons Learned**: An economic analysis translating health benefits into fiscal terms, and the involvement of oversight institutions were required to sensitize legislators. Addressing xenophobia required public sensitization and direct policymaker engagement, while implementation demanded flexible responses to shifting migration policies and continued civil society‐led monitoring and communication.

**Conclusions/Next Steps**: Peru's legislative achievement demonstrates how rights‐based, evidence‐informed advocacy can expand health coverage for marginalized migrants while generating public health and economic benefits. The modest number of people registered in 2025 underscores the need for continued monitoring and strengthened outreach to affected communities and health services.

## OAF3202 HIV Criminalization as Structural Violence: Science, Geopolitics and Community‐Led Pathways to Justice

E. J. Bernard, S. V. Villanueva, S. Beaumont, A. Symington

HIV Justice Network, Amsterdam, the Netherlands

**Background**: Despite four decades of scientific progress and global commitments to end AIDS, laws and practices that punish people living with HIV for non‐disclosure, perceived exposure or unintentional transmission persist despite the fact that many criminalized acts pose no or negligible risk, and that punitive approaches undermine public health and human rights. This persistence reflects not evidence failure, but structural violence embedded in legal, political and funding systems.

**Methods**: We performed a longitudinal analysis of our *Global HIV Criminalization Database* with comparative review of legislative reforms, strategic litigation outcomes, and prosecutorial and judicial guidance. Updated data to June 2026 will be presented, with disaggregation by demographics where possible. Findings are contextualized through community‐led case monitoring, advocacy experience, and alignment with international scientific and human rights standards as synthesized in HJN's soon‐to‐be‐published *Guidance on Good Practices in HIV Decriminalization*, supported by UNAIDS.

**Results**: HIV criminalization has not reduced HIV transmission. Instead, it deters testing, delays treatment, entrenches stigma, and disproportionately harms women, migrants and other key populations. While recent reforms demonstrate that change is feasible, progress is uneven and fragile. Reform efforts increasingly operate within a hostile environment marked by a coordinated backlash against science, gender equality, sexual and reproductive rights, and civil society space. And yet durable progress has emerged through three interconnected pathways:

1. legislative repeal or modernization of HIV‐specific offences;
2. strategic litigation and authoritative guidance aligning criminal law with contemporary science; and
3. sustained, community‐led advocacy and accountability.

Reforms that explicitly exclude zero‐risk conduct, prohibit criminalization of vertical transmission and constrain prosecutorial discretion are associated with reduced enforcement. Conversely, reforms that rely narrowly on biomedical thresholds or omit safeguards risk displacement of criminalization into general criminal or public health law.

**Conclusions**: HIV criminalization persists as a form of structural violence reinforced by geopolitical power, stigma and shrinking civic space. Ending it requires more than technical legal reform: it demands political resistance to anti‐science agendas, reinvestment in rights‐based multilateralism and centring community power. In a constrained and polarized global context, aligning law with science is both a public health necessity and a justice imperative.

## OAF3203 Policed Intimacy: Lived Experiences of HIV Criminalization Among People Living With HIV in Aotearoa, New Zealand

B. M. Hollingshead ^1,2,3^, R. Olin German^4^, P. Hanl^4^, M. Fisher^5^, J. Bruning^1^, M. Stewart^2^

^1^Positive Women Inc., Auckland, New Zealand; ^2^Toitū te Ao, Rotorua, New Zealand; ^3^University of Auckland, Auckland, New Zealand; ^4^Burnett Foundation Aotearoa, Auckland, New Zealand; ^5^Body Positive Inc., Auckland, New Zealand

**Background**: Despite major advances in HIV treatment and prevention, HIV criminalization remains embedded in many legal systems worldwide. In Aotearoa, New Zealand (NZ), people living with HIV may be prosecuted under general criminal or public health law for non‐disclosure, exposure or transmission, even with zero transmission risk. Evidence remains limited on how these laws are experienced by people living with HIV in NZ. This study explores how HIV criminalization is lived, interpreted and navigated, particularly its emotional, relational and behavioural consequences.

**Methods**: Qualitative data were drawn from open‐text responses in a national, anonymous online survey of adults living with HIV in 2025. Responses were analysed using reflexive thematic analysis, informed by a sociological framework. Analysis focused on how participants understood responsibility, risk, disclosure and intimacy in the context of criminalization.

**Results**: Participants overwhelmingly rejected the framing of HIV transmission as a criminal matter, emphasizing that HIV is a health issue best managed through care, education and support. Mistrust of police involvement was a recurring theme, and many participants described law enforcement as stigmatizing, punitive and poorly aligned with contemporary HIV science. In contrast, healthcare providers and community organizations were more trusted, although some participants expressed concern about unnecessary intervention in contexts of zero transmission risk.

Criminalization was experienced less as a legal event and more as a persistent emotional and psychological presence. Many participants described anxiety about disclosure, fear of legal consequences and anticipation of rejection or stigma, which shaped sexual and relational decision‐making. Strategies for navigating these pressures included abstinence, limiting sexual encounters to other people living with HIV, preferring anonymous encounters or relying on condoms (as a form of legal compliance). Confidence in U = U informed resistance to disclosure expectations for some, while others disclosed consistently to avoid legal risk despite emotional costs. Participants also described missed relationships, diminished intimacy and self‐imposed isolation as consequences of living under criminalization.

**Conclusions**: These findings demonstrate that HIV criminalization operates as a lived regime of governance reshaping intimacy, disclosure and self‐worth, even in the absence of prosecution. Perceived threat of criminalization constrains autonomy, produces anxiety and contributes to relational harm.

## OAF3204 HIV Criminalization Law Enforcement Associated With Increased Stigma Among Sexual Minority Men Living With HIV in the United States

D. Etwaru ^1^, H. Schmidt^1^, M. J. Heise^1^, K. Sassaman^1^, S. Mahuvakar^1^, A. D'Angelo^2^, T. B. Neilands^1^, D. T. Duncan^3^, K. J. Horvath^4^, S. Hirshfield^5^, R. Williams^6^, M. O. Johnson^1^, C. Grov^2^, A. Carrico^7^, M. Gandhi^1^, M. Spinelli^1^

^1^University of California, San Francisco, San Francisco, United States; ^2^CUNY School of Public Health, New York, United States; ^3^Columbia University, New York, United States; ^4^SDSU/University of California, San Diego, San Diego, United States; ^5^SUNY Downstate, Brooklyn, United States; ^6^University of Miami, Miami, United States; ^7^Florida International University, Miami, United States

**Background**: In the United States, HIV criminalization statutes prohibit otherwise lawful conduct or impose enhanced penalties for prohibited conduct among persons living with HIV (PLWH). There is one federal HIV criminalization law, and over half of U.S. states (28 vs. 22 states) have individual criminalization laws. Most of these statutes were enacted during the 1980s−1990s AIDS epidemic, reflecting limited transmission understanding, absent effective treatment and intersecting stigmas against groups most affected by HIV. We examined the associations of these criminalization laws with viral suppression and self‐reported HIV stigma in a national U.S. sample.

**Methods**: Data on HIV criminalization laws as of 2025 and number of HIV‐related arrests were collected from The Williams Institute public case reports (published between 2015 and 2024). Statutes commonly penalized non‐disclosure, potential HIV exposure through sex or blood, sex work, non‐consensual sexual contact and tissue donation. Linear and logistic regressions examined associations between state‐level criminalization laws and county‐level HIV‐related arrest frequencies and outcomes among sexual minority men living with HIV from the American Remote Contact HIV Epidemiology Study (ARCHES) recruited October 2023−2024 (*n* = 1000).

**Results**: Among 1039 counties in states with statutes, 66% reported zero arrests over the past decade; where arrests occurred, the median was 1 (IQR 1–4), maximum 643 (Los Angeles County, California; Figure 1). Of the 1000 participants in the ARCHES cohort, 549 lived in states with criminalization laws; 378 of these lived in states with ≥1 arrest in the past 10 years. State‐level laws and county‐level arrest counts did not explain viral suppression (*ps*>0.4) in ARCHES, nor did the presence of laws explain HIV‐related stigma (*p* = 0.3). However, participants in states with more HIV‐related arrests reported greater HIV‐related stigma (*β* = 0.11, 95% CI 0.01–0.21, *p* = 0.030), adjusting for age and race.

**Conclusions**: U.S. federal and state HIV‐criminalization laws continue despite improved understanding of HIV transmission and treatment‐as‐prevention. Enforcement of these outdated laws can greatly affect the quality of life experienced by people living with HIV. This study highlights the need for legislative reform to repeal HIV criminalization laws to address the stigma that they may impart, possibly compromising the goals of the U.S. Ending the HIV Epidemic Initiative.

**FIGURE 1:**
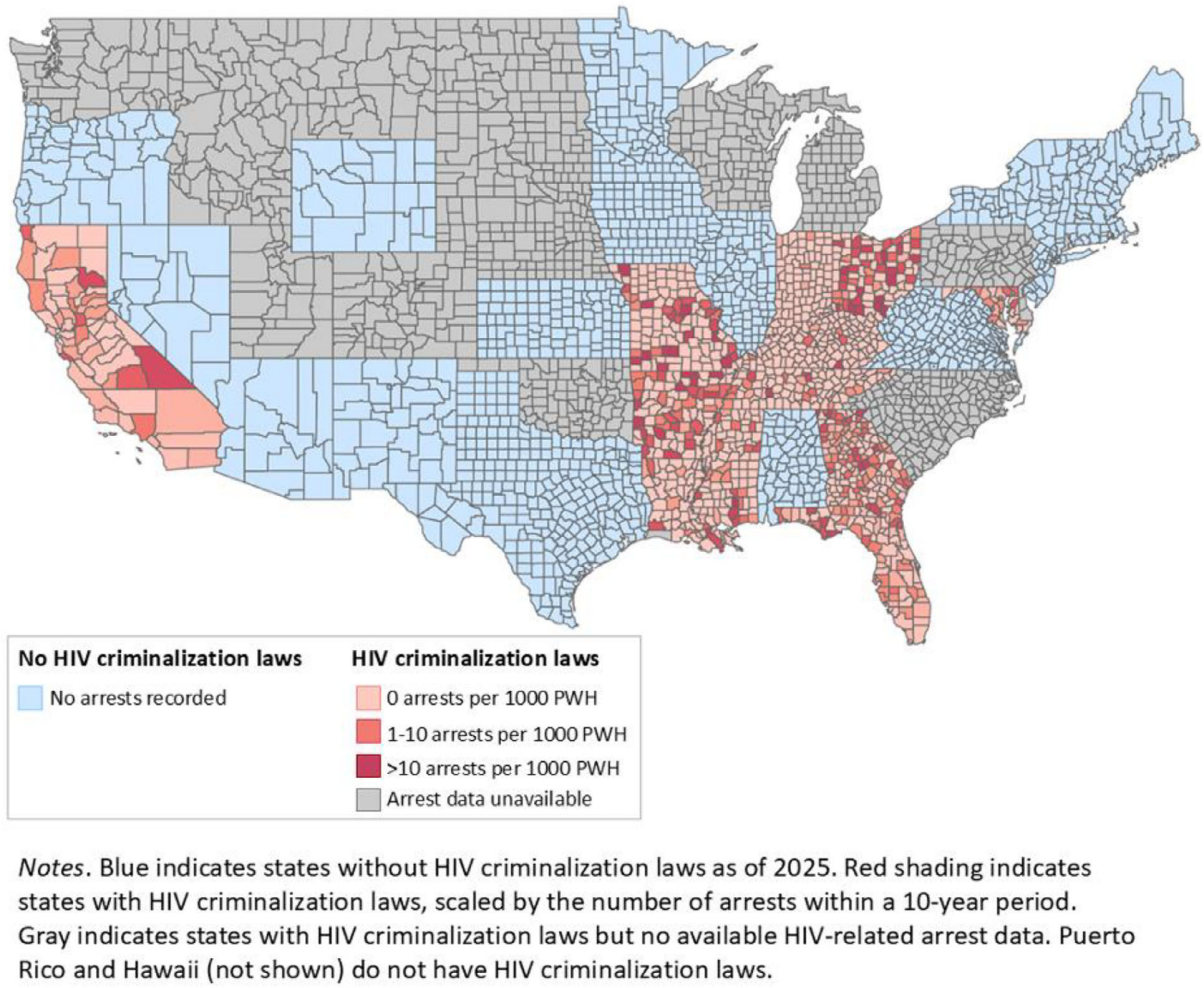
OAF3204

## OAF3205 From Headlines to Health Outcomes: Engaging Journalists to Counter HIV Stigma and Misinformation in Côte d'Ivoire and Cameroon

J. Messinga Nkonga ^1^, E. D. Guemne Kapche^1^, R. L. Guimessa Mezation^1^, D. C. E. Yao^2,3^, Team Medi +

^1^Fierté Afrique Francophone, Yaoundé, Cameroon; ^2^Fierté Afrique Francophone, Abidjan, Côte d'Ivoire; ^3^Global Black Gay Men Connect, Washington, United States

**Background**: In francophone Africa, the response to HIV remains hampered by the stigmatization of key populations and by inaccurate, sensationalist or moralistic media narratives, which fuel misinformation, reinforce barriers to access to testing, prevention and care, and undermine supportive environments based on human rights.

It is in this context that the project Media for Equality, Dignity and Inclusion in the Face of HIV in Francophone Africa (MEDI+) was implemented by Fiertés Afrique Francophone (FAF) with the support of UNAIDS to mobilize the media as a lever for public health and reducing HIV‐related stigma, integrating Sexual Orientation, Gender Identity/Expression and Sexual Characteristics (SOGIESC) and human rights issues.

**Description**: MEDI+ was rolled out between October and December 2025 in two pilot countries: Côte d'Ivoire (Abidjan) and Cameroon (Yaoundé). The programme targeted nine media professionals (five in Côte d'Ivoire and four in Cameroon) from television, radio, print media, online media and social media. The interventions combined: (i) a diagnostic study on media knowledge, attitudes and practices; (ii) the production of a simplified practical kit and contextualized training modules (HIV, U = U, combination prevention, PrEP/PEP, SOGIESC, human rights, journalistic ethics) based on existing reference materials; (iii) two national capacity‐building workshops (one in Cameroon and one in Côte d'ivoire); (iv) personalized mentoring to support each journalist in the production of a piece, structured by a framework note validated jointly with UNAIDS and supported by financial assistance.

**Lessons Learned**: The project improved understanding of key concepts (HIV ≠ AIDS, U = U, prevention) and reinforced the adoption of non‐stigmatizing media practices (language, angles, management of sensitive data). A total of 14 media productions were produced (instead of the nine planned), with four journalists producing additional content, illustrating a high level of ownership. Experience shows that training is more effective when combined with editorial mentoring, rigorous framing and production support.

**Conclusions/Next Steps**: MEDI+ demonstrates that the media can become key allies in the response to HIV when they are equipped and supported with a human rights‐based approach. This approach should be expanded to other Francophone countries, institutionalized with national actors, and long‐term monitored to maximize the impact on stigma and access to services.

## OAF3502 Impact of PEPFAR Funding Cuts on HIV Programme Performance and Outcomes in Sub‐Saharan Africa: A Systematic Review

J. S. Luke ^1^, S. Konah^1^

^1^Pathfinder International, Nairobi, Kenya

**Background**: The U.S. President's Emergency Plan for AIDS Relief (PEPFAR) has been central to HIV epidemic control in sub‐Saharan Africa (SSA). In January 2025, an abrupt freeze of U.S. foreign assistance disrupted HIV programmes across the region, raising concerns about service continuity and equity. We synthesized emerging empirical and modelling evidence to assess the impact of PEPFAR funding cuts on HIV programme performance and outcomes in SSA.

**Methods**: We conducted a systematic review following PRISMA 2020 guidelines (PROSPERO registration ID CRD420251239807). We searched PubMed, Embase, Google Scholar and grey literature published in 2025 that reported empirical or modelled impacts of PEPFAR funding disruptions in SSA. Modelling studies and grey literature were appraised using ISPOR‐SMDM and AACODS checklist, respectively. Due to heterogeneity in designs and outcomes, findings were synthesized narratively with descriptive summaries.

**Results**: Twenty‐one studies and reports met inclusion criteria. Empirical evidence demonstrated rapid, interlinked disruptions across all six WHO health‐system building blocks. The funding freeze resulted in the loss of more than 8000 PEPFAR‐supported health workers in South Africa and placed an estimated 16,700 positions at risk in Zimbabwe, with community‐based cadres most affected. Prevention services experienced the largest declines: PrEP initiations fell by 31%−64%, voluntary medical male circumcision by 65%−88% and condom distribution by over 50% in several countries. DREAMS programmes were suspended in at least 10 countries, disproportionately affecting adolescent girls and young women. ART initiation declined by 22%−30%, while viral load testing fell by 16%−68%. Modelling consistently projected that sustained cuts would increase HIV incidence (2%−78%) and mortality (4%−50%), with the largest relative impacts on children and key populations, and the greatest absolute mortality burden among women (see Figures 1 and Table 1).

**FIGURE 1:**
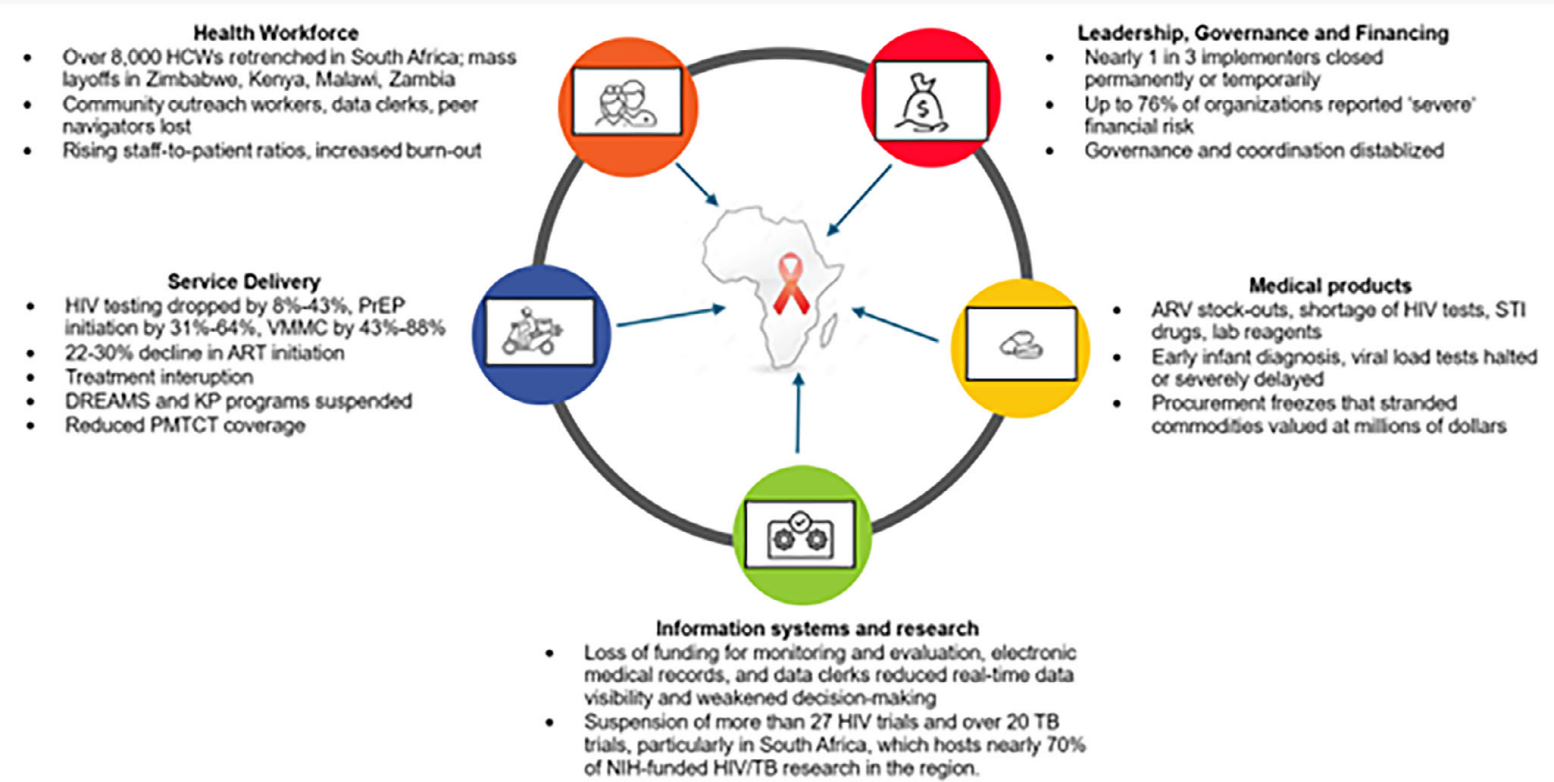
OAF3502 | Conceptual framework: pathways from PEPFAR funding cuts to health system and HIV outcomes in SSA.

**TABLE 1:**
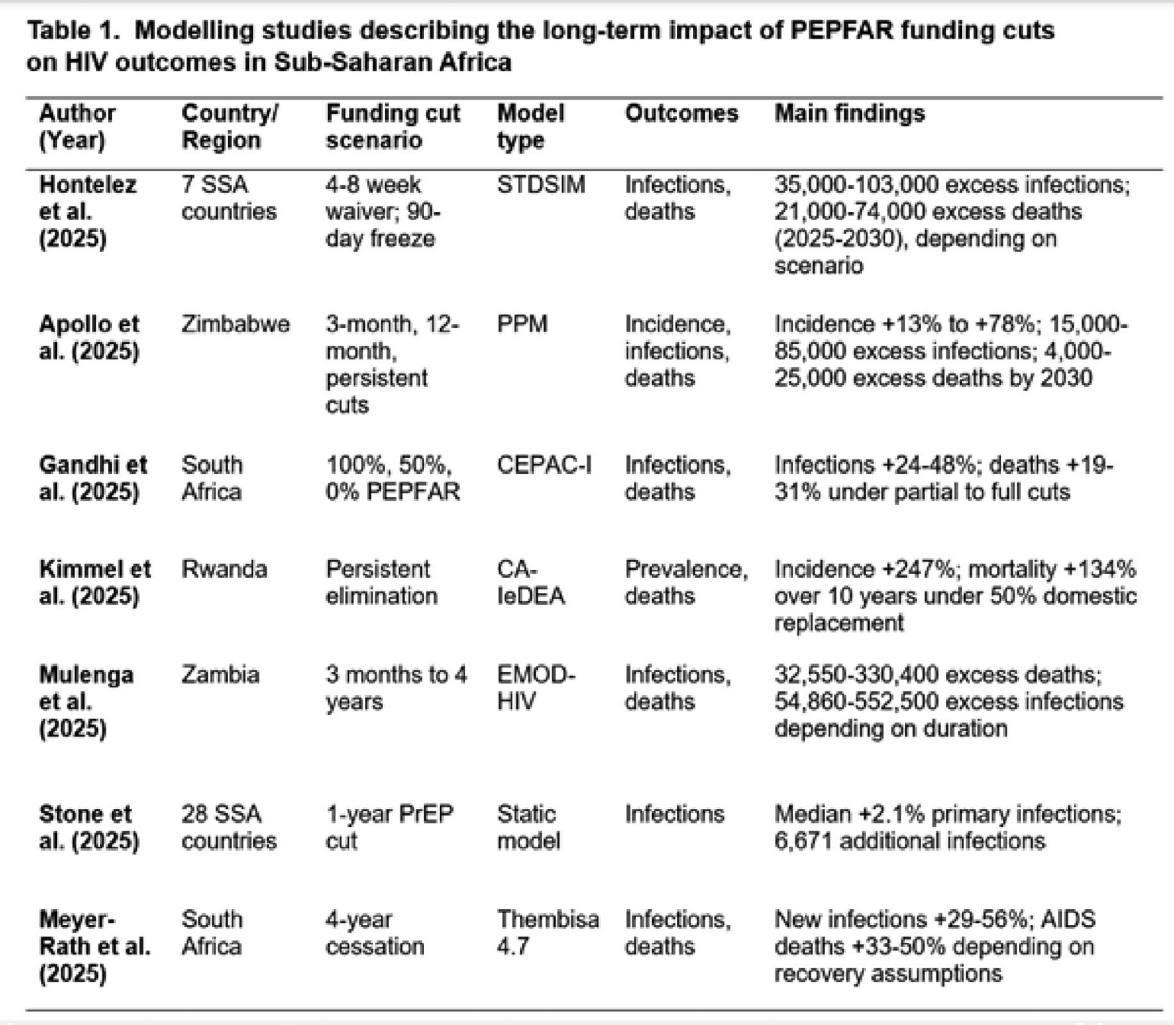
OAF3502 | Modelling studies describing the long‐term impact of PEPFAR funding cuts on HIV outcomes in sub‐Saharan Africa.

**Conclusions**: The 2025 PEPFAR funding freeze was associated with immediate, inequitable HIV service disruptions across SSA health systems. Convergent evidence signals a high risk of reversing epidemic gains. Protecting progress requires urgent, equity‐centred actions: bridge financing, safeguarding prevention and KP services, and implementing managed transition plans.

## OAF3503 Lessons From the Global Fund GC7 Reprioritization Process: Implications for Community‐Led HIV Responses

R. Montgomery, I. Ogunkola, A. Basenko, O. Szubert

International Network of People Who Use Drugs, London, United Kingdom

**Background**: In 2025, the Global Fund implemented a mid‐cycle reprioritization of Grant Cycle 7 (GC7) investments in response to major global financial and political shocks, including sharp reductions in external development assistance. The reprioritization process sought to protect lifesaving HIV, TB and malaria services under reduced funding envelopes. However, concerns emerged regarding its implications for community‐led, rights‐based HIV responses for key populations. This analysis documents community experiences of the reprioritization process to inform more equitable approaches as preparations for Grant Cycle 8 (GC8) begin.

**Description**: This community‐led analysis was conducted by global key population networks and draws on experiences from multiple Global Fund–supported countries during the GC7 mid‐cycle reprioritization period in 2025. The focus is on HIV grants affecting key populations, including people who use drugs, sex workers, trans and gender diverse people, and gay, bisexual and other men who have sex with men. Data sources included structured community feedback, surveys, country‐level consultations, technical assistance engagements and a multi‐country validation process. The analysis examined decision‐making processes, community engagement, access to information, implementation arrangements and the impact of accelerated service integration on community‐led responses.

**Lessons Learned**: The reprioritization process exposed persistent structural weaknesses within country funding models that disproportionately affect key population‐led responses. Human rights programming and community‐led interventions were frequently among the first activities paused, deferred or reduced. Limited transparency, compressed timelines, language barriers, and restricted access to financial and programmatic information constrained meaningful community engagement. Poorly defined and accelerated integration created uncertainty and risk, particularly in criminalized and politically hostile settings, where reliance on government‐run services undermined trust, confidentiality and access. Where targeted technical assistance and strong community mobilization were present, some key population priorities were retained or reinstated.

**Conclusions/Next Steps**: The GC7 reprioritization process functioned as an early warning signal for risks that may intensify under GC8 if structural safeguards are not strengthened. Protecting community‐led HIV responses requires clear guidance on integration, transparent decision‐making, sustained community engagement across the grant lifecycle and funding mechanisms that enable key population leadership. These lessons are directly relevant to future Global Fund funding cycles and broader HIV financing reforms in contexts of shrinking resources and political uncertainty.

## OAF3504 No Access, No Support: The Toll of Funding Suspension on HIV Prevention Care Among Trans Women in Janakpur, Nepal

G. N. Baguso ^1,2^, B. Suprasert^2^, D. Moktan^3^, S. Banik^4^, S. Sharma^5^, M. Dhakal^5^, E. C. Wilson^2,6^

^1^University of California, San Francisco, Institute of Health & Aging, San Francisco, United States; ^2^San Francisco Department of Public Health, Center for Public Health Research, San Francisco, United States; ^3^Independent Consultant, Los Angeles, United States; ^4^St. Bonaventure University, New York, United States; ^5^Blue Diamond, Kathmandu, Nepal; ^6^University of California, San Francisco, Department of Epidemiology and Biostatistics, San Francisco, United States

**Background**: Nepal witnessed a 94% reduction in new HIV cases over the last two decades, in great part due to HIV prevention support by USAID. With the 2019 implementation of PrEP, Nepal was on track to end their HIV epidemic by 2030. The elimination of USAID funding as of February 2025 threatens to reverse this progress, and populations most affected in Nepal's concentrated epidemic may be the most impacted. PrEP is no longer available, and community‐based HIV outreach and HIV testing may no longer reach key populations. We present data collected in 2025 on the immediate impact of USAID‐funded service suspension on transgender women in a rural region of Nepal.

**Methods**: This analysis presents data on changes in health and access to HIV prevention and care gathered from 28 transgender women in Janakpur, Nepal. Data were collected in October 2025, which was 8 months after the suspension of USAID funding. Descriptive statistics were used to summarize participant demographics and changes in access to HIV prevention.

**Results**: Most participants were under 40 years old (86%) and identified as transgender woman (36%) or Hijra (32%). The majority (68%) earned less than 20,000 NPR/month ($138.49 USD or 744.46 BRL) through informal sources of income such as dancing or singing (79%). Approximately 28.5% of participants were living with HIV; 63% were aware of PrEP, and 42% had taken PrEP previously. Prior to the suspension of USAID funding, 86% accessed USAID‐funded HIV prevention services. After the suspension of USAID, no participants had access to PrEP, 68% could no longer access condoms and lubricant, 29% could no longer access confidential HIV testing and 50% no longer had a clinic for healthcare services.

**Conclusions**: The dismantling of USAID‐funded services resulted in total elimination of access to PrEP, and severely reduced access to other types of HIV prevention for almost all participants in our study. Populations like transgender women are key to addressing the HIV epidemic globally and in Nepal. Cessation of funding for HIV prevention will lead to drastic and unnecessary increases in new HIV cases among transgender women in Nepal.

## OAF3505 Impact of Funding Interruptions on the HIV Response in Maputo City: An Operational and Statistical Evidence (2023–2025)

A. P. Magaia de Abreu ^1^, E. Cumaquela^2^, E. Chilundo^1^, T. Nhanombe^1^

^1^Maputo Municipal Council, Health and Quality of Life, Maputo City, Mozambique; ^2^Conselho Municipal de Maputo, Health and Quality of Life, Maputo City, Mozambique

**Background**: This study assessed the operational impact of funding interruptions on HIV service delivery in Maputo City. Between January and February 2025, the implementing partners temporarily suspended support to municipal health facilities, disrupting HIV testing, diagnosis, linkage and ART initiation. The hypothesis was that these funding cuts produced a statistically significant decline in key HIV programme indicators compared with the same period in 2023 and 2024.

**Methods**: A retrospective, comparative analysis was conducted using routine programme data from January 2023 to November 2025, focusing on trends for January–February 2023–2025. Indicators included total HIV tests, positive diagnoses, ART initiations, positivity and linkage. Monthly aggregated data were analysed for temporal trends and assessed statistically using chi‐square tests (χ^2^) to determine differences between years. The study population comprised all individuals tested for HIV in municipal health facilities.

**Results**: A marked decline followed the funding interruptions. In January 2023, 32,508 individuals were tested, with 1359 positives (4%) and 1441 ART initiations; in January 2024, 31,888 were tested, 1209 were positive (4%) and 1058 initiated ART. In contrast, January 2025 recorded only 17,256 tested, 607 positives and 563 ART initiations declines of 46%, 50% and 47%, respectively. February showed similar declines: 32,686 tested, 1401 positives and 1432 ART initiations (2023); 36,627, 1403 and 1267 (2024); versus 24,789, 944 and 890 (2025). Chi‐square analysis confirmed significant differences across years: total tests (χ^2^ = 124.55; *p* < 10^−28^), positives (χ^2^ = 628.30; *p* ≈ 10^−137^) and ART initiations (χ^2^ = 430.16; *p* ≈ 10^−94^), indicating these declines were not random.

**Conclusions**: Funding interruptions had immediate, severe effects on HIV service delivery, reducing testing coverage, case detection and timely ART initiation. Strengthening contingency financing, monitoring systems and implementing a structured recovery plan (Catch‐Up HIV 2025–2026) are essential to maintain epidemic control and prevent reversal of programme gains.

## OAE0505 Cost‐Effectiveness of Implementing Dual HIV/Syphilis and Triplex HIV/Syphilis/HBV Rapid Diagnostic Testing Among Pregnant Women in Kenya and South Africa

D. Coomes^1^, A. Malhotra^1^, Z. W. Y. Bo^1^, V. Tshivhase^2^, A. Monroe‐Wise^3^, S. Bajis^3^, N. Luhmann^4^, T. Chidarikire^5^, C. Johnson ^3^, M. Barr‐DiChiara^3^, M. Sharma^1^, A. Drake^1^

^1^University of Washington, Seattle, United States; ^2^CHAI, Pretoria, South Africa; ^3^World Health Organization, Geneva, Switzerland; ^4^Regional Health Agency, Paris, France; ^5^World Health Organization, Pretoria, South Africa

**Background**: Coverage of HIV, syphilis, and especially hepatitis B virus (HBV) testing among pregnant in Africa remains suboptimal. Multiplex rapid diagnostic tests (RDTs) could improve coverage by simplifying testing algorithms, thereby reducing vertical transmission and maternal and infant morbidity and mortality. We evaluated the cost‐effectiveness of multiplex testing for HIV, syphilis and HBV during pregnancy in Kenya and South Africa.

**Methods**: We developed Markov models for HIV, syphilis and HBV in pregnancy for Kenya and South Africa. Model parameters and costs were obtained from the literature, in‐country data and expert opinion. Multiplex test performance characteristics were based on commercially available products. We compared three tests (RDTs for HIV and hepatitis B surface antigen [HBsA], lab tests for syphilis) (scenario1) with a dual HIV/syphilis RDT plus an HBsAg RDT (2) and with a single triplex RDT (3). We assumed implementation of dual and triplex tests would increase testing coverage of covered infections to levels observed for HIV RDT. We calculated incremental cost‐effectiveness ratios (ICERs) and used a cost‐effectiveness threshold of $500/disability adjusted life year (DALY) averted.

**Results**: In both countries, triplex testing provided the largest health benefits of the scenarios modelled. In Kenya, dual HIV/syphilis + HBsAg testing (scenario 2) was cost‐effective versus single tests (scenario 1) (ICER $33/DALY averted), and triplex (scenario 3) was cost‐effective versus dual HIV/syphilis + HBsAg (ICER $196/DALY). Triplex increased syphilis and HBV coverage from 73% and 31% to 80% for both. Dual HIV/syphilis + HBsAg and triplex was modelled to avert 19% of congenital syphilis, while multiplex averted 32% of chronic HBV in infants. In South Africa, dual HIV/syphilis + HBsAg testing was cost‐effective versus single tests ($10/DALY averted), and triplex was cost‐effective versus dual HIV/syphilis + HBsAg ($215/DALY averted). Triplex averted 27% of congenital syphilis, and averted 42% of chronic HBV in infants.

**Conclusions**: Multiplex testing for HIV, syphilis and HBV in pregnancy can cost‐effectively prevent additional congenital syphilis and chronic HBV in South Africa and Kenya. Triplex RDTs could especially close HBV testing gaps and accelerate triple elimination of mother‐to‐child transmission of HIV, syphilis and HBV (Tables 1 and 2).

**TABLE 1:**
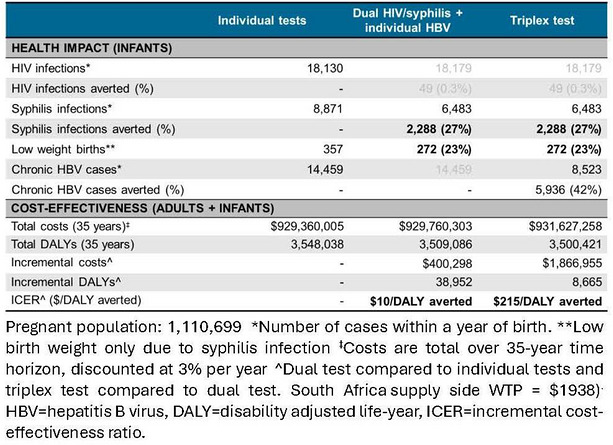
OAE0505 | Health and economic impact of dual HIV syphilis and syphilis rapid diagnostic tests plus hepatitis B surface antigen tests and triplex rapid diagnostic tests in South Africa.

**TABLE 2:**
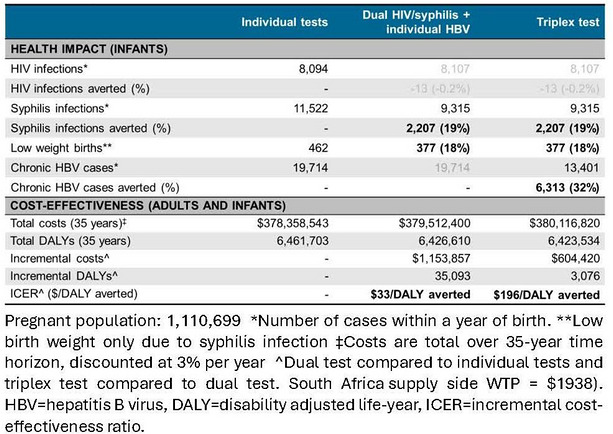
OAE0505 | Health and economic impact of dual HIV syphilis and syphilis rapid diagnostic tests plus hepatitis B surface antigen tests and triplex rapid diagnostic tests in Kenya.

## Late Breaking Abstracts

## OAA1506LB Sustained HIV‐1 Remission Following CCR5 Delta32/Delta32 Allogeneic Hematopoietic Stem Cell Transplantation: The Kansas City Patient

W. El Atrouni ^1^, S. Abhyankar^2^, L. A. Clough^1^, K. Snyder^1^, S. Brandenburg^1^, R. Balusu^2^, R. Soder^2^, K. Pfannenstiel^2^, B. Thedinger^3^, A. D. Badley^4^, J. P. McGuirk^2^, A. Singh^2^, T. W. Chun^5^

^1^The University of Kansas Medical Center, Internal Medicine, Infectious Diseases, Kansas City, United States; ^2^The University of Kansas Medical Center, Internal Medicine, Hematologic Malignancies and Cellular Therapeutics, Kansas City, United States; ^3^KCCare Health Center, Kansas City, United States; ^4^Mayo Clinic College of Medicine, Internal Medicine, Molecular Medicine, Infectious Diseases, Rochester, United States; ^5^National Institutes of Health, National Institute of Allergy and Infectious Diseases, Bethesda, United States

**Background**: HIV‐1 remission following allogeneic hematopoietic stem cell transplantation (alloHSCT) for haematological cancers has been reported in a limited number of patients. However, many questions remain regarding the critical factors needed for HIV reservoir elimination, limiting the development of scalable curative strategies.

**Methods**: We evaluated the impact of alloHSCT on HIV‐1 persistence in a 21‐year‐old male following analytical treatment interruption (ATI). The participant underwent alloHSCT for undifferentiated acute myeloid leukaemia from an HLA‐matched (10/10) unrelated female donor who was homozygous for CCR5 delta32/delta32. Plasma viraemia and CD4+ T cells were measured every 2 weeks following ATI. Levels of HIV‐1 reservoirs, including cells carrying replication‐competent virus and intact proviral DNA, as well as HIV‐specific T cell responses, and the presence of HIV‐specific antibodies, were examined longitudinally prior to and following ATI. Donor chimerism, CCR5 expression and the presence of antiretroviral drug levels were also evaluated.

**Results**: Prior to antiretroviral therapy (ART), plasma viraemia and the nadir CD4+ T cells were 2,030,000 copies/mL and 23 cells/µL (4%), respectively, in 2018. HIV‐1 antibodies, RNA and DNA were detected pre‐alloHSCT with predicted R5 DNA viral tropism. AlloHSCT, which occurred in October 2020 following myeloablative conditioning (busulfan/cyclophosphamide), led to full donor chimerism and remission. Post‐transplant course was complicated by poor graft function treated with selected CD34+ stem cell boost, and stage I acute graft‐versus‐host disease of the gastrointestinal tract treated with steroids. Post‐transplant, CCR5 surface expression in blood cells was undetectable. The patient initiated ATI in February 2025. HIV‐1 plasma viraemia remained undetectable (LOQ 20 copies/mL) for 14 months following ATI. Repeated measurements, conducted four times after transplant, including twice after ATI, showed no detectable intact HIV‐1 proviral DNA or replication‐competent virus in his blood. Antiretrovirals remained undetectable throughout the ATI period although on demand oral pre‐exposure prophylaxis was permitted and used once. HIV‐1‐specific antibodies and T cell responses to Gag and Env remained detectable prior to and following ATI.

**Conclusions**: We report HIV‐1 remission sustained for 14 months following ATI in our patient post alloHSCT from a CCR5 delta32/delta32 donor. Ongoing monitoring will further determine HIV reservoir eradication (see Figure 1).

**FIGURE 1:**
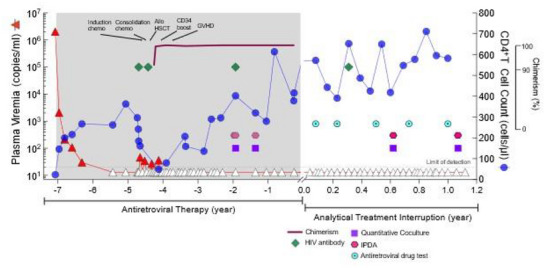
OAA1506LB | Longitudinal follow up from 2018 to 2026. Plasma HIV‐1 RNA levels (red line; left y‐axis) and CD4+ T cell counts (blue line; right y‐axis) from the time of HIV‐1 diagnosis to today. Red and white triangles indicate detected and undetectable plasma HIV‐1 RNA, respectively. Donor chimerism (%donor cells, burgundy line; second right y‐axis). Solid black lines indicate times for induction chemo, consolidation chemo, alloHSCT, CD34+ boost and diagnosis of GvHD. The grey shaded area indicates time on ART (raltegravir, emtricitabine/tenofovir, darunavir/cobicistat; then bictegravir, emtricitabine, tenofovir). Light blue circles indicate timepoints of therapeutic drug monitoring without detectable antiretroviral drug levels (i.e. emtricitabine). Red hexagons and purple squares indicate timepoints with negative HIV intact proviral DNA assay (IPDA) and quantitative co‐cultures (QVOA) on leukapheresis PBMCs, respectively.

## OAA2706LB Innate Immune Signatures of Post‐Interventional Control in Very Early Treated Women Living With Clade C HIV (FRESH Cohort)

Y. Merad ^1,2,3^, K. L. Dong^1,2,4^, M. Lancien^1,2^, T. S. Tan^1,2^, T. Ndung'u^1,5,6^, M. Lichterfeld^1,7,4^, X. G. Yu^1,2,4^

^1^Ragon Institute of MGB, MIT and Harvard, Cambridge, United States; ^2^Massachussets General Hospital, Boston, United States; ^3^Université Claude Bernard Lyon 1, CIRI, INSERM U1111, CNRS UMR5308, ENS Lyon, Lyon, France; ^4^Harvard Medical School, Cambridge, United States; ^5^The Doris Duke Medical Research Institute, UKZN, HIV Pathogenesis Program (HPP), Durban, South Africa; ^6^Africa Health Research Institute (AHRI), Durban, South Africa; ^7^Brigham and Women's Hospital, Boston, United States

**Background**: Early ART initiation is associated with viral control following treatment interruption, yet the immunological mechanisms for control remain poorly understood. Here, we evaluated innate and adaptive immune responses in young South African women from the FRESH cohort living with HIV‐1 clade C who initiated antiretroviral therapy (ART) during acute HIV, and underwent analytical treatment interruption (ATI) as a part of a phase 2b clinical trial assessing two broadly neutralizing antibodies plus a TLR7 agonist, a median of 7 years after initiating ART (range: 3−8).

**Methods**: PBMCs from 12 (of 20) trial participants from whom data/samples were available were analysed. High‐dimensional spectral flow cytometry was used to profile conventional T and NK cells, γδ T cells, MAIT cells and NK‐like T cells. HIV‐specific T‐cell responses were assessed with intracellular cytokine staining. Participants were grouped based on ATI outcome (timing/magnitude of rebound viral load [VL] and time to meeting ART restart criteria) as controllers, partial controllers and non‐controllers.

**Results**: Two participants maintained VL below 1000 copies/mL for ≥ 2.5 years (controllers), two had delayed ART restart but VL sometimes >1000 copies/mL (partial controllers) and eight met ART restart criteria after a median of 117 days (non‐controllers). Pre‐trial, all participants had low frequencies of HIV‐specific IFNγ + TNFα + CD8 T cells irrespective of ATI outcome group. After viral rebound, CD8 frequency increased in non‐controllers to levels higher than in controllers and partial controllers. The key distinction in controllers was an NK cell signature marked by increased NKG2A+ (*p* < 0.05) and decreased KIR‐educated cells among the CD56dim NK subset (*p* < 0.05). Consistently, controllers had a trend towards higher frequency of the HLA‐B‐21T signal peptide polymorphism (*p* = 0.0667) relative to non‐controllers.

**Conclusions**: While HIV‐specific CTL expansion seems to merely result from antigen re‐exposure during rebound, controllers exhibited a distinct NK cell signature involving NKG2A education. These findings suggest that innate immune responses may play a role in maintaining post‐intervention control in young women who initiated ART very early. Further work is needed to resolve the functional properties and dynamics of these immune subsets in the setting of early treated HIV (see Figures 1 and 2).

**FIGURE 1:**
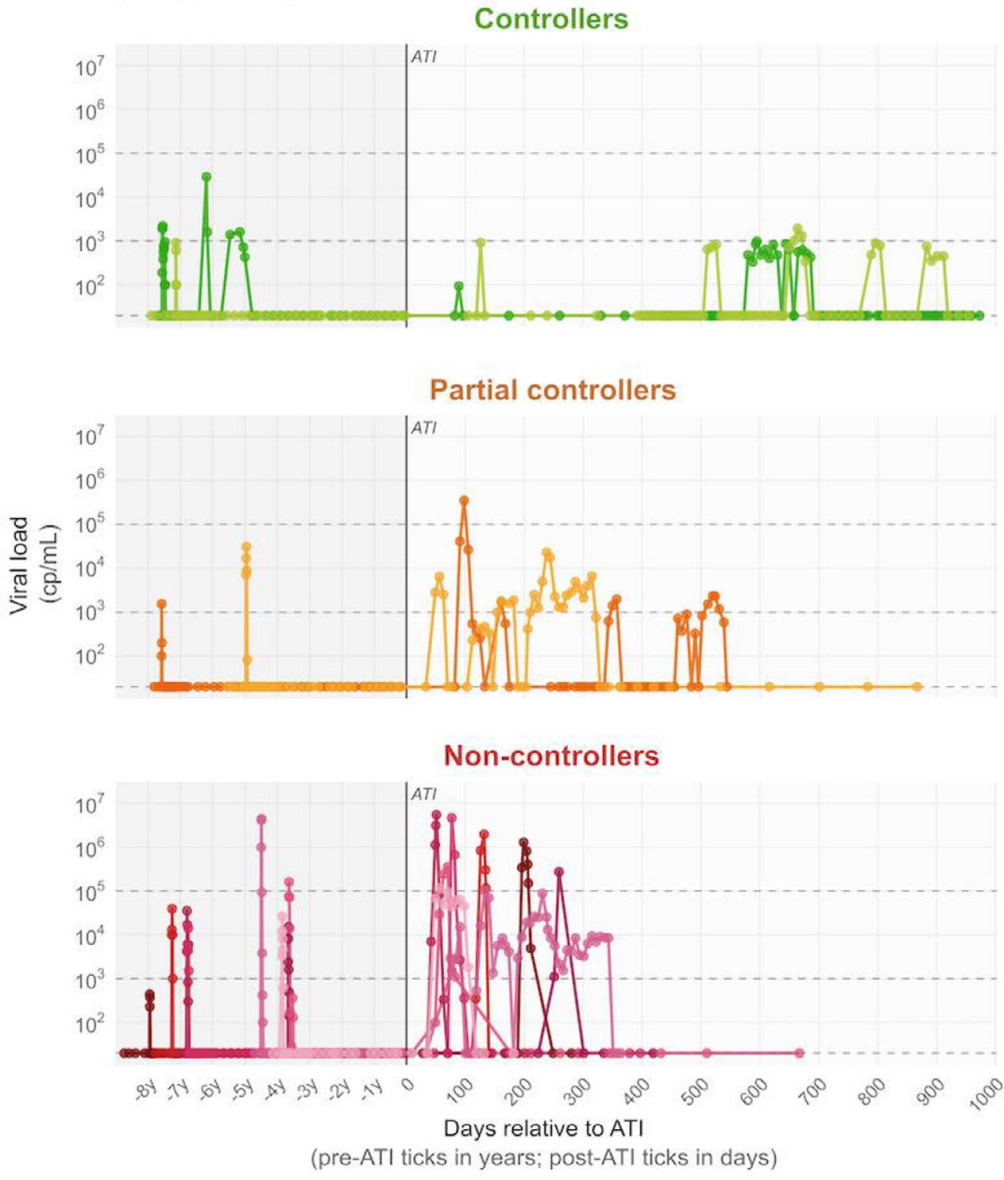
OAA2706LB | Longitudinal plasma viral load trajectories per participant.

**FIGURE 2:**
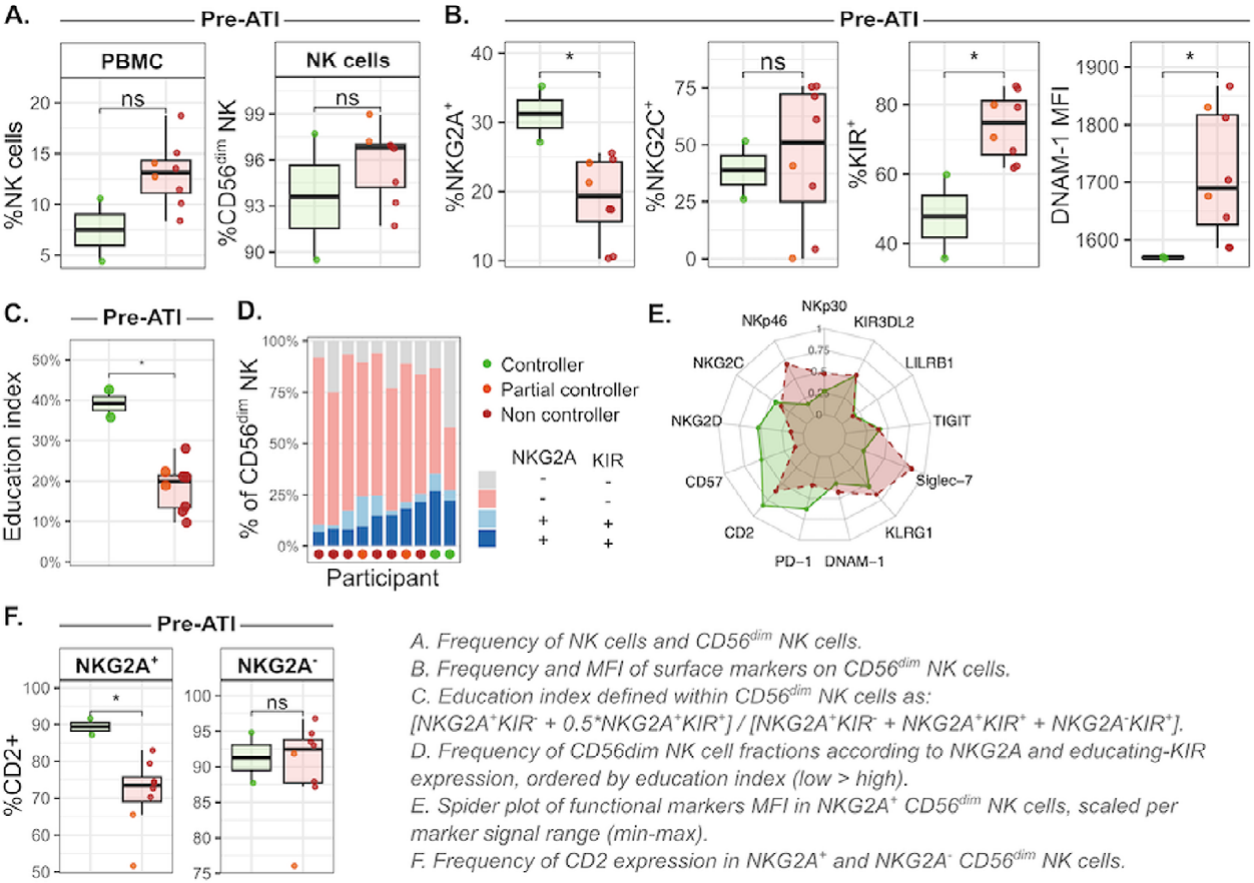
OAA2706LB | Frequency and phenotype of NK cell subsets at the pre‐ATI timepoint.

## OAA3006LB HIV‐1 mRNA‐LNP Vaccines Containing Clade C CH505 HIV‐1 Envelopes Protect Rhesus Macaques From Acquisition of HIV in an Autologous SHIV Challenge Model

W. B. Williams ^1^, X. Shen^2^, W. Rountree^3^, R. Spreng^3^, C. T. DeMarco^3^, T. Denny^3^, C. Bowman^3^, R. J. Edwards^3^, E. Kreider^4^, K. O. Saunders^1^, J. Pollara^2^, M. R. Betts^4^, D. Margolis^5^, S. Santra^6^, K. Bar^4^, D. Montefiori^2^, D. Weissman^4^, B. F. Haynes^7^, G. Ferrari^2^

^1^Duke University School of Medicine, Department of Surgery, Division of Surgical Sciences, Human Vaccine Institute, Durham, United States; ^2^Duke University School of Medicine, Department of Surgery, Division of Surgical Sciences, Durham, United States; ^3^Duke University School of Medicine, Human Vaccine Institute, Durham, United States; ^4^University of Pennsylvania, Perelman School of Medicine, Philadelphia, United States; ^5^University of North Carolina School of Medicine, Department of Medicine, Chapel Hill, United States; ^6^Harvard Medical School, Beth Israel Deaconess Medical Center, Boston, United States; ^7^Duke University School of Medicine, Department of Medicine, Human Vaccine Institute, Durham, United States

**Background**: We recently found that soluble‐stabilized envelope (Env) trimer protein derived from clade C HIV‐1 CH505 transmitted‐founder (TF) strain reproducibly generated protective neutralizing antibodies (NAbs) in a rhesus macaque (RM) autologous SHIV challenge model. mRNA‐based CH505TF env encapsulated by lipid nanoparticles (LNPs) served as boosting immunogen in HIV‐1 seronegative individuals (HVTN 312). Therefore, we evaluated the capacity of mRNA‐LNP HIV‐1 CH505 Env vaccines of clinical relevance to confer protection in RMs (NHP1008).

**Methods**: Nine NHP1008 RMs were primed (3X) bimonthly with mRNA‐LNP encoding CH505 M5.G458Y.N197D gp160 designed to induce precursors of the CH235 CD4bs broadly neutralizing antibody (bnAb) lineage. RMs were then boosted (3X) bimonthly with mRNA‐LNP CH505TF gp160 consistent with the vaccine strategy in HVTN 312. In addition, NHP1008 RMs received 3 monthly boosts with ferritin‐nanoparticle protein expressing CH505TF trimers to further develop CH505TF‐neutralizating activity. RMs were intra‐rectally challenged with a well‐established low dose CH505TF SHIV weekly (*n* = 10) starting at 2 months post‐final immunization (peak immunogenicity). We quantified plasma viraemia as SIV gag RNA copies/mL in qPCR, and plasma CH505TF NAb titres and epitope mapping in the TZM‐bl assay.

**Results**: NHP1008 RMs (3/9, 33%) were fully protected from autologous SHIV challenges compared to 100% acquisition rate in unimmunized, but SHIV CH505TF‐exposed controls (NHP170: *N* = 9). Moreover, 3/6 (50%) NHP1008 SHIV‐exposed RMs required 7−10 challenges for breakthrough acquisition, suggestive of partial‐protection. NHP1008 and NHP170 RMs required 9 (range = 4−10) and 2 (range = 1−7) median challenges, respectively, for breakthrough acquisition (*p*‐value = 0.00046, Log‐rank test); average peak viraemia was similar (∼6 Log10 RNA copies/mL) among SHIV‐acquired RMs. Additionally, all 9 (100%) NHP1008 RMs had plasma NAb signature suggestive of CD4bs Abs including CH235‐like bnAb precursors, and 7/9 (78%) generated plasma CH505TF NAbs [ID50 Geomean = 62; range = 22−352] at peak immunogenicity. One of 3 fully protected, and 5/6 SHIV‐infected, NHP1008 RMs generated plasma CH505TF NAbs at similar magnitudes, suggesting that CH505TF NAb titres were not the only contributors to protection.

**Conclusions**: We demonstrated HIV‐1 mRNA‐LNP vaccine‐induced protection in an autologous SHIV challenge model. Further studies will elucidate the specificities of protective immune responses to inform future vaccine designs.

## OAA3806LB Spatial Sequestration of Colonic HIV Reservoirs From CD8+ T Cells Fuelled by Self‐Sustained Immune Circuitry and Tertiary Lymphoid Structure Formation

J. Prigann ^1^, D. Boffelli^2^, A. Dietl^3^, A. Ralser^3^, L. Grzelak^1^, A. Rodriguez^4^, A. Naik^3^, N. R. Roan^1,5^, K. Pelka^3,6,7^, O. D. Klein^2^, M. Somsouk^4,8^, M. Ott^1,4,9^

^1^Gladstone Infectious Disease Institute, San Francisco, United States; ^2^Cedars‐Sinai Guerin Children's Los Angeles, Department of Pediatrics, Los Angeles, United States; ^3^Gladstone‐UCSF Institute of Genomic Immunology, San Francisco, United States; ^4^University of California San Francisco, Department of Medicine, San Francisco, United States; ^5^University of California San Francisco, Department of Urology, San Francisco, United States; ^6^University of California San Francisco, Department of Microbiology and Immunology, San Francisco, United States; ^7^Helen Diller Family Comprehensive Cancer Center UCSF, San Francisco, United States; ^8^San Francisco General Hospital UCSF, Division of Gastroenterology, San Francisco, United States; ^9^Biohub, San Francisco, United States

**Background**: In people with HIV (PWH), the gut is a major viral reservoir and the site of chronic immune activation, often linked to impaired gut health and inflammation‐induced comorbidities. However, how HIV continues to drive chronic immune activation, epithelial dysfunction and viral persistence despite decades of antiretroviral therapy (ART) remains unknown.

**Methods**: To address this, we performed single‐cell RNA sequencing and single cell‐resolved spatial transcriptomics on gut biopsies from people with ART‐controlled HIV and controls without HIV.

**Results**: Our results demonstrate persistent inflammatory signalling along the intestinal tract driven by IFN‐γ and TNF, and accompanied by CD8+ T cell accumulation. In the small intestine, CD8+ T cells infiltrate existing gut‐associated lymphoid tissue at local CXCR3 ligand‐enriched sites, promoting a feed‐forward loop of immune cell recruitment. In the large intestine, where organized lymphoid structures are largely absent, subepithelial CXCR3 ligand expression is associated with CD8+ T cell infiltration into epithelial stem cell niches and correlate with IFN‐γ‐induced stress signatures in adjacent colonocytes. Notably, the colonic mucosa of PWH contains tertiary lymphoid structures (TLS) spanning multiple maturation stages, from unstructured lymphocyte aggregates to mature lymphoid structures with distinct T and B cell zones defined by CCL19/CCL21 and CXCL13 gradients and active germinal centres. TLS remodel the surrounding tissue microenvironment, with adjacent crypts exhibiting aberrant regenerative gene signatures and irregular morphology consistent with chronic stem cell damage. Furthermore, rare HIV‐p24+ cells within TLS are spatially separated from CD8+ T cells, suggesting that TLS may serve as sanctuary sites for HIV persistence in the gut.

**Conclusions**: Together, these findings reveal unique site‐specific remodelling of intestinal immune niches and identify colonic TLS as hubs linking chronic inflammation, epithelial dysfunction and HIV persistence during ART.

## OAB0105LB Switching to Doravirine/Islatravir (100/0.25 mg) Once Daily Maintained Viral Suppression at Week 96 in the Presence of Baseline NNRTI Resistance‐Associated Mutations and/or M184I/V in Proviral DNA

T. Diamond, U. Nwoke, M. Fuszard, C. Zhang, S. Klopfer, M. Li, W. Greaves, J. Kim, M. C. Fox, E. Asante‐Appiah

Merck & Co., Inc., Rahway, United States

**Background**: Doravirine/islatravir (DOR/ISL 100/0.25 mg) is approved in the US and Japan for the treatment of HIV‐1. Three ongoing Phase 3 studies in virologically suppressed people living with HIV‐1 (P051‐NCT05631093; P052‐NCT05630755; P054‐NCT05766501) have demonstrated maintenance of virologic suppression through Week (W) 96. This analysis examined the prevalence and impact of baseline resistance‐associated mutations (RAMs) in proviral DNA on the virologic response to DOR/ISL at W96 in these studies, with a primary focus on non‐nucleoside reverse transcriptase inhibitor (NNRTI) RAMs and M184I/V.

**Methods**: Participants with HIV‐1 RNA <50 copies/mL and no history of virologic failure or documented DOR resistance at baseline were randomized (2:1) to switch to DOR/ISL 100/0.25 mg once‐daily or to continue baseline ART (bART; P051) or BIC/FTC/TAF (P052). In P054, adults who previously received DOR/ISL 100/0.75 mg switched to 100/0.25 mg on Day 1. In P051, participants continuing bART switched to DOR/ISL 100/0.25 mg at W48. Baseline RAMs present in proviral DNA were identified using GenoSure Archive (Monogram Biosciences).

**Results**: Among 1143 participants across the studies with baseline proviral DNA data who received DOR/ISL 100/0.25 mg on Day 1 and had W96 virologic data, HIV‐1 RNA <50 copies/mL was maintained in 294‐of‐300 (98%) or 63‐of‐66 (95%) of participants with baseline NNRTI RAMs or M184I/V, respectively. Among 34 participants with both NNRTI RAMs and M184I/V, HIV‐1 RNA <50 copies/mL at W96 was maintained in 31‐of‐34 (91%). The other three participants discontinued by W48; two had baseline exclusionary DOR RAMs and one had treatment‐emergent DOR resistance in a post‐hoc analysis. Among 155 participants in P051 with baseline proviral DNA data who switched from bART at W48 and had W96 virologic data, HIV‐1 RNA <50 copies/mL was maintained in all 31 participants with NNRTI RAMs, all four participants with M184I/V, and the one participant with both NNRTI RAMs and M184I/V.

**Conclusions**: Switching to DOR/ISL 100/0.25 mg in Phase 3 clinical studies maintained viral suppression at W96 despite the presence of baseline NNRTI RAMs or M184I/V in proviral DNA (Table 1).

**TABLE 1:**
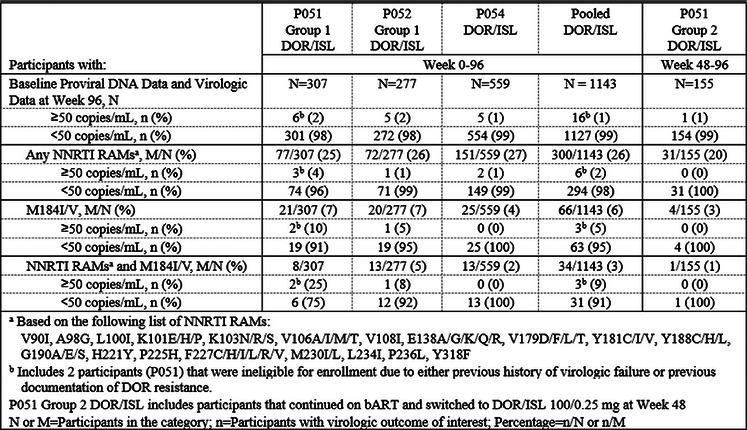
OAB0105LB | Virologic outcomes at week 96 in participants with baseline NNRTI RAMs and/or M184I/V.

## OAB0106LB In VOGUE: DTG/3TC 2‐Drug Regimen is Non‐Inferior to BIC/FTC/TAF 3‐Drug Regimen in a Randomized Trial in Adults With HIV Naive to Treatment

J. Ghosn ^1,2^, F. Pulido^3^, G. D. Lopardo^4^, A. Olczak^5^, D. Ripamonti^6^, C. Martin^7^, O. P. Ceballos^8^, H. Stocker^9,10^, M. J. Mendez Vasquez^11^, H. Gatanaga^12^, S. Metallidis^13^, D. Elbirt^14^, G. Whitlock^15^, J. I. Mateo González^16^, P. Mohammed^17^, K. Wang^18^, R. Wang^19^, A. Jewell^17^, E. Elliot^20^, R. Moodley^17^, B. Jones^17^, M. Kisare^17^, P. Budnik^17^, J. van Wyk^17^, for the VOGUE study group

^1^Université Paris Cité, INSERM, IAME, Paris, France; ^2^Bichat University Hospital, Department of Infectious Diseases, Paris, France; ^3^Hospital Universitario 12 de Octubre, imas12, UCM, Madrid, Spain; ^4^Fundacion Centro de Estudios Infectologicos, Buenos Aires, Argentina; ^5^Nicolaus Copernicus University Ludwik Rydygier Collegium, Bydgoszcz, Poland; ^6^Infectious Diseases Unit, ASST Papa Giovanni XXIII, Bergamo, Italy; ^7^Centre Hospitalier Universitaire Saint‐Pierre, Brussels, Belgium; ^8^Unidad de Atención Médica e Investigación en Salud, Mérida, Mexico; ^9^Department of Infectious Diseases, St Joseph Hospital, Berlin‐Tempelhof, Berlin, Germany; ^10^EPIMED GmbH, Berlinger, Germany; ^11^Porto University Hospital Centre, Lisboa, Portugal; ^12^AIDS Clinical Center, National Center for Global Health and Medicine, Tokyo, Japan; ^13^AHEPA Hospital, Aristotle University of Thessaloniki, Thessaloniki, Greece; ^14^Allergy, Immunology and HIV Unit, Kaplan Medical Center, Rehovot, Israel; ^15^Department of HIV/GUM, Chelsea and Westminster Hospital NHS Foundation Trust, London, United Kingdom; ^16^Consorcio Hospital General Universitario de Valencia, Valencia, Spain; ^17^ViiV Healthcare, London, United Kingdom; ^18^GSK, Collegeville, United States; ^19^ViiV Healthcare, Durham, United States; ^20^ViiV Healthcare, Madrid, Spain

**Background**: Antiretroviral therapy (ART) that achieves virologic suppression (VS) using fewer antiretroviral agents is an optimized approach to HIV treatment initiation. VOGUE is the first randomized, head‐to‐head study comparing the 2‐drug regimen dolutegravir/lamivudine (DTG/3TC) with the 3‐drug regimen bictegravir/emtricitabine/tenofovir alafenamide (BIC/FTC/TAF) as initial ART, including among individuals with high viral loads (VLs) or low CD4+ cell counts.

**Methods**: VOGUE (NCT05979311) is an ongoing phase 3b, randomized, open‐label, non‐inferiority, multi‐country study comparing DTG/3TC versus BIC/FTC/TAF as first‐line ART in adults with HIV‐1. Participants were enrolled without baseline VL or CD4+ cell count restrictions, and treatment was initiated before the availability of baseline genotype resistance testing results. The primary endpoint was the proportion of participants with HIV‐1 RNA <50 copies/mL at Week 48 (FDA Snapshot algorithm; –10% non‐inferiority margin). Secondary endpoints included time to VS (HIV‐1 RNA <50 copies/mL), confirmed virologic withdrawal (CVW; 2 consecutive VLs meeting non‐response or rebound criteria), treatment‐emergent resistance and safety.

**Results**: Among 509 randomized participants (DTG/3TC, *n* = 254; BIC/FTC/TAF, *n* = 255), median (range) age was 33 (18−84) years, 16% were assigned female sex at birth and 16% were non‐White; demographics were balanced between groups. At baseline, 47% and 16%, respectively, had VL ≥100,000 or ≥500,000 c/mL, and 16% had CD4+ cell count <200 cells/mm^3^. DTG/3TC demonstrated non‐inferior efficacy to BIC/FTC/TAF at Week 48, with HIV‐1 RNA <50 c/mL achieved in 89% and 92% of participants, respectively (Table 1; adjusted difference, –3% [95% CI, –8%, 2%]). Median time to VS was rapid and similar between groups (DTG/3TC, 4.1 [95% CI, 4.1, 4.1] weeks; BIC/FTC/TAF, 4.1 [95% CI, 4.1, 4.3] weeks; hazard ratio, 1.1 [95% CI, 0.9, 1.3]). CVWs were similar between treatment groups (DTG/3TC, *n* = 6; BIC/FTC/TAF, *n* = 7), with no treatment‐emergent resistance identified. Safety outcomes were comparable (Table 2), with no new safety signals observed.

**TABLE 1:**
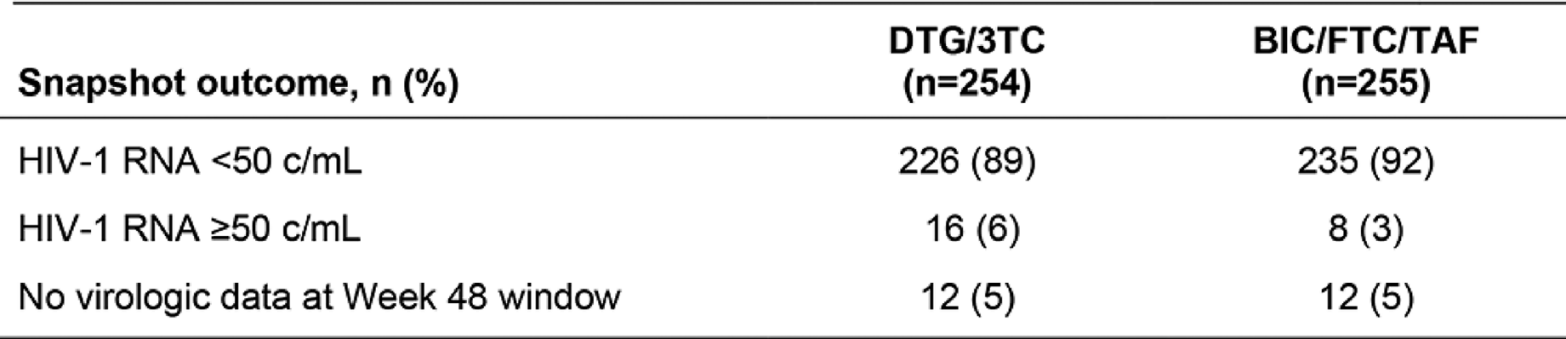
OAB0106LB | Virologic outcomes at week 48.

**TABLE 2:**
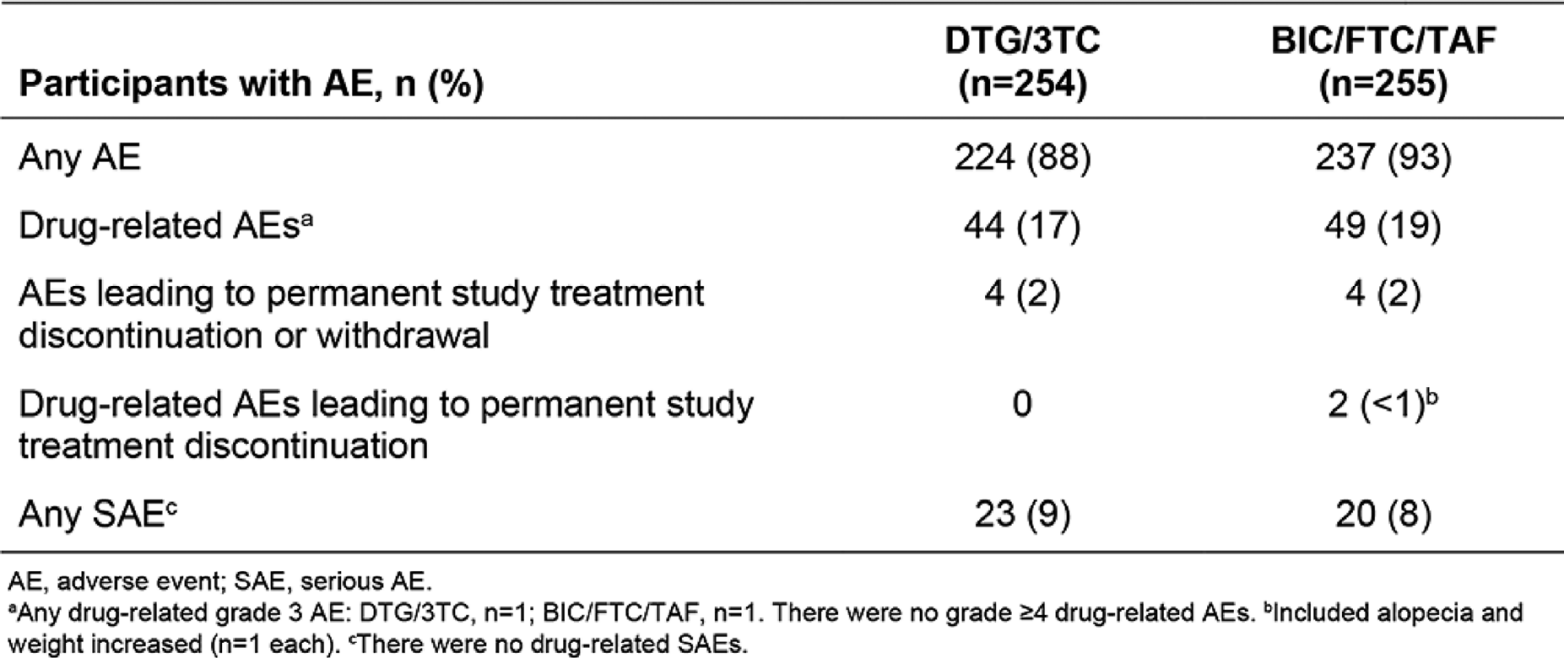
OAB0106LB | Summary of AEs at week 48.

**Conclusions**: In individuals naive to treatment, DTG/3TC is non‐inferior to BIC/FTC/TAF through Week 48, with no treatment‐emergent resistance. In a study population that includes high VLs and low CD4+ cell counts at baseline, these findings demonstrate DTG/3TC as an effective and simplified initial ART regimen.

## OAB1405LB Randomized, Active‐Controlled, Phase 2b Study Evaluating Efficacy and Safety of Switch to Islatravir (ISL) 2 mg With Ulonivirine (ULO) 200 mg Once Weekly in Virologically Suppressed Adults Living With HIV‐1

A. F. Luetkemeyer ^1^, M. Bloch^2^, A. Scribner^3^, F. Hinestrosa^4^, P. Kumar^5^, G. Sinclair^6^, C. Van Dam^7^, J. Morales‐Ramirez^8^, M. Gubler^9^, U. Okoro^10^, X. Xu^11^, K. Beckey^11^, L. M. Stamm^11^, R. P. Matthews^11^, M. Li^11^

^1^University of California San Francisco, San Francisco, United States; ^2^Holdsworth House, Sydney, Australia; ^3^DCOL Center for Clinical Research, Longview, United States; ^4^Orlando Immunology Center, Orlando, United States; ^5^Georgetown University Medical Center, Washington, DC, United States; ^6^Prism Health North Texas, Dallas, United States; ^7^Cone Health Regional Center for Infectious Disease, Greensboro, United States; ^8^Clinical Research Puerto Rico, San Juan, United States; ^9^Bern University Hospital, Bern, Switzerland; ^10^Merck & Co, Inc., Rahway, United States; ^11^Merck & Co., Inc., Rahway, United States

**Background**: The ongoing MK‐8591B‐060 study evaluates the nucleoside reverse‐transcriptase translocation inhibitor (NRTTI) ISL combined with the non‐nucleoside reverse‐transcriptase inhibitor (NNRTI) ULO as oral, once‐weekly treatment in virologically suppressed people living with HIV‐1.

**Methods**: Phase 2b, randomized, open‐label study (NCT06891066). Eligible participants (HIV‐1 RNA <50 copies/mL for ≥6 months on BIC/FTC/TAF, CD4+ T‐cell count ≥200 cells/mm^3^, no prior therapy failure or known mutations associated with ULO resistance) were randomized 1:1 to continue once‐daily BIC/FTC/TAF or switch to once‐weekly ISL (2 mg) with ULO (200 mg). The primary endpoint was the proportion of participants with HIV‐1 RNA ≥50 copies/mL at Week 24 (W24). Changes in CD4+ T‐cell counts and safety through W24 were also assessed. No formal hypothesis testing was planned.

**Results**: 78 participants switched to ISL + ULO and 79 continued BIC/FTC/TAF. The study population comprised 26.8% cisgender women, 1.9% trans women, 31.8% Black/African‐American participants and 24.2% Hispanic/Latino participants. Median age was 48.0 years (interquartile range: 39−57 years) and the median baseline CD4+ T‐cell count was 810 cells/mm^3^ (interquartile range: 655−1016 cells/mm^3^). At Week 24 (Table 1), no participants receiving ISL + ULO versus 1.3% (1/79) continuing BIC/FTC/TAF had HIV‐1 RNA ≥50 copies/mL (difference: –1.3%; 95% CI: –6.9, 3.5). No participants had HIV‐1 RNA ≥200 copies/mL, and none met criteria for post‐baseline resistance analysis. Mean changes from baseline CD4+ T‐cell counts were comparable between groups. Adverse event (AE) rates were generally similar between groups (Table 2). The rate of drug‐related AEs was higher with ISL + ULO (10/78 participants, 12.8%) than BIC/FTC/TAF (0.0%), which is not unexpected in an open‐label switch study. In the ISL + ULO group, there were three serious AEs (none drug related) and one treatment discontinuation due to drug‐related AEs (grade 2 “skin reaction”). No HBV reactivations or new acquisitions occurred.

**TABLE 1:**
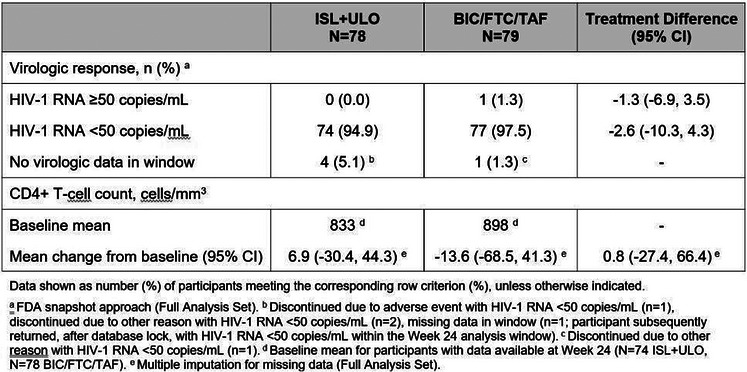
OAB1405LB | Key efficacy outcomes at week 24.

**TABLE 2:**
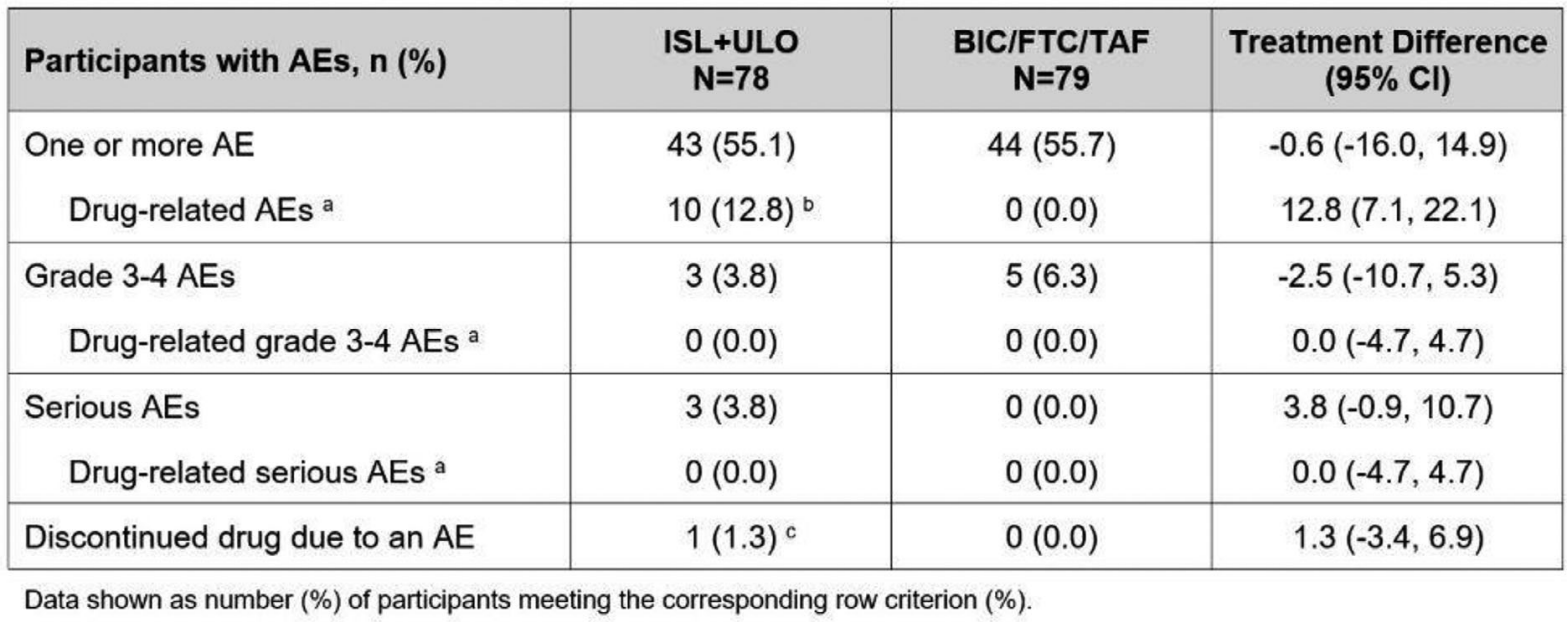
OAB1405LB | Safety summary through week 24.

**Conclusions**: ISL + ULO maintained a high rate of confirmed virologic suppression through W24, with efficacy comparable to BIC/FTC/TAF. ISL + ULO was generally well‐tolerated; no safety concerns were identified.

## OAB1406LB Once‐Weekly Oral Islatravir/Lenacapavir (ISL/LEN) Versus Daily Bictegravir/Emtricitabine/Tenofovir Alafenamide (B/F/TAF) in Virologically Suppressed Adults With HIV‐1: Week 48 Results of the ISLEND‐1 Phase 3 Trial

J. K. Rockstroh ^1^, M. N. Ramgopal^2^, A. Curran Fabregas^3^, M. Hedgcock^4^, K. A. Workowski^5^, S. Ronot‐Bregigeon^6^, I. Cassetti^7^, C. Brinson^8^, S. K. Gupta^9^, C.‐C. Hung^10^, M. O'Reilly^11^, J. Slim^12^, H. Gatanaga^13^, P. Shalit^14^, S.‐Y. Liu^15^, S. O. Klopfer^16^, F. Shihadeh^15^, N. Patel^16^, R.‐C. Mercier^15^, M. Shaughnessy^16^, H. Dvory‐Sobol^15^, D. SenGupta^15^, C. Llamoso^16^, M. S. Rhee^15^, C. Orkin^17^, for the ISLEND‐1 Study Team

^1^University Hospital Bonn, Department of Medicine I, Bonn, Germany; ^2^Midway Immunology and Research Center, Fort Pierce, United States; ^3^Vall d'Hebron University Hospital, Department of Infectious Diseases, Barcelona, Spain; ^4^Spectrum Health, Vancouver, Canada; ^5^Emory University, Department of Medicine, Atlanta, United States; ^6^APHM, Hôpital Sainte‐Marguerite, Aix‐Marseille Université, Marseille, France; ^7^Helios Salud, Buenos Aires, Argentina; ^8^Central Texas Clinical Research, Austin, United States; ^9^Indiana University School of Medicine, Indianapolis, United States; ^10^National Taiwan University Hospital Yunlin Branch, Department of Internal Medicine, Douliu City, Taiwan, Province of China; ^11^East Sydney Doctors, Darlinghurst, Australia; ^12^New York Medical College, Valhalla, United States; ^13^Japan Institute for Health Security, National Center for Global Health and Medicine, Tokyo, Japan; ^14^Tribalmed LLC, Seattle, United States; ^15^Gilead Sciences, Inc., Foster City, United States; ^16^Merck & Co., Inc., Rahway, United States; ^17^SHARE Collaborative, Blizard Institute, Faculty of Medicine and Dentistry, Queen Mary University of London, London, United Kingdom

**Background**: Once‐daily single‐tablet regimens transformed the treatment landscape of HIV‐1; however, adherence and persistence challenges still limit effective treatment. Long‐acting oral treatment options could help meet the diverse needs of people with HIV‐1 (PWH) and improve outcomes.

**Methods**: ISLEND‐1 (NCT06630286) is a global, multicentre, double‐blind, Phase 3 noninferiority trial. Virologically suppressed adults with HIV‐1 on B/F/TAF, with no previous virologic failure, were randomized 1:1 to switch to once‐weekly oral ISL/LEN (2/300 mg) or continue once‐daily B/F/TAF, plus matching placebos, for 96 weeks. The primary efficacy endpoint was the proportion of participants with HIV‐1 RNA ≥50 copies/mL at Week (W) 48 (US FDA‐defined snapshot algorithm; 4% noninferiority margin). Change from baseline in CD4+ T‐cell count and ISL/LEN discontinuations due to treatment‐emergent adverse events (AEs) were key secondary endpoints. The study will remain blinded until all participants complete their Week 96 visit.

**Results**: 607 participants were treated (ISL/LEN: *N* = 304; B/F/TAF: *N* = 303); median age was 49 years (48% ≥50 years; 15% ≥65 years), 21% were female, 31% Black and 26% Hispanic or Latine. Baseline CD4+ T‐cell count was 739 and 745 cells/µL in the ISL/LEN and B/F/TAF group, respectively. At W48, 0 ISL/LEN and 1 (0.3%) B/F/TAF participants had HIV‐1 RNA ≥50 copies/mL (Figure 1; difference: –0.3%; 95.002% confidence interval [CI]: –1.4, 0.8%), demonstrating noninferiority. 284 (93.4%) ISL/LEN and 280 (92.4%) B/F/TAF participants had HIV‐1 RNA <50 copies/mL (difference: 1.0%, 95% CI: –3.2, 5.2%). Mean change in CD4+ T‐cell count at W48 was –10 and –18 cells/µL with ISL/LEN and B/F/TAF, respectively (least‐squares mean difference: 12; 95% CI: –16, 39). There were no between‐group differences in lymphocyte counts at W48. No participant discontinued due to decreases in CD4+ T‐cells or lymphocyte counts. ISL/LEN was generally well tolerated, with a safety profile comparable to B/F/TAF (Table 1). One (0.3%) participant discontinued due to an AE of hepatitis B (incident infection in an unvaccinated individual).

**FIGURE 1:**
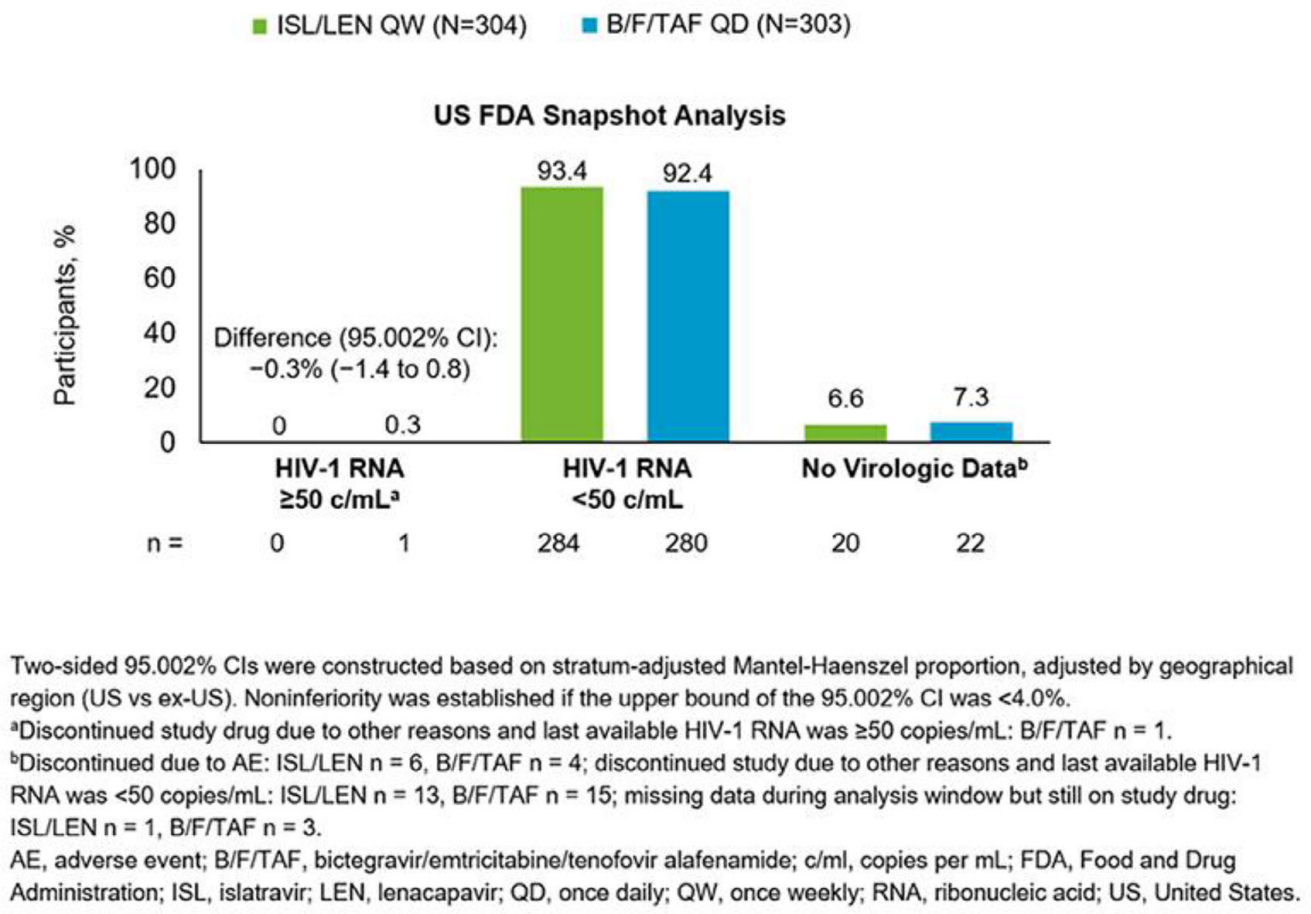
OAB1406LB | Virologic outcomes at week 48.

**TABLE 1:**
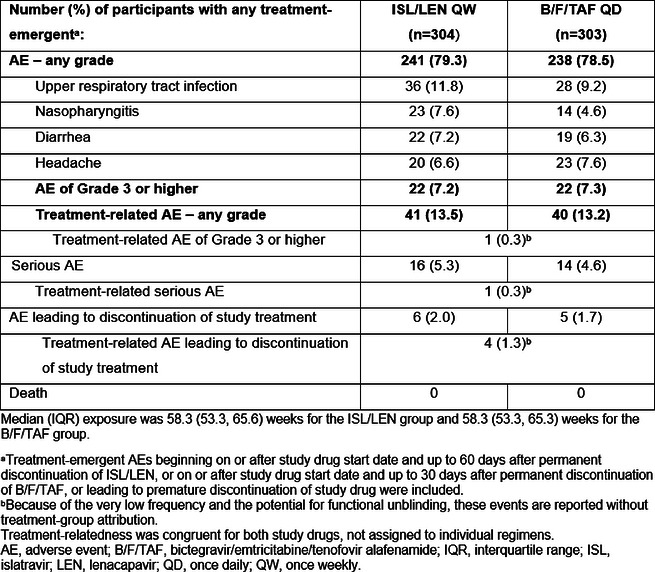
OAB1406LB | Safety summary.

**Conclusions**: ISL/LEN was noninferior to B/F/TAF, with no virologic failures at W48, and was well tolerated. ISL/LEN has the potential to be the first complete once‐weekly oral single‐tablet treatment regimen for virologically suppressed PWH.

**Funding**: Gilead Sciences, Inc. in collaboration with Merck Sharp & Dohme LLC.

## OAB2006LB High Virological Failure in Patients With Major DTG‐Associated DRTs Continuing on Standard Dose DTG: The Ndovu RCT

L. A. Ombajo ^1,2^, J. Penner^3,2^, V. Omodi^1,2^, J. Nkuranga^1,2^, Z. Muikamba^1^, E. W. Kamau^1^, D. Wamalwa^4^, A. Hill^5^, I. Ayakaka^6^, N. Ismael^7^, P. Munseri^8^, M. Bakari^8^, E. Otieno^1,2^, B. Ricky^1,2^, D. Khasowa^1^, J. Wagude^9^, Ndovu Study Group

^1^University of Nairobi, Clinical Medicine and Therapeutics, Nairobi, Kenya; ^2^University of Nairobi, Center for Epidemiological Modelling and Analysis, Nairobi, Kenya; ^3^University of British Columbia, Department of Family Practice, Vancouver, Canada; ^4^University of Nairobi, Paediatrics and Child Health, Nairobi, Kenya; ^5^University of Liverpool, Pharmacology and Therapeutics, Liverpool, United Kingdom; ^6^Solidar Med, Partnerships for Health, Maseru, Lesotho; ^7^Instituto Nacional de Saúde, Marracuene, Mozambique; ^8^Muhimbili University of Health and Allied Sciences, Internal Medicine, Dar es Salaam, Tanzania, the United Republic of America; ^9^Siaya County Referral Hospital, Siaya, Kenya

**Background**: There is insufficient data to inform the management of dolutegravir (DTG) resistance. We report results of the Ndovu study.

**Methods**: Ndovu is a multi‐country, open‐label, randomized, active‐controlled trial. Participants on DTG for at least 6 months, with persistent viraemia of ≥ 200 copies/mL despite enhanced adherence counselling, and ≥ one major DTG‐associated drug resistance mutations (DRMs) are randomized 1:1 to continue once daily DTG or to switch to ritonavir boosted darunavir (DRV/r), in combination with tenofovir (abacavir for children <30 kg) and lamivudine. Viral load is measured at months 1 and 3 to assess early viral suppression and at months 6, 9 and 12 to assess durability of suppression. The primary outcome is proportion of participants with viral load ≤200 copies/mL at month 6. On 8 April 2026, an early review of an interim analysis by the Independent Data Safety and Monitoring Board recommended stopping randomization to the DTG arm. We present the interim results on which this recommendation was made.

**Results**: Between 1 September 2025 and 2 April 2026, 112 participants with major DTG‐associated DRMs were screened, 7 had suppressed to a VL of <200 copies/mL by the time of screening, 72 participants were randomized, 38 to DTG and 34 to DRV/r. Median age was 36 years (IQR 20, 42), 60% were female and 59 (81%) had predicted high level resistance to DTG (Table 1). At month 1, 7/37 (19%) on DTG and 24/30 (80%) on DRV/r had a 1.0 log reduction in viral load (Figure 1) (difference [95% CI],–61.1 [–80.0 to –42.0], *p* = <0.001), At month 3, of 47 participants achieving a month 3 visit while on their assigned arm, 3/23 (13%) on DTG and 20/24 (83%) on DRV/r had VL <200 copies/mL (difference [95% CI],–70.3 [–91.0 to –50.0], *p* = <0.001).

**TABLE 1:** OAB2006LB | Participants baseline characteristics.

Characteristic (*N* = 72)	Continue DTG (*N* = 38)	Switch to DRV/r (*N* = 34)
**Country**		
Kenya	38 (100%)	33 (97.1%)
Lesotho	0	1 (2.9%)
Mozambique	0	0
Tanzania	0	0
**Sex**		
Female	25 (65.8%)	18 (52.9%)
Male	13 (34.2%)	16 (47.1%)
**Age (years)**		
Median (IQR)	30.5 (18.0, 42.0)	37.5 (20.0, 44.0)
≥15 years	32 (84.2%)	28 (82.4%)
3−14 years	6 (15.8%)	6 (17.6%)
**Enrolment viral load category**		
400−999	1 (2.6%)	0 (0.0%)
1000−9999	18 (47.4%)	15 (44.1%)
10,000−99,999	12 (31.6%)	13 (38.2%)
100,000−499,999	5 (13.2%)	5 (14.7%)
≥500,000	2 (5.3%)	1 (2.9%)
**Major resistance mutations**		
1. DTG‐associated DRMs		
G118R +/− (E138K, N155H, R263K)	22 (57.9%)	19 (55.9%)
N155H +/− (E92Q)	2 (5.3%)	4 (11.8%)
Q148HRK +/− (E138KA, G140A, N155H)	8 (21.1%)	8 (23.5%)
R263K	6 (15.8%)	3 (8.8%)
2. PI major DRMs	6 (15.8%)	6 (18.8%)
3. NRTI major DRMs	21 (55.3%)	24 (75.0%)
4. NNRTI major DRMs	18 (47.4%)	19 (59.4%)

Abbreviations: DRMs, drug‐resistance mutations; DRV/r, ritonavir‐boosted darunavir; DTG, dolutegravir; NNRTI, non‐nucleoside reverse transcriptase inhibitor; NRTI, nucleoside reverse transcriptase inhibitor; PI, protease inhibitor.

**FIGURE 1:**
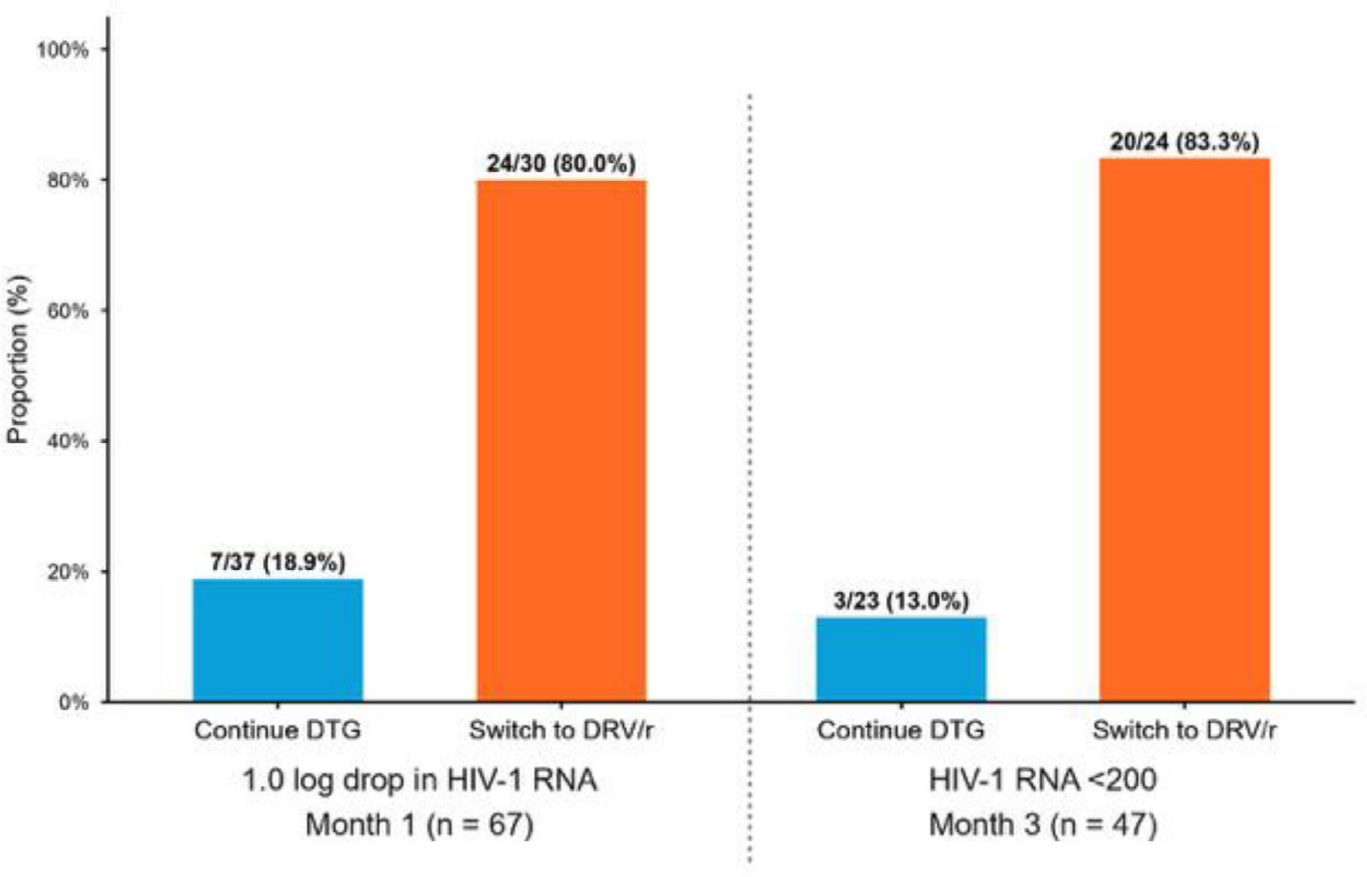
OAB2006LB | Viral load suppression at month 1 and at month 3.

**Conclusions**: In the presence of major DTG‐associated DRMs, continuing standard dose DTG was associated with high virological failure. With insufficient resources for DRT testing, better tools to predict DTG resistance and the need for switch to DRV/r are urgently required.

## OAB2306LB The Impact of Archived HIV‐1 Drug Resistance on Maintenance of Virologic Suppression Through Week 48 in Children Aged 2 to <15 Years Receiving DTG/3TC: PENTA‐21 Intensive Pharmacokinetic Sub‐Study

R. Wang ^1^, W. Chen^1^, A. Kumar^2^, M. K. Chan^3^, A. Gupta^2^, J. Boles^4^, J. C. Botha^5,6^, M. Byott^5,6^, M. J. Spyer^5,6^, P. Grant^7^, K. Gärtner^6^, K. Kamrunnahar^5,6^, M. Archary^8^, A. Violari^9^, E. Variava^9^, T. Cressey^10^, G. Ahimbisibwe^11^, A. R. Kekitiinwa^12^, C. Kityo^13^, C. Epalza^14^, C. Giaquinto^15^, D. Ford^3^, A. Turkova^3,16^, A. M. Buchanan^1^, E. Nastouli^5,6^

^1^ViiV Healthcare, Durham, United States; ^2^GSK, Bangalore, India; ^3^UCL Innovative Clinical Trials Unit, Institute of Clinical Trials and Methodology, London, United Kingdom; ^4^ViiV Healthcare, London, United Kingdom; ^5^Advanced Pathogen Diagnostic Unit, University College London Hospitals (UCLH), London, United Kingdom; ^6^Great Ormond Street Institute of Child Health, University College London, London, United Kingdom; ^7^Health Services Laboratories, London, United Kingdom; ^8^Enhancing Care Foundation, Department of Pediatrics and Children Health, Victoria Mxenge Hospital, University of KwaZulu‐Natal, Durban, South Africa; ^9^Perinatal HIV Research Unit, University of the Witwatersrand, Johannesburg, South Africa, Johannesburg, South Africa; ^10^AMS‐PHPT Research Collaboration, Faculty of Associated Medical Sciences, Chiang Mai University, Chiang Mai, Thailand; ^11^Makerere University—Johns Hopkins University Research Collaboration, Kampala, Uganda; ^12^Baylor College of Medicine Children's Foundation, Kampala, Uganda; ^13^Joint Clinical Research Centre, Kampala, Uganda; ^14^Pediatric Infectious Diseases Unit, Hospital Universitario 12 de Octubre, Madrid, Spain; ^15^University of Padova, Department of Women and Child Health, Padova, Italy; ^16^Great Ormond Street Hospital, London, United Kingdom

**Background**: Archived HIV‐1 drug resistance may persist in proviral DNA despite sustained virologic suppression (VS) in children with treatment experience. The prevalence and clinical relevance of resistance‐associated mutations (RAMs) during dolutegravir/lamivudine (DTG/3TC) maintenance remain uncertain. The D3/PENTA‐21 pharmacokinetic (PK) sub‐study evaluated the impact of archived HIV‐1 resistance on virologic outcomes through Week (W) 48.

**Methods**: Participants with historic resistance to 3TC or integrase strand transfer inhibitors (INSTIs) were excluded. Retrospective proviral HIV‐1 DNA analyses were performed on stored buffy coat samples using next‐generation sequencing for all PK sub‐study participants (*N* = 82) at baseline, W24, W48, viral rebound (confirmed viral load [VL] ≥50 copies/mL) and withdrawal. Virologic outcomes based on IAS‐USA (2022) RAMs across drug classes were determined by FDA Snapshot (HIV‐1 RNA ≥50 vs. <50 copies/mL) in participants with available genotypes, defined as the Proviral DNA Resistance Analysis Population (PRAP).

**Results**: Prevalence of archived major RAMs are shown in Table 1. The most frequent nucleoside reverse transcriptase inhibitor (NRTI) RAM was M184V/I, occurring in 16%, 15% and 16% at baseline, W24 and W48, respectively. A relatively high prevalence of the INSTI RAM G140R was noted, likely reflecting APOBEC‐mediated hypermutation in defective proviruses. In the PRAP, 78/79 (99%) and 76/79 (96%) participants maintained VS (VL <50 copies/mL) at W24 and W48, respectively. Participants with archived baseline resistance had similar suppression rates: 37/37 (100%) at W24 and 35/37 (95%) at W48. Virologic outcomes between participants with versus without archived baseline resistance are summarized in Table 2. Two participants, including one with archived M184V, had viral rebound through W48 and no treatment‐emergent INSTI or NRTI RAMs at failure.

**TABLE 1:**
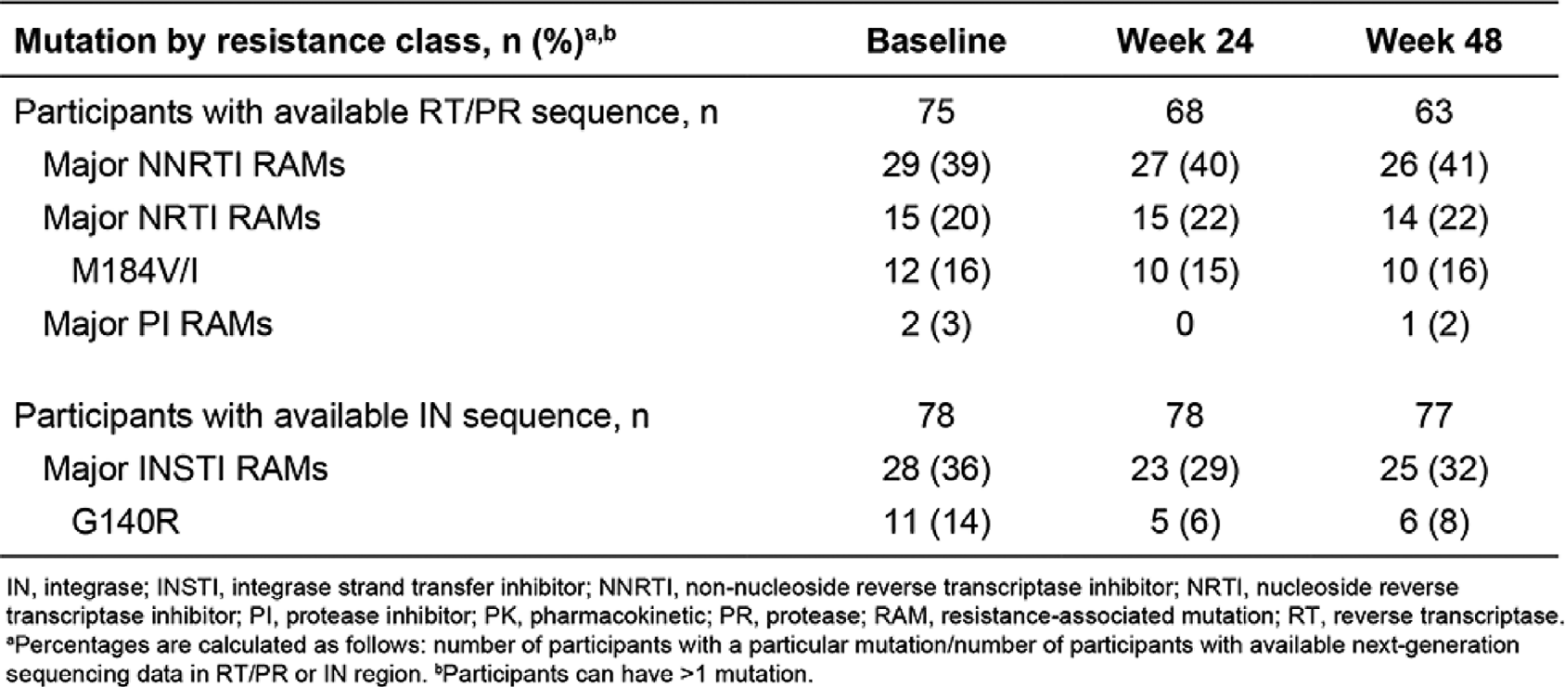
OAB2306LB | Prevalence of archived resistance mutations by drug class at baseline, week 24 and week 48 for all participants in the PK sub‐study (*N* = 82).

**TABLE 2:**
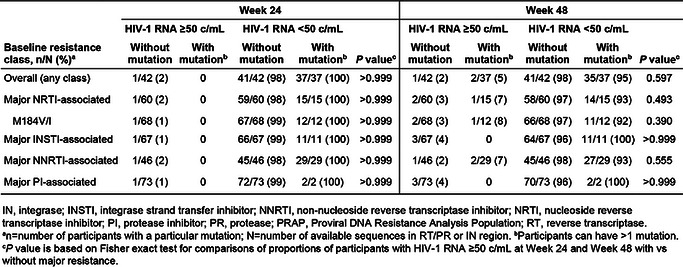
OAB2306LB | Comparison of virologic outcomes by archived resistance detected at baseline (snapshot analysis; PRAP).

**Conclusions**: In the PENTA‐21 nested PK sub‐study, high VS rates were maintained through W48 regardless of archived major RAMs. Pre‐existing M184V/I appeared to have limited impact on VS after switching to DTG/3TC, although large sample sizes and longer follow‐up are needed. Consistent with TANGO and SALSA studies in adults, these data support the potency and high genetic barrier of DTG/3TC as maintenance therapy in paediatric populations with treatment experience and VS, including in resource‐limited settings.

## OAB3406LB Opti‐DOR: 48 Week Data on DOR/3TC/TDF as an Alternative First‐Line Antiretroviral Therapy (ART) Regimen to Integrase Inhibitors for People With HIV and BMI>25 kg/m^2^ in a Randomized Phase 3B Non‐Inferiority Study

J. Woods ^1^, L. Lebina^2^, K. Moller^1^, G. Akpomiemie^1^, S. Sokhela^1^, N. Manentsa^1^, N. Behumuma^1^, A. Hill^3^, W. F. Venter^1^

^1^University of the Witwatersrand, Wits Ezintsha, Faculty of Health Sciences, Johannesburg, South Africa; ^2^Africa Health Research Institute (AHRI), Durban, South Africa; ^3^University of Liverpool, Department of Pharmacology and Therapeutics, Liverpool, United Kingdom

**Background**: Modern antiretroviral therapy has been associated with weight gain, particularly in women and Black populations. Tenofovir disoproxil fumarate (TDF)‐based and non‐integrase inhibitor therapy may offer a less weight‐promoting alternative. We compared virologic efficacy and metabolic outcomes of doravirine/lamivudine/TDF (DOR/3TC/TDF) versus dolutegravir/tenofovir alafenamide/emtricitabine (DTG/TAF/FTC) in antiretroviral‐naïve adults in South Africa.

**Methods**: An open‐label, randomized, phase 3b trial was conducted in antiretroviral‐naïve adults (initially enrolled adults with BMI >25 kg/m^2^; eligibility was later broadened by protocol amendment), with prespecified genotype exclusions, followed for 96 weeks and randomly assigned 1:1 to DOR/3TC/TDF or DTG/TAF/FTC. The primary endpoint was HIV‐1 RNA <50 copies/mL at Week 48 (modified FDA snapshot 10% non‐inferiority margin). Weight gain and metabolic changes were secondary endpoints of interest.

**Results**: Between October 2023 and March 2025, 600 participants were randomized: 413 (68.8%) were female; median age of 34 years (IQR 28–41); 597 (99.5%) were Black. DOR/3TC/TDF met the non‐inferiority criterion at Week 48, with HIV‐1 RNA <50 copies/mL in 266/299 (89.0%) participants versus 273/301 (90.7%) on DTG/TAF/FTC (adjusted difference −1.7% [95% CI −6.7 to + 3.2]). Virological failure occurred in 6/301 in DTG/TAF/FTC, 9/299 participants in DOR/3TC/TDF, 7 of which developed DOR resistance (2.3%). Median body weight gain was lower with DOR/3TC/TDF (3.00 vs. 5.00 kg; *p* < 0.001). Median DXA increases in total body fat percentage (1.53% vs. 2.16%; *p* < 0.001) and estimated visceral adipose tissue area (8.42 vs. 11.64 cm^2^; *p* = 0.037) were lower in the DOR/3TC/TDF arm. There were no changes in blood pressure or glucose over 48 weeks, with minimal lipid changes. Adverse events were comparable across arms.

**Conclusions**: DOR/3TC/TDF was non‐inferior to DTG/TAF/FTC for Week 48 virologic efficacy, both regimens were well tolerated, with DOR/3TC/TDF associated with less weight gain. Individual agent contribution to weight gain cannot be isolated as this was a regimen‐level comparison (see Table 1 and Figure 1).

**TABLE 1:**
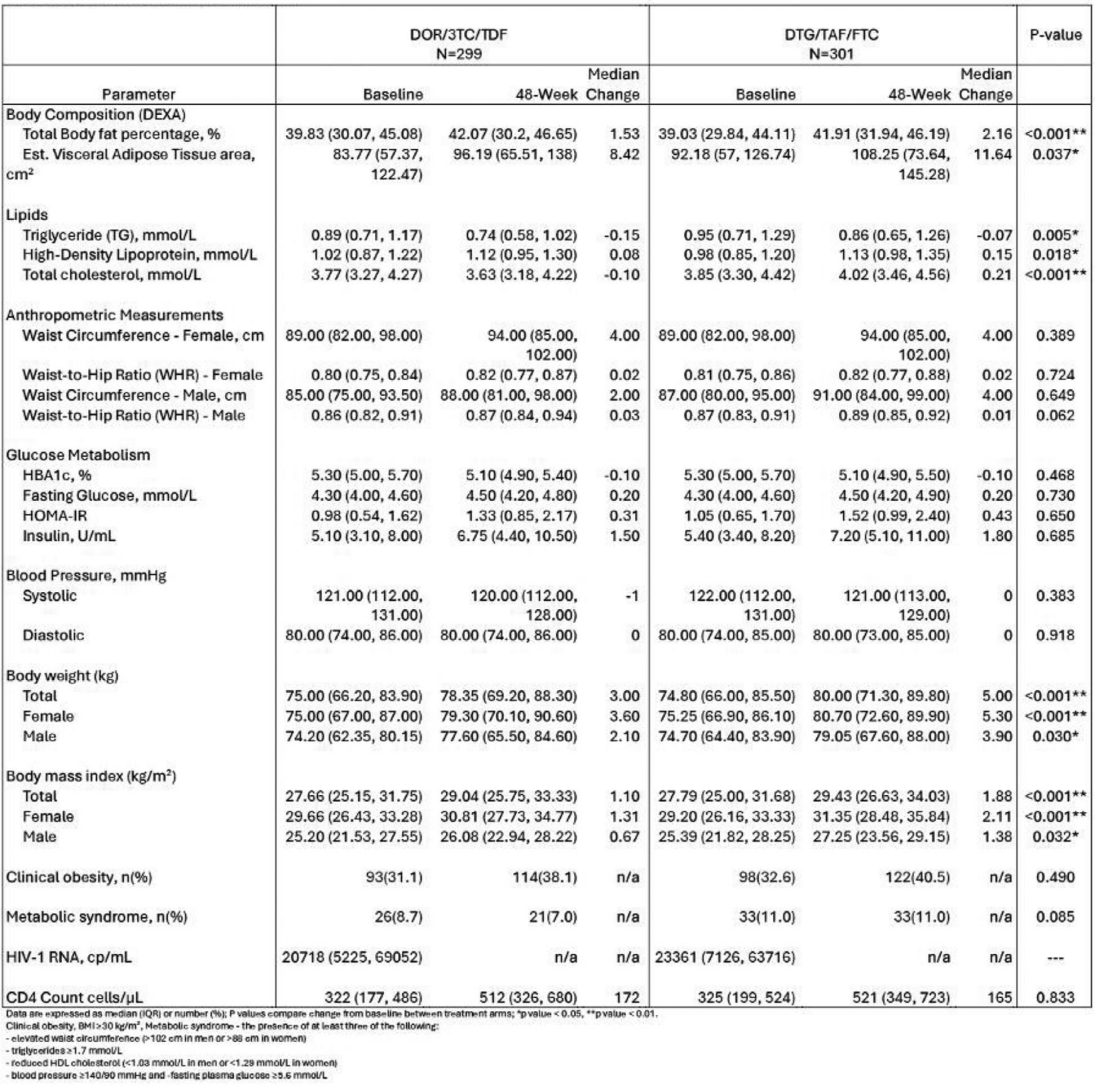
OAB3406LB | Opti‐DOR—Metabolic parameters.

**FIGURE 1:**
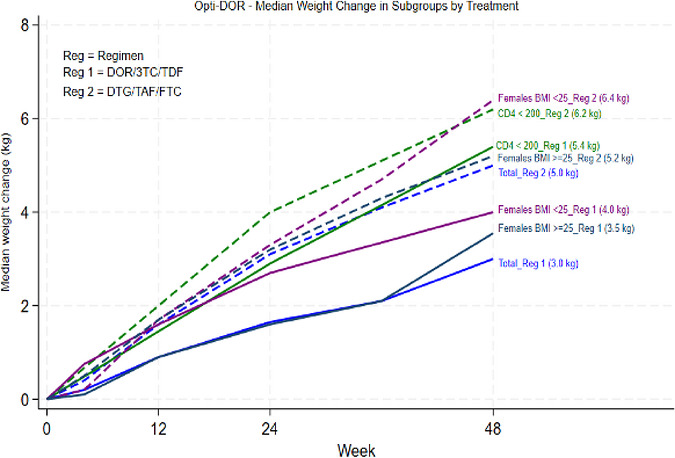
OAB3406LB

## OAB4106LB Combined Oral Treatment of Fluconazole Plus Flucytosine Versus Fluconazole Alone for the Treatment of HIV‐Associated Cryptococcal Antigenaemia: Results From the Multi‐Centre Phase‐3 EFFECT Randomized Trial

K. L. Murphy ^1,2,3^, N. Alam^4^, M. Y. S. Moosa^5^, G. S. M. Mfinanga^6^, S. L. Kivuyo^6,7^, J. S. Nel^8,9^, D. P. Wilson^10^, J. M. L. Tsitsi^8^, C. Menezes^8^, J. Black^11^, M. Bremer^1,12^, D. Kyazze^11,13^, E. Variava^8,14^, D. Verhagen^1^, D. Kabanda^1^, M. Xulu^1^, L. Maphalala^4^, L. Edkins^1,5^, B. Gosnell^5^, C. Cairns^5^, T. van Wyk^15,3^, R. P. Halley‐Stott^15,3^, N. Rutananukwa^6^, R. Ferdinand^6^, A. Mosses^6^, N. Makoko^6^, A. Thombrayil^1^, L. Richards^8^, I. Fatti^1^, R. Berghammer‐Böhmer^1^, J. V. Rundogo^1^, G. Okyere‐Manu^14^, V. Q. Dat^16^, T. Le^17,18^, J. Falconer^1,19^, A. Loyse^20,21^, C. Schutz^2,22^, R. M. Wake^20,1^, D. S. Lawrence^23,24,1^, E. van Widenfelt^25^, T. Chen^26^, D. Wang^26^, M. Eriksson^1^, J. Adams^20^, J. N. Jarvis^23,24^, S. Jaffar^25^, G. Meintjes^22,27,28^, T. S. Harrison^20,29^, N. P. Govender*^1,20,29^, S. F. Molloy*^20,1^, and the EFFECT trial team.

^1^University of the Witwatersrand, Wits Mycology Division, School of Pathology, Faculty of Health Sciences, Johannesburg, South Africa; ^2^University of Cape Town, Wellcome Centre for Infectious Diseases Research in Africa, Cape Town, South Africa; ^3^University of Cape Town, Neuroscience Institute, Cape Town, South Africa; ^4^Walter Sisulu University, Department of Internal Medicine, Dora Nginza Hospital, Gqeberha, South Africa; ^5^University of KwaZulu‐Natal, Division of Infectious Disease, Department of Internal Medicine, Victoria Mxenge Hospital, Nelson R. Mandela School of Medicine, Durban, South Africa; ^6^National Institute for Medical Research, Muhimbili Medical Research Centre, Dar es Salaam, Tanzania, the United Republic of; ^7^University of Barcelona, Faculty of Medicine and Health Sciences, Barcelona, Spain; ^8^University of the Witwatersrand, Division of Infectious Diseases, Department of Medicine, Faculty of Health Sciences, Johannesburg, South Africa; ^9^University of the Witwatersrand, Infectious Diseases and Oncology Research Institute, Faculty of Health Sciences, Johannesburg, South Africa; ^10^University of KwaZulu‐Natal, Department of Internal Medicine, Harry Gwala Regional Hospital, Nelson R. Mandela School of Medicine, Durban, South Africa; ^11^Walter Sisulu University, Infectious Diseases Unit, Department of Internal Medicine, Livingstone Tertiary Hospital, Gqeberha, South Africa; ^12^University of Cape Town, Department of Medical Microbiology, Groote Schuur Hospital, Cape Town, South Africa; ^13^Nelson Mandela University, Department of Medicine, Faculty of Health Sciences, Gqeberha, South Africa; ^14^University of the Witwatersrand, Perinatal HIV Research Unit, Faculty of Health Sciences, Klerksdorp, South Africa; ^15^University of Cape Town, Department of Medicine, Wellcome Centre for Infectious Disease Research in Africa, Cape Town, South Africa; ^16^Hanoi Medical University, Department of Infectious Diseases, Hanoi, Viet Nam; ^17^Duke University, Division of Infectious Diseases and International Health, School of Medicine, Durham, NC, United States; ^18^Pham Ngoc Thach University of Medicine, Tropical Medicine Research Center for Talaromycosis in Vietnam, Ho Chi Minh City, Viet Nam; ^19^Médecins Sans Frontières, South African Medical Unit, Johannesburg, South Africa; ^20^City St George's, University of London, Infection and Immunity Research Institute, School of Health and Medical Sciences, London, United Kingdom; ^21^City St George's, University of London, Centre for Healthcare Innovation, School of Health and Medical Sciences, London, United Kingdom; ^22^University of Cape Town, Department of Medicine, Cape Town, South Africa; ^23^London School of Hygiene and Tropical Medicine, Department of Clinical Research, Faculty of Infectious and Tropical Diseases, London, United Kingdom; ^24^Botswana Harvard Health Partnership, Gaborone, Botswana; ^25^University College London, Institute for Global Health, London, United Kingdom; ^26^Liverpool School of Tropical Medicine, Department of Clinical Sciences, Liverpool, United Kingdom; ^27^University of Cape Town, Institute of Infectious Disease and Molecular Medicine, Cape Town, South Africa; ^28^Queen Mary University of London, Blizard Institute, Faculty of Medicine and Dentistry, London, United Kingdom; ^29^University of Exeter, MRC Centre for Medical Mycology, Exeter, United Kingdom

**Background**: Pre‐emptive fluconazole treatment for HIV‐associated cryptococcal antigenaemia reduces the risk of cryptococcal meningitis and death, yet mortality in this group remains substantially higher than in CD4‐matched cryptococcal antigen (CrAg)‐negative individuals. We assessed whether an enhanced oral regimen of fluconazole plus flucytosine could reduce all‐cause mortality among adults with cryptococcal antigenaemia.

**Methods**: We performed a phase‐III open‐label randomized‐controlled superiority trial at 11 sites in South Africa and Tanzania, 2022−2025. Adults living with HIV with CD4 <100 cells/µL and newly diagnosed cryptococcal antigenaemia were randomized to 14 days of either: (i) fluconazole (1200 mg/day) plus flucytosine (100 mg/kg/day) (intervention) or (ii) fluconazole alone (1200 mg/day) (control). All participants were either cerebrospinal fluid CrAg‐negative at enrolment or declined lumbar puncture (with no meningitis symptoms). Consolidation fluconazole (800 mg/day) was administered for 8 weeks, thereafter 200 mg/day. The primary endpoint was 6‐month all‐cause mortality. Baseline CrAg titre and semi‐quantitative (SQ)‐scores were available in South Africa.

**Results**: Of 610 randomized participants, 6 met late exclusion criteria with 604 in the intention‐to‐treat (ITT) analysis. One participant was lost to follow‐up. Six‐month all‐cause mortality was 13% (40/304) in the intervention arm and 17% (50/300) in the control arm (risk ratio [RR] 0.79; 95% CI 0.54−1.16; *p* = 0.23). Results by country, per protocol and key sub‐groups are shown in Table 1. Six‐month all‐cause mortality in the CrAg titre ≥640 group was 6% (3/50) in the intervention arm and 19% (8/42) in the control arm (RR 0.32; 95% CI 0.09−1.11; *p* = 0.07). Results by SQ‐score are presented in Table 1 and Figure 1. The intervention was well tolerated with 178 serious adverse events (SAEs) among 97/304 (32%) participants versus 188 SAEs among 85/300 (28%) participants in the control arm, *p* = 0.37.

**TABLE 1:**
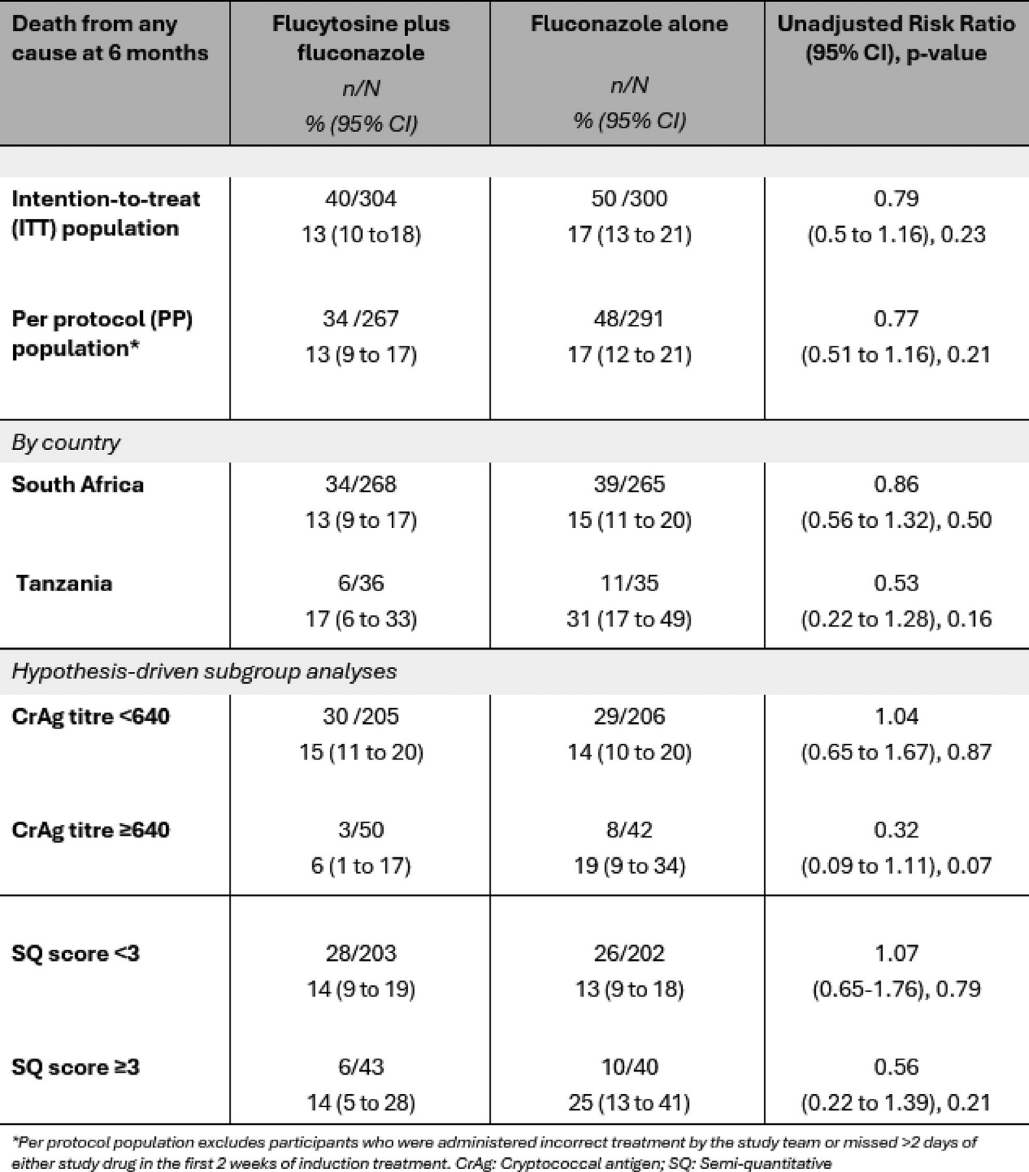
OAB4106LB | Summary of primary intention‐to‐treat, per protocol and sub‐group outcomes by study arm.

**FIGURE 1:**
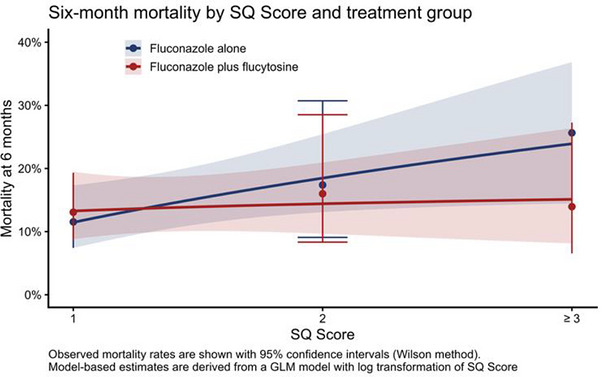
OAB4106LB

**Conclusions**: Combination treatment was not statistically superior to fluconazole in participants with antigenaemia without meningitis. In hypothesis‐driven sub‐group analyses, we observed a trend of increasing efficacy among participants with higher baseline CrAg titres/SQ scores although numbers were small. These groups could be targeted for a safe enhanced antifungal regimen in CrAg screening.

## OAC0206LB Molecular HIV Surveillance During Fiji's HIV Epidemic

D. Balak ^1^, J. Singh^1^, J. Wapling^2^, S. Butterly^3^, J. Gare^4^, M. Starr^3^, M. Hodgson^3^, J. Reddy^1^, J. Mitchell^1^, B. Obeng^2^, A. Kelleher^2,3^, P. Cunningham^3,2^, A. Kelly‐Hanku^2,4,5^

^1^Fiji Ministry of Health and Medical Services, SRH and HIV Unit, Suva, Fiji; ^2^Kirby Institute, UNSW Sydney, Sydney, Australia; ^3^NSW HIV Reference Laboratory, St Vincent's Centre for Applied Medical Research, St Vincent's Hospital, Sydney, Australia; ^4^Papua New Guinea Institute of Medical Research, Goroka, Papua New Guinea; ^5^The Fiji Institute of Pacific Health Research, Fiji National University, Suva, Fiji

**Background**: Fiji has the fastest growing HIV epidemic globally, increasing more than 30‐fold since 2010. An outbreak was declared in January 2025. Unsafe injecting drug use plays a key role, with people who inject drugs (PWID) accounting for 48% of people living with HIV (PLHIV) starting antiretroviral therapy (ART). To inform the national public health response, an HIV‐1 drug resistance (DR) and phylogenetic study was undertaken. This study is the only molecular surveillance of the HIV epidemic in Fiji and the first to include PWID as a key population.

**Methods**: A prospective study was conducted at Suva Sexual and Reproductive Health Clinic from February 2024 to July 2025. 198 consenting adult PLHIV provided dried blood spots for testing at the WHO‐accredited HIV‐1 DR Reference Laboratory in Australia. Protease‐reverse transcriptase (PR‐RT) and integrase (INT) regions were analysed for DR and PR‐RT for phylogenetic analyses.

**Results**: Of 198 samples, 110 (55.5%) were successfully sequenced and were representative of national epidemiological data with 93.6% from i‐Taukei (Indigenous Fijians), 78.2% male and 53.0% reported injecting drug use. Major HIV subtypes were C (46%) and CRF02_AG (50%). Pre‐treatment DR (PDR) (*n* = 107) was 0.9% for PIs, 2.8% for NRTIs, 20.6% for NNRTIs. INSTI DR (R263K) was observed in one individual (0.9%). Phylogenetic analysis identified three clusters with >10 PLHIV, all of which included PWID. The largest cluster (*n* = 47) included 33 PWID (including one PWID + men who have sex with other men), 2 sexual partners of PWID and 13 general population, inferring both injecting and sexual transmission within clusters.

**Conclusions**: With low rates of PDR, the identification of resistance to dolutegravir in a newly diagnosed person within 3 years of transitioning to INSTI‐based regimens is concerning and highlights the importance of sustaining PLHIV on ART. Phylogenetic analysis demonstrates large, highly connected networks driven by unsafe injecting practices. Access to sterile needles, syringes and injecting equipment would have played a key role in limiting HIV transmission Fiji. Unless harm reduction programmes such as needle and syringe programmes are fast tracked, Fiji's epidemic will continue apace.

## OAC0706LB HIV in the Triple Elimination Strategy of Mother to Child Transmission (Trimom Study): An Overview of Transmission and Post‐Partum Follow Up in The Gambia

K. Jammeh ^1^, D. Seo^1^, A. E. Jallow^1^, B. Dibba^1^, S. Drammeh^1^, E. Vo Quang^2^, G. Ndow^1^, M. Lemoine^3,1^, S. Boyer^4^

^1^Medical Research Council Unit The Gambia at LSHTM, Disease Control and Elimination (Hepatitis Research Group), Fajara, Gambia, the; ^2^L'Universite Paris Est Créteil, Paris, France; ^3^Imperial College London, Department of Metabolism, Digestion and Reproduction, London, United Kingdom; ^4^Aix Marseille Universite, INSERM, IRD, SESSTIM, Sciences Economiques & Sociales de la Santé & Traitement de l'Information Médicale, Marseille, France

**Background**: Sub‐Saharan Africa bears one of the highest burdens of vertical transmission of HIV, syphilis and hepatitis B virus (HBV). The WHO has proposed an integration of interventions into a “triple elimination” model to accelerate the elimination vertical transmission of HIV, syphilis and HBV in endemic areas. Vertical transmission of HIV accounts for nearly 9% of new acquisitions worldwide and significantly contributes to the HIV epidemic. In the absence of antiretroviral treatment, 15%−30% of children born to highly viraemic mothers living with HIV will acquire HIV during childbirth, and 5%−15% during the early nursing period.

**Methods**: Screening of pregnant women was conducted at four facilities in different regions of The Gambia. Treatment was initiated for all women diagnosed with HIV and syphilis, and women positive for HBV, meeting the WHO treatment criteria. The women were monitored during routine antenatal care visits until delivery. In addition, mothers and babies are followed up till 6 months postpartum. Collecting screening results of babies for HIV and HBV and assessing for features of congenital syphilis.

**Results**: From March 2024 to November 2025, 13,029 pregnant women were screened. 644 tested positive for any of the three conditions and 512 consented to enrolment into the follow up cohort. 164 women tested positive for HIV only, 434 for HBV only, and for 29 syphilis only, 12 were positive for HIV and HBV and 5 for HIV and syphilis. 123 women with HIV only were enrolled (75%), and ARV uptake was 95.9% (118/123). As of March 2026, 71 women with HIV and their babies are followed up into the postpartum period. All babies were started on Nevirapine syrup and the first infant screening results at 6−8 weeks were received for 46 babies. Two children tested positive (4.3%).

Category number of women% of total screened (13,029) (Table 1)

**TABLE 1:** OAC0706LB

Total pregnant women screened	13,029	100%
Any positive for HIV, HBV or syphilis	644	≈4.9%
HIV monoinfections	164	1.3%
HBV monoinfections	434	3.4%
Syphilis monoinfections	29	0.2%
HIV–HBV coinfections	12	0.1%
HIV–syphilis coinfections	5	0.04%

**Conclusions**: A simplified integrated triple elimination strategy is feasible in sub‐Saharan African countries. ART for mothers and ARV prophylaxis adherence with follow up in babies can end vertical transmission. Infant screening still requires strengthening.

## OAC1306LB Interim Analysis of Adverse Pregnancy Outcomes With Injectable CAB‐LA Versus Oral PrEP: Findings From the PrIMO Safety Cohort

F. Saidi ^1,2,3^, M. A. Squibb^2^, A. Masano^1^, L. Chinula^1,2,3^, C. Nakanga^1^, T. Mvalo^1,2^, M. Matoga^1^, A. K. Bula^1^, M. B. Chagomerana^1,2^, T. Phanga^1^, W. Kumwenda^1^, T. Mkochi^1^, G. Masiye^1^, J. Tsakama^1^, W. Nyirenda^1^, D. Makonokaya^1^, P. Dias^1^, B. Milala^1^, Z. Tembo^1^, A.‐G. Tepeka^1^, L. Chimpukuso^1^, M. E. Herce^2^, S. E. Rutstein^2^, V. Thonyiwa^4^, R. K. Nyirenda^5^, W. Ozitiosauka^6^, I. Hoffman^1,2^, M. C. Hosseinipour^1,2^, PRISM Study Group

^1^University of North Carolina Project Malawi, Lilongwe, Malawi; ^2^University of North Carolina at Chapel Hill, Chapel Hill, United States; ^3^Kamuzu University of Health Sciences, Obstetrics and Gynecology, Lilongwe, Malawi; ^4^United States Government Department of State, President's Emergency Plan for AIDS Relief, Lilongwe, Malawi; ^5^World Vision Malawi, Lilongwe, Malawi; ^6^Ministry of Health Malawi, Department of HIV, STI and Viral Hepatitis, Lilongwe, Malawi

**Background**: Pre‐exposure prophylaxis (PrEP) reduces incident HIV acquisitions and is a promising tool in the prevention of vertical transmission. Oral PrEP (FTC/TDF, 3TC/TDF) is safe during pregnancy and breastfeeding, but safety data on long‐acting injectable cabotegravir (CAB‐LA) are limited. The Pregnancy, Maternal, and Infant Outcomes (PrIMO) study was established to generate robust safety data as global access expands. The study, currently enrolling at Bwaila District Hospital in Lilongwe, Malawi, will follow 621 mother−infant pairs through 1 year postpartum.

**Methods**: Interim analyses, conducted after approximately 50% of births had occurred, included 362 women with pregnancy outcomes observed through 5 February 2026. Women were enrolled during pregnancy. Those already taking PrEP continued their regimen, while others chose between initiating oral PrEP or CAB‐LA. Participants attended monthly ANC visits according to Malawi National Guidelines and continue to attend postpartum visits with their infants through 52 weeks. The primary outcome is composite adverse pregnancy outcome, defined as the occurrence of any of the following: preterm birth (<37 weeks gestation), small for gestational age (birthweight <10th percentile for gestational age), stillbirth (≥28 weeks gestation) or spontaneous miscarriage (<28 weeks gestation). We estimated risk ratios and 95% confidence intervals (CI) using unadjusted Poisson regression models with robust variance estimators.

**Results**: 223 of 362 women (62%) chose CAB‐LA. Maternal demographics were similar between CAB‐LA versus oral PrEP groups, with median ages of 25.9 (IQR 22.2−30.2) and 25.5 (IQR 21.5−29.8), respectively. The majority of women in both groups were married (89% vs. 92%) and had a previous pregnancy (77% vs. 80%). Pregnancy outcomes and adverse events are presented in Table 1. The estimated relative risk of any adverse outcome comparing CAB‐LA to oral PrEP was 1.43 (95% CI: 0.70−3.15; *p* = 0.3).

**TABLE 1:** OAC1306LB | Pregnancy outcomes and adverse events included in composite measure.

Pregnancy outcomes	Oral PrEP *N* = 139	CAB‐LA *N* = 223	Relative risk (95% CI)
Non‐adverse event, full‐term live birth (≥ 37 weeks)	129 (93%)	200 (90%)	1 (Ref)
Composite adverse pregnancy outcome	10 (7.1%)	23 (10.3%)	1.43 (0.70, 3.15)
Small for gestational age (<10th percentile)	2 (1.4%)	1 (0.4%)	
Premature live birth (< 37 weeks)	5 (3.5%)	12 (5.4%)	
Spontaneous foetal death and/or still birth (≥ 28 weeks)	2 (1.4%)	7 (3.1%)	
Spontaneous abortion (< 28 weeks)	1 (0.7%)	3 (1.3%)	

**Conclusions**: Although CAB‐LA was associated with a higher relative risk of adverse pregnancy outcomes compared to oral PrEP, this difference was not statistically significant at interim analysis. As long‐acting HIV prevention expands globally, generating robust pregnancy safety data is imperative. Dedicated safety cohorts such as PrIMO are essential to ensure that pregnant women are not left behind in biomedical innovation.

## OAC1906LB The First 6 Months of National Injectable Lenacapavir PrEP Prescribing and Equity, United States, June−December 2025

A. Siegler ^1^, E. Koh^1^, E. Corbin‐Gutierrez^2^, X. Zang^3^, P. Sullivan^1^

^1^Emory University, Epidemiology, Atlanta, United States; ^2^NASTAD, Washington, DC, United States; ^3^University of Minnesota, Health Policy and Management, Minneapolis, United States

**Background**: Biannual injectable lenacapavir PrEP was approved by FDA in June 2025. Surveillance is critical to optimize HIV prevention policy and programmes.

**Methods**: Using a national medical and pharmacy claims database that captures a substantial majority of US PrEP claims, we assessed use of the two oral tenofovir‐based PrEP regimens and injectable lenacapavir (LEN) and cabotegravir (CAB) regimens by sociodemographics, insurance and copay amount. Use was defined as ≥1 prescription during the period; regimen groups were not mutually exclusive. We calculated PrEP‐to‐need ratios (PnR) as ratios of PrEP prescriptions to new HIV diagnoses. PrEP equity ratios (PER) are ratios of PnR between two groups, facilitating comparison across regimens and with a value of one indicating perfect equity.

**Results**: From 01 June 2025 to 31 December 2025, there were 7134 LEN users, 31,868 CAB users and 533,878 oral PrEP users (Table 1). LEN users were similar to other PrEP users in age and race/ethnicity. There were more females among LEN users than among oral PrEP users. For both LEN and other regimens, PrEP equity in terms of PER was lowest for Black and Hispanic persons. PER indicate that LEN was provided more equitably for females than oral PrEP and less equitably for younger persons. Substantially more LEN users were covered by Medicaid (28%) than were oral PrEP users (12%). Copays over $100 were more common for LEN (5%) than for oral PrEP regimens (1%).

**TABLE 1:** OAC1906LB

	LEN *n*	LEN %	LEN PER	CAB *n*	CAB %	CAB PER	Oral PrEP *n*	Oral PrEP %	Oral PER
Male	6045	85%	Ref	27,381	86%	Ref	487,271	91%	Ref
Female	1089	15%	0.78	4487	14%	0.71	46,603	9%	0.41
Black	1186	17%	0.17	5934	19%	0.20	80,737	16%	0.15
Hispanic	1213	17%	0.19	5675	18%	0.21	84,743	16%	0.17
White	4233	61%	Ref	18,076	58%	Ref	328,293	63%	Ref
Others	361	5%	—	1398	5%	—	26,361	5%	—
Age ≤ 24	558	8%	0.31	2554	8%	0.32	48,961	9%	0.39
Age 25−34	2208	31%	0.61	11,326	36%	0.70	184,584	35%	0.74
Age 35−44	2243	31%	Ref	10,043	32%	Ref	156,123	29%	Ref
Age ≥ 45	2125	30%	0.98	7945	25%	0.81	144,210	27%	0.95

**Conclusions**: In the first 6 months of US availability, LEN accounted for about 1% of all PrEP use. In this short period, disparities emerged that align with longstanding PrEP disparities across all regimens—race/ethnicity, sex and age. Higher LEN use by Medicaid users and higher rates of copay >$100 likely indicate that insurance‐related barriers hinder LEN use among commercially insured persons. Addressing structural and financial barriers to LEN will be critical for scaleup.

## OAC2806LB High Efficacy of Twice‐Yearly Subcutaneous Lenacapavir for PrEP Through 52 Weeks of Open‐Label Extension in PURPOSE 2

M. Losso ^1^, B. Santos^2^, J. R. Lama^3^, K. H. Mayer^4^, A. Avihingsanon^5^, N. Ndlovu^6^, N. Shah^7^, O. van Gerwen^8^, S. Cox^9^, L. B. Brown^9^, C. C. Carter^9^, P. Wong^9^, P. Arora^9^, J. Pilotto^10^, B. Grinsztejn^11^

^1^Hospital General de Agudos José María Ramos Mejía, Buenos Aires, Argentina; ^2^Hospital Nossa Senhora da Conceição, Grupo Hospitalar Conceição, Porto Alegre, Brazil; ^3^Asociación Civil Impacta Salud y Educación (IMPACTA Perú), Lima, Peru; ^4^The Fenway Institute, Fenway Health, Harvard Medical School, Boston, MA, United States; ^5^HIVNAT, Thai Red Cross AIDS and Infectious Diseases Research Centre and Center of Excellence in Tuberculosis; Chulalongkorn University, Bangkok, Thailand; ^6^Wits Reproductive Health and HIV Institute (Wits RHI), University of the Witwatersrand, Johannesburg, South Africa; ^7^Whitman‐Walker Health, Washington, DC, United States; ^8^University of Alabama at Birmingham, Heersink School of Medicine, Birmingham, AL, United States; ^9^Gilead Sciences, Inc., Foster City, CA, United States; ^10^Hospital Geral de Nova Iguacu & Instituto Oswaldo Cruz (Fiocruz), Rio de Janeiro, Brazil; ^11^Instituto Nacional de Infectologia Evandro Chagas, Fundação Oswaldo Cruz (Fiocruz), Rio de Janeiro, Brazil

**Background**: In the randomized blinded phase (RBP) of PURPOSE 2 (NCT04925752), twice‐yearly subcutaneous lenacapavir (LEN) for HIV pre‐exposure prophylaxis (PrEP) demonstrated superior efficacy to daily oral emtricitabine/tenofovir disoproxil fumarate (F/TDF) in cisgender men and gender‐diverse individuals. Following the RBP, participants could choose to receive LEN in an open‐label (OL) LEN extension phase. We report HIV incidence, safety, adherence to injections with OL LEN, and cumulative HIV incidence from study start for participants receiving LEN through 52 weeks of OL LEN follow‐up.

**Methods**: At the final RBP visit, PURPOSE 2 participants on randomized study drug were offered OL twice‐yearly subcutaneous LEN. We assessed HIV incidence in participants who received LEN in any study phase through OL LEN Week 52, and LEN plasma levels were measured in participants who seroconverted. Adverse events were assessed at all study visits. Adherence was defined as LEN administration within 28 weeks after the last injection.

**Results**: Through 24 March 2025, 99% of participants eligible to join the OL LEN phase completed the final RBP visits and 95% elected to initiate or continue LEN. From trial start, there were four incident HIV acquisitions in participants on LEN over 5295 person‐years (PY) of follow‐up (incidence 0.08/100 PY; 95% CI: 0.02−0.19); three HIV acquisitions occurred during the RBP (previously reported) and one HIV acquisition occurred on OL LEN (Figure 1). The participant who acquired HIV during the OL phase received all LEN injections on time and LEN plasma level assessment is ongoing. The incidence and severity of injection site reactions were similar with OL LEN to that observed to the LEN arm during the RBP. Adherence to OL LEN injections was 96% and 92% at Weeks 26 and 52, respectively, among participants switching from active F/TDF.

**FIGURE 1:**
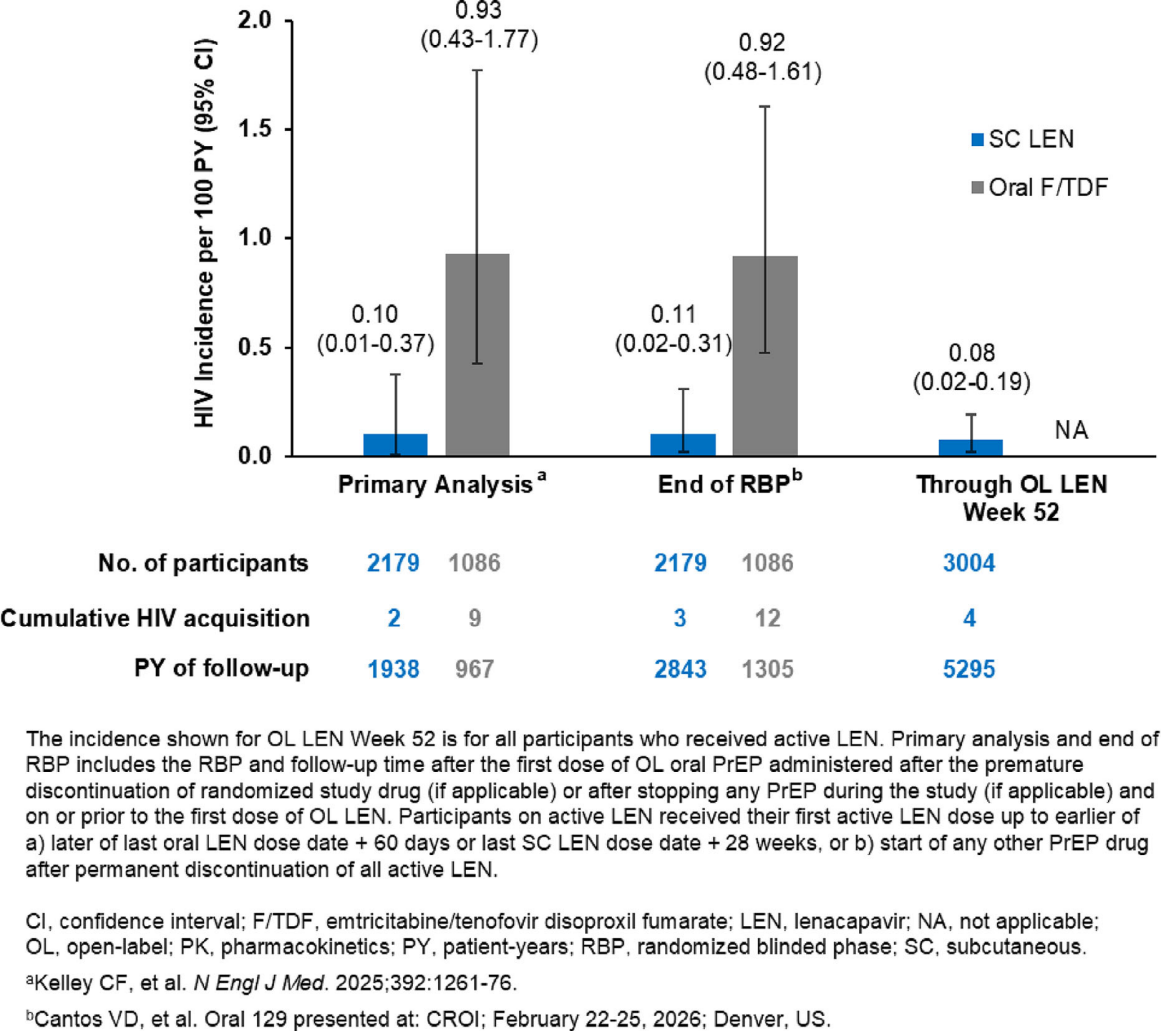
OAC2806LB | HIV incidence in PURPOSE 2 over time.

**Conclusions**: Twice‐yearly LEN continues to demonstrate high efficacy among participants who chose to initiate or continue OL LEN after the RBP. This, in combination with high adherence and a consistent safety profile, reinforces LEN as a transformative PrEP option for cisgender men and gender‐diverse people globally.

## OAD0406LB Social Network Disruption Among Sexual Minority Men Living With HIV Following Conflict‐Induced Displacement: A Longitudinal Cohort Study

S. Tomar^1^, B. Skaathun^2^, M. Filippovych^3^, C. Fang^4^, J. Wertheim^2^, I. Koutsenok^5^, P. Smyrnov ^3^, S. Friedman^6^, T. Vasylyeva^1^

^1^University of California Irvine, Population Health and Disease Prevention, Irvine, United States; ^2^UC San Diego, Division of Infectious Diseases and Global Public Health, School of Medicine, San Diego, United States; ^3^Alliance for Public Health, Kyiv, Ukraine; ^4^University of California San Diego, La Jolla, United States; ^5^UC San Diego, Department of Psychiatry, San Diego, United States; ^6^New York University, Department of Population Health, Grossman School of Medicine, New York, United States

**Background**: Conflict‐induced displacement threatens the social networks of people, including sexual minority men (SMM) living with HIV, potentially undermining support and increasing exposure to stigmatizing attitudes. Social connectedness is a known protective factor for health and wellbeing among people living with HIV. This study examined trajectories of social network disruption among displaced and non‐displaced SMM in Ukraine following the Russia−Ukraine war.

**Methods**: A longitudinal cohort of 82 SMM living with HIV in Kyiv and L'viv (26 displaced) was followed across two time points—baseline (2024−2025) and 6‐month follow‐up (2025−2026)—with retrospective pre‐war (2022) data collected at baseline. Egocentric network data collected from SMM (“ego”), about people in their networks (“alters”), were analysed for trends in degree (number of alters) across total, support and stigma networks. Group differences were compared using Wilcoxon tests.

**Results**: Displaced individuals began with higher pre‐war network degree (4.0 vs. 3.2), lost on average 3.5 alters (89% of their network) and gained on average 2.8 new alters by baseline, resulting in baseline degree of 3.3. Local SMM lost 1.45 alters (46% of their network) and gained 1.71 new alters by baseline, resulting in a baseline degree of 3.4. By 6‐month follow‐up, the degree for displaced SMM networks further reduced to 2.7 (vs. 3.0 for non‐displaced). Wilcoxon tests confirmed significantly greater network size decline from pre‐war to current (−0.69 vs. +0.27, *p* < 0.01) and pre‐war to follow‐up (−1.27 vs. −0.14, *p* < 0.05) in displaced SMM. Support network patterns were similar. Stigma networks also declined over time for both groups, with displaced SMM showing significantly greater reduction from pre‐war to follow‐up (−0.77 vs. −0.20, *p* < 0.01).

**Conclusions**: Conflict‐induced displacement severely disrupts the social networks of SMM living with HIV, with network size contracting by nearly 90% at peak disruption. Although displaced individuals forge new connections over time, their networks do not fully recover to pre‐war levels. While displacement does sever ties with stigmatizing contacts, the concurrent loss of supportive relationships carries serious downstream consequences for HIV‐related health and wellbeing. These findings highlight an urgent need to embed peer support interventions within humanitarian responses for displaced populations (see Figure 1a,b).

**FIGURE 1:**
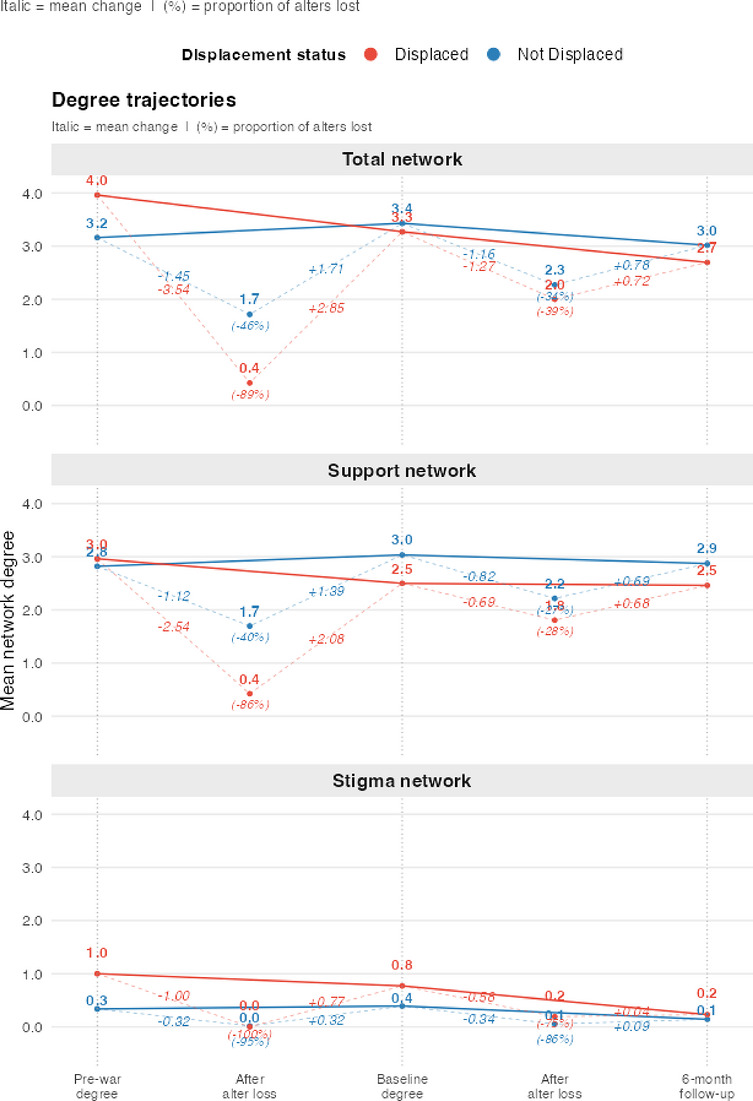
OAD0406LB | (a) Mean network degree—Trajectories by displacement status, (b) Mean network degreeChange between periods.

**FIGURE 1:**
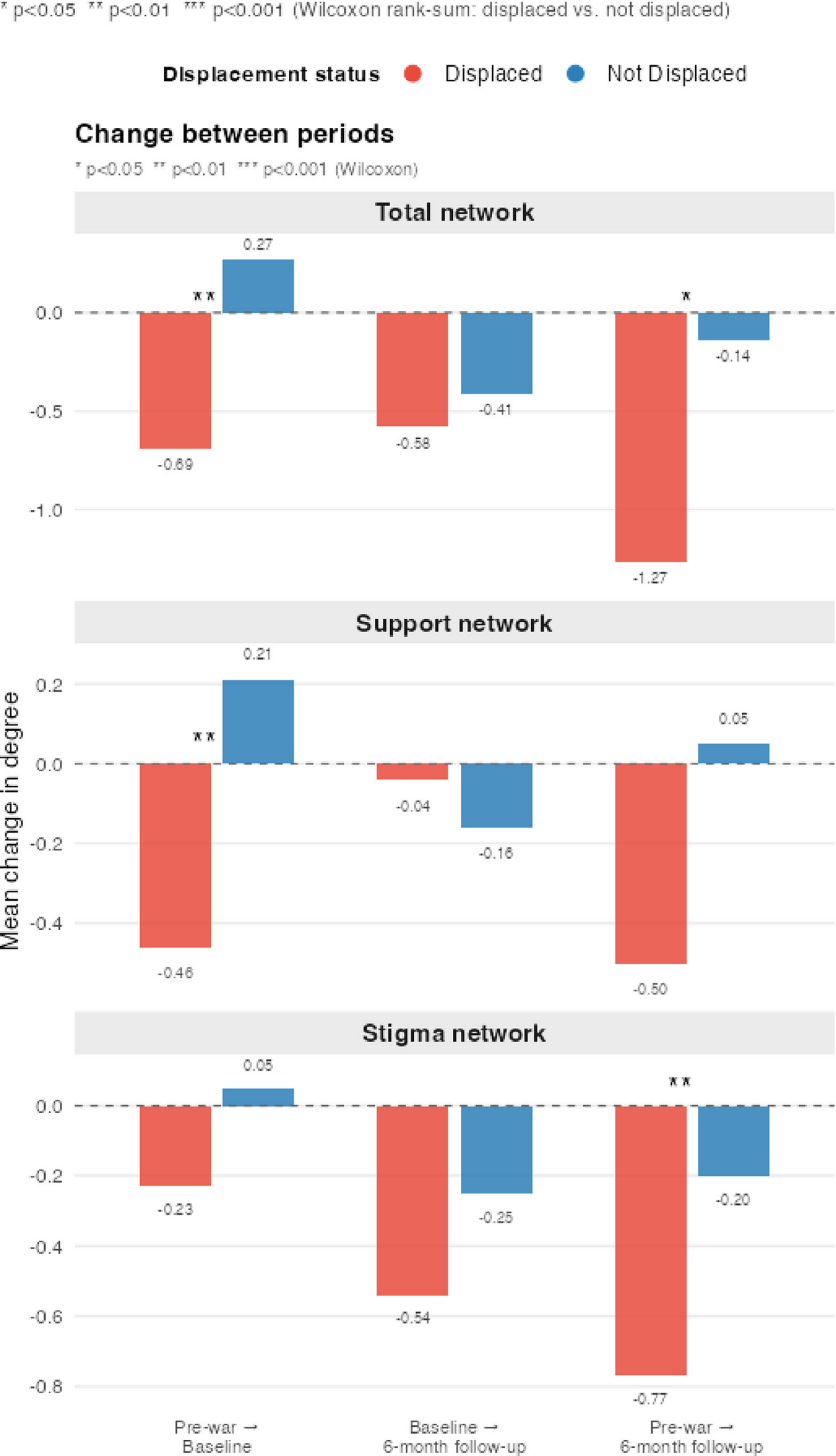
OAD0406LB | (Continued).

## OAD2106LB Persistent Internalized HIV Stigma Among Persons Living With HIV in Jamaica and Implications for Engagement in Care in the Context of Shifting Funding Priorities

S. Jarrett ^1^, J. Patrick^2^, J. Williams^1^, J.‐A. Nugent^2^, T. Ellis^1^

^1^Caribbean Vulnerable Communities, Kingston, Jamaica; ^2^Jamaica Network of Seropositives, Kingston, Jamaica

**Background**: HIV‐related stigma in Jamaica undermines quality‐of‐life and engagement in care and treatment among persons living with HIV (PLHIV). These analyses examined patterns of internalized HIV stigma and stigma‐related behaviours affecting care and treatment.

**Methods**: We examined internalized stigma among 836 PLHIV participating in the 2025 Jamaica HIV Stigma Index. Participants were interviewed in clinical and community settings. Descriptive analysis summarized the sample while chi‐square analyses explored differences in internalized stigma by demographic characteristics, key population membership, time since HIV diagnosis and support group membership.

**Results**: Among participants, 53% identified as female, 44% as male and 2% as transgender or nonbinary. Nearly half (49%) had lived with HIV for 10 years or more, over one‐third belonged to key populations, including men‐who‐have‐sex‐with‐men (MSM) (15%) and persons who had ever sold sex (13%). Almost half (46%) participated in HIV support groups, ranging from 28% among persons diagnosed within 1−3 years to 75% among cisgender women who had ever sold sex.

Overall, 38% reported at least one indicator of internalized HIV stigma. Feelings of worthlessness were more common among cisgender women than cisgender men (16.3% vs. 9.9%; X^2^ = 7.075, *p* < 0.01). Bisexual participants, cisgender women who had ever sold sex and MSM were more likely than non‐key population participants to hide their HIV status (90%, 90%, 89% vs. 81%, respectively; X^2^ = 8.973, *p* < 0.05) and report guilt related to HIV status (40%, 29%, 36% vs. 25%; X^2^ = 8.094, 3, *p* < 0.05). PLHIV not involved in support groups reported higher guilt (35% vs. 19%; *p* < 0.01), shame (*p* < 0.01), worthlessness (17% vs. 10%; *p* < 0.05) and feeling dirty (17% vs. 8%; *p* < 0.01).

Participants also reported self‐isolating behaviour due to their HIV status, including avoiding relationships, social support and healthcare services. Fear of disclosure to family and friends was reported as a reason for delaying treatment initiation (25%) and treatment interruption (30%).

**Conclusions**: Internalized stigma is common among PLHIV in Jamaica, including persons living long‐term with HIV and those outside of support groups and networks. As HIV funding decreases, we must balance prioritizing biomedical treatment with maintaining investment in psychosocial interventions that can reduce internalized stigma and sustain engagement in care and treatment.

## OAD3306LB Rewiring Risk: A Cognitive Dissonance‐Based Peer‐Led Intervention for HIV Prevention Behaviour Change Among Young Women in Wassa IDP Camp in Abuja, Nigeria

C. Nwagbo ^1^, D. Nweze^2^, I. Aliu^3^, B. Agi^2^, E. Tor^4^

^1^Beam Community Development 360 Initiative, Programmes, Abuja, Nigeria; ^2^Beam Community Development 360 Initiative, Monitoring and Evaluation, Abuja, Nigeria; ^3^Beam Community Development 360 Initiative, Programmes, Karu, Nigeria; ^4^Achieving Better Life Initiative and Empowerment for Women and Young Persons, Programmes, Abuja, Nigeria

**Background**: Young women in internally displaced persons’ (IDP) settings experience disproportionate HIV vulnerability, compounded by low‐uptake of prevention tools and persistent intention‐behaviour gaps. Baseline surveillance in Wassa IDP camp showed low consistent condom‐use (27.5%) and prevalent misconceptions regarding long‐acting injectable pre‐exposure prophylaxis (LAI‐PrEP). We evaluated the implementation outcomes and behavioural effects of peer‐led cognitive dissonance activation intervention designed to improve condom use and LAI‐PrEP readiness among young women in Wassa IDP.

**Description**: From September 2024 to December 2025, 260 sexually active young‐women aged 18−25 years (90.8% consent) in Wassa IDP camp; 26 groups (mean size = 10/group) participated in a 13‐session group‐based, peer‐led cognitive dissonance intervention delivered in stepped‐wedge rollout waves (mean size = 3 groups per month), adjusting for time‐steps and clustering (ICC = 0.06; design effect = 1.54; σ^2^ = 0.12); ICC estimates were stable across time periods (range: 0.05−0.07). Trained community facilitators implemented 60‐min standardized sessions composed of values‐behaviour discrepancy reflection, myth‐challenging dialogue, condom negotiation role‐play, and condoms and LAI‐PrEP education by demonstration. Fidelity was monitored using adapted Body‐Project adherence facilitator checklist, and participant session checklist. Outcomes of cognitive‐dissonance recognition, HIV prevention knowledge, condom use self‐efficacy, misconceptions and readiness for LAI‐PrEP uptake using validated dissonance awareness log, KAP survey, and analysed using mixed‐effects models accounting for clustering (ICC) and time‐step effects, for the evaluation.

**Lessons learned**: Stepped‐wedge analysis showed 75.5% rise of cognitive dissonance activation (*aRR *= 1.76; 95% CI: 1.38−2.24). HIV prevention knowledge significantly improved *aRD *= 37.4pp (95% CI; 30.2−44.6; *p* < 0.001), 81% decline in LAI‐PrEP misconceptions (*aRR *= 0.19; 95% CI: 0.11−0.34; *p* < 0.0001). Consistent condom‐use improved by 62.9% (*aRR *= 1.63; 95% CI; 1.24−2.14; *p* < 0.001). Readiness for LAI‐PrEP increased from 40.0% to 79.9% (*aRR *= 2.00; 95% CI: 1.47−2.72; *p* < 0.001). Biological outcome validation showed 41% reduction in incidence of Chlamydia (*aIRR *= 0.59, 95% CI: 0.36−0.96; *p* < 0.05). High session fidelity (88.2%) and strong peer diffusion (89.5%) via sociometric nomination. These findings suggest meaningful behavioural improvements.

**Conclusions/Next steps**: Peer‐led cognitive dissonance activation improved consistent condom‐use and LAI‐PrEP readiness among young women in IDP setting.

## OAD3606LB Effects of an AI‐Driven Intervention Based on Multimodal Fusion Machine Learning on Health Outcomes in People Living With HIV: A Randomized Controlled Trial

L. Zhang ^1^, H. Huang^1^, Q. You^1^, H. Chen^1^

^1^Sichuan University, School of Nursing, Chengdu, China

**Background**: Mobile health interventions have shown promise in supporting people living with HIV, yet limitations remain. This study developed an AI‐enhanced management platform based on multimodal fusion machine learning within a WeChat mini‐programme and evaluated its effectiveness on health‐related outcomes.

**Methods**: A randomized controlled trial was conducted among 100 people living with HIV recruited from two designated hospitals in Sichuan Province. Participants were randomly assigned to an intervention group (*n* = 50) or a control group (*n* = 50). The control group received routine follow‐up care, while the intervention group additionally used the AI‐driven WeChat mini‐programme developed based on the Supportive Care Needs Framework, Chronic Care Model and Behavior Change Wheel. The platform included modules for health education, consultation, reminders, medical information recommendations and intelligent Q&A. Data were collected at baseline (T0), 1 month (T1) and 3 months (T2), including self‐management behaviours, medication adherence, disease knowledge, quality of life and self‐efficacy. Statistical analyses included *t*‐tests, Mann–Whitney U tests, chi‐square tests and generalized estimating equations (GEE).

**Results**: Baseline characteristics were comparable between groups. At T1 and T2, the intervention group showed significantly higher self‐management behaviour scores, including disease knowledge management, symptom management and emotional–cognitive management (*p* < 0.05), with significant group, time and interaction effects.

Disease knowledge scores were also significantly higher in the intervention group at both follow‐ups (*p* < 0.001). Quality of life and self‐efficacy improved significantly in the intervention group compared with the control group (*p* < 0.05 and *p* < 0.001, respectively), with significant GEE effects.

No significant between‐group differences were observed in medication adherence (*p* > 0.05), although a significant time effect was found.

**Conclusions**: The AI‐driven mobile intervention demonstrated good acceptability and effectively improved self‐management, disease knowledge, quality of life and self‐efficacy among people living with HIV. However, its effect on medication adherence was not significant, warranting further investigation (see Figure 1).

**FIGURE 1:**
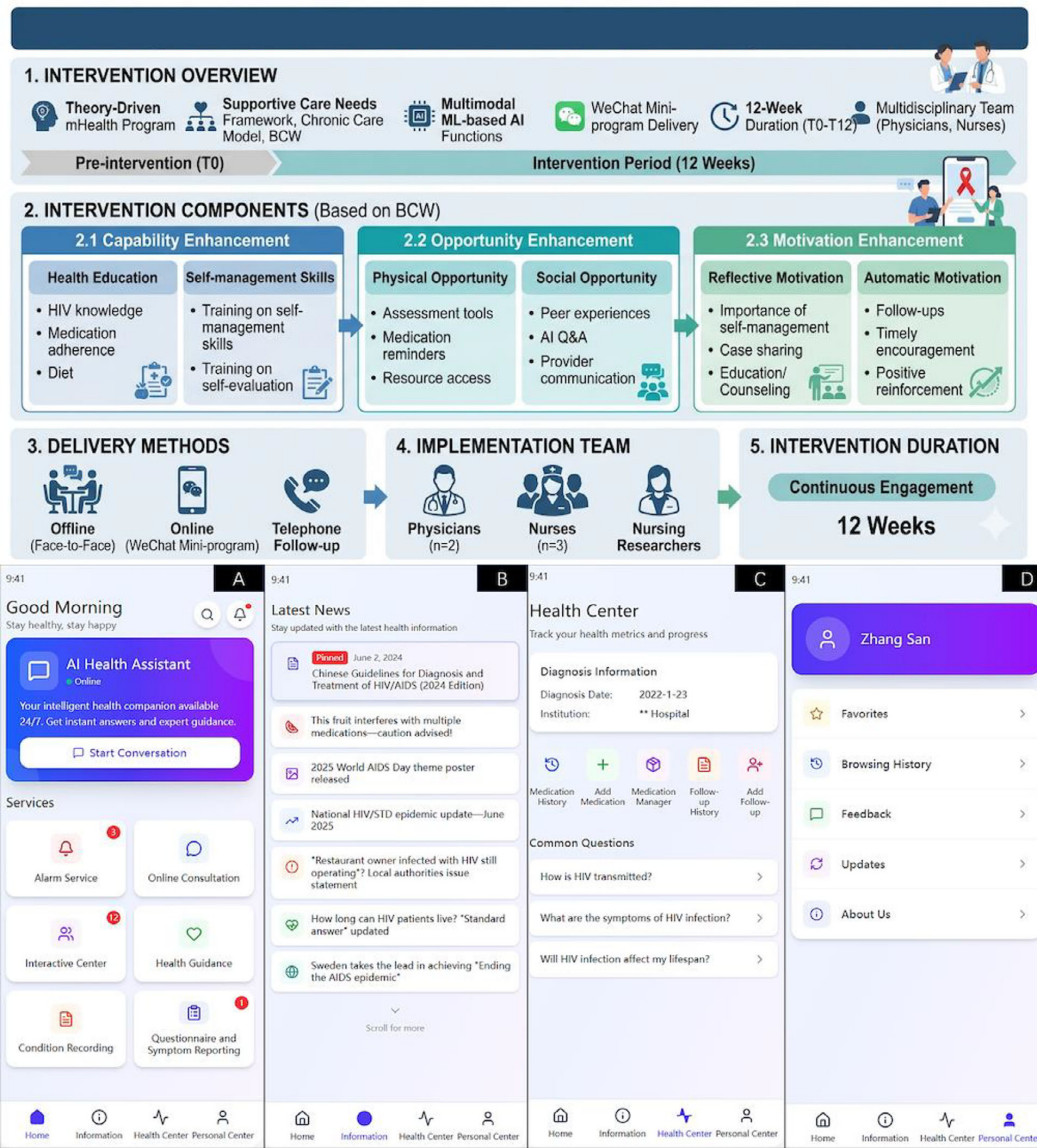
OAD3606LB | Overview of the 12‐week mHealth intervention programme for PLWH.

## OAD3906LB Anti‐LGBTQ+ Legislation, Mental Health and ART Non‐Adherence Among Sexual Minority Men, Trans Women and Gender‐Diverse People Living With HIV in Ghana: A Mediation Analysis

A. O. Gyamerah ^1^, P. Prado^2^, H. H. Leslie^3^, P. K. Ayeh^4^, N. A. Vanderpuye^4^, L. E. Nelson^5^, T. B. Neilands^3^, M. Lightfoot^6^, S. A. Lippman^3^

^1^University at Buffalo, Community Health and Health Behavior, Buffalo, United States; ^2^University of California, San Francisco, Center for AIDS Prevention Studies, San Francisco, United States; ^3^University of California, San Francisco, Division of Prevention Science, San Francisco, United States; ^4^West Africa AIDS Foundation, Accra, Ghana; ^5^Yale University, School of Nursing, New Haven, United States; ^6^OHSU‐PSU School of Public Health, Portland, United States

**Background**: Sexual minority stressors like anti‐LGBTQ+ legislation have been associated with adverse mental health and HIV outcomes among sexual and gender minorities (SGM) globally. We created the LGBTQ+ Criminalization Social Stressor Index (LCSS) to examine how social stressors experienced due to Ghana's anti‐LGBTQ+ legislation indirectly impact antiretroviral therapy (ART) through mental health (depression, alcohol use disorder, suicide risk) among sexual minority men (SMM), trans women and gender‐diverse people living with HIV (PLHIV).

**Methods**: We conducted a cross‐sectional survey with SMM, transgender women and gender‐diverse PLHIV (*N* = 256) in Accra, Ghana between April 2025 and January 2026. Descriptive analyses report means and frequencies of: socio‐demographics; depressive symptoms (CESD‐10), suicide risk (SBQ‐R), alcohol use disorder (AUDIT‐C); social support (MSPSS), and ART adherence. Guided by an intersectional sexual minority stress model, direct and indirect effects of LCSS—an index capturing frequency of 16 enacted social stressors (e.g. discrimination, violence) due to anti‐LGBTQ+ legislation (never, once, more than once; scored 0−32)—on ART adherence were examined, with mental health measures as mediators. Model covariates were social support, age, years living with HIV and years on ART. Mediation effects were assessed using lavaan SEM package in R (*p* < 0.05). See Table 1 for participant psychosocial and HIV‐related characteristics.

**TABLE 1:** OAD3606LB | Psychosocial and HIV‐related characteristics (*N* = 256).

	*n* (%)
**Median monthly income** (11 GHS = 1 USD), IQR	1200 GHS (700–2000 GHS)
**LGBTQ+ Criminalization Social Stressors (LCSS)**, mean score ± SD	6.9 ± 5.8
**Depressive symptoms** (CESD‐10), mean score ± SD	
Score ≥ 10 means significant depressive symptoms	11.7 ± 4.9
**At significant risk of suicide** (SBQ‐R ≥7)	71 (27.8%)
**Alcohol use disorder** (AUDIT‐C ≥4)	65 (25.5%)
**Social support (MSPSS), mean score ± SD**	3.4 ± 1.2
Low (1−2.9)	88 (34.5%)
Medium (3−5)	152 (59.6%)
High (5.1−7)	15 (5.9%)
**On ART**	243 (94.9%)
**Years since HIV diagnosis**, mean ± SD	5.8 ± 5.0
**Years since ART enrolment,** mean ± SD	5.2 ± 4.2
**ART non‐adherence**: missed ARV doses 4 days in a row within 3 months one or more times (*n* = 229)	71 (31.0%)

**Results**: Participant mean age was 30.7 years (SD = 7.5). Most identified as gay (44.5%) or bisexual (41.8%) and 41.0% identified as male, 44.9% as transgender female, and 14.1% as gender‐diverse. About 31% were non‐adherent to ART. LCSS was significantly associated with depressive symptoms (β = 0.45, *p* < 0.001), alcohol use disorder (β = 0.27, *p* = 0.005) and suicide risk (β = 0.56, *p* < 0.001). In path analysis (see Table 2), there was a significant indirect effect of LCSS on ART adherence via depressive symptoms (β = −0.10, *p* = 0.018), but not a significant direct effect (β = −0.03, *p* = 0.78).

**TABLE 2:** OAD3606LB | Mediation model predicting ART adherence (*n* = 229).

Path	B	SE	*p*
Depression			
Criminalization → mediator	0.450	0.062	<0.001
Social support → mediator	0.008	0.059	0.890
Mediator → ART adherence	−0.217	0.087	0.013
Direct effect	−0.028	0.100	0.780
Social support → ART adherence	0.106	0.099	0.284
Indirect effect	−0.098	0.041	0.018
Total effect	−0.125	0.093	0.176

*Note*: Social support included as a covariate.

**Conclusions**: Social stressors experienced due to Ghana's anti‐LGBTQ+ legislation negatively impact the mental health of SMM, transgender women, and gender‐diverse PLHIV and indirectly lowers ART adherence through depression. Stakeholders must oppose punitive legislation and integrate mental health into HIV care for SGM living with HIV.

## OAE0306LB Artificial Intelligence‐Assisted HIV Self‐Testing for Partner Notification in China: A Multi‐Province Implementation Study

Y. Lv ^1,2^, H. Lu^3^, H. Xu^4^, Q. Zhou^5^, Y. Shi^6^, Y. Yao^7^, S. Liang^8^, H. Chen^9^, H. Tang^10^, J. Fan^11^, J. Zhang^12^, Y. Yao^13^, J. Liu^14^, G. Wang^15^, G. Zhang^16^, L. Liang^17^, J. Xie^18^, H. Yan^19^, Y. Jiang^20^, M. Han^1,2,20^, C. Jin^1,2^

^1^Chinese Center for Disease Control and Prevention, National Center for AIDS/STD Control and Prevention, Beijing, China; ^2^National Key Laboratory of Intelligent Tracking and Forecasting for Infectious Diseases, Beijing, China; ^3^Beijing Center for Disease Prevention and Control, Beijing, China; ^4^Guangdong Association for STD and AIDS Prevention and Control, Guangzhou, China; ^5^Chongqing Center for Disease Control and Prevention, Chongqing, China; ^6^Yunnan Provincial Center for Disease Control and Prevention, Kunming, China; ^7^Zhejiang Provincial Center for Disease Control and Prevention, Hangzhou, China; ^8^Guangxi Center for Disease Control and Prevention, Nanning, China; ^9^Hunan Provincial Center for Disease Control and Prevention, Changsha, China; ^10^Hubei Provincial Center for Disease Control and Prevention, Wuhan, China; ^11^Jilin Provincial Center for Disease Control and Prevention, Changchun, China; ^12^Anhui Provincial Center for Disease Control and Prevention, Hefei, China; ^13^Guizhou Provincial Center for Disease Control and Prevention, Guiyang, China; ^14^Jiangxi Provincial Center for Disease Control and Prevention, Nanchang, China; ^15^Shandong Provincial Center for Disease Control and Prevention, Jinan, China; ^16^Henan Provincial Center for Disease Control and Prevention, Zhengzhou, China; ^17^Hebei Provincial Center for Disease Control and Prevention, Shijiazhuang, China; ^18^Fujian Provincial Center for Disease Control and Prevention, Fuzhou, China; ^19^Heilongjiang Provincial Center for Disease Control and Prevention, Harbin, China; ^20^Chinese Association of STD and AIDS Prevention and Control, Beijing, China

**Background**: In China, the proportion of late HIV diagnosis (first CD4 <200 cells/µL) has remained at about 40% for years, underscoring the urgent need for targeted active testing in vulnerable groups. However, conventional HIV self‐testing (HIVST) cannot transmit results to providers, and anonymous partner‐delivered testing lacks timeliness. An AI‐integrated HIV self‐testing device (Smart HIV), featuring optical sensing, AI result reading and encrypted cloud‐based transmission, was approved in China in 2024. This study evaluated its feasibility for partner notification and its effectiveness in detecting new acquisitions and reducing late diagnosis across 17 Chinese provinces.

**Methods**: A multi‐province implementation study was conducted from May 2025 to February 2026, involving 149 voluntary counselling and testing clinics and 39 community‐based organizations. Newly diagnosed HIV index cases were recruited during routine epidemiological investigations. After counselling and consent, they distributed Smart HIV kits to their sexual partners (excluding spouses). Partners self‐tested; results along with geolocation and timestamps were uploaded automatically. Positive results triggered a 24/7 AI‐powered hotline for confirmation and referral.

**Results**: Of 6593 newly diagnosed individuals approached, 2834 (43%) agreed to serve as index cases, distributing 4986 Smart HIV kits to their sexual partners. Results were successfully returned for 4028 (80.8%) partners, of whom 488 (12.1%) tested positive for HIV. Encouragingly, among partners who tested positive, with CD4 data (*n* = 258), only 31.0% had baseline CD4 <200 cells/µL, markedly lower than the 40% late diagnosis rate in routine facility‐based testing. Furthermore, partners testing positive had a significantly higher median CD4 count compared to index cases (356 vs. 286 cells/µL, *p* < 0.001), confirming earlier acquisition stage at detection. In a pilot city, the cost per newly diagnosed partner was approximately one‐third that of standard facility‐based testing.

**Conclusions**: AI‐assisted HIV self‐testing for partner notification is highly feasible and effective, achieving a high result return rate, detecting a substantial proportion of new acquisitions at earlier disease stages, and significantly reducing late diagnosis compared to routine testing. This privacy‐preserving strategy offers a scalable, cost‐effective approach to enhance active case finding and accelerate progress towards the first UNAIDS 95 target in China (see Figure 1).

**FIGURE 1:**
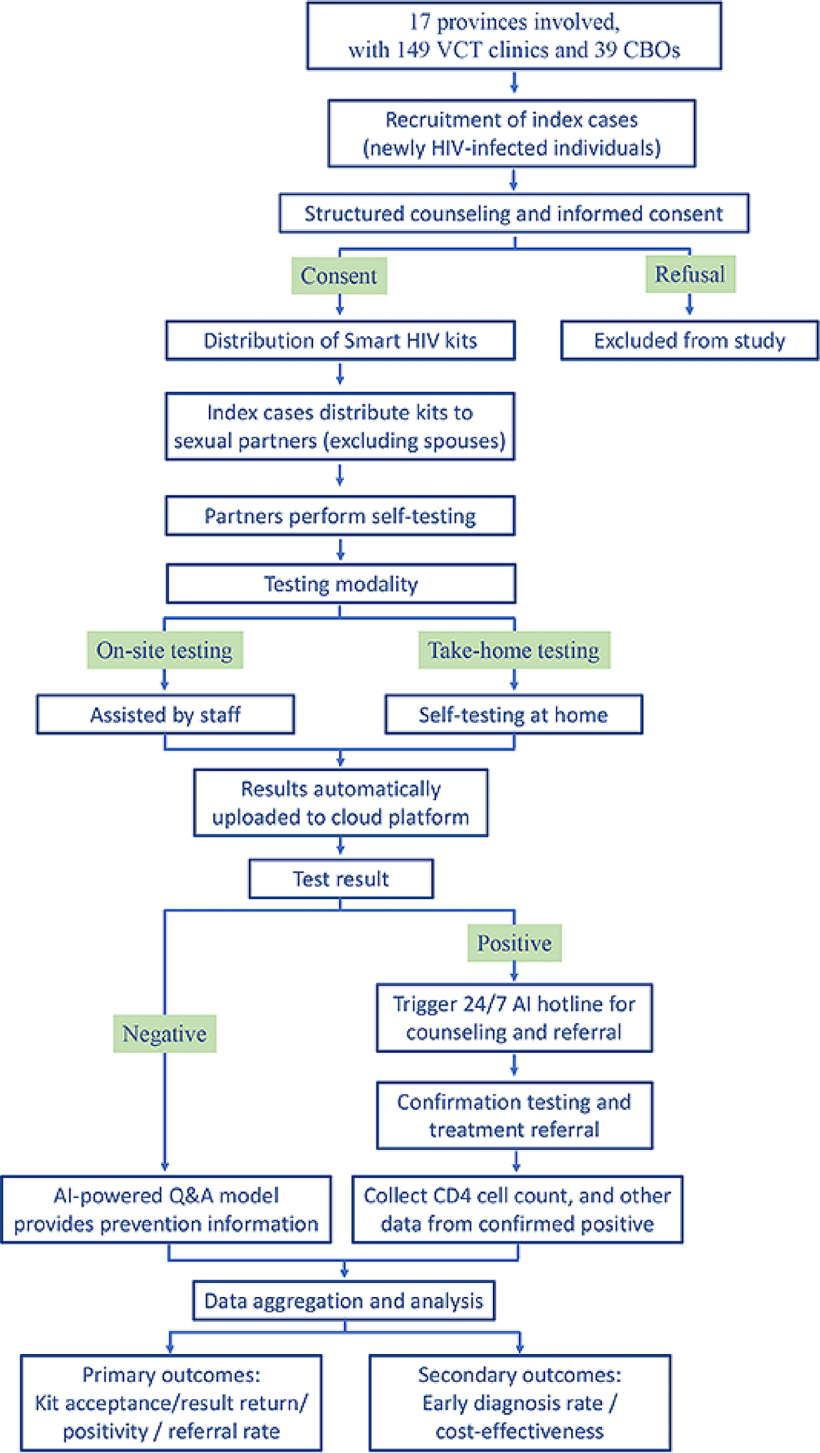
OAE0306LB | Study flow diagram of AI‐assisted HIV self‐testing for partner notification.

## OAE0506LB Beyond Donor Dependency: An Open‐Source Digital Platform to Sustain and Scale Methadone‐Assisted Therapy and HIV Integration in Kenya

L. Sitti ^1^, L. Otiso^2^, P. Masaulo^2^, P. Oyaro^2^, O. Musau^3^

^1^lvcthealth, Pharmacy, Nairobi, Kenya; ^2^Lvcthealth, Medical, Nairobi, Kenya; ^3^Lvcthealth, Prevention, Nairobi, Kenya

**Background**: Methadone‐assisted therapy (MAT) is critical for HIV prevention and treatment among people who inject drugs (PWID) in Kenya. However, scale‐up has relied heavily on donor funding and costly proprietary dispensing systems, creating sustainability risks amid global funding shifts.

**Description**: The EasyFlow‐L programme (2023–2025) was implemented across seven methadone‐assisted therapy (MAT) clinics in Coastal Kenya, targeting people who inject drugs (PWID), a key population at high risk of HIV. The facilities are transitioning from donor‐supported to county‐managed systems. EasyFlow‐L is a locally developed, open‐source platform supporting end‐to‐end MAT service delivery, including patient registration, biometric verification, clinical management, automated dispensing via a secure API and real‐time reporting, replacing costly proprietary systems.

The intervention focused on three components: improving dispensing efficiency and accuracy, reducing costs through elimination of licensing fees and integrating MAT with HIV services, including ART tracking. Key activities included user‐centred system development, staff training, deployment and ongoing technical support. Biometric authentication and automated reporting improved data quality and accountability, while rapid data migration ensured continuity of care.

**Lessons learned**: EasyFlow‐L reduced capital setup costs by 84% (USD 31,000 to USD 4800) and eliminated annual licensing fees (∼USD 14,500 per facility), enabling transition to county‐funded models. Dispensing time decreased by 90% (300 to 30 s), significantly improving clinic throughput and reducing patient waiting times. The system supported 79% retention and facilitated integration with HIV care, maintaining 161 clients on antiretroviral therapy (ART). Additional features, including biometric verification and automated reporting, enhanced accountability and data quality. Rapid data migration capabilities enabled continuity of care during service disruptions, with full patient transfers completed within 10 min.

**Conclusions/Next steps**: This intervention demonstrates how open‐source digital health solutions can mitigate the impact of donor funding reductions while strengthening HIV service delivery. By eliminating licenses enabling local ownership, EasyFlow‐L supports sustainable, scalable MAT programmes integrated with HIV care. User‐centred design contributed to high acceptability among healthcare workers and minimized technical complexity.

In conclusion, open‐source, locally developed digital platforms offer a viable pathway for sustaining HIV and harm reduction services in resource‐constrained settings. Scaling centralized, cloud‐based architectures could further enhance interoperability, patient mobility and national programme monitoring.

## OAE2606LB From Two Community Hubs to Every Thai Province: Scaling a Sex Worker‐Led HIV Self‐Testing Distribution Model Across Thailand

A. Thepbinkarn ^1^, S. Srichau^2^, S. Janyam^3^, C. Phaengnongyang^4^, S. Sumalu^4^

^1^Thammasart University, Bangkok, Thailand; ^2^Chulalongkorn University, Bangkok, Thailand; ^3^Srinakharinwirot University, Bangkok, Thailand; ^4^Service Worker In Group Foundation, Bangkok, Thailand

**Background**: HIV self‐testing (HIVST) is critical to closing the first 95 in Thailand, yet stigma, geography and clinic operating hours continue to keep sex workers, their partners and other sexually active populations away from facility‐based testing. SWING Foundation, a sex worker‐led organization, runs two community drop‐in centres in Bangkok (Silom) and Pattaya. In late 2025, SWING launched an online‐to‐offline request system that allows anyone in Thailand to order an HIVST kit through SWING's community channels and receive it by post, anonymously, anywhere in the country.

**Description**: Between 19 November 2025 and 6 May 2026, requests were submitted through SWING's online platform and community outreach networks, processed by trained peer staff at the two hubs, and dispatched by post nationwide. Each request captured age, gender, mailing address and follow‐up preferences. Recipients were invited to submit results through a confidential return channel and offered linkage to confirmatory testing and care, delivered by peer navigators familiar with each population.

**Lessons learned**: Across 168 days, two community sites processed 4412 HIVST requests (Silom 2368; Pattaya 2044), serving 4082 unique individuals. Daily volume scaled from launch to a peak of 47 requests per day in March 2026. Mailing addresses spanned all 77 of Thailand's provinces and 386 districts, with 67% of addressed requests destined for provinces outside Bangkok. The fulfilment rate reached 95.4%, with a median turnaround of 17.6 days from request to delivery. Requesters had a median age of 27.6 (74% aged under 35); 47% identified as male, 44% female, 2.1% transgender and 0.6% non‐binary. Among 122 results returned, 8 were reactive (6.6%), well above Thailand's general adult HIV prevalence, indicating that the model is reaching populations at elevated risk. All reactive cases were offered peer‐supported linkage to confirmation and care.

**Conclusions/Next steps**: A sex worker‐led, two‐site community model achieved nationwide HIVST coverage in under 6 months, demonstrating that key population organizations can extend HIV testing access far beyond their physical footprint when paired with simple digital request systems and postal logistics. Strengthening result‐return rates and formal integration with the NHSO HIVST scheme are immediate priorities for sustainable scale.

## OAE3106LB Maintaining Continuity of HIV Services in the Armed Forces of the Philippines: A Public Health and Sustainability Approach

V. Vasireddy ^1,2^, M. Develos^3^, E. R. Arellano^4^, B. E. Nillos^5^, J. S. Cavanaugh^2^

^1^Walter Reed Army Institute of Research, Armed Forces Research Institute of Medical Sciences (AFRIMS), Manila, Philippines, the; ^2^Walter Reed Army Institute of Research, Military HIV Research Program (MHRP), Bethesda, United States; ^3^Henry Jackson Foundation for the Advancement of Military Medicine (HJFMRI), Manila, Philippines, the; ^4^Armed Forces of the Philippines, Manila, Philippines, the; ^5^US Department of Defense HIV/AIDS Prevention Program, Manila, Philippines, the

**Background**: The Philippines faces Southeast Asia's fastest‐growing HIV epidemic, with a 563% increase in cases since 2017, with the population between the ages of 20−35 being the most affected. This rapid increase in new HIV transmissions poses a threat to national security through degradation of productivity and military readiness. In response, the U.S. President's Emergency Plan for AIDS Relief (PEPFAR), via the U.S. Department of War HIV/AIDS Prevention Program (DHAPP) and the Walter Reed Army Institute of Research–Armed Forces Research Institute of Medical Sciences (WRAIR‐AFRIMS), launched an HIV programme with the Armed Forces of the Philippines (AFP) in 2021.

**Description**: Early efforts established a military‐to‐military platform focused on HIV awareness, policy development, and scaling diagnostic and treatment services at key facilities to control HIV acquisitions and sustain force readiness. The programme then transitioned to a system‐wide model providing counselling, testing, antiretroviral therapy (ART), laboratory strengthening and workforce training. This abstract describes the measures undertaken by the AFP and WRAIR‐AFRIMS to transform the HIV response from a predominantly donor‐supported programme into an AFP‐led sustainable model that supports continuous HIV service delivery and sustain through uncertain PEPFAR funding (Picture 1).

**Lessons learned**: By 2024, updated HIV/AIDS Prevention and Control Guidelines institutionalized routine testing and non‐discrimination policies, allowing personnel living with HIV to remain in service if medically fit. Services now include dependents and civilian staff, shifting towards a broader public health approach. Importantly, these efforts strengthened military readiness for the AFP by enabling earlier diagnosis, reducing HIV‐related morbidity, and maintaining deployability of personnel through timely treatment and monitoring. Since inception, the AFP has trained over 1000 AFP health workers on HIV testing and treatment, performed over 20,000 diagnostic tests with 97% of clients testing positive enrolled in treatment, and 93% of those on treatment achieving viral suppression.

**Conclusions/Next steps**: The AFP HIV programme has successfully evolved from a PEPFAR‐supported initiative into a sustainable, policy‐driven, AFP‐led response. By sustaining high testing uptake and rapid linkage to ART, the programme safeguards military personnel and civilians, minimizes lost duty time and ensures operational military readiness for missions of critical importance to national security (see Figure 1).

**FIGURE 1:**
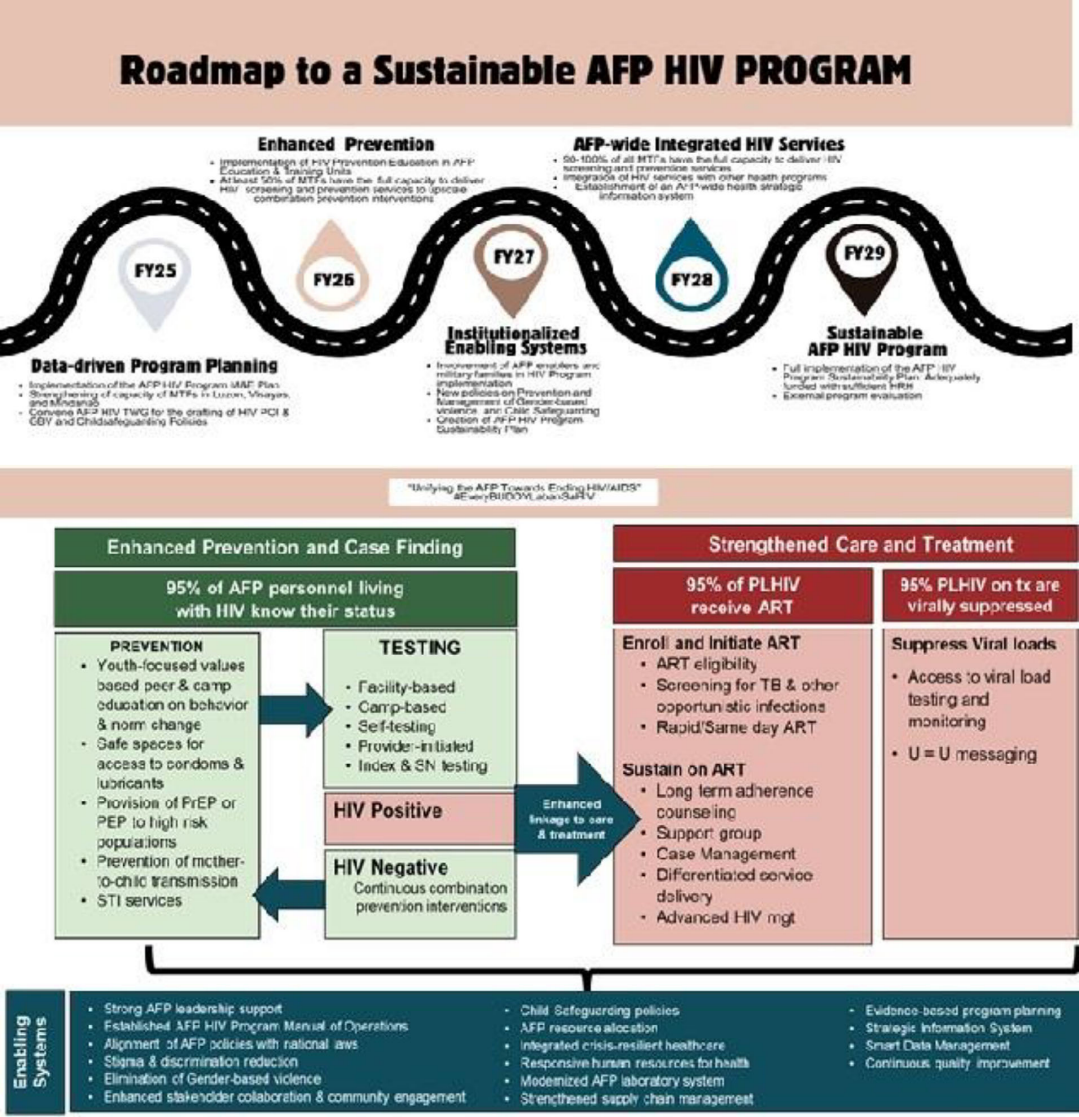
OAE3106LB

## OAE4006LB Transforming Prevention Landscapes: Community‐Led Implementation of Lenacapavir (LEN) in Nairobi

J. Kamau ^1,2,3^, L. Wells^4^, J. Mathenge^5^, A. Onyango^6^, M. Njoroge^7^, J. Muema^3^

^1^Kenya Methodist University, Health Systems Management, Nairobi, Kenya; ^2^HOYMAS KENYA, HIV Lead Clinician, Nairobi, Kenya; ^3^Centre for International Health Education and Biosecurity (CIHEB) Kenya, Clinical Services, Nairobi, Kenya; ^4^Stephen Lewis Foundation, Programs & Partnerships, Toronto, Canada; ^5^HOYMAS KENYA, Executive Directorate, Nairobi, Kenya; ^6^HOYMAS KENYA, MERL, Nairobi, Kenya; ^7^HOYMAS KENYA, HRM, Nairobi, Kenya

**Background**: The introduction of lenacapavir (LEN) in Kenya marks a pivotal shift in the national HIV response. As of January 2026, Kenya has begun rolling out this twice‐yearly subcutaneous injection, targeting high‐burden counties like Nairobi, Kisumu and Mombasa after clinical trials proved to be 100% effective. From a community perspective, LEN is widely viewed as a “freedom tool” that eliminates the daily pill fatigue and social stigma associated with oral PrEP. However, community readiness is tempered by concerns over sustainable funding and equitable access.

**Description**: HOYMAS, operating in Nairobi, piloted LEN delivery through a localized rollout tailored to populations facing adherence barriers. Key interventions included:

- Clinic adaptation: Modified one‐stop‐shop services to accommodate subcutaneous injections and 26‐week tracking.
- Treatment literacy: Peer‐led sessions demystified LEN's mode of action and addressed concerns about long‐acting dosing.
- Choice‐based counselling: Clients selected between oral PrEP, CAB‐LA and LEN based on lifestyle needs.
- Virtual engagement with the clinical staff through webinars and WhatsApp.

**Lessons learned**: Among 232 PrEP clients enrolled in the HOYMAS LEN rollout between February and April 2026,

- Uptake patterns: 89% selected LEN, 7% chose CAB‐LA and 4% remained on oral PrEP.
- Switching behaviour: Importantly, 60% of clients transitioned from oral PrEP to long‐acting injectable options (LEN or CAB‐LA), underscoring the demand for alternatives to daily dosing.
- Early uptake was driven by demand for discretion and autonomy.
- Resistance stemmed primarily from injection anxiety and concerns about drug persistence if side effects occurred.
- Peer navigators proved critical, offering real‐time peer support and demand creation.
- Those who transitioned from oral PrEP often cited “pill fatigue” and the desire for a prevention method that aligned with busy, mobile lifestyles.
- Clients value being offered multiple PrEP options. One remarked, “It isn't about being told what's best—it is about being asked what works for me.”

**Conclusions/Next steps**: Community‐led implementation of LEN demonstrates that biomedical innovation succeeds when rooted in trust, choice and peer navigation. HOYMAS’ experience provides a scalable blueprint for long‐acting PrEP delivery across sub‐Saharan Africa. Next steps include analysis of 12‐month persistence data and advocacy for policy recognition of community‐led clinics as primary sites for long‐acting PrEP.

## OAF1206LB An Innovation Withheld: Power, Control and the Delayed Rollout of Lenacapavir in HIV High‐Burden African Countries

A. W. Matendawafa ^1^, K. Richards^1^, F. Hassan^2^, T. Johnson^1^

^1^African Alliance, Johannesburg, South Africa; ^2^Health Justice Initiative (HJI), Cape Town, South Africa

**Background**: Lenacapavir, a twice‐yearly injectable PrEP, represents a transformative scientific advance in HIV prevention. Yet, previous PrEP rollouts demonstrate that efficacy rarely translates into equitable population‐level impact. Established ethical frameworks require post‐trial access planning before research begins. This paper examines whether these obligations are being met during the early rollout of lenacapavir across high HIV‐burden African countries.

**Description**: We reviewed publicly available data from 2024 to 2026 on licensing, donor procurement, approvals, shipments and rollout reports across South Africa, Eswatini, Lesotho, Mozambique, Kenya, Uganda, Zambia and Zimbabwe, countries accounting for approximately 40% of global HIV acquisitions. Qualitative insights came from civil society, programme implementers and policy observers. Access was analysed across four milestones: regulatory approval, supply, rollout, coverage, and benchmarking delivered volumes against commitments and population needs.

**Lessons Learned**: Although licensing agreements cover 120 countries, the global rollout is currently limited to 8–12 African countries, significantly fewer than the 18 countries initially prioritized by Gilead and UNAIDS. Milestones are misaligned: approval does not ensure supply, supply does not guarantee readiness or equitable coverage. Initial volumes are starkly inadequate, relative to need. By April 2026, we found that South Africa received 37,920 doses (18,960 person‐years). Kenya received 21,000 doses (approximately 10,500 person‐years), Lesotho 6000 doses and Uganda 19,200 doses, covering 9600 people against 37,000 annual acquisitions. Eswatini's 4200‐dose led to stockouts, failing to prioritize key populations. Zimbabwe's initial phase targets 46,000 people across 24 sites. Findings point to a geographically limited rollout, unclear prioritization frameworks, stockouts and constrained national control over pricing, licensing, volumes, and timelines. Respondents consistently described current implementation as “scarcity management” rather than meeting real‐time prevention needs.

**Conclusions/Next steps**: Lenacapavir's rollout exposes a central tension: a highly effective intervention delivered through a managed‐access model that limits scale, transparency and ownership. Without enforceable commitments on supply, affordability, timelines and post‐trial access, scientific breakthroughs now risk reproducing the inequities they are meant to address. Pending or future rollouts must move beyond phased rhetoric, towards binding mechanisms that ensure those most affected by HIV are the first to benefit at scale.

## OAF1706LB Assessing Transition Readiness: A Qualitative Review of 28 U.S. Global Health MOUs and Implications for HIV, TB and Malaria

J. Ratevosian ^1^, I. Castillo^2^, C. Collins^2^

^1^Duke University, Duke Global Health Institute, Durham, United States; ^2^Friends of The Global Fight, Washington, United States

**Background**: The America First Global Health Strategy represents a major shift in U.S. global health policy, transitioning financing and programme leadership for HIV, tuberculosis and malaria to countries through bilateral Memoranda of Understanding (MOUs). This transition raises critical questions about sustainability, service continuity and health governance. While the strategy emphasizes country ownership, it also introduces new risks, including financing gaps, weakened community systems, and fragmentation across bilateral and multilateral mechanisms such as the Global Fund.

**Methods**: We conducted a structured qualitative analysis based on information from 28 publicly announced MOUs. Available MOU texts and U.S. State Department press releases were reviewed across five domains:

1. co‐financing requirements and fiscal assumptions;
2. inclusion of non‐state actors, including community‐based, faith‐based and private sector partners;
3. alignment with the Global Fund and other financing mechanisms;
4. U.S. funding trends and
5. provisions for scaling innovation, including long‐acting HIV prevention and digital tools.

Findings were supplemented by policy and document review to assess implications for oversight, implementation risks and governance gaps.

**Results**: MOUs consistently outline accelerated 3‐ to 5‐year transition timelines (typically 2026–2030). Compared to FY25 U.S. funding levels, the MOU framework suggests a potential reduction in U.S. support of approximately 33%. At the same time, many countries face constrained fiscal space and challenging geopolitical conditions, raising concerns about feasibility and co‐financing capacity. While non‐state actors are widely acknowledged as essential to service delivery, few MOUs define concrete mechanisms for sustaining their financing. Across MOUs, limited specificity in implementation planning and variability in commitments suggest uneven readiness for transition.

**Conclusions**: The America First Global Health Strategy transition process reflects a rapid pivot towards country ownership but exposes a core implementation gap: ambitious targets without sufficiently defined delivery pathways. Risks include service disruption, particularly in prevention and community‐led programmes, and potential reversals in disease control. These findings highlight the need for close monitoring of early warning indicators, careful assessment of co‐financing assumptions, and stronger transparency and accountability mechanisms. Without alignment across bilateral and multilateral actors and sustained political commitment, this transition risks undermining decades of progress against HIV, TB and malaria.

## OAF2506LB Shifts in PEPFAR‐Supported Paediatric Treatment: Rapid Analysis of Fiscal Year 2025 PEPFAR Programme Data

R. Godbole ^1,2^, J. Wachinger^1^, C. Nichols^3^, M. Courey^3^, J. Bukenya^4^, F. Tanser^5,6^, R. Mpembeni^7^, K. Ngure^8^, T. Bärnighausen^1,6,9^, S. A. McMahon^1^

^1^Heidelberg Institute of Global Health, Heidelberg University Hospital, Heidelberg, Germany; ^2^Clinton Health Access Initiative, Boston, United States; ^3^Independent Researcher, Washington, United States; ^4^Makerere University College of Health Sciences, Department of Community Health and Behavioural Sciences, Kampala, Uganda; ^5^Stellenbosch University, South African Centre for Epidemiological Modelling and Analysis (SACEMA), Centre for Epidemic Response and Innovation (CERI), School for Data Science and Computational Thinking, Stellenbosch, South Africa; ^6^Africa Health Research Institute (AHRI), Somkhele and Durban, South Africa; ^7^Muhimbili University of Health and Allied Sciences, School of Public Health and Social Sciences, Department of Epidemiology and Biostatistics, Dar es Salaam, Tanzania, the United Republic of; ^8^Jomo Kenyatta University of Agriculture and Technology, Nairobi, Kenya; ^9^Harvard University, Department of Global Health and Population, Harvard T. H. Chan School of Public Health, Boston, United States

**Background**: Newly released data enables the first assessment of the status of the PEPFAR programme at the end of fiscal year 2025 (FY2025) following a year of disruption. Documented declines in the number of children living with HIV (CLHIV, <15 years old) on treatment have been characterized by the U.S. Department of State as “consistent with historical trends.” Our analysis aims to address two gaps in the existing literature focused on PEPFAR's FY2025 paediatric treatment shifts: (1) isolating analysis to “PEPFAR‐supported treatment” (site‐level service delivery and technical assistance) specifically, and (2) empirically assessing whether treatment shifts represent departures from established programme trajectories.

**Methods**: We analyse public PEPFAR programme data available at data.pepfar.gov. Country‐level analysis focuses on 21 countries reporting >2000 CLHIV on PEPFAR‐supported treatment at the end of FY2024. PEPFAR “central support” reporting, which is reported inconsistently across countries and years, is excluded to enable consistent comparisons and to exclude individuals on treatment that benefit solely from PEPFAR financing at the national or subnational level (PEPFAR). Detrended z‐scores compare each country's FY2025 change against its own FY2019–2024 historical distribution.

**Results**: Globally, 77,163 fewer CLHIV received PEPFAR‐supported treatment in FY2025 than FY2024 (–14.2%), exceeding the total reported decline when central support is included (–53,771). Among 21 focus countries, 20 reported absolute paediatric declines with proportional losses among CLHIV exceeding those among adults. South Africa recorded the largest absolute decline (–30,880; –45%), followed by Zimbabwe (–6809; –18%), Kenya (–6426; –14%) and Malawi (–4730; –15%). India (–49%) recorded the steepest proportional loss. Z‐score analysis identified departures from historical paediatric trajectories (z < –1) in five countries: Uganda (–4.00), Haiti (–2.65), Zambia (–1.47), South Africa (–1.25) and Kenya (–1.13).

**Conclusions**: Our results show a global contraction in CLHIV receiving PEPFAR‐supported treatment, with declines documented across countries and disproportionately among children compared to adults. Z‐score findings in five countries challenge broad assertions of programmatic stability. While PEPFAR programme data cannot confirm individual or population‐level treatment continuity, these signals warrant urgent country‐level investigation and restoration of public dissemination of routine age‐disaggregated PEPFAR data (see Figure 1).

**FIGURE 1:**
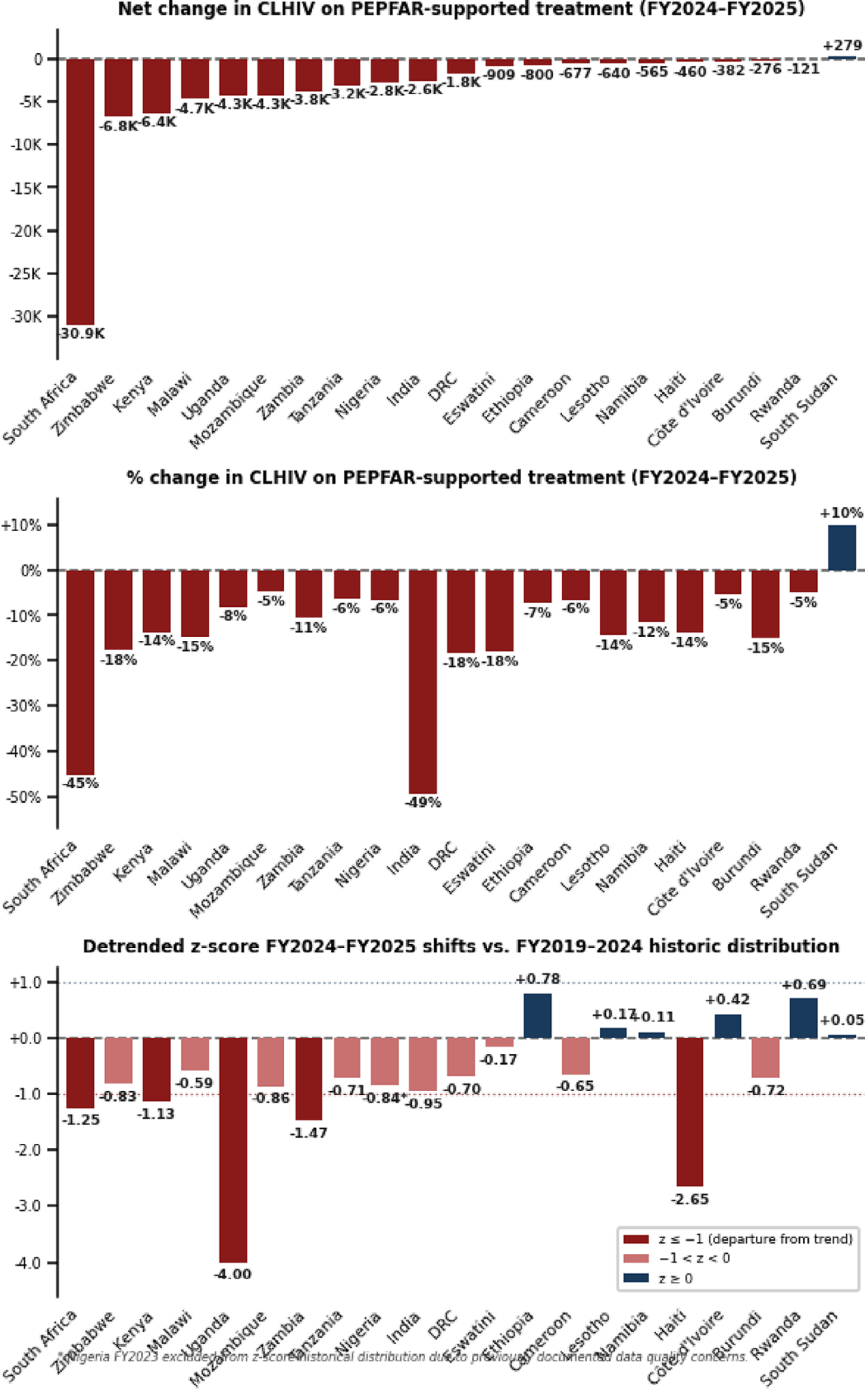
OAF2506LB

## OAF3206LB Rapid Assessment of the Impact of the Men Who Have Sex With Men Crisis on HIV Prevention and Treatment Services in Senegal

C. B. D. Gueye ^1^, A. Sagna^2^, C. T. Koulibaly^2^, S. Thiam^3^, Cheikh Bamba Dieye Gueye

^1^National Council for the Fight Against AIDS, National AIDS Control Council Monitoring, Evaluation and Research Unit, Dakar, Senegal; ^2^National AIDS Control Council, Monitoring, Evaluation and Research Unit, Dakar, Senegal; ^3^National AIDS Control Council, Executive Secretary, Dakar, Senegal

**Background**: The recent arrests of men who have sex with men (MSM), which occurred on 4 February 2026, related to alleged “unnatural acts” and intentional HIV transmission, took place within a sensitive epidemiological context. Extensive media coverage has reignited tensions surrounding sexuality, HIV and human rights. The objective of this study was to assess the impact of this crisis on HIV prevention and treatment services.

**Methods**: This was a mixed‐methods descriptive study (quantitative and qualitative) covering all 157 care sites in the country and focusing on the continuum of prevention and treatment services for people living with HIV (PLHIV). Data collection took place on 26 February and continued into March. This data was processed using Excel and analysed with SPSS 20.0 software.

**Results**: The number of people living with HIV (PLHIV) followed at 22 sites was 8803 (end of February) and 12,079 (end of March) across 42 sites. Before the arrests (end of January), 2425 patients had sought medical care, compared to 1803 patients at the end of February and 512 at the end of March. There were 15 cases of treatment discontinuation. Among the reasons for not seeking care, 52.2% were afraid of being identified as men who have sex with men (MSM), 47.8% were afraid of experiencing verbal or physical harassment and 39.1% were afraid of being arrested by the police. The percentage of MSM reporting episodes of depression or anxiety increased from 44.4% to 78.1%. The number of screenings increased from 1889 to 2121 and then to 2726. This reflects a renewed interest in HIV among the general population since this crisis. Qualitative analysis highlighted the importance of strengthening confidentiality and the protection of personal data, as well as improving communication and awareness about the disease.

**Conclusions**: The arrests that occurred during the first week of February 2026 impacted the continuum of HIV services. Recommendations include strengthening communication about HIV and highlighting the progress made by Senegal in the response to HIV. It is necessary to review the HIV law and strengthen the rights of these key populations.

## OAF3506LB Antiretroviral Delivery Performance Following Global Fund Procurement Restructuring: A 17‐Year Interrupted Time Series Analysis Across 125 Countries

S. Nurmsoo ^1,2,3^

^1^Brigham and Women's Hospital, Department of Radiology, Boston, United States; ^2^Harvard Medical School, Boston, United States; ^3^Dana‐Farber Cancer Institute, Department of Radiology, Boston, United States

**Background**: As the international community reassesses progress towards the UNAIDS 95‐95‐95 targets, supply chain performance has received limited attention as a structural barrier to public health goals. In 2019, the Global Fund restructured its Pooled Procurement Mechanism, replacing PFSCM's single‐agent role with a competitive multi‐agent model. Effects of this reorganization have not been evaluated.

**Methods**: An interrupted time series (ITS) analysis of the Global Fund Price and Quality Reporting database was performed (94,386 transactions; ARV: 41,114; TB: 22,244; 125 countries; 2008−2024), including newly released 2024 data unavailable at the original deadline, with a pre‐specified breakpoint of 2019. The primary outcome was 30‐day late delivery. Separate ITS models were fitted for ARV and TB; TB served as a negative control given negligible PFSCM exposure, though cold chain requirements differ. Logistic regression examined predictors including commodity, procurement channel, year and COVID‐19 period. Sensitivity analyses varied the threshold (14, 30, 60 days) and breakpoint (2018, 2019, 2020).

**Results**: ARV late delivery rates increased by 21.2 percentage points after the 2019 restructuring (*p* = 0.001, *R* ^2^ = 0.69), while TB rates decreased by 15.5 percentage points (*p* = 0.004, *R* ^2^ = 0.51), implicating an ARV‐specific effect predating COVID‐19. PFSCM, handling 60.7% of ARV transactions at peak in 2018 at a 24.6% late rate, was replaced by channels at 35.9% before reaching zero market share in 2020; PFSCM was independently associated with lower late delivery odds (OR 0.61, 95% CI 0.57−0.64). Ethiopia recorded 75.5% ARV late delivery versus 22.7% in South Africa, which deteriorated from 21.2% to 47.6% post‐2019. ARV rates in 2024 remain 8.2 percentage points above pre‐2019 baselines with no evidence of recovery. Results were robust across all sensitivity analyses.

**Conclusions**: The 2019 restructuring coincided with sustained ARV delivery deterioration persisting 5 years later. Divergence from TB performance provides quasi‐experimental support for an ARV‐specific mechanism. However, the concurrent WHO‐recommended transition to dolutegravir‐based therapy cannot be empirically separated as a contributing factor. These findings warrant urgent attention in the next Global Fund replenishment cycle and successor target‐setting (see Figure 1).

**FIGURE 1:**
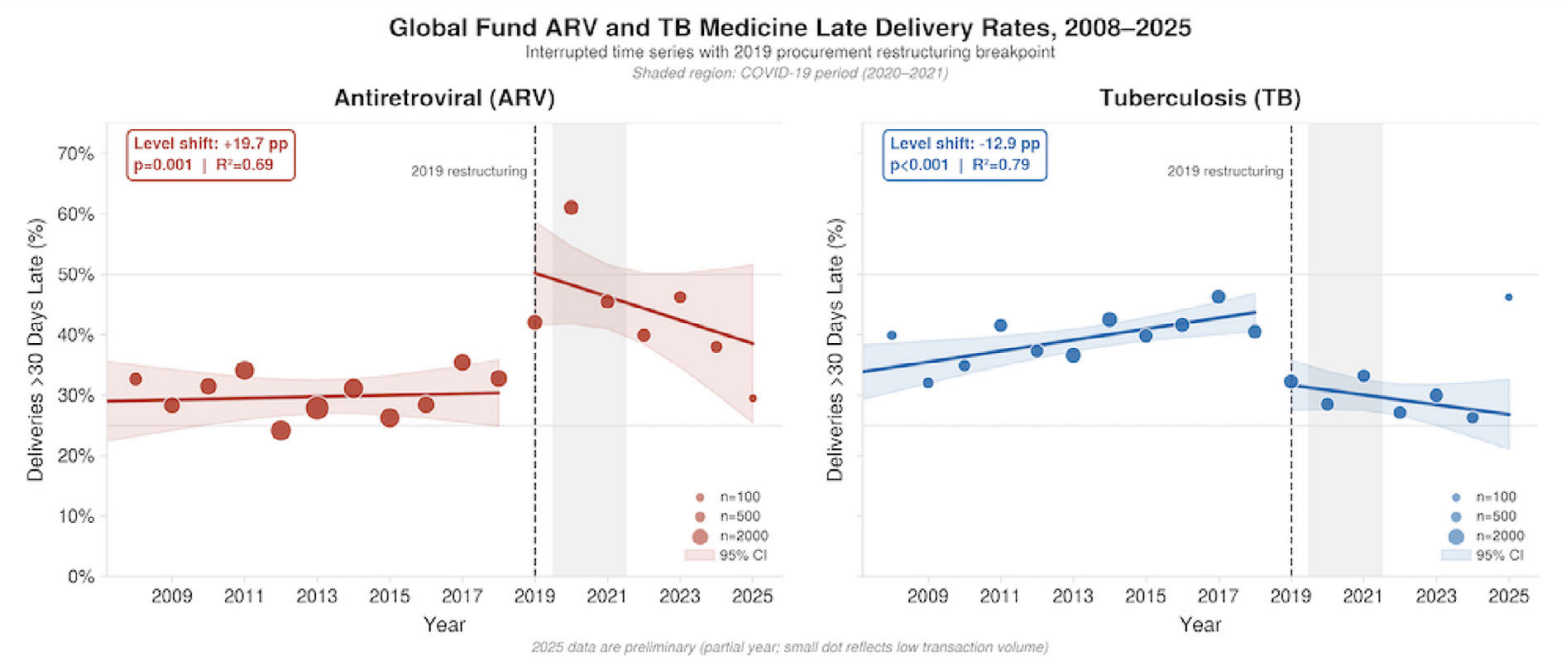
OAF3506LB

## OAX1102LB Uptake, Persistence and Preference for Long‐Acting Injectable Versus Oral PrEP Among Mobile Men in Uganda and South Africa: Primary User Effectiveness and Implementation Outcomes in the Mobile Men Trial

E. Ruzagira ^1,2^, L. Lebina^3^, N. Garret^4^, E. Webb^2^, S. Kusemererwa^5,2^, S. Nabukenya^5^, B. Nayiga Kalanzi^6^, L. Bansi‐Matharu^7^, A. Phillips^7^, J. Seeley^2,3^, M. Shahmanesh^3,7^, L.‐G. Bekker^4^, J. Fox^8^, the Mobile Men Study Group

^1^MRC/UVRI and LSHTM Uganda Research Unit, Entebbe, Uganda; ^2^London School of Hygiene and Tropical Medicine, London, United Kingdom; ^3^Africa Health Research Institute, Durban, South Africa; ^4^Desmond Tutu HIV Foundation, Cape Town, South Africa; ^5^MRC/UVRI and LSHTM Uganda Research Unit, Entebbe, Uganda; ^6^MRC/UVRU and LSHTM Uganda Research Unit, Entebbe, Uganda; ^7^University College London, London, United Kingdom; ^8^King's College London, London, United Kingdom

**Background**: Men who travel for work face elevated HIV vulnerability and limited access to prevention services. Adherence to daily oral pre‐exposure prophylaxis (PrEP) is often challenging in this population, whereas long‐acting injectable PrEP may improve uptake and persistence.

**Methods**: Mobile Men is a phase 3b, open‐label, trial comparing cabotegravir (CAB‐LA) with oral tenofovir/emtricitabine (TDF/FTC; daily/event‐driven) among men who travel for work. The study comprises a 9‐month randomized phase followed by a 9‐month choice phase. From August to November 2024, participants were enrolled via mobile clinics at taxi ranks in East London and KwaZulu‐Natal, South Africa, and through referrals from fishing communities to a clinic in Masaka, Uganda, with same‐day PrEP initiation after HIV testing. Outcomes were PrEP uptake; 9‐month persistence (receipt of all scheduled CAB‐LA injections/quarterly oral PrEP visits with continuous drug coverage, within a ± 7‐day window); and participants’ preference and actual product choice at month 9.

**Results**: Of 428 men screened, 400 were randomized to CAB‐LA (*N* = 202) or oral PrEP (*N* = 198), with a median age of 32 years (IQR 25−39); one CAB‐LA participant did not initiate treatment. Overall persistence was low (22%) but higher with CAB‐LA than oral PrEP (28% vs. 16%; OR 2.54, 95% CI 1.43, 5.43; *p* = 0.002). At month 9, 304/400 (76%) attended the study visit; injectable PrEP was most preferred (169/304, 56%), followed by event‐driven (81/304, 27%) and daily oral PrEP (54/304, 18%). Preferences varied by prior exposure: 74% in the CAB‐LA arm preferred injectable PrEP, while preferences in the oral PrEP arm were more evenly distributed (37% injectable, 38% event‐driven, 25% daily). Overall, 58% chose CAB‐LA versus 43% oral PrEP (*p* = 0.01). 110/134 (76%) randomized to CAB‐LA chose CAB‐LA, while 54/131 (38%) on oral PrEP chose CAB‐LA (*p* < 0.001).

**Conclusions**: Same‐day PrEP delivery is feasible for this population. Although persistence was low, it was higher with injectable PrEP, which was also the preferred modality, particularly among prior users. Long‐acting PrEP is a promising HIV prevention option for mobile populations; ongoing qualitative analyses will inform strategies to improve persistence, and cost‐effectiveness will be assessed.

## OAX1103LB Measuring PEPFAR Disruptions at Scale: Results From a 46‐Country Implementing Partner Survey

J. Sherwood^1^, E. Lankiewicz ^1^, C. Gironda^2^, A. Sharp^3^, K. Alicea‐Jorgensen^4^, A. Bonifaz^2^, K. Rucinski^5^, M. Davis^2^, B. Honermann^1^, G. Millett^1^, S. Baral^4^

^1^amfAR The Foundation for AIDS Research, Public Policy Office, Washington DC, United States; ^2^Funders Concerned About AIDS, Washington DC, United States; ^3^Data Et Cetera, Washington DC, United States; ^4^Johns Hopkins School of Public Health, Epidemiology, Baltimore, United States; ^5^Johns Hopkins School of Public Health, International Health, Baltimore, United States

**Background**: Substantial changes to U.S. foreign aid in early 2025 altered the PEPFAR program. While initial reports showed acute HIV service declines, no PEPFAR‐wide assessment of the effects on implementing partners (IPs) exists. The PEPFAR Pulse study provides the first large‐scale survey of PEPFAR IPs to document the scale and scope of PEPFAR disruptions globally.

**Methods**: From November 2025 to April 2026, an electronic survey was distributed to all country‐level IPs intended to receive PEPFAR funding in FY2025, reaching 166 IPs across 46 countries (23%,166/708). Contact information was sourced from professional networks and public sources, with each IP contacted up to three times by email. Data were analyzed using descriptive and inferential statistics (R 4.3.3).

**Results**: Overall, 3% (5/158) of organizations permanently closed, 52% (82/158) had at least one terminated award, and 81% (122/150) were asked to restrict their work to comply with an additional U.S. policy. Terminations led to 1,714 closed service sites, including 1,010 public health facilities, 325 access points, and 126 drop‐in centers. Locally headquartered organizations were significantly more impacted by terminations (Table 1). By program area, IPs most commonly had permanently stopped providing key population (KP) services (73%, 45/62), community‐based services (46%, 35/76), socioeconomic support services (49%, 20/41), or prevention services (43%, 40/93). Among IPs providing HIV treatment, 21% (18/85) permanently stopped at least one HIV clinical care activity. Data systems and supply chains were also disrupted, with IPs reporting inability to obtain condoms (23%, 25/109), lab commodities (23%, 25/109), PrEP (22%, 22/109), and ARVs (20%, 22/109). Even among IPs with no terminated awards, the majority censored language (61%, 11/18), stopped a service (61%, 11/18), or stopped services to specific populations (44%, 8/18) to continue receiving funding.

**Conclusions**: U.S. funding and policy changes caused PEPFAR‐wide disruptions, with disproportionate impacts on local organizations and KPs. The loss of these implementers threatens program sustainability and risks reversing HIV epidemic control given the biggest service losses were among people most vulnerable to HIV acquisition and transmission. Ultimately, moving forward will require sustainable solutions to maintain HIV services including supporting locally based implementers and KP‐led HIV programming to reach populations who lost PEPFAR support.

**TABLE 1:** OAX1103LB | Organizational disruptions by implementing partner type.

Disruption type	Overall^a^ % (*n*)	Local organizations^b^ % (*n*)	International organizations % (*n*)	*p*‐value^c^
≥1 award terminated	56.6 (82/153)	62.9 (56/89)	40.6 (26/64)	0.008^*^
≥1 award delayed/may not be paid	55.1 (81/147)	47.7 (41/86)	65.6 (40/60)	0.043^*^
Any site closures	15.8 (25/158)	23.3 (21/90)	5.9 (4/68)	0.004^*^
Asked to comply with additional U.S. policies	81.3 (122/150)	77.9 (67/86)	85.9 (55/64)	0.290

^a^ Overall denominators for groups vary based on individual variable non‐response and available data on IP type (ie. local vs. international).

^b^ Local partners defined as those headquartered in the same country as the award implementation

^c^ *p*‐values calculated using Fisher's exact test.

* *p* < 0.05.

## OAX1104LB Safety and Preliminary Efficacy of 6‐Monthly Subcutaneous CAP256V2LS Plus VRC07‐523LS for HIV Prevention in African Women: Results of the CAPRISA 012C Phase 2 Randomized Controlled Trial

S. Mahomed ^1,2^, F. Osman^1^, I. Harkoo^1^, D. Potloane^1^, I. T. Sikazwe^3^, E. Kamuti^3^, N. N. Mkhize^4,5^, K. Carlton^6^, N. Doria‐Rose^6^, L. Lewis^1^, D. Archary^1,2^, T. N. Gengiah^1^, N. Samsunder^1^, L. E. Mansoor^1^, N. Devnarain^1^, J. Mascola^6^, R. A. Koup^6^, M. Castro^6^, S. Narpala^6^, L. Serebryannyy^6^, B. Pozzetto^7^, S. Paul^7^, C. Hankins^8^, M.‐R. Abrahams^1,9^, L. Morris^1,10^, C. Williamson^1,9^, P. Moore^1,4,5^, Q. Abdool Karim*^1,11^, S. S. Abdool Karim*^1,11^

^1^Centre for the AIDS Programme of Research in South Africa (CAPRISA), University of KwaZulu‐Natal, Durban, South Africa; ^2^Department of Medical Microbiology, School of Laboratory Medicine and Medical Science, University of KwaZulu‐Natal, Durban, South Africa; ^3^Centre for Infectious Disease Research (CIDRZ), Lusaka, Zambia; ^4^South African Medical Research Council Antibody Immunity Research Unit, Faculty of Health Sciences, University of the Witwatersrand, Johannesburg, South Africa; ^5^National Institute for Communicable Diseases, National Health Laboratory Service, Johannesburg, South Africa; ^6^Vaccine Research Center, National Institute of Allergy and Infectious Diseases, National Institutes of Health, Bethesda, Maryland, United States; ^7^Centre International de Recherche en Infectiologie, Université Claude Bernard Lyon 1, Inserm, CNRS, ENS Lyon, Université Jean Monnet de Saint‐Etienne, France, Saint‐Etienne, France; ^8^Amsterdam Institute for Global Health and Development, Academic Medical Centre, University of Amsterdam, Amsterdam, the Netherlands; ^9^Division of Medical Virology, Institute of Infectious Disease and Molecular Medicine, University of Cape Town and National Health Laboratory Services, Cape Town, South Africa; ^10^Faculty of Health Sciences, University of the Witwatersrand, Johannesburg, South Africa; ^11^Department of Epidemiology, Mailman School of Public Health, Columbia University, New York, United States

**Background**: CAPRISA 012C is the first trial of a combination of two broadly neutralizing antibodies (bnAbs). It assessed the safety and efficacy of fixed‐dose subcutaneous CAP256V2LS plus VRC07‐523LS (selected to achieve both neutralization breadth and potency) for HIV prevention in young women in South Africa and Zambia.

**Methods**: A randomized, double‐blind, placebo‐controlled phase 2 trial was conducted with 1023 women aged 18–30 years who were assigned 1:1 to CAP256V2LS plus VRC07‐523LS (*n* = 512) or placebo (*n* = 511). BnAb recipients received 1.2 g of each bnAb at baseline, followed by 600 mg CAP256V2LS plus 1200 mg VRC07‐523LS every 24 weeks. Plasma and genital concentrations of both bnAbs were measured 3‐monthly. The primary endpoint was safety. HIV incidence in bnAb and placebo recipients was compared overall and in subgroups based on the neutralization sensitivity of 36 of the 39 breakthrough viruses to CAP256V2LS and VRC07‐523LS using the TZM‐bl assay at thresholds of 0.1 and 1 µg/mL, respectively.

**Results**: Baseline demographic characteristics and oral PrEP uptake were similar in placebo and bnAb recipients. Serious adverse events occurred in 7 bnAb and 11 placebo recipients, and the remaining side effect frequencies were similar in both groups except for local infusion site reactions, which were mostly mild, occurring more commonly in bnAb recipients. HIV incidence rates were 2.61 (17/650 women‐years [wy]) and 3.38 (22/651wy) per 100wy in the placebo and bnAb recipients, respectively (incidence rate ratio [IRR] 1.29, 95% confidence interval [CI] 0.69–2.43). In placebo versus bnAb recipients, 67% (10/15) versus 95% (20/21) of the viruses were resistant to CAP256V2LS, and 60% (9/15) and 81% (17/21) were resistant to VRC07‐523LS. For viruses sensitive to CAP256V2LS and VRC07‐523LS, the HIV IRRs (CIs) were 0.20 (0.02−1.71) and 0.67 (0.19–2.36), respectively. HIV incidence in relation to bnAb concentrations and sensitivity provides additional insights for these HIV outcomes.

**Conclusions**: Fixed‐dose subcutaneous CAP256V2LS plus VRC07‐523LS was safe but not effective in reducing overall HIV incidence. Higher than expected bnAb resistance may explain the lack of overall efficacy, as there was a trend towards protection for viruses sensitive to one or both bnAbs. Matching bnAbs with circulating viruses will be important for future bnAb studies.

## OAX1105LB Every‐8‐Weeks Injectable Cabotegravir and Rilpivirine is Superior to Daily Oral Tenofovir Disoproxil Fumarate/Lamivudine/Dolutegravir in Adolescents Living With HIV in Sub‐Saharan Africa: LATA 96‐Week Results

M. Bwakura Dangarembizi ^1^, E. Chappell^2^, A. R. Kekitiinwa^3^, C. Kityo^4^, A. Siika^5^, M. Archary^6^, L. Jafta^7^, H. A. Mujuru^1^, L. D. Anena^3^, S. Nakabuye^4^, C. Kiilu^5^, R. Mngqibisa^6^, G. Akabwai^3^, H. Mugerwa^4^, S. Walker^8^, J. Seeley^9,10^, D. Burger^11^, A. Omar^2^, A. Bamford^2^, A. South^2^, H. Nguyen^2^, C. Giaquinto^7^, B. Spittle^2^, D. Ford^2^, S. Pett^2^, LATA trial team

^1^University of Zimbabwe Clinical Research Centre, Harare, Zimbabwe; ^2^UCL Innovative CTU (formerly the Medical Research Council Clinical Trials Unit at University College London (UCL)), London, United Kingdom; ^3^Baylor College of Medicine Children's Foundation, Kampala, Uganda; ^4^Joint Clinical Research Centre, Kampala, Uganda; ^5^Moi University Clinical Research Centre, Eldoret, Kenya; ^6^Department of Paediatrics and Children Health, Victoria Mxenge Hospital, Enhancing Care Foundation, University of KwaZulu‐Natal, Durban, South Africa; ^7^Fondazione Penta ETS, Padova, Italy; ^8^Centre for Health Economics, University of York, York, United Kingdom; ^9^Medical Research Council/ Uganda Virus Research Institute and London School of Hygiene and Tropical Medicine Uganda Research Unit, Entebbe, Uganda; ^10^Africa Health Research Institute, Nelson R. Mandela School of Medicine, 3rd Floor, K‐RITH Tower Building, Durban, South Africa; ^11^Department of Pharmacy, Radboud University Medical Center Nijmegen, Nijmegen, the Netherlands

**Background**: Long‐acting injectable (LAI) cabotegravir/rilpivirine (CAB/RPV) antiretroviral therapy (ART) has demonstrated high efficacy, safety and acceptability in adults with human immunodeficiency virus (HIV). Adolescents with HIV who may face additional adherence challenges could also benefit from LAIs.

**Methods**: LATA was a non‐inferiority trial in Kenya, South Africa, Uganda and Zimbabwe. Adolescents (12−19 years), with viral load (VL) <50 copies/mL for ≥12 months on ART and no prior treatment failure, were randomized to every‐8‐weeks LAI CAB/RPV or daily oral control (tenofovir/lamivudine/dolutegravir [TLD]). Primary outcome was intent‐to‐treat adjusted Kaplan−Meier proportion with confirmed VL ≥ 50 copies/mL (viral rebound) by week 96. Non‐inferiority margin and significance level depended on control event rate (8.9% margin, 99%CI for 6% event rate). VLs were 24‐weekly. We present 96‐week data.

**Results**: 476 (235 LAI, 241 control) participants were enrolled (256 [54%] female, median age 16.5 years [IQR: 14.8−18.1], prior ART 11.7 years [IQR: 8.5−14.1], 98% vertically acquired HIV). At week 96, 98% remained in follow‐up. Two (0.9%) LAI versus 15 (6.4%) control participants experienced viral rebound, difference LAI‐control –5.5% (99% CI: –10.3, –1.5); non‐inferiority of LAIs was demonstrated using an 8.9% margin (Figure 1). LAI was superior to control (*p* = 0.001). One of two LAI‐arm participants with viral rebound switched to oral ART for an adverse event (AE) before rebound. There were no differences in proportions with confirmed VL ≥200 or ≥1000 copies/mL (Table 1). There were five permanent discontinuations of LAI (one confirmed VL≥200 copies/mL, two AE‐related to LAI (serious hypersensitivity reaction [HSR]; non‐serious drug eruption), two planning pregnancy). No control participants discontinued TLD. Eight LAI and 10 control participants experienced ≥1 serious AE (*p* = 0.636). HSR was the only LAI‐related SAE. 213 (91%) in LAI arm reported ≥1 injection site reaction (ISR), 3 (1%) had ≥1 grade ≥3 ISR. 94% of LAI participants reported that taking LAIs was a lot easier than taking oral ART.

**FIGURE 1:**
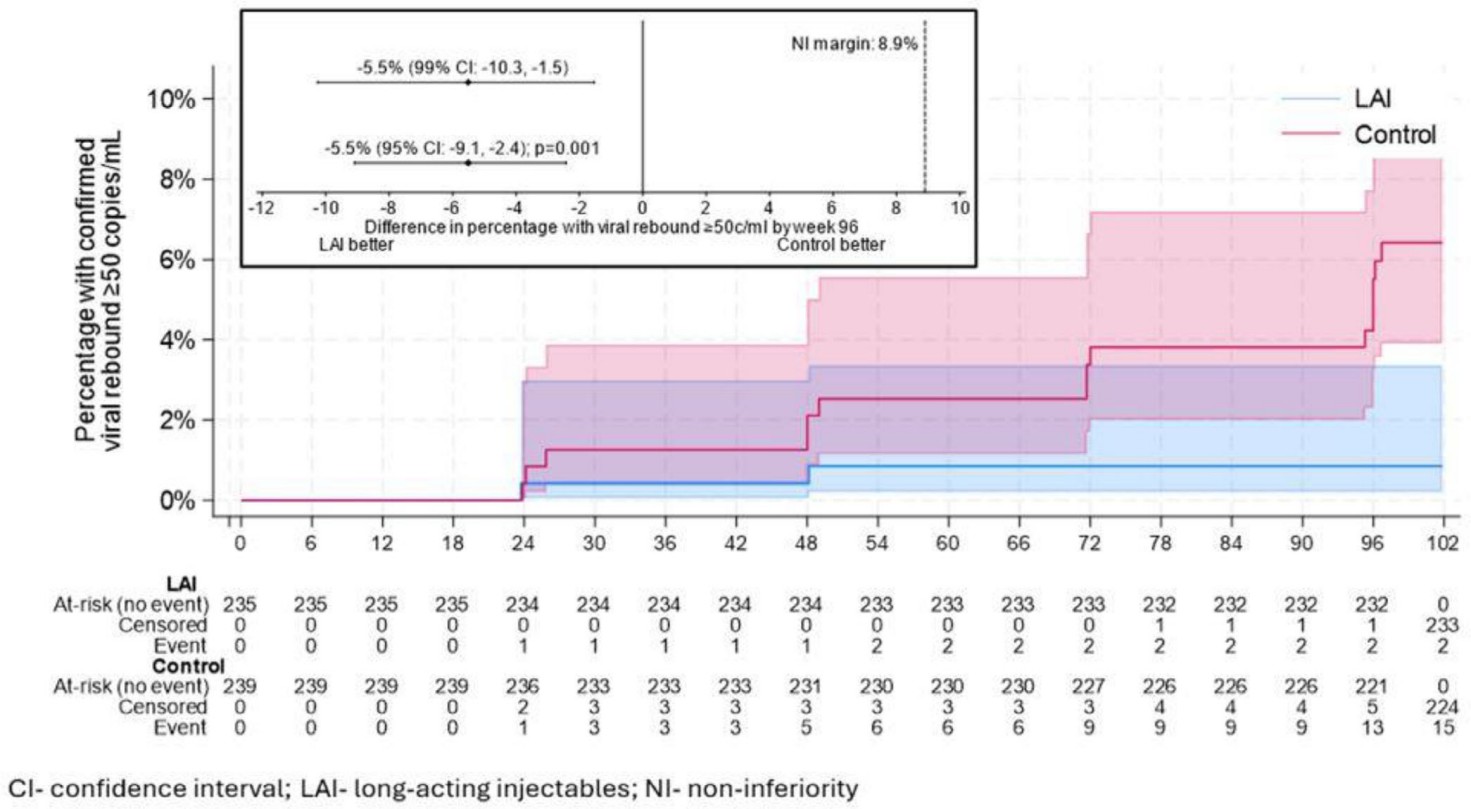
OAX1105LB | LATA primary outcome—Proportion with confirmed viral rebound (≥50 copies/mL) by week 96, by trial arm.

**TABLE 1:**
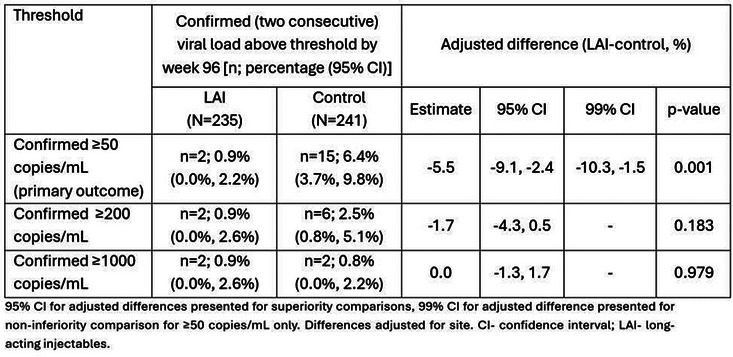
OAX1105LB | Confirmed (two consecutive) viral load above threshold by week 96, by trial arm.

**Conclusions**: LAI CAB/RPV was superior to TLD for maintenance of virological suppression in adolescents with HIV, showed no new safety concerns and was preferred.

## OAX1106LB Once‐Weekly Oral Islatravir/Lenacapavir (ISL/LEN) Versus Daily Standard‐of‐Care Therapy in Virologically Suppressed Adults With HIV‐1: Week 48 Results of the ISLEND‐2 Phase 3 Trial

A. E. Colson ^1^, P. N. Kumar^2^, P. J. Ruane^3^, P. Chetchotisakd^4^, W. Ratanasuwan^5^, A. Liberty^6^, R. G. Nahass^7^, A. Arenas‐Pinto^8^, F. A. Post^9^, D. A. Baker^10^, P. Cahn^11^, E. Hofmann^12^, D. Watanabe^13^, P.‐L. Lu^14^, A. H. E. Roukens^15^, M. Parczewski^16^, H. Wang^17^, K. Chen^18^, F. Shihadeh^17^, M. Shaughnessy^18^, H. Dvory‐Sobol^17^, D. SenGupta^17^, C. Llamoso^18^, M. S. Rhee^17^, O. Ogbuagu^19^, for the ISLEND‐2 Study Team

^1^Community Resource Initiative, Boston, United States; ^2^Georgetown University Hospital, Washington, D. C., United States; ^3^Ruane Clinical Research Group, Inc., Los Angeles, United States; ^4^Srinagarind Hospital, Khon Kaen University, Khon Kaen, Thailand; ^5^Siriraj Hospital, Mahidol University, Faculty of Medicine, Bangkok, Thailand; ^6^Perinatal HIV Research Unit‐Soweto, University of the Witwatersrand, Johannesburg, South Africa; ^7^IDCare, Hillsborough, United States; ^8^Institute for Global Health, University College London, London, United Kingdom; ^9^King's College Hospital NHS Foundation Trust, London, United Kingdom; ^10^East Sydney Doctors, Darlinghurst, Australia; ^11^Fundación Huésped, Buenos Aires, Argentina; ^12^Inselspital, Bern University Hospital, University of Bern, Department of Infectious Diseases, Bern, Switzerland; ^13^AIDS Medical Center, NHO Osaka National Hospital, Osaka, Japan; ^14^Kaohsiung Medical University Hospital, Kaohsiung, Taiwan, Province of China; ^15^Leiden University Center for Infectious Diseases, Leiden University Medical Center, Leiden, the Netherlands; ^16^Pomeranian Medical University, Department of Infectious, Tropical Diseases and Acquired Immune Deficiency, Szczecin, Poland; ^17^Gilead Sciences, Inc., Foster City, United States; ^18^Merck & Co., Inc., Rahway, United States; ^19^Yale School of Medicine, Yale University, New Haven, United States

**Background**: Need exists for oral HIV‐1 treatment options with longer dosing intervals to improve adherence and outcomes in people with HIV‐1 (PWH).

**Methods**: ISLEND‐2 (NCT06630299) is a global, multicentre, randomized, open‐label, Phase 3 noninferiority trial. Virologically suppressed adult PWH on daily oral standard‐of‐care (SOC) antiretroviral therapy, with no prior virologic failure, were randomized 1:1 to switch to once‐weekly oral ISL/LEN (2/300 mg) or continue daily SOC, for 96 weeks. The primary efficacy endpoint was the proportion of participants with HIV‐1 RNA ≥50 copies/mL at Week (W) 48 (US FDA‐defined snapshot algorithm; 4% noninferiority margin). Proportion of participants with HIV‐1 RNA <50 copies/mL, change from baseline in CD4+ T‐cell count and ISL/LEN discontinuations due to adverse events (AEs) were key secondary endpoints.

**Results**: 626 participants were treated (ISL/LEN: *N* = 314; SOC: *N* = 312); median age was 52 years (58% ≥50 years; 14% ≥65 years), 34% were female, 31% Black and 20% Hispanic or Latine. At W48, one (0.3%) ISL/LEN and four (1.3%) SOC participants had HIV‐1 RNA ≥50 copies/mL (Figure 1; difference: –1.0%; 95.002% confidence interval [CI]: –3.0, 1.1), demonstrating noninferiority. 299 (95.2%) ISL/LEN and 298 (95.5%) SOC participants had HIV‐1 RNA <50 copies/mL (difference: –0.3%, 95% CI: –3.9, 3.2%). Mean change in CD4+ T‐cell count at W48 was –45 and –8 cells/µL with ISL/LEN and SOC, respectively (least‐squares mean difference: –31; 95% CI: –58, –4), potentially reflecting higher mean baseline counts in the ISL/LEN group (ISL/LEN: 795 cells/µL vs. SOC: 761 cells/µL), with convergence at W48 (750 cells/µL in both groups; Figure 1). Mean percentage change in CD4+ T‐cell counts at W48 were similar, with overlapping CIs (ISL/LEN: –0.2%, 95% CI: –4.2, 3.9; SOC: 2.4%, 95% CI: –0.8, 5.6). There were no between‐group differences in lymphocyte counts at W48, and no participant discontinued due to reduced CD4+ T‐cell or lymphocyte counts. ISL/LEN was generally well tolerated (Table 1). One (0.3%) ISL/LEN participant discontinued due to an AE of hepatitis B (incident infection in an unvaccinated individual).

**FIGURE 1:**
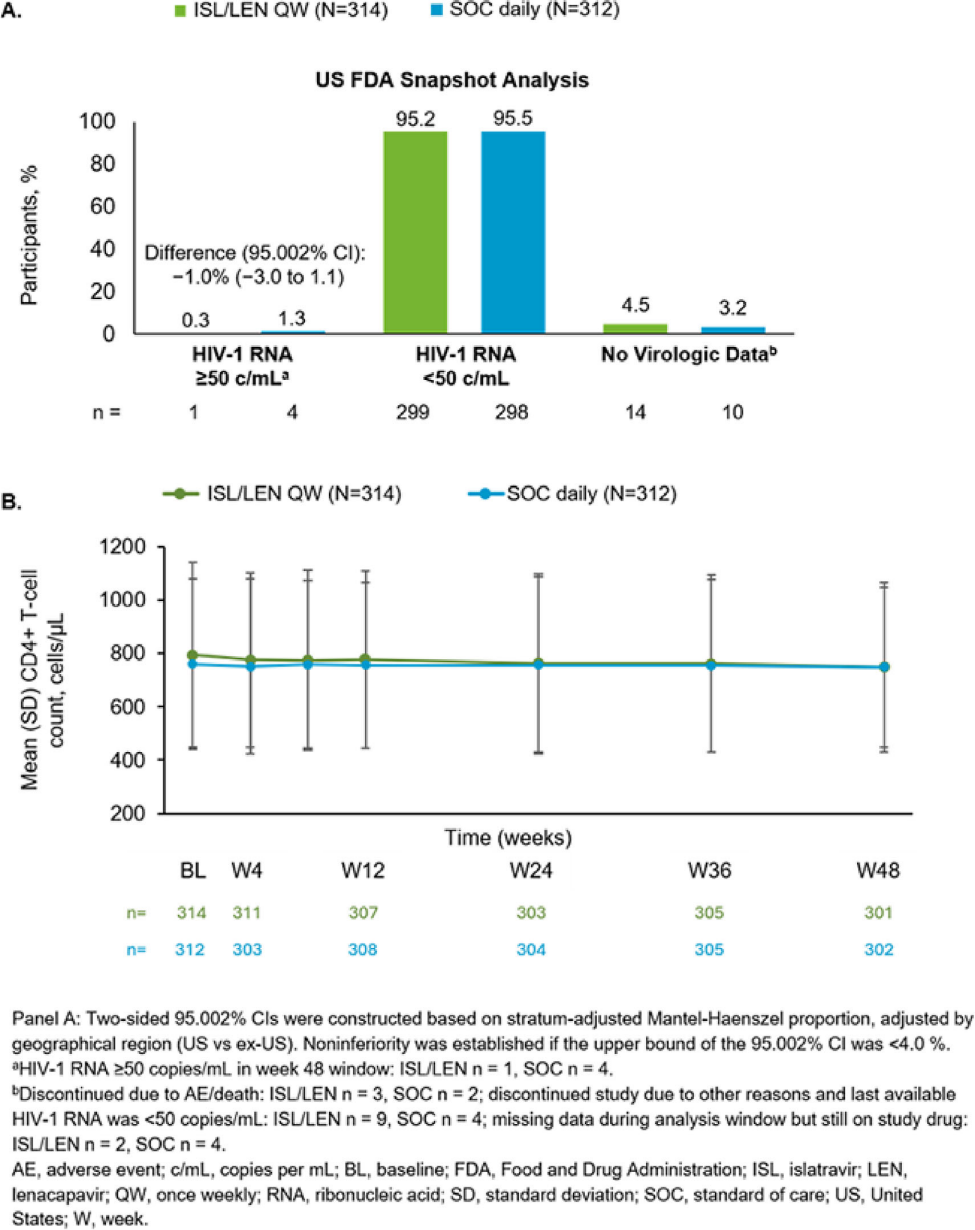
OAX1106LB | Virologic outcomes at week 48 (A) mean (SD) CD4^+^ T‐cell count through week 48 (B).

**TABLE 1:**
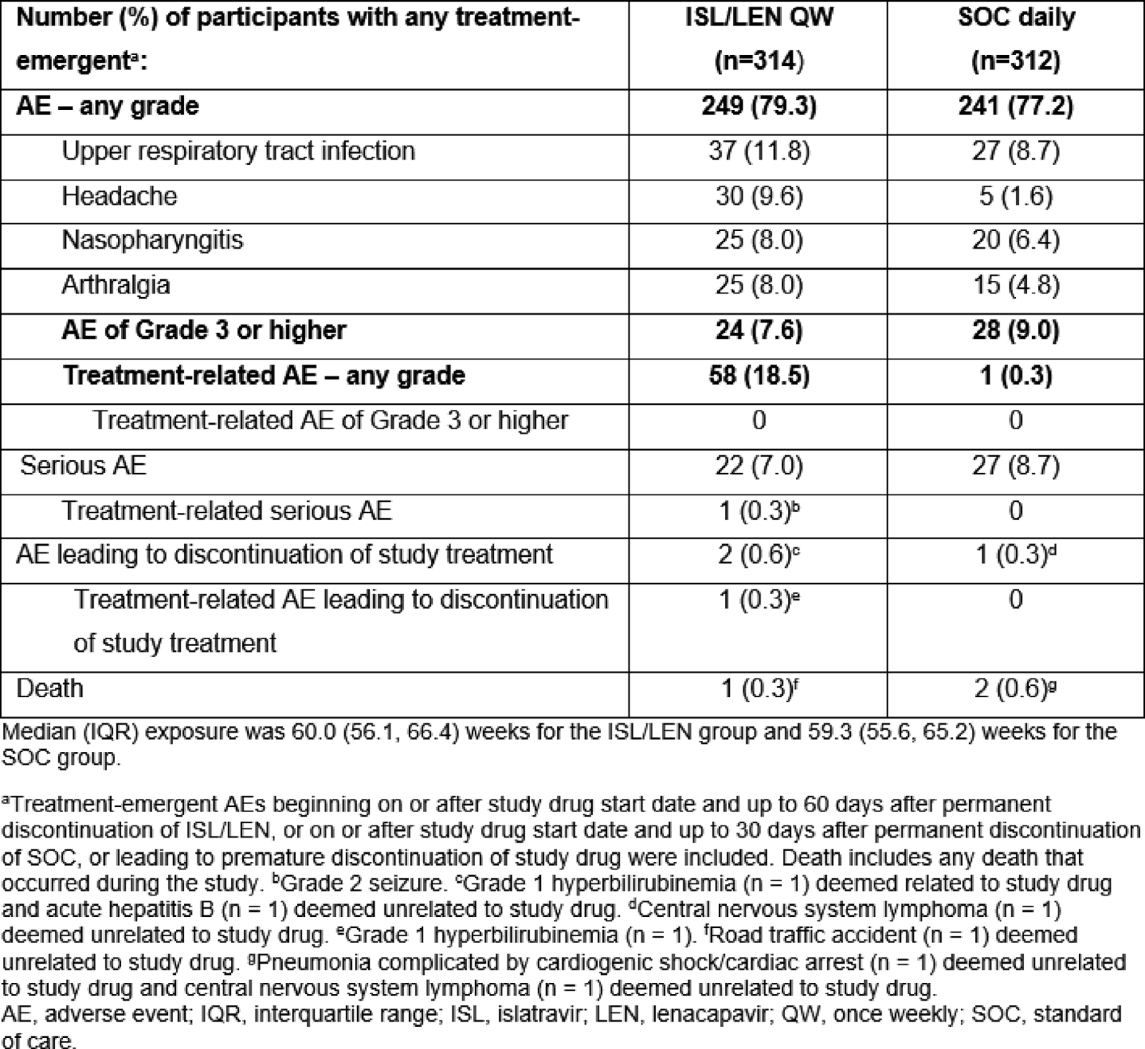
OAX1106LB | Safety summary.

**Conclusions**: ISL/LEN was efficacious and well tolerated and has the potential to be the first complete once‐weekly oral single‐tablet treatment regimen for HIV‐1.

**Funding**: Gilead Sciences, Inc. and Merck Sharp & Dohme LLC.

## Author Index

### A

A Price, M. OAA2702

Abbasi, M. OAB0903

Abbate, M. C. OAE3105

Abdel‐Mohsen, M. OAA1002

Abdool Karim, Q. OAX1104LB

Abdool Karim, S. S. OAX1104LB

Abdullahi, R. OAE0302

Abhyankar, S. OAA1506LB

Abimuku, A. OAC1304

Abrahams, M.‐R. OAX1104LB

Abubakar, M. M. OAD3603

Achiangia Njukeng, P. OAD3303

Achieng Ombajo, L. OAB1402

Acubo, N. OAE2605

Adam, A. OAD2105, OAF2502

Adams, J. OAB4106LB

Adebayo, O. OAC1304

Adekogbe, A. OAD3302

Adekunle, A. OAC1304

Adetoye, S. OAE0302

Adeyemi, O. OAD2404

Afolabi, B. OAD3302

Afre, A. OAB4105

Agbaje, A. OAC1304

Agbo, F. OAC0205

Agi, B. OAD3306LB

Agostino, C. OAA3002

Agyemang, E. OAB2003

Ahimbisibwe, G. OAB2306LB

Ahmar, M. OAF1203

Aikaeli, F. OAE3702

Airoje, O. OAE0302

Aiyana, O. OAA1005

Ajonye, A. OAD2404

Ajuka‐Patrick, V. OAC1304

Akabwai, G. OAX1105LB

Akatukunda, B. OAB1402

Ake, J. OAA3003, OAB2304

Akhtar, A. OAC2802

Akinlalu, A. OAD3302

Akinniranye, H. OAD3302

Akpomiemie, G. OAB3406LB

Alam, N. OAB4106LB

Albertini, M. OAB0904

Alejos, B. OAB3405

Ali, A. OAC0705

Ali, E. OAF2502

Alicea‐Jorgensen, K. OAX1103LB

Aliu, I. OAD3306LB

Aliyu Magaji, R. OAC0205

Allavena, C. OAA1503, OAB0102

Allinder, S. M. OAE2903

Allombert, M. OAA1503

Almeida, F. OAD1802

Alozie, J. OAD3302

Alrubayyi, A. OAA2702

Altamirano, J. OAC2805

Altice, F. OAD3605

Alves Silva, P. V. OAE0305

Amadiegwu, S. OAD2404

Amado, C. OAE2605

Amatsombat, T. OAD1803

Amole, C. OAF1202

An, H. OAA3004

Anderson, J. OAB3404

Andia Salazar, O. OAF1204

Andorf, S. OAB3403

Anena, L. D. OAX1105LB

Ankunda, V. OAB1402

Annequin, M. OAD0403

Anoma, C. OAD3903

Anosike, C. OAD3603

Antonov, R. OAD2105

Anyona, M. OAC1903

Aondoakula, G. OAD3302

Aoun, Z.‐M. R. OAD0805

Apamo, P. OAC0703

Apiputthipan, R. OAE2904

Araújo de Oliveira Silva, J. OAE3105

Aragon, A. OAB3404

Archary, D. OAX1104LB

Archary, M. OAB2306LB, OAX1105LB

Arellano, E. R. OAE3106LB

Arenas‐Pinto, A. OAB0905, OAB3402, OAX1106LB

Arendse, J. OAB4102

Armas‐Kolostroubis, L. OAC2805

Arnold, H. OAE2603

Arora, L. OAD3905

Arora, P. OAC2806LB

Aruku, C. OAD2404

Asante‐Appiah, E. OAB0105LB

Asewe, M. OAC1903

Ashie, M. OAD3302

Ashinze, P. OAD2404

Asif, M. OAC1305

Aslanabadi, A. OAA3002

Asselin, J. OAC4203

Aung, H. L. OAC4203

Auriche, M. OAC1905

Avettand‐Fènoël, V. OAB0102

Avettand‐Fenoel, V. OAA1503

Avihingsanon, A. OAC2806LB

Awad, K. OAF2502

Awotoye, J. OAC1304

Ayakaka, I. OAB2006LB

Ayeh, P. K. OAD3906LB

Ayo, I. OAD3302

Azurunwa, O. OAD3302

### B

Bärnighausen, T. OAF2506LB

Bélanger, É. OAA2703

Bacha, J. OAB2003

Badley, A. D. OAA1506LB

Bagasol Jr., E. OAE3104

Baguso, G. N. OAF3504

Bahemana, E. OAB2304

Bahemuka, U. OAB1402

Bahia, F. OAB3404

Bajis, S. OAE0505

Bakari, M. OAB2006LB

Baker, D. A. OAB1403, OAX1106LB

Bako, H. OAD3302

Balak, D. OAC0206LB

Balakasi, K. OAE4004

Baltrusaitis, K. OAA2705

Balusu, R. OAA1506LB

Bamford, A. OAX1105LB

Banda, P. OAE3102

Banerjee, P. OAC1903

Bani‐Sadr, F. OAB0102

Banik, S. OAF3504

Bansi‐Matharu, L. OAX1102LB

Bar, K. OAA3006LB

Baral, S. OAX1103LB

Barbosa, M. A. OAE3105

Baril, J.‐G. OAB2305

Barlow, M. OAB0905, OAB3402

Barnabas, S. OAA3803

Barr‐DiChiara, M. OAE0505

Barriga Talero, M. A. OAE1603

Barrows, D. OAA3805

Bascombe, F. OAB0905, OAB3402

Basenko, A. OAF3503

Bashorun, A. OAC1304

Basnayake, M. OAD1805

Batista, W. OAB4103

Bauermeister, J. A. OAD0803

Baumhart, C. OAE3102

Bbosa, N. OAB1402

Beaumont, S. OAF3202

Beckey, K. OAB1405LB

Behuhuma, O. OAA3803

Behumuma, N. OAB3406LB

Bekker, L.‐G. OAX1102LB

Bello Raji, H. OAC1304

Benalycherif, A. OAC1905

Benedetti, M. OAC2803, OAC4202

Benites, C. OAD2403, OAF2505

Benitez, C. OAB4103

Benoliel Rocha, T. OAC1302

Berardi, J. OAA1502

Berchmans Niyibizi, J. OAB0104

Berenger, C. OAD0403

Berghammer‐Böhmer, R. OAB4106LB

Berianidze, L. OAF1705

Berini, C. A. OAA1005

Bernard, E. J. OAF3202

Bernard, N. F. OAA1005

Bertoni Giron, L. OAA1002

Betts, M. R. OAA3006LB

Bhandari, R. OAA3804

Bhebhe‐Moyo, A. OAE3102

Biello, K. B. OAD3602

Bieniasz, P. D. OAA3805

Bilash, O. OAD3305

Bilets, A. OAE1602, OAF2503

Birungi, J. OAE3702

Bissa, M. OAA3003

Biswas, K. OAC1305

Black, J. OAB4106LB

Blackmon, A. OAC2804

Blanco, J. L. OAB3405

Blankespoor, S. OAD3602

Blanquart, L. OAC1905, OAD0403

Blithe, D. OAC2804

Bloch, M. OAB1405LB

Bo, Z. W. Y. OAE0505

Boccardi, A. OAF2505

Boczar, K. OAB2305

Bode, L. OAA2705

Boffelli, D. OAA3806LB

Bogart, L. OAE3704

Boles, J. OAB2306LB

Bonifaz, A. OAX1103LB

Boonruang, J. OAD1803

Boontho, K. OAD1803

Bor, J. OAE2905

Bordignon Antonio, M. OAB2302

Bosch, R. OAA1502

Botha, J. C. OAB2306LB

Boulle, A. OAB4102

Bourassa, C. OAA2703

Bourne, A. OAC4204

Bourrelly, M. OAD0403

Bowman, C. OAA3006LB

Boyer, S. OAC0706LB

Brandenburg, S. OAA1506LB

Bremer, M. OAB4106LB

Bresciani Martins de Andrade, P. OAE0305

Briedenhann, E. OAF1202

Brinson, C. OAB1403, OAB1406LB

Brisson, J. OAD0404

Brites, C. OAB2302, OAB3404

Brody, R. OAA2705

Brown, J. A. OAB2004

Brown, K. OAC2805

Brown, L. B. OAD0803, OAC2806LB

Brun, L. R. OAB3405

Brunet, L. OAC2805

Bruning, J. OAF3203

Buason, T. OAC2204

Bubala, P. B. OAC1904

Buchanan, A. M. OAB2306LB

Budnik, P. OAB0106LB

Bueno da Silva, M. C. OAE0305

Bukenya, J. OAF2506LB

Bukusi, E. OAC1903

Bukusi, E. A. OAC0203

Bula, A. K. OAC1306LB

Bulamba, R. OAC0202

Buono, N. OAC1303

Burger, D. OAX1105LB

Burns, N. OAB2304

Butler, V. OAF1202

Butterly, S. OAC0206LB

Buttram, M. E. OAD2103

Bwakura Dangarembizi, M. OAX1105LB

Bwalya, I. OAE3102

Byott, M. OAB2306LB

### C

C Moreira, R. OAB2302

Cabuso, M. R. OAE3104

Cahn, P. OAX1106LB

Caires, P. OAE2902

Cairns, C. OAB4106LB

Callander, D. OAD2105

Camiro‐Zuñiga, A. OAB4103

Candia, E. OAB4103

Canis, M. OAA3805

Cantos, V. D. OAD0803

Cantu Jungles, T. M. OAA1002

Cardoso, S. OAB0902

Cardoso, S. W. OAB3404

Cardozo, M. OAB4103

Cardozo, T. OAA3003

Carlton, K. OAX1104LB

Carmody, M. OAB3404

Caron‐Roy, S. OAD3305

Carras‐Terzian, E. OAE4005

Carrico, A. OAF3204

Carroll, T. OAA3805

Carter, A. OAC4203

Carter, C. OAD0803

Carter, C. C. OAC2802, OAD0802, OAC2806LB

Casari, F. OAB0904

Caskey, M. OAA1502

Cassetti, I. OAB1406LB

Castellanos, E. OAF1705

Castilho, J. L. OAB2302

Castillo Hernandez, N. OAB2005

Castillo, I. OAF1706LB

Castillo, M. OAF2505

Castro Ramírez, S. OAE1603

Castro‐Arteaga, M. OAD0404

Castro, M. OAX1104LB

Catellani, B. OAB0904

Cavanaugh, J. S. OAE3106LB

Cazier, C. OAB4104

Cearanovski, C. OAE3703

Ceballos, O. P. OAB0106LB

Cervantes, M. OAC1905

Cervo, A. OAB0904

Chéret, A. OAB0102

Chagomerana, M. B. OAC1306LB

Chakrapani, V. OAD2102

Chan, M. K. OAB2306LB

Chancham, A. OAD1803

Chanda, D. OAE3102

Chandravanshi, M. OAA2703

Chang, C. OAB3403

Chang, M. OAD2402

Chang, M. M. OAB1404

Chang, W.‐T. OAA1003

Chappell, E. OAX1105LB

Charles, S. OAE4005

Chartrand‐Lefebvre, C. OAB2305

Chasara, C. OAA3802

Chen, B. A. OAC2804

Chen, H. OAD3606LB, OAE0306LB

Chen, H.‐C. OAA2703

Chen, K. OAX1106LB

Chen, N.‐Y. OAE0502

Chen, T. OAB4106LB

Chen, W. OAB2306LB

Chen, Y. OAC2802, OAD0805

Cheng, C.‐Y. OAE0502

Chetchotisakd, P. OAX1106LB

Chewere, L. OAB2002

Chibuye, J. OAD3304

Chidarikire, T. OAC0704, OAE0505

Chilundo, E. OAF3505

Chilyabanyama, O. OAE3102

Chimpandule, T. OAB2002

Chimpukuso, L. OAC1306LB

Ching, D. A. OAE3104

Chinogurei, C. OAB4102

Chinula, L. OAA2705, OAC1306LB

Chisamba Baila, G. OAE2903

Chitembo, L. OAE3102

Chitsanupong, B. OAF1705

Chiu, T.‐J. OAA2703

Chokephaibulkit, K. OAB0103, OAC2204

Chomont, N. OAA1504

Chougnet, C. A. OAB3403

Chow, E. OAC4203

Chuang, T.‐T. OAC4204

Chumpolkulwong, K. OAA2704

Chun, T. W. OAA1506LB

Chuy, F. OAB0903

Ciglenecki, I. OAD2105

Cindy, A. A. OAF0604

Claassen, C. W. OAE3102

Clark, J. OAD0805

Claussen, L. OAE0302

Clough, L. A. OAA1506LB

Clow, W. OAA3804

Cluesman, S. R. OAD2104

Cochran, Q. OAC2805

Coelho, L. OAD0805

Cohen, C. R. OAC0203, OAC0204

Cohn, L. B. OAA1504

Cojocari, A. OAE3703

Collazos, D. A. OAE1603

Collins, C. OAF1706LB

Collins, S. E. OAB1403

Colson, A. E. OAX1106LB

Cook, P. OAB1403, OAB2303

Coomes, D. OAE0505

Copertino, D. OAA3805

Corção, P. OAB2302

Corbin‐Gutierrez, E. OAC1906LB

Cordeiro, T. OAB2302

Cords, L. OAA1505

Corey, L. OAA3004

Corley, M. OAA1002

Cornelisse, V. OAC4203

Cornell, M. OAE4004

Costa Chaves, G. OAF1203

Costa Leite, I. OAC4202

Costa, M. OAD0403

Cotugno, N. OAA3803

Courey, M. OAF2506LB

Coutinho, C. OAC2803, OAC4202

Couto, A. OAE2605

Cox, S. OAC2806LB

Creegan, M. OAA2704

Cressey, T. OAB2306LB

Cresswell, F. V. OAB1402

Cristina Gaspar, P. OAC1302

Crofoot, G. OAB1403

Cross, S. OAE0504

Crowell, T. OAB2304

Crusells, M. J. OAB3405

Cruz Villanueva, L. OAC1905

Cumaquela, E. OAF3505

Cunha, M. OAC4202

Cunningham, P. OAC0206LB

Curran Fabregas, A. OAB1406LB

Curran, A. OAB3405

Cuzin, L. OAB0102

Cyktor, J. C. OAA1502

### D

D'Angelo, A. OAF3204

Däumer, M. OAA1505

Díaz, G. OAC2205

Daama, A. OAC0202

Dadabhai, S. OAA2705

Daka, D. OAE3102

Dakum, P. OAC1304

Danesh, A. OAA3805

Dano, E. OAB2003

Darisheva, M. OAD2402

Dart, C. OAC2804

Dasgupta, S. OAC1305

Dasteh Goli, K. OAA3002

Dat, V. Q. OAB4106LB

Date, A. OAB4104

David, K. OAD2105

Davidson, K. OAA3804

Davies, M.‐A. OAB4102

Davis, M. OAX1103LB

Davis, R. E. OAD2103

Dayton, M. OAA3804

de Albuquerque Moraes, C. OAB2302

de Almeida Rodrigues, M. G. OAE0305

de Barros Campelo Júnior, E. OAE0305

De Cecco, C. N. OAB3403

de França Diniz Rocha, V. OAE0305

De Miguel, M. OAB3405

De Paris, K. OAA1004

de Ruiter, A. OAC2805

de Souza Mendes, G. OAE0305

De‐Almeida, C. OAB0902

De‐Brito, P. OAB0902

Deac, A. OAD3605

Deeks, S. G. OAA1504

DeJesus, E. OAB1403

Delobel, P. OAA1503

Delpierre, C. OAB0102

DeMarco, C. T. OAA3006LB

DeMarrais, P. OAA2705

Denny, T. OAA3006LB

Deprez, A. OAC1905

Develos, M. OAE3106LB

Devine‐Ducharme, V. OAB2305

Devlin, B. OAC2804

Devnarain, N. OAX1104LB

Dezanet, L. OAE2902

Dhakal, M. OAF3504

Dharejo, Z. OAC0705

Di Benedetto, F. OAB0904

Di Gregorio, S. OAB3405

Diamond, T. OAB0105LB

Dias, P. OAC1306LB

Dibba, B. OAC0706LB

Diero, L. OAC0204

Dietl, A. OAA3806LB

DiFrancesco, R. OAA1502

Dilling, RT. OAA3805

Dittmer, U. OAA1505

Dlamini, A. OAE3103

Dlamini, P. OAB4104

do Socorro Marques de Oliveira, R. OAC1302

Dobbs, P. D. OAD2103

Doblecki‐Lewis, S. OAD0803

Doerflinger, M. OAA3804

Domingo, P. OAB3405

Dominguez‐Rodriguez, S. OAA3803

Dong, K. L. OAA2706LB

Doria‐Rose, N. OAX1104LB

Dorman, E. OAC2804

Dorward, J. OAB2004

dos Santos, A. OAB2303

Dourado, I. OAE2902

Dovel, K. OAE4004

Doyle, J. OAB4104

Drake, A. OAE0505

Drammeh, S. OAC0706LB

Driemeyer Correia Horvath, J. OAE0305

Dube, M. OAB4104

Duerr, A. OAC2205

Dukashe, M. OAE2905

Duncan, D. T. OAF3204

Duplessis, C. OAA3004

Duque Vizcaino, M. OAB2005

Durand, M. OAA2703, OAB2305

Durier, C. OAA1503

Durier, Y. OAC2204

Duvivier, C. OAB0102

Dvory‐Sobol, H. OAB1406LB, OAX1106LB

### E

Edelman, A. OAC2804

Edkins, L. OAB4106LB

Edwards, R. J. OAA3006LB

Egoavil‐Espejo, R. OAB3403

Egwu, J. E. OAC0205

El Atrouni, W. OAA1506LB

El‐Bassel, N. OAD2402

El‐Far, M. OAB2305

Elbirt, D. OAB0106LB

Elliot, E. OAB0106LB

Ellis, T. OAD2106LB

Elmubarak, M. OAF2502

Elrayah, A. OAF2502

Elsner, C. OAA1505

Álvarez, E. OAA3005

Elyanu, P. OAB4104

Engamba, D. OAE3102

Engen, P. A. OAA1002

Epalza, C. OAB2306LB

Eriksson, M. OAB4106LB

Erlandson, K. OAB2303

Eron, J. OAB1403

Esbjörnsson, J. OAA2702

Escolano, A. OAA3002

Eshun‐Wilsonova, I. OAB1402

Espinosa Ortiz, A. OAA1504

Esser, S. OAA1505

Esteban, M. OAA3005

Etwaru, D. OAF3204

Euvrard, J. OAB4102

Euzebio de Lima, C. OAF1705

Evans, M. E. OAB2003

Ezenri, G. OAD3603

### F

Falconer, J. OAB4106LB

Fan, J. OAE0306LB

Fang, C. OAD0406LB

Farias, A. OAC4202

Fatti, I. OAB4106LB

Faye, A. OAC1905, OAD0403

Fedorchenko, V. OAD3605

Feijó Andrade, R. G. OAE0305

Fera, D. OAA3002

Ferdinand, R. OAB4106LB

Fernandes de Camargo, R. OAE3105

Ferrari, G. OAA3004, OAA3006LB

Fiedler, M. OAA1505

Fields, S. OAD0805

Filippovych, M. OAD0406LB

Finzi, A. OAA2703

Fiorentino, M. OAD0403

Fisher, M. OAF3203

Fittipaldi, J. OAB0902

Fly Phiri, J. OAB2002

Flynn, P. OAA2705

Foare, R. OAA1503

Ford, D. OAB2306LB, OAX1105LB

Fortin, C. OAB2305

Fournier, B. OAD3305

Fox, J. OAX1102LB

Fox, M. C. OAB0105LB

Franceschini, E. OAB0904

Franchini, G. OAA3003

Frank, I. OAD0803

Freeman, M. L. OAA1502

Freire Maresca, A. OAD0403

Friedman, S. OAD0406LB

Fromentin, R. OAA1504

Fry, S. OAB0103

Frye, V. OAD2402

Furch, B. OAA3004

Fusco, J. S. OAC2805

Fuszard, M. OAB0105LB

### G

Gärtner, K. OAB2306LB

Gómez, C. E. OAA3005

Güerri, R. OAB3405

Gadzayi, M. R. OAC0702

Gallant, J. OAB1403

Gandhi, M. OAF3204

Gaolathe, T. OAE3704

Garay, M. G. OAA3002

García, Á. OAD2403

Garcia Ferreira, A. C. OAE0305

Garcia‐Fraile, L. J. OAB3405

Garcia, A. OAB4103

Gare, J. OAC0206LB

Garges, E. OAA2704

Garret, N. OAX1102LB

Garrett, N. OAB1402, OAB2004

Garrib, A. OAE3702

Gatanaga, H. OAB0106LB, OAB1406LB

Gaur, A. OAB1403

Gaur, A. H. OAB0103

Gengiah, T. N. OAX1104LB

Ghahari, N. OAA1005

Gharib, Y. OAB0905, OAB3402

Ghosh, D. OAB2303

Ghosh, S. OAA3004

Ghosn, J. OAC1905, OAB0106LB

Giaquinto, C. OAA3803, OAB2306LB, OAX1105LB

Giarletta, L. OAB3403

Gichuru, E. OAC1903

Gideon, E. OAC1304

Gilbert, L. OAD2402

Gill, K. OAD0802

Gilmour, J. OAA2702

Gilson, R. OAB0905, OAB3402

Girard, G. OAC1905

Gironda, C. OAX1103LB

Giusti, P. OAF2505

Glatt, T. OAA3004

Glenn Fowler, M. OAA2705

Go, V. OAD0805

Godbole, R. OAF2506LB

Goeiman, H. OAB4102

Goh, B. OAD1805

Goldenberg, S. G. OAC0202

Gomani, G. OAB2002

Gomez, J. L. OAD2104

Gonçalves Veloso, V. OAC4202

Gonese, G. OAC0702

Gonzalez Cortes, M. A. OAE1603

Gosnell, B. OAB4106LB

Goujard, C. OAA1503

Govender, N. P. OAB4106LB

Grangeiro, A. OAE2902

Grant, P. OAB2306LB

Grau, L. OAD3605

Grazia Lain, M. OAA3803

Greaves, W. OAB0105LB

Greco, D. OAE2902

Grigorchuk, E. OAD2402

Grinsztejn, B. OAB0902, OAB2302, OAC2803, OAC4202, OAD0805, OAC2806LB

Grint, D. OAB1402

Grov, C. OAF3204

Grzelak, L. OAA3806LB

Guadamuz, T. E. OAD0402

Guadarrama, A. OAD2105

Guaraldi, G. OAB0904

Gubler, M. OAB1405LB

Guemne Kapche, E. D. OAF3205

Guerra, S. OAA3005

Guerrini, G. P. OAB0904

Gueye, C. B. D. OAF3206LB

Guignard, A. OAC2805

Guillen Cañizares, J. R. OAE1603

Guimessa Mezation, R. L. OAF3205

Gupta, A. OAB2306LB

Gupta, S. K. OAB1406LB

Guy, R. OAC4203

Gvozdetska, O. OAE1602

Gyamerah, A. O. OAD3906LB

### H

Haddock, B. OAA1504

Hafeez, F. OAC0705

Hahn, W. OAA3004

Haire, B. OAC4203

Halley‐Stott, R. P. OAB4106LB

Hamaker, B. OAA1002

Hamby, T. OAB2304

Han, M. OAE0306LB

Hankins, C. OAX1104LB

Hanl, P. OAF3203

Hare, J. OAA2702

Harkey, K. OAC1903

Harkoo, I. OAC2802, OAX1104LB

Harrer, T. OAA1505

Harrison, T. S. OAB4106LB

Harutyunyan, A. OAF1205

Hashim, R. OAD2105

Hassan, A. S. OAA2702

Hassan, F. OAF1206LB

Hatziioannou, T. OAA3805

Hawley, I. OAD0802

Hayab, S. OAD3302

Haynes, B. F. OAA3006LB

Haynes, R. OAB2003

Heaps, A. OAC2803

Heath‐Paynter, D. OAC4203

Heath, S. OAA3004

Hecht, J. OAC2203

Hedgcock, M. OAB1406LB

Heger, E. OAA1505

Hein, P. A. OAF2504

Heise, M. J. OAF3204

Hellard, M. OAC4203

Hellström, E. OAB0103

Hempongchanachai, C. OAE2904

Hendricks, S. OAA3004

Henriette de Lannoy, L. OAC1302

Hensen, B. H. OAD3303

Hentzien, M. OAB0102

Heptinstall, J. OAA3004

Herce, M. E. OAC1306LB

Hernández‐Leyva, M. OAC2203

Hetman, L. OAE1602

Hill, A. OAE0504, OAB2006LB, OAB3406LB

Hinestrosa, F. OAB1405LB

Hiransuthikul, A. OAD1803

Hirschhorn, L. OAE3704

Hirshfield, S. OAD2103, OAF3204

Hoagland, B. OAC2803, OAC4202

Hocqueloux, L. OAB0102

Hodgson, M. OAC0206LB

Hoege, D. OAE2903

Hoeth, D. OAC2803

Hoffman, I. OAC1306LB

Hofmann, E. OAX1106LB

Hoh, R. OAA1504

Hojilla, J. C. OAD0803

Hollingshead, B. M. OAF3203

Holmes, C. B. OAE2903

Honermann, B. OAX1103LB

Horvath, K. J. OAF3204

Hossain, T. OAA3802

Hosseinipour, M. C. OAC1306LB

Hsieh, A. OAA2702

Huaman, M. A. OAB3403

Huang, E. OAC2203

Huang, H. OAD3606LB

Huang, J. OAA3004

Huang, P. OAC4204

Huber, A. OAE0503

Hughto, J. M. W. OAD3602

Hung, C.‐C. OAB1406LB

Hunter, E. OAA2702

Huryn, D. OAA2703

Huynh, T. T. OAA3805

Hypolito, L. OAB2302

Hyrien, O. OAA3004

### I

Idabor, C. OAD3603

Idahosa, A. OAB1402

Idepefo, F. OAC0205

Idipo, D. OAC1303

Ifeanyi, O. J. OAF0603

Imani, B. OAD2105

Imarhiagbe, C. OAD3302

Imerbsin, R. OAA2704

Inyali, J. OAE1605

Isah, A. OAD3603

Isernia, V. OAC1905

Ismério Moreira, R. OAC4202

Ismael, N. OAB2006LB

Isnard, S. OAA1005

Ivorra, J. OAC2804

### J

J, R. OAD3905

Jacobs, J. OAC2803

Jacobs, T. OAB4102

Jaffar, S. OAE3702, OAB4106LB

Jafta, L. OAX1105LB

Jagnarine, T. OAB4103

Jahn, A. OAB2002

Jallow, A. E. OAC0706LB

Jamil, M. S. OAC0705

Jammeh, K. OAC0706LB

Janamnuaysook, R. OAD1803

Jankowski, C. OAB2303

Janyam, S. OAD0405, OAE2606LB

Jao, J. OAA2705

Jarrett, S. OAD2106LB

Jarvis, J. N. OAB4106LB

Jenkins, S. OAF1202

Jensen, B.‐E. O. OAA1505

Jewell, A. OAB0106LB

Jiamsiri, S. OAC2204

Jiang, Y. OAE0306LB

Jimenez, H. OAB0903

Jin, C. OAE0306LB

Jjuuko, B. N. OAE2604

Johnson, C. OAE0505

Johnson, L. F. OAB4102

Johnson, M. O. OAF3204

Johnson, T. OAF1206LB

Jonas, K. OAD0402

Jones, B. OAB0106LB

Jones, R. B. OAA1502

Jones, RB. OAA3805

Jongrakthaitae, S. OAA2704

Juma, L. OAC1903

Jupimai, T. OAD0805

### K

Kabanda, D. OAB4106LB

Kafeero, P. OAB1402

Kaiser, R. OAA1505

Kaleebu, P. OAB0104

Kalima, M. OAE3102

Kalombo, C. OAE3102

Kalonga, F. OAB2002

Kalu, D. OAD3302

Kalu, G. OAF0603

Kalu, K. OAE1605

Kamau, E. W. OAB2006LB

Kamau, J. OAE4006LB

Kamgwira, Y. OAE2903

Kampamba, D. OAE3102

Kampilimba‐Mwango, L. OAE3102

Kamrunnahar, K. OAB2306LB

Kamtsendero, L. OAE4004

Kamulaza, L. OAA1004

Kamupira, M. OAC2804

Kamuti, E. OAX1104LB

Karanjkar, V. OAB4105

Kareithi, T. OAC1903

Karita, E. OAA2702

Kasale, D. OAE2903

Kasaro, M. OAA1004

Kasonde, M. OAE3102

Kasozi, C. OAC0204

Kassim, S. OAB1402

Kasujja, F. X. OAE3702

Kaur, J. OAD2102

Kauss, B. OAD1802

Kawalazira, G. OAE2903

Kay, A. OAB4104

Kazibwe, D. OAD3902

Keawboon, B. OAA2704

Kebotsamang, K. OAE3704

Keipo, V. OAD3903

Keith, K. OAE2603

Kekitiinwa, A. R. OAB2306LB, OAX1105LB

Kelleher, A. OAC0206LB

Kelley, C. F. OAD0805

Kelly III, R. OAA2704

Kelly‐Hanku, A. OAC0206LB

Kenny, A. OAC4205

Kersey, K. OAB0103

Kerwin, M. OAA3002

Keshavarzian, A. OAA1002

Kgotlaetsile, K. OAE3704

Khaba, T. OAA3802

Khan, A. OAD3905

Kharandiuk, I. OAD3605

Khasowa, D. OAB2006LB

Khumcha, B. OAC2204

Khuu, V. OAB2303

Kibuuka, H. OAB2304

Kiene, S. OAC0202

Kigen, K. OAE3705

Kigozi, G. OAC0202

Kiilu, C. OAX1105LB

Kikaire, B. OAB0104

Kikonyogo, R. OAD3902

Kilembe, W. OAA2702

Kim, J. OAB0105LB

Kimani, M. OAD2105

King, R. OAE2905

Kintu, A. OAD0802

Kiptinness, C. OAC1903

Kisare, M. OAB0106LB

Kithinji, C. OAD2105

Kitonsa, J. OAB1402

Kityo, C. OAB2306LB, OAX1105LB

Kivuyo, S. OAE3702

Kivuyo, S. L. OAB4106LB

Kiwanuka, N. OAC2802

Kiweewa, F. M. OAC2802

Klein, O. D. OAA3806LB

Kleinbeck, K. OAC2804

Klopfer, S. OAB0105LB

Klopfer, S. O. OAB1406LB

Knops, E. OAA1505

Ko, W.‐C. OAA1003

Koech, E. OAC0703

Koh, E. OAC1906LB

Kohli, M. OAB3402

Kokiçi, J. OAA2702

Komba, L. OAB4104

Konah, S. OAF3502

Kondratyuk, S. OAF1203

Kongjaroen, Y. OAD0402

Korutaro, V. OAA2705

Kosalaraksa, P. OAB0103

Kosciow, B. OAD0803

Koulibaly, C. T. OAF3206LB

Koup, R. A. OAX1104LB

Koutsenok, I. OAD0406LB

Krebs, S. J. OAA2704

Kreider, E. OAA3006LB

Kriews, A. OAA3002

Kritsanavarin, U. OAC2204

Kroemer, G. OAA1005

Krummenauer, A. OAC1302, OAC1902

Ku, H.‐C. OAA1003

Ku, S. W.‐W. OAC4204, OAE0502

Kubeka, A. OAD0802

Kublin, J. OAA3004

Kuhn, L. OAA2705

Kulik, G. OAB2303

Kulp, D. OAA3004

Kulp, D. W. OAA3002

Kumar, A. OAB2306LB

Kumar, P. OAB1405LB

Kumar, P. N. OAX1106LB

Kumwenda, W. OAC1306LB

Kuru‐Utumpala, J. OAD1805

Kusemererwa, S. OAX1102LB

Kusen, M. OAD1805

Kwaghga, L. OAC0205

Kyasanku, E. OAC0202

Kyazze, D. OAB4106LB

### L

Lübke, N. OAA1505

La Rosa, A. OAB3403

Lacerda, M. V. G. OAB3404

Lacson, R. OAB2003

Laenger, N. OAA3002

Laguing, J. OAE4003

Laker Odongpiny, E. OAB1402

Lalloo, U. OAB0103

Lama, J. R. OAB3403, OAC2806LB

Lamaury, I. OAB0102

Lancien, M. OAA2706LB

Landay, A. OAA1002

Landman, R. OAC1905

Landovitz, R. OAC2803

Landovitz, R. J. OAD0805

Lankiewicz, E. OAX1103LB

Lannap, F. OAD2404

Lavender, D. OAE2603

Lawrence, D. S. OAB4106LB

Lawrence, N. OAD0805

Lawrenson, G. OAB3402

Le Gac, S. OAC1905

Le, T. OAB4106LB

Lebese, L. OAC0704

Lebina, L. OAB3406LB, OAX1102LB

Lechartier, M. OAA1503

Lederer, R. OAA3004

Lee, G. Q. OAA3805

Leggat, D. J. OAA2704

Legkostup, L. OAE1602

Lella, P. OAC2202

Lemoine, M. OAC0706LB

Leon, M. OAB3403

Leslie, H. H. OAD3906LB

Levi, J. OAE0504

Levis, B. OAC2805

Lewin, S. R. OAA3804

Lewis‐Kulzer, J. OAC0204

Lewis, L. OAB2004, OAX1104LB

Lewis, T. OAE4005

Li, C.‐W. OAC4204, OAE0502

Li, M. OAB0105LB, OAB1405LB

Li, X. OAD3302

Liang, L. OAE0306LB

Liang, S. OAE0306LB

Liao, C.‐T. OAA1003

Libau, E. OAE2602

Liberty, A. OAB0103, OAX1106LB

Lichterfeld, M. OAA2706LB

Lightfoot, M. OAD3906LB

Lim, S. H. OAD0402

Lin, B.‐Y. OAA1003

Lindemann, M. OAA1505

Lippman, S. A. OAD2104, OAD3906LB

Lishimpi, K. OAE3102

Listerud, L. OAD0803

Littwitz‐Salomon, E. OAA1505

Liu, H. OAB1403

Liu, J. OAE0306LB

Liu, S.‐Y. OAB1406LB

Llamoso, C. OAB1406LB, OAX1106LB

Lockman, S. OAA2705

Logie, C. OAD3305

Loh, P. OAD1805

Longenecker, C. T. OAB3403

Lopardo, G. D. OAB0106LB

Lopez Mendez, J. OAF1204

Lopez, P. OAE3104

Lorenzo Redondo, R. OAA1002

Losso, M. OAC2806LB

Loubser, S. OAA3803

Louw, C. OAC2802

Loyse, A. OAB4106LB

Lu, H. OAE0306LB

Lu, P.‐L. OAX1106LB

Luetkemeyer, A. F. OAB1405LB

Luhmann, N. OAE0505

Luke, J. S. OAF3502

Lunkuse, J. F. OAB1402

Luque, R. OAB4103

Luz, E. OAB2302

Luzuriaga, K. OAA2705

Lv, Y. OAE0306LB

### M

Mabanga, T. OAA1005

Macías‐González, F. OAC2203

Macheso, S. OAB2002, OAE4004

Mackiewicz, L. OAA3804

Madlala, P. OAA3802

Madruga, V. OAB3404

Madybaeva, D. OAD3605

Magaia de Abreu, A. P. OAF3505

Magno, L. OAE2902

Mahlobo, B. OAA3802

Mahmoudi, T. OAA3802

Mahomed, S. OAB1402, OAB2004, OAX1104LB

Mahuvakar, S. OAF3204

Maiga, A. OAA3803

Maina, N. OAC1303

Maischack, F. OAA1505

Makhooa, N. OAF0605

Makinson, A. OAB0102

Makoko, N. OAB4106LB

Makonokaya, D. OAC1306LB

Makunike‐Chikwinya, B. OAC0702

Malahleha, M. OAC2802

Malambo, W. OAC1904

Maldarelli, F. OAA3003

Maleestharn, A. OAC2204

Malen, R. OAC1903

Malhotra, A. OAE0505

Malunga, R. OAB2002

Mandalakas, A. OAB4104

Mandisarira, J. OAC0702

Manentsa, N. OAB3406LB

Manojai, N. OAE2904

Mansoor, L. E. OAC2802, OAX1104LB

Manyele, C. OAD3304

Maomela, T. OAC0704

Mapfumo, C. OAC0702

Maphalala, L. OAB4106LB

Mar, H. OAA1502

Maradan, G. OAD0403

Marcos‐Villar, L. OAA3005

Margolis, D. OAA3006LB

Markarian, C. OAC2805

Marsh, A. OAC0704

Martin, C. OAB0106LB

Martin, T. OAA3004

Martinez‐Picado, J. OAA1505

Martinez‐Pourcher, V. OAB0102

Martinez, E. OAB3405

Masano, A. OAC1306LB

Masaulo, P. OAE0506LB

Mascola, J. OAX1104LB

Masia, M. OAB3405

Masiye, G. OAC1306LB

Masombuka, X. OAB2004

Massa, P. OAE2902

Masson, M. OAE4005

Masud, H. OAD2105

Maswai, J. OAB2304

Matanje, B. OAE4004

Mate, C. OAE2605

Matendawafa, A. W. OAF1206LB

Mateo González, J. I. OAB0106LB

Mathenge, J. OAE4006LB

Matoga, M. OAC1306LB

Matos, C. M. OAE3105

Matse, S. OAE3103

Matthews, R. P. OAB1405LB

Maurer‐Stroh, F. OAE2602

Mawandia, S. OAE0302

Mayanja, Y. OAB0104

Mayer, K. H. OAD3602, OAC2806LB

Mbeje, S. S. OAB2004

McCauley, M. OAD0805

McCloskey, J. OAC4203

McCloskey, N. OAA3004

McCrimmon, T. OAD2402

McElrath, M. J. OAA3004

McFarland, W. OAC2202

McGuirk, J. P. OAA1506LB

Mchantaf, G. OAA1503

McKellar, M. S. OAB1403, OAC4205

McMahon, J. OAB1403

McMahon, S. A. OAF2506LB

Meanley, S. OAD0803

Medjahed, H. OAA2703

Meiffredy, V. OAA1503

Meintjes, G. OAB4102, OAB4106LB

Meisner, A. OAC1903

Mellors, J. OAC2803

Mellors, J. W. OAA1502

Mellouk, O. OAF1203

Melo, M. B. OAA3002

Mena, L. OAE0304

Menacho Alvirio, L. A. OAC2205

Menard, A. OAB0102

Mendelsohn, J. B. OAD3305

Mendes Andrade, J. R. OAE0305

Mendes, R. C. OAE0305

Mendez Vasquez, M. J. OAB0106LB

Mendo Ze, G. M. OAF1703

Mendonça Collaço Véras, N. OAC1302

Mendoza‐Lopez, C. OAA3802

Menezes, C. OAB4106LB

Mengesha, B. OAB2305

Mensah, C. OAC1304

Merad, Y. OAA2706LB

Mercier, R.‐C. OAB1406LB

Messier‐Peet, M. OAB2305

Messinga Nkonga, J. OAF3205

Metallidis, S. OAB0106LB

Meyer, L. OAA1503

Mfinanga, G. S. M. OAB4106LB

Mfinanga, S. OAB0104, OAE3702

Michael Mkwashapi, D. OAC0204

Michard, F. OAD0403

Michels, D. OAD0403

Michielsen, K. OAD3303

Middelkoop, K. OAD0805

Milala, B. OAC1306LB

Milford, C. OAD0802

Milic, J. OAB0904

Miller, A. P. OAC0202

Miller, GI. OAA3805

Miller, I. OAA1502

Millett, G. OAX1103LB

Mimiaga, M. J. OAD3602

Mims, D. K. OAB1404

Miner, M. OAA3004

Mirzazadeh, A. OAF2502

Mitchell, J. OAC0206LB

Mkandawire, B. OAD3304

Mkhize, N. N. OAX1104LB

Mkhontfo, M. OAE3103

Mkochi, T. OAC1306LB

Mngqibisa, R. OAX1105LB

Moagedi, M. OAE3704

Moawed, S. OAB0903

Mocello, A. R. OAD2104

Mogaetsho, E. OAE3704

Mohammed, P. OAB0106LB

Mohring, A. OAA1505

Moktan, D. OAF3504

Molechan, C. OAA3803

Moller, K. OAB3406LB

Molloy, S. F. OAB4106LB

Monceaux, V. OAA1503

Monroe‐Wise, A. OAE0505

Montefiori, D. OAA3006LB

Montevirgen, N. OAB2003

Montgomery, E. T. OAD0802

Montgomery, R. OAF3503

Moodley, M. OAA3802

Moodley, R. OAB0106LB

Moolla, H. OAB4102

Moonga, G. OAC1904

Moore, P. OAX1104LB

Moosa, M. Y. S. OAB4106LB

Mora, M. OAC1905, OAD0403

Morales‐Ramirez, J. OAB1405LB

Morris, L. OAX1104LB

Morrocchi, E. OAA3803

Morse, G. D. OAA1502

Moscovich, T. OAE0304

Mosepele, M. OAE3704

Mosery, N. OAD0802

Moshomo, T. OAE3704

Mosses, A. OAB4106LB

Mosugu, T. OAC2203

Mouquet, H. OAA1503

Mourelo, S. OAB3405

Moyo, B. OAC0702

Mpango, L. OAE3103

Mpembeni, R. OAF2506LB

Mphande, M. OAE4004

Mponponsuo, K. OAB1403

Mtenga, A. OAB0104

Mthombeni, K. OAF1202

Mudany, M. OAD3604

Mudhume, V. OAB0104

Mudzanani, H. OAD1804

Muecksch, F. OAA3805

Muema, J. OAE4006LB

Mugagga, I. OAD3902

Mugamba, S. OAC0202

Mugambi, M. OAC1903

Mugerwa, H. OAX1105LB

Mugun, C. OAD2105

Muhammed, F. OAD3302

Muhumuza, U. OAD0804

Muikamba, Z. OAB2006LB

Mujuru, H. A. OAX1105LB

Mulenga, C. OAA1004

Mulenga, L. B. OAE3102

Mulenga, M. OAE3102

Mulengwa, D. OAB4104

Mullick, S. OAF1202

Munseri, P. OAB2006LB

Munthali, A. OAB4104

Murphy, K. L. OAB4106LB

Murray‐Krezan, C. OAB3404

Murray, M. OAB2305

Murtala Safana, K. OAC0205

Murwira, S. OAC0702

Musau, O. OAE0506LB

Mushangwe, B. OAC0702

Musick, B. OAC0204

Musonda, M. OAD3304

Musonda, P. OAA1004

Musosha, M. OAE3102

Mussini, C. OAB0904

Mustafa, K. OAC0705

Mutinta‐Mbewe, M. OAE3102

Muvwimi, M. OAE3102

Muwanguzi, P. OAD2405

Muwanula, N. OAE0503

Muyembe, G. OAE3102

Mvalo, T. OAC1306LB

Mwakyosi, L. B. OAE2604

Mwansa, W. OAD3304

Mwendar, G. OAD2105

Mwitumwa, M. OAE3102

### N

N'Guessan, J. OAD3903

Nabbuto, J. OAB1402

Nabitaka, L. K. OAC1303

Nabukenya, S. OAX1102LB

Nabunya, R. OAD2405

Nahass, R. G. OAX1106LB

Nahumuza, Z. OAD0804

Naicker, N. OAA3004

Naidoo, N. OAF1202

Naik, A. OAA3806LB

Naing, E. OAA1502

Nair, G. OAC2802

Nakabuye, S. OAX1105LB

Nakakande, J. OAC0202

Nakanga, C. OAC1306LB

Nakigozi, G. OAC0202

Nalubega, S. J. OAD2405

Nalugoda, F. OAC0202

Namakoola, I. OAE3702

Namusukuma, N. OAE3102

Nandakumar, S. OAB4104

Nanyange, B. OAD0804

Nanyonjo, A. OAD3902

Nanyonjo, M. OAB1402

Naqvi, A. OAC2803

Narayanan, P. OAD1805

Narpala, S. OAX1104LB

Narrea, J. OAB3403

Nastouli, E. OAB2306LB

Natukunda, E. OAB0103

Nayiga Kalanzi, B. OAX1102LB

Nazer, S. OAC2803

Ndhlovu, A. OAE3102

Ndhlovu, Z. OAA3802

Ndlovu, N. OAC2806LB

Ndow, G. OAC0706LB

Ndung'u, T. OAA3802, OAA3803, OAA2706LB

Ndunyu, L. OAC0203

Neilands, T. B. OAD2104, OAF3204, OAD3906LB

Nel, D. OAD1804

Nel, J. S. OAB4106LB

Nelson, L. E. OAD3906LB

Ngige, A. OAD3603

Ngoma, G. OAE3102

Ngubane, T. OAA3802

Ngure, K. OAC1903, OAF2506LB

Nguyen, H. OAX1105LB

Nhanombe, T. OAF3505

Nichols, C. OAF2506LB

Niehaus, E. D. OAC4205

Nigussie, N. OAE0302

Nijhuis, M. OAA1505

Nillos, B. E. OAE3106LB

Njengele‐Tetyana, Z. OAD0802

Njoroge, M. OAE4006LB

Nkale, J. OAC0202

Nketsiah, K. OAE4002

Nkosi, T. OAD0802

Nkuranga, J. OAB2006LB

Noestlinger, C. OAD3303

Norcross, C. OAB1402

Northbrook, S. OAC2204

Nsubuga Nyombi, T. OAC1303

Nuñez Pumacallahui, S. K. OAE0303

Nugent, J.‐A. OAD2106LB

Nunes, E. OAB0902

Nurmsoo, S. OAF3506LB

Nuttall, J. OAC2804

Nwachuya, C. OAD3603

Nwagbo, C. OAD3306LB

Nweze, D. OAD3306LB

Nwoke, U. OAB0105LB

Nyabiage, A. OAC0703

Nyamaizi, A. OAD0802

Nyawanda, B. OAB0104

Nyirenda, M. OAE3702

Nyirenda, R. K. OAC1306LB

Nyirenda, W. OAC1306LB

Nzoolo, R. OAE3102

### O

O'Brien, B. E. OAB4104

O'Cleirigh, C. OAD3602

O'Neal, I. OAC2202

O'Reilly, M. OAB1406LB

Obeng, B. OAC0206LB

Ocheret, D. OAF1705

Odak, J. OAC0203

Odebunmi, T. OAD3302

Odhiambo, D. OAC0703

Odhiambo, F. OAC0204

Odongo, P. OAD3604

Ofim, K. OAE0302

Ogbonna, E. OAD3302

Ogbuagu, O. OAB1403, OAX1106LB

Ogirima, F. OAD3302

Ogorry, O. OAE0302

Ogunkola, I. OAF3503

Ogwu, J. OAC1304

Ojok, S. OAD3305

Okeke, N. L. OAC4205

Okoro, U. OAB1405LB

Okpokoro, E. OAC1304

Okyere‐Manu, G. OAB4106LB

Oladokun, O. OAD3302

Olajubu, O. OAD3904

Olalekan Ayodeji, K. OAC1304

Olanrewaju, A. O. OAB1404

Olatosi, B. OAD3302

Olczak, A. OAB0106LB

Olin German, R. OAF3203

Oliveira Leite, B. OAE2902

Oliveira, V. OAB2303

Olivera, J. OAD2403

Olley, M. OAD2404

Olupitan, O. OAC1304

Olutola, A. OAD2404

Omar, A. OAX1105LB

Omari, E. OAC0703

Ombagi, J. OAC0703

Ombajo, L. A. OAB2006LB

Omemo, P. OAC0203

Omodele, R. OAD3302

Omodi, V. OAB2006LB

Omollo, V. OAC1903

Ong'wen, P. OAC1903

Ong, E. J. OAE2602

Ong, J. OAA3804, OAC4203

Onoya, D. OAE2905

Onyango, A. OAE4006LB

Onyehalu, J. C. OAD3603

Oo, A. T. OAE1604

Opatola, T. OAD3904

Ordóñez, C. E. OAE0304

Orkin, C. OAB1406LB

Ornelas Pereira, I. OAC1302, OAC1902

Ortblad, K. OAC1903

Ortega, J. A. OAE1603

Osman, F. OAX1104LB

Otieno, E. OAB2006LB

Otieno, P. OAC1903

Otiso, L. OAE0506LB

Ott, M. OAA3806LB

Otwombe, K. OAA3803

Ouvrard, F. OAC1905

Ovies, C. OAA1502

Owarwo, N. OAB1402

Owino, E. OAC0703

Owira, P. OAD2105

Owor, M. OAA2705

Owuor, N. OAC0703

Owuoth, J. OAB2304

Owusu, S. E. OAE4002

Oyaro, P. OAE0506LB

Oyedele, T. OAD0805

Oyeledun, B. OAD3302

Oyeniyi, R. OAD3904

Oyuga, R. OAC0703

Ozitiosauka, W. OAC1306LB

Ozorowski, G. OAA3004

### P

Pérez, F. OAB4103

Pínzon Zakzuk, J.f. OAF1204

Page, K. OAB3404

Pagtakhan, R. OAE4003

Pal, V. K. OAA3805

Palanee‐Phillips, T. OAD0802

Palaypayon, N. OAB2003

Palella, F. J. OAA1002

Palich, R. OAB0102

Palkar, A. OAB4105

Pallesen, J. OAA3004

Palma, P. OAA3803

Pansegrouw, J. OAA3802

Pantalone, D. W. OAD3602

Pantoja, C. OAC2205

Parczewski, M. OAX1106LB

Parikh, U. OAC2803

Parker, Z. OAB2304

Parks, K. R. OAA3004

Parsons, M. S. OAA2704

Pasha, M. S. OAC0705

Pasqualotto, A. OAB4103

Pastellides, C. OAB2004

Pastrana, M. OAD2105

Patel, N. OAB1406LB

Pati Pascom, A. R. OAC1902

Patino‐Mateus, J. D. OAE0304

Patpeerapong, P. OAE2904

Patrick, J. OAD2106LB

Pattarayanon, P. OAC2204

Paul, R. OAB2304

Paul, S. OAX1104LB

Paula Betaressi da Silva, A. OAC1302

Payuha, T. OAD1803

Pazgier, M. OAA2703

Pechsamrit, A. OAE2904

Peeler, D. OAF1203

Pelka, K. OAA3806LB

Pellegrini, M. OAA3804

Penner, J. OAB2006LB

Penrose, K. OAC2803

Peppa, D. OAA2702

Peralta, F. OAC1905

Perazzo, H. OAB0902

Perdiguero, B. OAA3005

Pereira Kuss, C. OAE0305

Pereira, G. OAE2602

Peres Queiroz de Paiva, J. OAE0305

Perez‐Brumer, A. OAD0404

Person, A. OAA1504

Pett, S. OAX1105LB

Pett, S. L. OAB0905, OAB3402

Pfannenstiel, K. OAA1506LB

Phaengnongyang, C. OAD0405, OAE2606LB

Phanga, T. OAC1306LB

Phanuphak, N. OAD0805, OAD1803

Phillips, A. OAD3302, OAX1102LB

Phiri, C. OAE3102

Phiri, H. OAE3102

Phiri, K. OAE4004

Phiri, M. OAE3102

Phiri, S. OAE4004

Phuang‐Ngern, Y. OAA2704

Piñeirua‐Menéndez, A. OAC2203

Pignataro, M. F. OAF1203

Pignedoli, C. OAC1905

Pillay, L. OAF1202

Pilotto, J. OAC2806LB

Pintye, J. OAC1903

Pipitone, F. OAC1905

Pius, J. OAD2404

Plagianos, M. OAC2804

Pokaprakarn, T. OAA1004

Polakowski, L. OAA3004

Pollara, J. OAA3006LB

Pongsakul, P. OAC2204

Post, F. A. OAX1106LB

Potloane, D. OAX1104LB

Poverga, R. OAE3703

Pozzetto, B. OAX1104LB

Prado, P. OAD3906LB

Prigann, J. OAA3806LB

Primbetova, S. OAD2402

Prommali, P. OAC2204

Protière, C. OAA1503, OAD0403

Prudence Tatiana, N. M. OAD3303

Prytuliak, P. OAE1602

Psaros, C. OAD0805

Puangmalee, T. OAC2204

Pulido, F. OAB0106LB

Pumphosuwan, P. OAD1803

Puryear, S. OAC2802

### Q

Queiroga de Souza, L. T. OAE3105

Queiroz da Silva, A. OAE3105

Quinn, B. OAC4203

Quintanilha, L. OAB2302

Quintanilla Cantizano, M. S. OAF1702

Quiroga‐Gutierrez, N. OAE0304

### R

Rahman, M. A. OAA3003

Raivoso, M. OAE2605

Ralser, A. OAA3806LB

Ramaiya, K. OAE3702

Ramalho, A. OAB3404

Ramgopal, M. N. OAB1406LB

Ramirez Correa, J. F. OAE1603

Ramirez, S. E. OAB3403

Ramon, J. S. OAE0304

Ramos Iglesias, N. OAB2005

Ramotsababa, M. OAE3704

Rana, R. OAC1305

Ranadive, P. OAB2302

Ratanasuwan, W. OAX1106LB

Ratevosian, J. OAF1706LB

Rathore, L. OAC1305

Rautenberg, C. OAA1505

Reddy, B. OAA1502

Reddy, J. OAC0206LB

Reddy, K. OAA3802, OAA3803

Reddy, N. OAA3802

Reddy, S. OAE3704

Rees, T. OAC4203

Reese, A. OAE2603

Reeves, D. B. OAA1504

Reisner, S. L. OAD3602

Relano Rodriguez, I. OAA3002

Resar, D. OAF1202

Reyes Galindo, N. OAE1603

Reyes Rojas, A. C. OAF1204

Reynes, J. OAB0102

Rhee, M. S. OAB1406LB, OAX1106LB

Riabokon, S. OAF2503

Rice, W. OAD0805

Richard, J. OAA2703

Richards, A. OAD0803

Richards, K. OAF1206LB

Richards, L. OAB4106LB

Ricky, B. OAB2006LB

Rincо́n, G. OAC1905

Rincon, G. OAD0403

Rinehart, A. OAD0805

Rios, C. OAB4103

Ripamonti, D. OAB0106LB

Rittenhouse, K. OAA1004

Rivera‐Buendía, F. OAC2203

Roan, N. R. OAA3806LB

Robb, M. OAA3003

Roberta Pati Pascom, A. OAC1302

Robson, I. OAE4004

Roche, M. OAA3804

Roche, S. OAC1903

Rockstroh, J. K. OAB1406LB

Rodriguez, A. OAA3806LB

Rodriguez, C. A. OAB0103

Rojo, P. OAA3803

Rolim Gonçalves de Alencar, N. OAE0305

Romero Placeres, M. OAB2005

Romero‐Martin, L. OAA1503

Romero, N. OAD2403

Rono, B. OAC0203, OAC1903

Ronot‐Bregigeon, S. OAB1406LB

Rooney, J. OAD0805

Rosadiño, J. D. OAE4003

Rossi, P. OAA3803

Roukens, A. H. E. OAX1106LB

Rountree, W. OAA3006LB

Routy, J.‐P. OAA1005

Rouveix, E. OAD0403

Rowland‐Jones, S. L. OAA2702

Royston, L. OAA1005

Rozanova, J. OAD3605

Ruane, P. J. OAX1106LB

Rucinski, K. OAX1103LB

Ruiter, R. A. OAE2905

Ruiz, R. OAC2202

Rukyalekere Kekitiinwa, A. OAA2705

Rundogo, J. V. OAB4106LB

Rungmaitree, S. OAC2204

Runya, B. OAF1704

Rutananukwa, N. OAB4106LB

Rutstein, S. E. OAC1306LB

Ruzagira, E. OAA2702, OAB1402, OAX1102LB

Ryan, P. OAB3405

Ryzhenko, N. OAF2503

### S

Sabirova, M. OAD3605

Sacdalan, C. OAB2304

Saetun, P. OAA2704

Saez‐Cirion, A. OAA1503

Safren, S. A. OAD0805, OAD3602

Sagaon Teyssier, L. OAC1905

Saggese, G. OAD2104

Sagna, A. OAF3206LB

Saidi, F. OAC1306LB

Sajadi, M. M. OAA3002

Sajjaweerawan, C. OAA2704

Sakabe, S. OAE0305

Sakala, J. OAA1004

Saldana, C. S. OAE0304

Salgado, M. OAA1505

Samsunder, N. OAX1104LB

Samuel, K. D. OAD2103

Sanches, M. OAD3305

Sanchez, M. OAB3403

Sanders, E. J. OAA2702

Santafe, N. OAE1603

Santos, B. OAC2806LB

Santra, S. OAA3006LB

Sanyu, G. OAB1402

Saplagio, N. G. OAB2003

Sassaman, K. OAF3204

Saunders, K. O. OAA3006LB

Schöler, L. OAA1505

Schaafsma, T. OAC1903

Scheck, R. OAA1502

Scheim, A. I. OAD2102

Schiavon Nogueira, R. OAB2302

Schifanella, L. OAA3003

Schmidt, H. OAF3204

Schroeder, T. OAA1505

Schuetz, A. OAA2704

Schuler, E. OAB2304

Schulze zur Wiesch, J. OAA1505

Schutz, C. OAB4106LB

Schwarzer‐Sperber, H. S. OAA1505

Schwarzer, R. OAA1505

Scopel, C. T. OAF0602

Scribner, A. OAB1405LB

Sebastião, Y. OAA1004

Sebro, A. OAB4103

Secco Torres da Silva, M. OAC4202

Seeger, A. OAB4104

Seeley, J. OAX1102LB, OAX1105LB

Segil, J. OAD3602

Semeere, A. OAC0204

SenGupta, D. OAB1406LB, OAX1106LB

Senkoro, M. OAB0104

Seo, D. OAC0706LB

Serebryannyy, L. OAX1104LB

Sevelius, J. OAD2104

Sevrukaite, R. OAD3605

Shah, N. OAB2304, OAC2806LB

Shah, P. OAC1903

Shah, S. A. OAC0705

Shahmanesh, M. OAX1102LB

Shaikh, M. W. OAA1002

Shalit, P. OAB1406LB

Shankaran, S. OAA1002

Sharma, M. OAC1903, OAE0505

Sharma, S. OAF3504

Sharp, A. OAX1103LB

Shaughnessy, M. OAB1406LB, OAX1106LB

Shayna, OAD3905

Shea, S. OAA1502

Sheira, L. OAC2202

Shen, X. OAA3006LB

Shenoi, S. OAD3605

Shepherd, B. E. OAB2302

Sherman, A. OAA3004

Sherman, C. OAC2805

Sherwood, J. OAX1103LB

Shi, Y. OAE0306LB

Shihadeh, F. OAB1406LB, OAX1106LB

Shinde, A. OAB4105

Shirobokov, V. OAB2305

Showalter, C. OAE0302

Shumskaya, N. OAD3605

Shunmugam, M. OAD2102

Siame, M. OAE3102

Sibiya, P. OAD0802

Siegler, A. OAC1906LB

Siika, A. OAX1105LB

Sikazwe, I. T. OAX1104LB

Silsorn, D. OAA2704

Silva Carvajal, H. H. OAF1204

Silva de Castro, I. OAA3003

Silva Rodrigues, D. OAE0305

Silva Torres, T. OAB2302, OAC4202

Simelane, W. OAE3103

Simukonda, K. OAD3304

Sin, L. OAA3005

Sinclair, G. OAB1405LB

Sindano, N. OAA1004

Sineke, T. OAE2905

Sing'oei, V. OAB2304

Singh, A. OAA1506LB

Singh, J. OAC0206LB

Singh, Y. OAD0805

Sinha, P. OAA1502

Sinyange, N. OAC1904

Sinyangwe, G. OAE3102

Siriwardana, R. OAD1805

Sitti, L. OAE0506LB

Sivasubramanian, M. OAD2102

Sivile, S. OAE3102

Skaathun, B. OAD0406LB

Slabbert, M. OAD1804

Slim, J. OAB1406LB

Smit, J. OAD0802

Smith, F. OAB2003

Smith, L. OAA2704

Smyrnov, P. OAD0406LB

Snyder, K. OAA1506LB

Soares, F. OAE2902

Sobolieva, Y. OAF2503

Soder, R. OAA1506LB

Sokhela, S. OAB3406LB

Soko, E. OAD3304

Sommerfeldt, S. OAD3305

Somphoh, Y. OAC2204

Somsouk, M. OAA3806LB

Soomro, A. A. OAC0705

Soto‐Torres, L. OAD0805

Sousa Ribeiro, G. A. OAE0305

South, A. OAX1105LB

Spann, K. OAA2705

Spener‐Gomes, R. OAE0305

Spinelli, M. OAF3204

Spire, B. OAC1905, OAD0403

Spittle, B. OAX1105LB

Spreng, R. OAA3006LB

Spyer, M. J. OAB2306LB

Squibb, M. A. OAC1306LB

Srichau, S. OAD0405, OAE2606LB

Srimuang, T. OAE2904

Sripanidkulchai, K. OAC2204

Sriplienchan, S. OAB2304

Ssemwanga, D. OAB1402

Stäger, S. OAA2703

Stamm, L. M. OAB1405LB

Starr, M. OAC0206LB

Steffy, T. OAB4104

Stern, J. OAA1504

Stewart, M. OAF3203

Stocker, H. OAB0106LB

Stoové, M. OAC4203

Strehlau, R. OAB0103

Stringer, J. OAA1004

Strong, C. OAC4204, OAE0502

Su, Y.‐C. OAA1003

Sudhakaran, A. OAB0103

Sued, O. OAB2005, OAB4103

Sugarman, J. OAD0805

Suh, J. S. OAB0903

Sule, A. OAD3302

Suleiman, I. OAD3302

Sullivan, P. OAC1906LB

Sumagaysay, M. OAE3104

Sumalu, S. OAD0405, OAE2606LB

Sundararaj, M. OAC2203

Suprasert, B. OAF3504

Supraset, B. OAC2202

Surafel Abay, E. OAD0804

Sutter, K. OAA1505

Suttner, C. OAB2004

Swan, T. OAF1203

Swetha, U. OAD3905

Symington, A. OAF3202

Szubert, O. OAF3503

### T

Taddee, N. OAE2904

Tagarro, A. OAA3803

Tajebe, F. OAA2703

Takalani, A. OAA3004

Tamuno, F. OAD3302

Tan, A. OAA3804, OAE2602

Tan, R. K. J. OAE2602

Tan, T. S. OAA2706LB

Tang, H. OAE0306LB

Tang, H.‐J. OAA1003

Tanser, F. OAF2506LB

Tarquis Medina, M. OAA3002

Tauzin, A. OAA2703

Teague, A. OAB3402

Teixeira, F. OAE0305

Tejan, M. OAB2003

Tembo, Z. OAC1306LB

Tepeka, A.‐G. OAC1306LB

Terán, R. OAD2403

Terlikbayeva, A. OAD2402

Terry, S. OAA1502

Terto Junior, V. OAF0602

Thedinger, B. OAA1506LB

Thepbinkarn, A. OAE2606LB

Thiam, S. OAF3206LB

Thielen, A. OAA1505

Thomas, K. OAC1903

Thombrayil, A. OAB4106LB

Thonyiwa, V. OAC1306LB

Thormann, M. OAB4103

Thu, M. OAF2504

Thy, K. H. OAD0405

Tiago Bernardo de Matos, A. OAC1302

Tian, J. OAD3602

Tiberi, O. OAE2605

Tiemessen, C. T. OAA3803

Timm, J. OAA1505

Tippett Barr, B. A. OAB2002

Tlagadi, A. OAD0802

Tng, C. OAC4203

Tobar Robalino, R. R. OAB4103

Todd, L. OAE2603

Toeque, M.‐G. OAC2805

Toh, H. S. OAA1003

Tolbert, W. D. OAA2703

Tomar, S. OAD0406LB

Tomaras, G. OAA3004

Tophong, S. OAC2204

Tor, E. OAD3306LB

Townsley, S. M. OAA2704

Traeger, M. OAC4203

Tragonlugsana, N. OAA2704

Traoré, P.‐W. H. B. OAB2305

Traudt, T. OAA1504

Trefiglio, R. OAC2803

Tremblay, C. OAB2305

Tressières, B. OAB0102

Tressler, R. OAA1502

Trilling, M. OAA1505

Trottier, B. OAB2305

Truong, H.‐H. M. OAC0204

Tsai, A. C. OAD2102

Tsai, Y.‐S. OAA1003

Tsakama, J. OAC1306LB

Tshivhase, V. OAE0505

Tsitsi, J. M. L. OAB4106LB

Tuhebwe, D. OAC0202

Tumusiime, V. OAB1402

Tun, M. S. OAF2504

Tupinambás, U. OAE2902

Turkova, A. OAB2306LB

### U

Uamba Tualufo, S. V.d. C. OAE2605

Udanwojo, D. OAD3302

Ughade, S. OAB4105

Ugochukwu, E. J. OAD3603

Ukoha‐Kalu, B. O. OAD3603

Ukor, M. OAD3302

Umechinedu, R.‐D. OAD3302

Urbano, E. OAA3002

Urio, R. OAB0104

Urujchtchairut, P. OAC2204

Usman, K. OAD2404

### V

Vélez Agudelo, Y. OAD0404

Valdez Ramalho Madruga, J. OAB2302

Valencia Gil, R. E. OAF1702

Valencia, J. OAB3403

Vambe, D. OAB4104

van Assen, M. OAB3403

Van Dam, C. OAB1405LB

van der Molen, J. S. OAB2004

van der Ploeg, S. OAF0602

van Eygen, V. OAB1402

van Gerwen, O. OAC2806LB

Van Grevenynghe, J. OAA1005

Van Huizen, R. OAD0403

van Widenfelt, E. OAE3702, OAB4106LB

van Wyk, J. OAB0106LB

van Wyk, T. OAB4106LB

Vanderpuye, N. A. OAD3906LB

Vanhamel, J. OAD3303

Vannappagari, V. OAC2805

Variava, E. OAB2306LB, OAB4106LB

Varma, R. OAC4203

Vasan, S. OAB2304

Vasantiuppapokakorn, M. OAC2204

Vasireddy, V. OAE3106LB

Vasylyeva, T. OAD0406LB

Vazquez Bello, L. OAB2005

Vazquez, M. J. OAB3405

Velasquez, G. OAC2202

Veloso, V. OAB0902, OAB2302, OAC2803

Venter, F. OAE0504

Venter, W. F. OAB3406LB

Venuto, C. S. OAA1502

Vera Vargas, A. OAF2505

Vera, A. OAD2403

Vera, J. H. OAB1402

Veras, M. A. S. M. OAD2104

Verhagen, D. OAB4106LB

Vieira Gratão Cordeiro, E. R. OAE0305

Vieira, V. A. OAB0103

Viens, H. OAE2603

Vilchez, M. OAB3403

Villanueva, M. OAA1002

Villanueva, S. OAC2205

Villanueva, S. V. OAF3202

Villapando, A. J. OAE3104

Villaran, M. OAA3004

Vimonpatranon, S. OAA2704

Violari, A. OAB2306LB

Visser, J. OAC2804

Vo Quang, E. OAC0706LB

Vujcich, D. OAC4203

Vulesevic, B. OAB2305

### W

Wachinger, J. OAF2506LB

Wafula, S. OAC0703

Wagner Cardoso, S. OAB2302

Wagude, J. OAB2006LB

Wajasuwan, N. OAE2904

Wake, R. M. OAB4106LB

Waldron, E. M. OAD0805

Walker, A. OAA1505

Walker, S. OAX1105LB

Walsh, A. A. OAA3002

Wamalwa, D. OAB2006LB

Wang, D. OAB4106LB

Wang, G. OAE0306LB

Wang, H. OAD0402, OAX1106LB

Wang, K. OAB0106LB

Wang, L. OAA3804

Wang, R. OAB0106LB, OAB2306LB

Wango, G. OAE3705

Wapling, J. OAC0206LB

Waratworawan, W. OAD0402

Ward, A. B. OAA3004

Ward, A. R. OAA1502

Watanabe, D. OAX1106LB

Watson, D. OAD0803

Watya, S. OAC0202

Watyaba, B. OAB0104

Wayburne, L. OAB2004

Webb, E. OAX1102LB

Webel, A. OAB2303

Wei, C. OAD0402

Weiner, D. OAA3004

Weiner, D. B. OAA3002

Weiser, S. D. OAC0203

Weissman, D. OAA3006LB

Weissman, S. OAD3302

Wellington, T. OAA3003

Wells, L. OAE4006LB

Wensing, A. OAA1505

Were, D. OAC1903

Wertheim, J. OAD0406LB

West, B. OAD2402

Westin, M. OAE2902

Whitlock, G. OAB0106LB

Wilkin, T. OAA1502

Wilkinson, A. OAC4203

Williams, J. OAD2106LB

Williams, R. OAF3204

Williams, W. B. OAA3006LB

Williamson, C. OAX1104LB

Wilson, D. P. OAB4106LB

Wilson, E. OAC2202

Wilson, E. C. OAF3504

Wilson, M. OAB2303

Win, T. OAF2504

Winchester, V. OAD2105

Wong, P. OAC2806LB

Woode, E. K. OAA3003

Woods, J. OAB3406LB

Wools‐Kaloustian, K. OAC0204

Workowski, K. A. OAB1406LB

Wosornu, S. K. OAE4002

Wu, H.‐J. OAC4204, OAE0502

### X

Xi, M. OAD3305

Xie, J. OAE0306LB

Xu, H. OAE0306LB

Xu, X. OAB1405LB

Xulu, M. OAB4106LB

### Y

Yalley, C. OAE4002

Yan, H. OAE0306LB

Yang, D. OAA2703

Yang, J. OAB2003

Yao, D. C. E. OAF3205

Yao, Y. OAE0306LB

Yawe, I. OAB1402

Yazdanpanah, Y. OAD0403

Yeo, R. Q. OAE2602

Yew, K. C. OAE2602

Ylannoutsos, C. T. OAC0204

Yonko, E. OAD3602

You, Q. OAD3606LB

Young, A. T. OAE2603

Young, F. OAD1805

Yu, C. OAA3004

Yu, K. OAC1903

Yu, X. G. OAA2706LB

Yusoff, Y. OAD1805

### Z

Zahabu, E. J. OAE2604

Zamora, A. OAB2003

Zamzu, M. OAC1304

Zang, X. OAC1906LB

Zapolska, N. OAE1602

Zare, Z. OAA3002

Zaviryukha, I. OAD3605

Zea, A. OAD0404

Zeziulin, O. OAD3605

Zhang, B. OAB3403

Zhang, C. OAB0105LB

Zhang, G. OAE0306LB

Zhang, J. OAE0306LB

Zhang, L. OAD3606LB

Zhang, N. OAB1403

Zholnerova, N. OAD2402

Zhou, Q. OAE0306LB

Zimmermann, L. OAC1905

Zougouri, L. OAD3903

Zyambo, K. OAE3102

